# Detecting anomalies in graph networks on digital markets

**DOI:** 10.1371/journal.pone.0315849

**Published:** 2024-12-23

**Authors:** Agata Skorupka

**Affiliations:** SGH Warsaw School of Economics, Warsaw, Poland; Agricultural Research Organization, ISRAEL

## Abstract

The study examines different graph-based methods of detecting anomalous activities on digital markets, proposing the most efficient way to increase market actors’ protection and reduce information asymmetry. Anomalies are defined below as both bots and fraudulent users (who can be both bots and real people). Methods are compared against each other, and state-of-the-art results from the literature and a new algorithm is proposed. The goal is to find an efficient method suitable for threat detection, both in terms of predictive performance and computational efficiency. It should scale well and remain robust on the advancements of the newest technologies. The article utilized three publicly accessible graph-based datasets: one describing the Twitter social network (TwiBot-20) and two describing Bitcoin cryptocurrency markets (Bitcoin OTC and Bitcoin Alpha). In the former, an anomaly is defined as a bot, as opposed to a human user, whereas in the latter, an anomaly is a user who conducted a fraudulent transaction, which may (but does not have to) imply being a bot. The study proves that graph-based data is a better-performing predictor than text data. It compares different graph algorithms to extract feature sets for anomaly detection models. It states that methods based on nodes’ statistics result in better model performance than state-of-the-art graph embeddings. They also yield a significant improvement in computational efficiency. This often means reducing the time by hours or enabling modeling on significantly larger graphs (usually not feasible in the case of embeddings). On that basis, the article proposes its own graph-based statistics algorithm. Furthermore, using embeddings requires two engineering choices: the type of embedding and its dimension. The research examines whether there are types of graph embeddings and dimensions that perform significantly better than others. The solution turned out to be dataset-specific and needed to be tailored on a case-by-case basis, adding even more engineering overhead to using embeddings (building a leaderboard of grid of embedding instances, where each of them takes hours to be generated). This, again, speaks in favor of the proposed algorithm based on nodes’ statistics. The research proposes its own efficient algorithm, which makes this engineering overhead redundant.

## Introduction

### Problem statement

The emergence of the Internet as a trade and communication channel significantly reduced barriers of entry to the market [1–3]. This, connected with the ease of automatic content generation, creates a threat of anomalous activities: establishing fake identities, performing fraudulent transactions, or spreading false information [4–8]. This, in turn, poses a substantial danger to market efficiency for both market actors and regulators, hence a need to identify those threats. The article focuses on detecting users performing such activities and defines anomaly as being a bot or displaying fraudulent behavior, depending on the market analyzed. It may use the terms anomalies and anomalous behavior interchangeably. Due to limitations related to length and depth of analysis, this study is limited to supervised problems, leaving unsupervised learning aside as a future research area.

The digital market is defined as a market relying on an online presence rather than a physical one [2, 3]. This study analyzes two subtypes of peer-to-peer online markets where trust is crucial: cryptocurrency markets and social networks. The latter can also be defined as the information market because information (data) is an essential medium of exchange between the users in those networks [1]. The choice of these markets is determined by the fact that they are subject to a relatively low regulation burden and, therefore, primarily rely on trust between users. This makes them especially prone to fraudulent activities.

As the digital market relies on an online presence rather than a physical one, a significant reduction in transaction costs is possible [2, 3, 9]. Transaction costs include costs of entry to the market and verification, often by simplified identity verification, if any—all is needed is access to the Internet and e-mail account. Therefore, there also emerged a possibility of using fake and automated accounts to forge ratings, opinions, or even transactions [10, 11]. These actions are hazardous in markets with low levels of regulation and significant role of trust, such as information and cryptocurrency markets. The constantly growing number of users and the possibilities of automatic content generation using generative AI chatbots (e.g., ChatGPT-4 model) make this threat even more prevalent.

Catalog of anomalous behavior may contain the black market for buying likes, follows, or even opinions [4–8], disseminating disinformation [12, 13], political conspiracy [14], automatically posting fake reviews or opinions [15–17], seeding discord or polarizing discussions [12, 18] as well as influence behaviors of other market players (i.e. voting pattern or herd behavior on financial markets; see [1]. The literature also describes these activities as computational propaganda [12]. There is also a possibility of conducting explicitly fraudulent or manipulative (e.g., bot-driven) transactions on financial markets using fake identities [19].

Analyzed markets can be represented as graphs, where vertices represents users and edges—interactions between them. Therefore, graph methods for anomaly detection can be used and compared to more traditional ones. The state-of-the-art from the literature uses graph embeddings [20, 21] where embedding is a mapping of the whole graph to the vector space with the help of linear algebra, random walks, and deep learning techniques. Unfortunately, they are not always computationally efficient, especially in the case of larger graphs. This study looks forward to contributing to this research area by proposing an algorithm suitable for modern economy needs, including data size, by proposing its own algorithm based on efficient node statistics.

The outcomes of the following article may serve as an objective benchmark for comparing other supervised state-of-the-art algorithms due to the usage of labeled datasets. Fraudulent behavior is a subset of anomalous behavior, where fraud has already been determined (e.g., by judicial order, [22]. The fact of being unanimously determined is still a minority in such cases: most of them are justified convictions [23]. Consequently, most of the research focuses on unsupervised techniques or generating artificial anomaly samples, which influences the objectivity of the results and makes results difficult to compare. The presented research combats these anomaly-specific dataset disadvantages, serving as a reliable benchmark for future comparisons.

### Literature overview

Traditional bot detection methods can be divided into behavioral-based and network-based methods [16, 17, 24]. The former is based on text features from reviews or social media posts [25–32] or timestamps [33–35]. It is doubtful whether the conclusions from these studies still hold, as it is founded on datasets from before advancements in text generation (generative AI models). These technological developments made automatic content creation more accessible and advanced, up to the point that it is impossible to differentiate between automatic and human-generated text even for the human eye, not to mention machine learning models. On the other hand, graph structure is not as easy to simulate [20, 21], meaning it is more difficult to do than one click of the mouse. It requires a more careful and coordinated effort. In the case of forging, whole troll farms are required because graph structure depends on the action of vast groups of users. This, in turn, makes the barrier of entry more prohibitive.

On the other hand, network-based techniques are based on belief propagation [36, 37], node ranking [38–40], and iterative learning, for example, assigning scores regarding fairness of rating based on earlier defined rating consensus [16, 17] or expected behavior. These include random walk algorithms and others based on the node’s neighborhood [41]. There are numerous drawbacks of behavioral methods, such as related to ethical concerns, generated the current progress in text-generating technologies, as well as the fact that text data is not always available (e.g., in the financial markets) or generalizable (e.g., languages or the emergence of new topics with time, such as COVID-2019 or specifics of particular elections). Taking this into account, network-based techniques are a promising direction of research [20, 21, 42]. Although the usage of graph features and embeddings has already been presented in the literature [20, 21], it remains a relatively little exploited area and bears some significant limitations (e.g., graph size). This study aims to cover this gap.

Unlike traditional methods that rely primarily on textual data, graph-based methods utilize the relational dynamics between entities, offering a more nuanced detection of irregular patterns that indicate anomalous activities [43]. However, most of the graph analysis in the field of anomaly detection focuses on citation and product networks [41, 45–46].

Since the concept of cryptocurrency is relatively new, anomaly detection in this area is still developing. [47] surveys anomaly detection methods in blockchain networks and indicates that although cryptocurrency fraud detection has been investigated in the literature [33, 48–50], graph methods are not extensively used there. To the author’s knowledge, only several papers were published in this area, primarily focusing on deep learning techniques using graphs [51–53]. Therefore, the proposed research contributes to this relatively new field of research, focusing on non-black box graph techniques.

Furthermore, this article shows how graph-derived data can improve the model performance and reliability, especially compared to text data or other user characteristics. Using features based on user characteristics, such as personal data or text they posted, is always associated with a risk around users’ privacy and ethics [14]. Furthermore, these features are characterized by many disadvantages, such as engineering overhead (text data needs to be highly processed, including time-consuming model training), lack of generalizability (new topics outside training sample appearing, e.g., COVID-19), lack of property of being time or language-invariant, as well as low availability in some networks [20, 21]. Furthermore, with the newest technology advancements, such as generative AI models (e.g., ChatGPT, Anthropic Claude), automatic text generation has allowed the forging of human-written text so that even the human eye cannot distinguish them. Therefore, it is more and more difficult to use text data as a reliable input to models, and a need arises for a more robust and stable way of anomaly detection.

This research contributes to the literature on graph algorithms by proposing its own computationally efficient algorithm. Most existing literature focuses on using graph embeddings as predictors to machine learning models, as it is widely recognized that they capture the essential information about nodes [20, 21]. Their popularity is also attributed to their complexity and usage of deep learning techniques underneath, contrary to simple nodes’ statistics, e.g. their importance measures (such as degree centrality). However, these reasons make embeddings computationally inefficient, as the study shows. This is the case, especially with large graphs, which are more prevalent nowadays due to an ever-growing user base resulting in the growth of graph size. This speaks in favor of nodes’ statistics, which are based on, as the name suggests, statistics and, hence, can be computed significantly faster. Node statistics also have another advantage: better explainability and interpretability instead of serving as a black-box algorithm. This research proposes its own algorithm based on node and neighborhood statistics.

### Research goal

The research attempts to find the best-performing and scalable method for anomaly detection tasks while ensuring users’ anonymity and protecting their personal data. The study argues that node statistics, underestimated in the literature to the benefit of deep learning algorithms, may not only perform as well as behavioral data input and graph embeddings, being state-of-the-art solutions but also save engineering and computation time.

On that basis, the article proposes a new computationally efficient algorithm for graph anomaly detection based on nodes’ neighborhood statistics and compares its performance to state-of-the-art methods (embeddings). The computational gain from using this procedure ranges from making infeasible problems feasible (e.g., the TwiBot-20 dataset use case) to dozens of hours of saved computation time (other datasets analyzed in the study).

Time-saving also manifests in the second dimension, which is engineering overhead. There is no straightforward way, not even a rule of thumb, to determine in advance which embedding type should be used for a given problem and in which dimension. The only way is to compute several embeddings in several dimensions and check which one is best suited to the specific use case by comparing the desired metrics on the test dataset, which is time-consuming (considering various algorithms and possible dimensions, where generating each one of them may take several hours). Using node statistics solves this problem: one can prepare a set of features within minutes without extensive engineering overhead and achieve stable results. Furthermore, contrary to embedding algorithms and output values, they allow for better model explainability and interpretability.

Conclusions drawn from the following research and the tools presented may help detect anomalies in digital markets by the market regulator or third parties such as NGOs. The term regulator can describe legislative organs and investigative or supervising bodies, such as the US Securities and Exchange Commission. Potential beneficiaries may also be entities belonging to the banking or cybersecurity sectors and willing to detect anomalous activities in their transactions or network traffic. Depending on the applicable law and the regulator’s strategy, conclusions may be used for informative and prohibitive purposes. Formalized anomaly detection methods using graphs presented in the article may help implement more robust consumer protection and reduce political risks while increasing market efficiency and transparency on the Internet.

## Materials and methods

### Datasets

Experiments are conducted using three publicly accessible graph-based datasets: TwiBot-20, Bitcoin OTC, and Bitcoin Alpha. The following feature sets are extracted for each dataset: node statistics (see Table 2) and graph embeddings (see Table 3). In addition, TwiBot-20 is supplied with text data. The lack of text data for Bitcoin datasets stems from their characteristics. In reality, most digital markets are not supplied with these data or are not publicly available (privacy concerns).

**Table 2:** Node statistics for TwiBot-20, Bitcoin OTC and Bitcoin Alpha datasets.

degree centrality	number of neighbors (edges) a node has;
closeness centrality	a measure of node importance based on the criterion of how close a given node is, on average, in relation to other nodes, i.e. average of the shortest path length (number of edges) from the node to every other node in the graph.
betweenness centrality	a measure of node importance based on the number of the shortest paths between other nodes that pass through a particular vertex. This measure is based on the idea of identifying the nodes that are most often included in the shortest path between other nodes. As a useful analogy to picture the meaning of this measure can serve the most frequently visited road intersection [58].
harmonic centrality	another variant of closeness centrality, but based on the sum of inverse of the distances rather than distances themselves in order to take unconnected graphs into account.
pagerank centrality	a recursive measure based on the assumption that a node is as important as the nodes referring to it, i.e. based on the number of incoming interconnections and the importance of the sources themselves (Page, 1999). It was designed for the Google search engine and is still used there.
eccentricity centrality	a metric based on the longest of the shortest paths between the node and all other nodes in the graph, reflecting how easy the node can be reached by other nodes.
hub score	an importance measure based on the number of outgoing nodes
authority score	an importance measure based on the number of incoming nodes
Burt’s constraint	also referred to as a constraint, a metric measuring how much a given node is related to nodes to which its neighbors are related.
local clustering coefficient	the likelihood that the neighbors of a given node are connected as well.

**Table 3:** Graph embeddings used for TwiBot-20, Bitcoin OTC and Bitcoin Alpha datasets.

name	type	description
DeepWalk	classical	analogous to word2vec, creates embedding for node instead of words, order of neighborhood is a context window
Node2vec	classical	variation of DeepWalk with parameterized random walk
Struc2vec	structural	generates a predefined number of random walks from each node in order to build a model predicting the probability of occurrence of a given node in a specific context + hierarchical clustering
RolX	structural	generates binary features structural properties and applies non-negative matrix factorization to map each node to vector representation
ReFeX		binary features based on structural properties
Graph Convolutional Networks (GCN)	structural	each node represented as a computation graph resembling a tree and convolution operation is performed and the weight matrix is learned

#### TwiBot-20

The TwiBot-20 dataset is a representation of the Twittersphere prepared by [54]. It is characterized by a directed graph where nodes are Twitter users, and the edge represents the relationship of following, which is a Twitter analogy for a subscription. Therefore, TwiBot-20 is a graph with 229,573 nodes, information from the user’s profile they decided to share, 200 most recent user posts (also referred to as tweets), and labels indicating whether a particular user is a bot.

In the following article, the largest connected component of the graph, characterized by 156,115 nodes, is analyzed. The anomaly on this market is defined as being a bot. Only 6 percent of the dataset is labeled, i.e., supplied with a non-empty value of the feature representing the fact of being a bot. Such a ratio is typical for anomaly detection datasets due to difficulties with unambiguously determining anomalous behavior, as outlined in Introduction and Problem setting. To mitigate this constraint, embeddings as well as node features were prepared on the basis of the whole graph, but for the training and testing only labelled nodes wereused.

Obviously, with this number of users, the TwiBot-20 dataset does not represent the whole Twitter network. It was obtained using sampling designed using a specific algorithm to ensure representativeness [54]. Even though it might be perceived as a dataset drawback, in this case, it will serve as robustness check for the graph-based models, i.e. whether conclusions from graph derived metrics still hold when the data is distorted/uncomplete.

The sampling process is as follows. First of all, most of the approaches in the literature narrow down social network sampling to one specific topic of users’ posts, which limits information and generalization of conclusions, including the reliability and stability of model results [54]. TwiBot-20 is not using assumptions of this type, at the same time diversifying users more extensively. Instead of arbitrarily narrowing down the user base, the breadth-first search was implemented starting from different root nodes—users, following their ‘follow’ relationship up to layer 3 of the neighborhood. When on the one side lack of completeness of the graph may be perceived as a drawback from the generalization perspective, it allows for computational feasibility, ensuring user diversification of geographical regions, as well as domains of user posts (referred to as tweets) at the same time. In addition, the dataset contains user profile characteristics that users decided to share and the most recent 200 tweets.

Another drawback of the TwiBot-20 dataset, as well as other datasets derived from social networks, is the fact that there are no objective ground truth labels provided. The fact of being a bot is usually not definitely determined even by the company hosting the social network, or, even if so, not shared publicly. A recent example may be the case of Twitter acquisition by Elon Musk and his doubts around number of bots there (see e.g., Duffy, C., Fung, B., “Elon Musk commissioned this bot analysis in his fight with Twitter. Now it shows what he could face if he takes over the platform”). Therefore, all that can be done is manual annotation using human inference, or using machine learning algorithms to label the datasets. Both approaches are biased: first, with human assumptions and lack of accuracy, and second, with the fact that the anomaly detection algorithm is trying to solve the task prepared by another algorithm, not objective reality. The authors of [54] hired independent contractors to manually annotate the tweets, with five annotators assigned to each Twitter user. There were only two requirements for annotators: being an active Twitter user and using guidelines specifically prepared by researchers, containing findings from the previous literature. Examples of such criteria are lack of originality in posts, automated activity, frequent external links, and irrelevant URLs, and repeated content [55].

Having said that, there is a high probability that annotators labeled users using information visible on their profile. The users’ profile characteristics contained the following information: whether tweets of the user are private, count of followers, count of users following, number of lists the user is a member of, the fact of being a verified user, the fact of having a profile picture and the fact of being interested in a specific domain. Especially including the fact of being a verified user to a set of predictors as in [54] seems doubtful, given that each verified user was considered a non-bot. This may lead to overestimated model metrics and a lack of model generalizability. To take the annotation bias into account, the user’s profile characteristics are not incorporated into anomaly detection model features in the following research.

**Fig 1:**
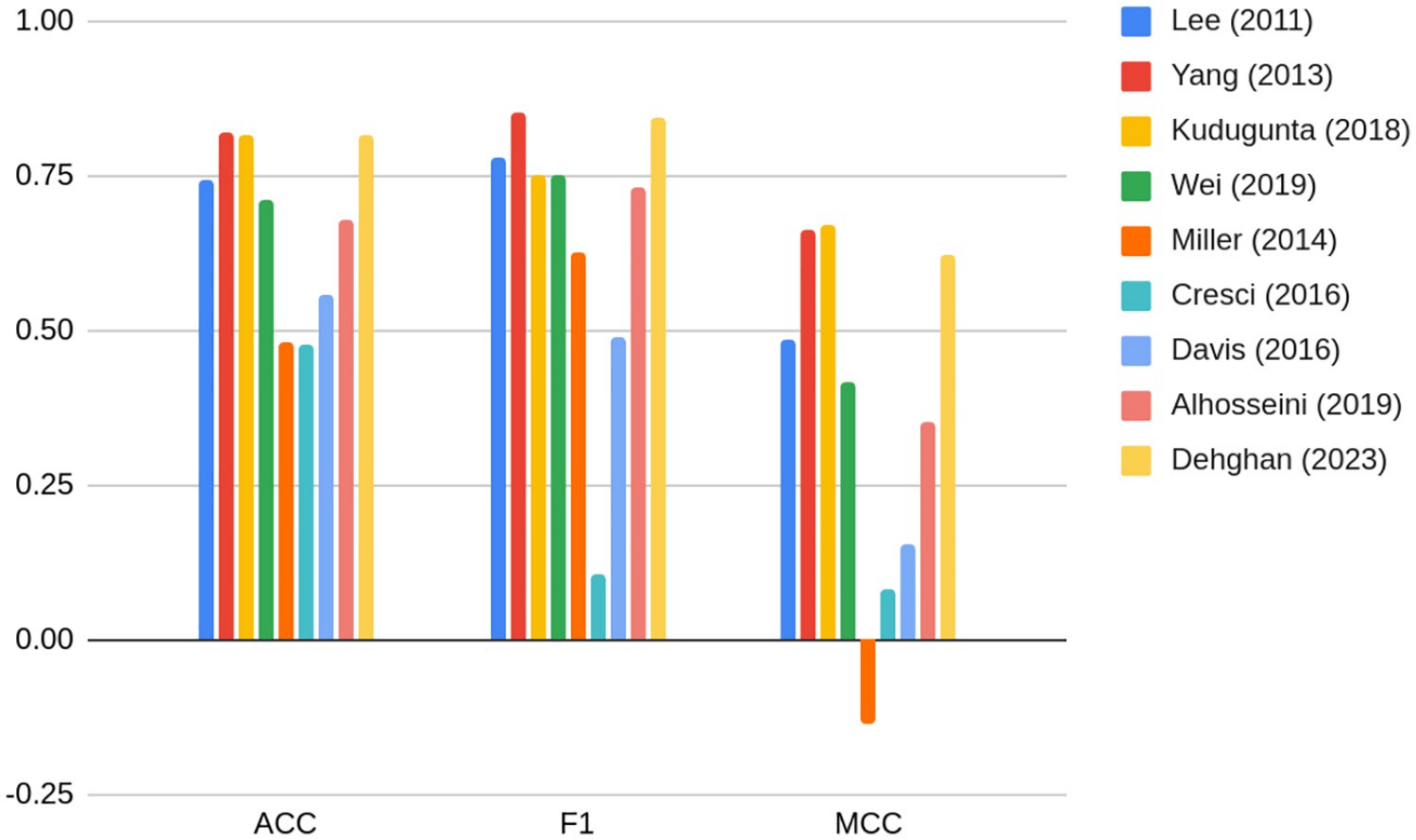
Bot detection on the TwiBot-20 dataset using various methods presented in the literature performance comparison including accuracy, F1, and Matthew’s Correlation Coefficient. Source: [54].

Authors of [54] investigate various methods of bot detection performance on TwiBot-20 datasets. Metrics for this exercise are provided on Fig 1.

Metrics presented in Fig 1 may serve as a benchmark for the following analysis, although with certain caveats. There is a need to bear in mind that there is a high probability that due to the manual annotation, the user’s profile data may be the strongest predictor of being a bot, especially a feature indicating whether the user is verified. In this research this set of features was abandoned in the modeling due to the fact of possible annotation bias, as well as analyzing performance of graph features in comparison with text data only.

Furthermore, the graph data from digital markets are rarely supplied with user profile information due to market specifics or privacy concerns. Social networks may be an exception in this matter, however, there was a visible shift towards stronger data privacy there in recent years as well, imposing limitations on public data extraction. Making inferences based on personal data, or even storing and processing them, is ethically, if not legally, doubtful. The purpose of the following research is to contribute to strengthening market participants’ safety, not the opposite. Therefore it focuses on graph-derived features, where all that is needed is information about connections of entities, without any knowledge about their identity.

#### Bitcoin OTC and Bitcoin Alpha

In both Bitcoin datasets, graphs represent the whole network of both trading platforms—OTC and Alpha, which is a major advantage of those datasets. This means that no sampling is involved. On the contrary, full information about each node’s relationships is available. These datasets consist of 5,881 and 3,783 nodes, where 5 and 6 percent respectively are supplied with a feature representing the fact of being a benign or fraudulent user. This, as well as more detailed statistics were presented in Table 1.

**Table 1:** NLP feature set variables for TwiBot-20.

tweets_no	number of tweets posted by user, maximum 200 recent tweets due to dataset limitations,
av_tweet_len	average tweet length (number of characters),
std_tweet_len	standard deviation of tweet length (number of characters),
links_no	number of hyperlinks in user’s tweets overall,
links_per_tweet	average number of hyperlinks in user’s tweets,
mentions_no	number of mentions of other users in user’s tweets overall,
mentions_per_tweet	average number of mentions of other users in user’s tweets,
no_langs	number of languages dominant in user’s tweets,
no_odd_languages	number of odd languages in user’s tweets; odd language is defined as present in less than 10% tweets overall,
perc_en	percentage of user’s tweets in English,
perc_legit	percentage of user’s tweets in legit language; legit language is defined as present in more than 10% of a given user’s tweets overall,
av_sent	average sentiment score, calculated only for tweets in English,
std_sent	standard deviation of sentiment score, calculated only for tweets in English,
positive_sent_perc	percentage of user’s tweets with positive sentiment, calculated only for tweets in English.

An anomaly is defined on this market as a user described as fraudulent by other market paricipants. It is worth mentioning that a fraudulent user can be both a bot and a real person. Both Bitcoin datasets are directed weighted graphs where the node represents a user, whereas the edge denotes the fact of being rated [16, 17]. Edge weights depict the value of the rating, which can take place only after a transaction. Therefore, connections between the nodes may serve as a proxy for transactions. Due to the cryptocurrency market’s specifics, no further information such as user characteristics or text is available besides graph connections and labels, e.g. whether the given user is a benign user or fraudulent one. The latter is a definition of anomaly applicable to this dataset, assumed in this article. In this case, it is of secondary importance if the fraudulent accounts are automated bot accounts, or not, even though there exists a quite high probability of such a correlation.

Rating value, being an edge weight, can range from -10 to 10 and describes how well the transaction was performed by an assessed user, where -10 represents total distrust (transaction was fraudulent) and 10 total trust (transaction went perfect. Only users with a positive rating are allowed to rate others, and a rating may be issued only once for a given user. The rating is conditional to the fact that the actual transaction took place and can be updated later.

Therefore, one of the main advantages of Bitcoin datasets, apart from representing the whole network, is that they include a relatively objected ground truth, which is quite uncommon among graph datasets for anomaly detection due to difficulty of determining one in a definite (legal) way. This bears a lot of value regarding the reliability of conclusions and the possibility of objective algorithm comparisons. Ground truth was constructed as per [16, 17], by rescaling rating values from -1 to 1. Benign users are defined as platform founders, as well as users rated positively by them (at least 0.5). On the opposite, a user is considered fraudulent when he is rated at most -0.5 by the group of benign market actors [16, 17]. The rest of the users are determined as neither benign nor fraudulent and therefore hold a missing value in the respective column in the dataset.

**Fig 2:**
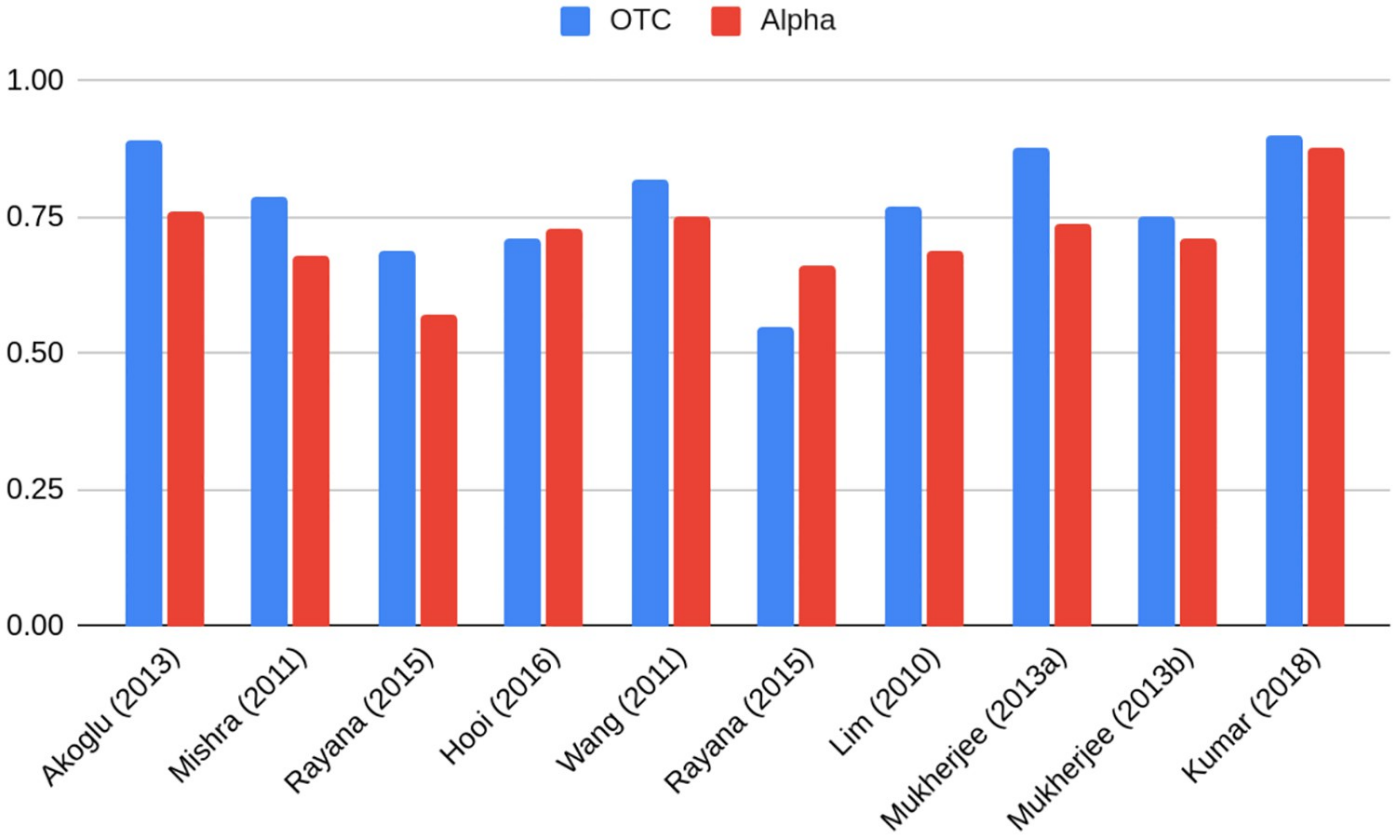
Performance of various fraudulent user detection algorithms on Bitcoin OTC and Bitcoin Alpha datasets, AUC metrics. Source: [16, 17].

Authors of [16, 17] presents an overview of the algorithms in the literature, benchmarking them on the Bitcoin OTC and Bitcoin Alpha datasets, using AUC metrics. An overview is presented on Fig 2 and can serve as a benchmark for anomaly detection model performance.

### Feature sets

The purpose of the experiment is to compare methods for anomaly detection, both in terms of performance and computation, to find the best one. In order to do this, three categories of models have to be compared:

- Based on text features (also defined further as Natural Language Processing, NLP)
- Based on graph embeddings
- Based on node statistics.

To compare methods in universal fashion and receive robust results, three datasets were examined. Therefore, three types of feature sets as outlined above were prepared for the TwiBot-20 dataset, and two for both Bitcoin OTC and Bitcoin Alpha datasets due to lack of NLP data. For the sake of clarity, feature sets are defined as input to the model. Each feature set serves as a predictor in a separate model for each of three datasets in order to determine the best performing one. For that purpose, F1 metrics is used as a criterion, as it is the metrics recommended for data with high percentage of unlabeled values. This makes it a good fit for specifics of anomaly detection based datasets.

All feature sets are prepared using Python software.

For TwiBot-20, text features have been defined as in [20] (see Table 1). For node statistics, a standard set of features proposed in the literature is used (see Table 2) generated using NetworkX library. Moreover, to create a more informed embedding and allow it to compete with other traditional embeddings, the study proposes to include information about node’s neighbor’s statistics. Hence, mean, standard deviation, minimum, and maximum was computed for each feature, taking into account various neighborhood orders, 1 to 4. Maximum value of 4 has been selected in the analysis as a result of experimentation and discovering that adding more neighborhood layers has not resulted in significant model improvement.

There is a need to answer the question which embedding should be used in the experiment to receive robust and generalizable results. There are a variety of algorithms proposed in the literature, as well as each embedding instance can be prepared in different numbers of dimensions. Literature distinguishes two types of embeddings [20, 21]: node (also referred to as classical) as well as structural embeddings. In classical embeddings, similarity is defined as node proximity in the graph. Structural embeddings, in turn, define node similarity as an equivalent role in the term of network structure and their relationship to other nodes, i.e. whether the node has a similar neighborhood structure (isolated node, star pattern, one neighbor, etc.). Structural equivalence is learned using deep learning techniques, therefore is more black box and more difficult to control while engineering.

For the sake of experiment, the following instances of embeddings were prepared using Python software and corresponding libraries: Node2vec, DeepWalk, Struc2vec, ReFeX, RolX as well as GCN (Graph Convolutional Networks). Their list and description is included in Table 3. Each embedding instance can be prepared in a given dimension, which may lead to variation in model outcomes. As per [20, 21], the following grid of dimensions was prepared for each embedding type (k): 4, 8, 16, 32, 64, 128. Struc2vec and GCN were not generated for the TwiBot-20 dataset (156,115 nodes) due to the limitations of Python implementation (out of memory error, even though using a machine with 96 RAM).

In order to check if compressing dimensions will result in increase in model performance, each embedding with dimension above 16 was compressed down to dimension 16 using two separate algorithms: Principal Component Analysis (PCA, [56] and Uniform Manifold Projection and Approximation (UMAP, [57]. UMAP compression was prepared with three different seeds due to its stochastic nature. Number of seeds and compression dimensions was chosen based on [20, 21].

This results in three categories of feature sets for TwiBot-20 and two categories of feature sets for Bitcoin data. Furthermore, the embedding subcategory consists of 6 separate feature supersets—different embeddings types. Each of them is prepared in 6 different dimensions (4, 8, 16, 32, 64, 128), 3 of which (those above 16) are a base for another feature set by being subject to compression to dimension 16. This has been done using two different methods, PCA and UMAP, so yields in two variants of feature sets. This results in an abundance of feature sets and quite a high number of models (74 for the TwiBot-20 dataset and 151 for both Bitcoin datasets). Author believes that only such a meta-analysis and careful examination of each and every embedding version will allow to compare methods in an universal manner and provide robustness and stability of the results, as well as account for randomness of embeddings compared to node based statistics. Author believes such a comparison, especially if assuming advantage of node based statistics, where random component is not present, should take randomness component out of the picture and take conservative approach for evaluation.

### Models

Finally, here is a question how to build a model for each feature set and dataset combination, aiming for maximum generalizability of results. To find the best model we applied the AutoML procedure using the Python h2o package. AutoML automatically examines a grid of models and hyperparameters to find the best one, taking away the tuning overhead and uncertainty around model score. Again, this experiment design is aimed at testing as many variants as possible to achieve unbiased and robust results, as well as removing random component from the comparison.

The implementation of AutoML applied in the analysis uses 5-fold cross-validation and prepares following models: Generalized Linear Model (GLM), XGBoost, and Random Forest with automatically tuned parameters, as well as ensemble models based on them. One ensemble class was best on the best-performing models, while the second one—was on the basis of all of the analyzed models. Gradient Boosting Machines and Deep Learning Neural Networks were excluded from the analysis due to their variability between runs in the used software implementation (even when using the same random seed). This has been done in order to ensure full reproducibility of the results. Having said that, each of 74 (TwiBot-20) and 151 (Bitcoin OTC and Alpha) models for each of three dataset is a best performing model chosen from a subset of dozens of models.

### Summarized procedure

For the sake of clarity, the overall procedure can be summarized as following steps:

1. Preparing feature sets for each dataset using python software (see next paragraph for details).
2. Building the bot/fraudulent user classification model for each dataset and feature set combination using python software and the following models:
  - XGBoost with default parameter settings,
  - h2o AutoML, applying automatic parameter tuning and choosing the best model out of the following model set:
    - XGBoost,
    - GLM,
    - Random Forest,
    - ensemble of all prepared models,
    - ensemble of best models in their class.
3. Choosing the best model for each dataset and feature set combination based on F1 metrics.
4. Preparing ranking of best models for each dataset based on F1 metrics.

In the course of step one, the following feature sets were prepared for each dataset—Bitcoin OTC, Bitcoin Alpha, TwiBot-20:

- node statistics (centrality measures: degree, closeness, betweenness, harmonic, pagerank, eccentricity, hub score, authority score, Burt’s constraint, local clustering coefficient) plus statistics of node’s neighbors of the first order (mean, standard deviation, minimum, and maximum);
- node statistics defined as above compressed to the dimension of 16 using following compression algorithms:
  - PCA,
  - UMAP,
- node statistics described above plus statistics of node’s neighbors of order up to the fourth level of neighborhood (mean, standard deviation, minimum, and maximum for each and every order of neighborhood from first to fourth);
- node statistics defined as above compressed to dimension of 16 using:
  - PCA,
  - UMAP,
- following graph embeddings:
  - DeepWalk,
  - Node2vec,
  - ReFeX,
  - RolX,
  - Struc2vec (for Bitcoin datasets),
  - GCN (for Bitcoin datasets).

Two latter algorithms could not be created for the TwiBot-20 dataset due to their computational limits for large graphs. On the other hand, node features based approach (the own algorithm proposed by the study) didn’t experience such problems and could be prepared within minutes for each dataset. Moreover, due to inability to determine the optimal size of embedding in advance, each embedding instance was prepared in the following dimensions (k) of the output vector: 4, 8, 16, 32, 64, 128.

- each embedding of the dimension of above 16 was additionally subject to compression to 16 dimensions using:
  - PCA,
  - UMAP,
- node statistics including statistics of neighbors of the first order and best performing embedding was subject to compression to 16 dimensions using:
  - PCA,
  - UMAP,
- node statistics including statistics of neighbors of first, second, third and fourth order and best-performing embedding was subject to compression to 16 dimensions using:
  - PCA,
  - UMAP,
- NLP features based on text data for TwiBot-20.

UMAP is a stochastic algorithm, therefore to account for its randomness, each dataset involving this procedure was prepared three independent times, using three different seeds. This was done in order to determine its stability. Another caveat is that Struc2vec and GCN were not created for the TwiBot-20 dataset due to implementation limitations, as outlined in the previous Subsection. This indicates that some types of embeddings cannot be easily created or created at all for bigger datasets, in contrast to node statistics.

To compare methods in universal fashion and receive robust results, three datasets were examined. Therefore, three types of feature sets as outlined above were prepared for the TwiBot-20 dataset, and two for both Bitcoin OTC and Bitcoin Alpha datasets due to lack of NLP data. For the sake of clarity, feature sets are defined as input to the model. Each feature set serves as a predictor in a separate model for each of three datasets in order to determine the best performing one. For that purpose, F1 metrics is used as a criterion, as it is the metrics recommended for data with high percentage of unlabeled values. This makes it a good fit for specifics of anomaly detection based datasets.

## Results and discussion

### TwiBot-20

**Fig 3:**
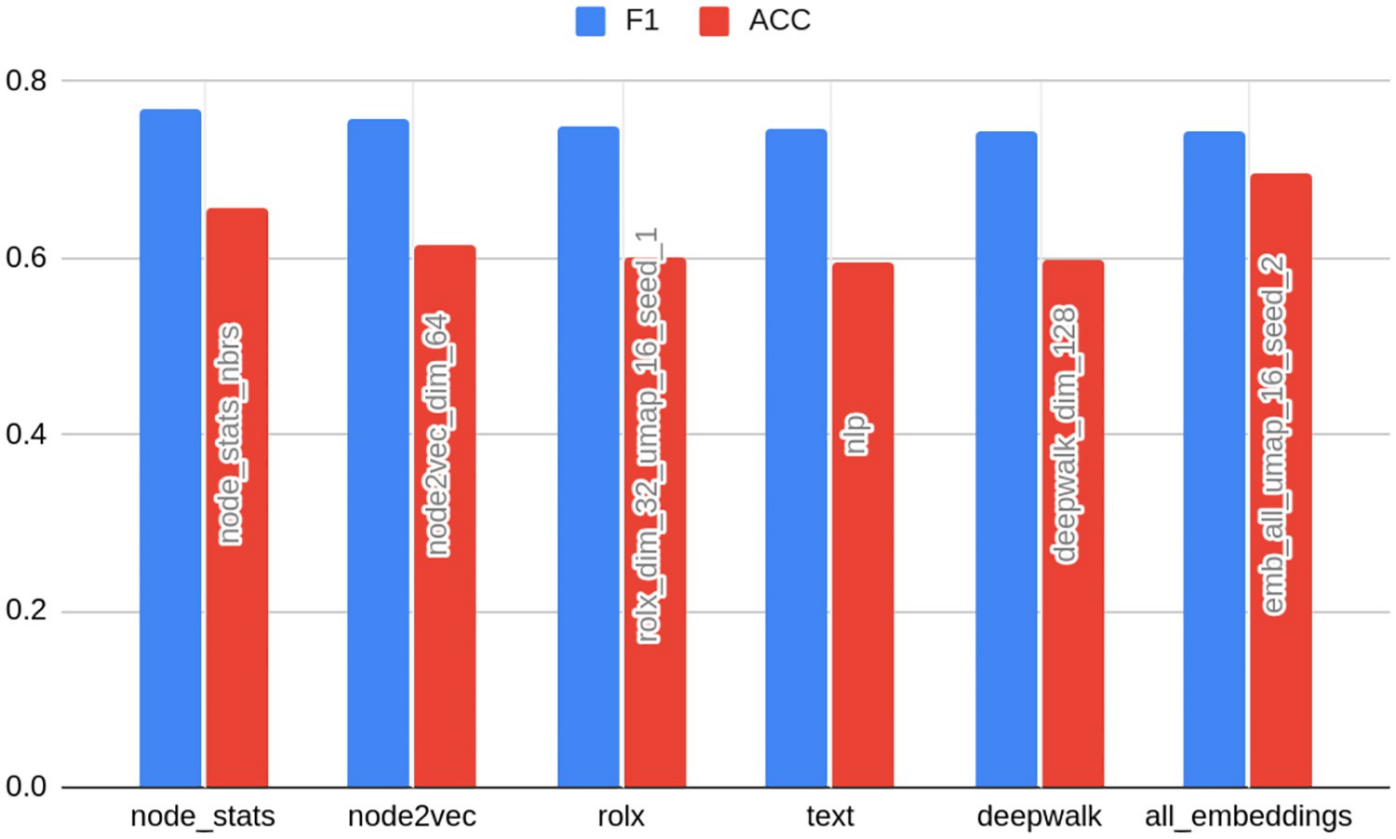
Results for TwiBot-20 dataset—best models in the class of embedding type. Source: own calculations.

For clarity purposes, Table 4 presents the results of the ten best models detecting bots on TwiBot-20 data. The full table with all feature sets can be found in S1 Appendix (2 d., e., f.). Fig 3 presents the rank of the best models in specific embedding classes.

**Table 4:** Results for TwiBot-20 dataset—best 10 models. Full results are available in S1 Appendix as Table 2d.

rank	accuracy	F1	MCC	AUC	feature_set	type	compression_name	compression_dim
**1**	0.659	0.770	0.272	0.691	node_stats_nbrs	node_stats	no_compression	NA
**2**	0.661	0.768	0.273	0.690	node_stats_nbrs_emb	node_stats	no_compression	NA
**3**	0.661	0.768	0.273	0.690	node_stats_emb	node_stats	no_compression	NA
**4**	0.647	0.765	0.243	0.666	node_stats	node_stats	no_compression	NA
**5**	0.631	0.759	0.187	0.631	node_stats_nbrs_pca_16	node_stats	pca	NA
**6**	0.616	0.757	0.168	0.612	node2vec_dim_64	node2vec	no_compression	64
**7**	0.610	0.757	0.085	0.547	node2vec_dim_64_umap_16_seed_0	node2vec	umap	64
**8**	0.611	0.756	0.073	0.543	node2vec_dim_64_umap_16_seed_2	node2vec	umap	64
**9**	0.608	0.756	0.075	0.535	node2vec_dim_64_umap_16_seed_1	node2vec	umap	64
**10**	0.620	0.755	0.157	0.608	node_stats_nbrs_emb_umap_16_seed_0	node_stats	umap	0

Source: own calculations.

Given that the maximum F1 score achieved in the literature (see: Fig 1) amounts to 0.85 [54], the best result amongst models presented below does not seem impressive: 0.77. On the other hand, it is worth underlining that in our experiment profile features were not used, as there exists the suspicion that data were manually annotated to be bot or non-bot on the basis of exactly these characteristics [54]. Indeed, [58] shows that adding profile features to a similar set of embeddings we propose, boosts F1 metrics to 0.84, so almost equal to the maximum obtained in the literature. This indicates to model score 0.77 as being subject to overfitting problem.

It shows the imperfection of not only the obtained dataset, especially the manual annotation process, but generally a challenge posed by datasets for anomaly detection. This is due to the fact that undoubtedly determining anomalies is a difficult task due to onthonogical constraints. Regardless of that, to avoid bias, as well as for generalization purposes, profile data should not be taken into account. That holds especially given that they can be obtained only from specific networks, if at all. Another concern is posed by the protection of user privacy, which results in ethical, if not legal, discussion.

In Table 4 and Fig 3, the feature set name is constructed with a prefix of feature set name, which is an embedding name with the addition of dim and original dimension (e.g. node2vec_dim_64), node_stats in case of node statistics, node_stats_nbrs when node statistics are supplemented with the statistics of neighbors, and nlp for text data. Whenever compression is applied, the name is suffixed with the compression method type name (pca or umap) and the final dimension number after the compression, such as node2vec_dim_64_pca_16. In the case of UMAP, the seed number is appended (e.g. node2vec_dim_64_umap_16_seed_0).

Table 4 and Fig 3 also show the original and final compression dimensions in columns. The former is presented only in the case of embeddings; in other cases, it does not matter for the purpose of the analysis and is displayed as NA (not applicable). If the feature set name has the suffix emb, the best-performing embedding was added to node statistics.

After taking profile characteristics out of the picture, one can make another interesting observation: none of the ten best models contains NLP data. The extended table in the Technical annex shows that the NLP features-based model ranks as 42th. This confirms what was already described in the Introduction section. Text data not only is rarely available but also requires lots of manual overhead, sometimes not offering a sufficient gain. The hypothesis about better performance of graph-based methods holds even under the assumption that the structure of the whole graph is not preserved, i.e. the TwiBot-20 graph was sampled from the whole Twitter network. Relatively high predictive power of graph-derived data, even though the graph structure was sampled, is proof of the robustness of proposed approach.

Node statistics-based models (the algorithm proposed by the study) take all first five places. They are followed by a classical embedding—Node2vec. The latter may come as a surprise, especially given that classical embeddings did not perform well on other datasets. However, this is likely to be attributed to the process of the TwiBot-20 graph generation. As indicated, Twitter data was sampled by random choice of starting nodes and then followed by their relationships up to layer 4 (the detailed process was described in Materials and Methods). This way, only the nearest neighborhoods are preserved, being reflected by classical embedding. This is contrary to the overall node role in the whole network, embedded in turn into structural embeddings. That being said, Fig 3 shows that one cannot draw general conclusions about the superiority of classical embeddings even when the graph is sampled, as the second best-performing embedding with very similar F1 metrics is RolX (structural one). This makes the choice of an appropriate embedding even more complicated. Fig 3 also indicates that the F1 loss associated with a choice of worse embedding amounts only to 0.03 on the analyzed dataset (compared to over 0.1 in case of others). Again, this shows the robustness of graph-based methods in this case.

As many as 5 out of 10 best models were based on compressed features, both PCA and UMAP. Moreover, all of the featured best-performing embeddings had an original dimension of 64. Fig 3 contains average metrics for models grouped by dimension number and indeed this dimension is on average the best predictor. This indicates that there is no linear dependency between embedding dimension number and model performance: at first, the F1 score increases with the increase of dimension number, but later (in the case of 128) a slight deterioration is observed. Also, Table 4 shows that the form of this relationship form can vary between types of embeddings.

Plots depicting the detailed relationship between embedding dimensions and model performance can be found under position 1d of S1 Appendix.

**Fig 4:**
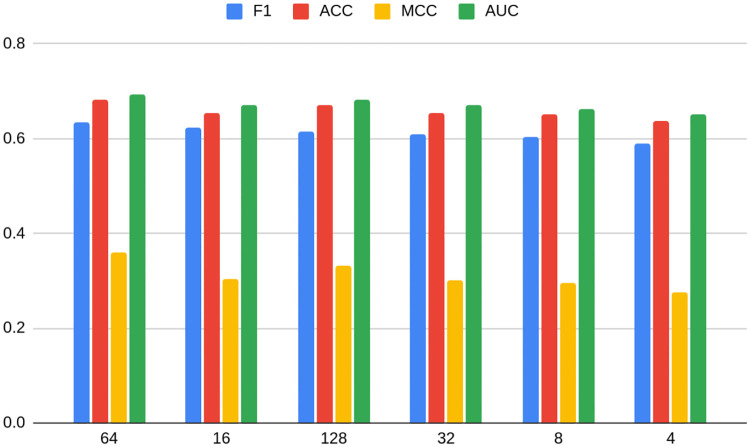
Average metrics for models grouped by dimension number for the TwiBot-20 dataset. Source: own calculations.

Also, embedding with the original dimension 64 performed a little bit better than its compressed version. It may mean that compression can help to extract information that is more meaningful for the given use case. Nevertheless, this conclusion does not hold with every embedding dimension and with every embedding type. Fig 4 shows the percentage of models where models based on the same dimension performed better in the compressed version. The main conclusion is that even though compression helped in the minority of cases, particularly it did not increase F1 metrics almost at all, there is still not enough evidence to determine whether there exists a general rule of thumb regarding compression usage. On the other hand, it seems to be embedding type and dimension number specific. Therefore, engineering effort related to the case by case choice of most suitable embedding is significant. An extended version of the table may be found under the position 2g of S1 Appendix.

**Fig 5:**
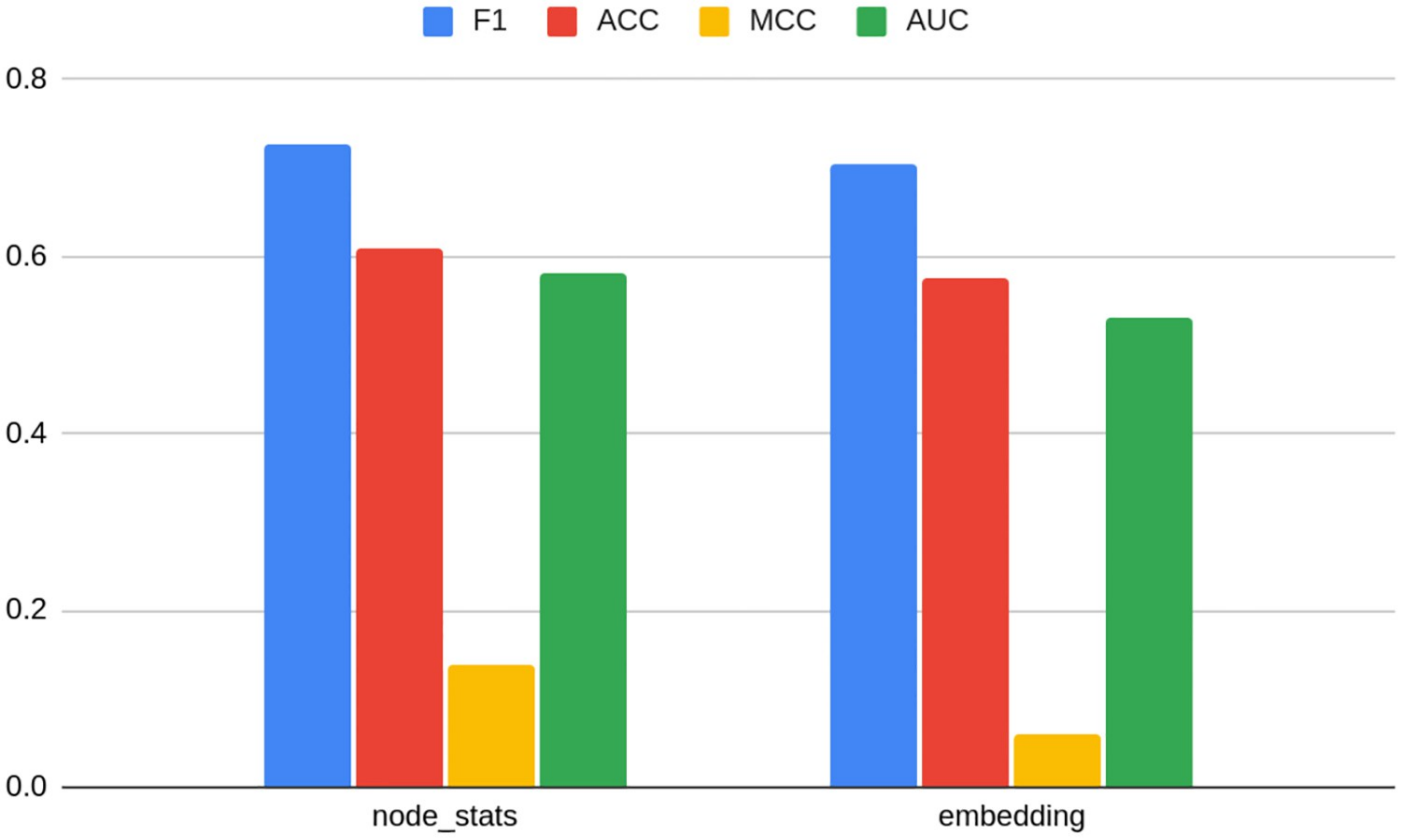
Average metrics for TwiBot-20 dataset models grouped by the fact of being based on embedding or on node statistics. Source: own calculations.

Since node statistics are performing consistently well, i.e. taking first five places, as well as saving computational time and effort regarding embedding and dimension choice, they seem to be an attractive predictor of anomalies. Fig 5 presents average metrics for models grouped by the fact of being based on embedding or on node statistics and it is clearly visible that the latter outperforms the former. Node statistics also have another major advantage: when GCN and Struc2vec embeddings could not be created for TwiBot-20 due to computational constraints (even on AWS compute and memory optimized virtual machine), and other types of embeddings took hours to generate, node based statistics were created within minutes. This only shows how powerful the proposed algorithm is. The trade off might be the amount of information node statistics store, so the study proposes enriching the feature set with the statistics (mean, max, min, standard deviation) of neighbors of different orders. Efficient implementation increases computational time from several to dozen minutes, which is still significantly more than hours, or infinity in the case of infeasible GCN and Struc2vec algorithms.

Given trends in the development of digital markets, graph sizes can only increase over time. Furthermore, graph structure may change quickly, especially in the case of computational propaganda methods (using troll farms), therefore there exists a need for a computationally efficient solution to quickly recalculate results. Node statistics seem to fulfill these requirements. Also, adding statistics of neighbors up to order 4 may seem to be worth an effort: it results in 0.01 F1 metrics gain, while not increasing computational time significantly.

Node statistics including neighbors up to fourth order, enriched with the best-performing embedding, yield a 0.768 F1 score. This results in second place just after node statistics including neighbors. Nevertheless, the model ranked second and had slightly better accuracy. The difference in metric values between those two is no bigger than 0.002 percentage points, which again proves the robustness of graph methods, especially taking node statistics into account. Combined with good performance regarding model metrics one can make a conclusion that enriching node statistics with information about neighbors of higher ranks materially increases the predictive power of the model (0.012 difference in F1 score).

On the other hand, extending the feature set with best-performing embedding did not seem to improve model results that much in the case of TwiBot-20. A slight deterioration of 0.002 difference in F1 score may be observed in the case of node statistics with neighbors. In the case of pure statistics without neighbors, adding an embedding to the feature set increased F1 by 0.003. This means that the best-performing embedding did not contain much more useful information than node statistics in the context of anomaly detection for this particular dataset.

The fact that ReFeX is not present in Table 4 is also worth underlining. It ranks 27th with a loss in the F1 metrics of 0.02 compared to the best-performing model. This may mean that even though it is an embedding constructed on the basis of node features, it holds different information than pure node statistics. In the case of this anomaly detection task, they turned out to be less useful, at least on the TwiBot-20 dataset. This is another argument in favor of using node statistics with its neighbors’ information for constructing a feature set for anomaly detection.

### Bitcoin OTC and Bitcoin Alpha

**Fig 6:**
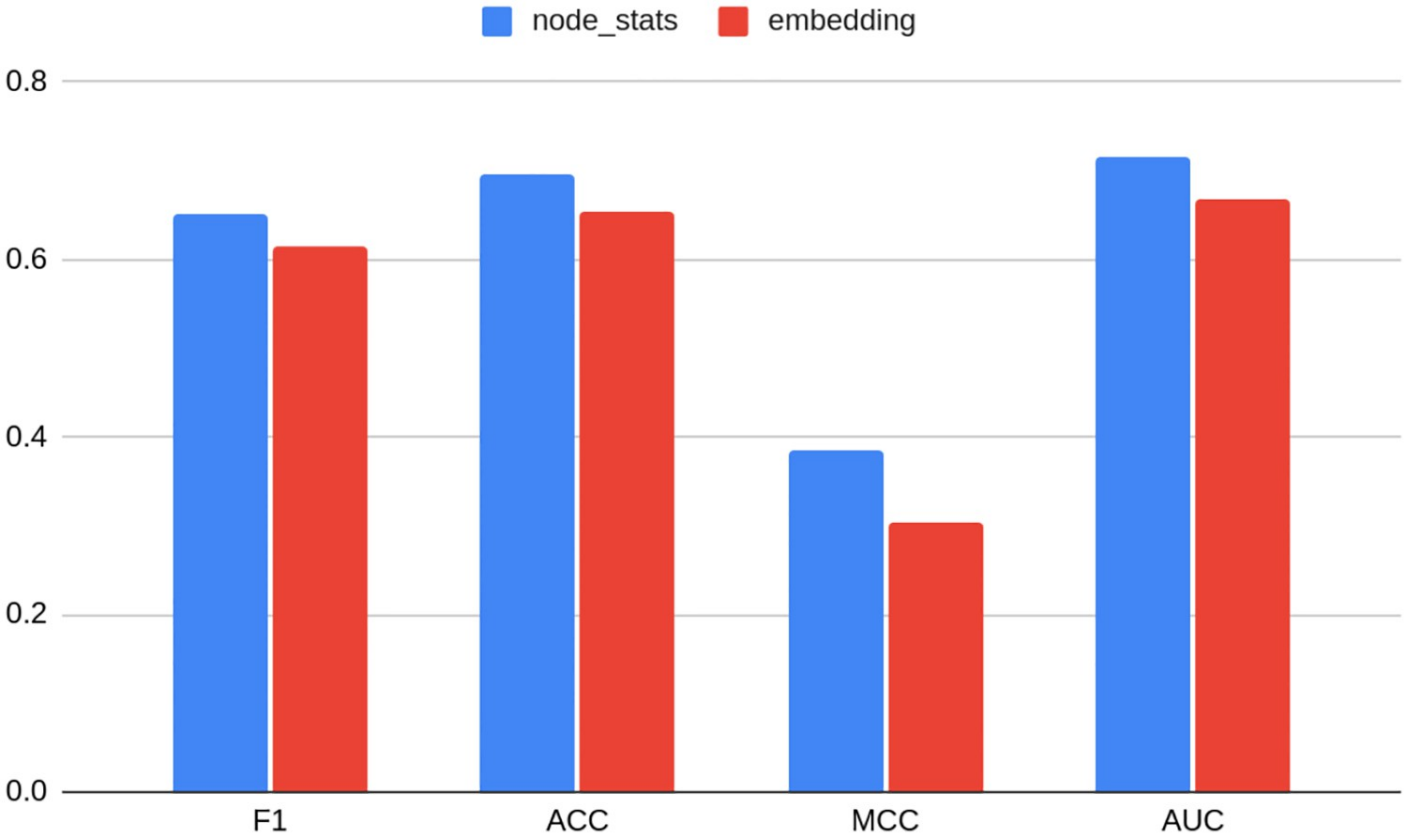
Average metrics for Bitcoin OTC dataset models grouped by the fact of being based on embedding or on node statistics. Source: own calculations.

**Fig 7:**
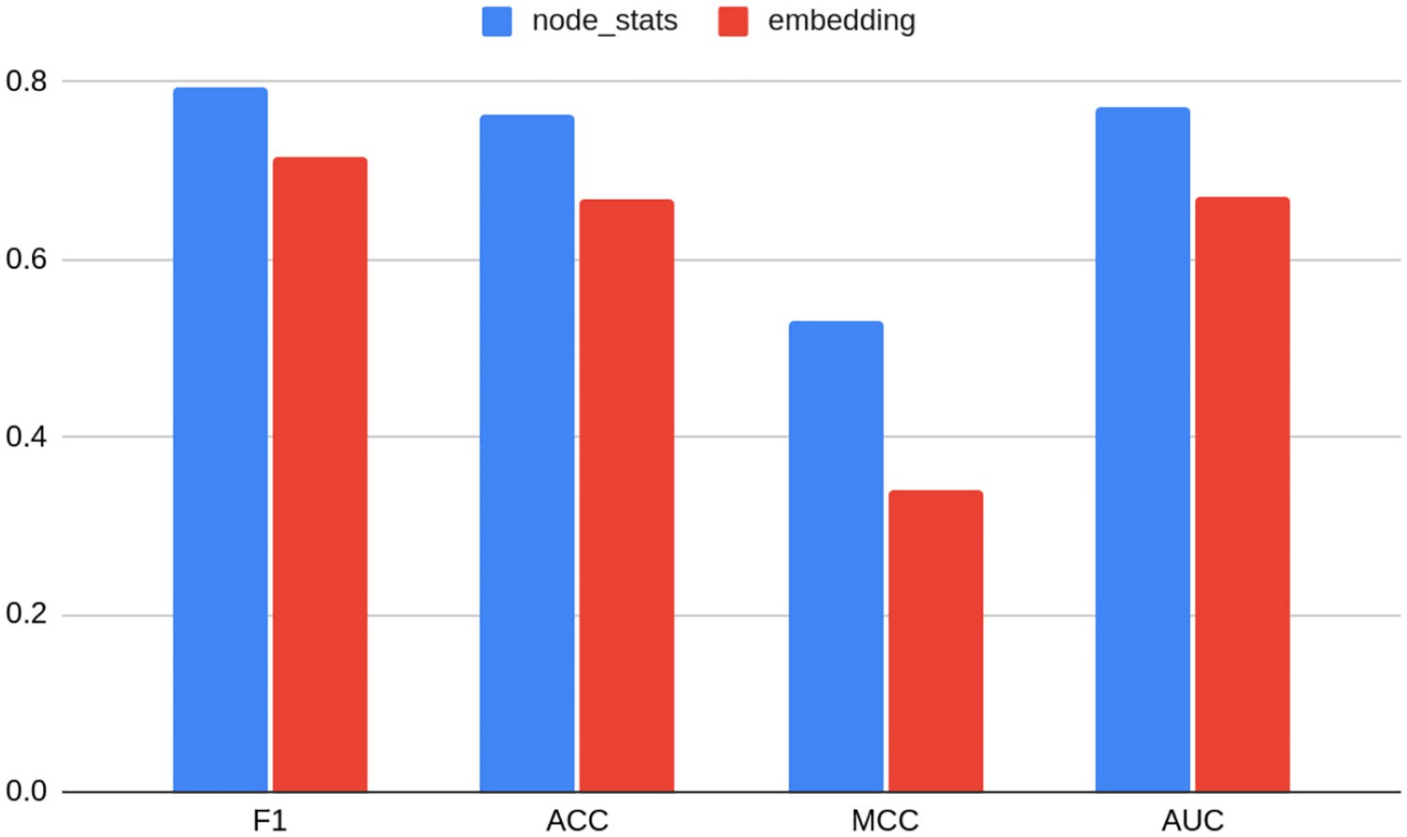
Average metrics for the Bitcoin Alpha dataset models grouped by the fact of being based on embedding or on node statistics. Source: own calculations.

Similarly as in the previous Subsection, Tables 5 and 6 and Figs 6 and 7 present results of anomaly detection models for Bitcoin OTC and Bitcoin Alpha datasets respectively. Extended versions of these may be found under positions 2e-2i of S1 Appendix. Table 5 and 6 show ten best models, whereas Figs 6 and 7 contain the best models in their own groups.

**Table 5:** Results for Bitcoin OTC dataset—best 10 models. Full results are available in the S1 Appendix as Table 2e.

rank	accuracy	F1	MCC	AUC	feature_set	type
**1**	0.895	0.891	0.790	0.928	refex	node_stats
**2**	0.895	0.886	0.789	0.950	rolx_dim_128_pca_16	rolx
**3**	0.884	0.884	0.773	0.916	emb_all_pca_16	all_embeddings
**4**	0.874	0.878	0.760	0.906	struc2vec_dim_128	struc2vec
**5**	0.874	0.878	0.760	0.906	node_stats_emb_pca	node_stats
**6**	0.874	0.878	0.760	0.918	node_stats_nbrs_emb_umap_16_seed_0	node_stats
**7**	0.874	0.872	0.750	0.918	node_stats_nbrs_emb_pca_16	node_stats
**8**	0.874	0.872	0.750	0.884	struc2vec_dim_32	struc2vec
**9**	0.874	0.872	0.750	0.893	struc2vec_dim_128_pca_16	struc2vec
**10**	0.874	0.872	0.750	0.893	gcn_degree_cols_dim_32_umap_16_seed_0	gcn_degree

Source: own calculations.

**Table 6:** Results for Bitcoin Alpha dataset—best 10 models. Full results are available in S1 Appendix as Table 2f.

rank	accuracy	F1	MCC	AUC	feature_set	type
**1**	0.847	0.864	0.701	0.896	node_stats_nbrs_emb_pca_16	node_stats
**2**	0.833	0.864	0.701	0.861	node_stats_emb_pca_16	node_stats
**3**	0.847	0.861	0.695	0.831	struc2vec_dim_64_umap_16_seed_1	struc2vec
**4**	0.847	0.861	0.695	0.870	struc2vec_dim_64_umap_16_seed_0	struc2vec
**5**	0.833	0.860	0.689	0.869	node_stats_pca_16	node_stats
**6**	0.833	0.857	0.679	0.854	bitcoin_alpha_gf	graph_features
**7**	0.833	0.857	0.679	0.868	struc2vec_dim_32	struc2vec
**8**	0.819	0.851	0.665	0.890	node_stats_emb	node_stats
**9**	0.833	0.850	0.668	0.824	node_stats_emb_umap_16_seed_1	node_stats
**10**	0.833	0.850	0.668	0.844	struc2vec_dim_128_umap_16_seed_1	struc2vec

Source: own calculations.

The first interesting observation on the Bitcoin datasets stemming from Tables 5 and 6 is that the best metrics determined in the literature after an overview of state-of-the-art algorithms, i.e. AUC 0.90 and 0.88 for Bitcoin OTC and Bitcoin Alpha respectively [16], were both overperformed by the following analysis. This speaks in favor of the efficiency of graph-based methods for the anomaly detection task.

The first seven models outperformed the best metrics from the literature for Bitcoin OTC. The best model based on ReFeX embedding amounted to approximately 0.93 AUC. For the Bitcoin Alpha dataset, only one model beats the best score from the literature, and it is based on node statistics, neighbors, best embedding, and PCA compression, which may speak in favor of the research hypothesis proposing a procedure for preparing a set of predictors in that way.

ReFeX is the only analyzed embedding based on node statistics, converting them to binary features using matrix factorization techniques, therefore was marked in the analysis as belonging to the “node statistics” group. The fact of being ranked in the Bitcoin OTC table is opposite to the case of TwiBot-20 and Bitcoin Alpha, where ReFeX does not take any place among the ten best models. This has two implications: first, that ReFeX may hold different information than node statistics indeed, but it is dataset specific whether they are more useful in case of anomaly detection. In particular, TwiBot-20 was a sampled dataset, which might have influenced the results. The second implication is that whereas the choice of embedding type might result in high variation of the results, node statistics are performing consistently well.

For Bitcoin OTC, other node statistic-based models ranked in 5th, 6th, and 7th position, which is a bit different result than in the case of TwiBot-20 and Bitcoin Alpha. On the contrary, for Bitcoin Alpha, as many as six models out of ten are based on node statistics, including the top two. Nevertheless, Figs 6 and 7 still show that models based on node statistics perform on average better compared to embeddings for both datasets.

Also, for Bitcoin OTC the F1 loss associated with node statistics-based models compared to the best model amounted only to 0.01, which proves robustness of its results. That being said, all of the abovementioned node feature-based models also included best-performing embedding, RolX of dimension 128 compressed to 16. This is an argument speaking in favor of the three-step procedure for constructing graph feature sets, including node statistics, neighbor statistics, and the best-performing embedding (can be also enriched with feature compression). This is both performance and computationally efficient, serving in practice as embedding—as it maps a graph into a vector space.

It may be worth mentioning that the pure node feature-based model for the Bitcoin OTC dataset (i.e. without embedding included in the feature set), displays F1 score of 0.86, and an efficiency gain of 0.02, which is still a good result. With AUC equal to 0.92 this model is still outperforming state-of-the-art algorithms from the extended literature review [16].

**Fig 8:**
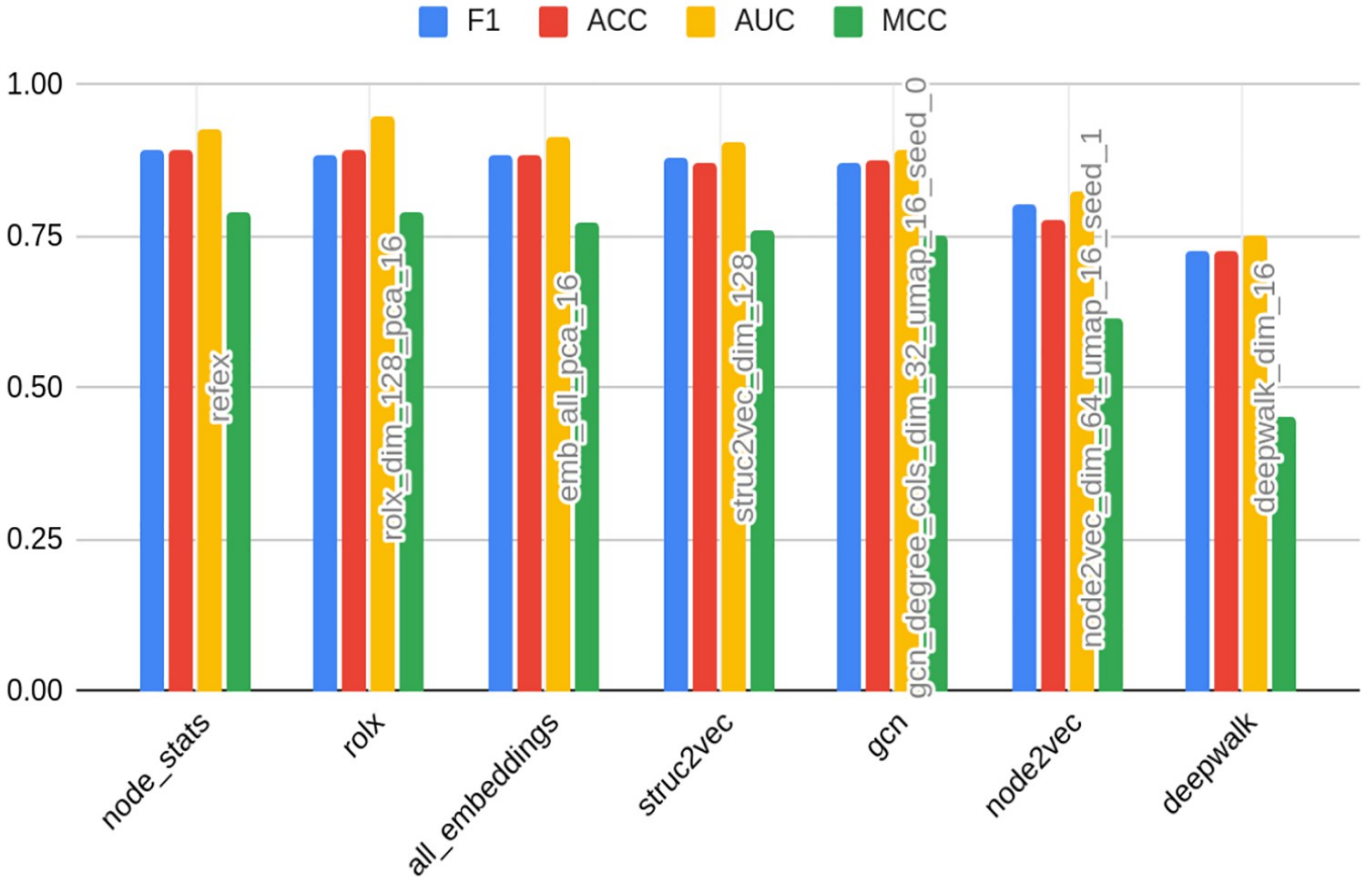
Best models in the class of embedding type for Bitcoin OTC. Source: own calculations.

**Fig 9:**
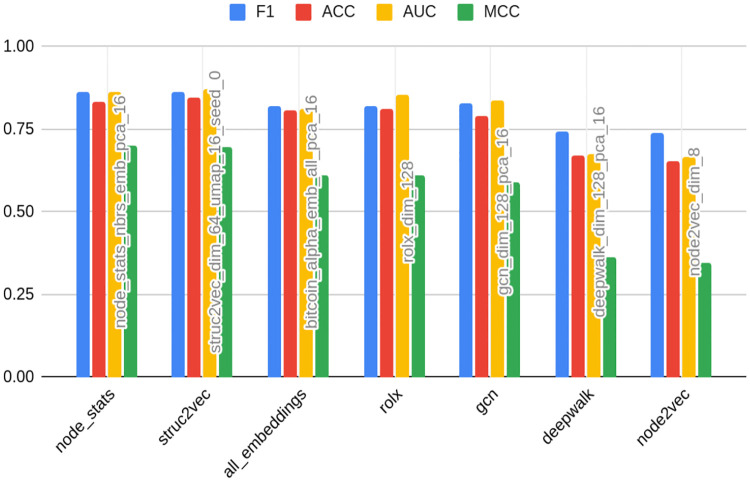
Best models in the class of embedding type for Bitcoin Alpha. Source: own calculations.

From the data contained in Figs 8 and 9, it seems that structural embeddings perform significantly better than classical ones. For Bitcoin OTC there is a little discrepancy between best and worst structural in terms of F1 metrics (0.89 vs 0.87). RolX does not result in almost any efficiency loss compared to the best model F1 score, 0.89. For Struc2vec this value accounts for 0.002. The best classical embedding in this rank is Node2vec and results in a loss of 0.09 percentage points, and the worst classical (DeepWalk) yields a respective loss of 0.16 when compared to the best model. As mentioned above, that shows that the choice of the embedding used in the analysis may result in a significant deterioration of model performance. Node statistics, on the contrary, perform consistently and models based on them are characterized by robust performance. The loss associated with them is relatively small, even if not ranking as the best on the leaderboard.

For Bitcoin Alpha, the best-performing embedding turned out to be Struc2vec, ranking in the 3rd, 4th, 6th, and 10th positions. Similarly to Bitcoin OTC dataset analysis, the performance of this structural embedding turns out to be stable, in the sense that it is ranking high in its different variants. Also analogously, the following conclusions hold: firstly, structural embeddings are performing better than classical. Secondly, there is a relatively low dispersion of model performance metrics among structural ones. The worst structural (GNN) results in 0.82 F1 metrics, compared to 0.86 best model F1 metrics, meaning 0.04 loss.

On the other hand, choosing a classical embedding for Bitcoin Alpha would yield a 0.12 loss in both DeepWalk, as well as Node2vec cases, which speaks in favor of using structural embeddings for the anomaly detection task. It is worth reminding that it was Node2vec that performed best among embeddings on the Twibot-20 dataset. This, in turn, may be attributed to the way the dataset was constructed, namely sampling up to layer 4, which naturally preserves local properties rather than structural, but may also show how much risk and uncertainty is attributed to the choice of the type and dimension of the embedding.

Knowing that choice of embedding type and dimension is a strongly dataset-specific problem, the only solution to determine the best one is to prepare all of them (on the grid of dimensions). Next step would be to compare metrics from models built on the top of each and every of them. That does not seem like a computationally feasible solution, taking into account that generating one embedding type in a given dimension for smaller graphs (Bitcoin OTC and Alpha) took couple of hours. Furthermore, graph structure changes over time, meaning that this task might be required to be conducted again in short time intervals. This might be ensured by using node statistics based feature sets (where generation time accounts for minutes, even after including neighor information).

**Fig 10:**
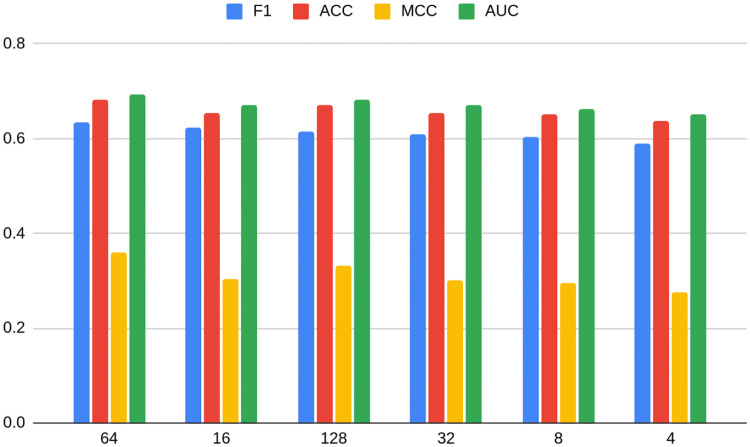
Models grouped by dimension number for Bitcoin OTC dataset, average metrics. Source: own calculations.

**Fig 11:**
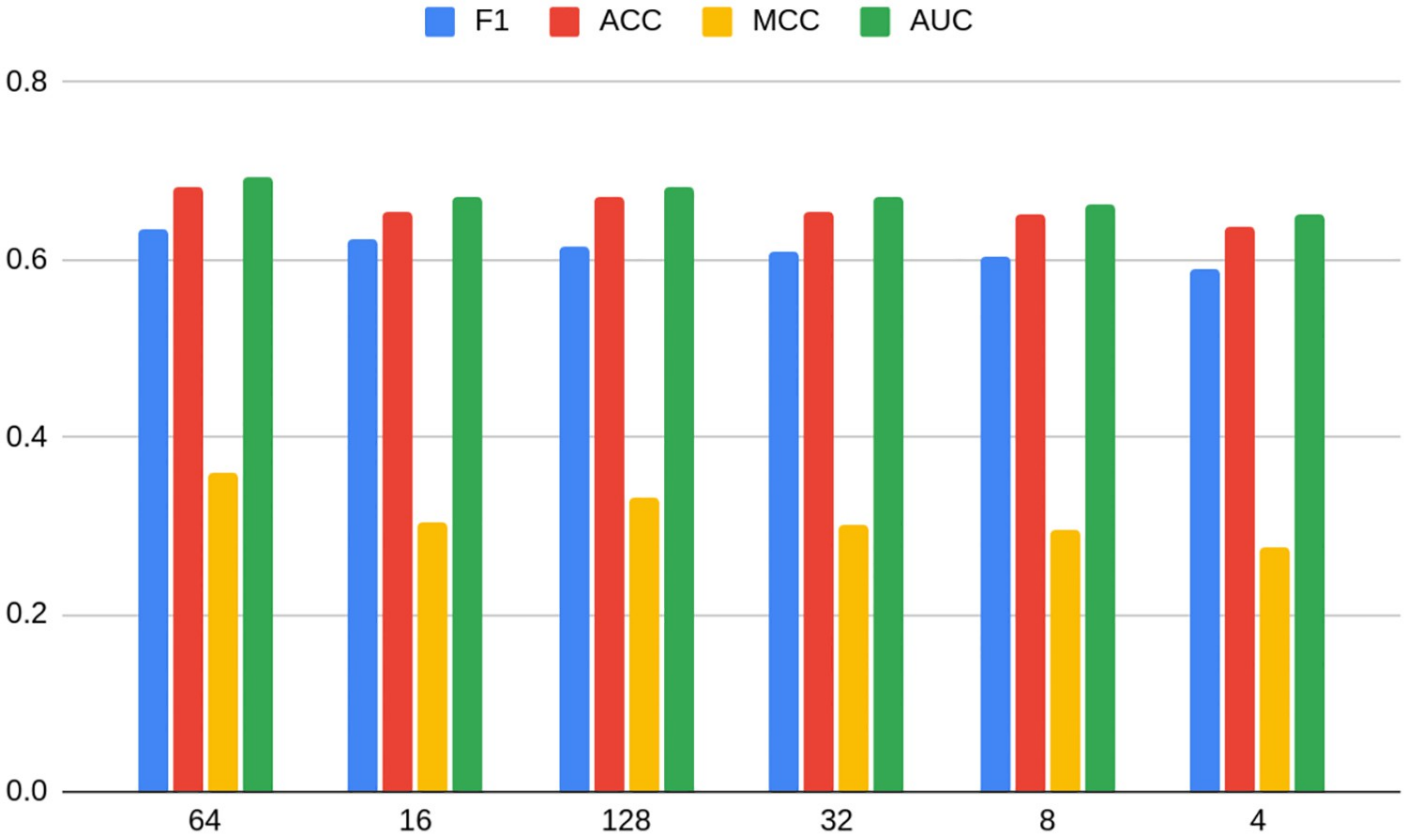
Models grouped by dimension number for Bitcoin Alpha dataset, average metrics. Source: own calculations.

Figs 10 and 11 show average metrics for models grouped by dimension number for Bitcoin OTC and Bitcoin Alpha datasets, respectively. In the case of the former, a non-linear relationship similar to TwiBot-20 is more or less preserved. It experiences a deterioration of performance when reaching dimension 32, but on the other hand arriving at the maximum with dimension 64 (just as for TwiBot-20), to fall down again later. On the other hand, the form of the relationship is more unclear in the case of Bitcoin Alpha. The maximum is reached earlier, namely with dimension 8, and differently as predecessors, the second best being 128. It is also worth noting that the minimum dimension (4) is not performing the worst. In the case of Bitcoin Alpha, the worst performer is dimension 16.

Again, the conclusion can be drawn that not only is the task of choosing the best embedding, as well as its dimension dataset-specific, but also it may differ between datasets of similar characteristics. Even though non-linear relationships seem to hold in most cases, the Bitcoin Alpha dataset shows that there is still not enough evidence in order to confidently use it as a rule of thumb in practice. As shown on Figs 5 and 6, the choice of non-optimal embedding may result in a relatively strong deterioration of the model performance, even as much as 12 percentage points.

**Fig 12:**
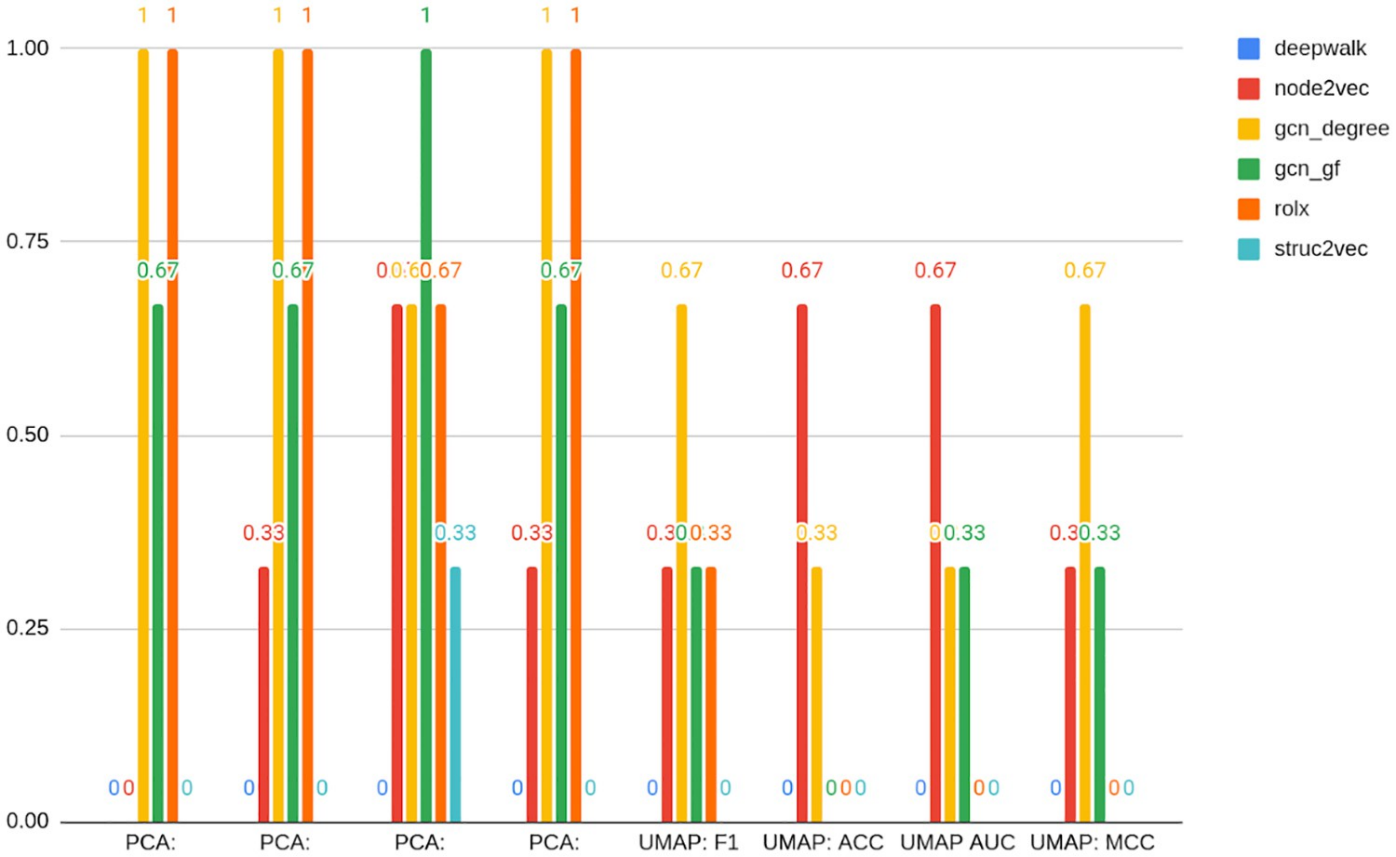
Models outperformed by the model with same feature set but compressed, Bitcoin OTC dataset, fraction. Source: own calculations.

**Fig 13:**
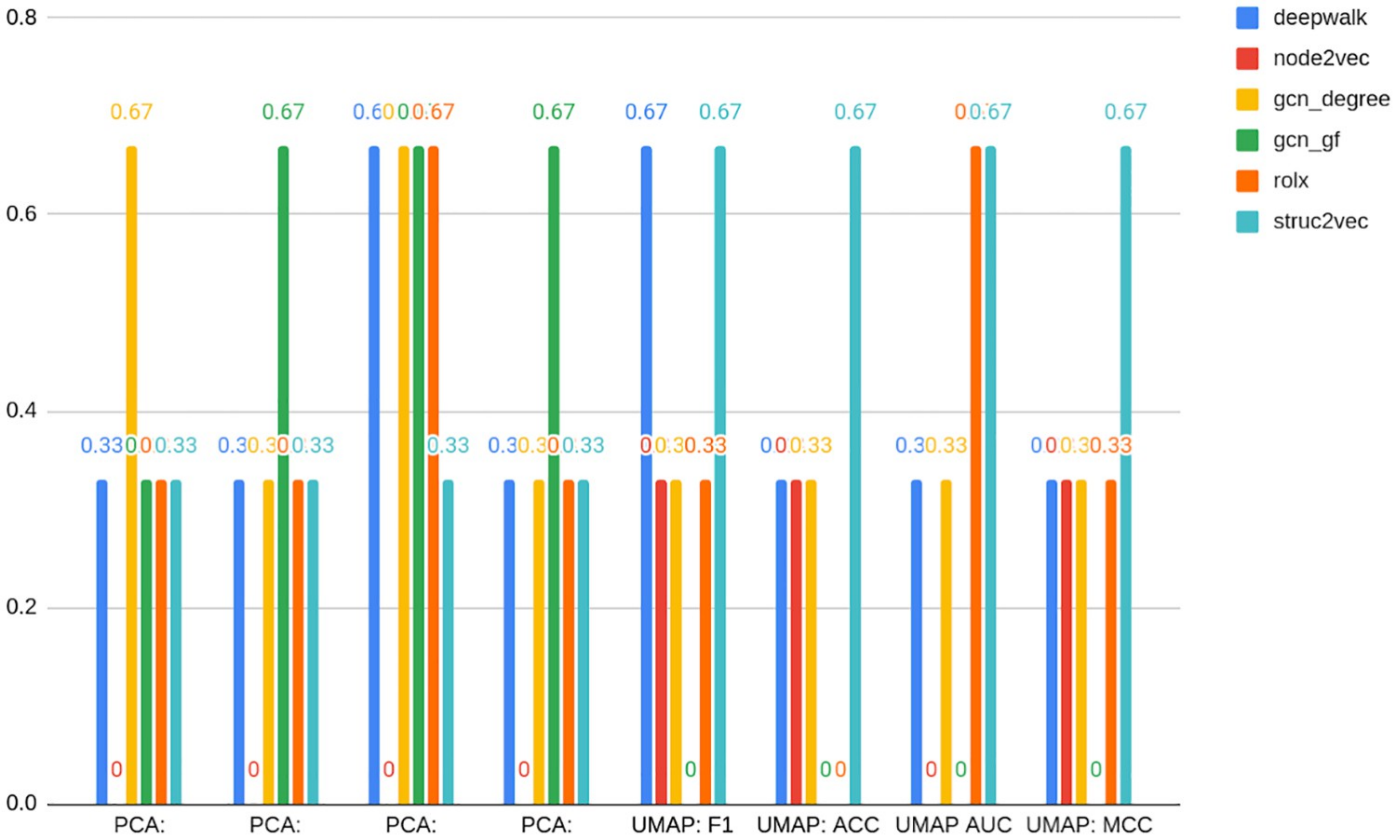
Models outperformed by the model with same feature set but compressed, Bitcoin Alpha dataset, fraction. Source: own calculations.

Figs 12 and 13 show the percentage of models in the given embedding class that perform better when compressed and support the evidence that compression helps in a subset of cases that are feature set, as well as dataset-specific. For example, for Bitcoin OTC, compression helped to achieve better F1 results in 45 percent of cases using PCA and 28 percent using UMAP, which accounts for 35 percent on average. For Bitcoin Alpha, these numbers amount to 33 percent (PCA), and 39 percent (UMAP), which yields 36 percent on average.

This is not to say that results speak in favor of not using compression, as there definitely exists a subset of cases, where it increases model performance. Thanks to the low computational cost associated with dimensionality reduction, it is useful to check it case by case. Extended versions of the table, as well as plots for detailed dimensionality analysis, may be found under positions 2h, 2i, and 1e, 1f respectively.

## Summary and conclusion

Traditional methods for anomaly detection involved models built on the basis of behavioral data, such as information about users and user-generated content (e.g., text). This poses many privacy and ethical concerns regarding data extraction and processing and becomes increasingly restricted. The advancements in generative AI methods have allowed for lowering transaction costs and barriers of entry to create artificial text content in an automated and massive way. It is challenging to distinguish artificial content from human-generated one, even for the human eye, not to mention machine learning models.

As shown in the previous subsections, both the computational effort and engineering overhead related to embedding are significant. The creation itself varies from a couple of hours (in the case of the graph with slightly over 3,000 nodes) to infinite, which means not being able to generate the embedding at all (graph with above 150,000 nodes). Then, the choice of the best embedding instance requires generating multiple types of those (based on different algorithm). To make an informed decision about its dimension, this generation should account for the grid of multiple dimensions, which results in a couple of hours times the number of embedding types (additionally multiplied by the number of dimensions).

Of course one can skip the leaderboard building and make a more or less informed guess regarding the embedding, choice of the suboptimal embedding might result in a relatively high performance loss. For Bitcoin OTC, choice of best model based on DeepWalk algorithm would yield F1 loss of as much as 0.164, whereas for Bitcoin Alpha this value accounted for 0.124. By using models based on node statistics these concerns do not apply anymore. One can compute them within minutes, even for a big graph (above 150,000 nodes). Moreover, these models are characterized by better explainability and interpretability, and might be quickly reproduced or recalculated.

During the study, it was shown that none of the ten best models for the TwiBot-20 dataset contains NLP data (Table 4). The extended table in the Technical annex presents that the NLP features-based model ranks 42nd out of 74. What is more, score dispersion among NLP models is bigger than for graph-based ones, which translates to lower stability of the results.

This confirms that text data not only requires lots of manual overhead and is easy to replicate by automated chatbots, but also is both less generalizable and robust than graph-derived data for the given task. It is worth bearing in mind that TwiBot-20 is a sampled dataset, so the whole graph is not preserved. This only speaks in favor of the robustness of graph-based data results, meaning they are performing significantly better even if not complete or accurate. Graph-based methods may become more prevalent as generative AI technologies allow for replicating human-like text in a high quality and low cost manner, quite opposite to replicating reliable graph structure.

The study proposes usage of computationally efficient graph statistics instead of graph embeddings, which, even though favored by the literature, are associated with high computational and engineering costs. Moreover, models built on the top of node statistics outperformed in average not only models built using graph embeddings for all three datasets Figs 5, 12 and 13, but also literature’s state-of-the-art models [16]. The leader models were characterized by AUC amounting to 0.93 (Fig 1) and 0.90 (Fig 2) versus 0.90 and 0.88 respectively, which is already a significant achievement.

Having said that, the study proposes its own algorithm aiming at the balance between computational efficiency and the amount of structural information contained in the data. This can be achieved by enriching node statistics with statistics of their neighbors up to level 4, such as minimums, maximums, mean, and standard deviations of selected node statistics. This results in 0.01 improvement in terms of F1 in the case of both TwiBot-20 and Bitcoin OTC dataset, compared to the model built on the top of simple node statistics. For Bitcoin Alpha the corresponding difference accounts for -0.025, meaning, on the opposite, efficiency loss. Even though it is not true that adding neighbor statistics always materially increases the predictive power of models, models based on them are consistently ranked high. This speaks in favor of solution stability. They were not only the best in the class of node statistics-based models but also overall in TwiBot-20 and Bitcoin Alpha datasets. Even though Bitcoin OTC dataset was an exception, the model of interest still ranked as 7th out of 151). These models took 6, 3, and 5 positions on the leaderboard out of 10 for the TwiBot-20, Bitcoin OTC, and Bitcoin Alpha respectively. Having said that, even though adding data about node statistics does not always result in a big efficiency gain, it will not materially deteriorate model performance while offering results’ robustness and computational efficiency.

**Fig 14:**
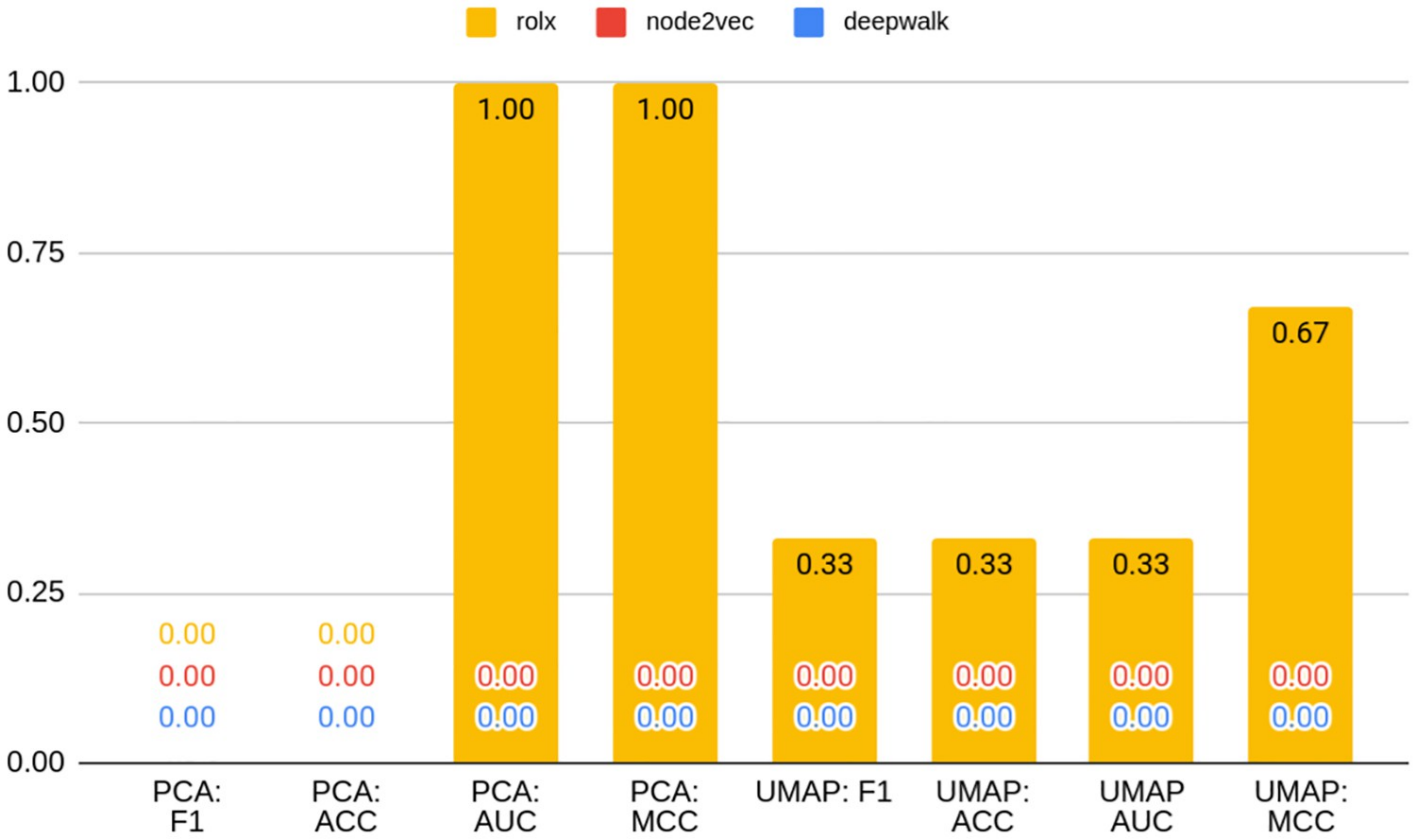
Percentage of models where a model built using a given embedding instance after compression outperformed the model without compression for the TwiBot-20 dataset. Source: own calculations.

Figs 12–14 shows that selection of best performing embedding type as well as their dimension is dataset specific and it is not possible to construct any heuristics for this problem. The same goes for compression: models based on the compression performed better than the original ones in, on average, 0.05 percent for TwiBot-20, 35 percent for Bitcoin OTC, and 36 percent for Bitcoin Alpha Figs 12–14. This adds engineering overhead to methods based on traditional embeddings, as optimal feature set selection may be done only by preparing a grid of feature sets and iterating through them to find the best performing one. This is necessary because efficiency losses can be significant when using suboptimal embedding Figs 4, 11 and 12. Given computational inefficiency of traditional embeddings this is a suboptimal solution, quite opposite to the proposed procedure based on enriching node statistics with neighbor information. Not only can they be engineered within minutes, but also do not require specific comparisons or selections, still producing stable and consistently high metrics results.

All above conclusions show the stability and efficiency, both performance, as well as computational, of anomaly detection methods based on node statistics and their neighbors’ data. Its time of execution takes only several minutes in the case of a 156k nodes graph compared to several hours (or infeasibility) regarding graph embeddings.

Even though it can’t be conclusive whether adding neighbor node statistics up to level 4 as well as compression always contributes to efficiency gain, they never contribute to significant performance loss. Their results have proven to be robust and stable among 151 models per dataset. The proposed method is not associated with high computational cost, quite opposite to the construction of embeddings. It also takes off the burden of preparing multiple feature sets and comparing their performance to choose the best one. Therefore, it is useful to define the following procedure for building a feature set for the task of anomaly detection in the graph networks:

- extracting vertices statistics from a given graph;
- computing statistics (mean, maximum, minimum and standard deviation) of neighbors of vertices up to a given level (we propose 4);
- performing dimensionality reduction techniques using PCA or UMAP.

The last step may be optional depending on how well the feature sets with and without the compression perform in the given use case. Obviously, other available data about the network may be added to this feature set—before or after the compression.

### Concluding remarks

This study contributes to the existing research in several dimensions. First, it proposes to apply anomaly detection methods to the digital markets considered jointly. Secondly, it focuses on network structure instead of behavioral data [25, 28, 54]. As the former comes from the graph structure of the whole network, as opposed to the content generated by the user, it is far more difficult to tamper with. This cannot be said about automatic content generation: due to the newest technologies available, even the human eye cannot recognize whether the content was human-generated or not. This means that traditional approaches to anomaly detection will quickly become obsolete.

Furthermore, the research comprehensively compares different methods of graph data extraction and finds out that state-of-the-art approaches, such as graph embeddings [20], quickly become computationally inefficient or even infeasible, especially in the case of large graphs. What is more, they are associated with high engineering overhead. The study suggests the usage of graph neighbor-based statistics instead, to obtain efficiency both in terms of model results, as well as computations. It proposes its own algorithm on the top of them. The embedding advocated by the study can result in solving problems infeasible when using traditional graph embeddings [20], saving hours of computing time and minimizing engineering overhead associated with embedding choice. On top of that, the proposed approach turned out to outperform state-of-the-art methods compared in the literature up to this point [54, 59].

Further research in that area could benefit from robustness analysis, using other datasets or graphs simulated with the help of graph generation software. In particular, it would be interesting to see how research findings generalize to other markets or areas, where anomalous activities are present. Also, an interesting direction will be to continue analyzing the importance of particular node statistics and embedding dimensions in terms of model explainability [20, 21].

Studying larger graphs would open the door for more extensive optimization of computational efficiency. For example, one may achieve it by building an embedding representing node statistics in a more exhaustive way, but at the same time more complex than simple statistics calculation. Furthermore, it may be useful to continue research on developing measures for embedding quality for a given dataset, to avoid extensive work in order to determine which embedding is best for a given task [59].

Last, but not least, a promising direction of further research might be the extension of the presented methodology to unsupervised tasks, so datasets where labels do not exist and there is no a priori knowledge which users are anomalous. This may be particularly useful as most of datasets where suspicion of anomalous activity exist do not have ground truth unambiguously determined, or not determined at all.

## Supporting information

S1 Appendix Code might be found under: https://kaggle.com/code/agatasko/anomalies-graph-networks. The Technical appendix can be found under: https://www.kaggle.com/datasets/agatasko/tech-appendix.
List of supplements:
plots:a. 01_TwiBot_20_histograms.htmlb. 02_Bitcoin_OTC_histograms.htmlc. 03_Bitcoin_Alpha_histograms.htmld. 04_TwiBot_20_dimensionality.htmle. 05_Bitcoin_OTC_dimensionality.htmlf. 06_Bitcoin_Alpha_dimensionality.htmltables:a. 01_TwiBot_20_statistics.csvb. 02_Bitcoin_OTC_statistics.csvc. 03_Bitcoin_Alpha_statistics.csvd. 04_TwiBot_20_results.csve. 05_Bitcoin_OTC_results.csvf. 06_Bitcoin_Alpha_results.csvg. 07_TwiBot_20_compression_results.csvh. 08_Bitcoin_OTC_compression_results.csvi. 09_Bitcoin_Alpha_compression_results.csv
(ZIP)
