# Supplementary material for: Detecting anomalies in graph networks on digital markets
Source: PLoS One. 2024 Dec 23;19(12):e0315849. doi: 10.1371/journal.pone.0315849 (PMC11666048; doi:10.1371/journal.pone.0315849)
Supplement: S1 Appendix — The Technical appendix can be found under: https://www.kaggle.com/datasets/agatasko/tech-appendix. List of supplements: plots:a. 01_TwiBot_20_histograms.htmlb. 02_Bitcoin_OTC_histograms.htmlc. 03_Bitcoin_Alpha_histograms.htmld. 04_TwiBot_20_dimensionality.htmle. 05_Bitcoin_OTC_dimensionality.htmlf. 06_Bitcoin_Alpha_dimensionality.htmltables:a. 01_TwiBot_20_statistics.csvb. 02_Bitcoin_OTC_statistics.csvc. 03_Bitcoin_Alpha_statistics.csvd. 04_TwiBot_20_results.csve. 05_Bitcoin_OTC_results.csvf. 06_Bitcoin_Alpha_results.csvg. 07_TwiBot_20_compression_results.csvh. 08_Bitcoin_OTC_compression_results.csvi. 09_Bitcoin_Alpha_compression_results.csv (ZIP) [file pone.0315849.s001.zip › tech_appendix/plots/html/01_TwiBot_20_histograms.html]

01\_TwiBot\_20\_histograms


In [1]:

```
import numpy as np
import pandas as pd
import matplotlib.pyplot as plt
```

## TwiBot-20¶

### NLP data¶

In [2]:

```
nlp = pd.read_csv("twibot_20_nlp.csv")
for col in nlp.columns[1:-1]:
    x = nlp.loc[nlp["label"] == 1, col]
    y = nlp.loc[nlp["label"] == 0, col]
    
    bins = np.linspace(min(nlp[col]), max(nlp[col]), 50)

    plt.hist(round(x, 5), bins, alpha=0.5, label='bots', weights=np.ones(len(x))/len(x))
    plt.hist(y, bins, alpha=0.5, label='non-bots', weights=np.ones(len(y))/len(y))
    plt.legend(loc='upper right')
    plt.title(col)
    #plt.savefig(f'./charts/{col}.pdf') 
    plt.show()
```

### node statistics¶

In [3]:

```
twibot_gf = pd.read_csv("twibot_20_gf.csv")
```

In [4]:

```
twibot_gf = pd.read_csv("twibot_20_gf.csv")
for col in twibot_gf.columns[1:-1]:
    x = twibot_gf.loc[twibot_gf["label"] == 1, col]
    y = twibot_gf.loc[twibot_gf["label"] == 0, col]
    
    bins = np.linspace(min(twibot_gf[col]), max(twibot_gf[col]), 50)

    plt.hist(round(x, 5), bins, alpha=0.5, label='bots', weights=np.ones(len(x))/len(x))
    plt.hist(y, bins, alpha=0.5, label='non-bots', weights=np.ones(len(y))/len(y))
    plt.legend(loc='upper right')
    plt.title(col)
    #plt.savefig(f'./charts/{col}.pdf') 
    plt.show()
```
