# Supplementary material for: Detecting anomalies in graph networks on digital markets
Source: PLoS One. 2024 Dec 23;19(12):e0315849. doi: 10.1371/journal.pone.0315849 (PMC11666048; doi:10.1371/journal.pone.0315849)
Supplement: S1 Appendix — The Technical appendix can be found under: https://www.kaggle.com/datasets/agatasko/tech-appendix. List of supplements: plots:a. 01_TwiBot_20_histograms.htmlb. 02_Bitcoin_OTC_histograms.htmlc. 03_Bitcoin_Alpha_histograms.htmld. 04_TwiBot_20_dimensionality.htmle. 05_Bitcoin_OTC_dimensionality.htmlf. 06_Bitcoin_Alpha_dimensionality.htmltables:a. 01_TwiBot_20_statistics.csvb. 02_Bitcoin_OTC_statistics.csvc. 03_Bitcoin_Alpha_statistics.csvd. 04_TwiBot_20_results.csve. 05_Bitcoin_OTC_results.csvf. 06_Bitcoin_Alpha_results.csvg. 07_TwiBot_20_compression_results.csvh. 08_Bitcoin_OTC_compression_results.csvi. 09_Bitcoin_Alpha_compression_results.csv (ZIP) [file pone.0315849.s001.zip › tech_appendix/plots/html/04_TwiBot_20_dimensionality.html]

01\_TwiBot\_20\_dimensionality


In [1]:

```
import pandas as pd
import matplotlib.pyplot as plt
import itertools
```

# Dimension and compression vs model performance¶

## TwiBot-20¶

In [2]:

```
dataset_nam = "twibot"

y_test = pd.read_excel(f"./dimensionality_input/{dataset_nam}/results/ytest.xlsx"
                      ).drop(columns=["Unnamed: 0", "index"], errors="ignore")

results = pd.read_csv(f"./dimensionality_input/{dataset_nam}/results/results_aggregated.csv", 
                          index_col = 0)
```

In [3]:

```
# UMAP: only seed 0 for readability

a = results["embedding_type"].unique().tolist()
a.remove("graph_features")
a.remove("all_embeddings")
a

for model, metric, embedding_type, compression_name in list(
    itertools.product(results["model"].unique(),
                      #["acc", "f1", "mcc", "auc"],
                      ["f1"],
                      a,
                      results["compression_name"].unique())):
    
    if compression_name == "no_compression":
        pass
    else:
        print(f"------------- \n {model}, {metric}, {embedding_type}, {compression_name}")

        temp = results[results["compression_name"].isin(["no_compression", compression_name])]
        temp = temp[(temp["embedding_type"] == embedding_type) & (temp["model"] == model)]
        if compression_name == "umap":
            temp = temp[(temp["seed"] == 0) | (temp["seed"].isna())]

        temp2 = temp.sort_values(by="original_dim_number", ascending=True)

        fig, ax = plt.subplots(figsize=(12,8))
        plt.scatter(temp2["original_dim_number"], temp2[metric], label = temp2["embedding_name"])
        plt.title(f"Metric: {metric} vs embedding dimension ({embedding_type}, {compression_name}, {model} model)")
        plt.xlabel("original dim number")
        plt.ylabel(metric)

        for index in range(len(temp2["original_dim_number"])):
            ax.text(temp2["original_dim_number"].iloc[index],
                    temp2[metric].iloc[index],
                    temp2["embedding_name"].iloc[index], size=10)
        plt.show()
```

```
------------- 
 h2o, f1, node2vec, pca
```

```
------------- 
 h2o, f1, node2vec, umap
```

```
------------- 
 h2o, f1, rolx, pca
```

```
------------- 
 h2o, f1, rolx, umap
```

```
------------- 
 h2o, f1, text, pca
```

```
------------- 
 h2o, f1, text, umap
```

```
------------- 
 h2o, f1, deepwalk, pca
```

```
------------- 
 h2o, f1, deepwalk, umap
```

```
------------- 
 xgboost, f1, node2vec, pca
```

```
------------- 
 xgboost, f1, node2vec, umap
```

```
------------- 
 xgboost, f1, rolx, pca
```

```
------------- 
 xgboost, f1, rolx, umap
```

```
------------- 
 xgboost, f1, text, pca
```

```
------------- 
 xgboost, f1, text, umap
```

```
------------- 
 xgboost, f1, deepwalk, pca
```

```
------------- 
 xgboost, f1, deepwalk, umap
```
