# Supplementary material for: Detecting anomalies in graph networks on digital markets
Source: PLoS One. 2024 Dec 23;19(12):e0315849. doi: 10.1371/journal.pone.0315849 (PMC11666048; doi:10.1371/journal.pone.0315849)
Supplement: S1 Appendix — The Technical appendix can be found under: https://www.kaggle.com/datasets/agatasko/tech-appendix. List of supplements: plots:a. 01_TwiBot_20_histograms.htmlb. 02_Bitcoin_OTC_histograms.htmlc. 03_Bitcoin_Alpha_histograms.htmld. 04_TwiBot_20_dimensionality.htmle. 05_Bitcoin_OTC_dimensionality.htmlf. 06_Bitcoin_Alpha_dimensionality.htmltables:a. 01_TwiBot_20_statistics.csvb. 02_Bitcoin_OTC_statistics.csvc. 03_Bitcoin_Alpha_statistics.csvd. 04_TwiBot_20_results.csve. 05_Bitcoin_OTC_results.csvf. 06_Bitcoin_Alpha_results.csvg. 07_TwiBot_20_compression_results.csvh. 08_Bitcoin_OTC_compression_results.csvi. 09_Bitcoin_Alpha_compression_results.csv (ZIP) [file pone.0315849.s001.zip › tech_appendix/plots/pdf/01_TwiBot_20_histograms.pdf]

01_TwiBot_20_histograms

August 19, 2023

[1]:
```
import numpy as np
import pandas as pd
import matplotlib.pyplot as plt
```

0.1 TwiBot-20

0.1.1 NLP data

[2]:
```
nlp = pd.read_csv("twibot_20_nlp.csv")
for col in nlp.columns[1:-1]:
    x = nlp.loc[nlp["label"] == 1, col]
    y = nlp.loc[nlp["label"] == 0, col]

    bins = np.linspace(min(nlp[col]), max(nlp[col]), 50)

    plt.hist(round(x, 5), bins, alpha=0.5, label='bots', weights=np.
 ↪ones(len(x))/len(x))
    plt.hist(y, bins, alpha=0.5, label='non-bots', weights=np.ones(len(y))/
 ↪len(y))
    plt.legend(loc='upper right')
    plt.title(col)
    #plt.savefig(f'./charts/{col}.pdf')
    plt.show()
```

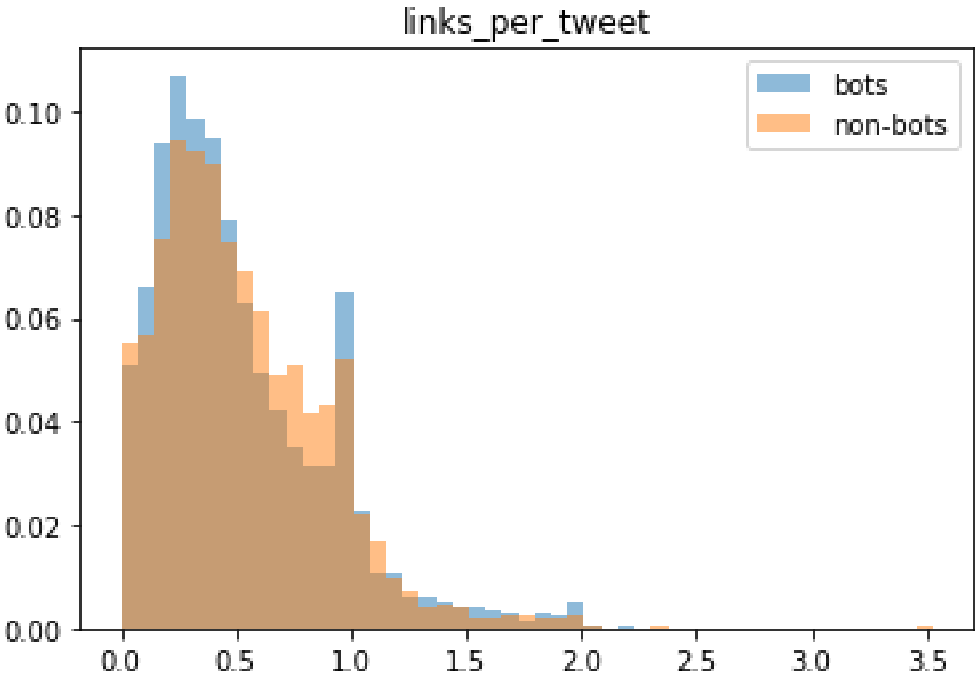
links_per_tweet
bots
non-bots
0.10
0.08
0.06
0.04
0.02
0.00
0.0
0.5
1.0
1.5
2.0
2.5
3.0
3.5

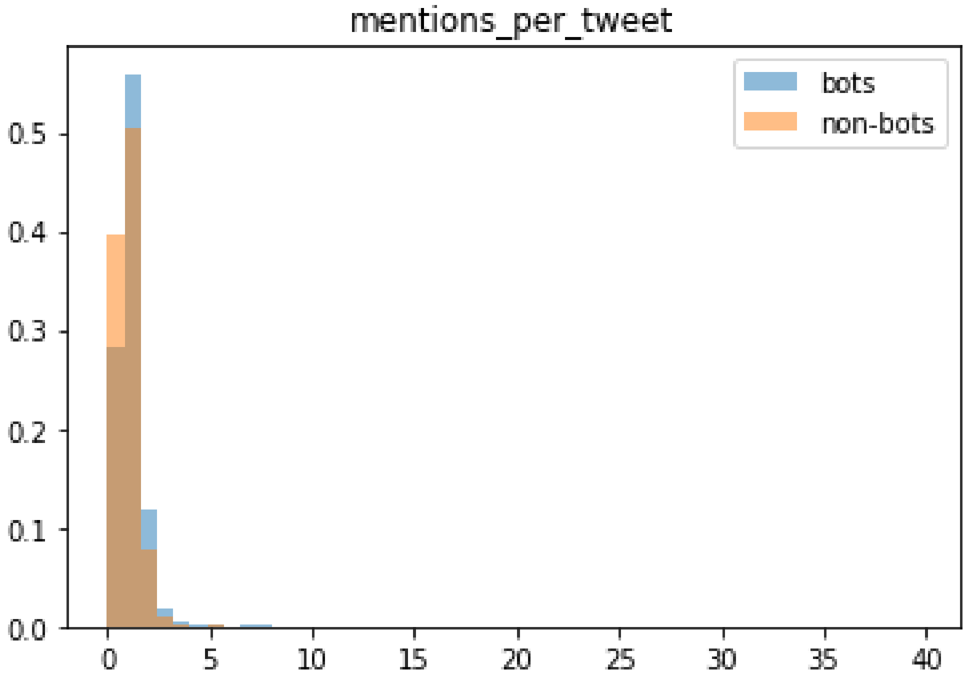
mentions_per_tweet
bots
non-bots
0.5
0.4
0.3
0.2
0.1
0.0
0
5
10
15
20
25
30
35
40

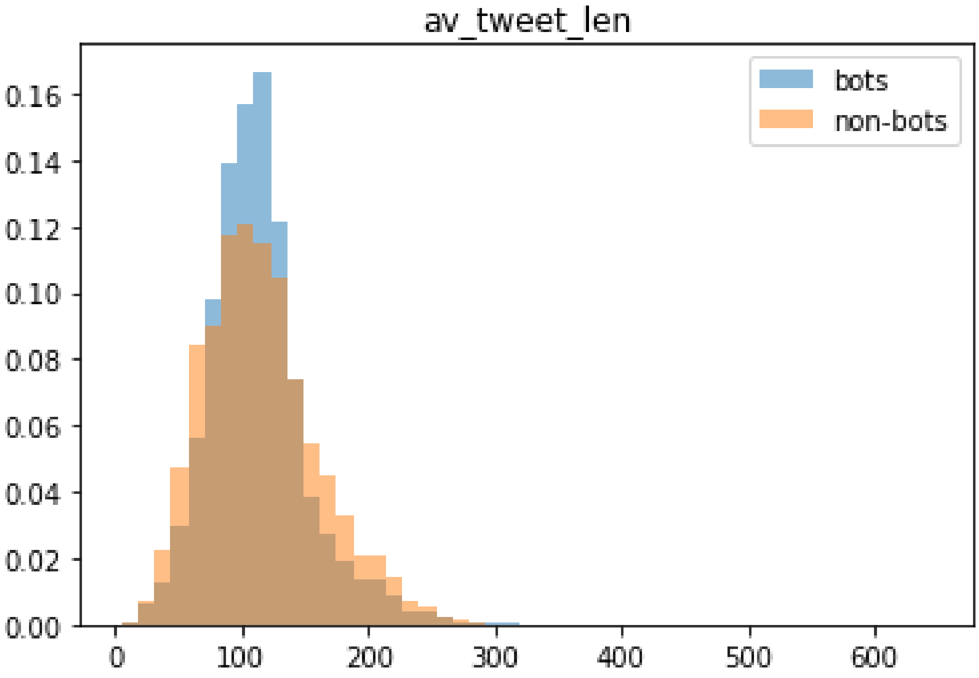
av_tweet_len
bots
non-bots
0.16
0.14
0.12
0.10
0.08
0.06
0.04
0.02
0.00
0
100
200
300
400
500
600

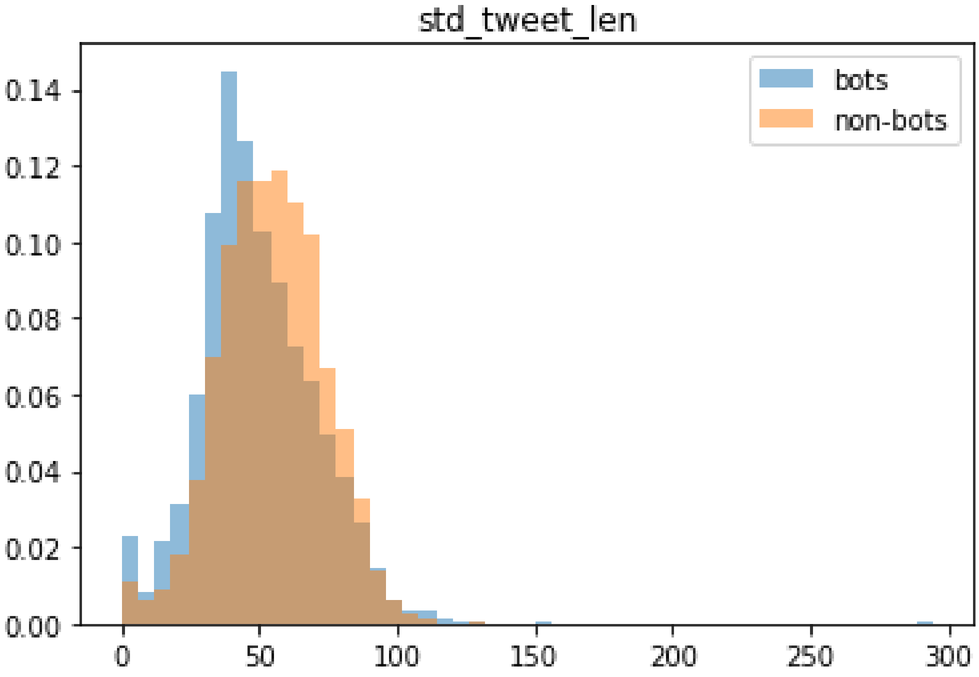
std_tweet_len
bots
non-bots
0.14
0.12
0.10
0.08
0.06
0.04
0.02
0.00
0
50
100
150
200
250
300

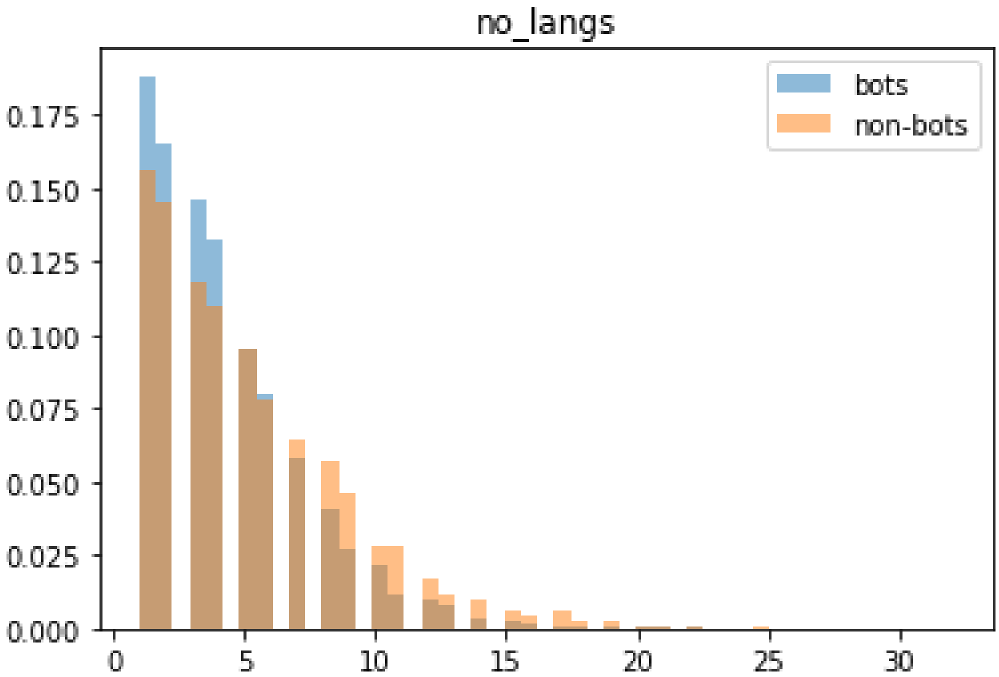
no_langs
bots
non-bots
0.175
0.150
0.125
0.100
0.075
0.050
0.025
0.000
0
5
10
15
20
25
30

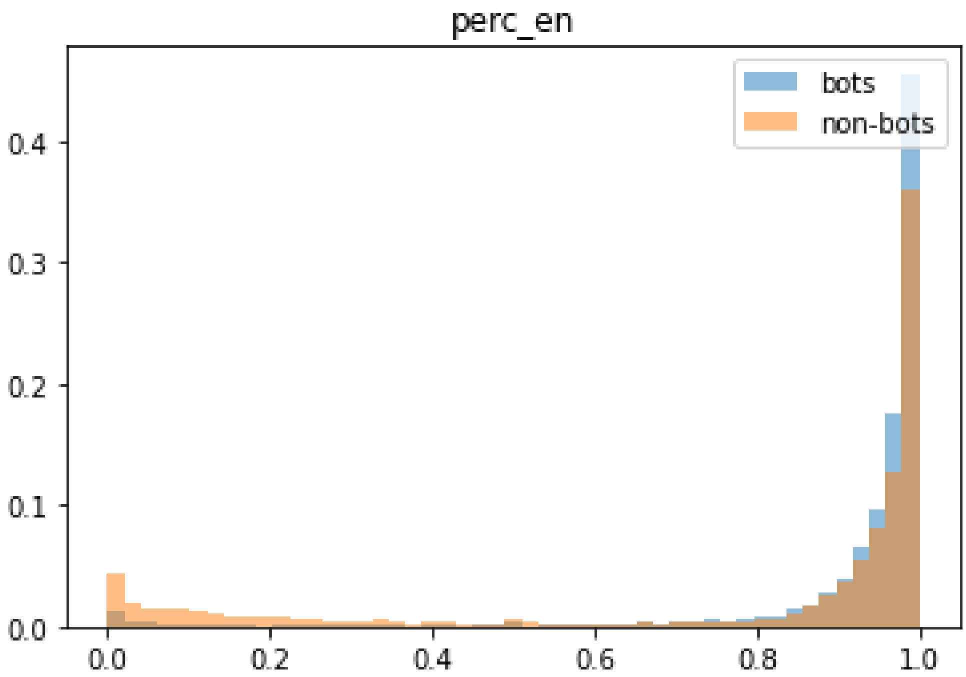
perc_en
bots
non-bots
0.4
0.3
0.2
0.1
0.0
0.0
0.2
0.4
0.6
0.8
1.0

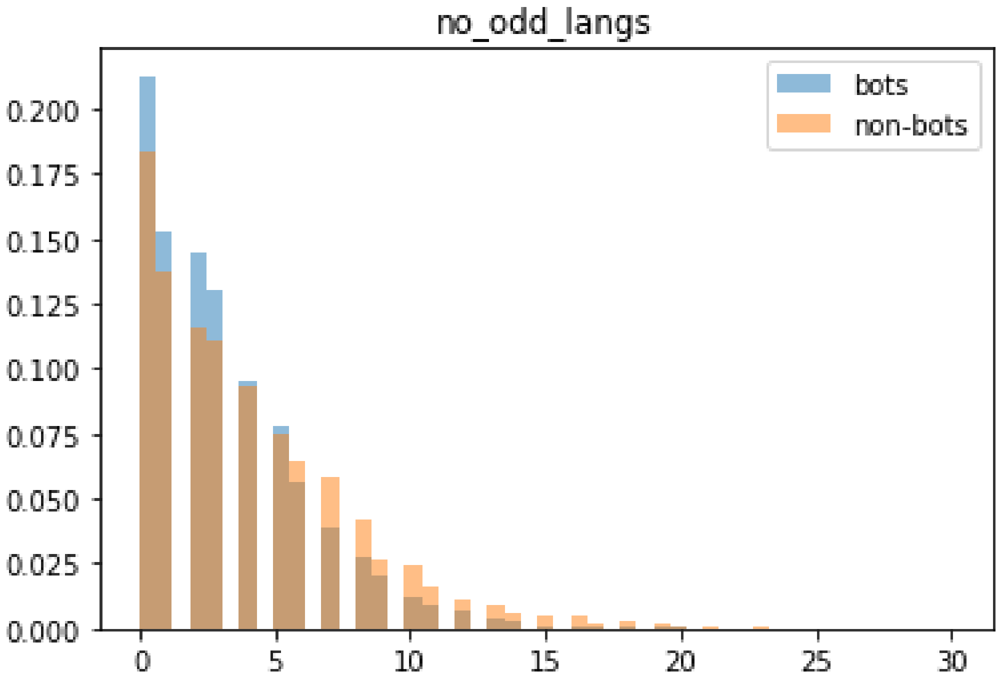
no_odd_langs
bots
non-bots
0.200
0.175
0.150
0.125
0.100
0.075
0.050
0.025
0.000
0
5
10
15
20
25
30

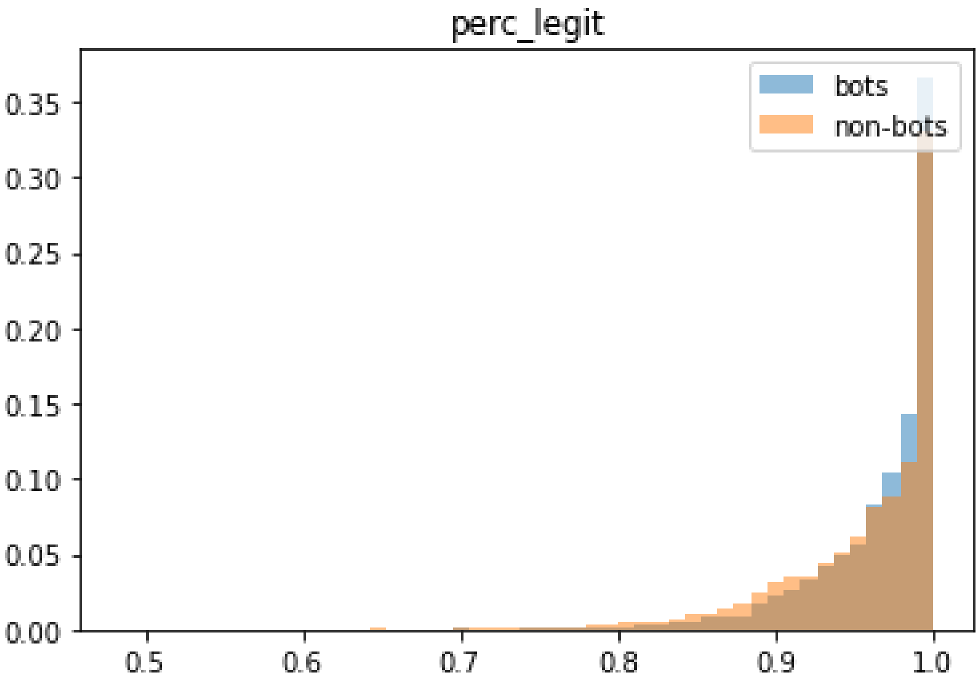
perc_legit
bots
non-bots
0.35
0.30
0.25
0.20
0.15
0.10
0.05
0.00
0.5
0.6
0.7
0.8
0.9
1.0

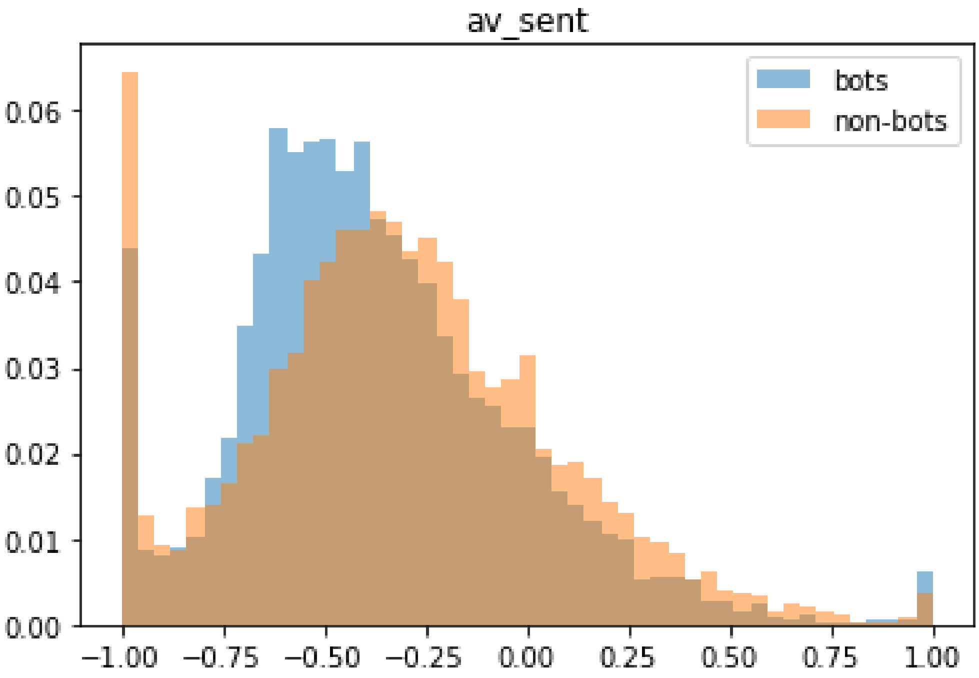
av_sent
bots
non-bots
0.06
0.05
0.04
0.03
0.02
0.01
0.00
−1.00
−0.75
−0.50
−0.25
0.00
0.25
0.50
0.75
1.00

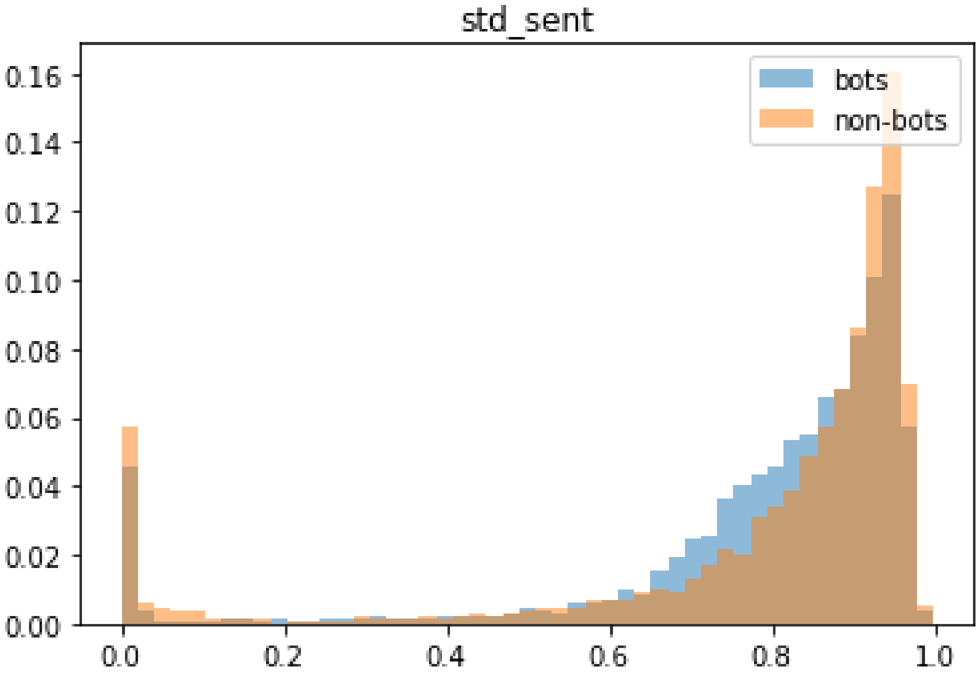
std_sent
bots
non-bots
0.16
0.14
0.12
0.10
0.08
0.06
0.04
0.02
0.00
0.0
0.2
0.4
0.6
0.8
1.0

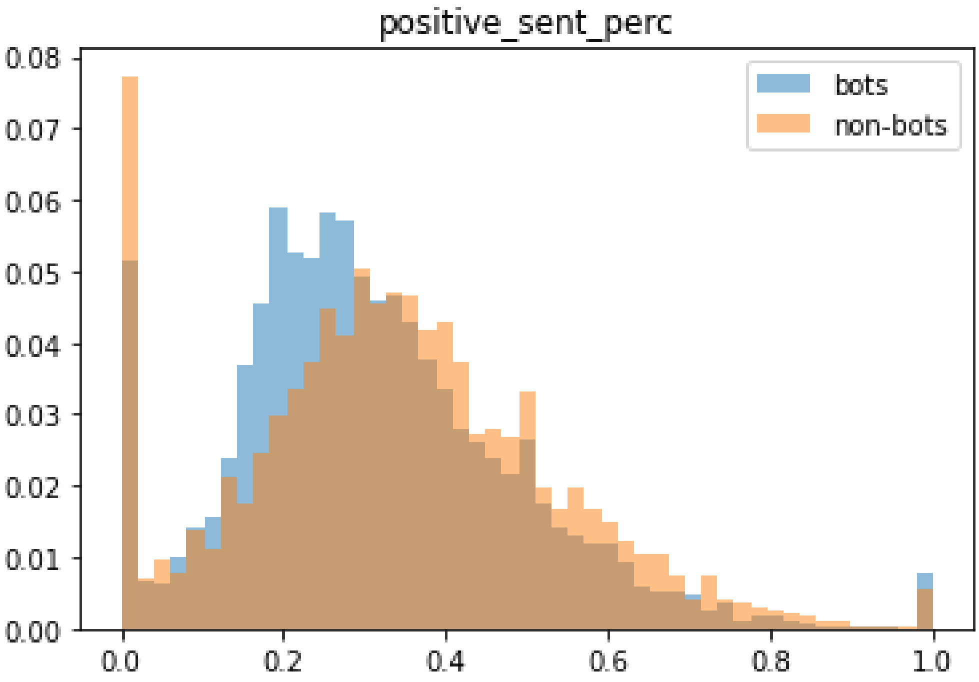

0.1.2 node statistics

[3]:
```
twibot_gf = pd.read_csv("twibot_20_gf.csv")
```

[4]:
```
twibot_gf = pd.read_csv("twibot_20_gf.csv")
for col in twibot_gf.columns[1:-1]:
    x = twibot_gf.loc[twibot_gf["label"] == 1, col]
    y = twibot_gf.loc[twibot_gf["label"] == 0, col]

    bins = np.linspace(min(twibot_gf[col]), max(twibot_gf[col]), 50)

    plt.hist(round(x, 5), bins, alpha=0.5, label='bots', weights=np.
 ↪ones(len(x))/len(x))
    plt.hist(y, bins, alpha=0.5, label='non-bots', weights=np.ones(len(y))/
 ↪len(y))
    plt.legend(loc='upper right')
    plt.title(col)
    #plt.savefig(f'./charts/{col}.pdf')
    plt.show()
```

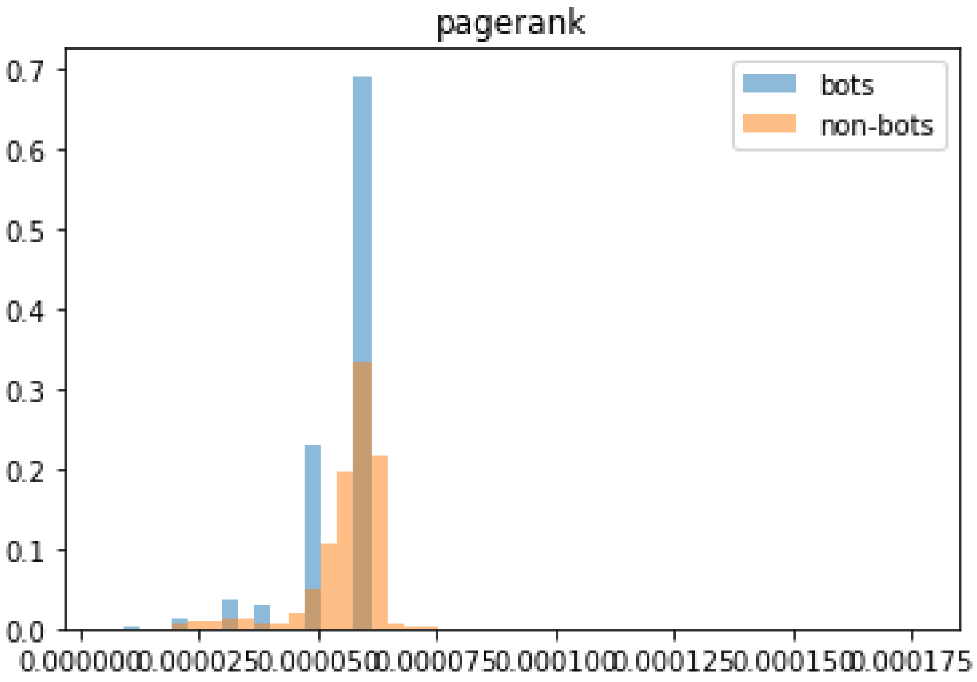
pagerank
bots
non-bots
0.7
0.6
0.5
0.4
0.3
0.2
0.1
0.0
0.000000
0.000025
0.000050
0.000075
0.000100
0.000125
0.000150
0.000175

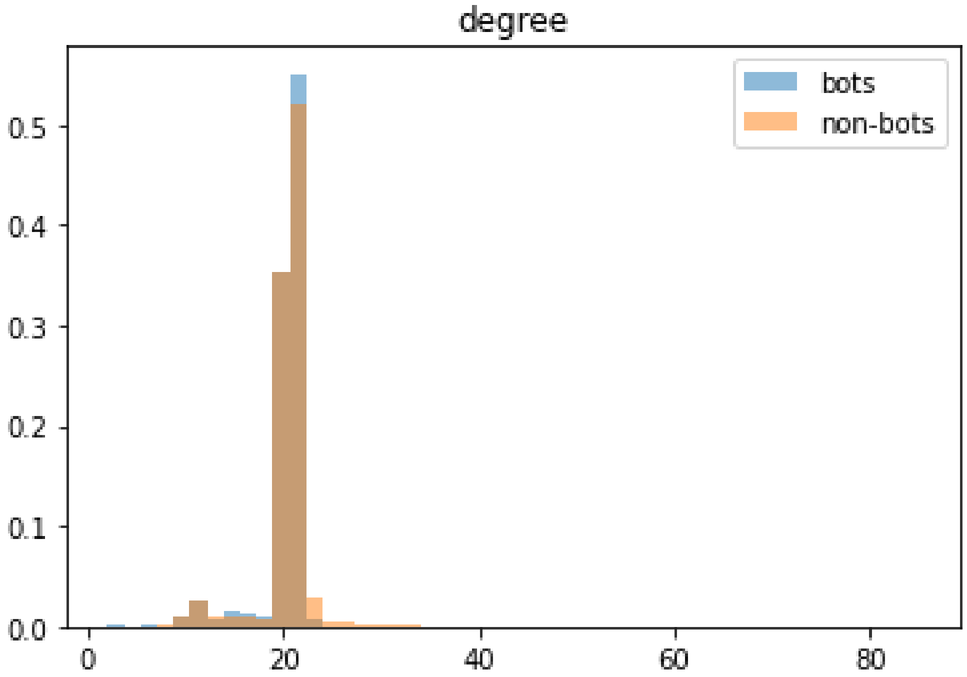
degree
bots
non-bots
0.5
0.4
0.3
0.2
0.1
0.0
0
20
40
60
80

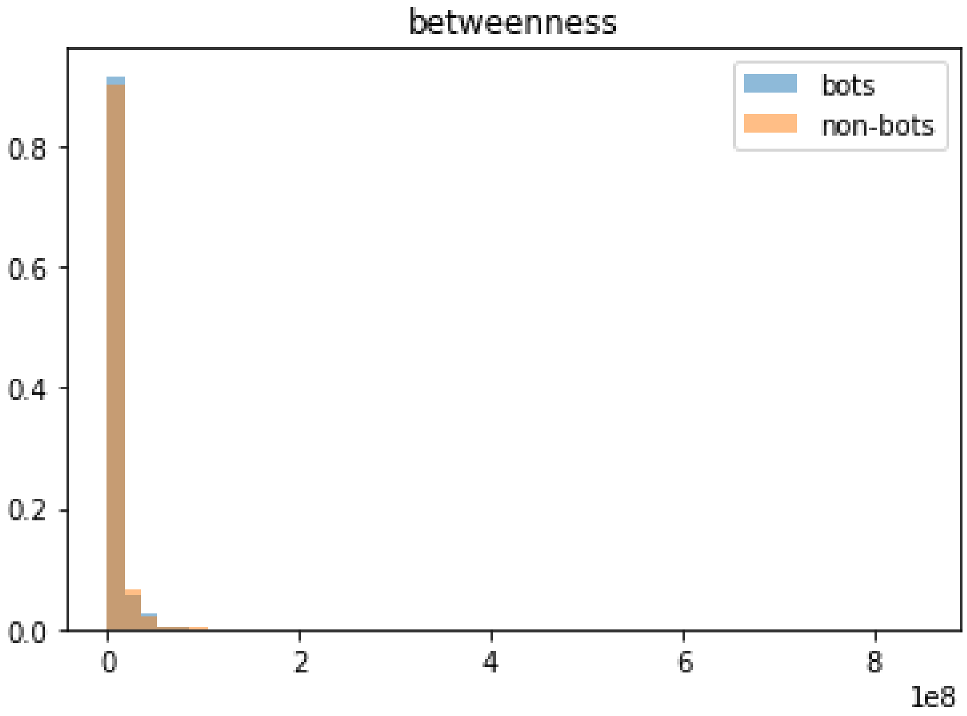
betweenness
bots
non-bots
0.8
0.6
0.4
0.2
0.0
0
2
4
6
8
1e8

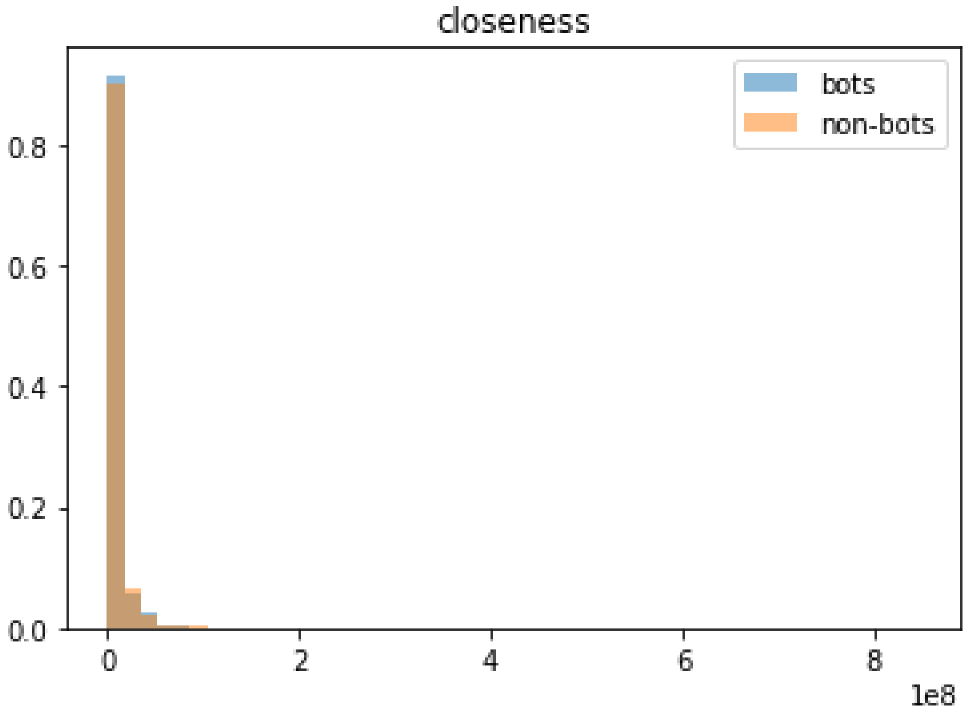
closeness
bots
non-bots
0.8
0.6
0.4
0.2
0.0
0
2
4
6
8
1e8

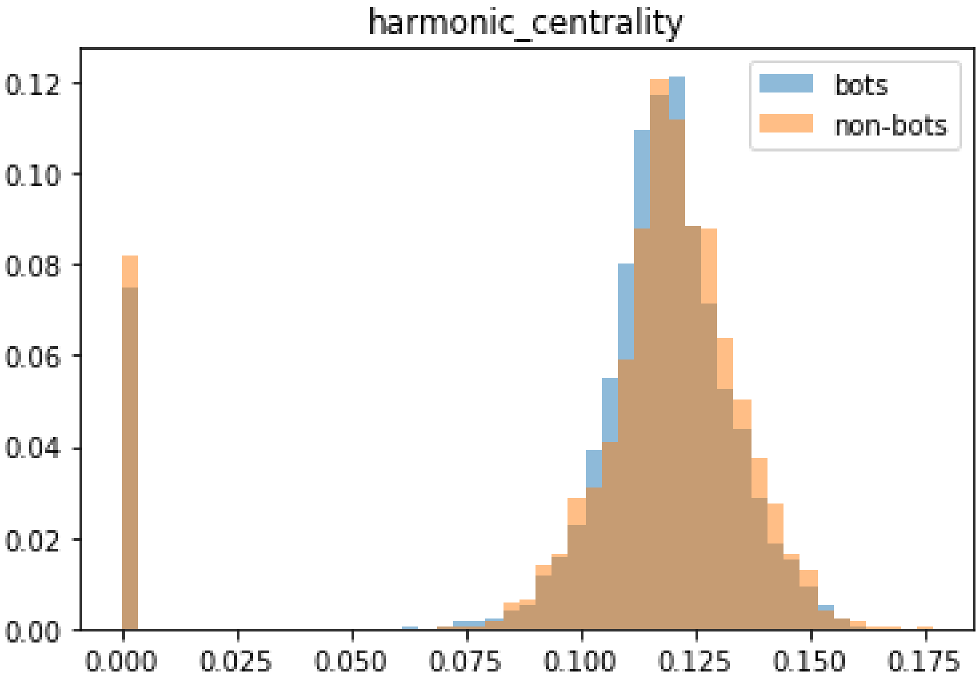
harmonic_centrality
bots
non-bots
0.00
0.02
0.04
0.06
0.08
0.10
0.12
0.000
0.025
0.050
0.075
0.100
0.125
0.150
0.175

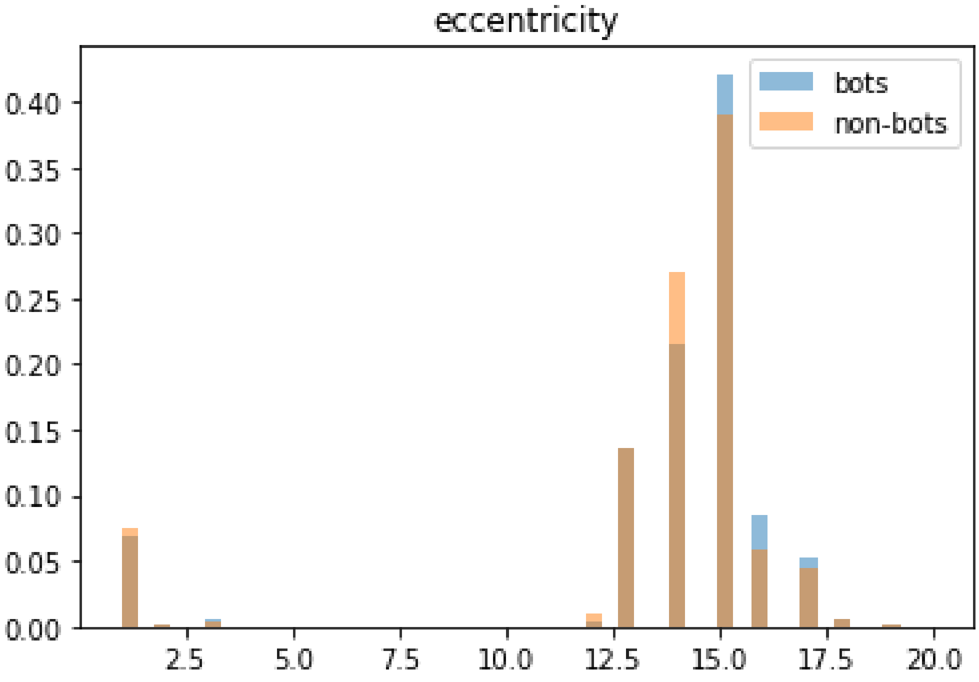
eccentricity
bots
non-bots
0.00
0.05
0.10
0.15
0.20
0.25
0.30
0.35
0.40
2.5
5.0
7.5
10.0
12.5
15.0
17.5
20.0

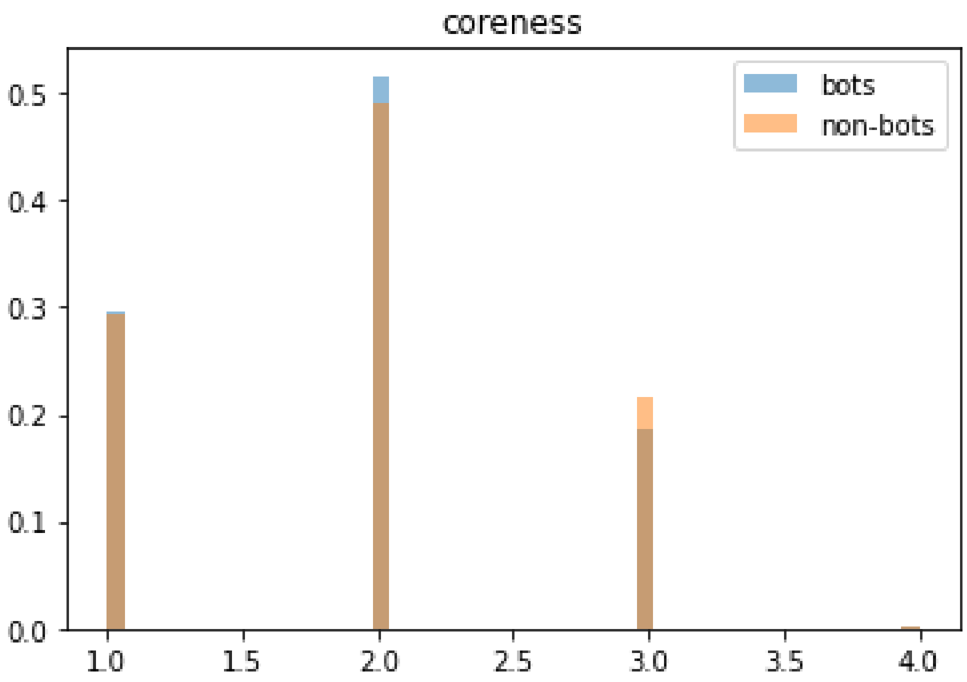
coreness
bots
non-bots
0.0
0.1
0.2
0.3
0.4
0.5
1.0
1.5
2.0
2.5
3.0
3.5
4.0

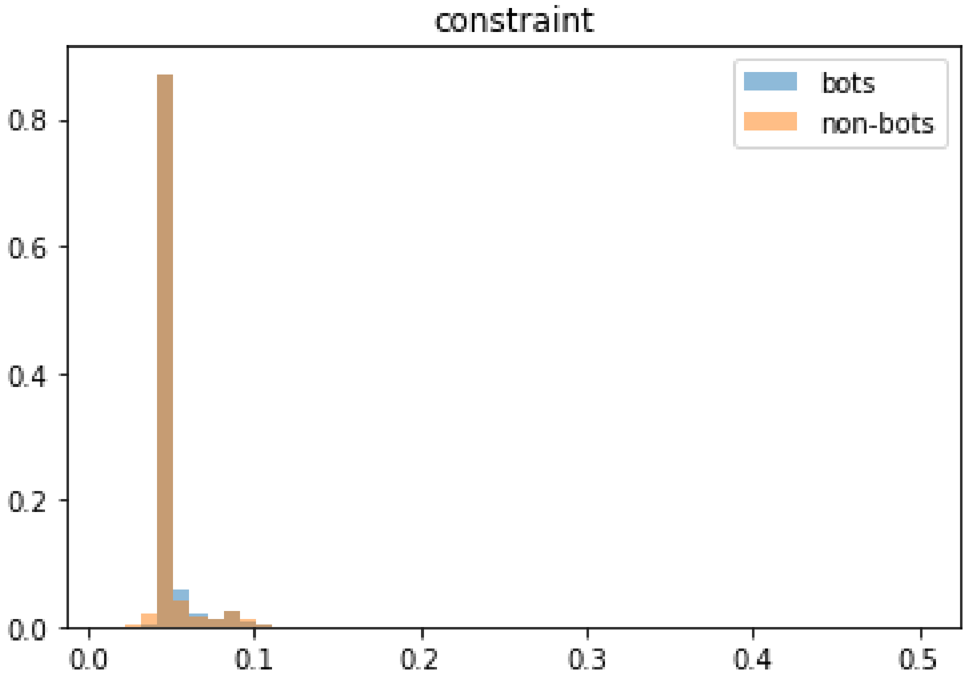
constraint
bots
non-bots
0.0
0.2
0.4
0.6
0.8
0.0
0.1
0.2
0.3
0.4
0.5

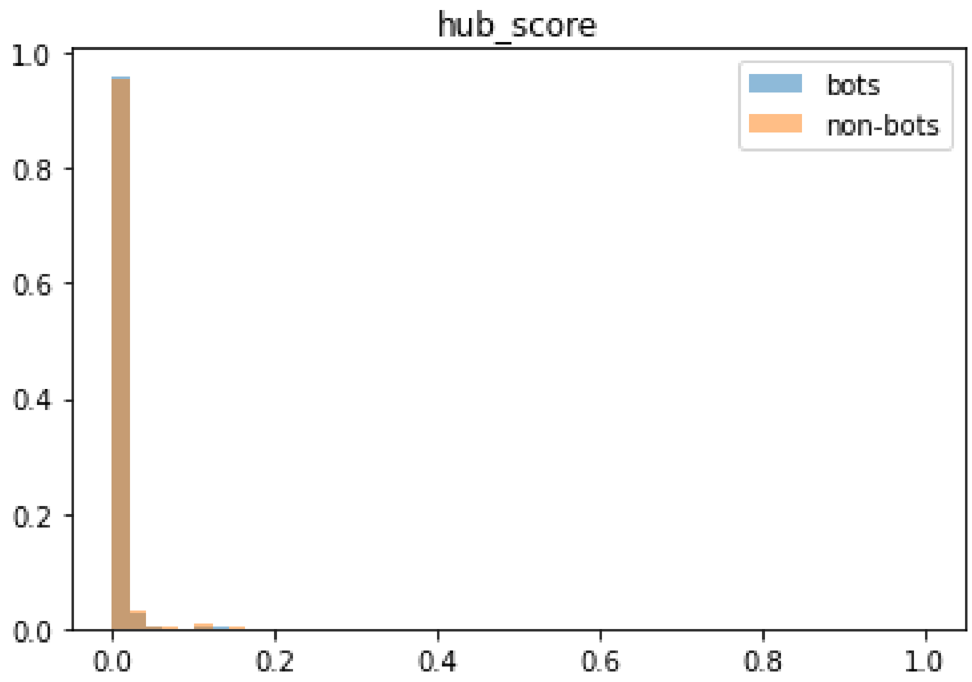
hub_score
bots
non-bots
1.0
0.8
0.6
0.4
0.2
0.0
0.0
0.2
0.4
0.6
0.8
1.0

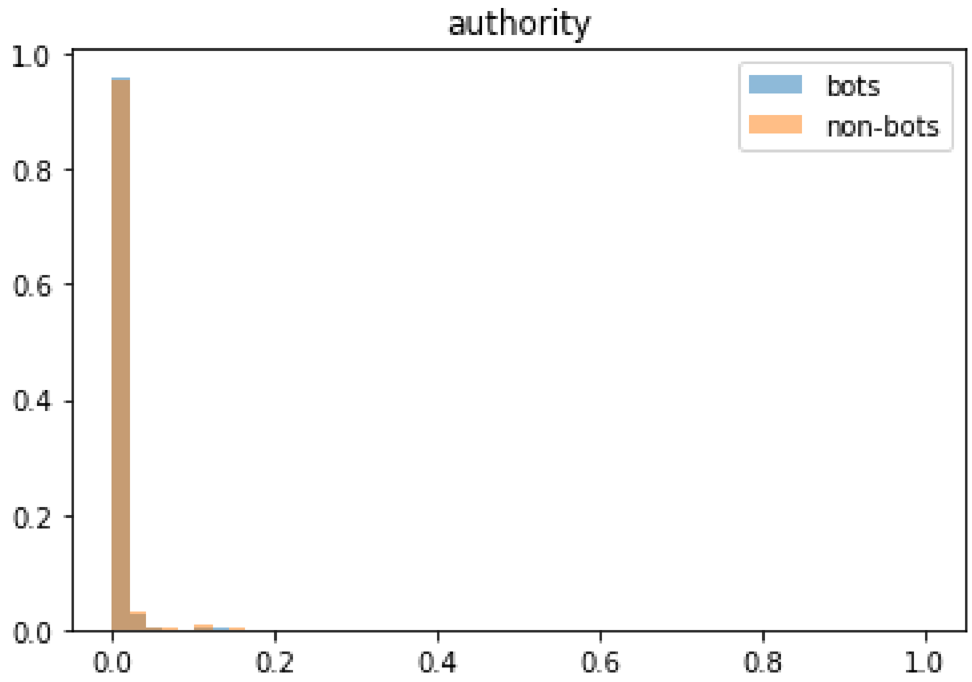
authority
bots
non-bots
1.0
0.8
0.6
0.4
0.2
0.0
0.0
0.2
0.4
0.6
0.8
1.0

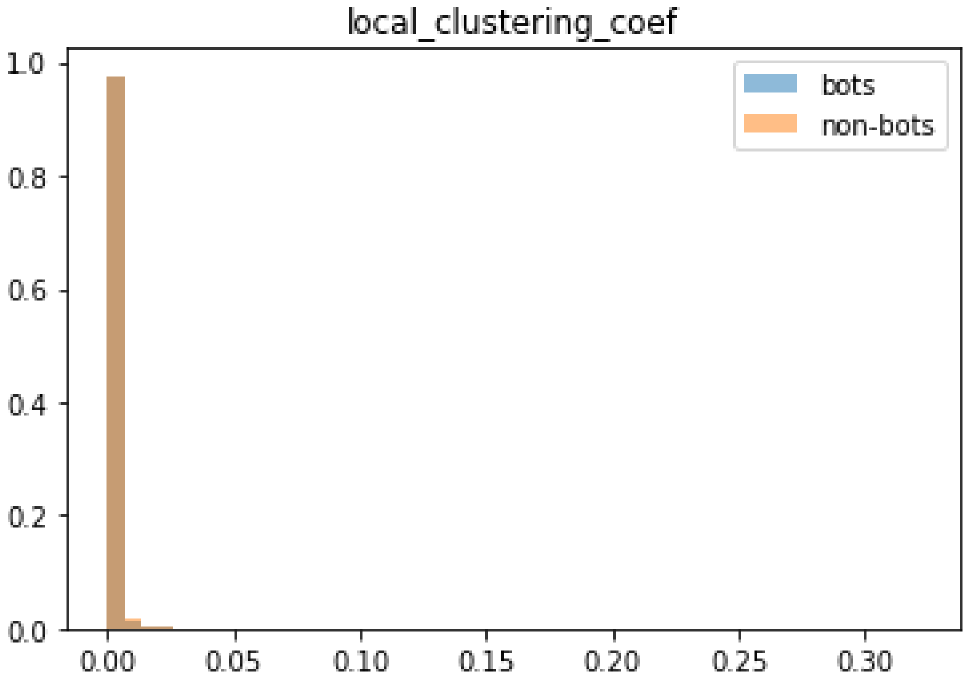
local_clustering_coef
bots
non-bots
1.0
0.8
0.6
0.4
0.2
0.0
0.00
0.05
0.10
0.15
0.20
0.25
0.30

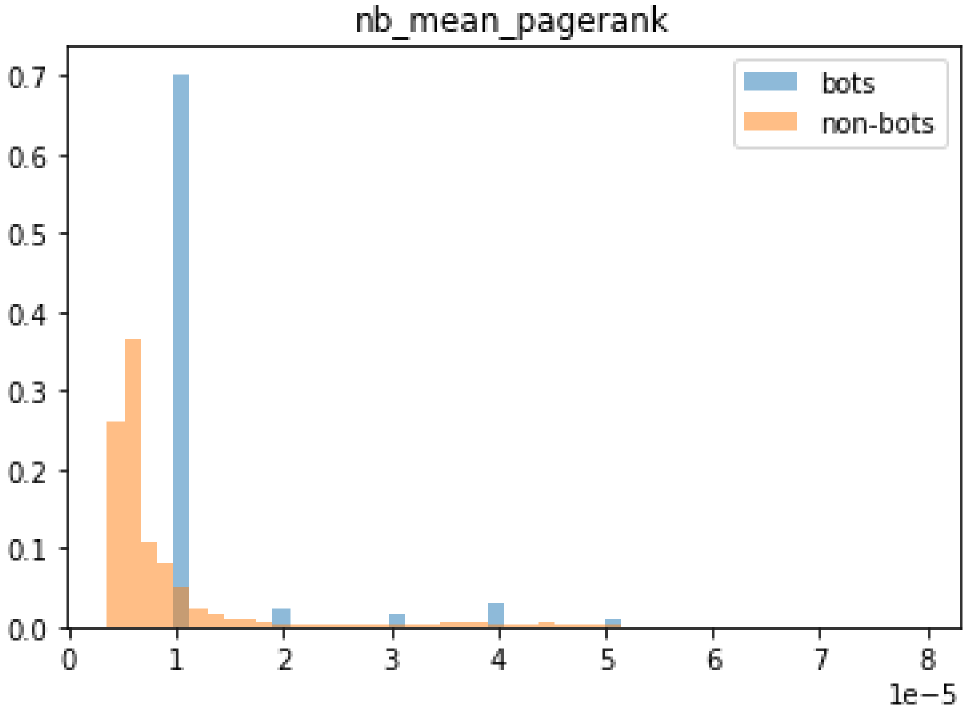
nb_mean_pagerank
bots
non-bots
0.7
0.6
0.5
0.4
0.3
0.2
0.1
0.0
0
1
2
3
4
5
6
7
8
1e−5

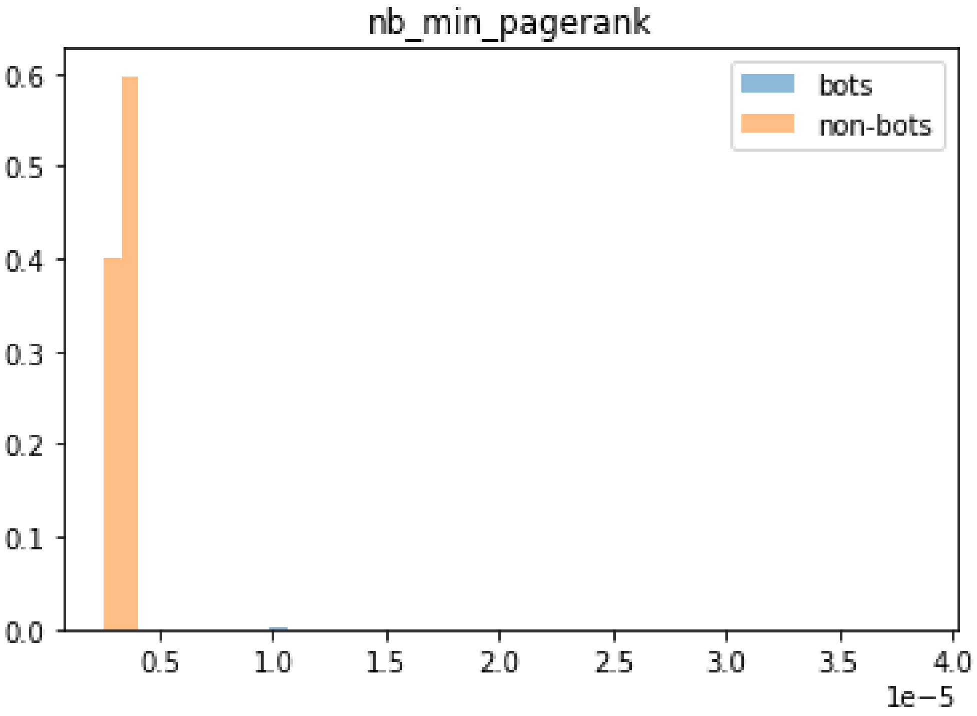
nb_min_pagerank
bots
non-bots
0.0
0.1
0.2
0.3
0.4
0.5
0.6
0.5
1.0
1.5
2.0
2.5
3.0
3.5
4.0
1e−5

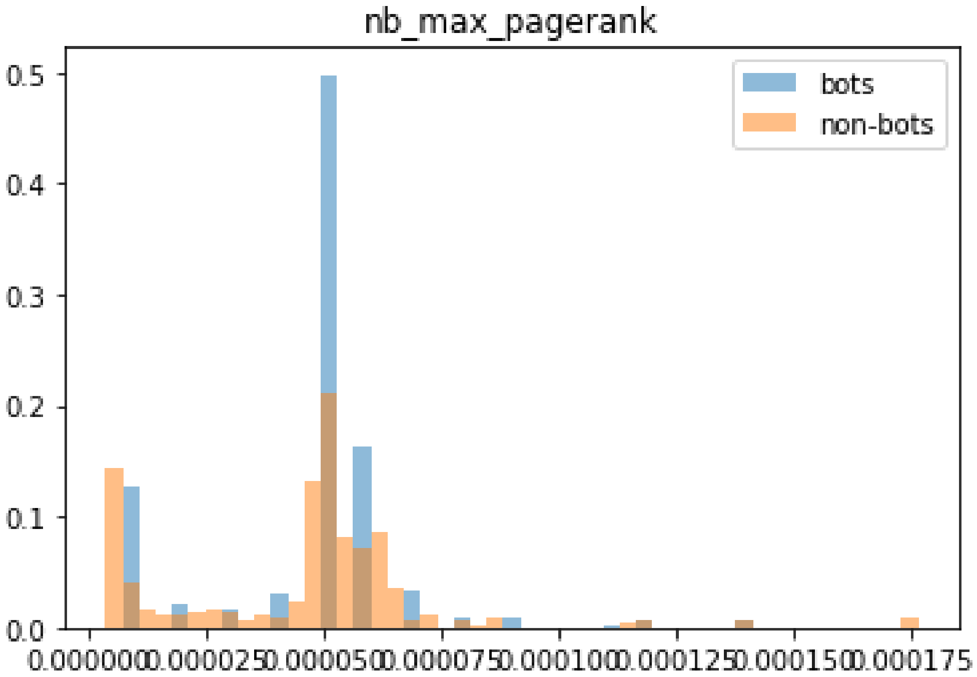
nb_max_pagerank
bots
non-bots
0.0
0.1
0.2
0.3
0.4
0.5
0.000000
0.000025
0.000050
0.000075
0.000100
0.000125
0.000150
0.000175

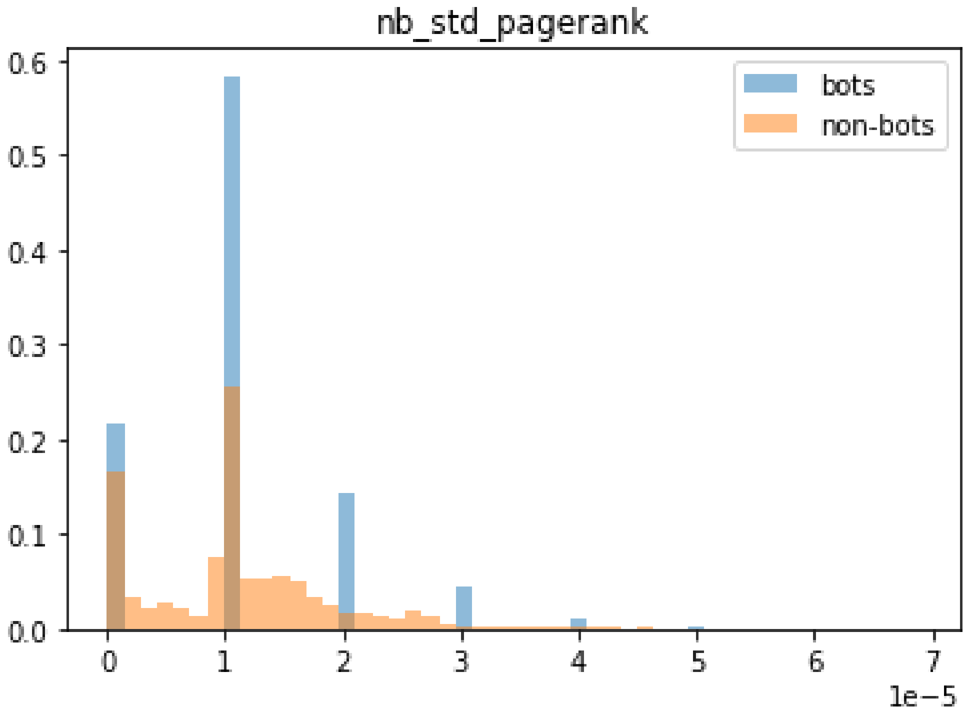
nb_std_pagerank
bots
non-bots
0.0
0.1
0.2
0.3
0.4
0.5
0.6
0
1
2
3
4
5
6
7
1e−5

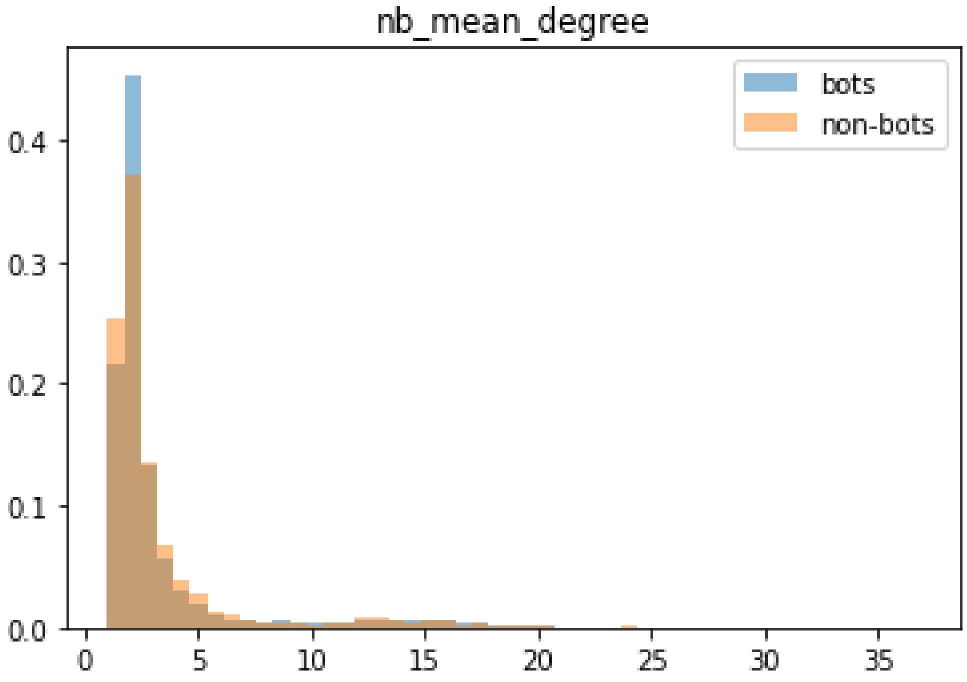
nb_mean_degree
bots
non-bots
0.0
0.1
0.2
0.3
0.4
0
5
10
15
20
25
30
35

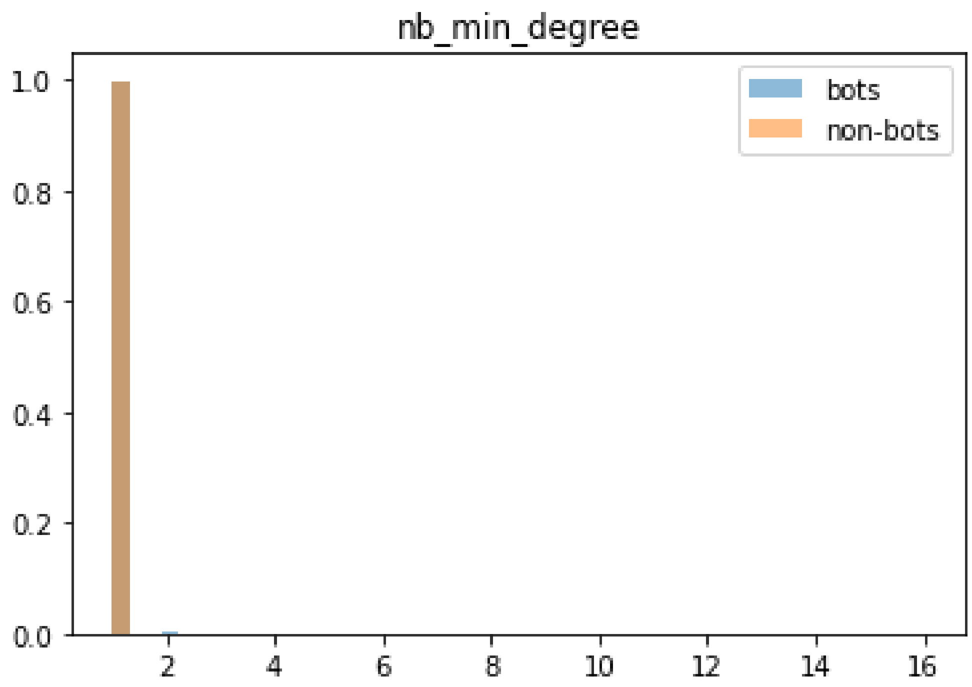
nb_min_degree
bots
non-bots
1.0
0.8
0.6
0.4
0.2
0.0
2
4
6
8
10
12
14
16

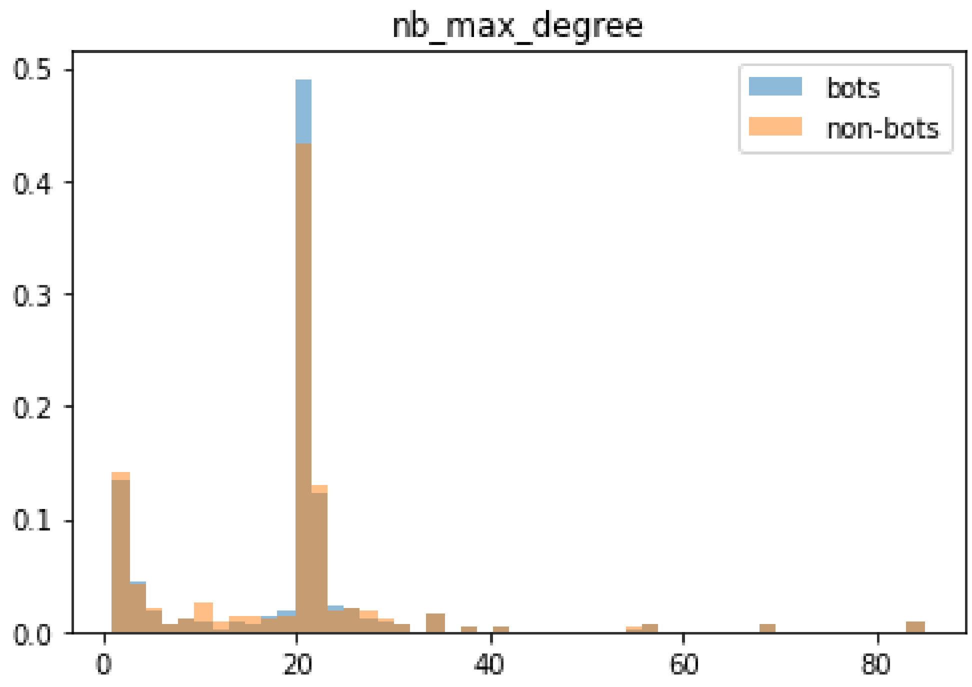
nb_max_degree
bots
non-bots
0.5
0.4
0.3
0.2
0.1
0.0
0
20
40
60
80

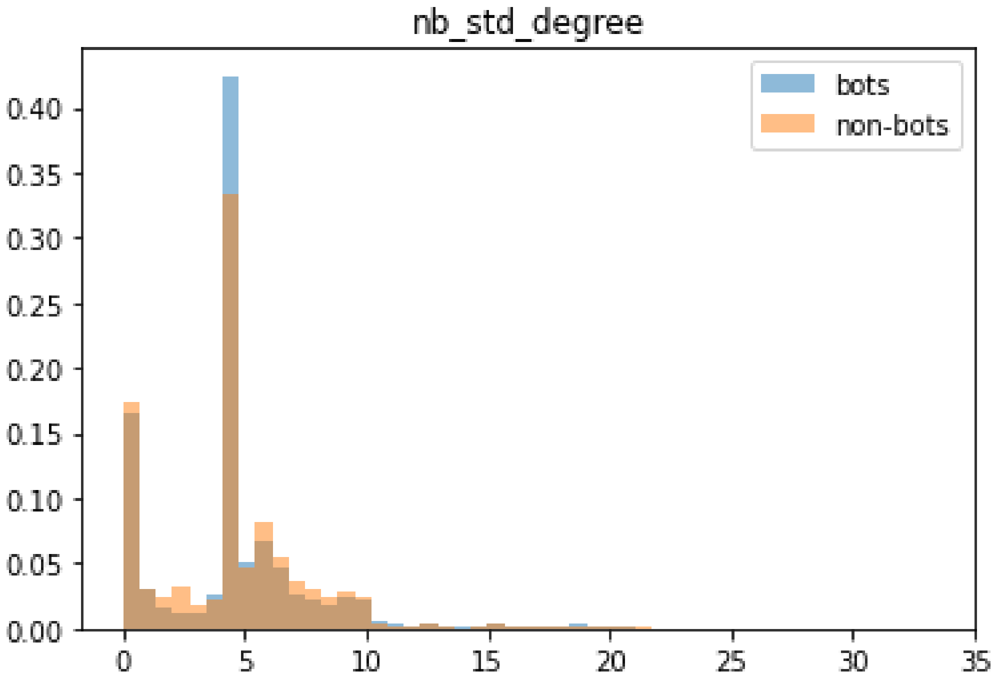
nb_std_degree
bots
non-bots
0.40
0.35
0.30
0.25
0.20
0.15
0.10
0.05
0.00
0
5
10
15
20
25
30
35

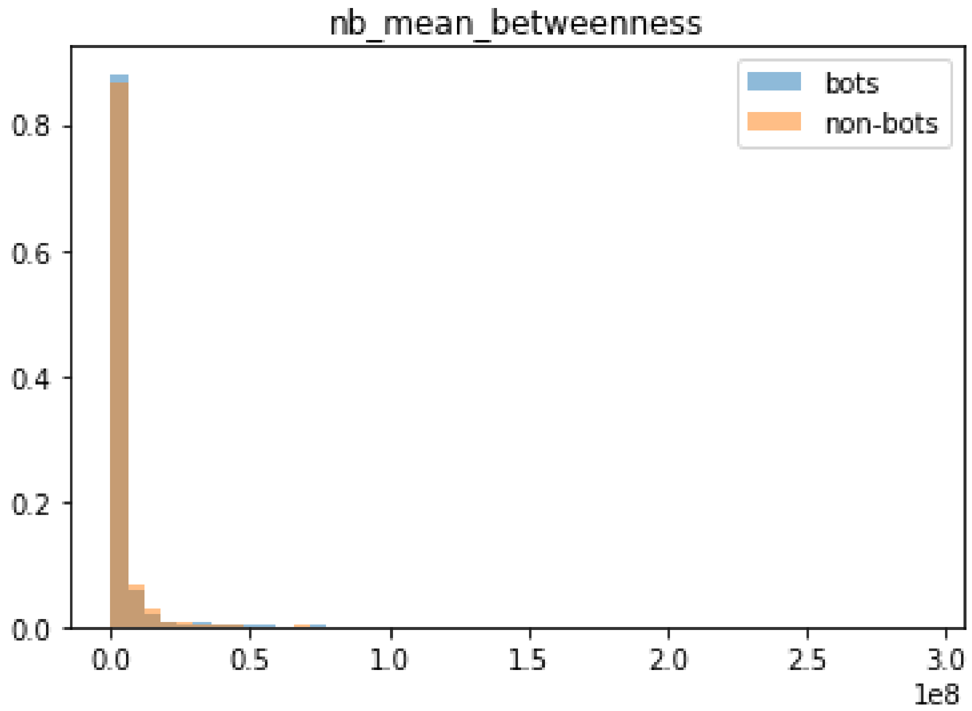
nb_mean_betweenness
bots
non-bots
0.8
0.6
0.4
0.2
0.0
0.0
0.5
1.0
1.5
2.0
2.5
3.0
1e8

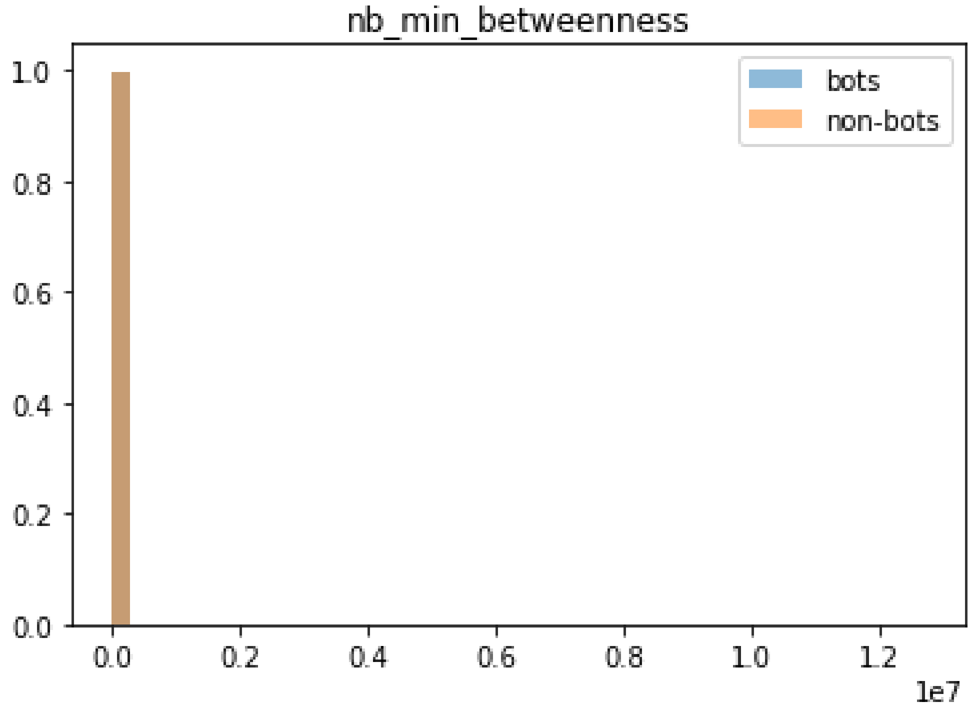
nb_min_betweenness
bots
non-bots
1.0
0.8
0.6
0.4
0.2
0.0
0.0
0.2
0.4
0.6
0.8
1.0
1.2
1e7

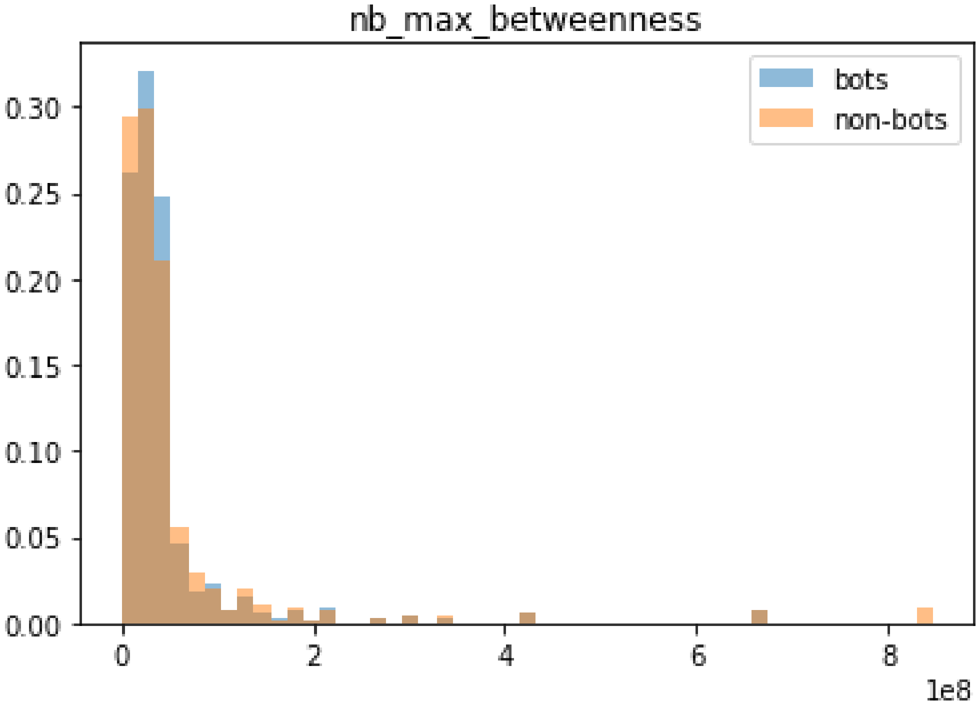
nb_max_betweenness
bots
non-bots
0.30
0.25
0.20
0.15
0.10
0.05
0.00
0
2
4
6
8
1e8

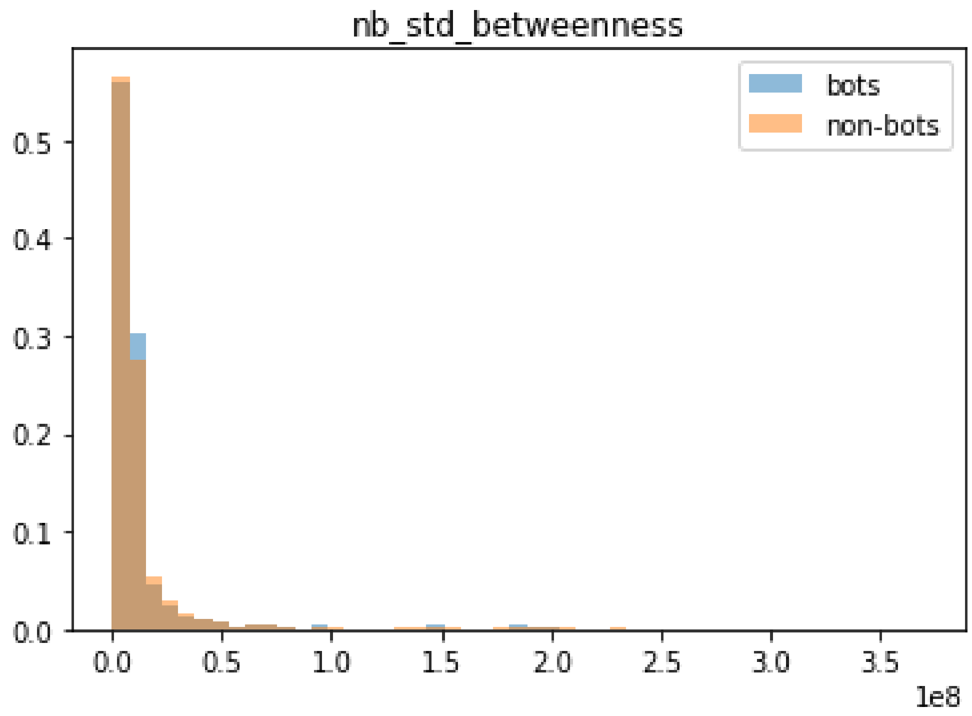
nb_std_betweenness
bots
non-bots
0.5
0.4
0.3
0.2
0.1
0.0
0.0
0.5
1.0
1.5
2.0
2.5
3.0
3.5
1e8

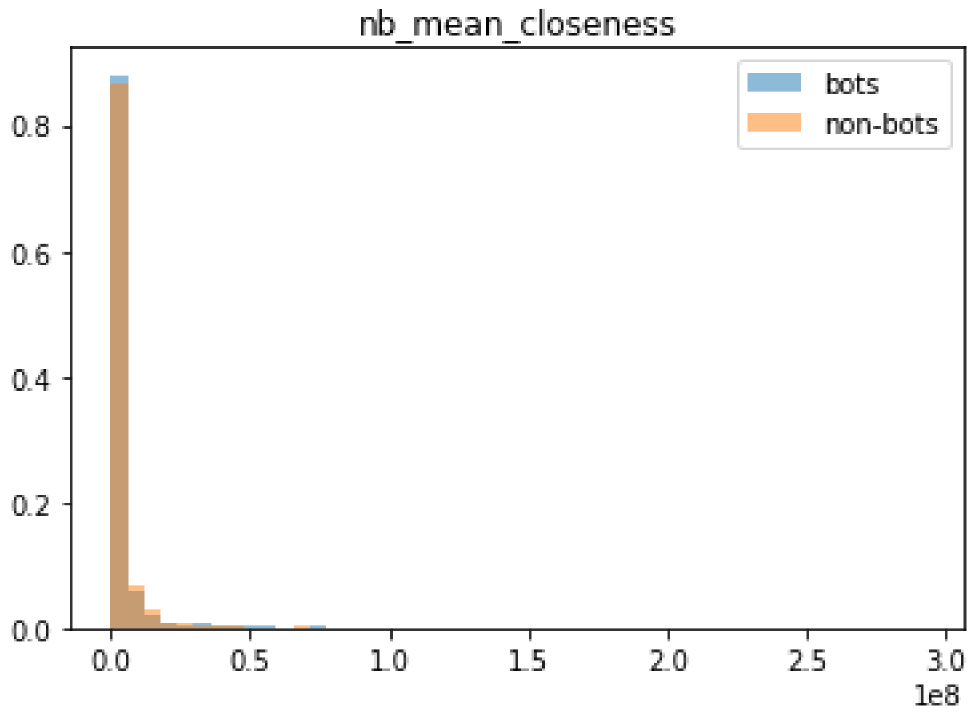
nb_mean_closeness
bots
non-bots
0.8
0.6
0.4
0.2
0.0
0.0
0.5
1.0
1.5
2.0
2.5
3.0
1e8

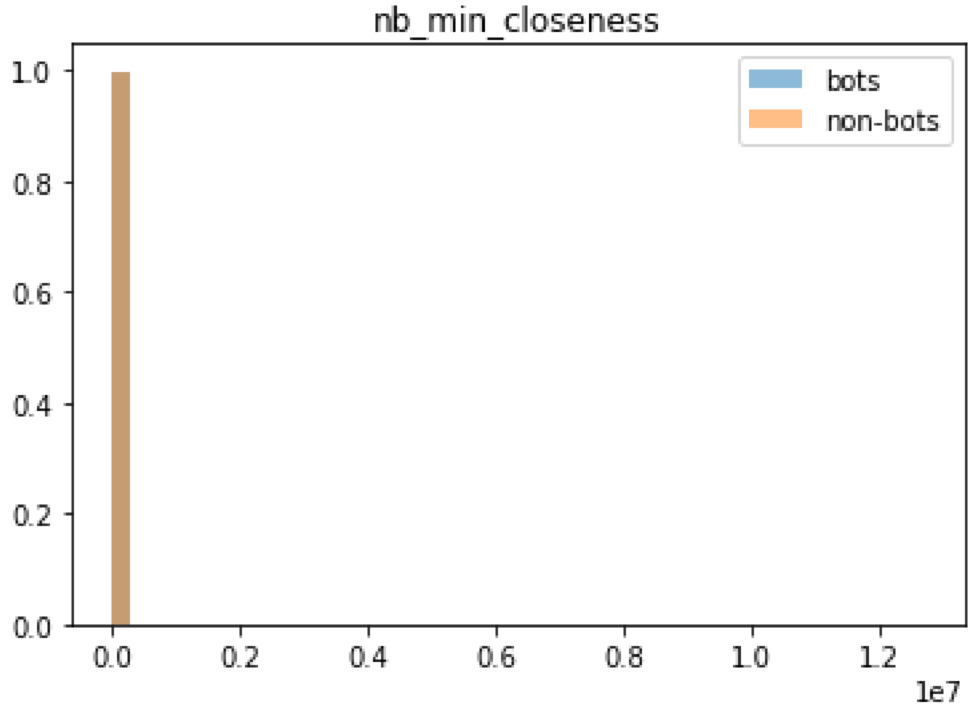
nb_min_closeness
bots
non-bots
1.0
0.8
0.6
0.4
0.2
0.0
0.0
0.2
0.4
0.6
0.8
1.0
1.2
1e7

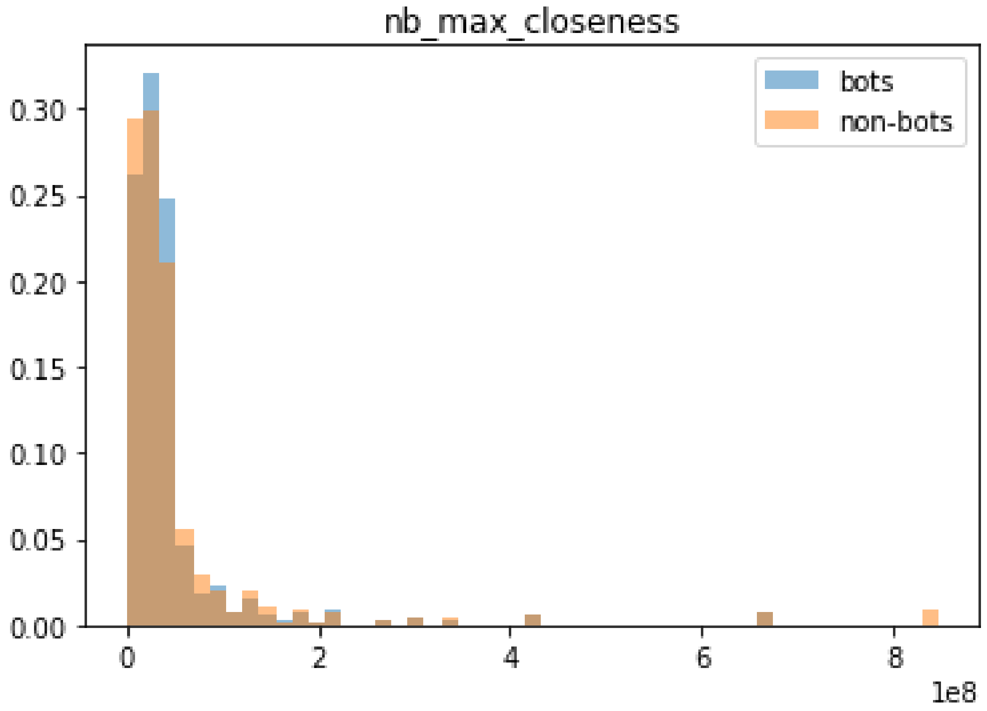
nb_max_closeness
bots
non-bots
0.30
0.25
0.20
0.15
0.10
0.05
0.00
0
2
4
6
8
1e8

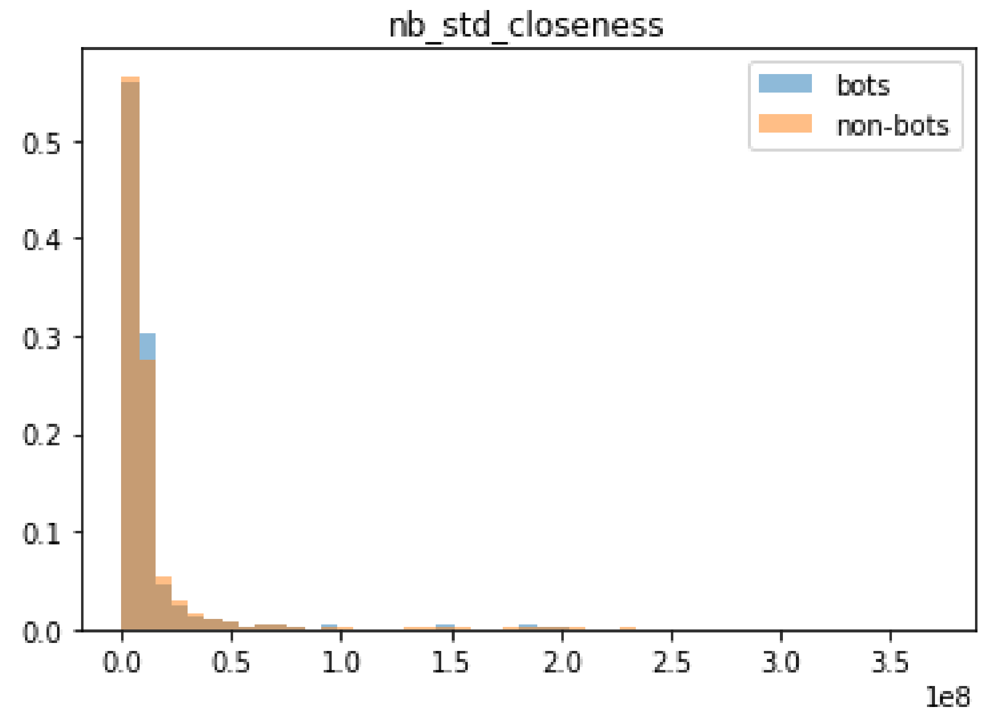
nb_std_closeness
bots
non-bots
0.5
0.4
0.3
0.2
0.1
0.0
0.0
0.5
1.0
1.5
2.0
2.5
3.0
3.5
1e8

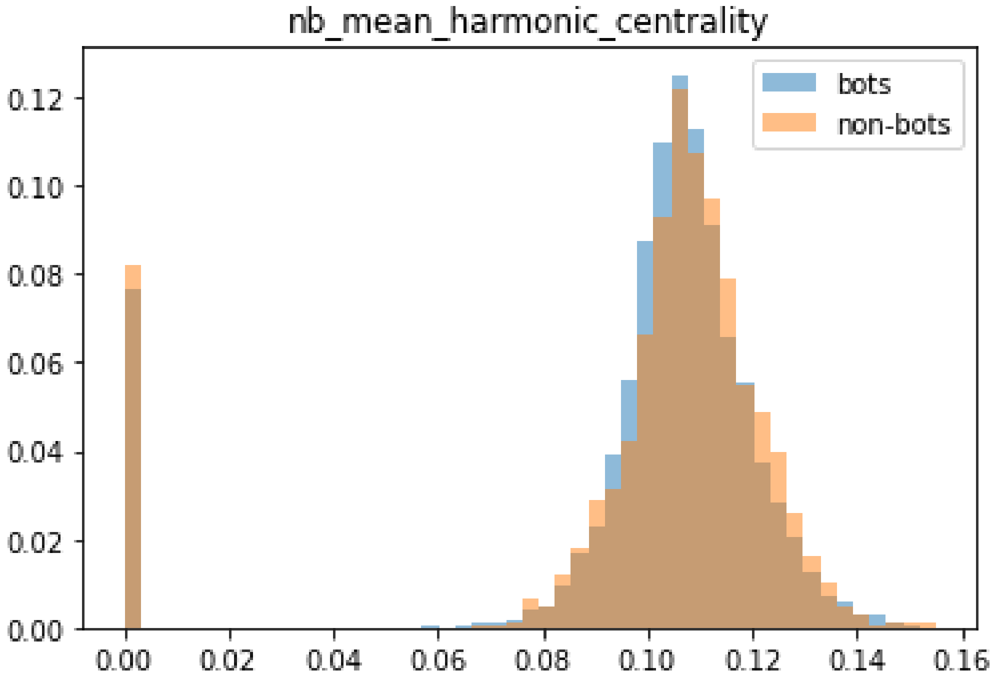
nb_mean_harmonic_centrality
bots
non-bots
0.12
0.10
0.08
0.06
0.04
0.02
0.00
0.00
0.02
0.04
0.06
0.08
0.10
0.12
0.14
0.16

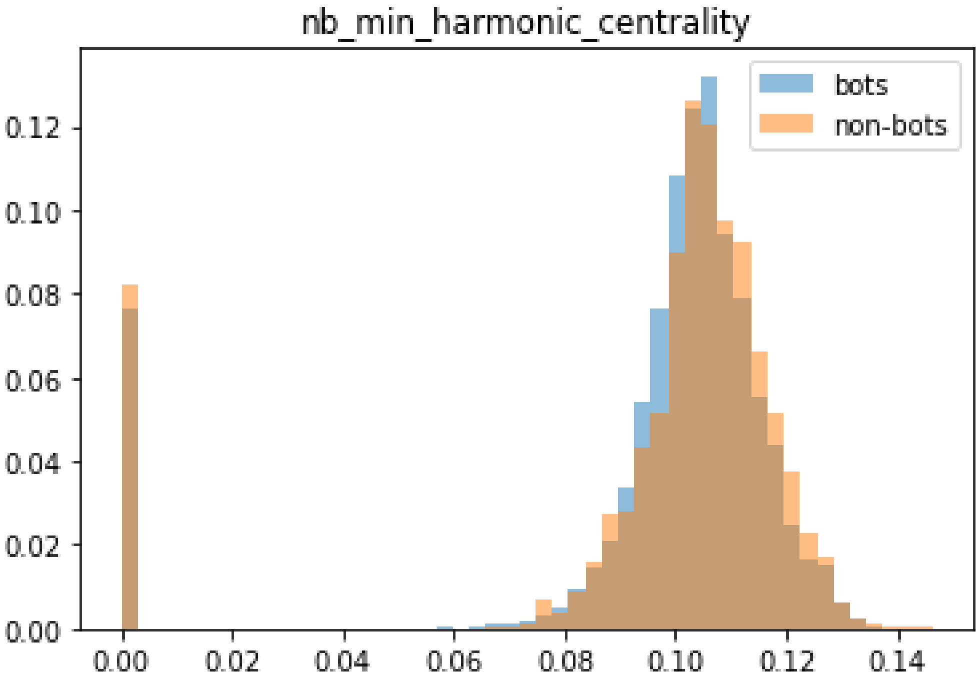
nb_min_harmonic_centrality
bots
non-bots
0.12
0.10
0.08
0.06
0.04
0.02
0.00
0.00
0.02
0.04
0.06
0.08
0.10
0.12
0.14

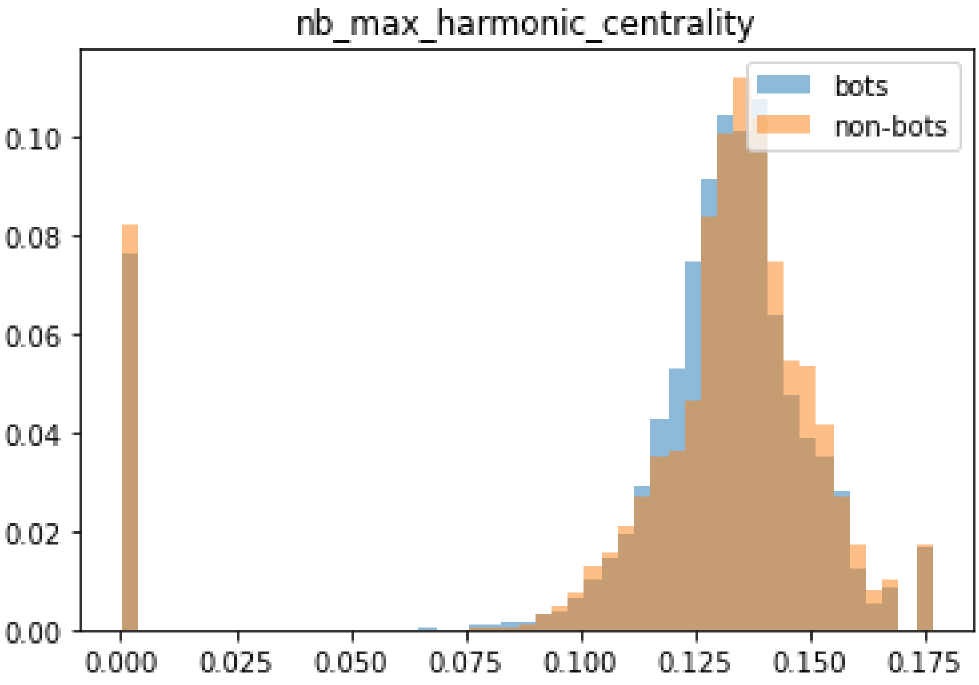
nb_max_harmonic_centrality
bots
non-bots
0.10
0.08
0.06
0.04
0.02
0.00
0.000
0.025
0.050
0.075
0.100
0.125
0.150
0.175

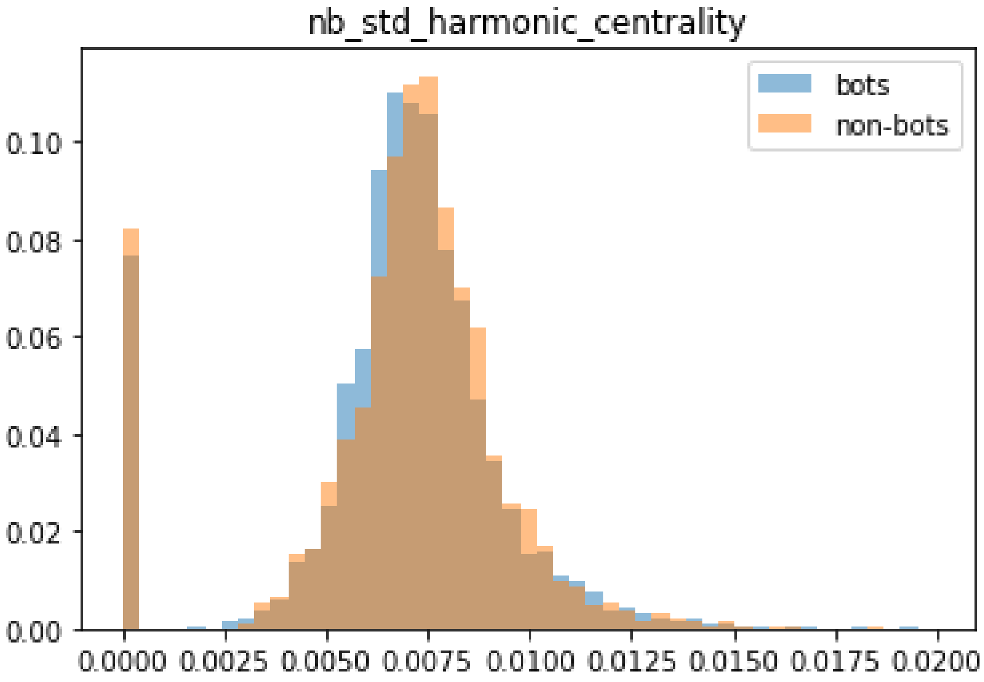
nb_std_harmonic_centrality
bots
non-bots
0.10
0.08
0.06
0.04
0.02
0.00
0.0000
0.0025
0.0050
0.0075
0.0100
0.0125
0.0150
0.0175
0.0200

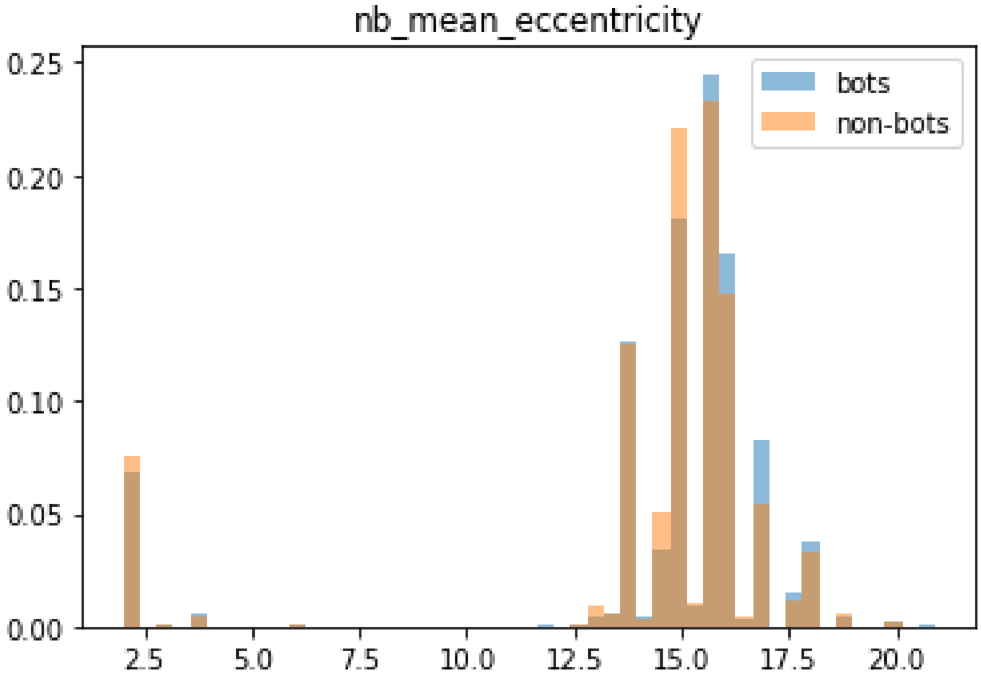
nb_mean_eccentricity
bots
non-bots
0.25
0.20
0.15
0.10
0.05
0.00
2.5
5.0
7.5
10.0
12.5
15.0
17.5
20.0

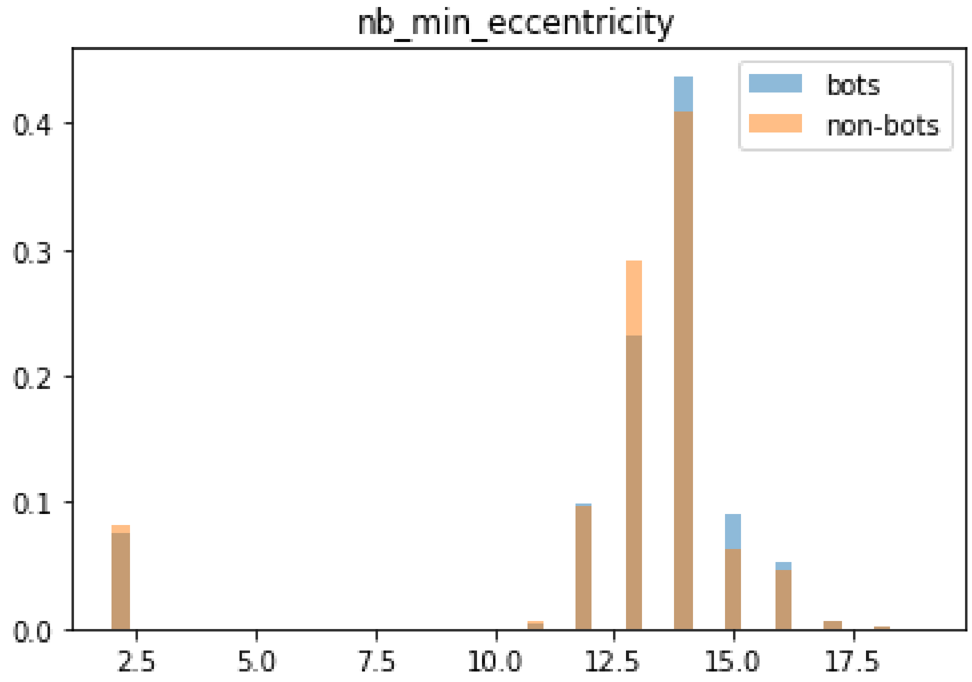
nb_min_eccentricity
bots
non-bots
0.0
0.1
0.2
0.3
0.4
2.5
5.0
7.5
10.0
12.5
15.0
17.5

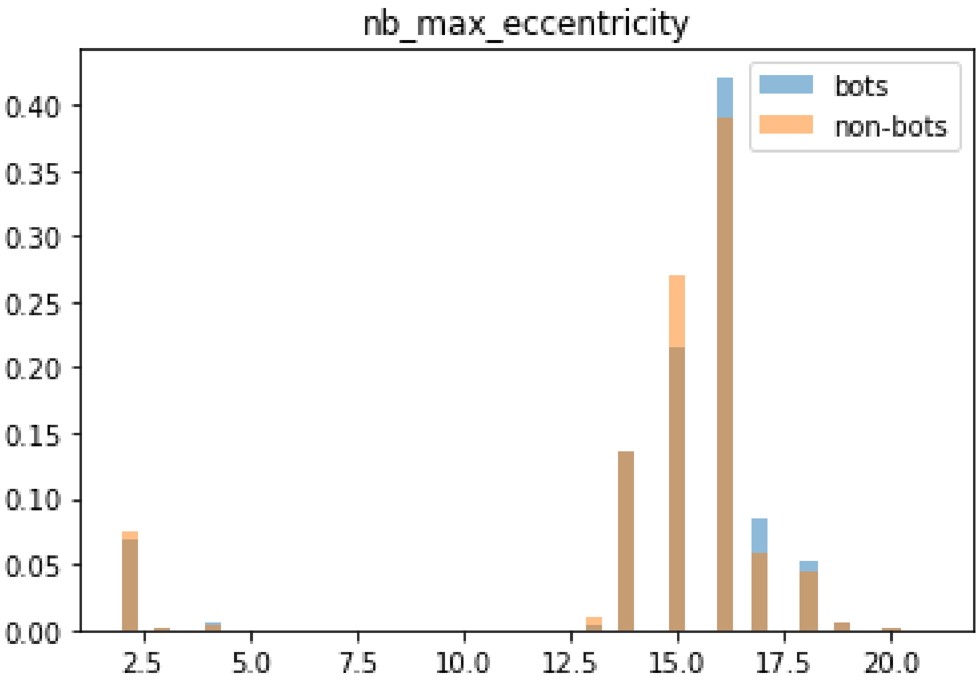
nb_max_eccentricity
bots
non-bots
0.00
0.05
0.10
0.15
0.20
0.25
0.30
0.35
0.40
2.5
5.0
7.5
10.0
12.5
15.0
17.5
20.0

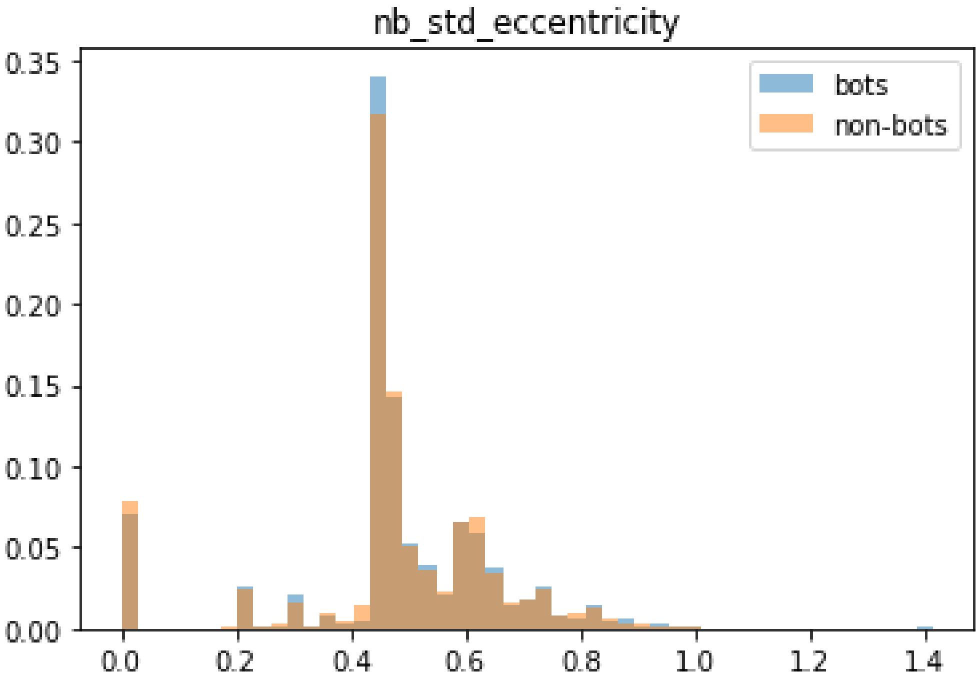
nb_std_eccentricity
bots
non-bots
0.35
0.30
0.25
0.20
0.15
0.10
0.05
0.00
0.0
0.2
0.4
0.6
0.8
1.0
1.2
1.4

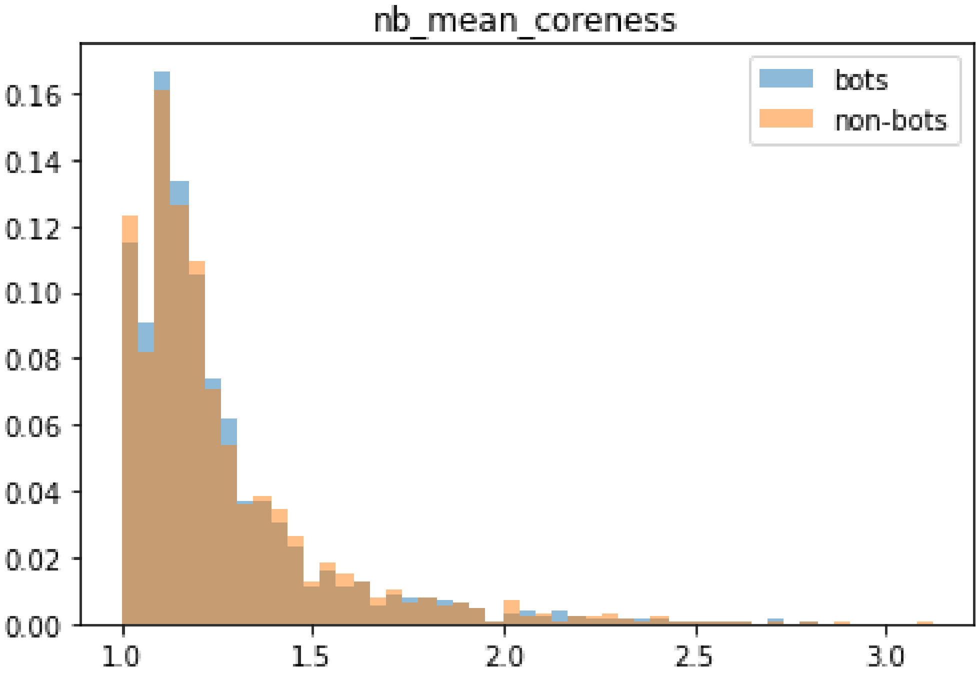
nb_mean_coreness
bots
non-bots
0.16
0.14
0.12
0.10
0.08
0.06
0.04
0.02
0.00
1.0
1.5
2.0
2.5
3.0

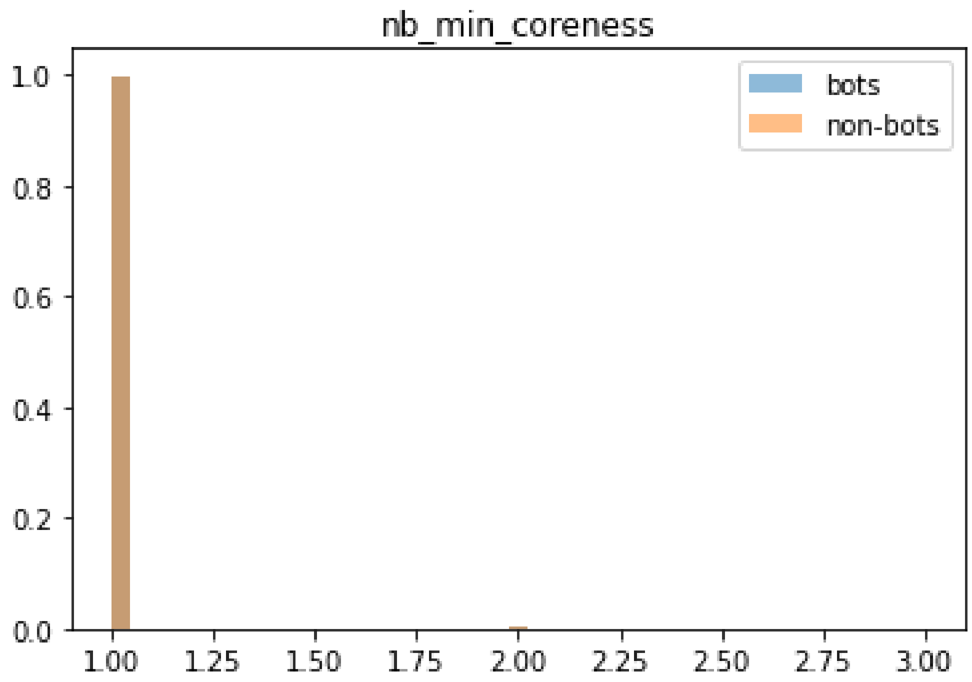
nb_min_coreness
bots
non-bots
1.0
0.8
0.6
0.4
0.2
0.0
1.00
1.25
1.50
1.75
2.00
2.25
2.50
2.75
3.00

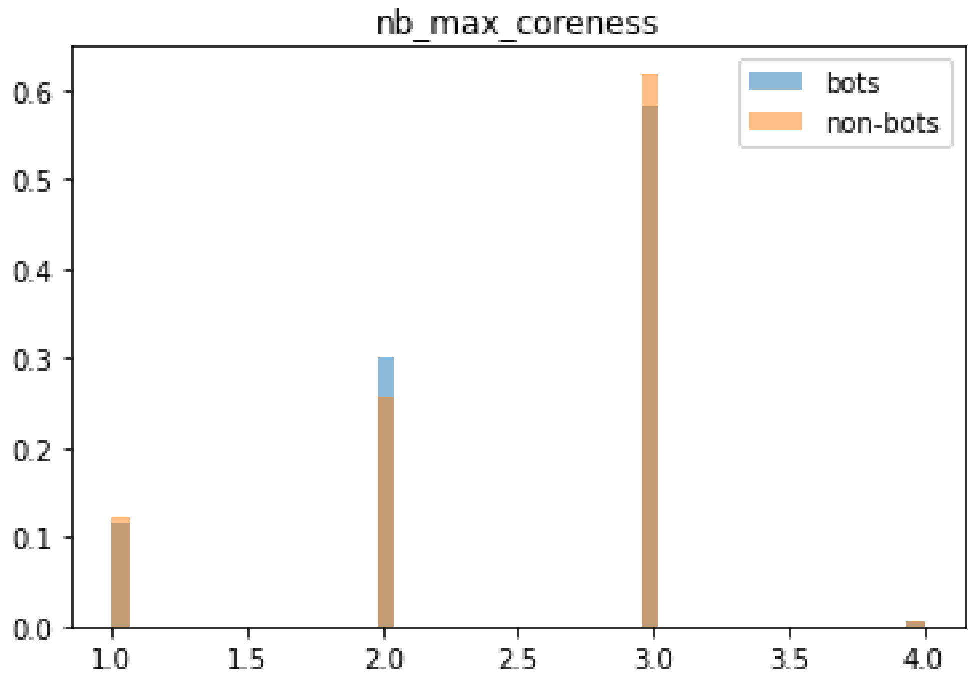
nb_max_coreness
bots
non-bots
0.6
0.5
0.4
0.3
0.2
0.1
0.0
1.0
1.5
2.0
2.5
3.0
3.5
4.0

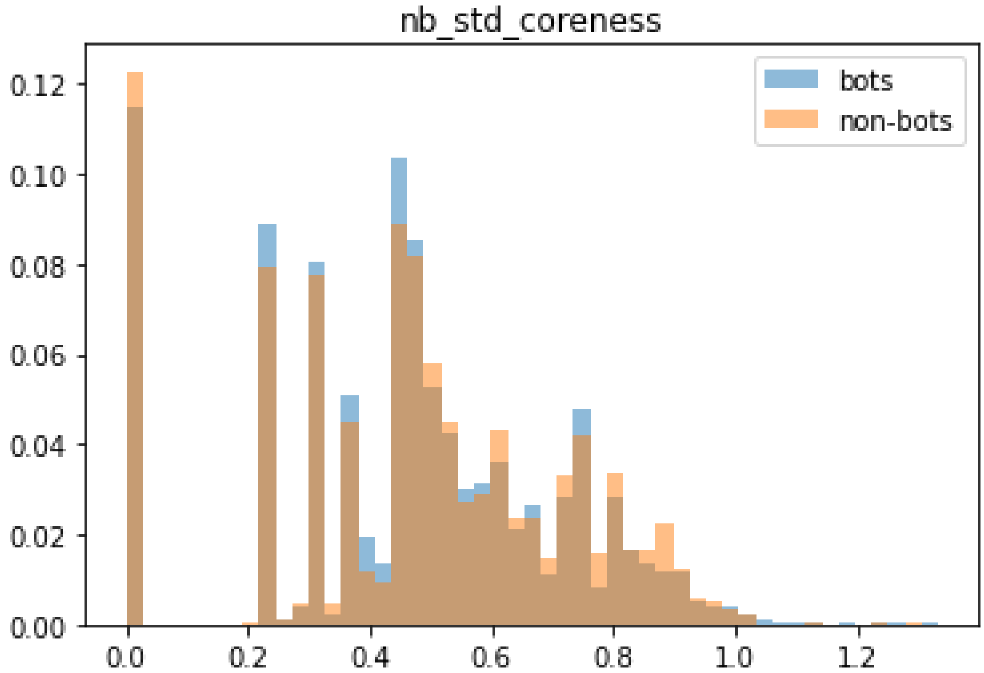
nb_std_coreness
bots
non-bots
0.12
0.10
0.08
0.06
0.04
0.02
0.00
0.0
0.2
0.4
0.6
0.8
1.0
1.2

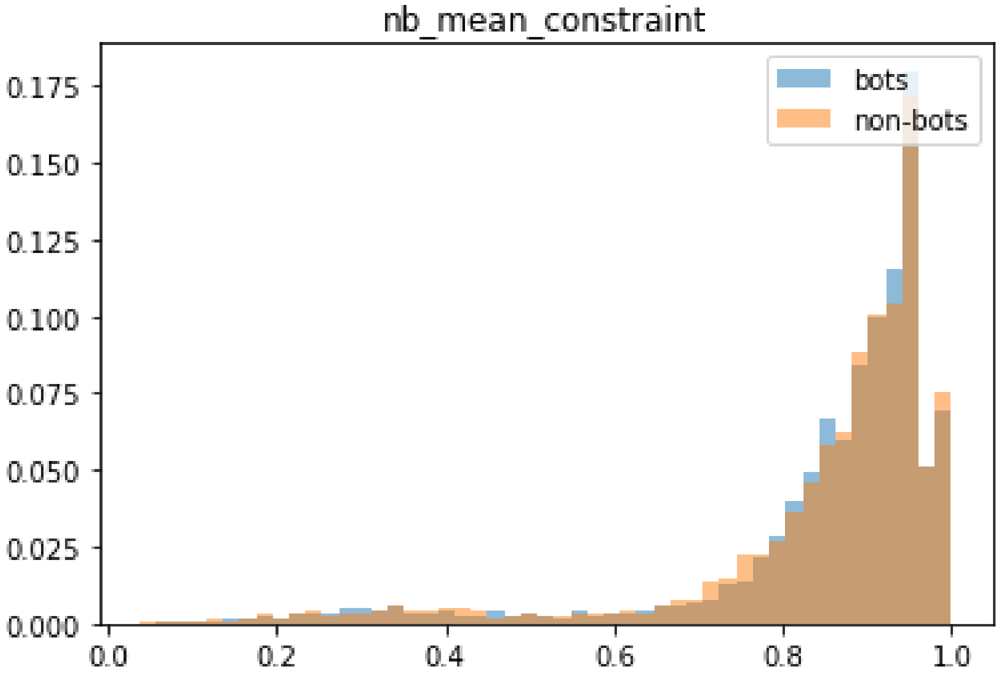
nb_mean_constraint
bots
non-bots
0.175
0.150
0.125
0.100
0.075
0.050
0.025
0.000
0.0
0.2
0.4
0.6
0.8
1.0

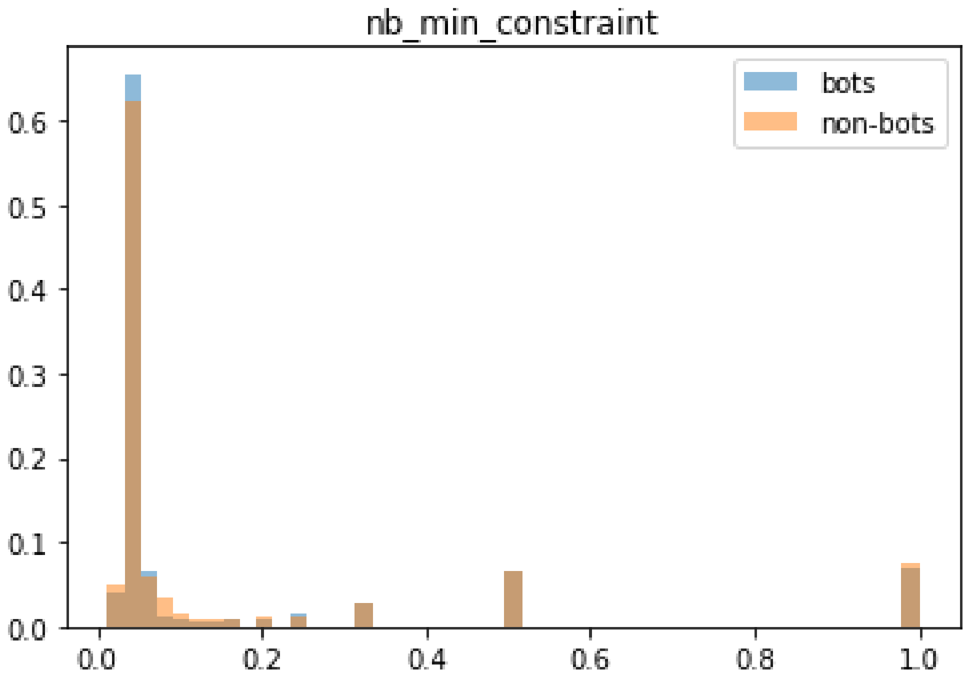
nb_min_constraint
bots
non-bots
0.6
0.5
0.4
0.3
0.2
0.1
0.0
0.0
0.2
0.4
0.6
0.8
1.0

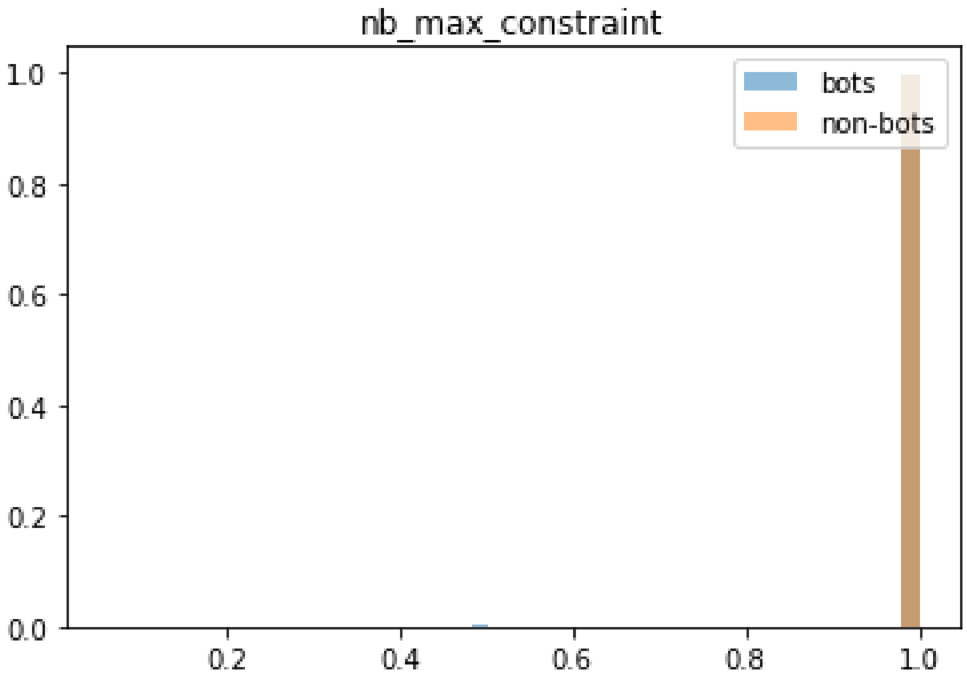
nb_max_constraint
bots
non-bots
1.0
0.8
0.6
0.4
0.2
0.0
0.2
0.4
0.6
0.8
1.0

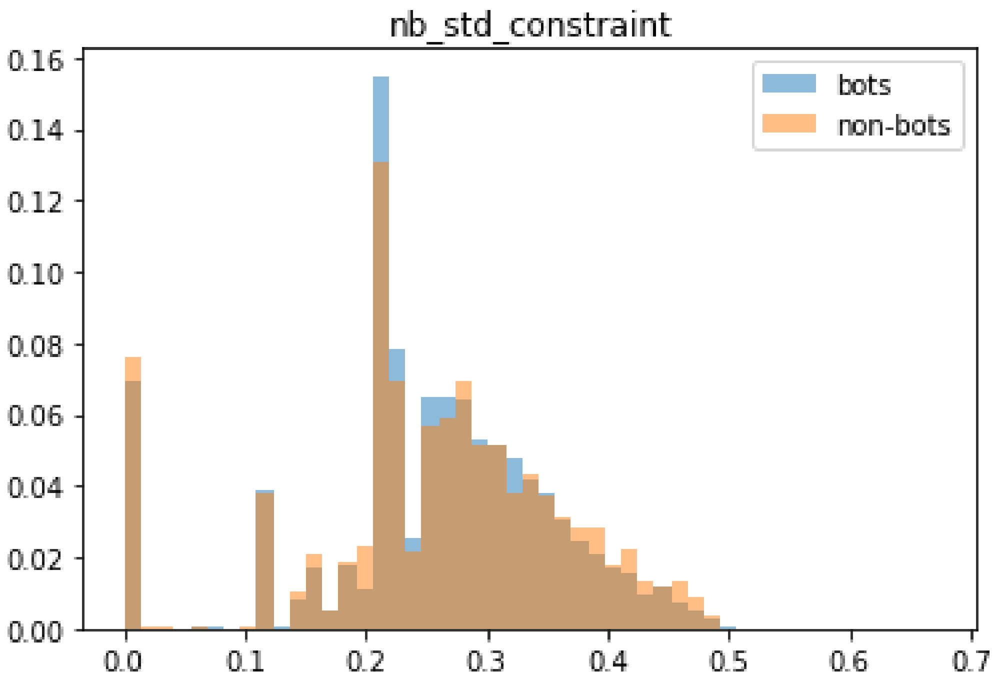
nb_std_constraint
bots
non-bots
0.16
0.14
0.12
0.10
0.08
0.06
0.04
0.02
0.00
0.0
0.1
0.2
0.3
0.4
0.5
0.6
0.7

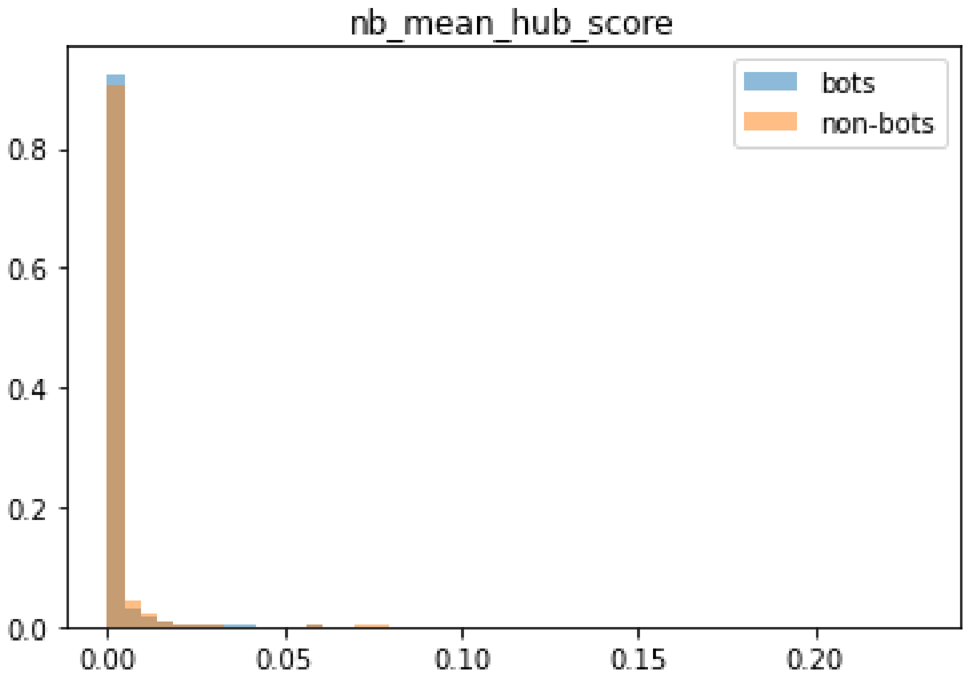
nb_mean_hub_score
bots
non-bots
0.8
0.6
0.4
0.2
0.0
0.00
0.05
0.10
0.15
0.20

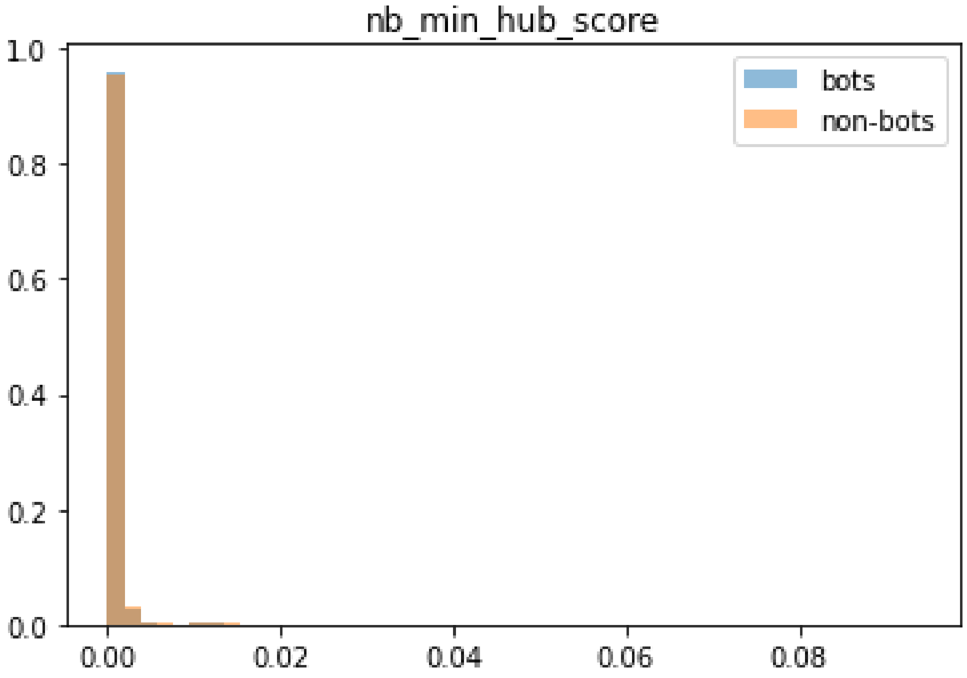
nb_min_hub_score
bots
non-bots
1.0
0.8
0.6
0.4
0.2
0.0
0.00
0.02
0.04
0.06
0.08

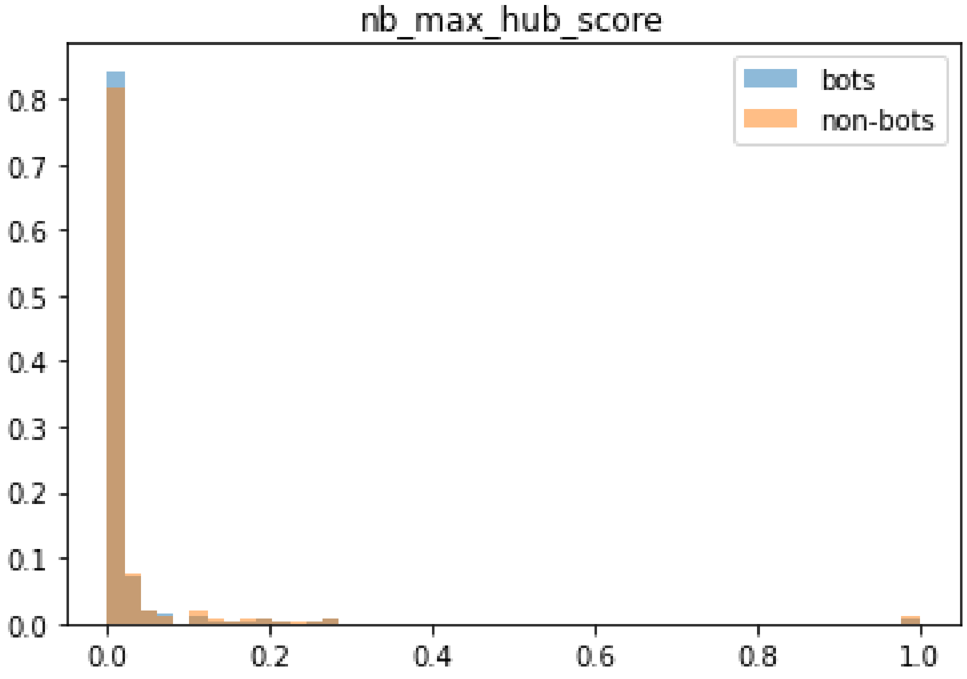
nb_max_hub_score
bots
non-bots
0.8
0.7
0.6
0.5
0.4
0.3
0.2
0.1
0.0
0.0
0.2
0.4
0.6
0.8
1.0

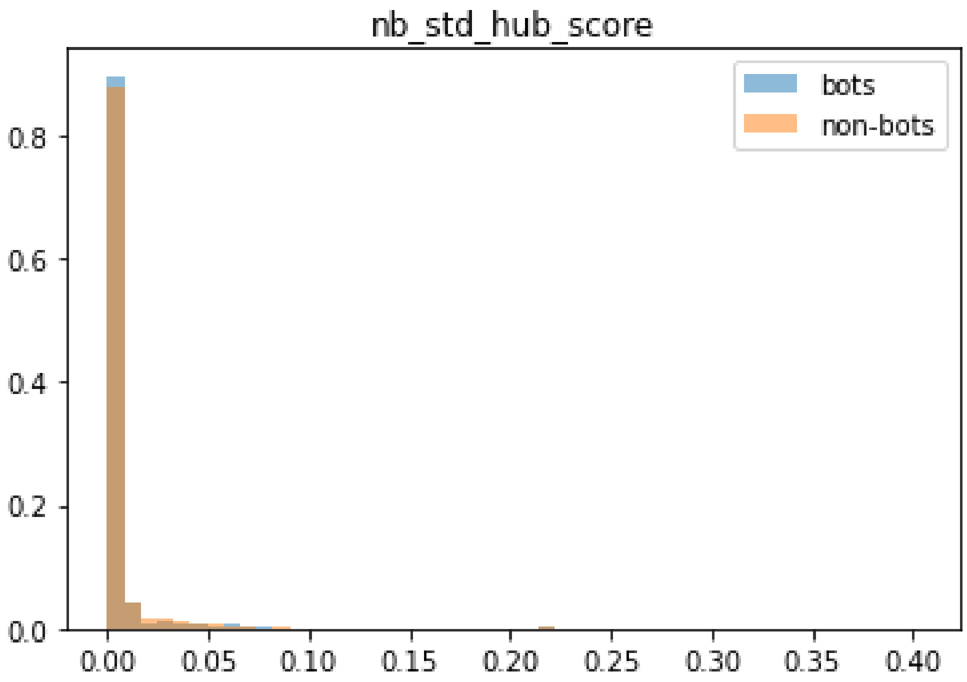
nb_std_hub_score
bots
non-bots
0.0
0.2
0.4
0.6
0.8
0.00
0.05
0.10
0.15
0.20
0.25
0.30
0.35
0.40

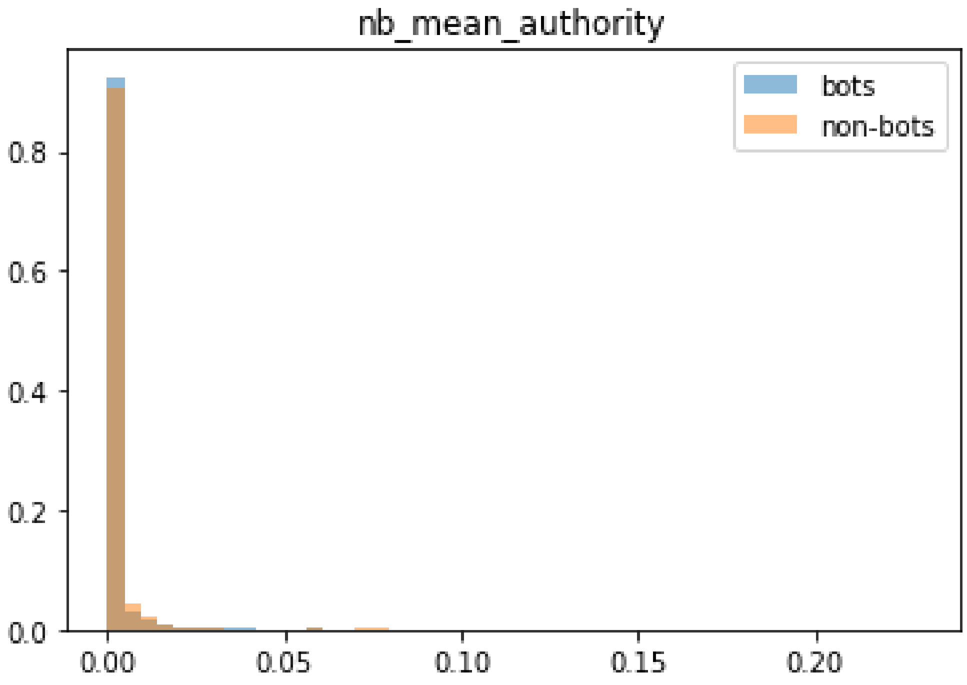
nb_mean_authority
bots
non-bots
0.0
0.2
0.4
0.6
0.8
0.00
0.05
0.10
0.15
0.20

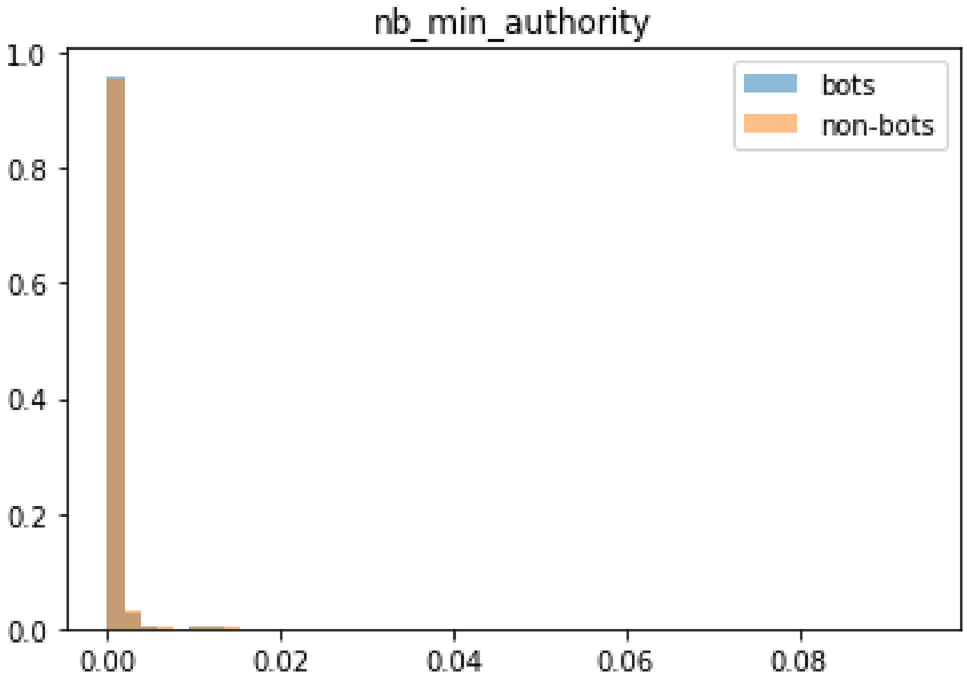
nb_min_authority
1.0
0.8
0.6
0.4
0.2
0.0
0.00
0.02
0.04
0.06
0.08
bots
non-bots

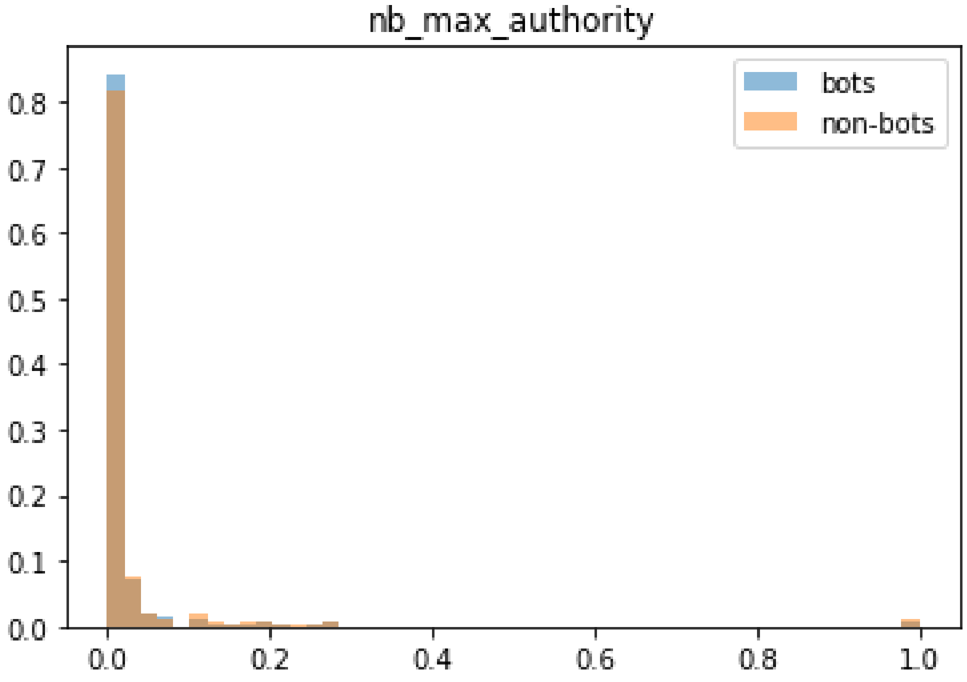
nb_max_authority
0.8
0.7
0.6
0.5
0.4
0.3
0.2
0.1
0.0
0.0
0.2
0.4
0.6
0.8
1.0
bots
non-bots

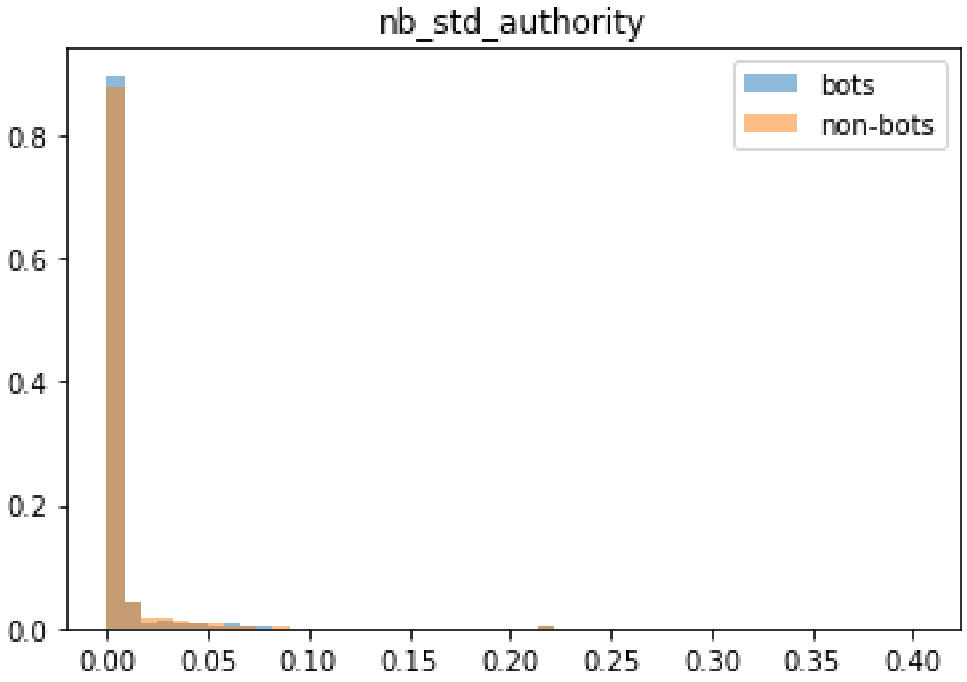
nb_std_authority
bots
non-bots
0.0
0.2
0.4
0.6
0.8
0.00
0.05
0.10
0.15
0.20
0.25
0.30
0.35
0.40

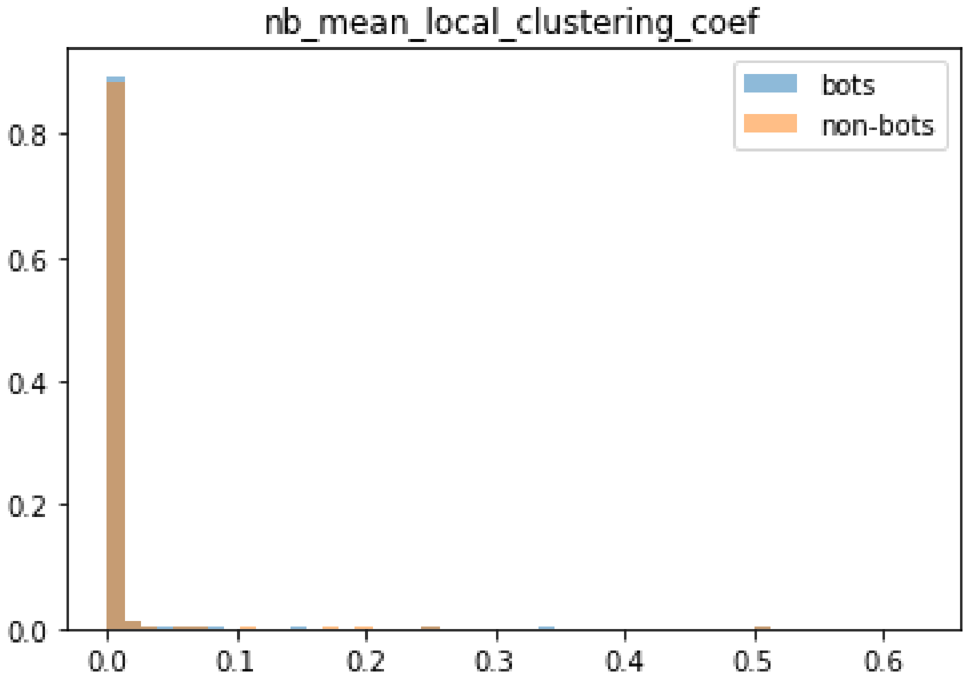
nb_mean_local_clustering_coef
bots
non-bots
0.0
0.2
0.4
0.6
0.8
0.0
0.1
0.2
0.3
0.4
0.5
0.6

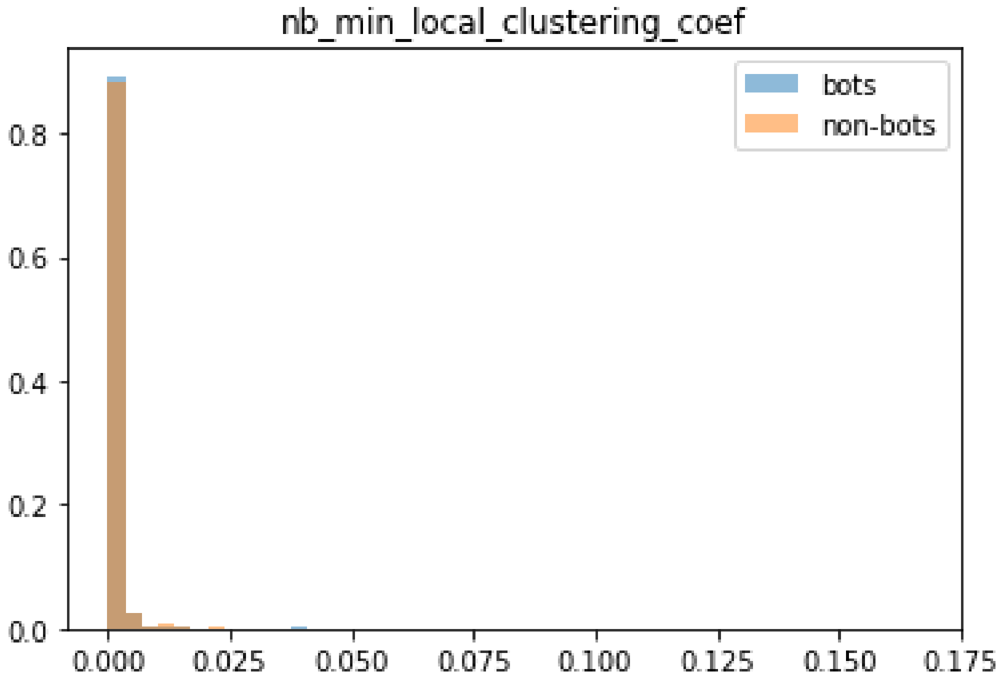
nb_min_local_clustering_coef
bots
non-bots
0.0
0.2
0.4
0.6
0.8
0.000
0.025
0.050
0.075
0.100
0.125
0.150
0.175

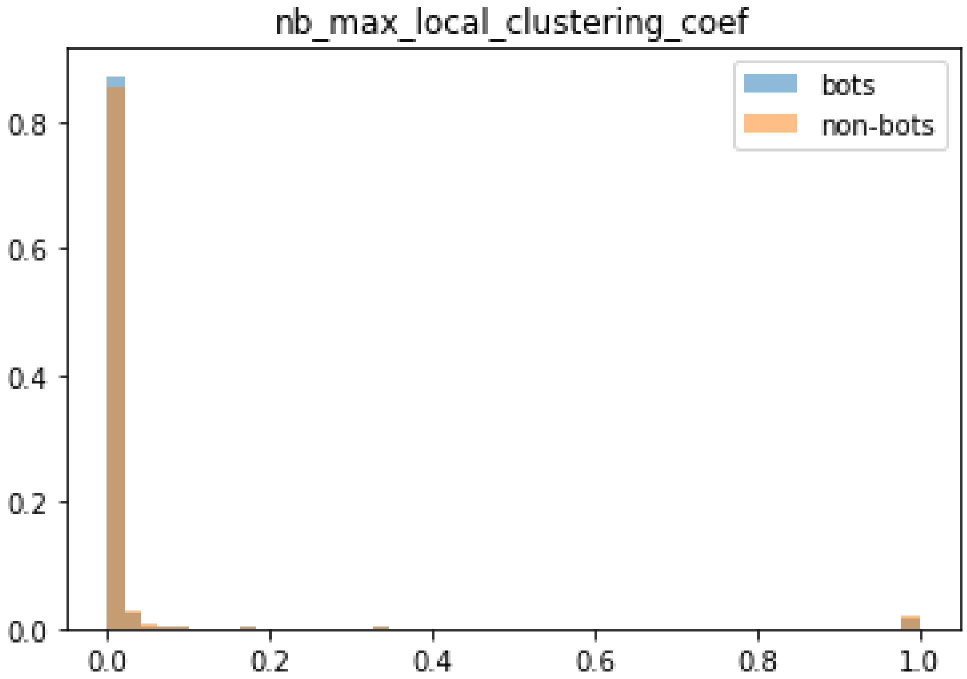
nb_max_local_clustering_coef
bots
non-bots
0.0
0.2
0.4
0.6
0.8
0.0
0.2
0.4
0.6
0.8
1.0

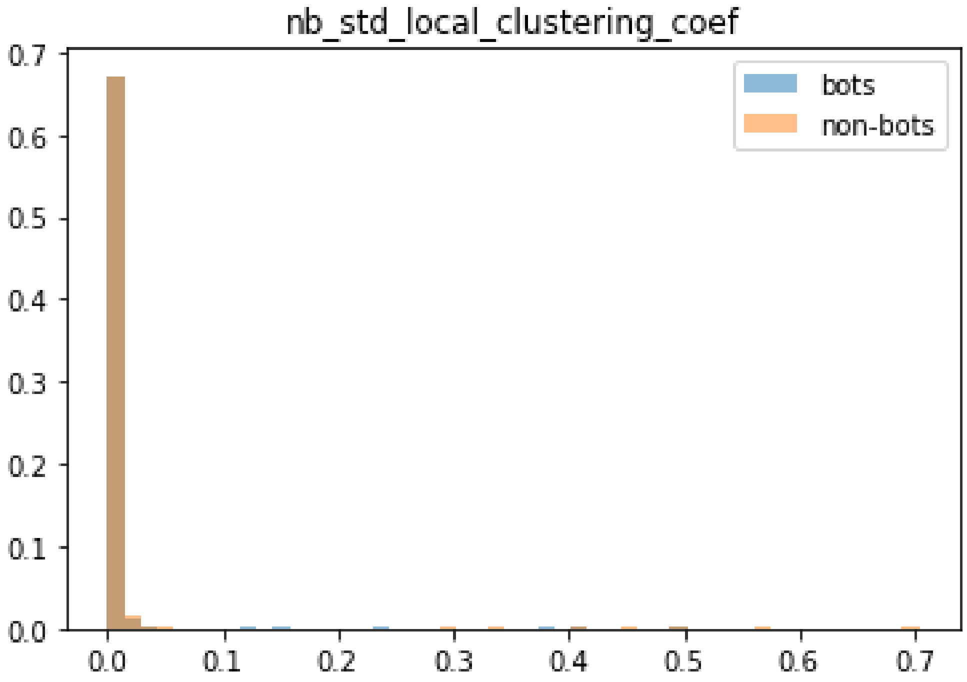
nb_std_local_clustering_coef
bots
non-bots
0.0
0.1
0.2
0.3
0.4
0.5
0.6
0.7

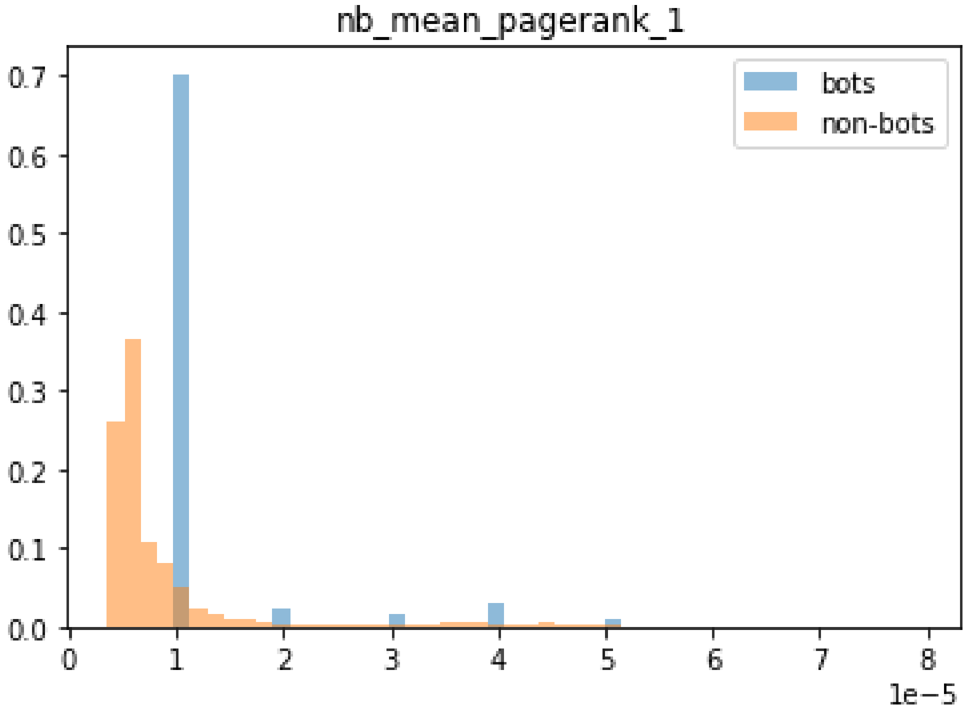
nb_mean_pagerank_1
bots
non-bots
0.0
0.1
0.2
0.3
0.4
0.5
0.6
0.7
0
1
2
3
4
5
6
7
8
1e−5

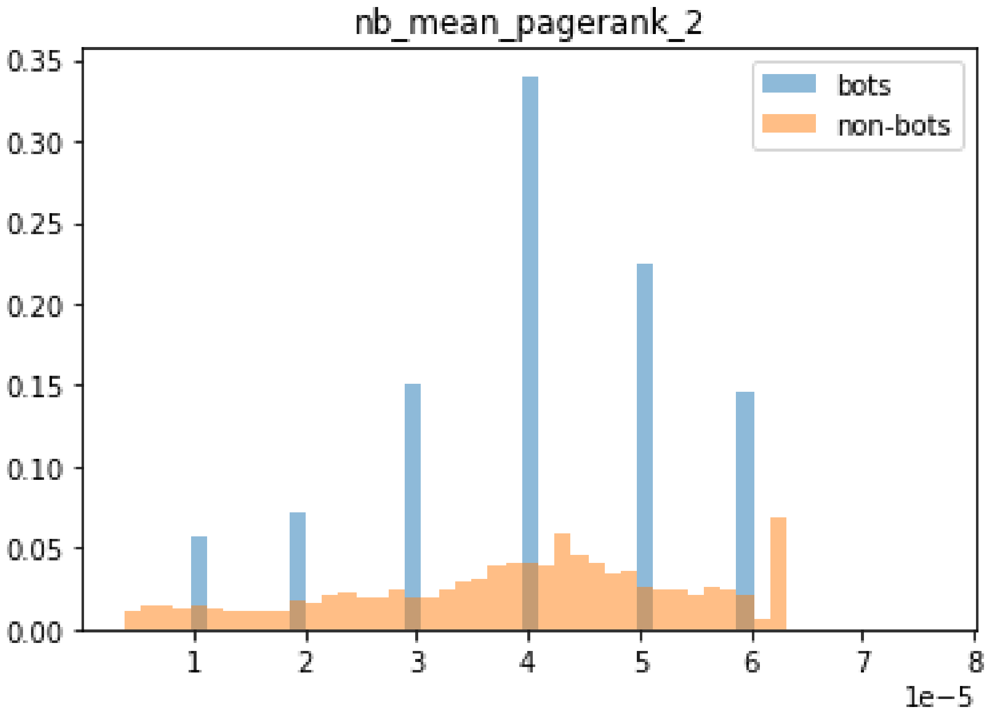
nb_mean_pagerank_2
bots
non-bots
0.35
0.30
0.25
0.20
0.15
0.10
0.05
0.00
1
2
3
4
5
6
7
8
1e−5

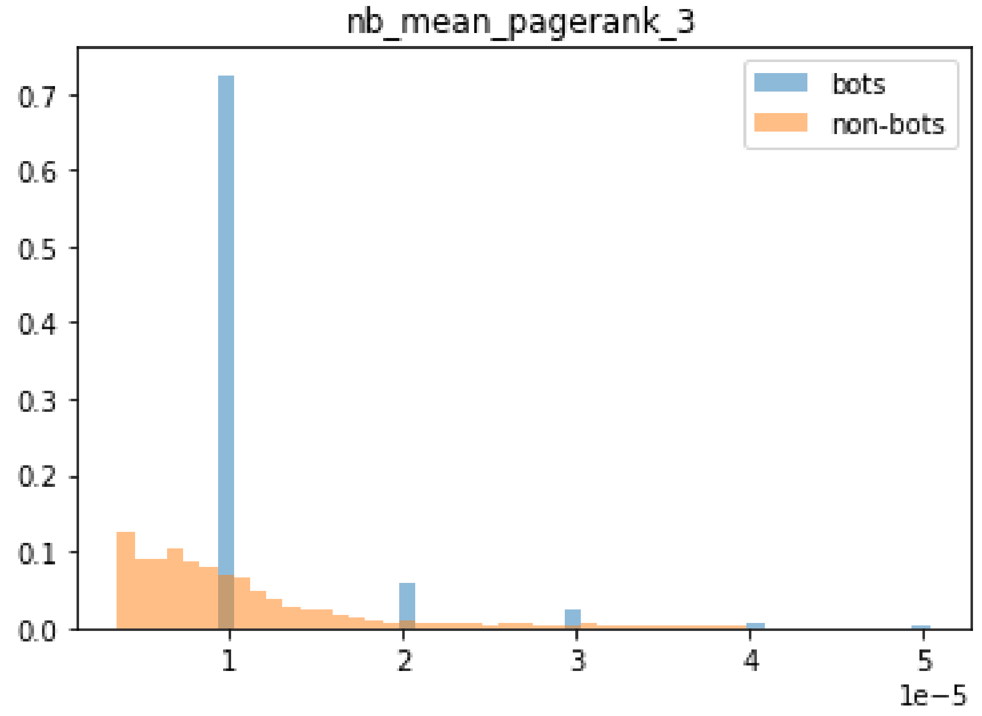
nb_mean_pagerank_3
bots
non-bots
0.7
0.6
0.5
0.4
0.3
0.2
0.1
0.0
1
2
3
4
5
1e−5

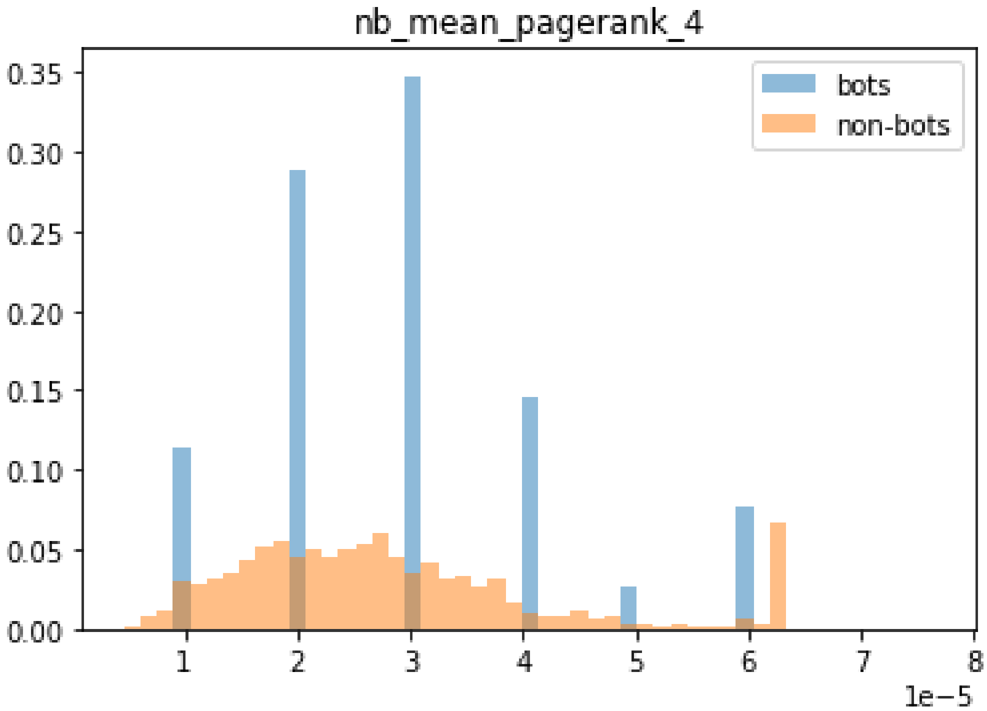
nb_mean_pagerank_4
bots
non-bots
0.00
0.05
0.10
0.15
0.20
0.25
0.30
0.35
1
2
3
4
5
6
7
8
1e−5

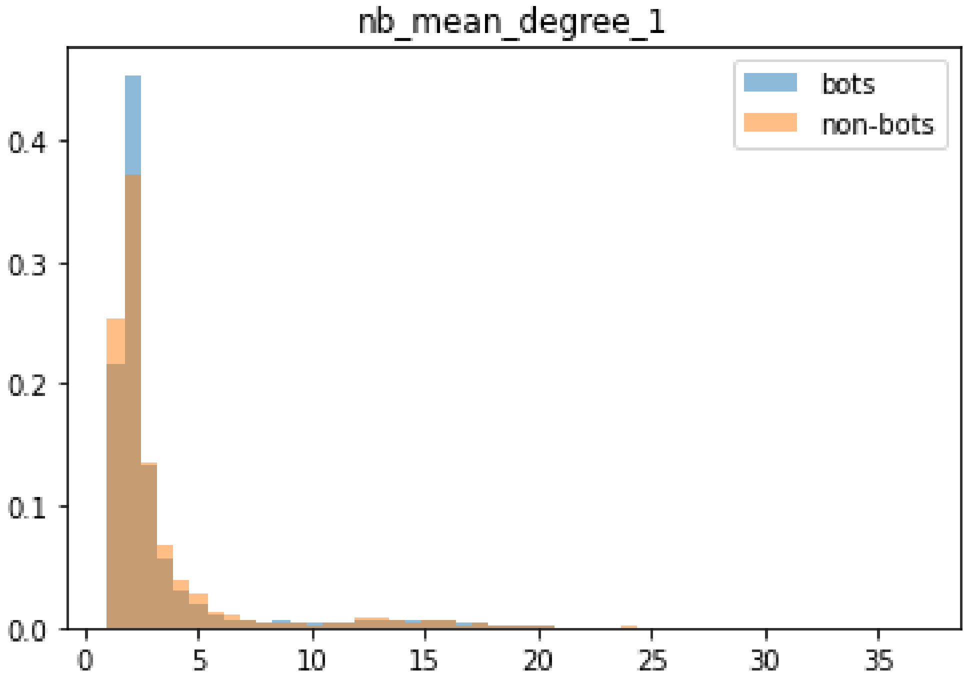
nb_mean_degree_1
bots
non-bots
0.0
0.1
0.2
0.3
0.4
0
5
10
15
20
25
30
35

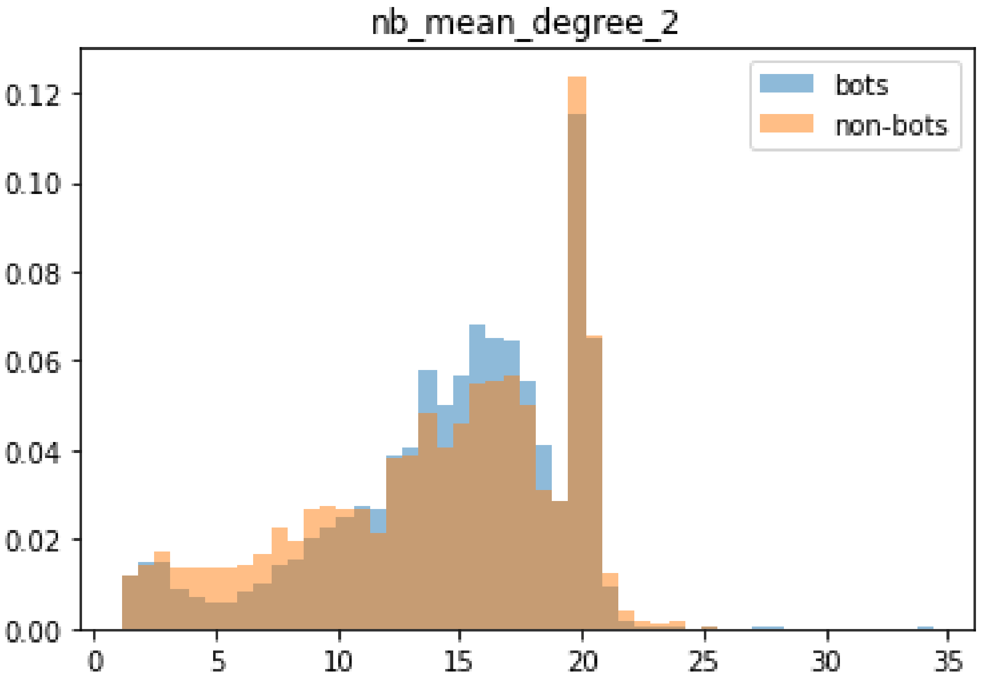
nb_mean_degree_2
bots
non-bots
0.12
0.10
0.08
0.06
0.04
0.02
0.00
0
5
10
15
20
25
30
35

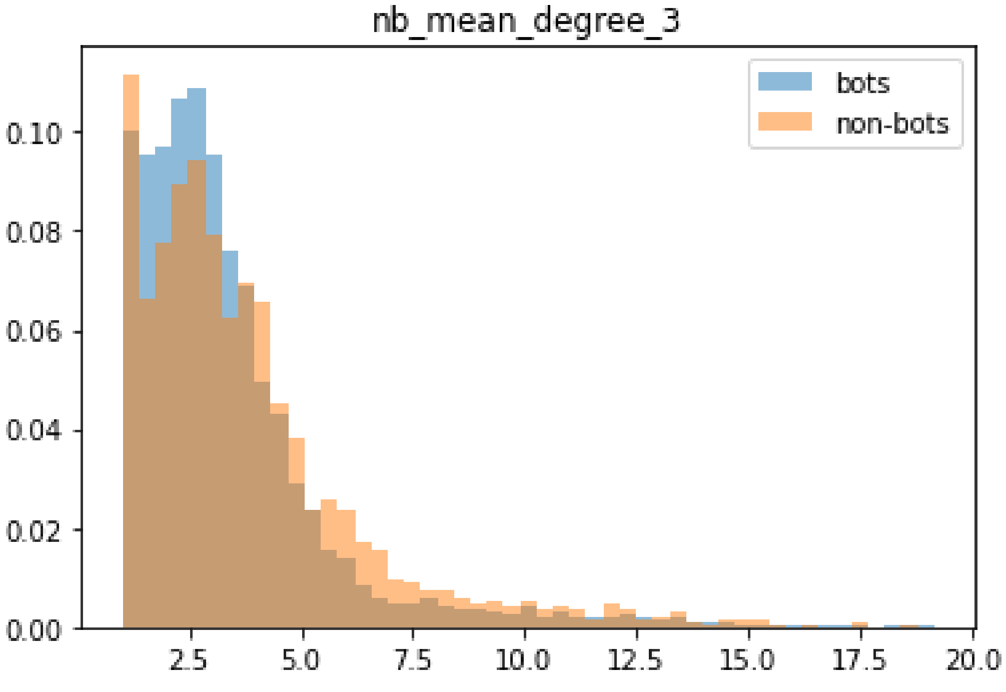
nb_mean_degree_3
bots
non-bots
0.10
0.08
0.06
0.04
0.02
0.00
2.5
5.0
7.5
10.0
12.5
15.0
17.5
20.0

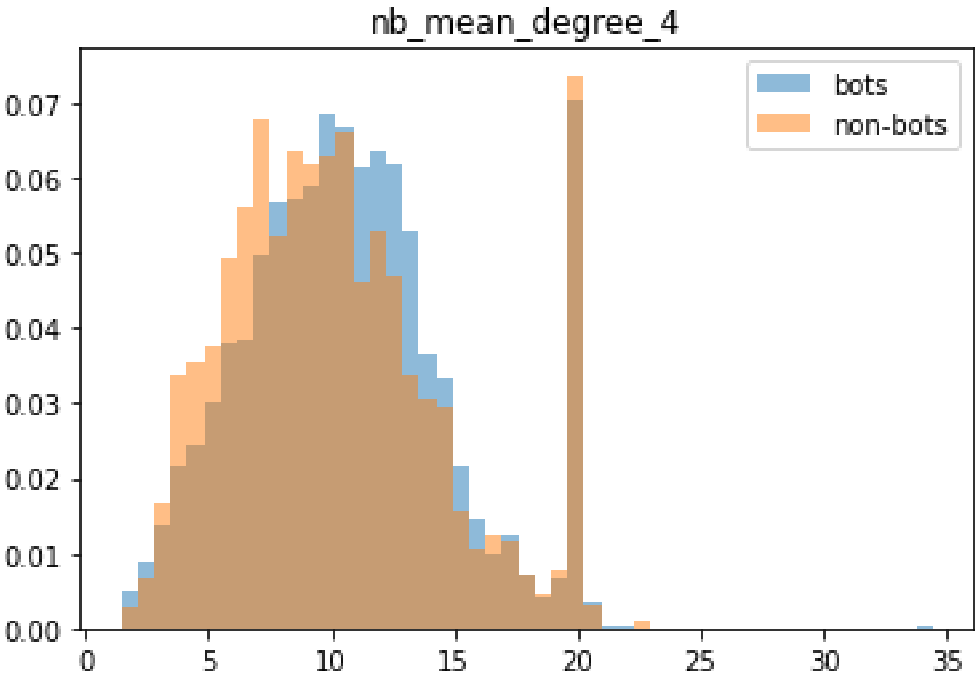
nb_mean_degree_4
bots
non-bots
0.07
0.06
0.05
0.04
0.03
0.02
0.01
0.00
0
5
10
15
20
25
30
35

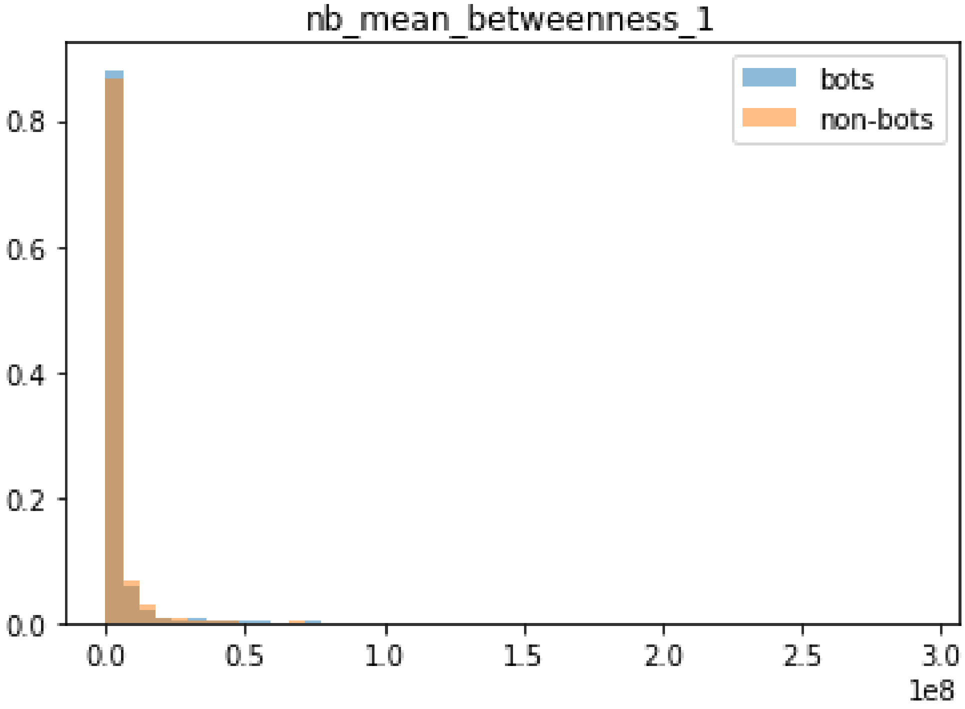
nb_mean_betweenness_1
bots
non-bots
0.8
0.6
0.4
0.2
0.0
0.0
0.5
1.0
1.5
2.0
2.5
3.0
1e8

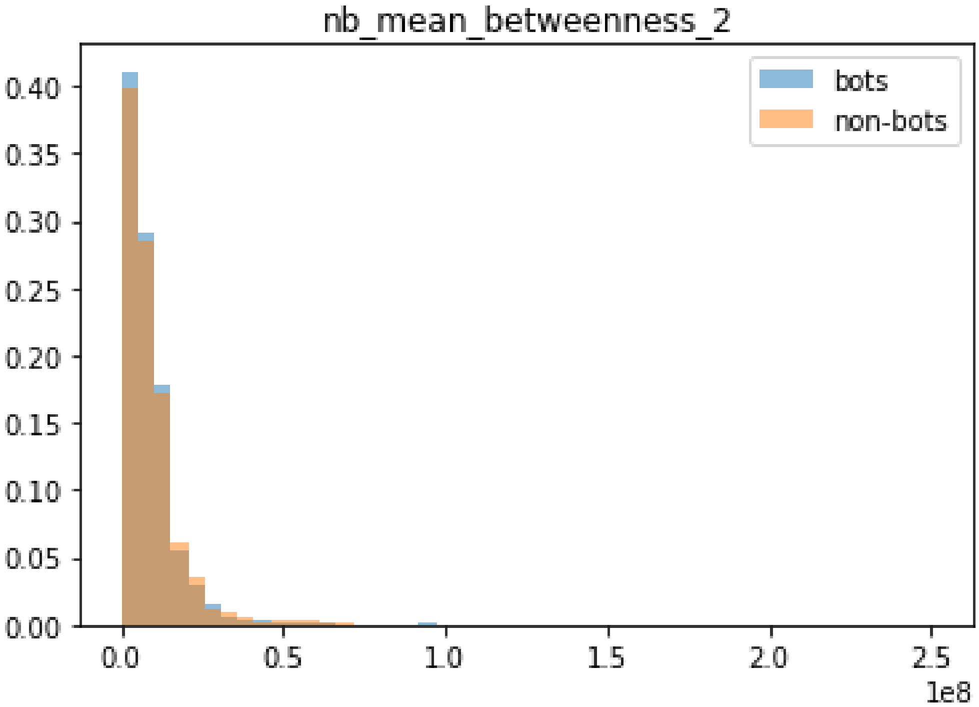
nb_mean_betweenness_2
bots
non-bots
0.40
0.35
0.30
0.25
0.20
0.15
0.10
0.05
0.00
0.0
0.5
1.0
1.5
2.0
2.5
1e8

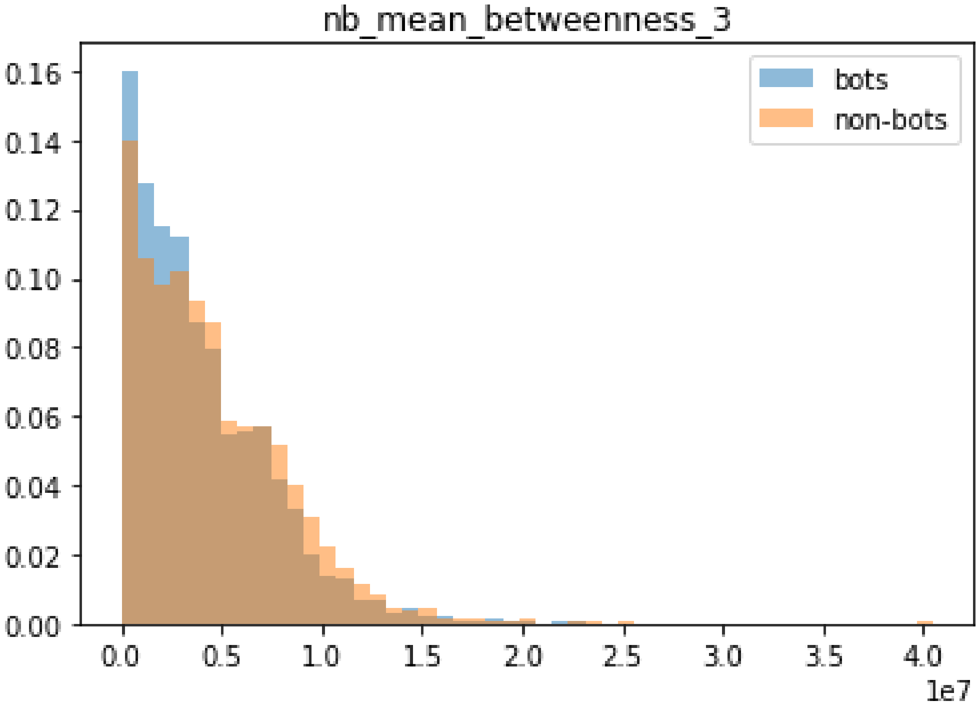
nb_mean_betweenness_3
bots
non-bots
0.16
0.14
0.12
0.10
0.08
0.06
0.04
0.02
0.00
0.0
0.5
1.0
1.5
2.0
2.5
3.0
3.5
4.0
1e7

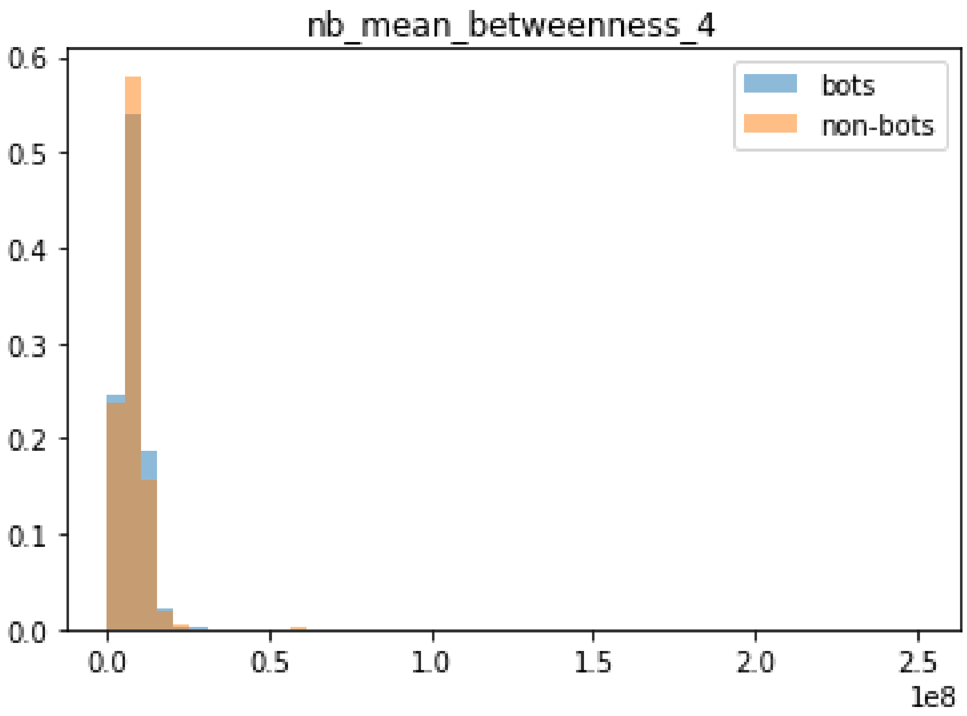
nb_mean_betweenness_4
bots
non-bots
0.0
0.1
0.2
0.3
0.4
0.5
0.6
0.0
0.5
1.0
1.5
2.0
2.5
1e8

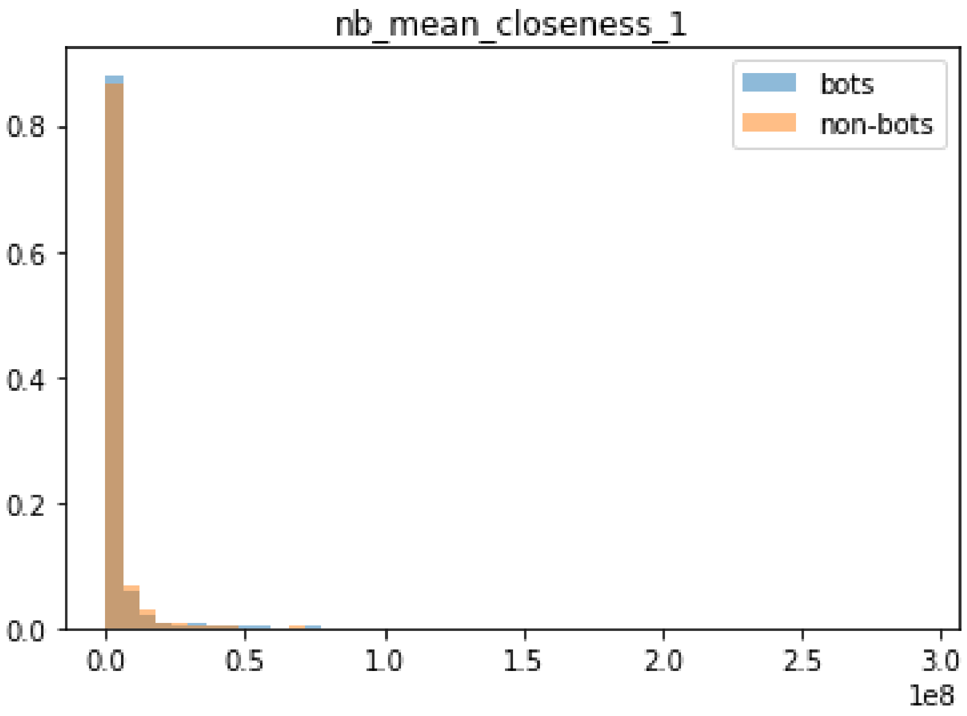
nb_mean_closeness_1
bots
non-bots
0.0
0.2
0.4
0.6
0.8
0.0
0.5
1.0
1.5
2.0
2.5
3.0
1e8

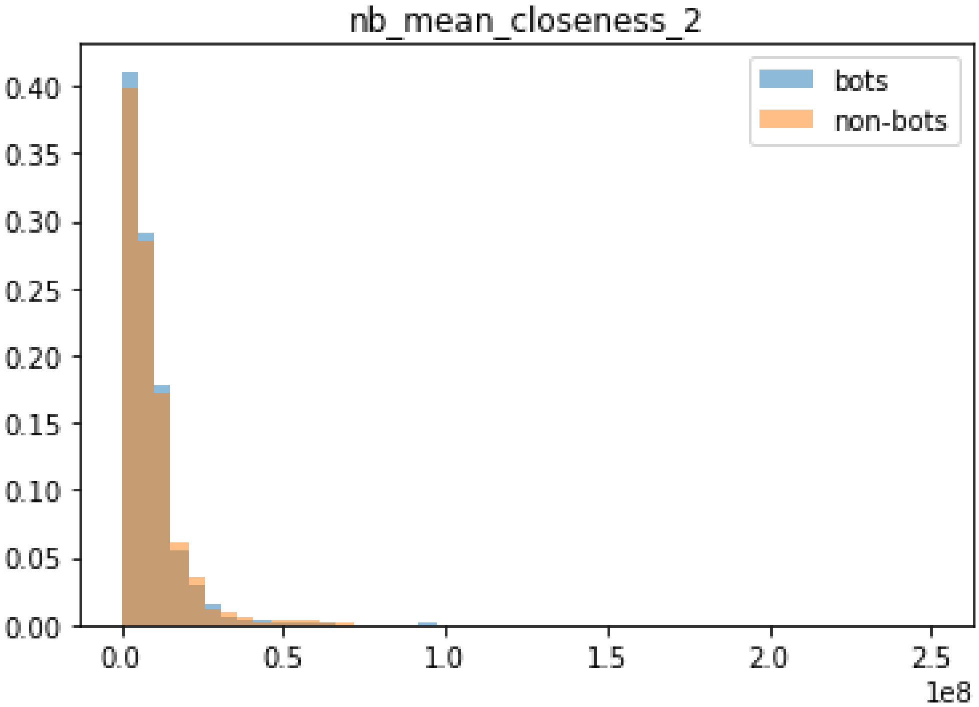
nb_mean_closeness_2
bots
non-bots
0.40
0.35
0.30
0.25
0.20
0.15
0.10
0.05
0.00
0.0
0.5
1.0
1.5
2.0
2.5
1e8

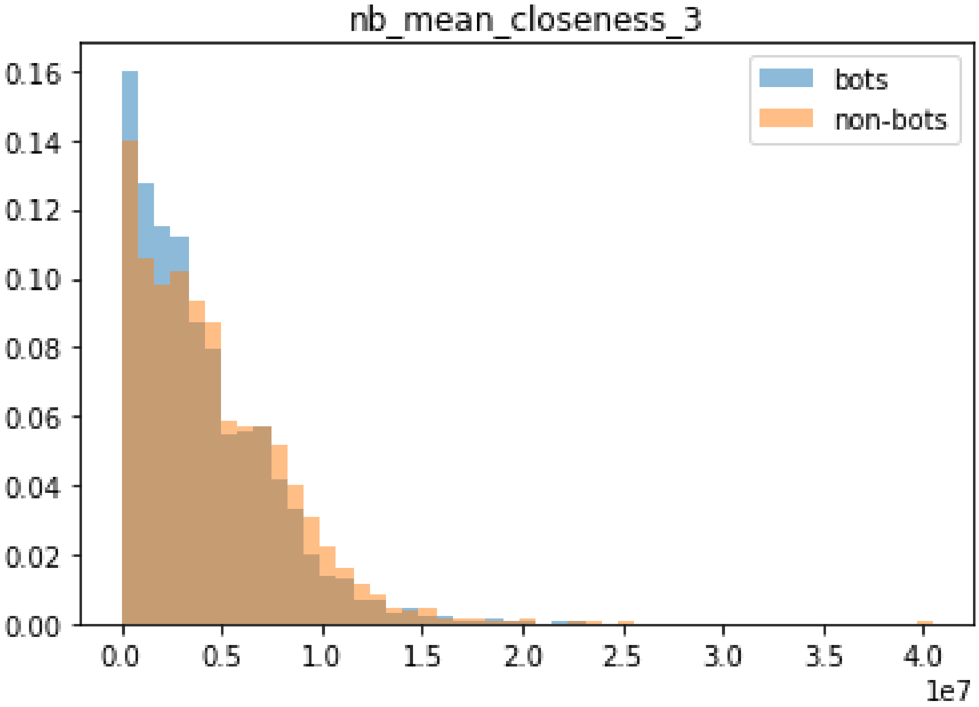
nb_mean_closeness_3
bots
non-bots
0.16
0.14
0.12
0.10
0.08
0.06
0.04
0.02
0.00
0.0
0.5
1.0
1.5
2.0
2.5
3.0
3.5
4.0
1e7

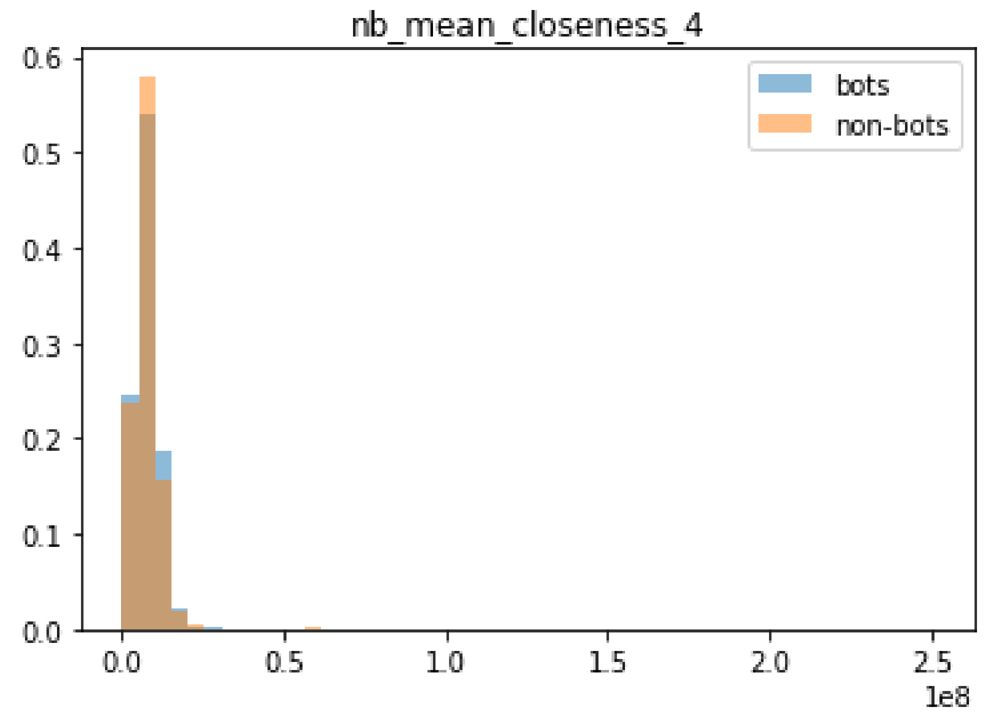
nb_mean_closeness_4
bots
non-bots
0.0
0.1
0.2
0.3
0.4
0.5
0.6
0.0
0.5
1.0
1.5
2.0
2.5
1e8

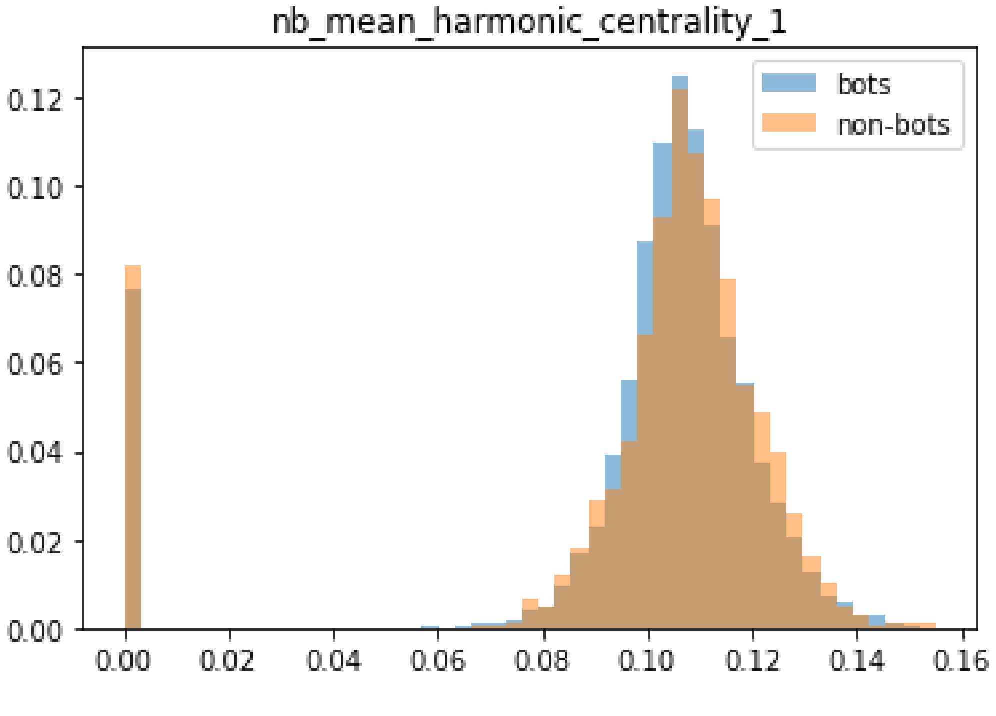
nb_mean_harmonic_centrality_1
bots
non-bots
0.00
0.02
0.04
0.06
0.08
0.10
0.12
0.00
0.02
0.04
0.06
0.08
0.10
0.12
0.14
0.16

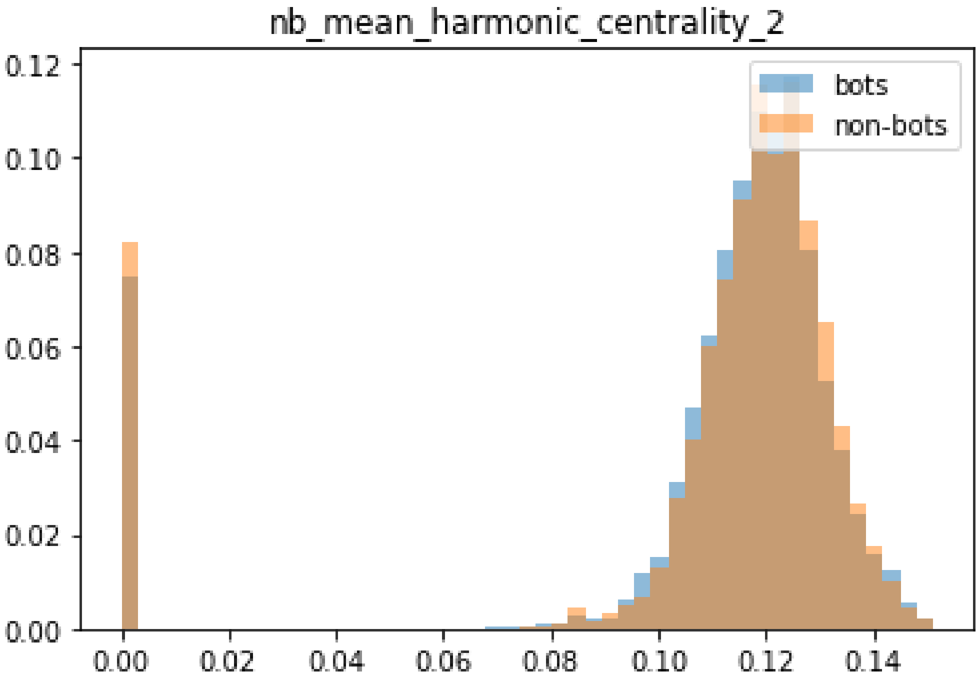
nb_mean_harmonic_centrality_2
bots
non-bots
0.00
0.02
0.04
0.06
0.08
0.10
0.12
0.14

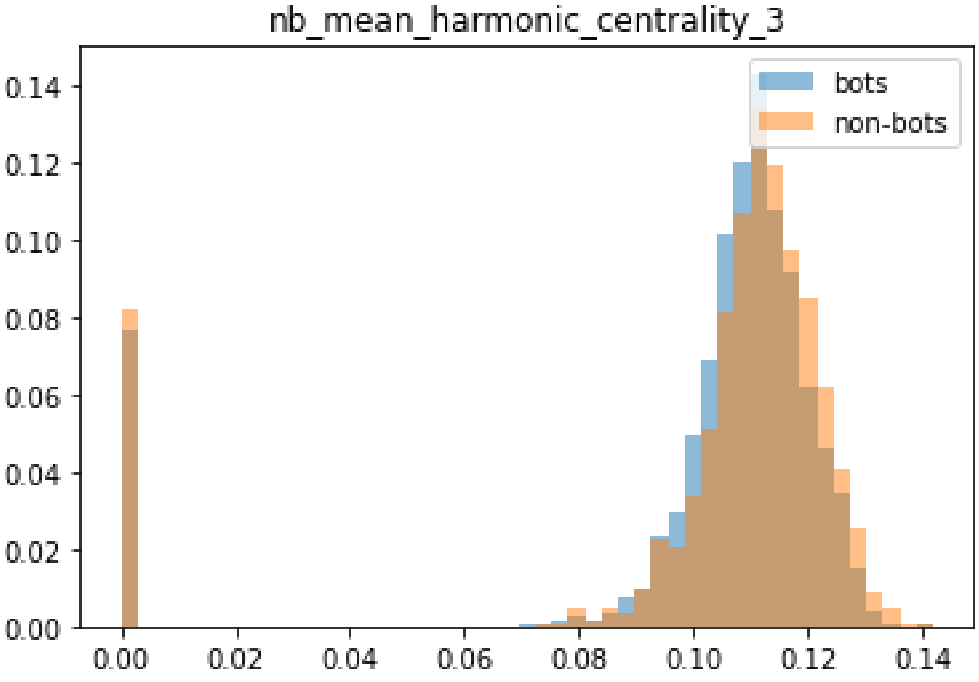
nb_mean_harmonic_centrality_3
bots
non-bots
0.00
0.02
0.04
0.06
0.08
0.10
0.12
0.14

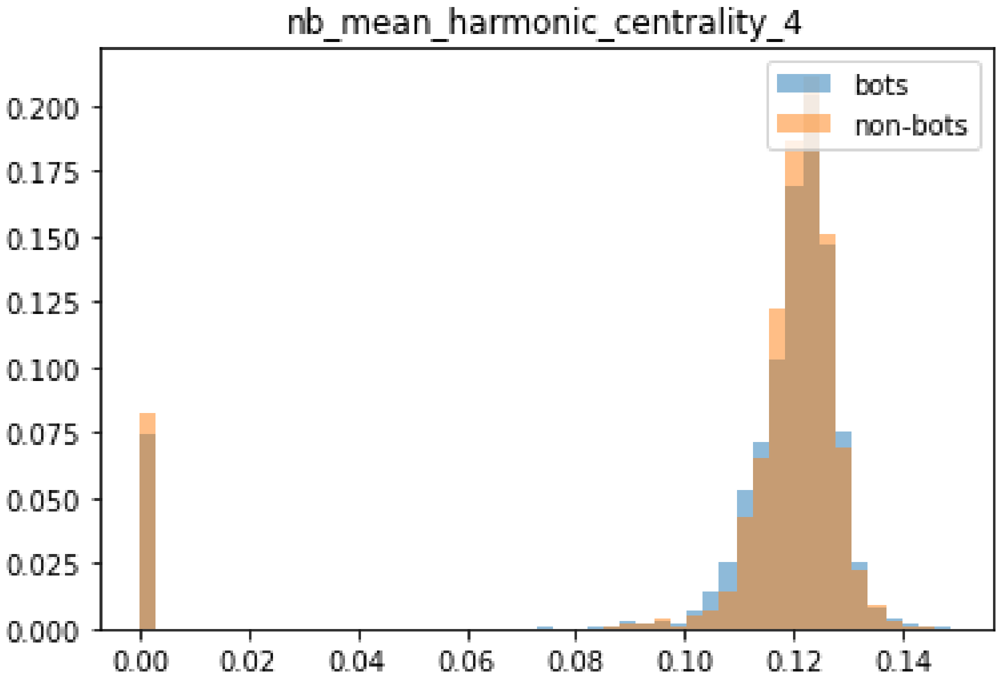
nb_mean_harmonic_centrality_4
bots
non-bots
0.200
0.175
0.150
0.125
0.100
0.075
0.050
0.025
0.000
0.00
0.02
0.04
0.06
0.08
0.10
0.12
0.14

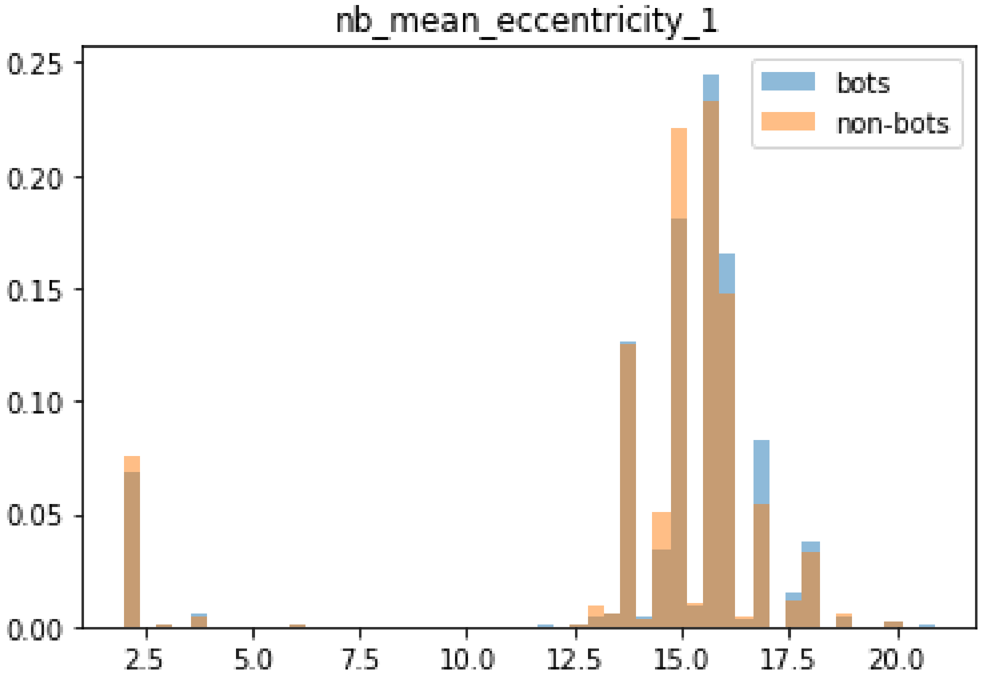
nb_mean_eccentricity_1
bots
non-bots
0.25
0.20
0.15
0.10
0.05
0.00
2.5
5.0
7.5
10.0
12.5
15.0
17.5
20.0

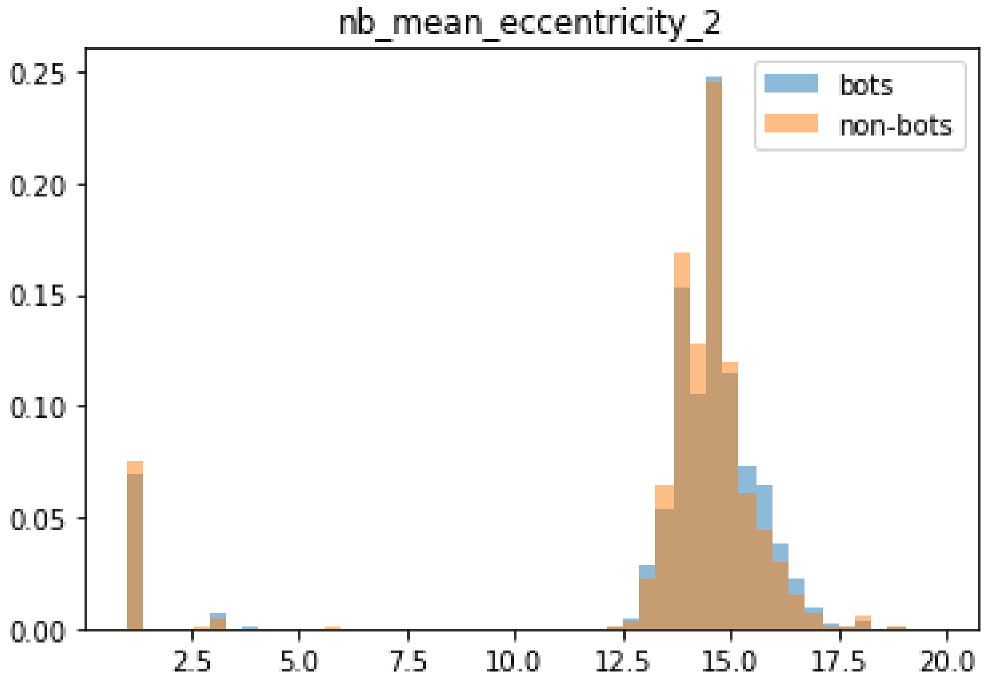
nb_mean_eccentricity_2
bots
non-bots
0.25
0.20
0.15
0.10
0.05
0.00
2.5
5.0
7.5
10.0
12.5
15.0
17.5
20.0

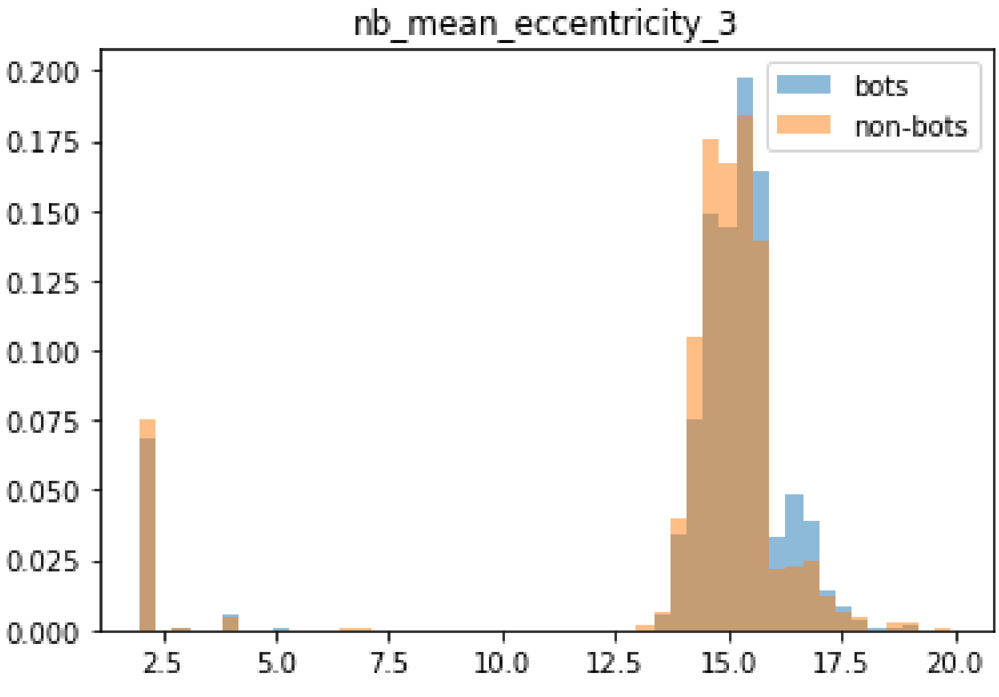
nb_mean_eccentricity_3
bots
non-bots
0.200
0.175
0.150
0.125
0.100
0.075
0.050
0.025
0.000
2.5
5.0
7.5
10.0
12.5
15.0
17.5
20.0

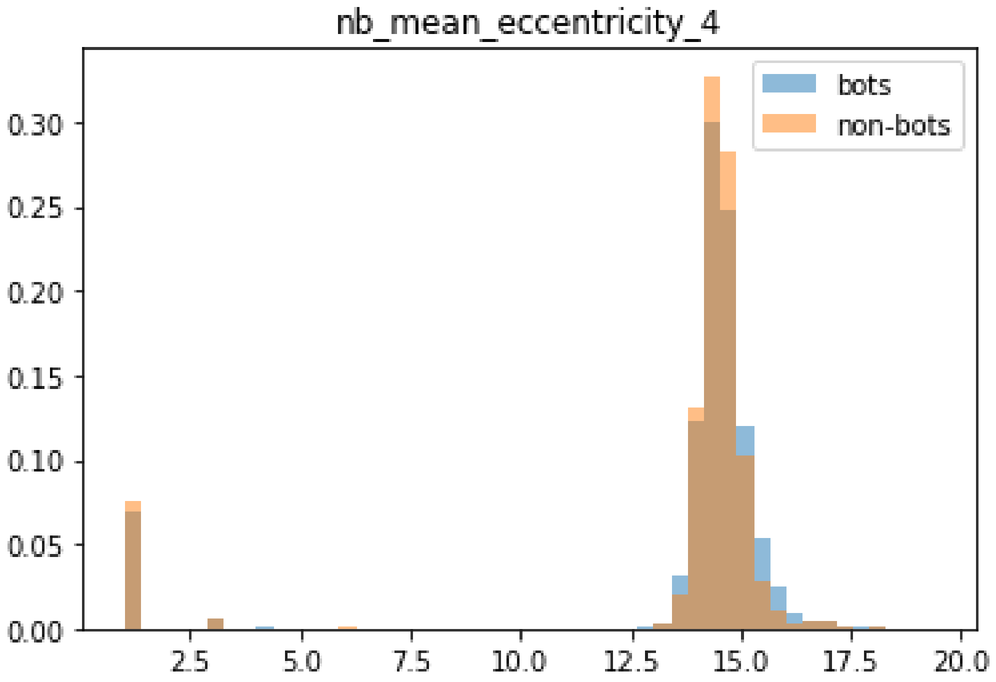
nb_mean_eccentricity_4
bots
non-bots
0.30
0.25
0.20
0.15
0.10
0.05
0.00
2.5
5.0
7.5
10.0
12.5
15.0
17.5
20.0

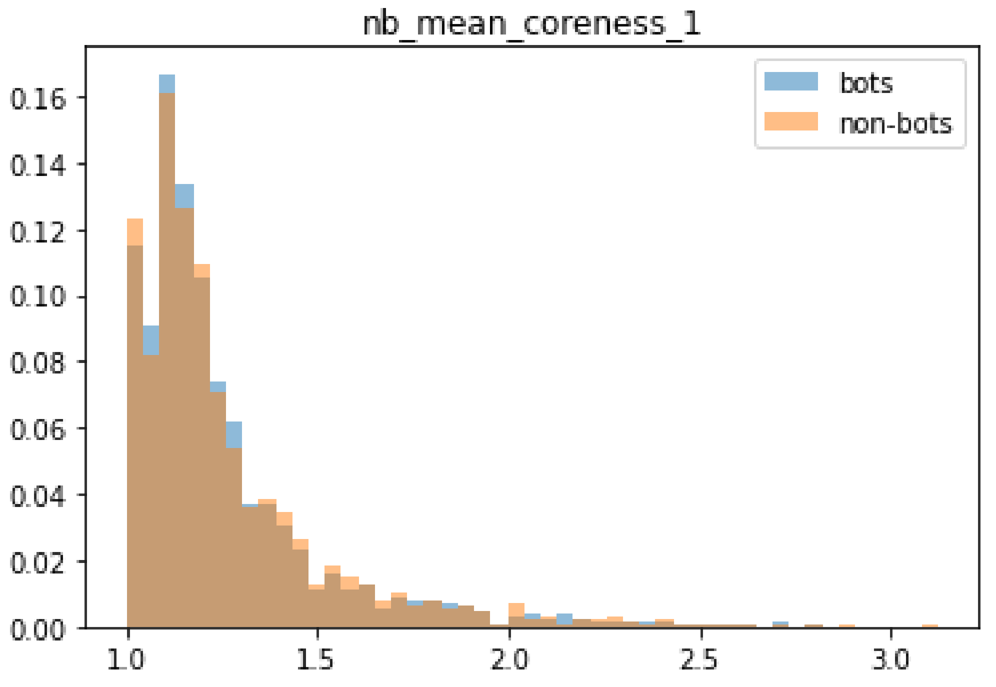
nb_mean_coreness_1
bots
non-bots
0.16
0.14
0.12
0.10
0.08
0.06
0.04
0.02
0.00
1.0
1.5
2.0
2.5
3.0

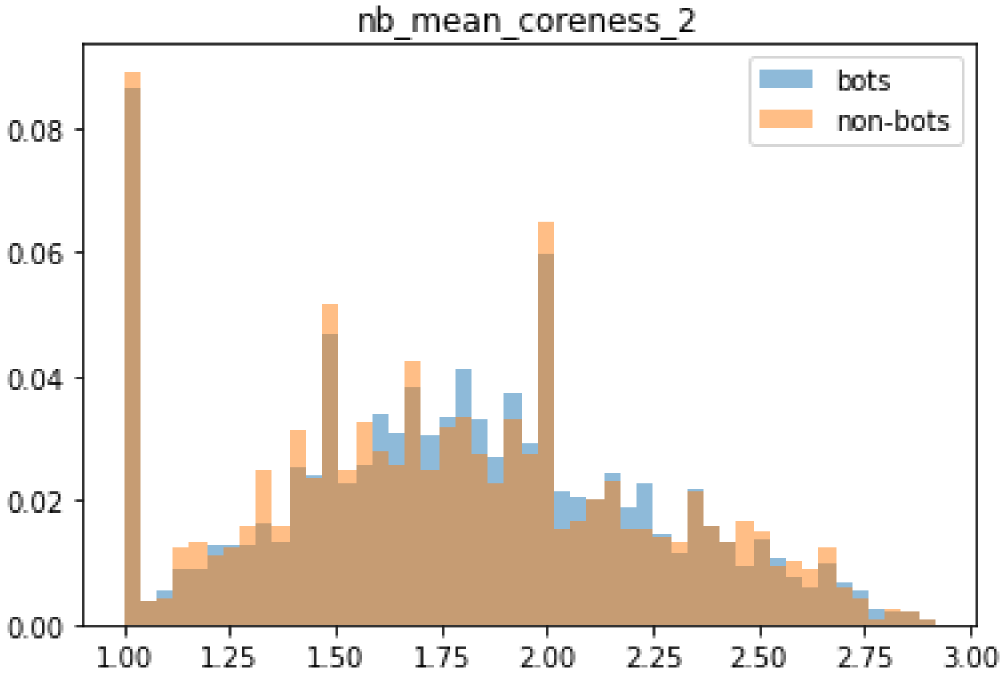
nb_mean_coreness_2
bots
non-bots
0.08
0.06
0.04
0.02
0.00
1.00
1.25
1.50
1.75
2.00
2.25
2.50
2.75
3.00

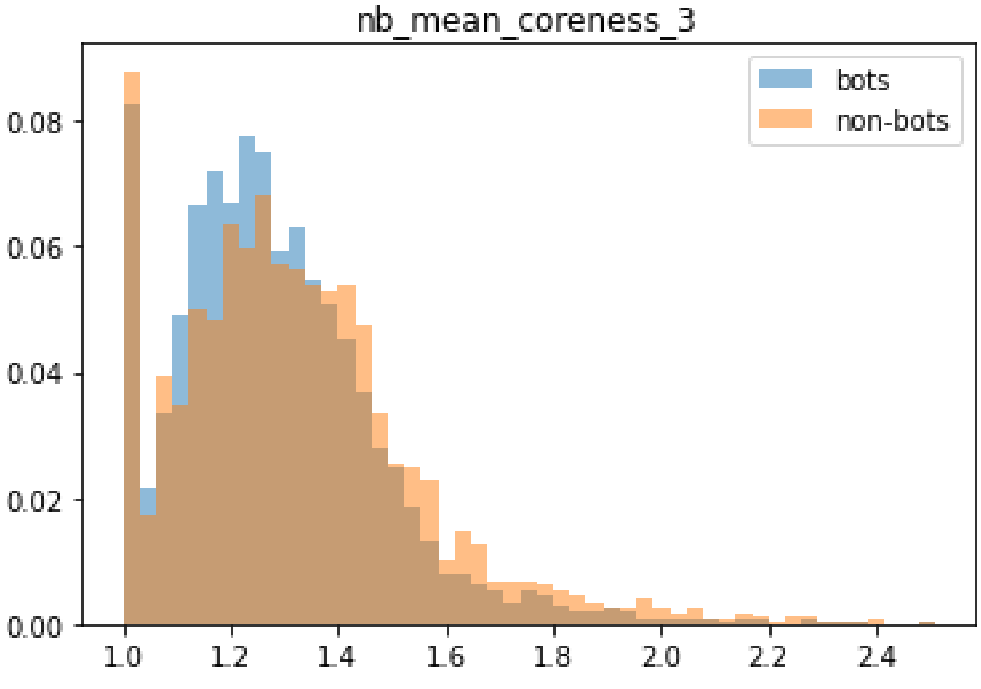
nb_mean_coreness_3
bots
non-bots
0.08
0.06
0.04
0.02
0.00
1.0
1.2
1.4
1.6
1.8
2.0
2.2
2.4

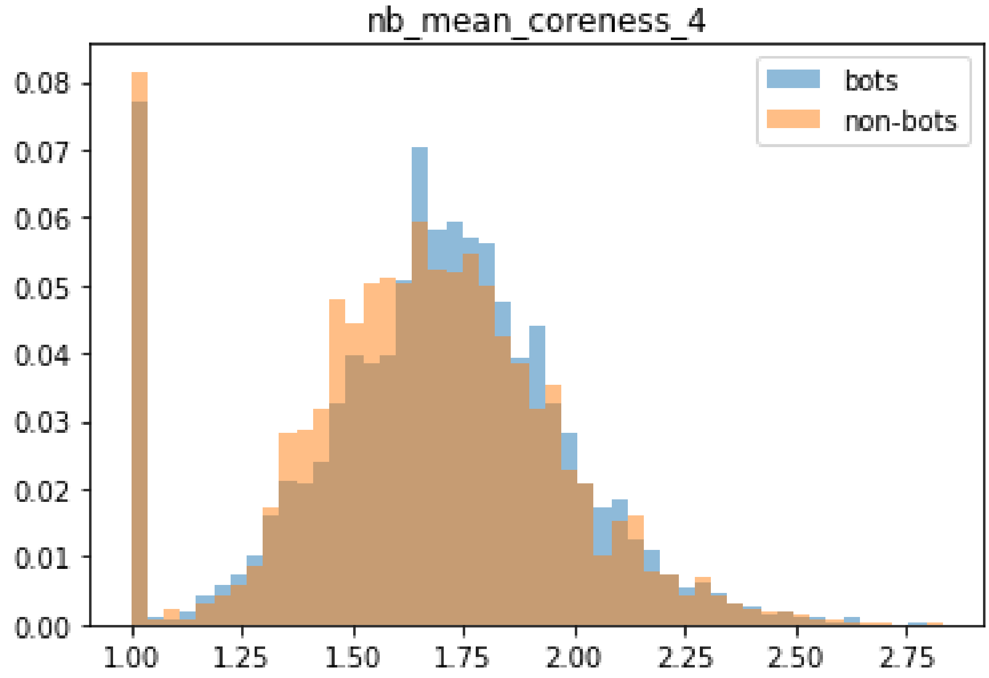
nb_mean_coreness_4
bots
non-bots
0.00
0.01
0.02
0.03
0.04
0.05
0.06
0.07
0.08
1.00
1.25
1.50
1.75
2.00
2.25
2.50
2.75

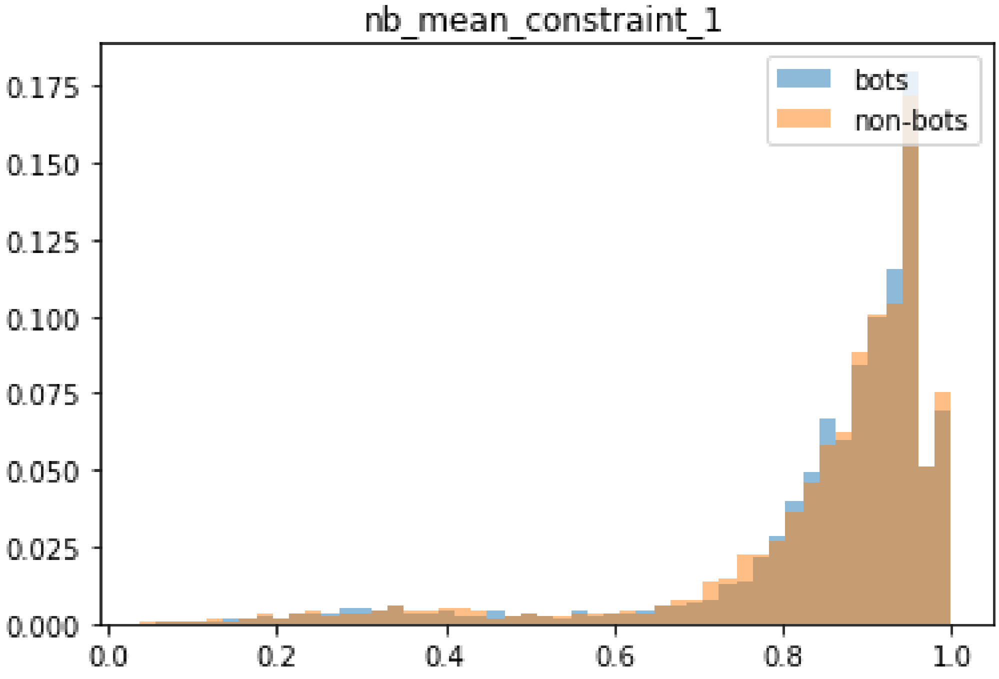
nb_mean_constraint_1
bots
non-bots
0.000
0.025
0.050
0.075
0.100
0.125
0.150
0.175
0.0
0.2
0.4
0.6
0.8
1.0

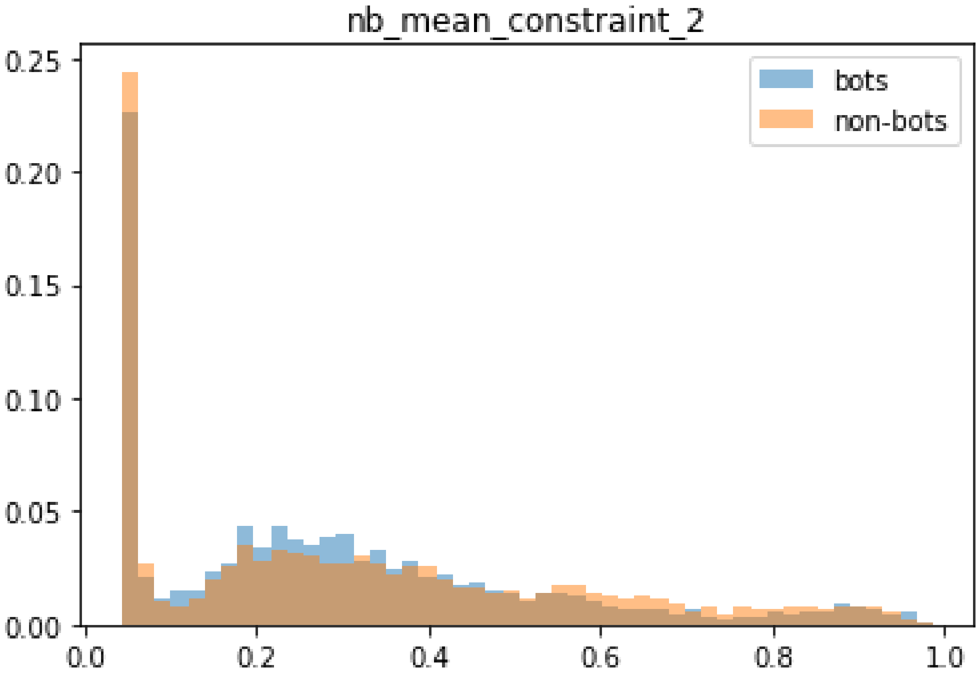
nb_mean_constraint_2
bots
non-bots
0.25
0.20
0.15
0.10
0.05
0.00
0.0
0.2
0.4
0.6
0.8
1.0

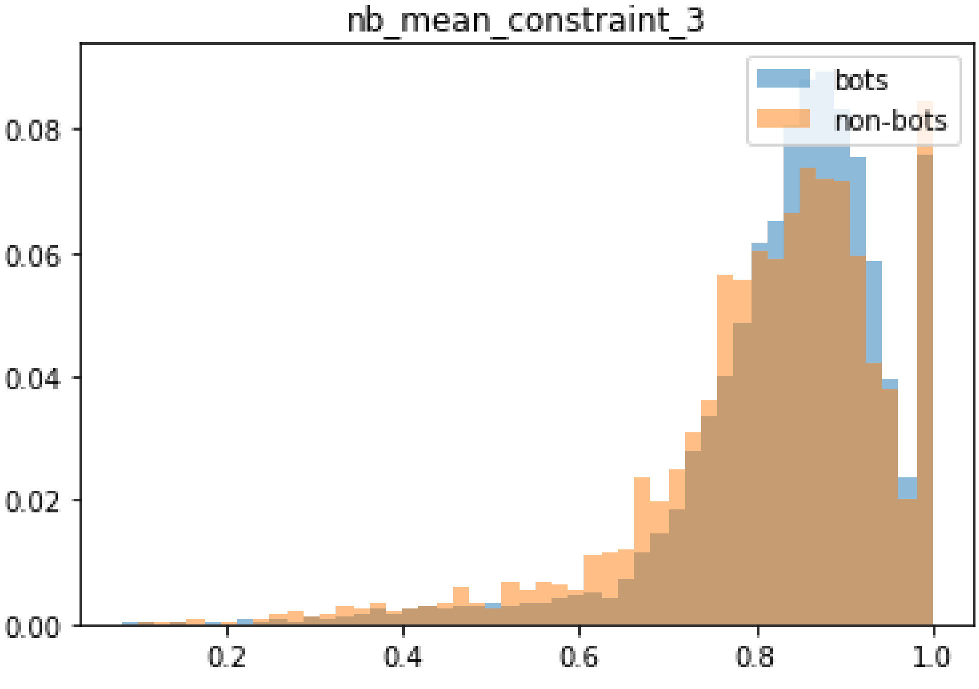
nb_mean_constraint_3
bots
non-bots
0.08
0.06
0.04
0.02
0.00
0.2
0.4
0.6
0.8
1.0

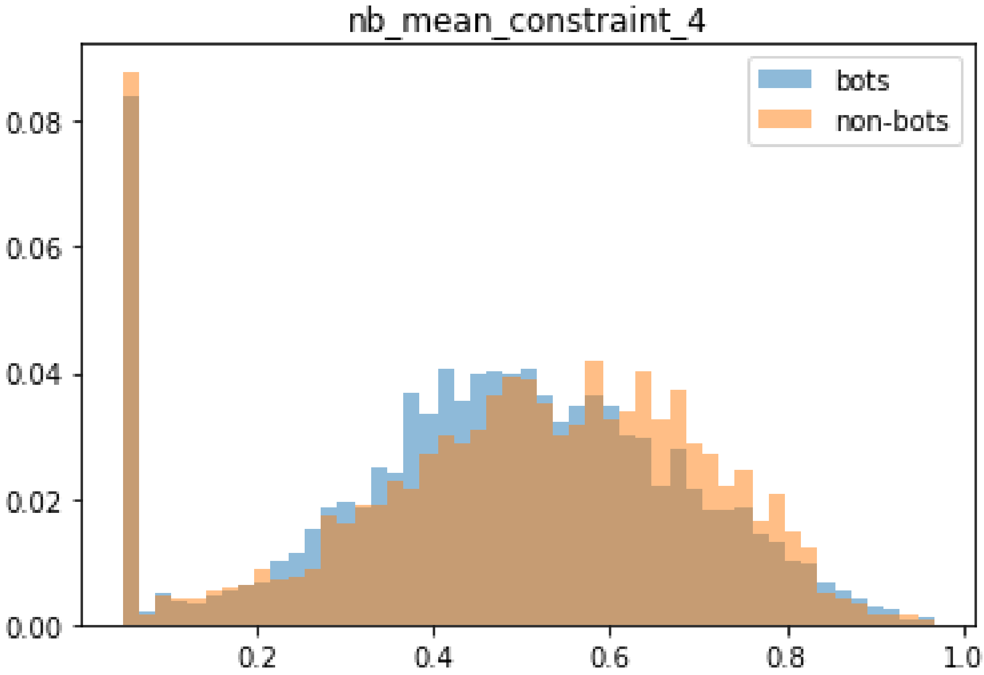
nb_mean_constraint_4
bots
non-bots
0.08
0.06
0.04
0.02
0.00
0.2
0.4
0.6
0.8
1.0

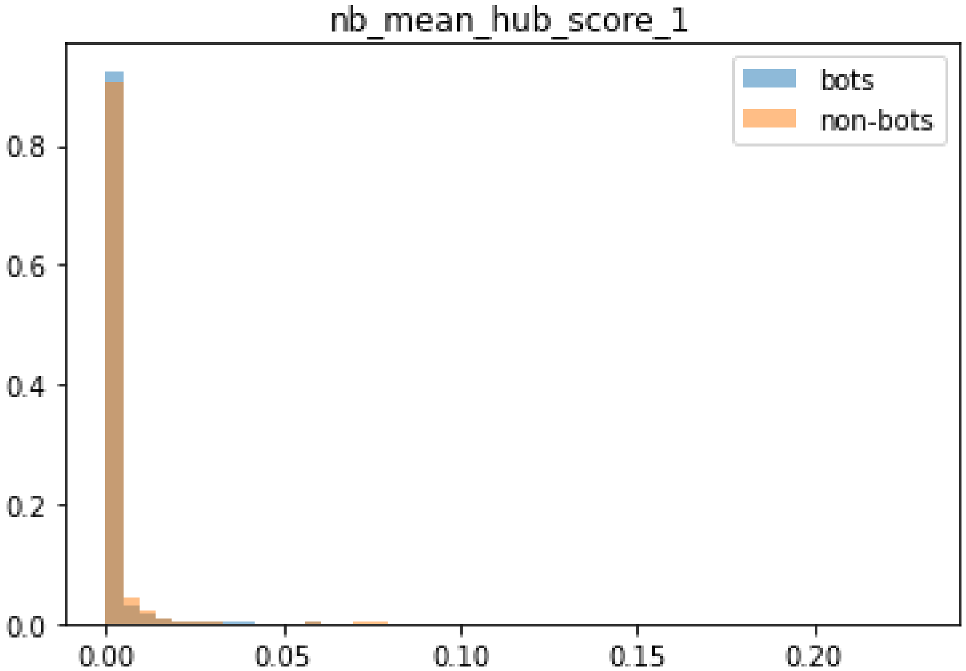
nb_mean_hub_score_1
bots
non-bots
0.8
0.6
0.4
0.2
0.0
0.00
0.05
0.10
0.15
0.20

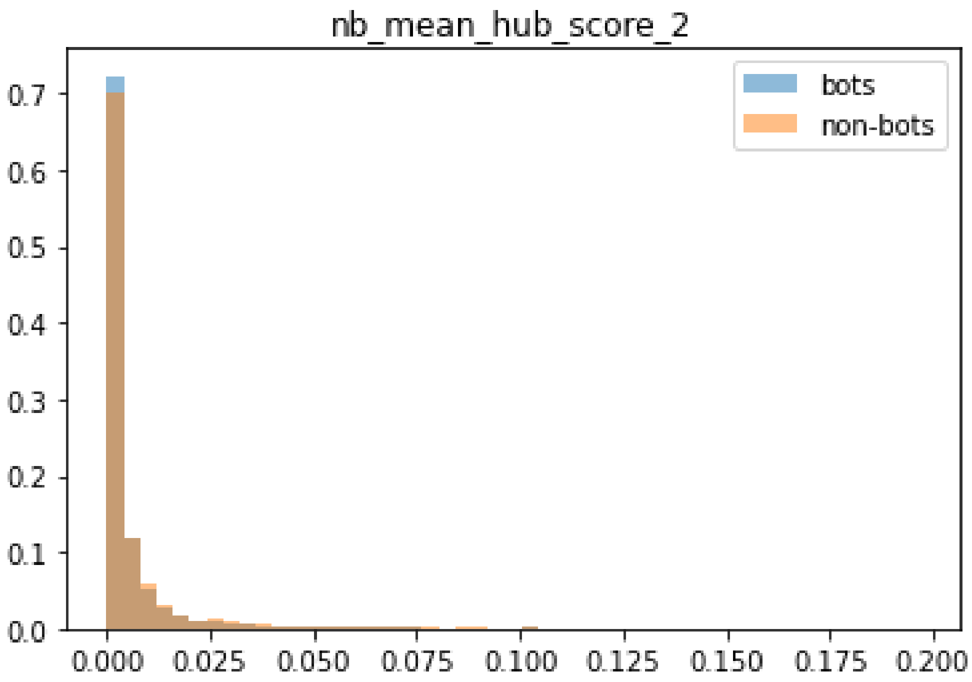
nb_mean_hub_score_2
bots
non-bots
0.0
0.1
0.2
0.3
0.4
0.5
0.6
0.7
0.000
0.025
0.050
0.075
0.100
0.125
0.150
0.175
0.200

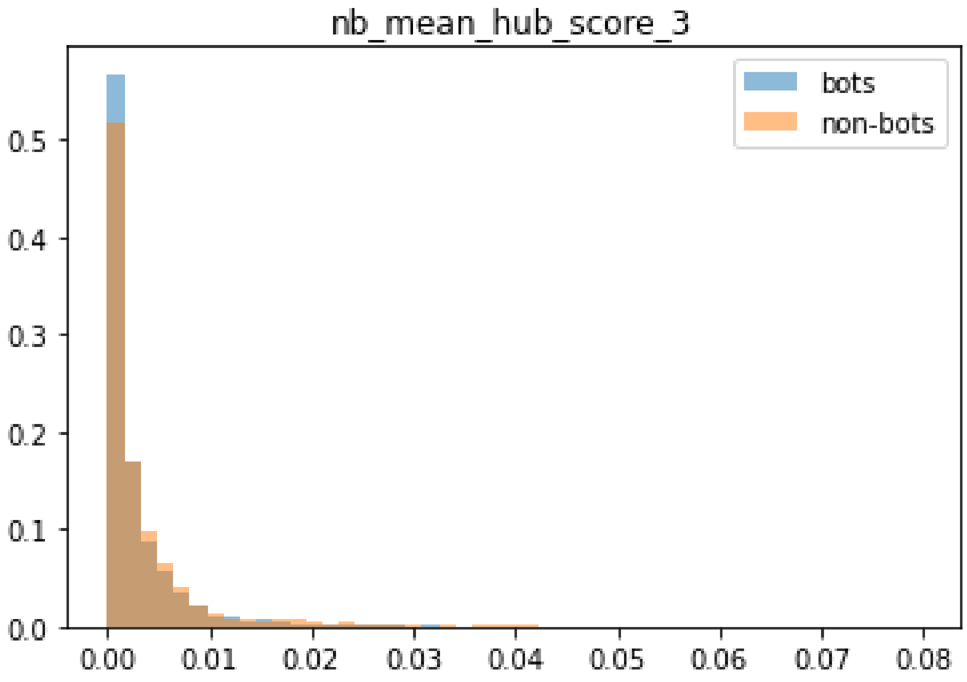
nb_mean_hub_score_3
bots
non-bots
0.0
0.1
0.2
0.3
0.4
0.5
0.00
0.01
0.02
0.03
0.04
0.05
0.06
0.07
0.08

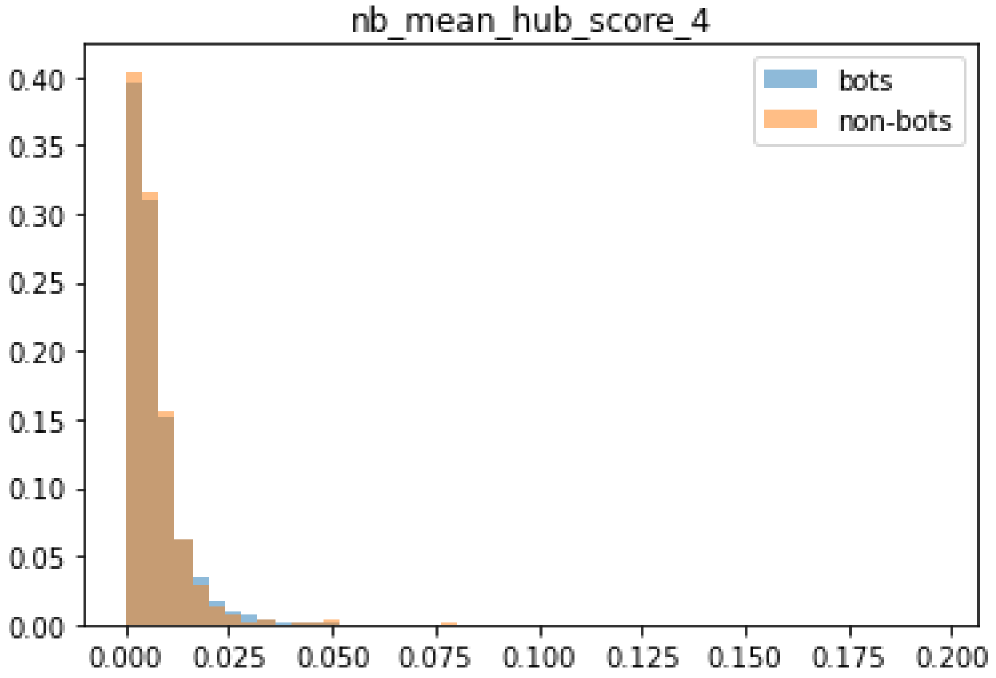
nb_mean_hub_score_4
bots
non-bots
0.40
0.35
0.30
0.25
0.20
0.15
0.10
0.05
0.00
0.000
0.025
0.050
0.075
0.100
0.125
0.150
0.175
0.200

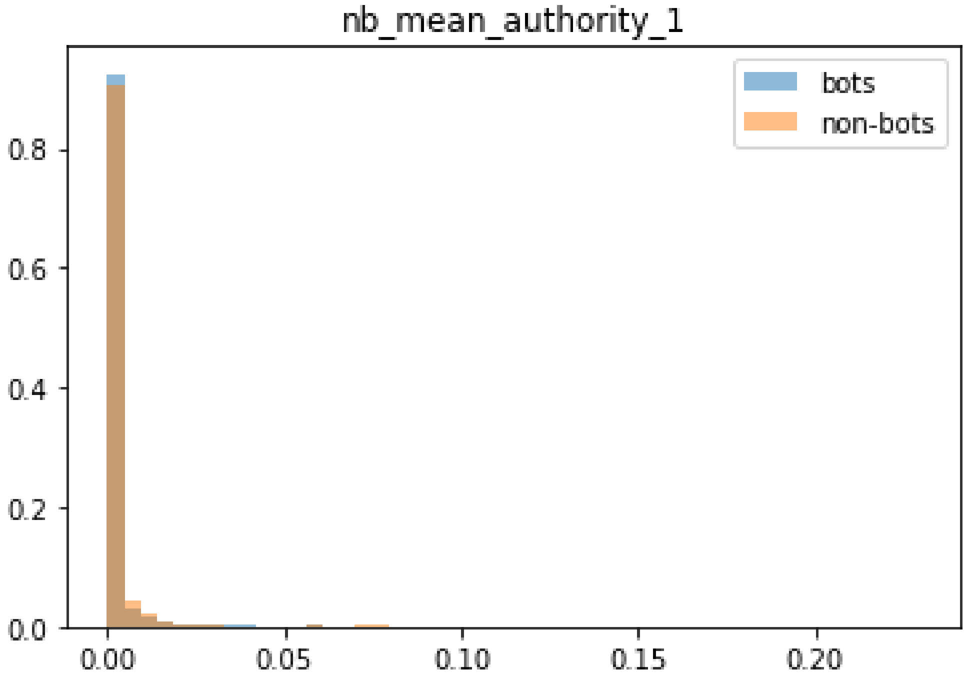
nb_mean_authority_1
bots
non-bots
0.8
0.6
0.4
0.2
0.0
0.00
0.05
0.10
0.15
0.20

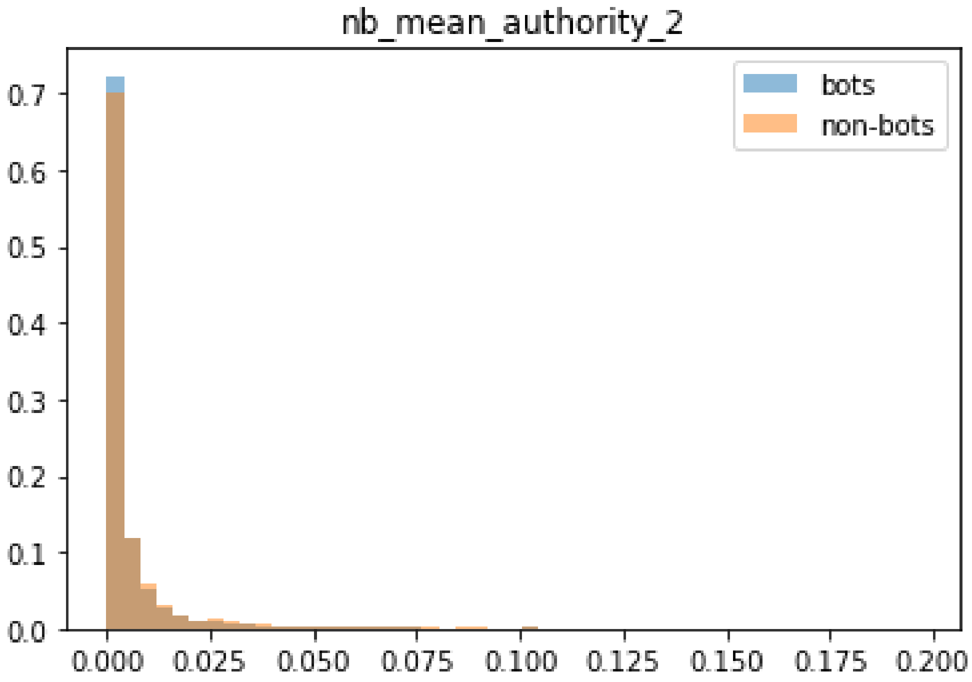
nb_mean_authority_2
bots
non-bots
0.0
0.1
0.2
0.3
0.4
0.5
0.6
0.7
0.000
0.025
0.050
0.075
0.100
0.125
0.150
0.175
0.200

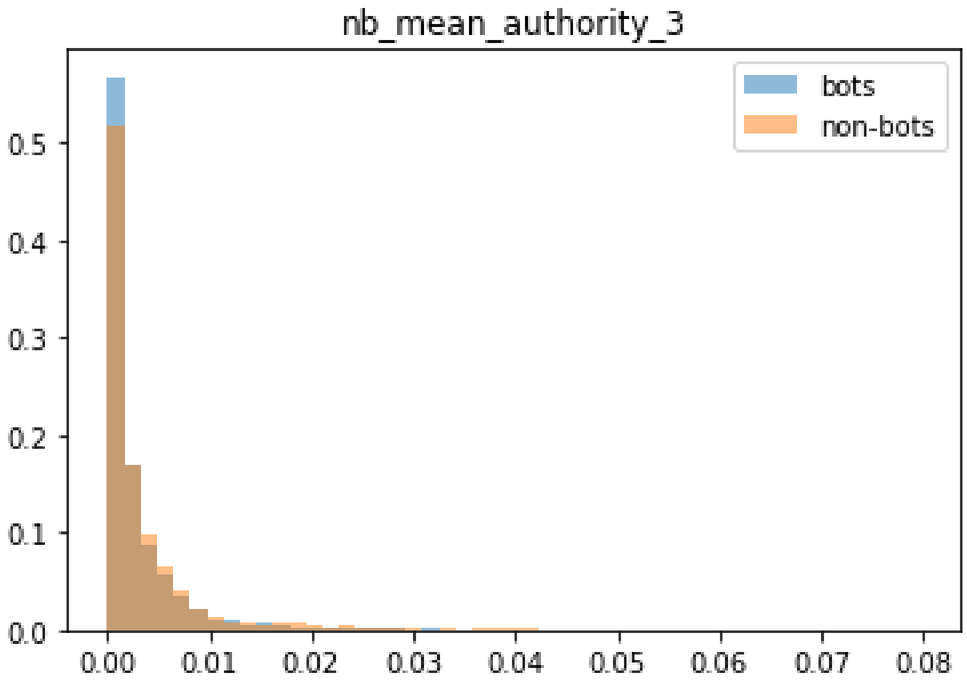
nb_mean_authority_3
bots
non-bots
0.0
0.1
0.2
0.3
0.4
0.5
0.00
0.01
0.02
0.03
0.04
0.05
0.06
0.07
0.08

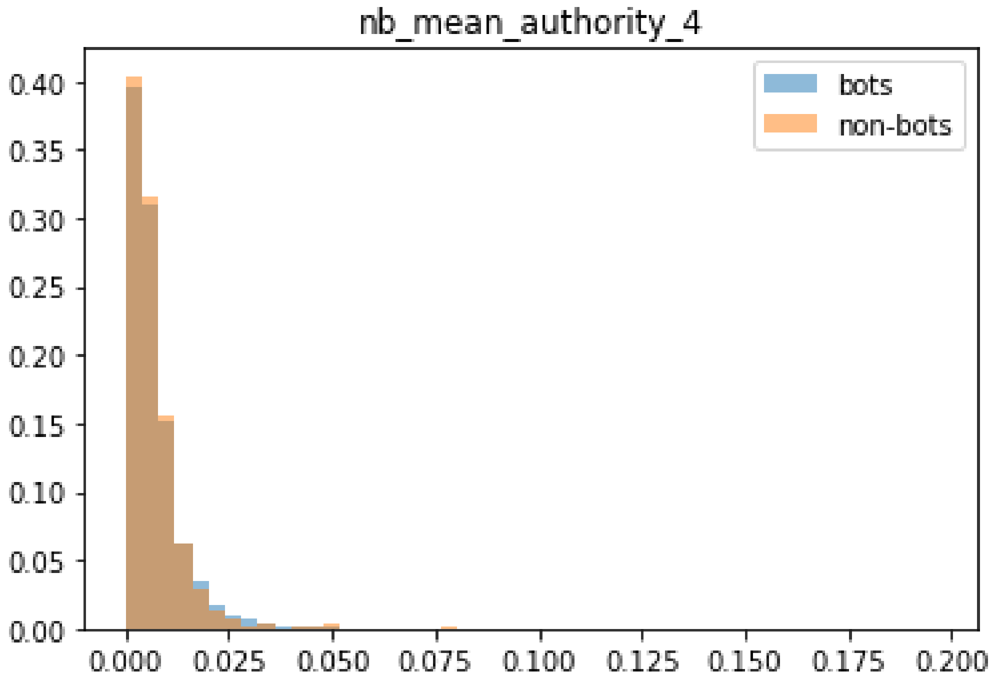
nb_mean_authority_4
bots
non-bots
0.40
0.35
0.30
0.25
0.20
0.15
0.10
0.05
0.00
0.000
0.025
0.050
0.075
0.100
0.125
0.150
0.175
0.200

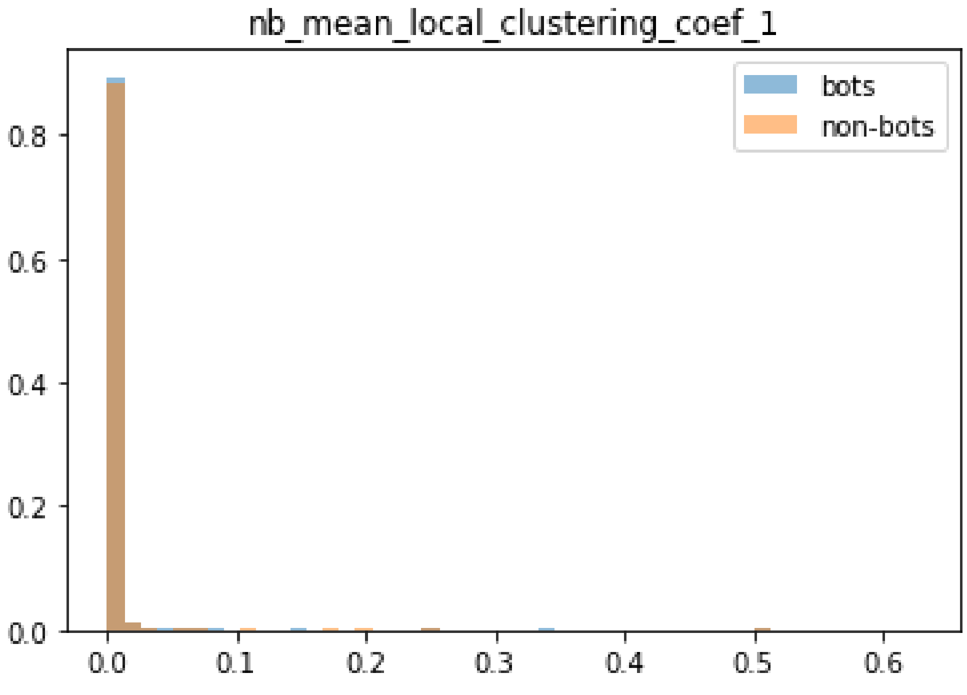
nb_mean_local_clustering_coef_1
bots
non-bots
0.8
0.6
0.4
0.2
0.0
0.0
0.1
0.2
0.3
0.4
0.5
0.6

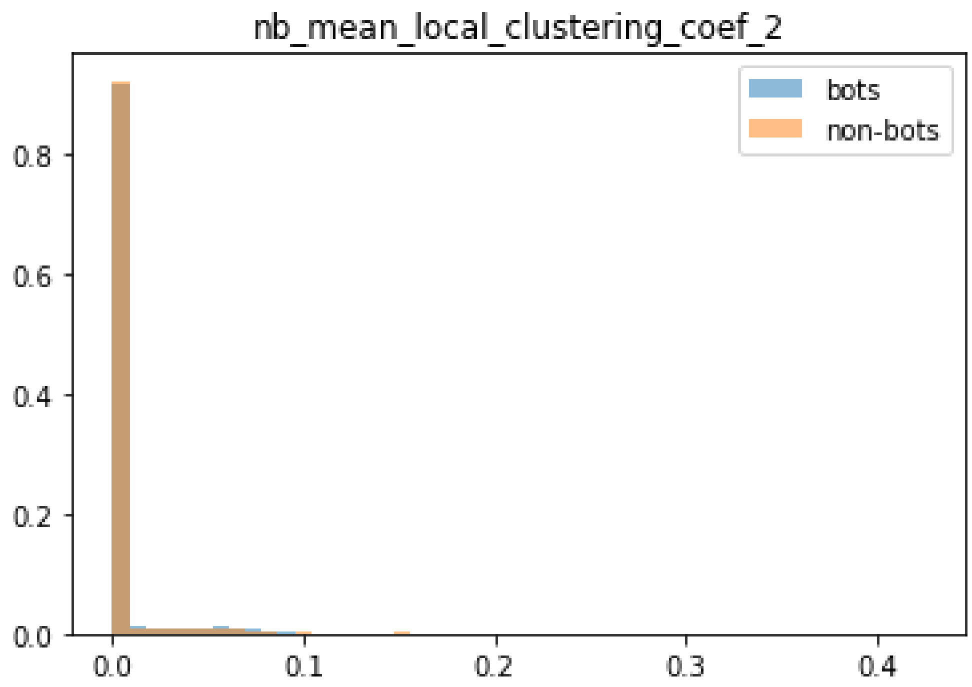
nb_mean_local_clustering_coef_2
bots
non-bots
0.8
0.6
0.4
0.2
0.0
0.0
0.1
0.2
0.3
0.4

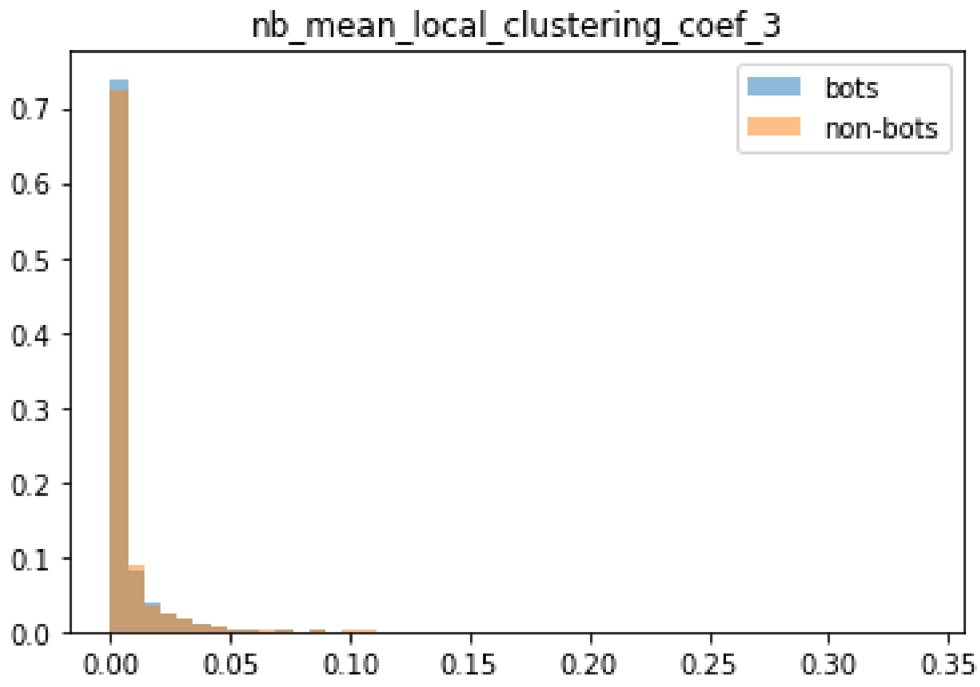
nb_mean_local_clustering_coef_3
bots
non-bots
0.7
0.6
0.5
0.4
0.3
0.2
0.1
0.0
0.00
0.05
0.10
0.15
0.20
0.25
0.30
0.35

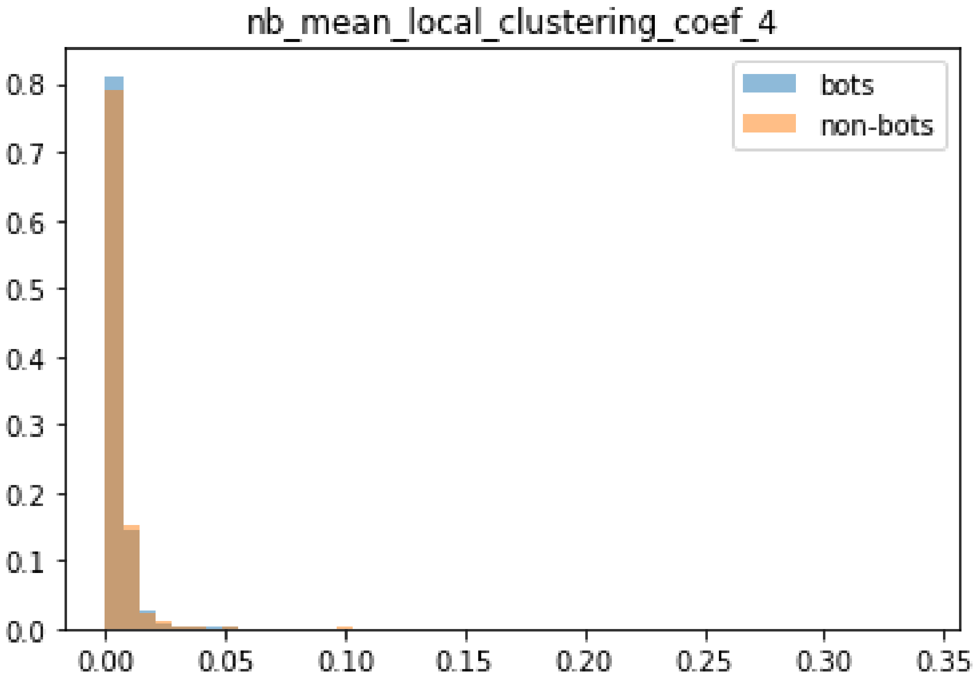
nb_mean_local_clustering_coef_4
bots
non-bots
0.0
0.1
0.2
0.3
0.4
0.5
0.6
0.7
0.8
0.00
0.05
0.10
0.15
0.20
0.25
0.30
0.35

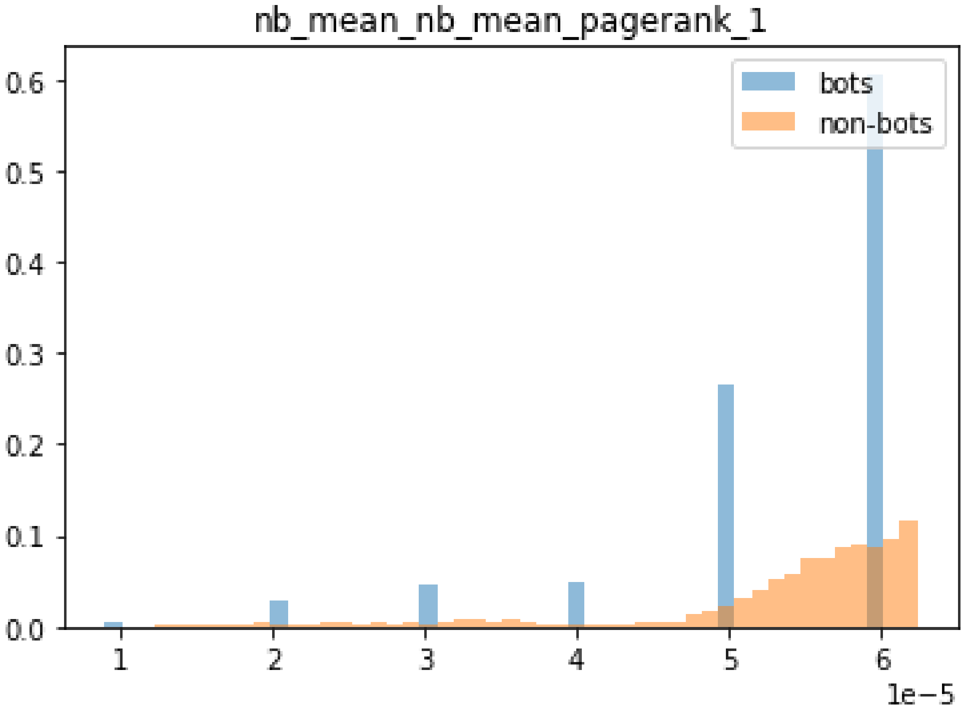
nb_mean_nb_mean_pagerank_1
bots
non-bots
0.0
0.1
0.2
0.3
0.4
0.5
0.6
1
2
3
4
5
6
1e−5

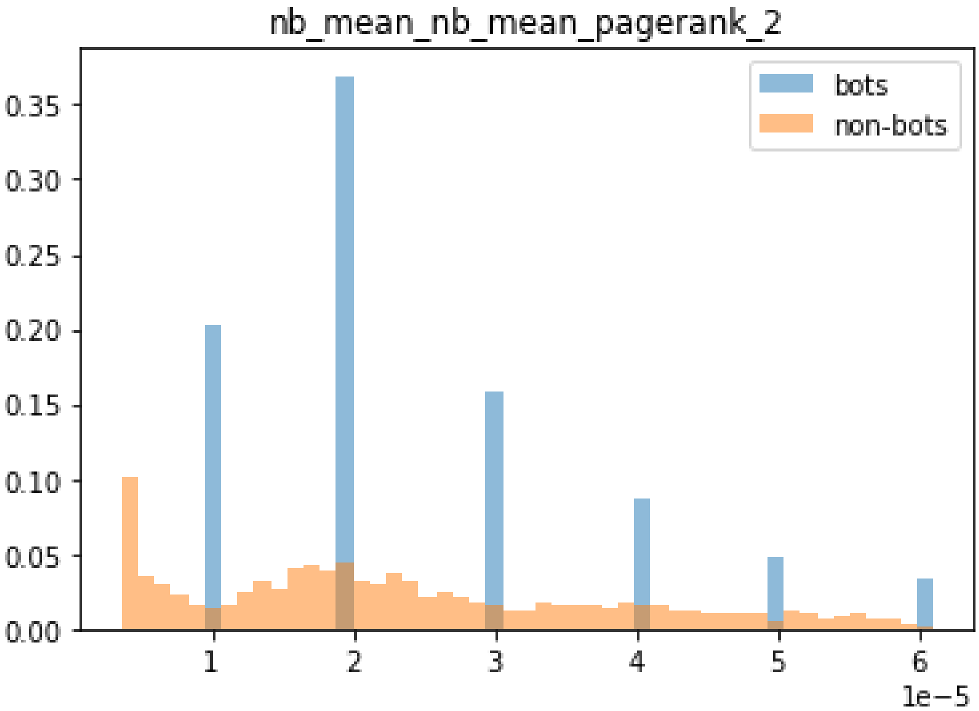
nb_mean_nb_mean_pagerank_2
bots
non-bots
0.35
0.30
0.25
0.20
0.15
0.10
0.05
0.00
1
2
3
4
5
6
1e−5

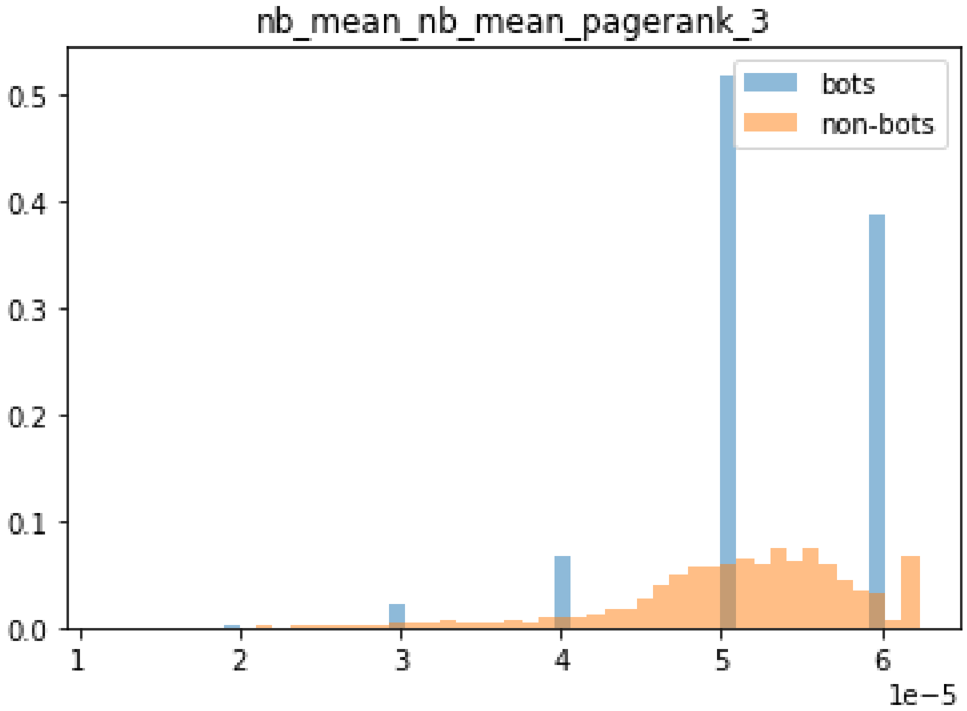
nb_mean_nb_mean_pagerank_3
bots
non-bots
0.5
0.4
0.3
0.2
0.1
0.0
1
2
3
4
5
6
1e−5

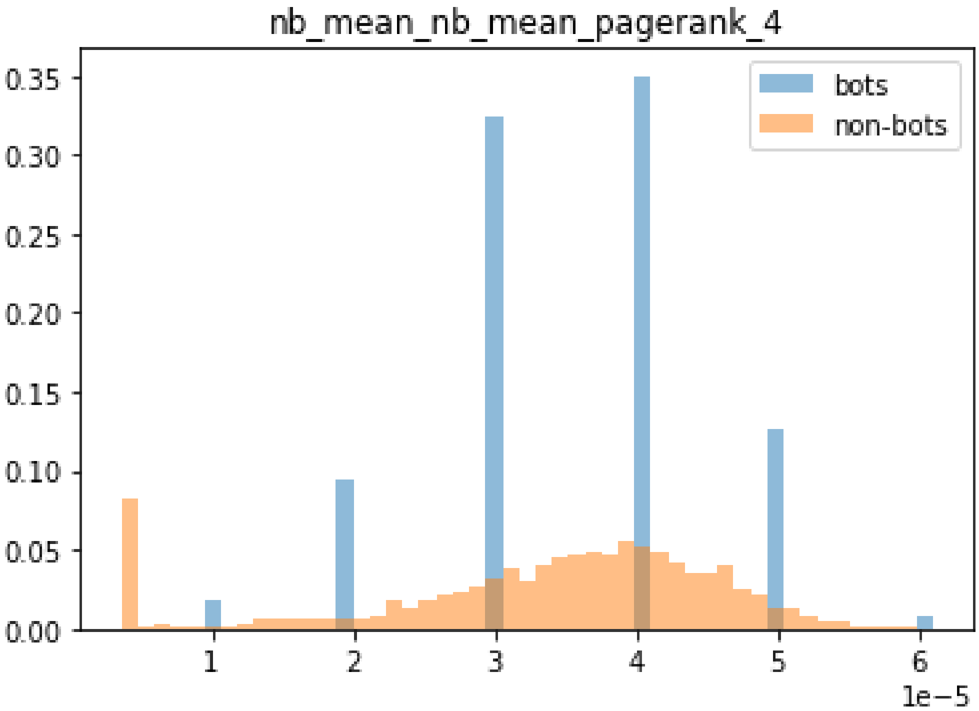
nb_mean_nb_mean_pagerank_4
0.35
0.30
0.25
0.20
0.15
0.10
0.05
0.00
1
2
3
4
5
6
1e−5
bots
non-bots

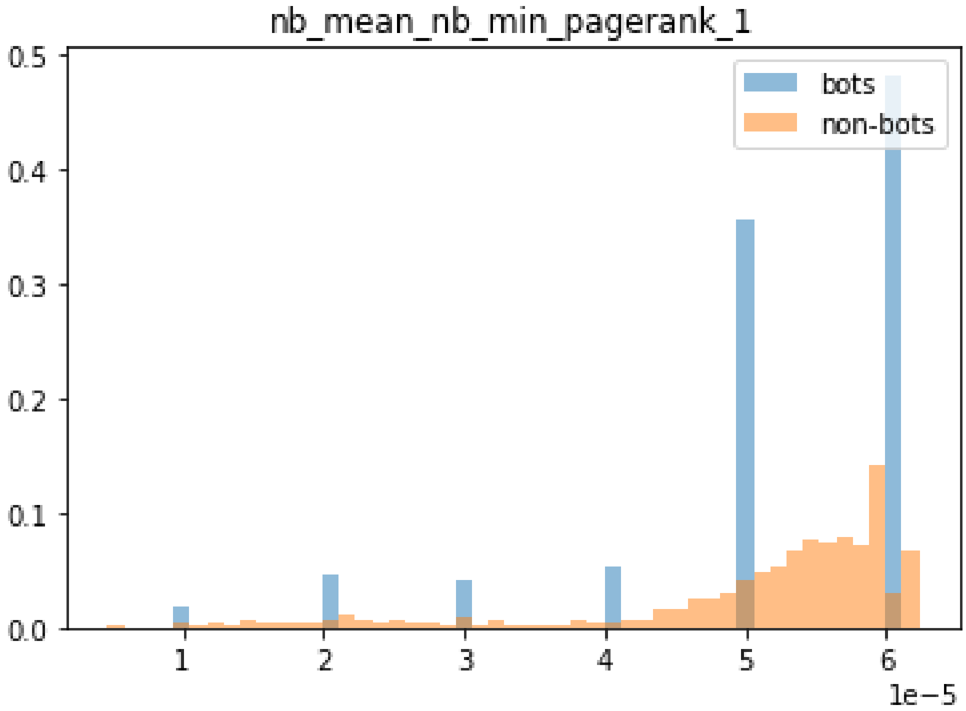
nb_mean_nb_min_pagerank_1
0.5
0.4
0.3
0.2
0.1
0.0
1
2
3
4
5
6
1e−5
bots
non-bots

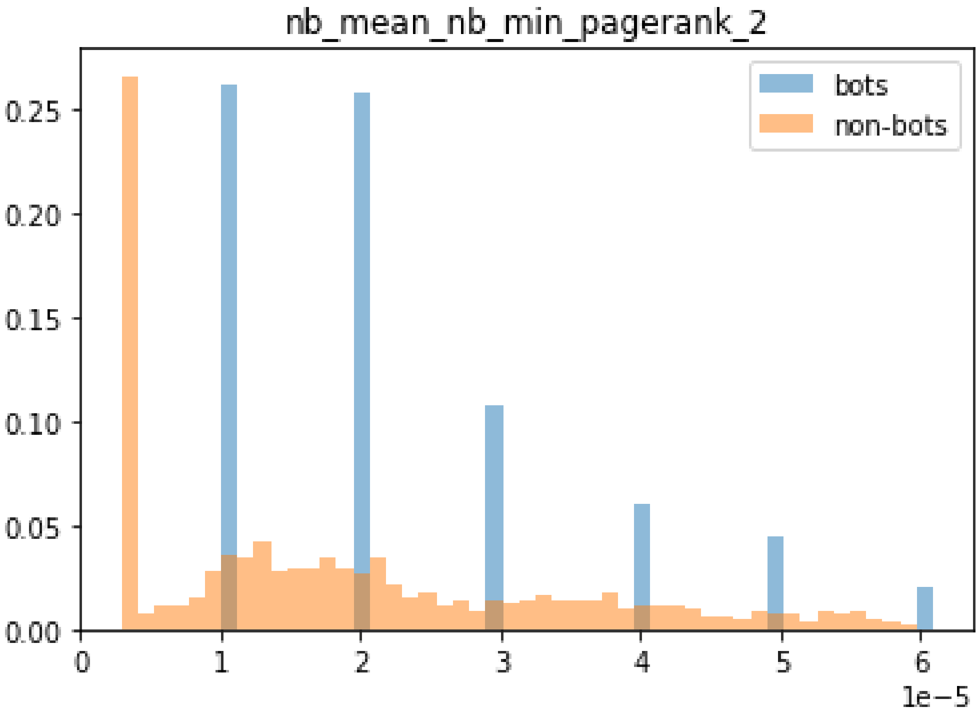
nb_mean_nb_min_pagerank_2
bots
non-bots
0.25
0.20
0.15
0.10
0.05
0.00
0
1
2
3
4
5
6
1e−5

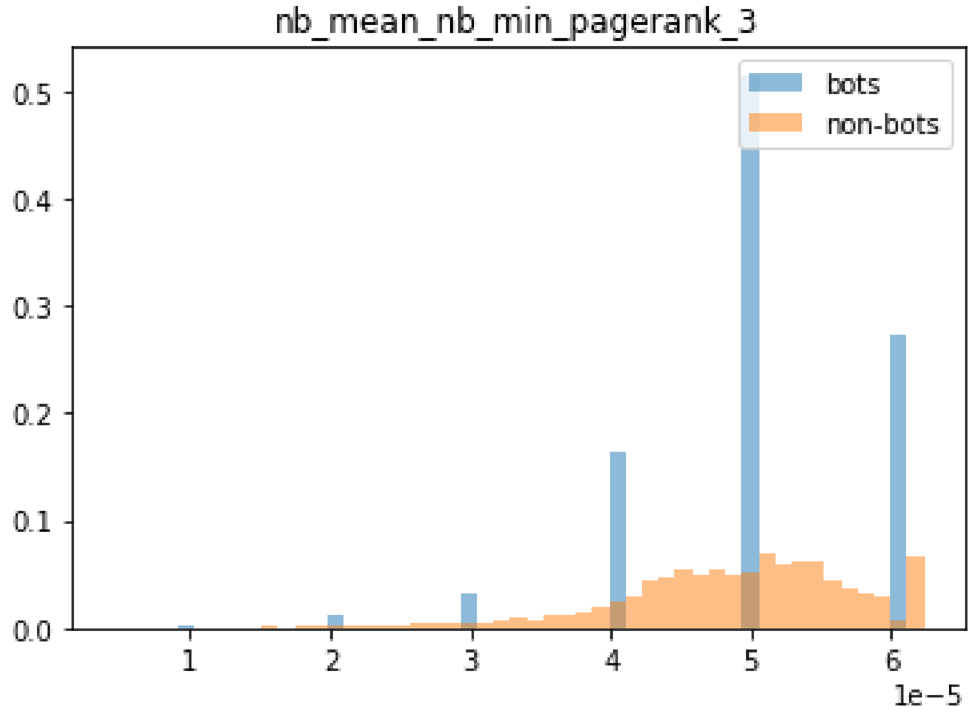
nb_mean_nb_min_pagerank_3
bots
non-bots
0.5
0.4
0.3
0.2
0.1
0.0
1
2
3
4
5
6
1e−5

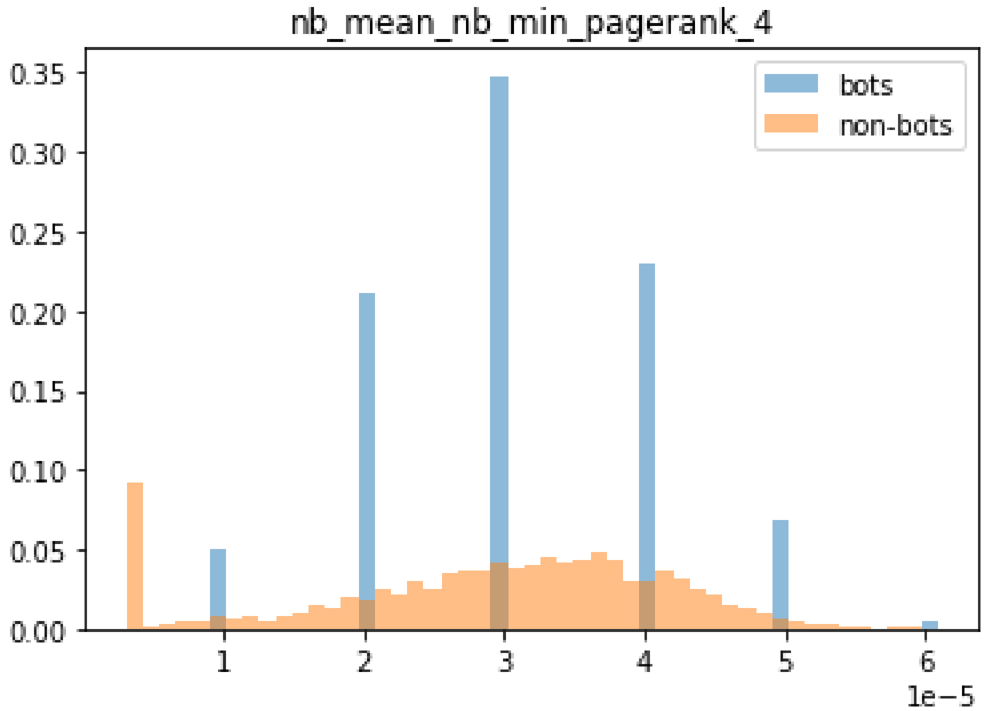
nb_mean_nb_min_pagerank_4
bots
non-bots
0.35
0.30
0.25
0.20
0.15
0.10
0.05
0.00
1
2
3
4
5
6
1e−5

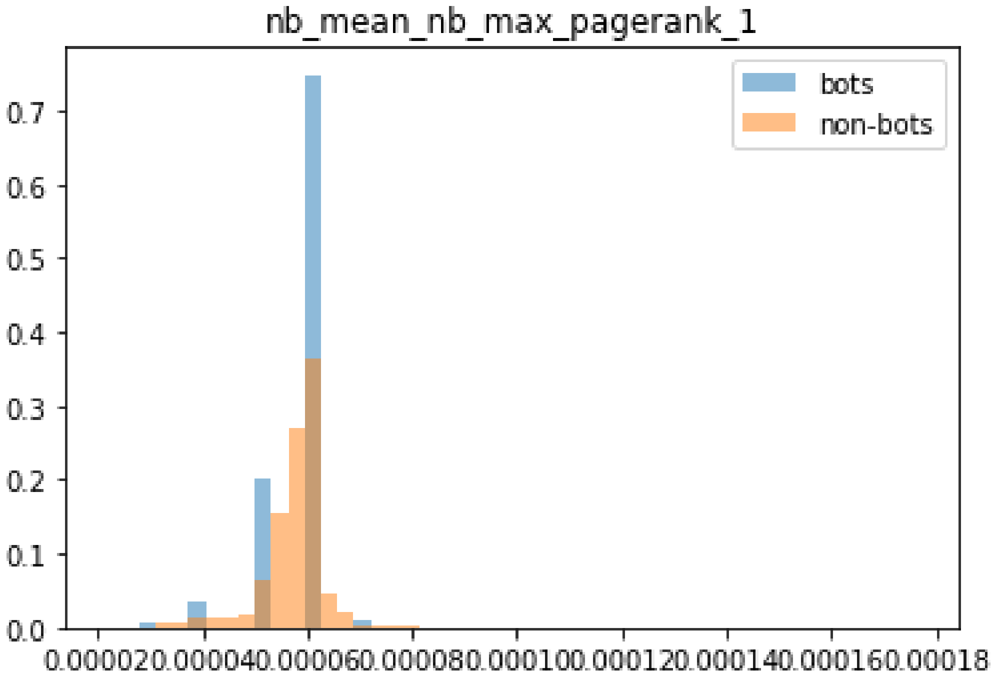
nb_mean_nb_max_pagerank_1
bots
non-bots
0.7
0.6
0.5
0.4
0.3
0.2
0.1
0.0
0.00002
0.00004
0.00006
0.00008
0.00010
0.00012
0.00014
0.00016
0.00018

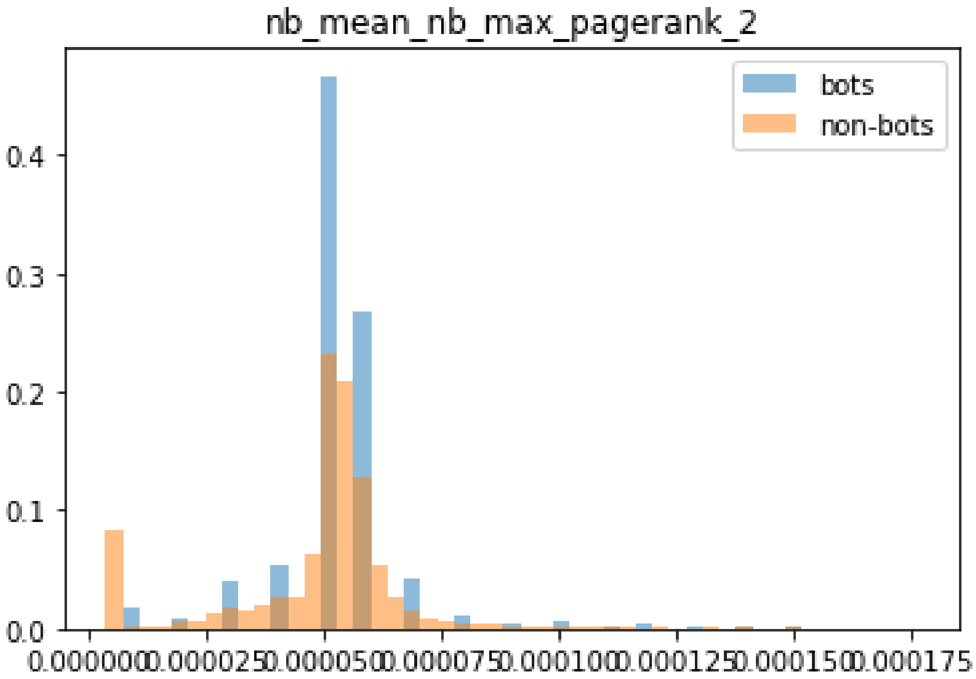
nb_mean_nb_max_pagerank_2
bots
non-bots
0.4
0.3
0.2
0.1
0.0
0.000000
0.000025
0.000050
0.000075
0.000100
0.000125
0.000150
0.000175

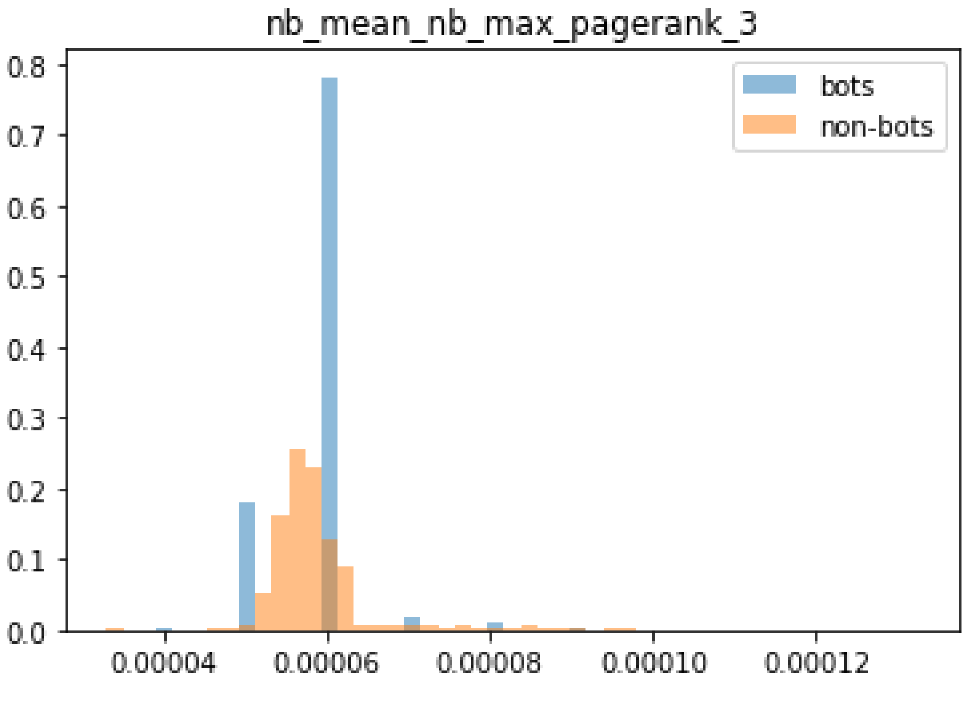
nb_mean_nb_max_pagerank_3
bots
non-bots
0.8
0.7
0.6
0.5
0.4
0.3
0.2
0.1
0.0
0.00004
0.00006
0.00008
0.00010
0.00012

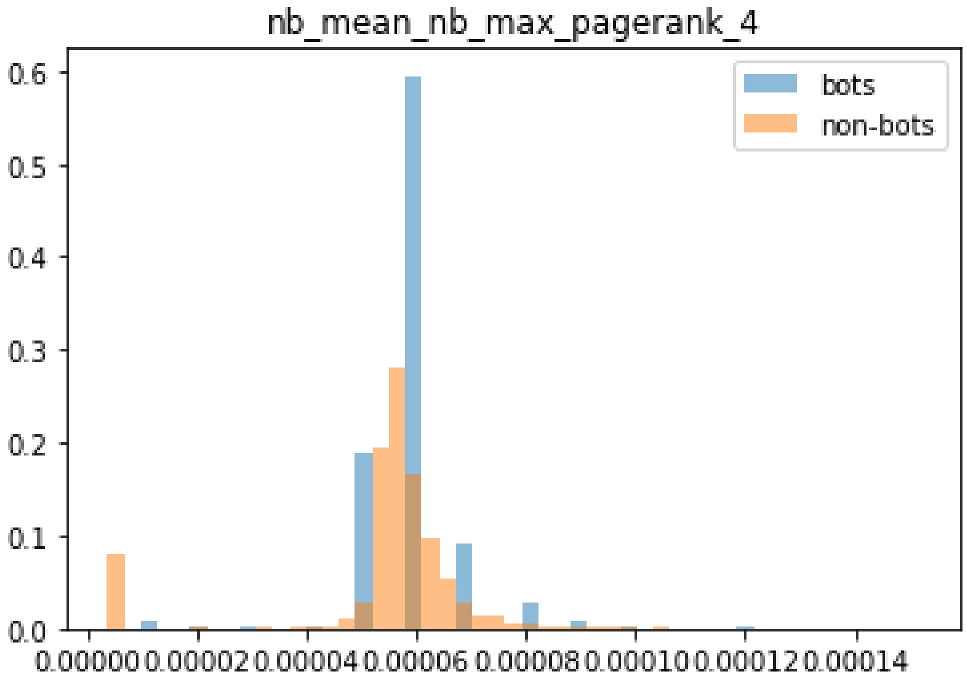
nb_mean_nb_max_pagerank_4
bots
non-bots
0.6
0.5
0.4
0.3
0.2
0.1
0.0
0.00000
0.00002
0.00004
0.00006
0.00008
0.00010
0.00012
0.00014

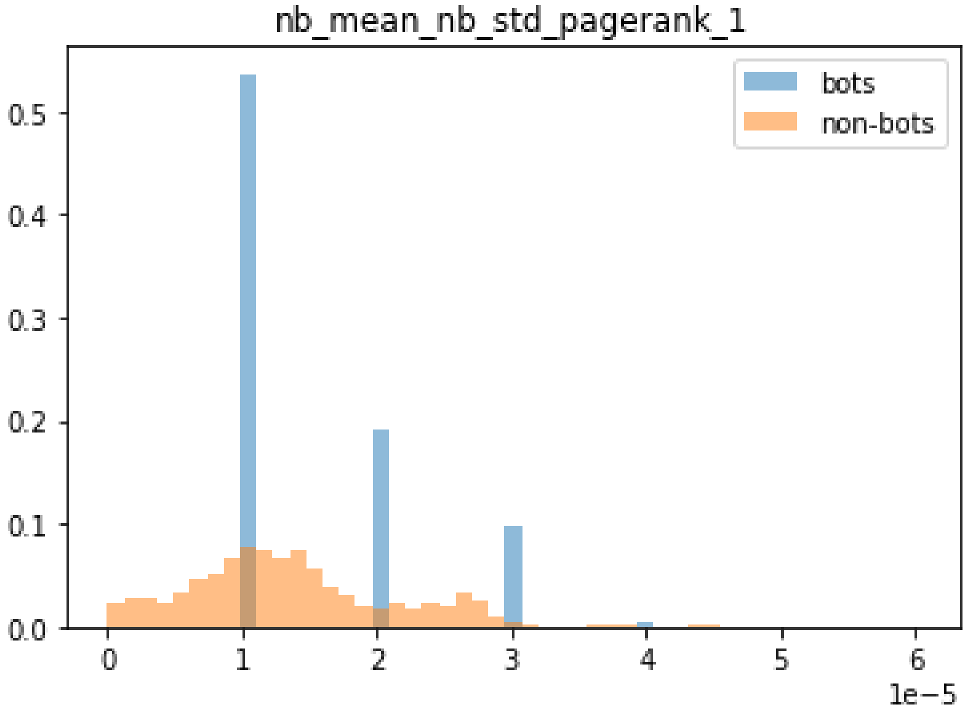
nb_mean_nb_std_pagerank_1
bots
non-bots
0.5
0.4
0.3
0.2
0.1
0.0
0
1
2
3
4
5
6
1e−5

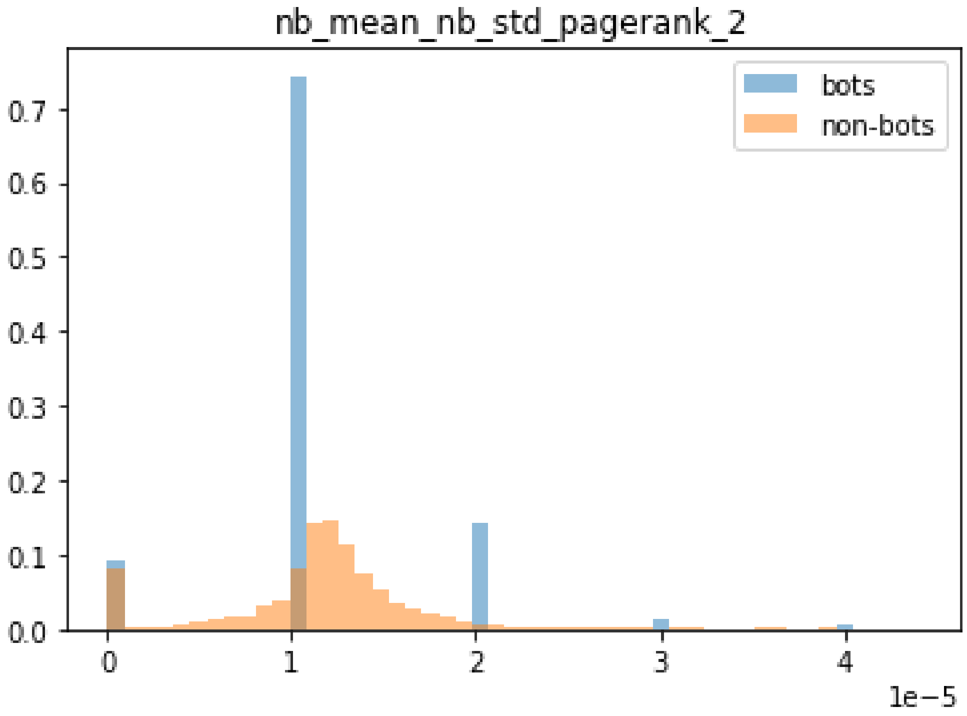
nb_mean_nb_std_pagerank_2
bots
non-bots
0.7
0.6
0.5
0.4
0.3
0.2
0.1
0.0
0
1
2
3
4
1e−5

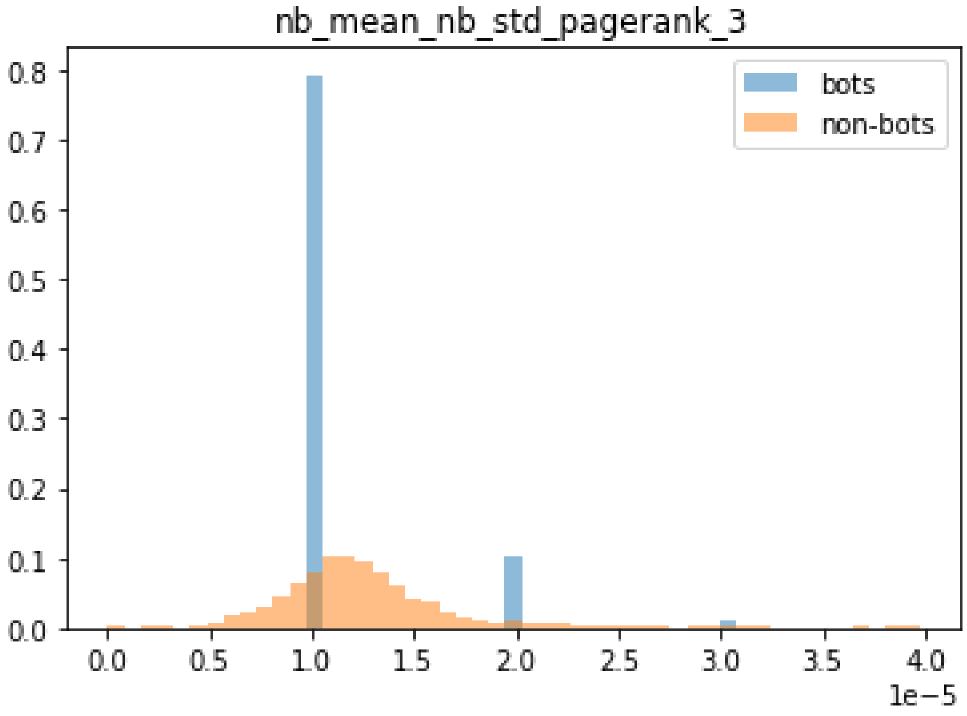
nb_mean_nb_std_pagerank_3
bots
non-bots
0.8
0.7
0.6
0.5
0.4
0.3
0.2
0.1
0.0
0.0
0.5
1.0
1.5
2.0
2.5
3.0
3.5
4.0
1e−5

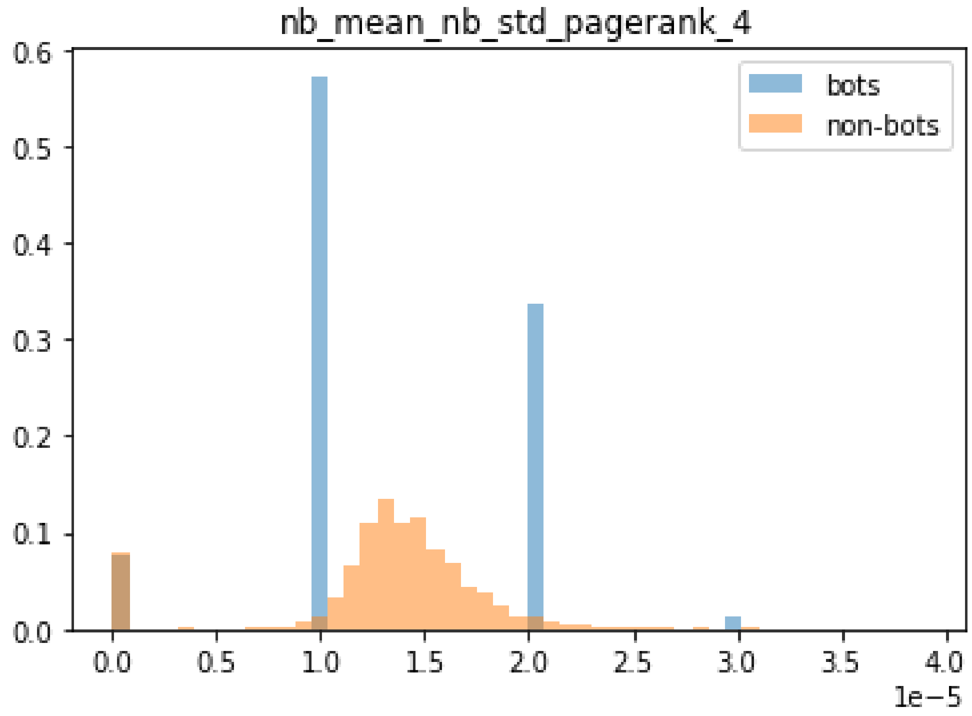
nb_mean_nb_std_pagerank_4
bots
non-bots
0.6
0.5
0.4
0.3
0.2
0.1
0.0
0.0
0.5
1.0
1.5
2.0
2.5
3.0
3.5
4.0
1e−5

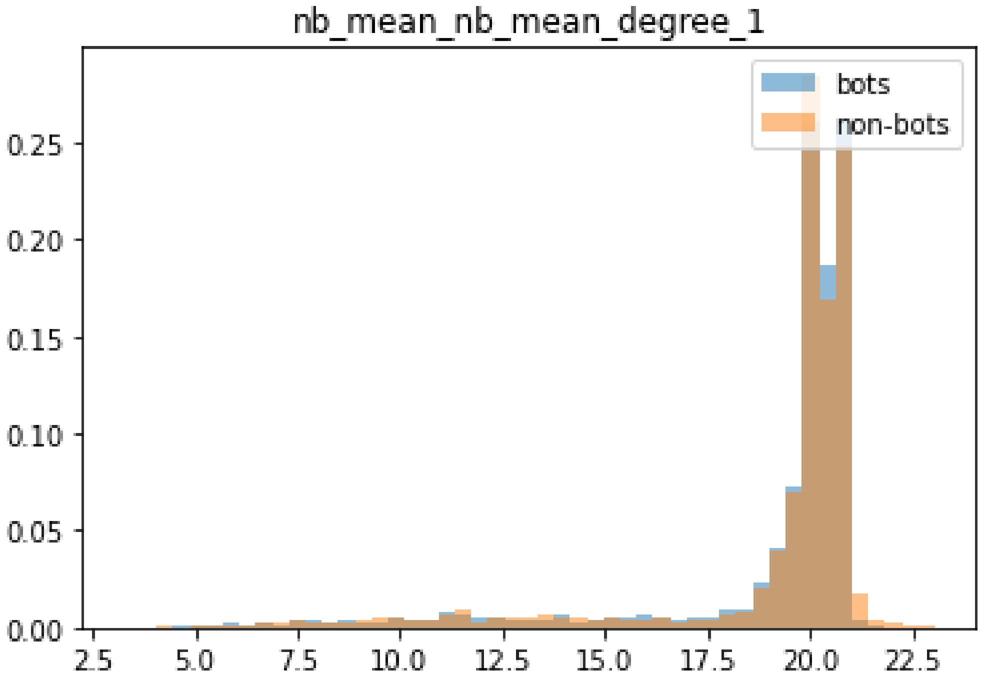
nb_mean_nb_mean_degree_1
bots
non-bots
0.25
0.20
0.15
0.10
0.05
0.00
2.5
5.0
7.5
10.0
12.5
15.0
17.5
20.0
22.5

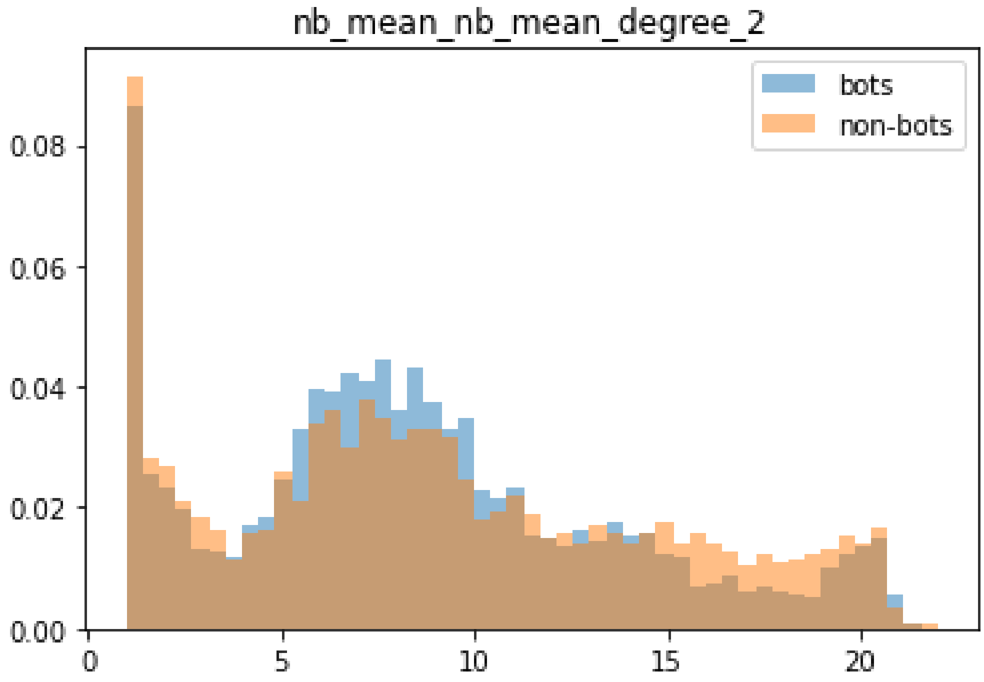
nb_mean_nb_mean_degree_2
bots
non-bots
0.00
0.02
0.04
0.06
0.08
0
5
10
15
20

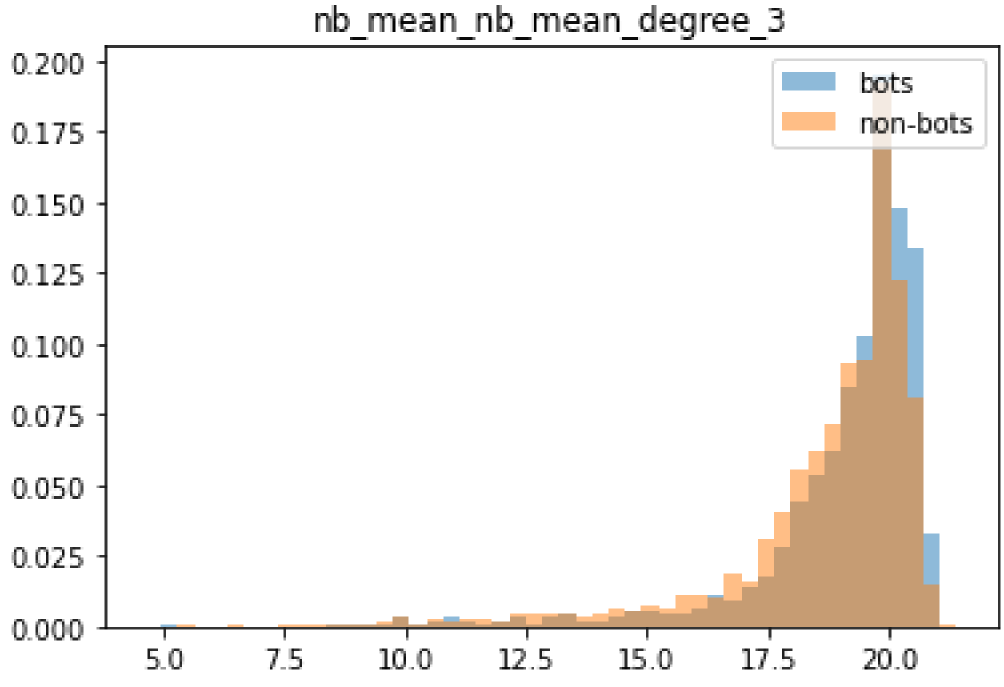
nb_mean_nb_mean_degree_3
bots
non-bots
0.000
0.025
0.050
0.075
0.100
0.125
0.150
0.175
0.200
5.0
7.5
10.0
12.5
15.0
17.5
20.0

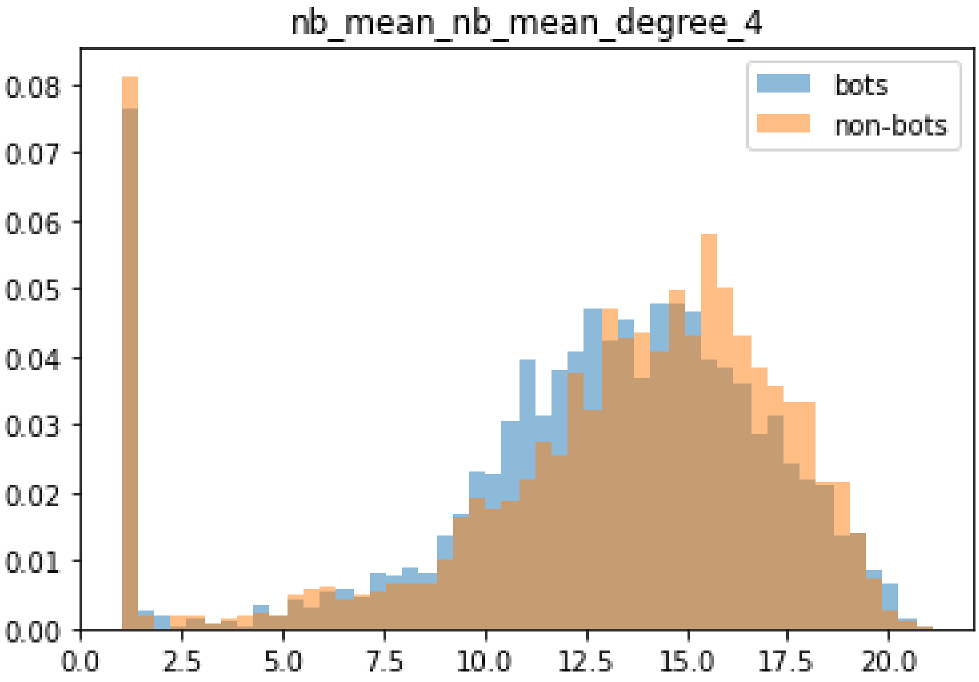
nb_mean_nb_mean_degree_4
bots
non-bots
0.00
0.01
0.02
0.03
0.04
0.05
0.06
0.07
0.08
0.0
2.5
5.0
7.5
10.0
12.5
15.0
17.5
20.0

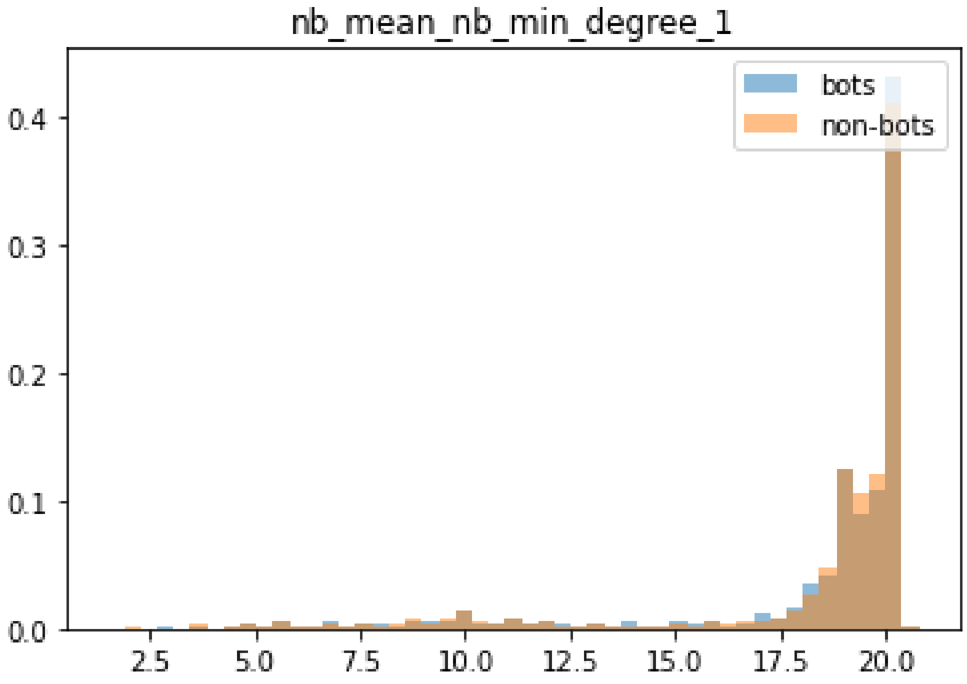
nb_mean_nb_min_degree_1
bots
non-bots
0.0
0.1
0.2
0.3
0.4
2.5
5.0
7.5
10.0
12.5
15.0
17.5
20.0

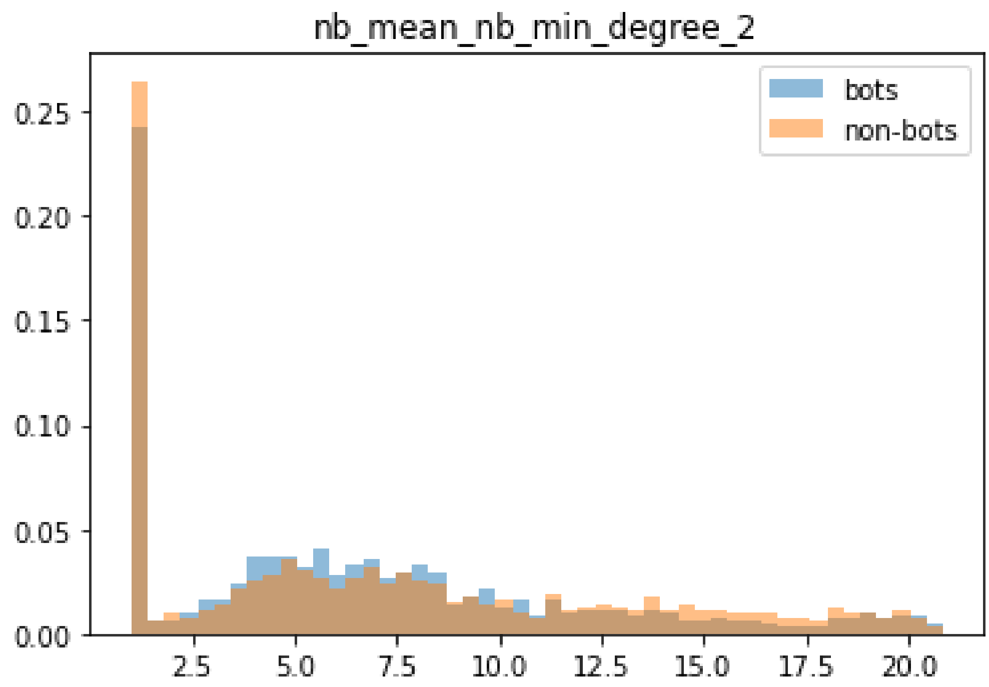
nb_mean_nb_min_degree_2
bots
non-bots
0.25
0.20
0.15
0.10
0.05
0.00
2.5
5.0
7.5
10.0
12.5
15.0
17.5
20.0

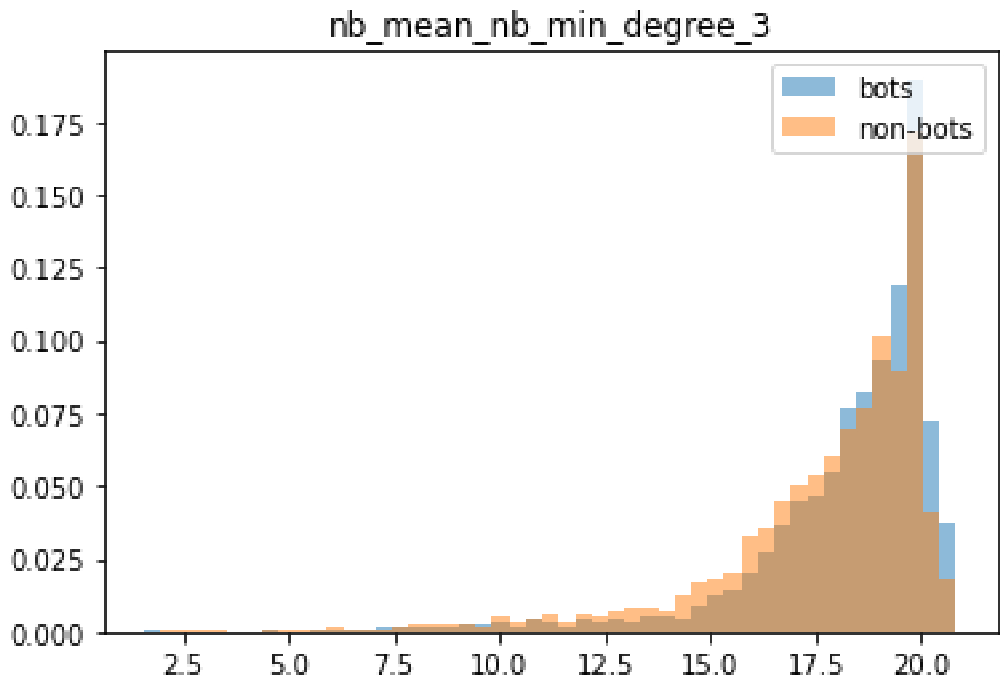
nb_mean_nb_min_degree_3
bots
non-bots
0.175
0.150
0.125
0.100
0.075
0.050
0.025
0.000
2.5
5.0
7.5
10.0
12.5
15.0
17.5
20.0

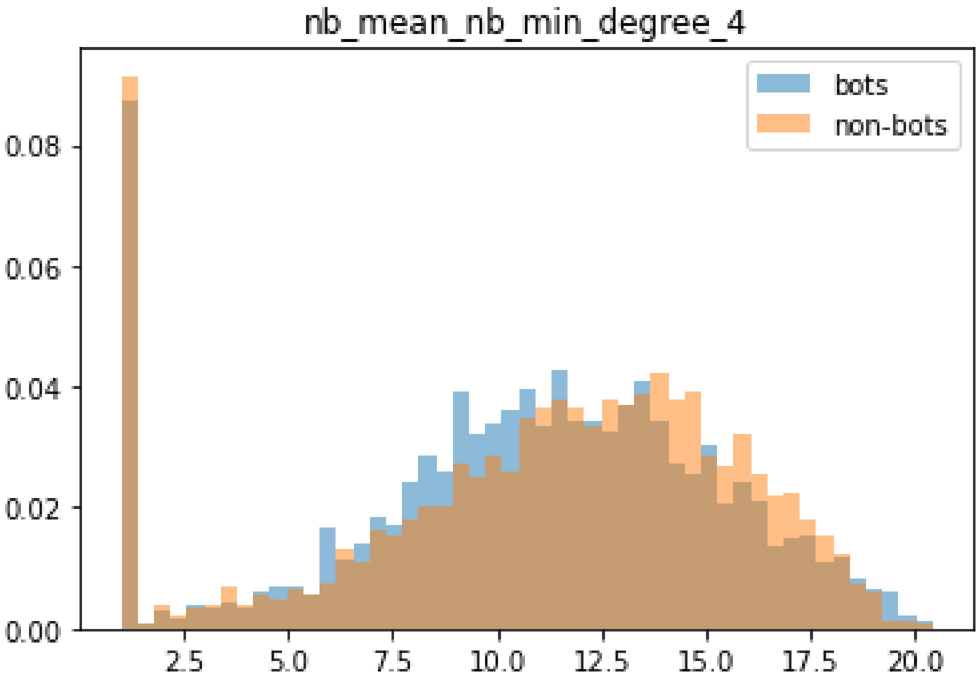
nb_mean_nb_min_degree_4
bots
non-bots
0.08
0.06
0.04
0.02
0.00
2.5
5.0
7.5
10.0
12.5
15.0
17.5
20.0

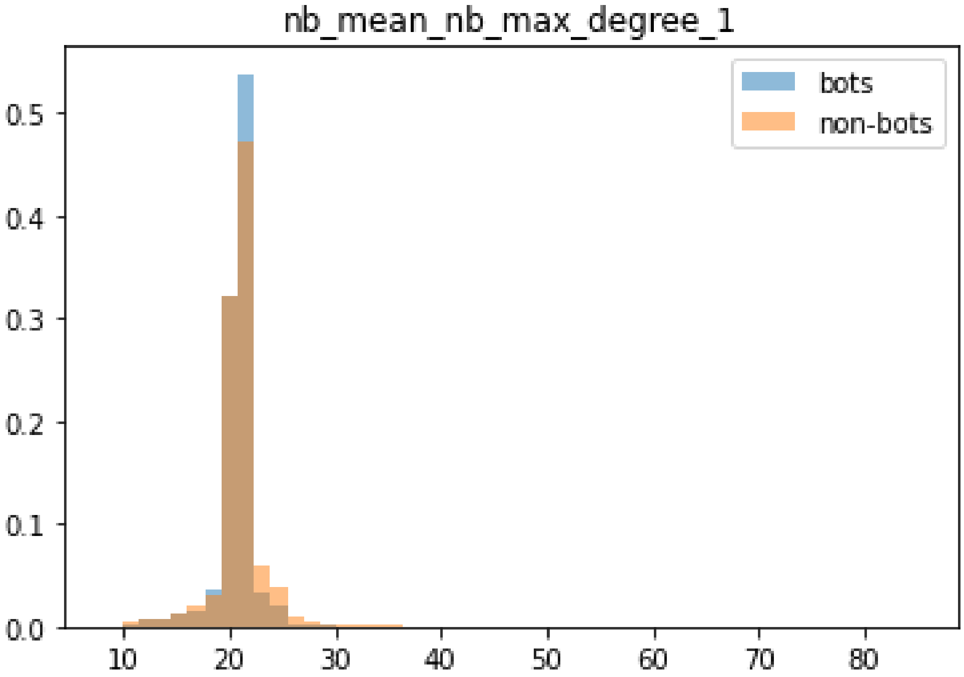
nb_mean_nb_max_degree_1
bots
non-bots
0.5
0.4
0.3
0.2
0.1
0.0
10
20
30
40
50
60
70
80

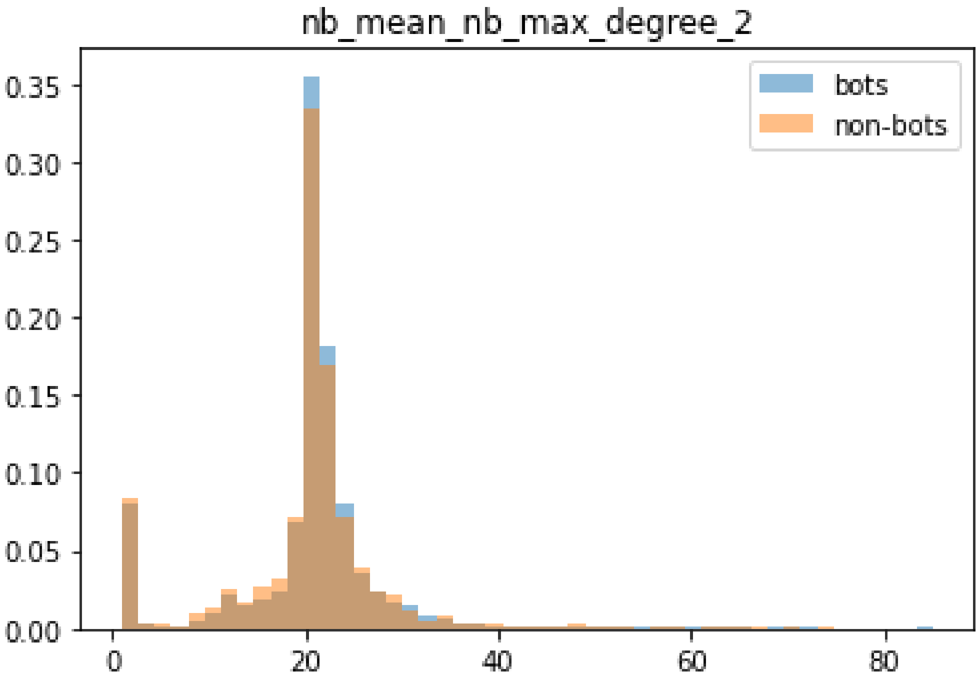
nb_mean_nb_max_degree_2
bots
non-bots
0.35
0.30
0.25
0.20
0.15
0.10
0.05
0.00
0
20
40
60
80

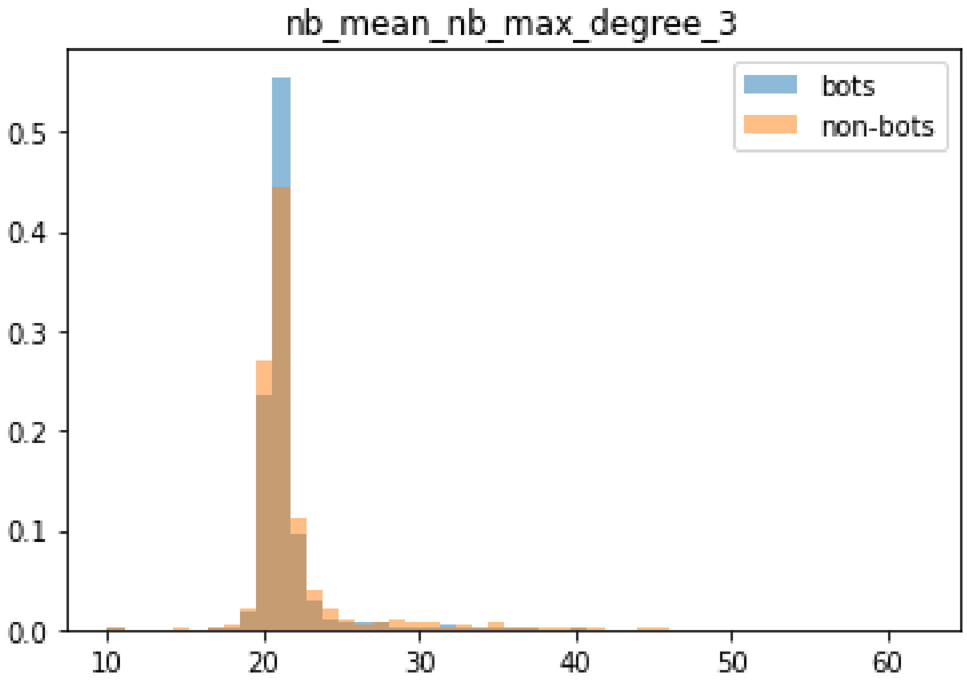
nb_mean_nb_max_degree_3
bots
non-bots
0.5
0.4
0.3
0.2
0.1
0.0
10
20
30
40
50
60

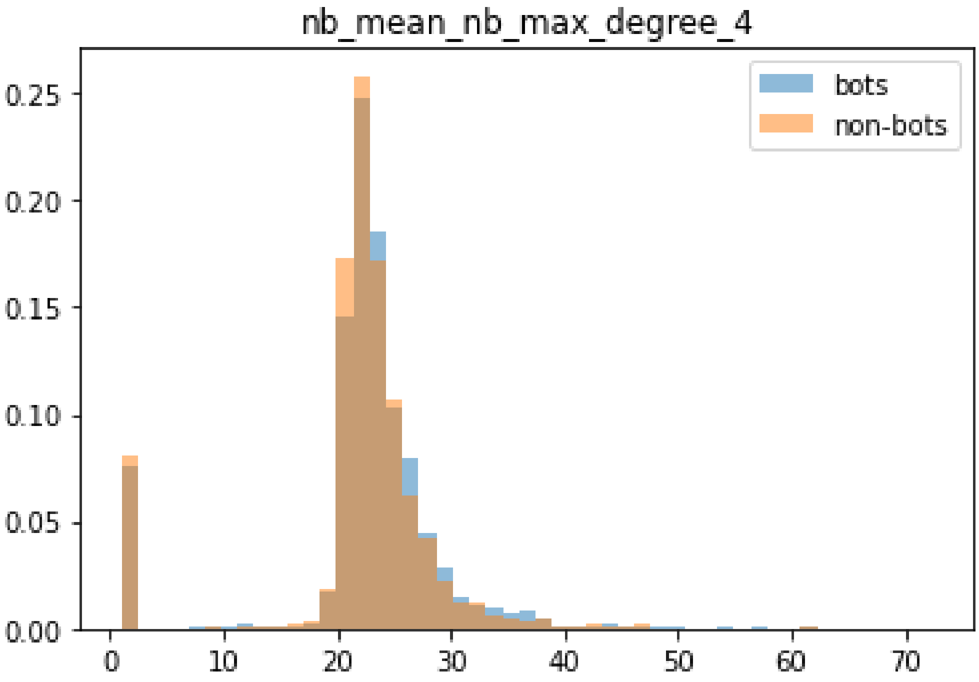
nb_mean_nb_max_degree_4
bots
non-bots
0.25
0.20
0.15
0.10
0.05
0.00
0
10
20
30
40
50
60
70

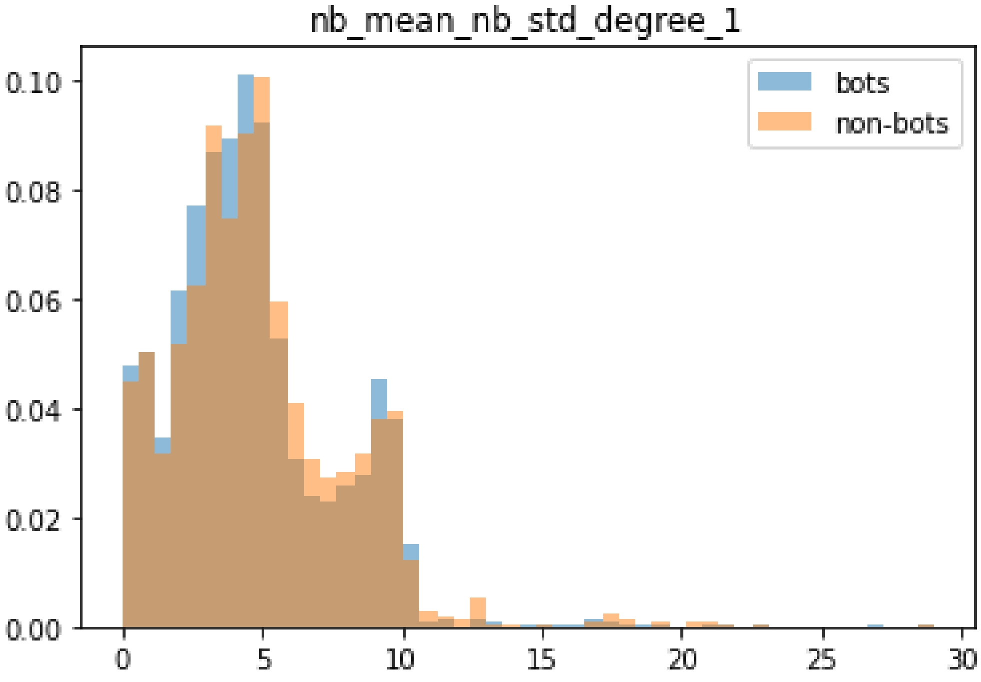
nb_mean_nb_std_degree_1
bots
non-bots
0.10
0.08
0.06
0.04
0.02
0.00
0
5
10
15
20
25
30

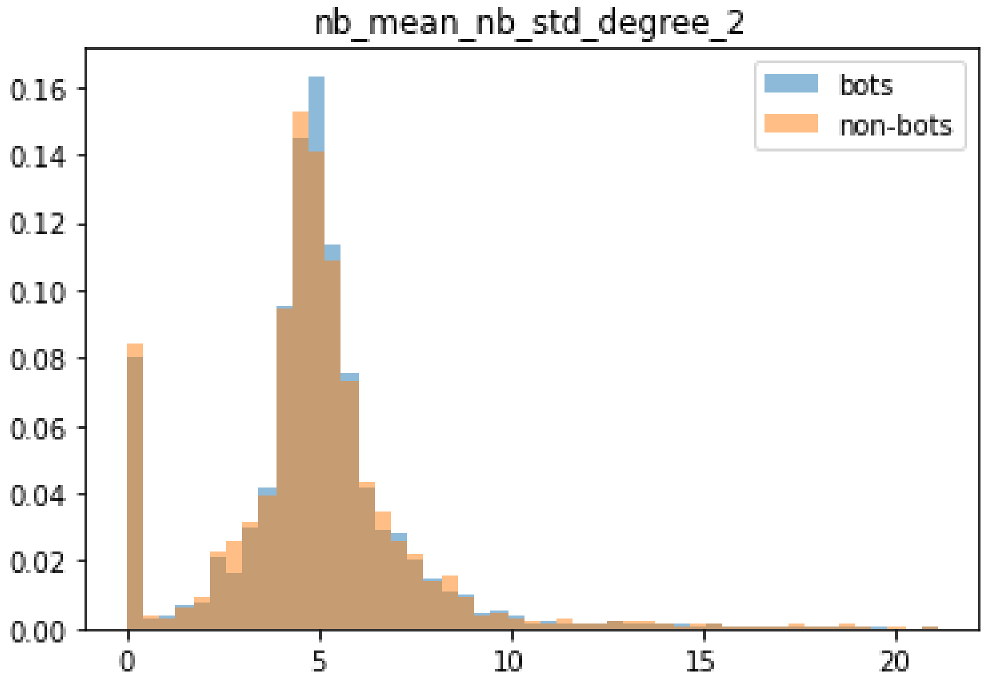
nb_mean_nb_std_degree_2
0.16
0.14
0.12
0.10
0.08
0.06
0.04
0.02
0.00
0
5
10
15
20
bots
non-bots

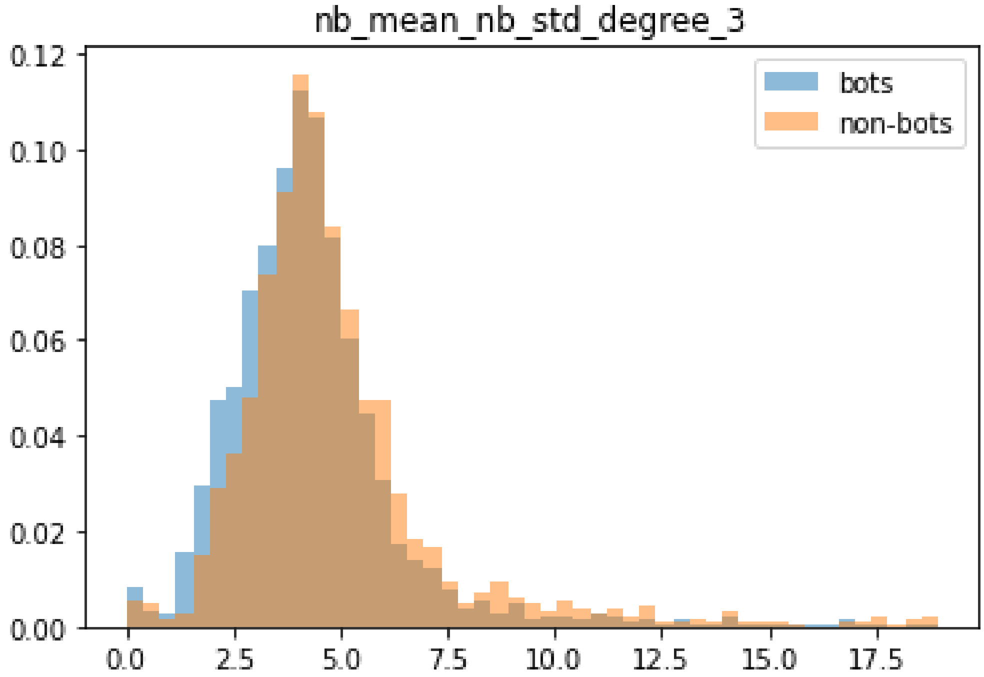
nb_mean_nb_std_degree_3
0.12
0.10
0.08
0.06
0.04
0.02
0.00
0.0
2.5
5.0
7.5
10.0
12.5
15.0
17.5
bots
non-bots

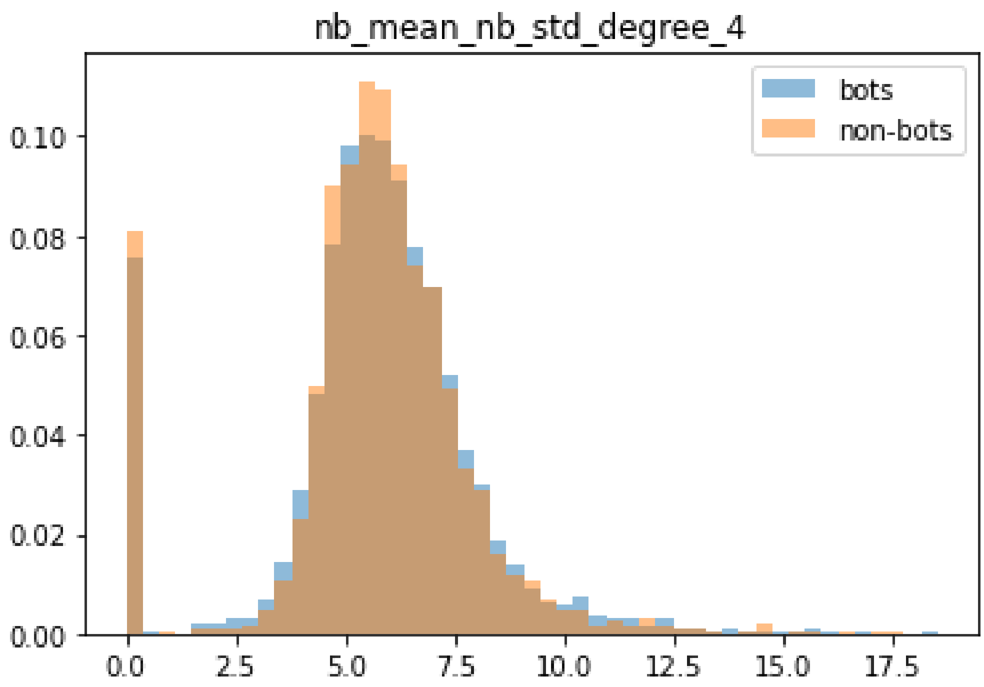
nb_mean_nb_std_degree_4
bots
non-bots
0.10
0.08
0.06
0.04
0.02
0.00
0.0
2.5
5.0
7.5
10.0
12.5
15.0
17.5

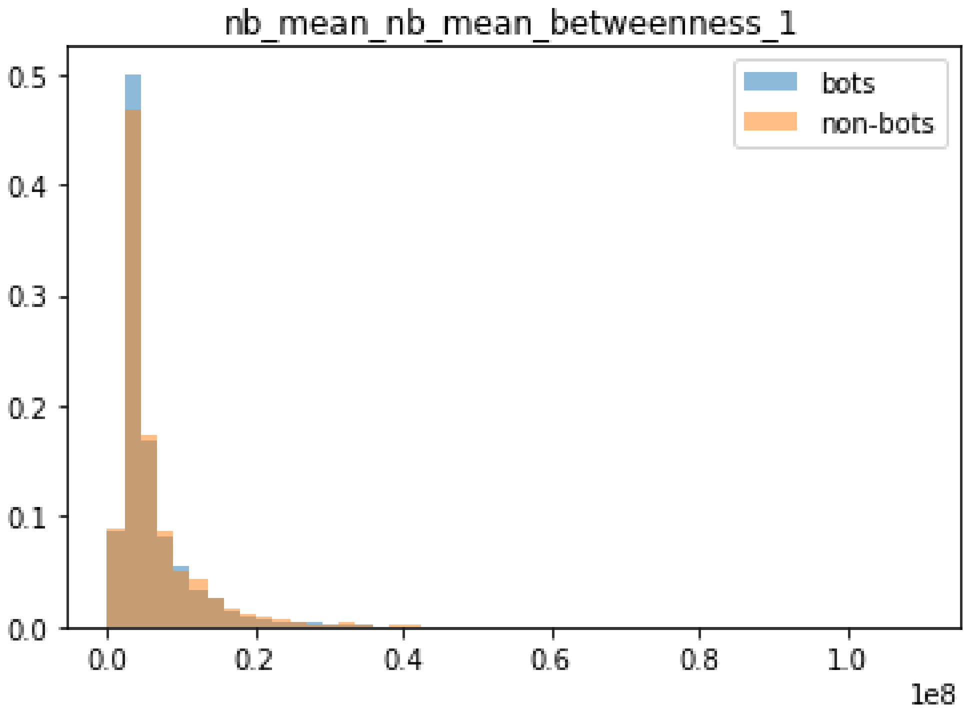
nb_mean_nb_mean_betweenness_1
bots
non-bots
0.5
0.4
0.3
0.2
0.1
0.0
0.0
0.2
0.4
0.6
0.8
1.0
1e8

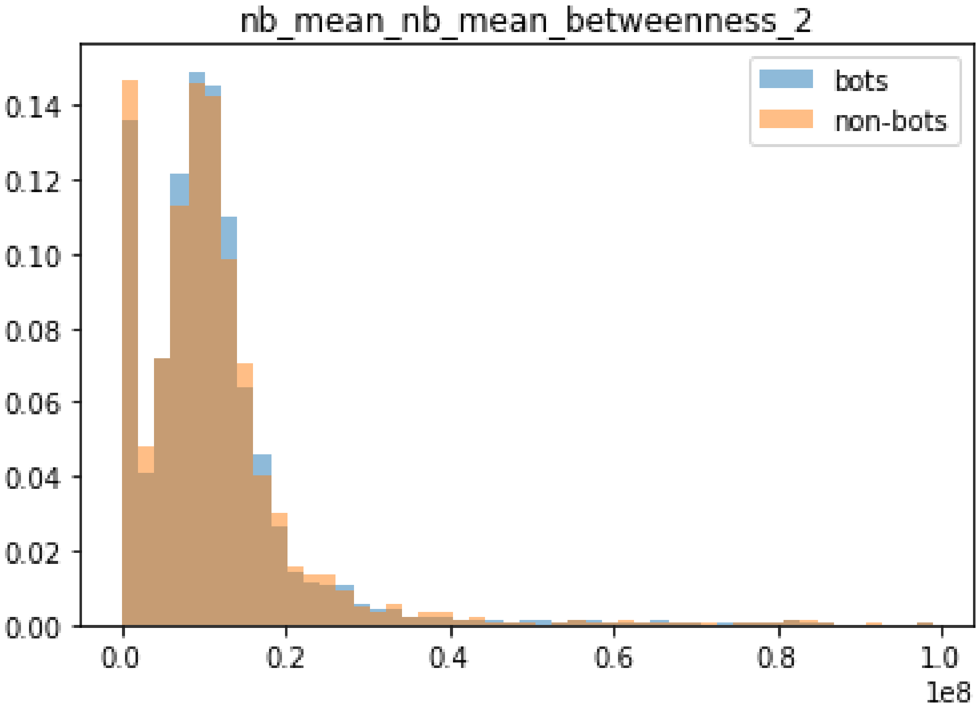
nb_mean_nb_mean_betweenness_2
bots
non-bots
0.00
0.02
0.04
0.06
0.08
0.10
0.12
0.14
0.0
0.2
0.4
0.6
0.8
1.0
1e8

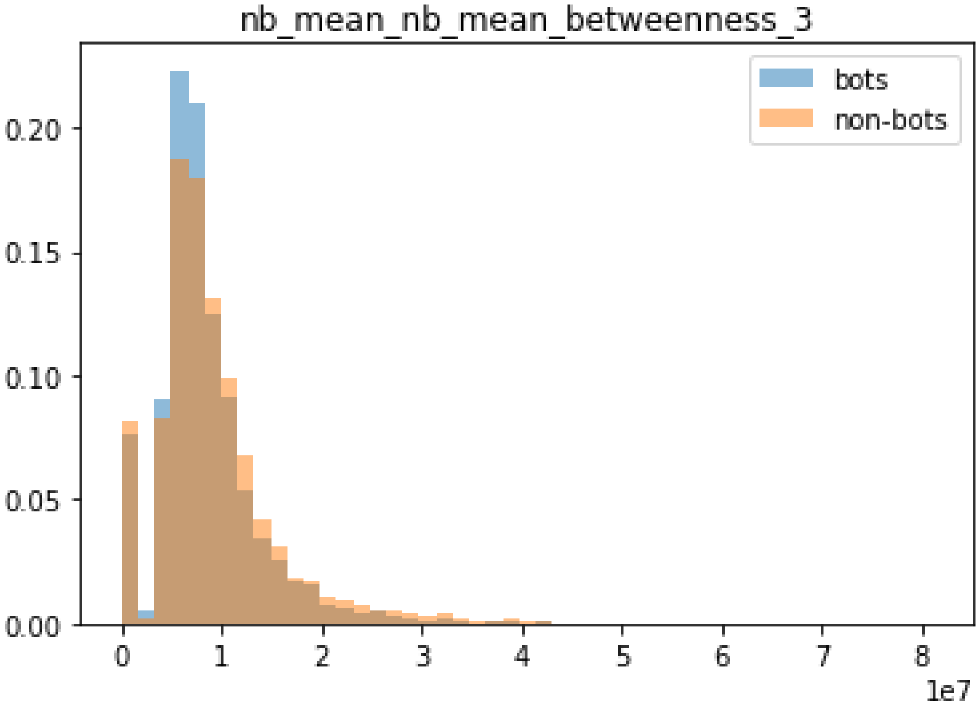
nb_mean_nb_mean_betweenness_3
bots
non-bots
0.00
0.05
0.10
0.15
0.20
0
1
2
3
4
5
6
7
8
1e7

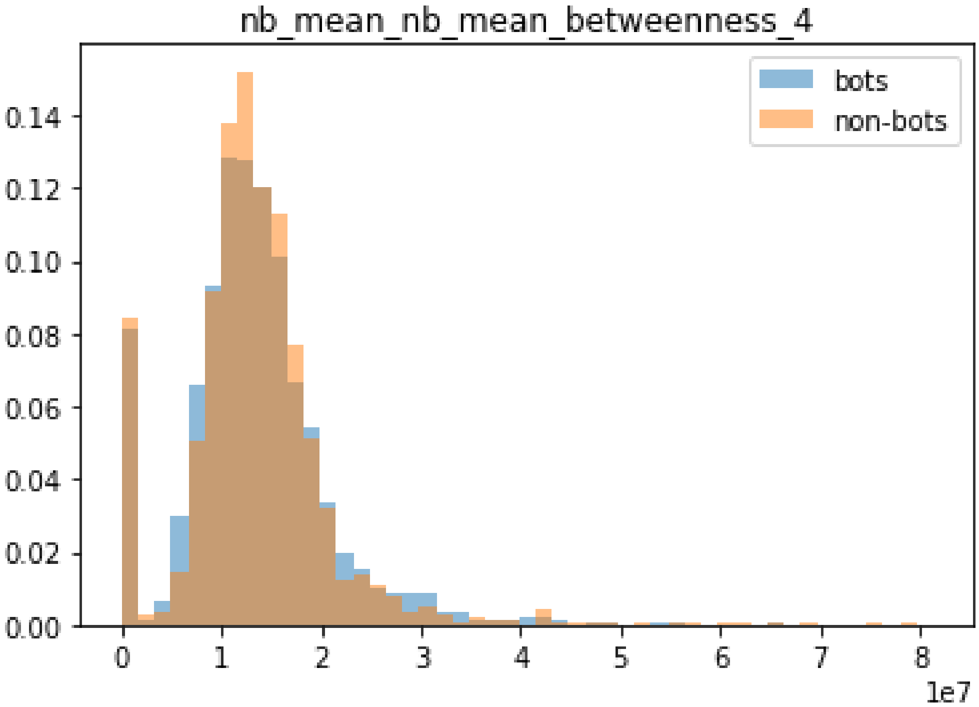
nb_mean_nb_mean_betweenness_4
bots
non-bots
0.14
0.12
0.10
0.08
0.06
0.04
0.02
0.00
0
1
2
3
4
5
6
7
8
1e7

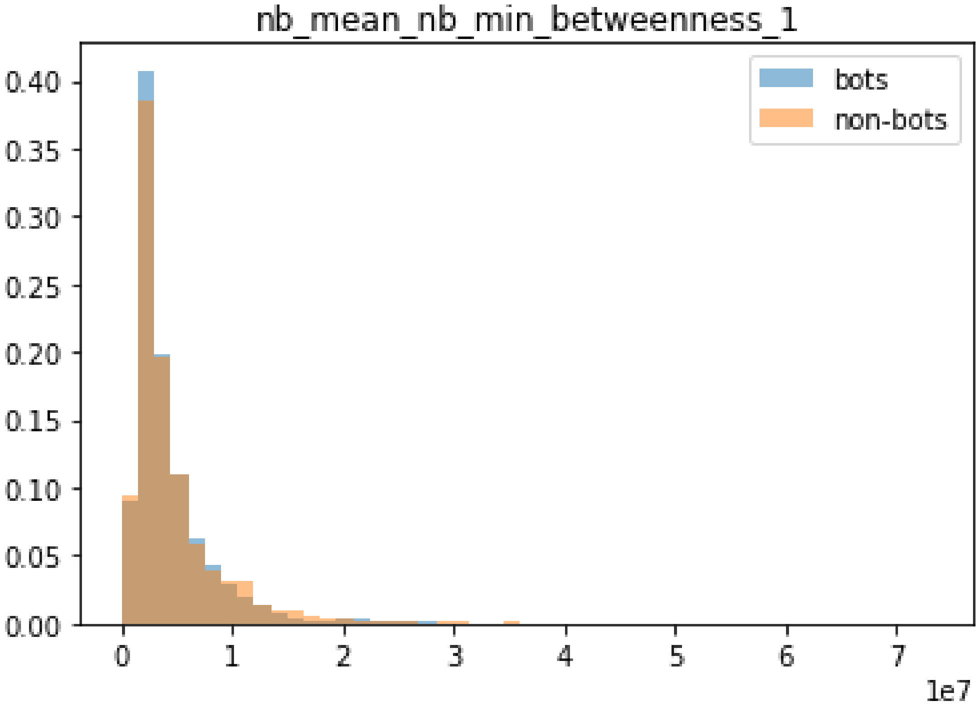
nb_mean_nb_min_betweenness_1
bots
non-bots
0.40
0.35
0.30
0.25
0.20
0.15
0.10
0.05
0.00
0
1
2
3
4
5
6
7
1e7

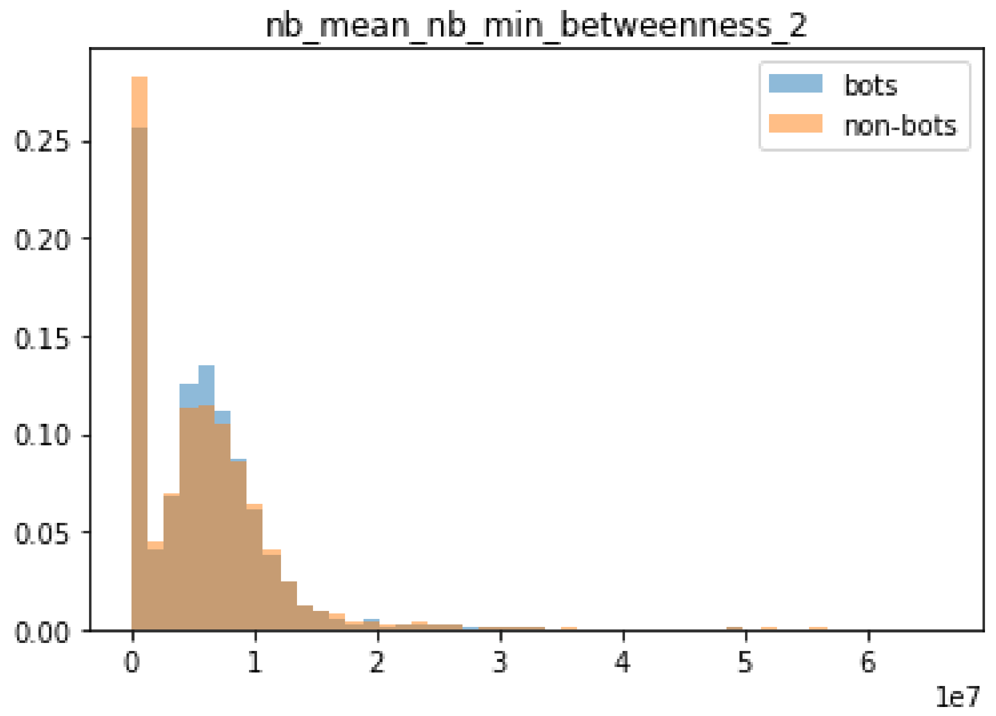
nb_mean_nb_min_betweenness_2
bots
non-bots
0.25
0.20
0.15
0.10
0.05
0.00
0
1
2
3
4
5
6
1e7

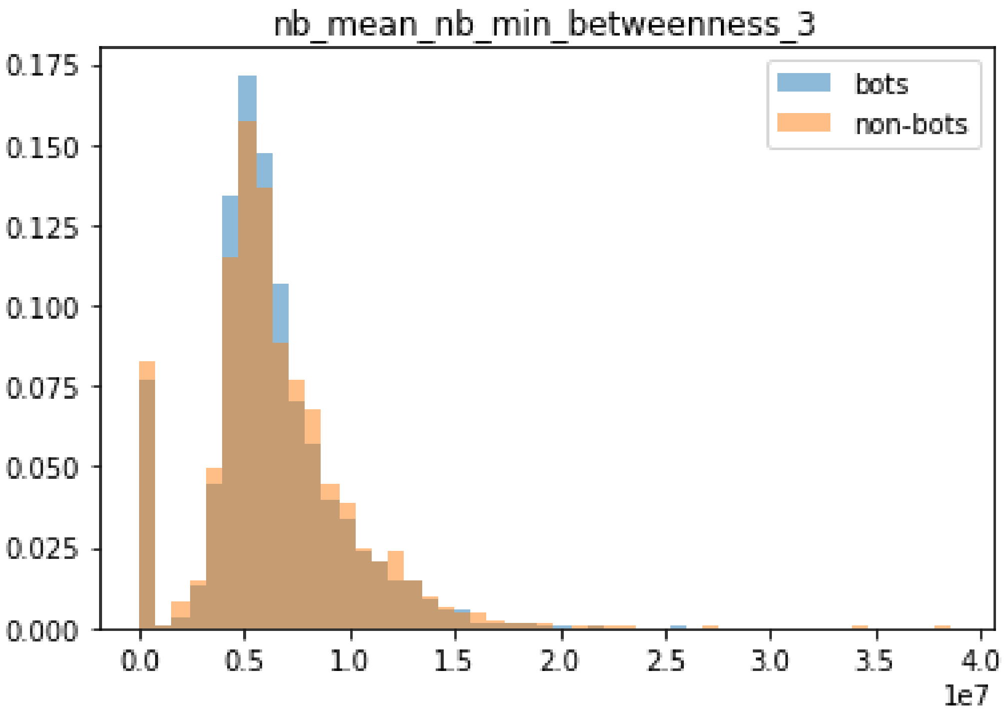
nb_mean_nb_min_betweenness_3
bots
non-bots
0.175
0.150
0.125
0.100
0.075
0.050
0.025
0.000
0.0
0.5
1.0
1.5
2.0
2.5
3.0
3.5
4.0
1e7

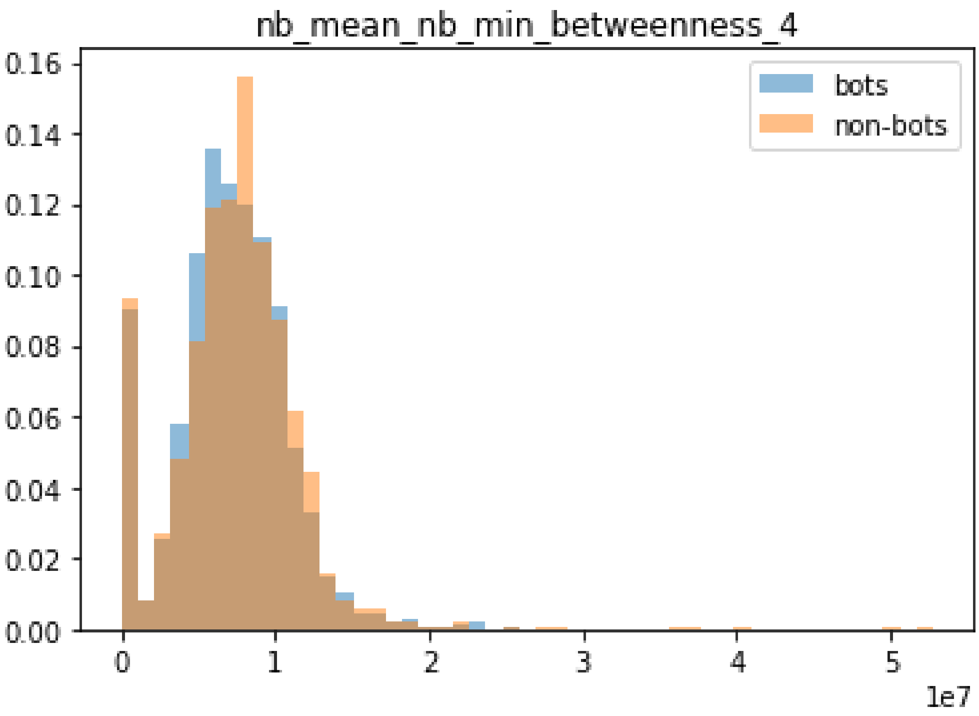
nb_mean_nb_min_betweenness_4
bots
non-bots
0.16
0.14
0.12
0.10
0.08
0.06
0.04
0.02
0.00
0
1
2
3
4
5
1e7

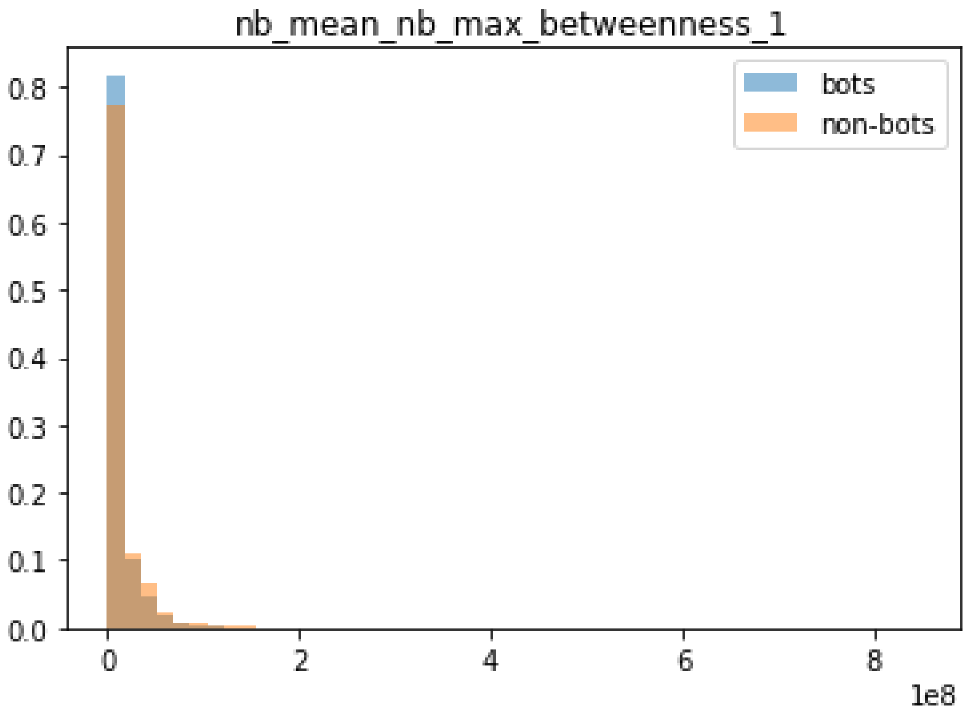
nb_mean_nb_max_betweenness_1
bots
non-bots
0.8
0.7
0.6
0.5
0.4
0.3
0.2
0.1
0.0
0
2
4
6
8
1e8

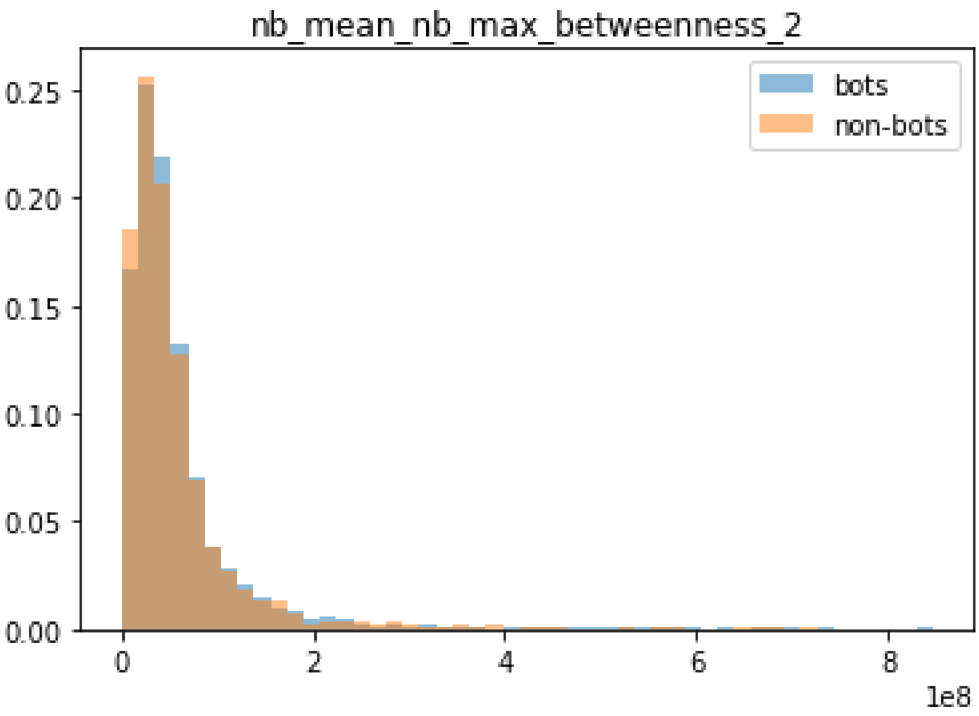
nb_mean_nb_max_betweenness_2
bots
non-bots
0.25
0.20
0.15
0.10
0.05
0.00
0
2
4
6
8
1e8

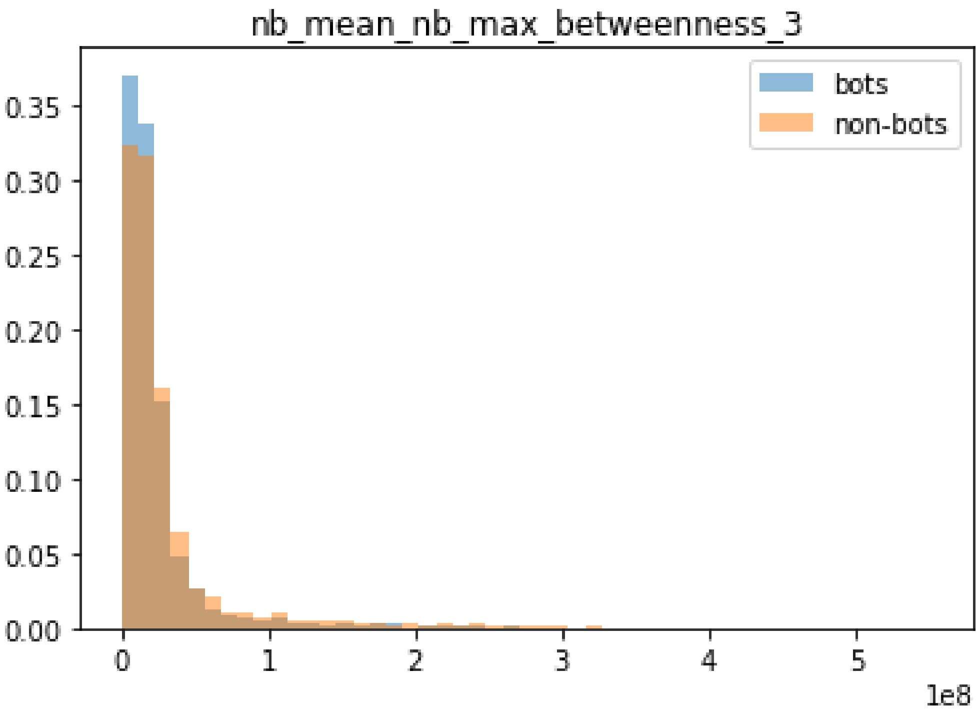
nb_mean_nb_max_betweenness_3
bots
non-bots
0.35
0.30
0.25
0.20
0.15
0.10
0.05
0.00
0
1
2
3
4
5
1e8

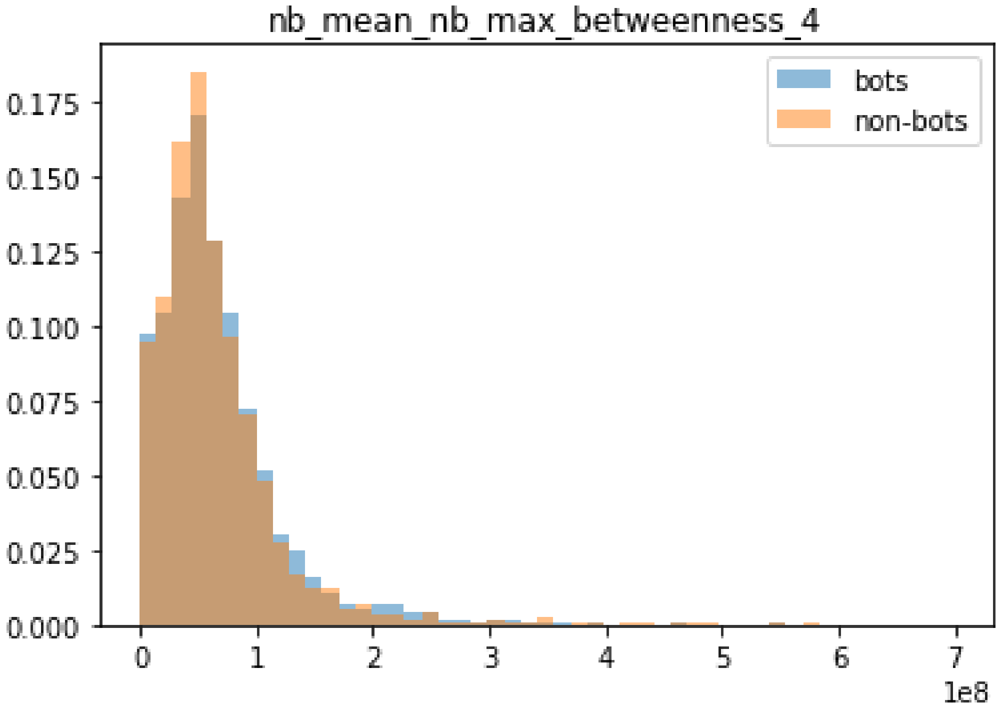
nb_mean_nb_max_betweenness_4
bots
non-bots
0.175
0.150
0.125
0.100
0.075
0.050
0.025
0.000
0
1
2
3
4
5
6
7
1e8

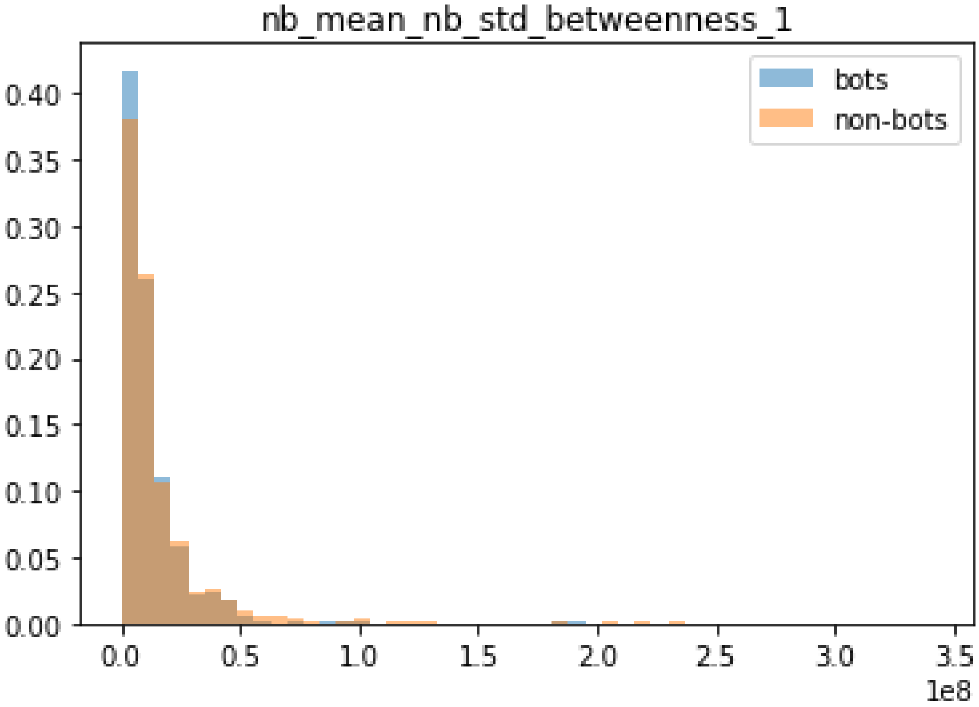
nb_mean_nb_std_betweenness_1
bots
non-bots
0.40
0.35
0.30
0.25
0.20
0.15
0.10
0.05
0.00
0.0
0.5
1.0
1.5
2.0
2.5
3.0
3.5
1e8

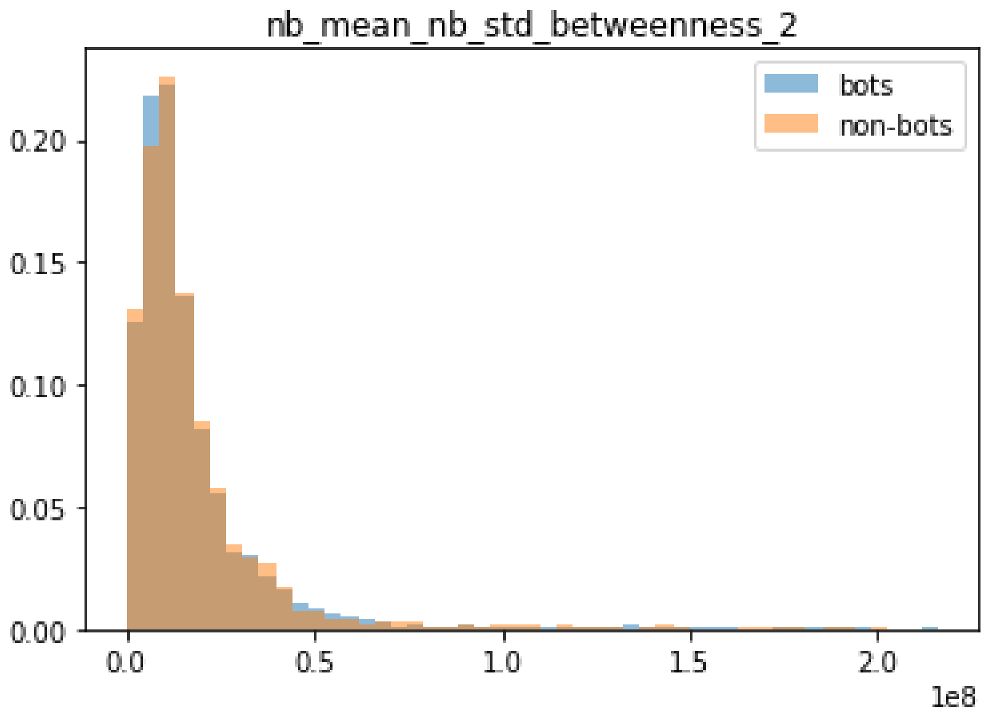
nb_mean_nb_std_betweenness_2
bots
non-bots
0.20
0.15
0.10
0.05
0.00
0.0
0.5
1.0
1.5
2.0
1e8

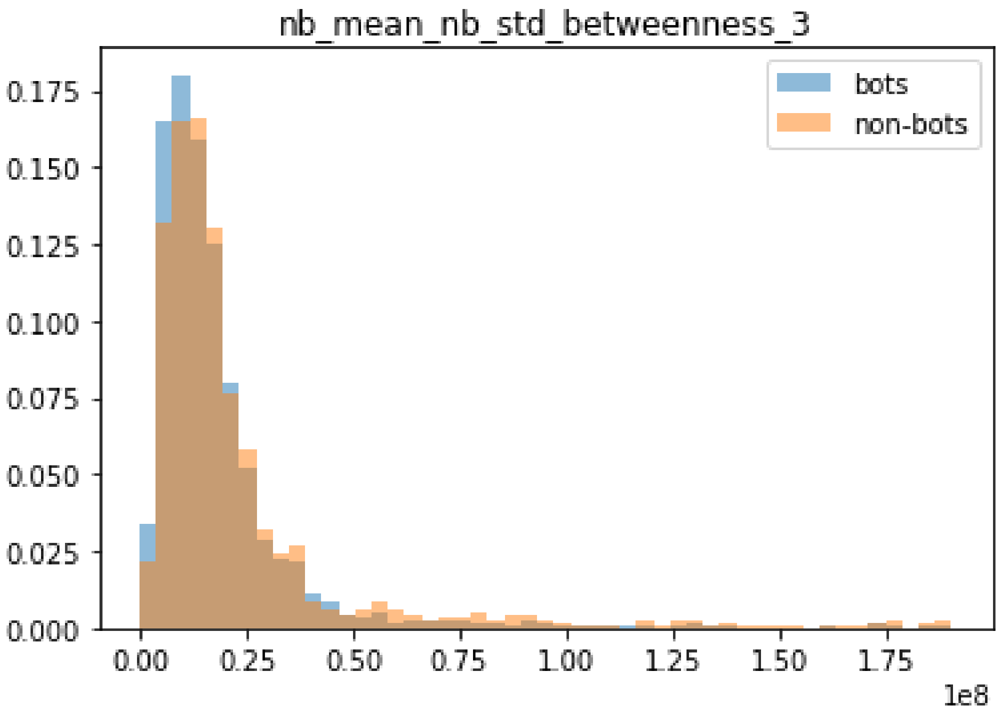
nb_mean_nb_std_betweenness_3
bots
non-bots
0.175
0.150
0.125
0.100
0.075
0.050
0.025
0.000
0.00
0.25
0.50
0.75
1.00
1.25
1.50
1.75
1e8

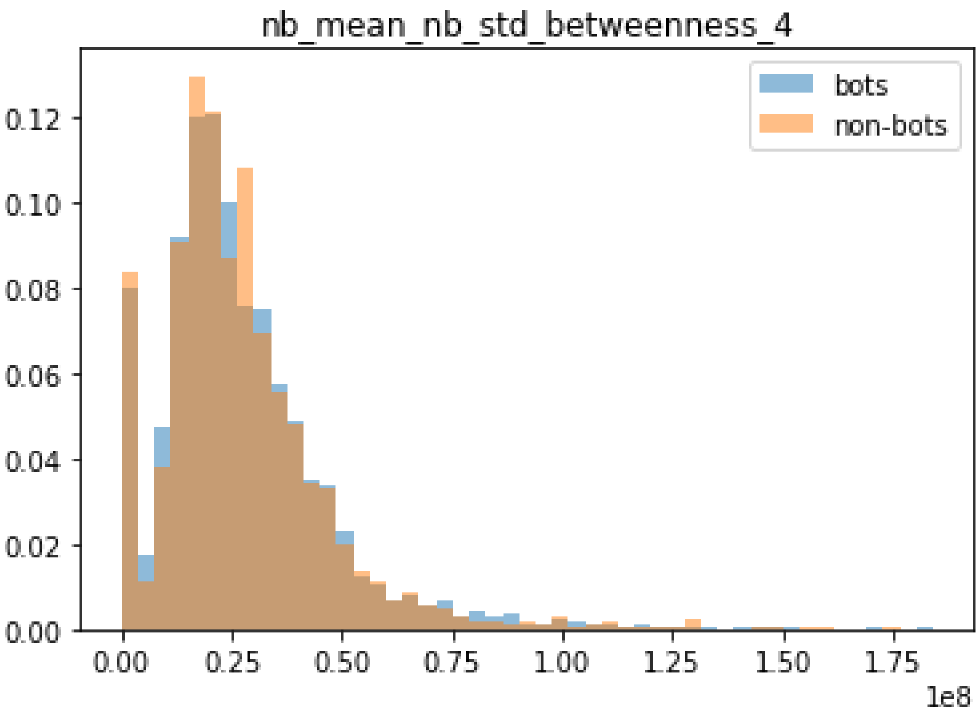
nb_mean_nb_std_betweenness_4
bots
non-bots
0.12
0.10
0.08
0.06
0.04
0.02
0.00
0.00
0.25
0.50
0.75
1.00
1.25
1.50
1.75
1e8

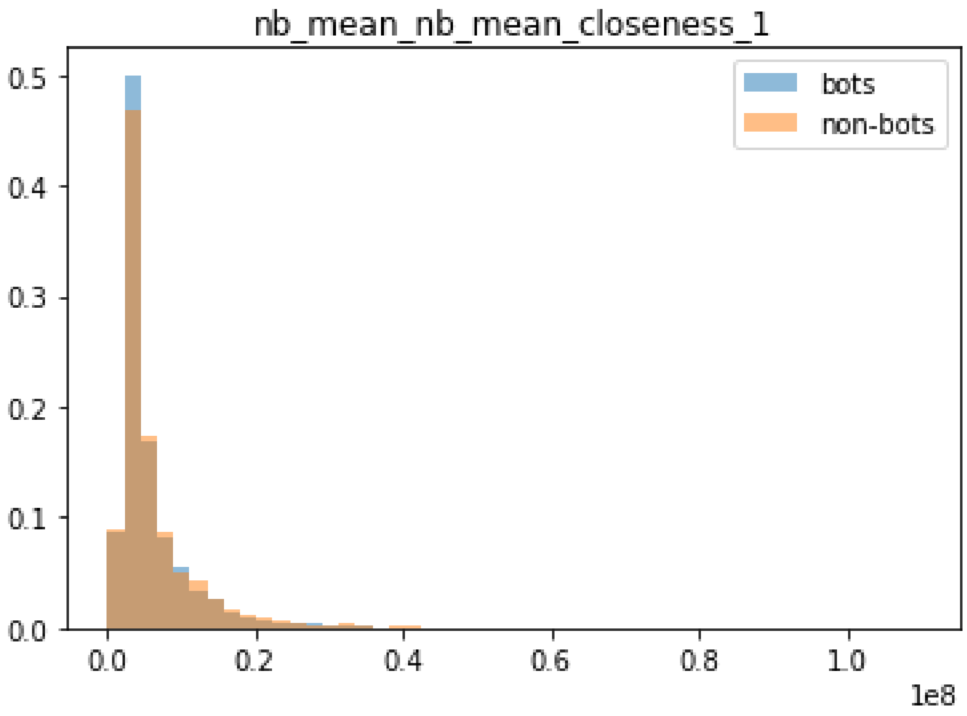
nb_mean_nb_mean_closeness_1
bots
non-bots
0.5
0.4
0.3
0.2
0.1
0.0
0.0
0.2
0.4
0.6
0.8
1.0
1e8

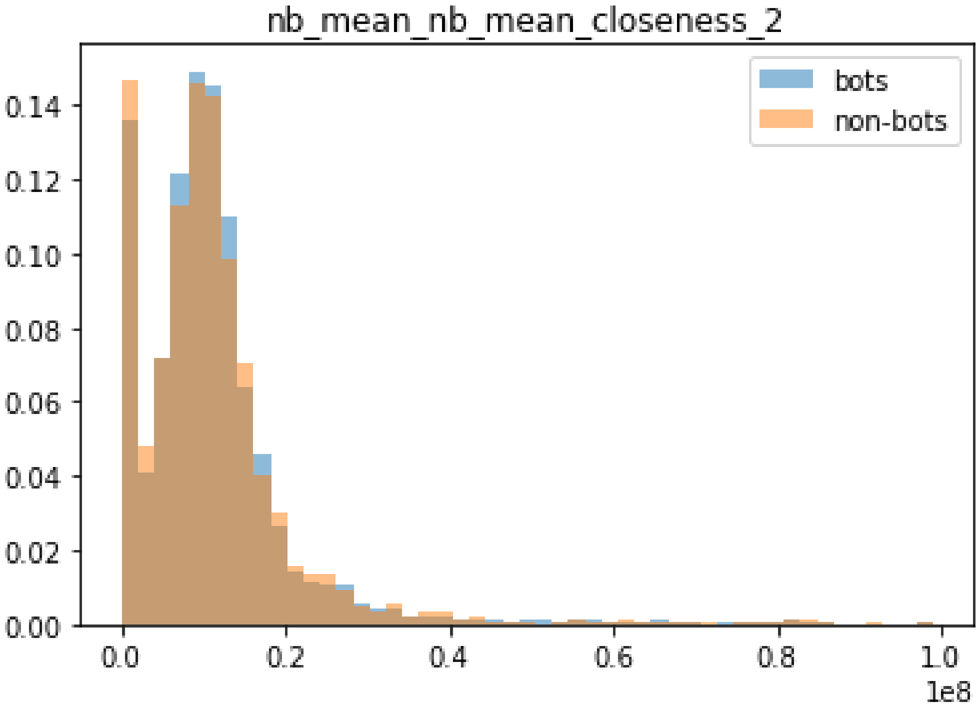
nb_mean_nb_mean_closeness_2
bots
non-bots
0.00
0.02
0.04
0.06
0.08
0.10
0.12
0.14
0.0
0.2
0.4
0.6
0.8
1.0
1e8

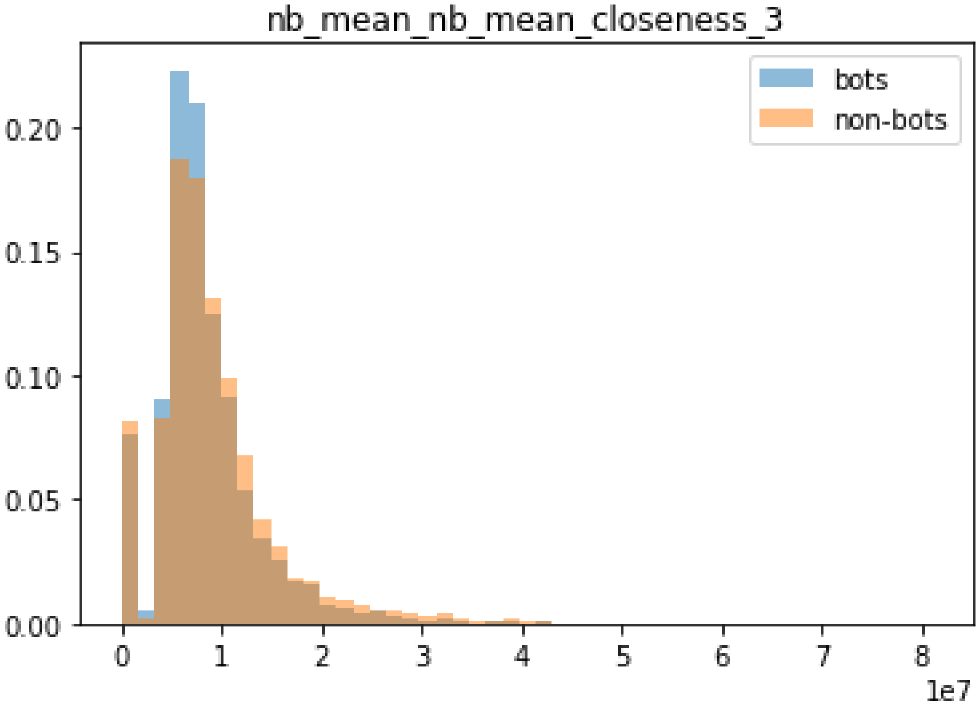
nb_mean_nb_mean_closeness_3
bots
non-bots
0.00
0.05
0.10
0.15
0.20
0
1
2
3
4
5
6
7
8
1e7

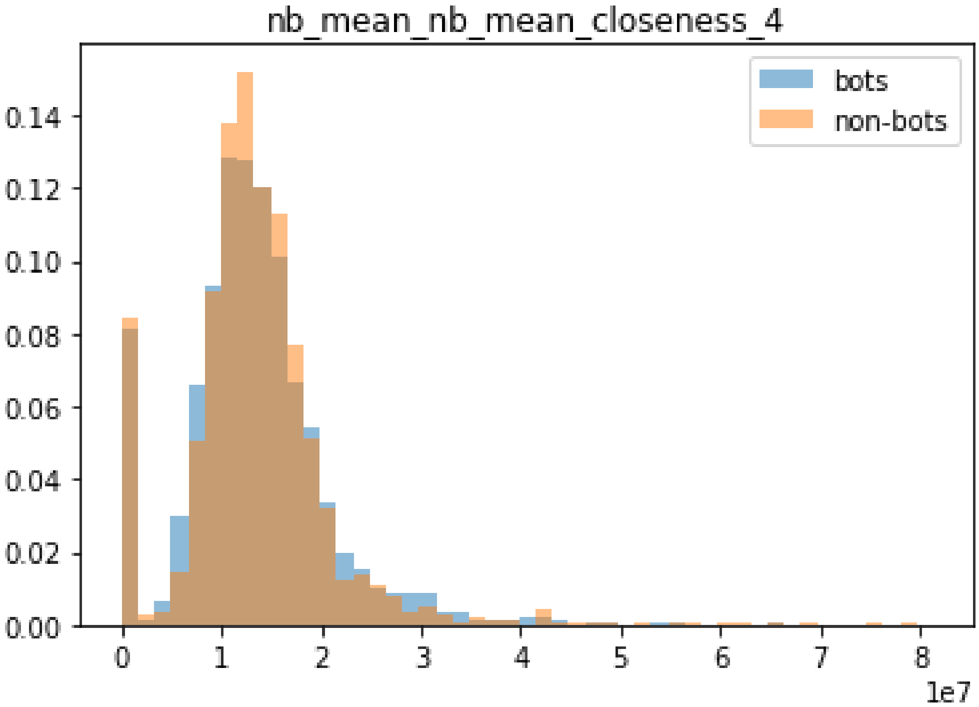
nb_mean_nb_mean_closeness_4
bots
non-bots
0.14
0.12
0.10
0.08
0.06
0.04
0.02
0.00
0
1
2
3
4
5
6
7
8
1e7

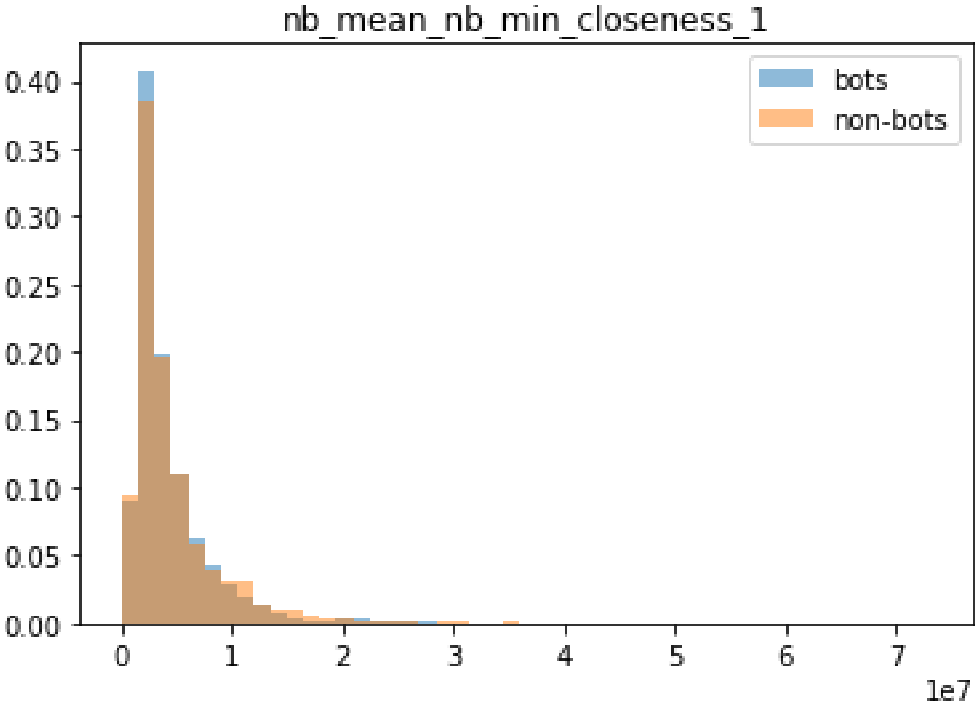
nb_mean_nb_min_closeness_1
bots
non-bots
0.40
0.35
0.30
0.25
0.20
0.15
0.10
0.05
0.00
0
1
2
3
4
5
6
7
1e7

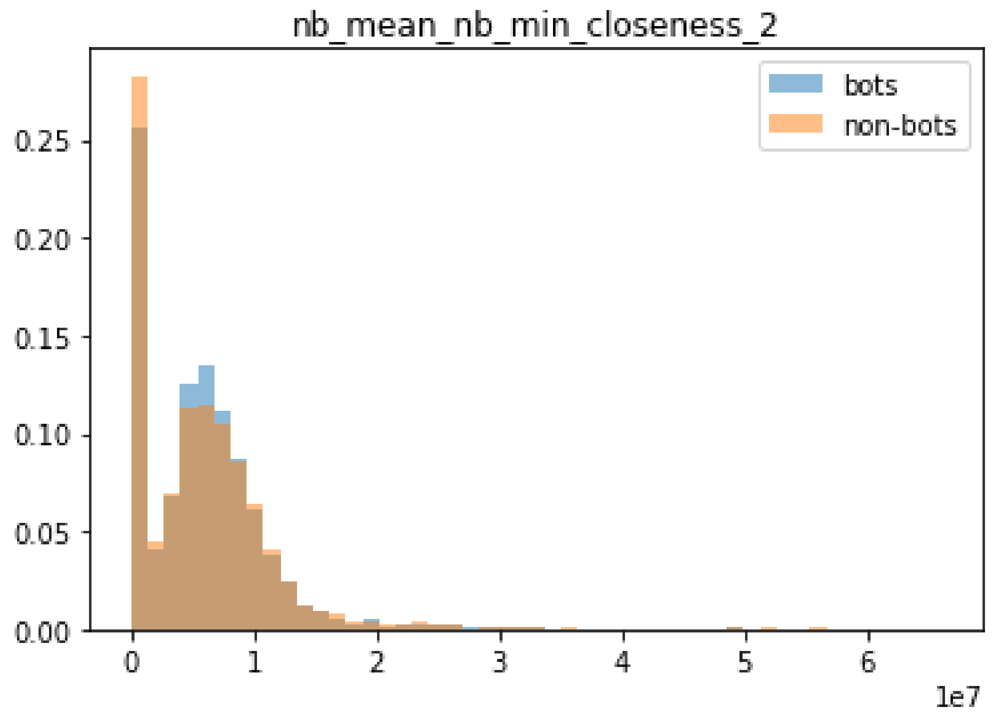

nb_mean_nb_min_closeness_2
bots
non-bots
0.25
0.20
0.15
0.10
0.05
0.00
0
1
2
3
4
5
6
1e7

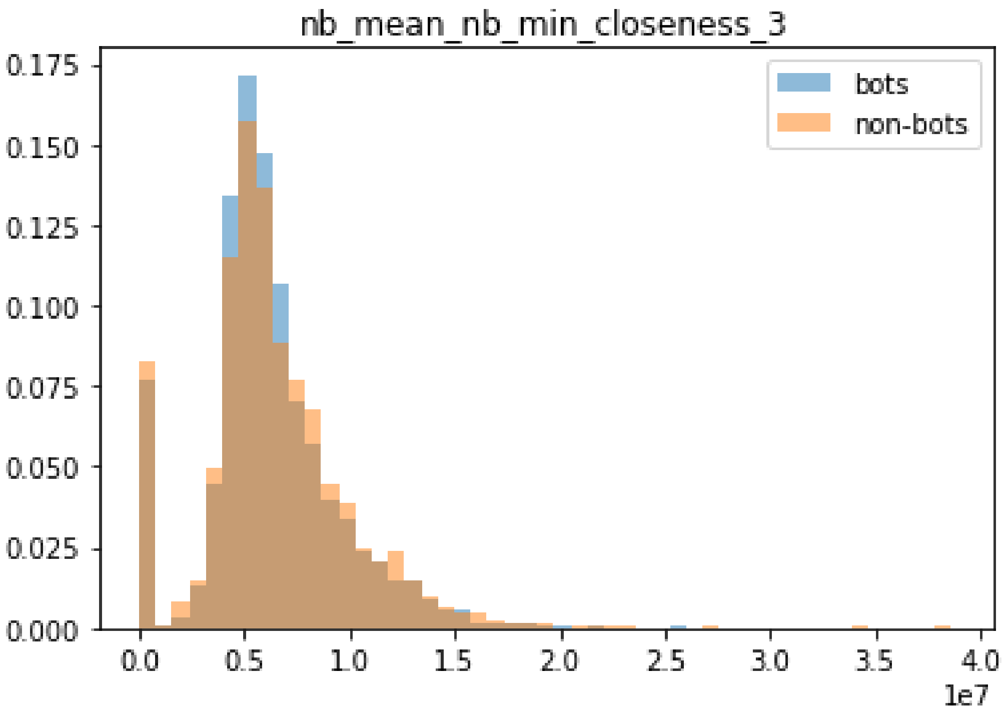

nb_mean_nb_min_closeness_3
bots
non-bots
0.175
0.150
0.125
0.100
0.075
0.050
0.025
0.000
0.0
0.5
1.0
1.5
2.0
2.5
3.0
3.5
4.0
1e7

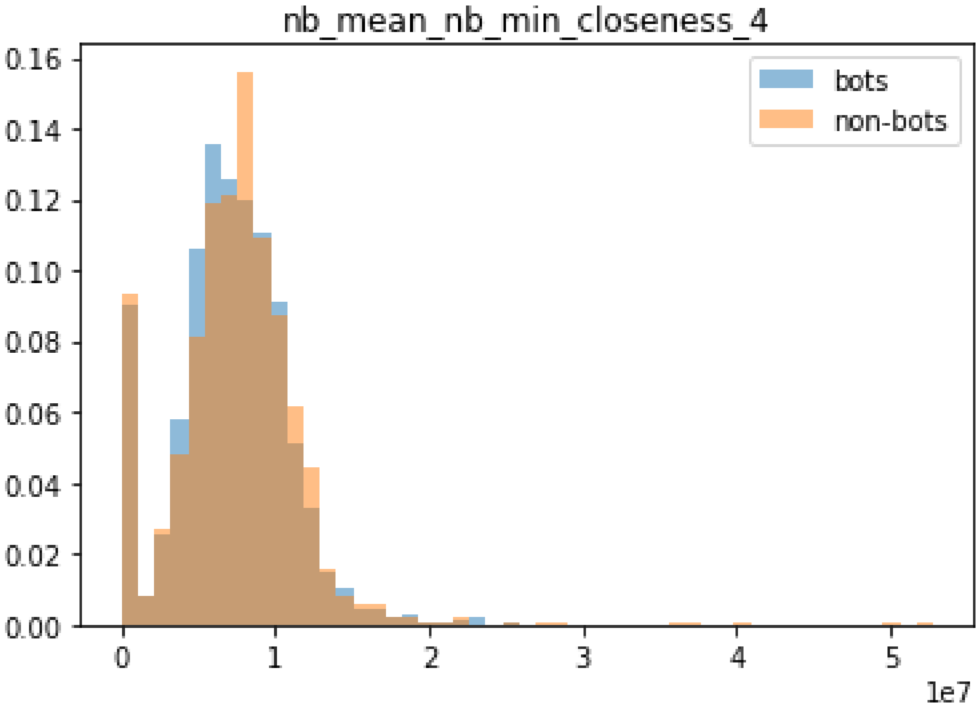
nb_mean_nb_min_closeness_4
bots
non-bots
0.16
0.14
0.12
0.10
0.08
0.06
0.04
0.02
0.00
0
1
2
3
4
5
1e7

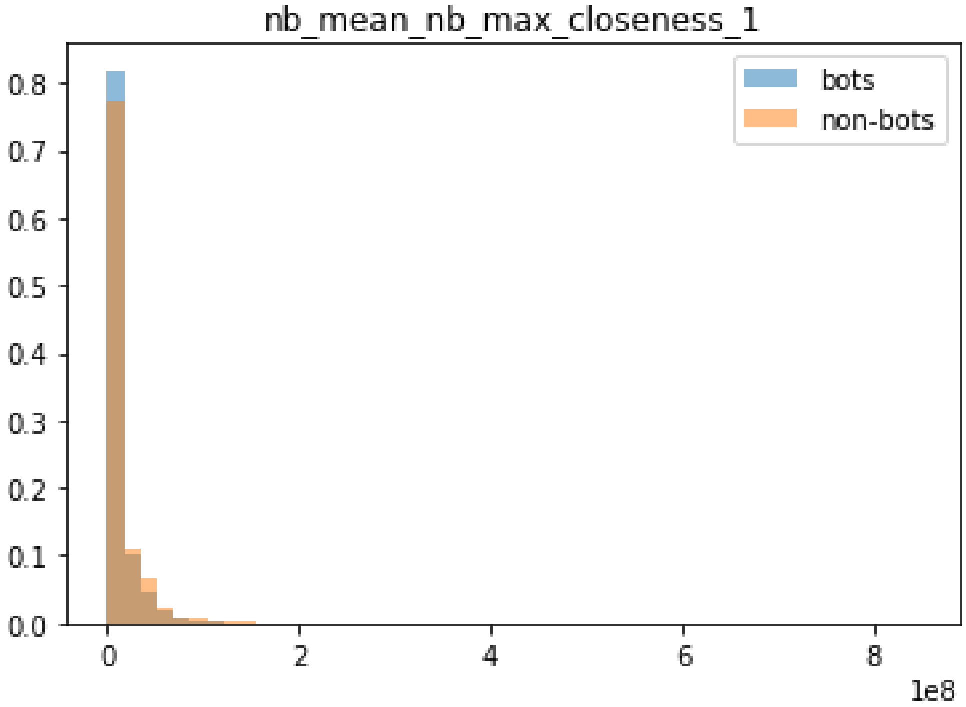
nb_mean_nb_max_closeness_1
bots
non-bots
0.8
0.7
0.6
0.5
0.4
0.3
0.2
0.1
0.0
0
2
4
6
8
1e8

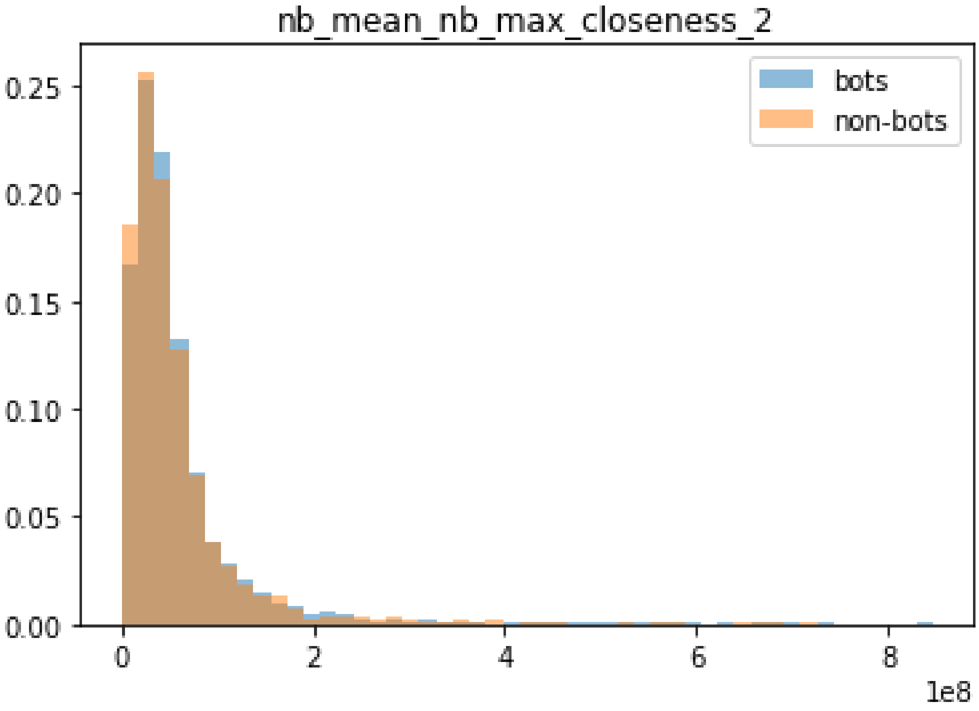
nb_mean_nb_max_closeness_2
0.25
0.20
0.15
0.10
0.05
0.00
0
2
4
6
8
1e8
bots
non-bots

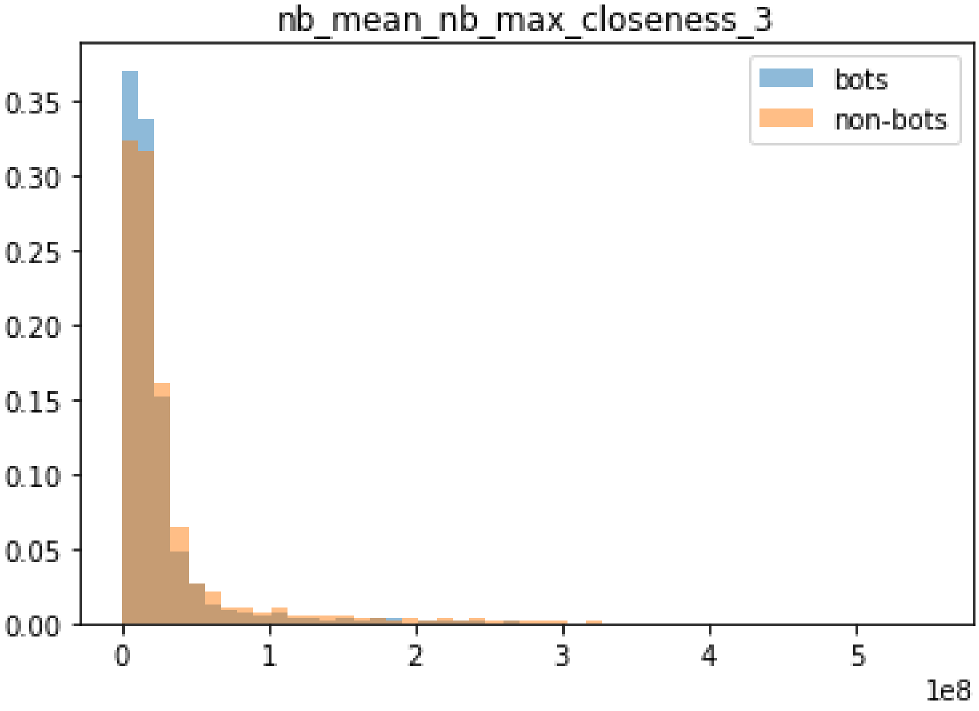
nb_mean_nb_max_closeness_3
0.35
0.30
0.25
0.20
0.15
0.10
0.05
0.00
0
1
2
3
4
5
1e8
bots
non-bots

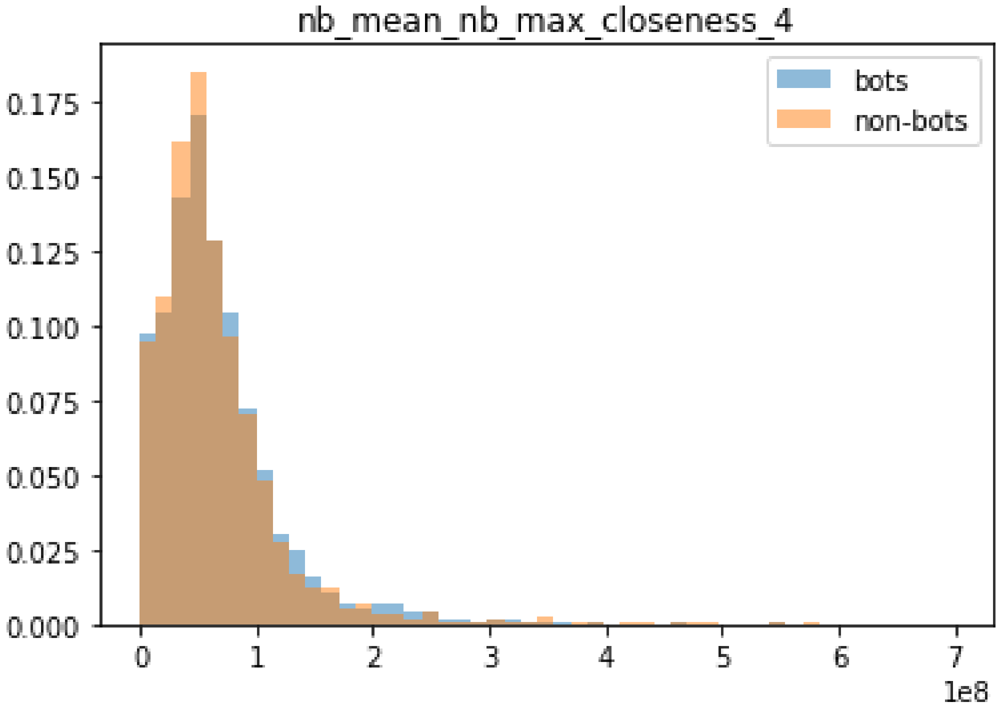
nb_mean_nb_max_closeness_4
bots
non-bots
0.000
0.025
0.050
0.075
0.100
0.125
0.150
0.175
0
1
2
3
4
5
6
7
1e8

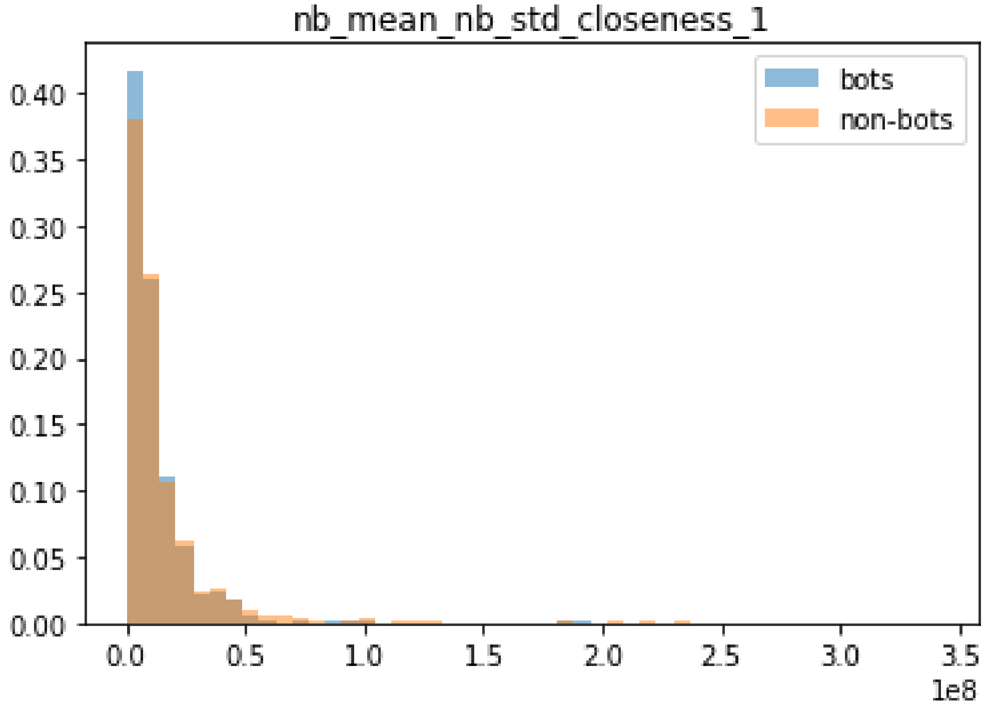
nb_mean_nb_std_closeness_1
bots
non-bots
0.00
0.05
0.10
0.15
0.20
0.25
0.30
0.35
0.40
0.0
0.5
1.0
1.5
2.0
2.5
3.0
3.5
1e8

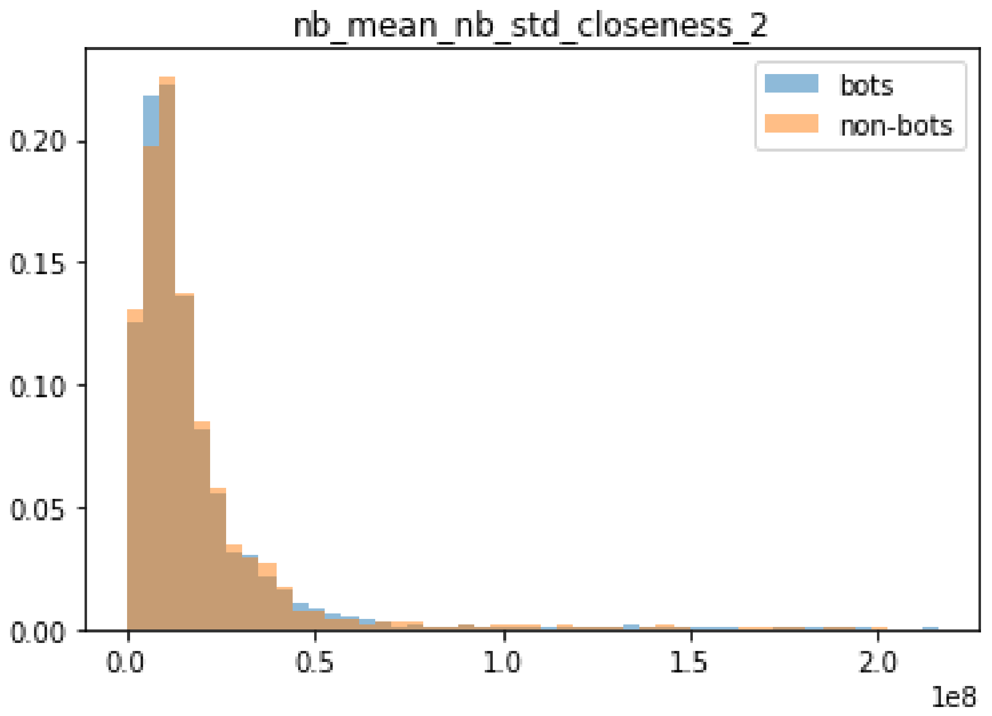
nb_mean_nb_std_closeness_2
bots
non-bots
0.20
0.15
0.10
0.05
0.00
0.0
0.5
1.0
1.5
2.0
1e8

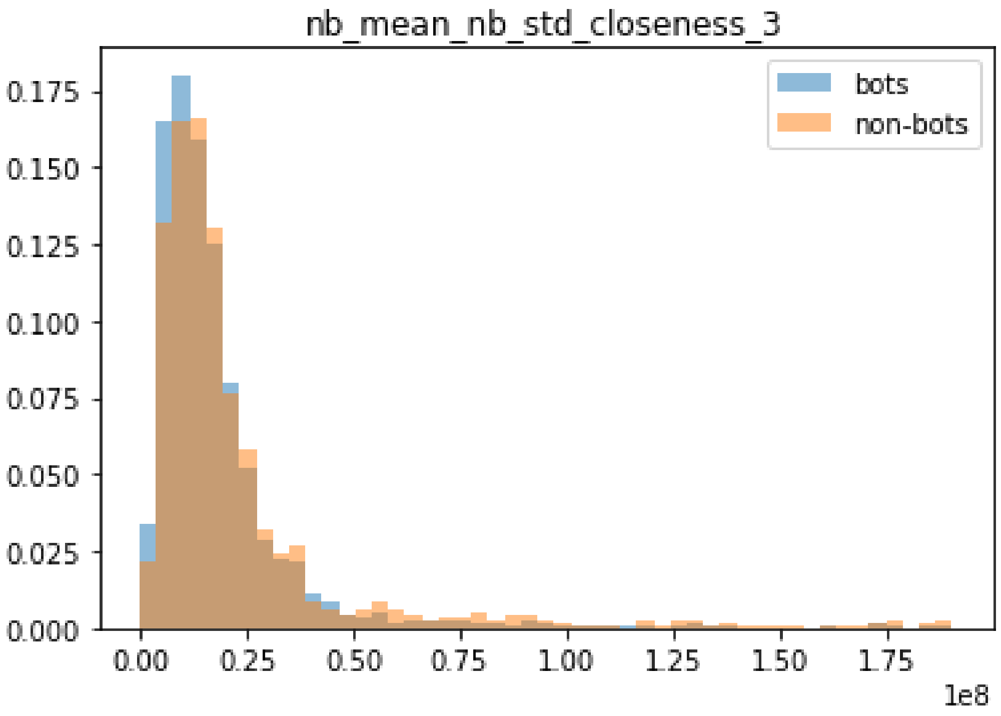
nb_mean_nb_std_closeness_3
bots
non-bots
0.175
0.150
0.125
0.100
0.075
0.050
0.025
0.000
0.00
0.25
0.50
0.75
1.00
1.25
1.50
1.75
1e8

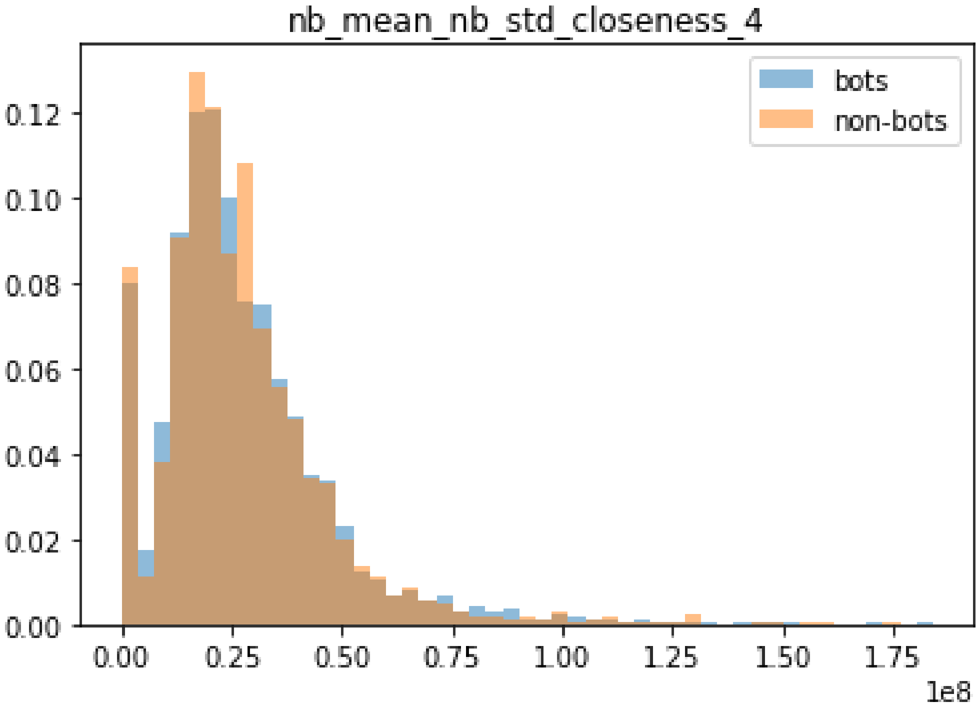
nb_mean_nb_std_closeness_4
bots
non-bots
0.12
0.10
0.08
0.06
0.04
0.02
0.00
0.00
0.25
0.50
0.75
1.00
1.25
1.50
1.75
1e8

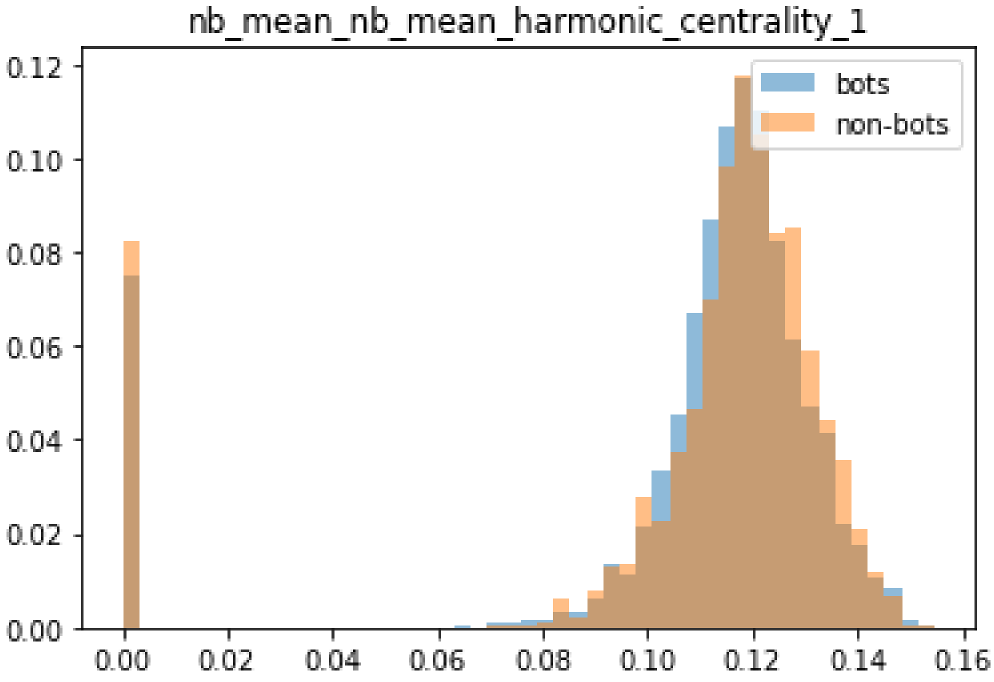
nb_mean_nb_mean_harmonic_centrality_1
bots
non-bots
0.12
0.10
0.08
0.06
0.04
0.02
0.00
0.00
0.02
0.04
0.06
0.08
0.10
0.12
0.14
0.16

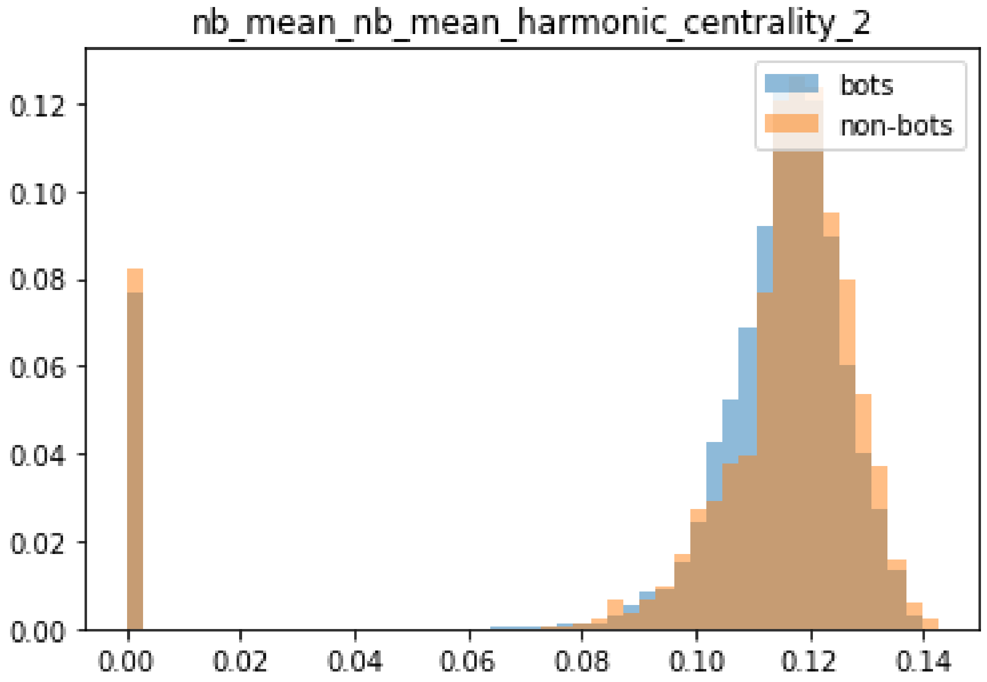
nb_mean_nb_mean_harmonic_centrality_2
bots
non-bots
0.00
0.02
0.04
0.06
0.08
0.10
0.12
0.14

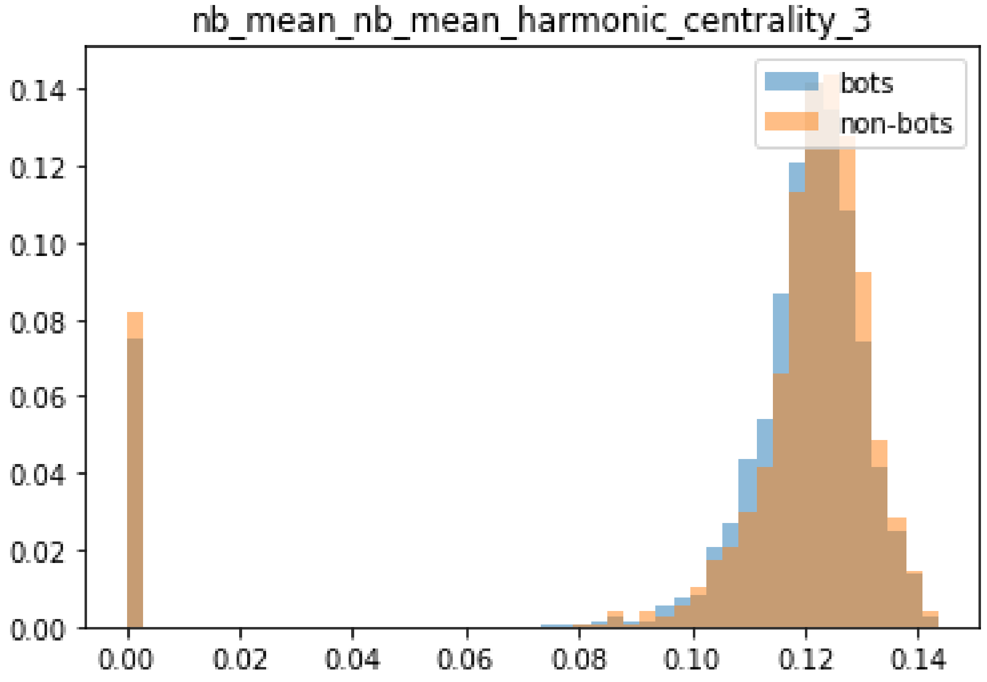
nb_mean_nb_mean_harmonic_centrality_3
bots
non-bots
0.00
0.02
0.04
0.06
0.08
0.10
0.12
0.14

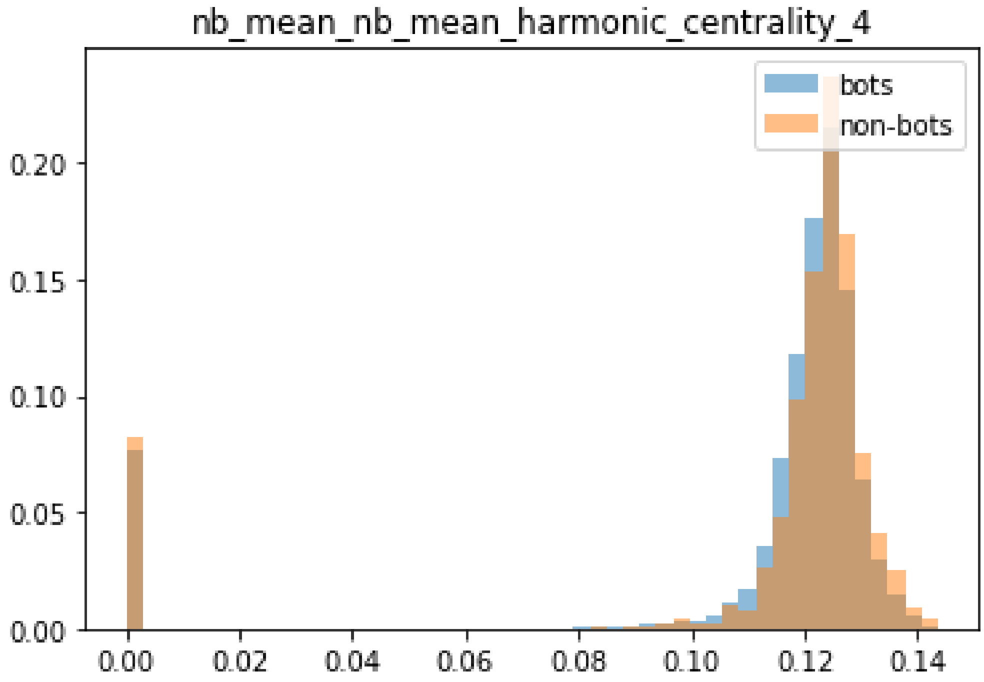
nb_mean_nb_mean_harmonic_centrality_4
bots
non-bots
0.20
0.15
0.10
0.05
0.00
0.00
0.02
0.04
0.06
0.08
0.10
0.12
0.14

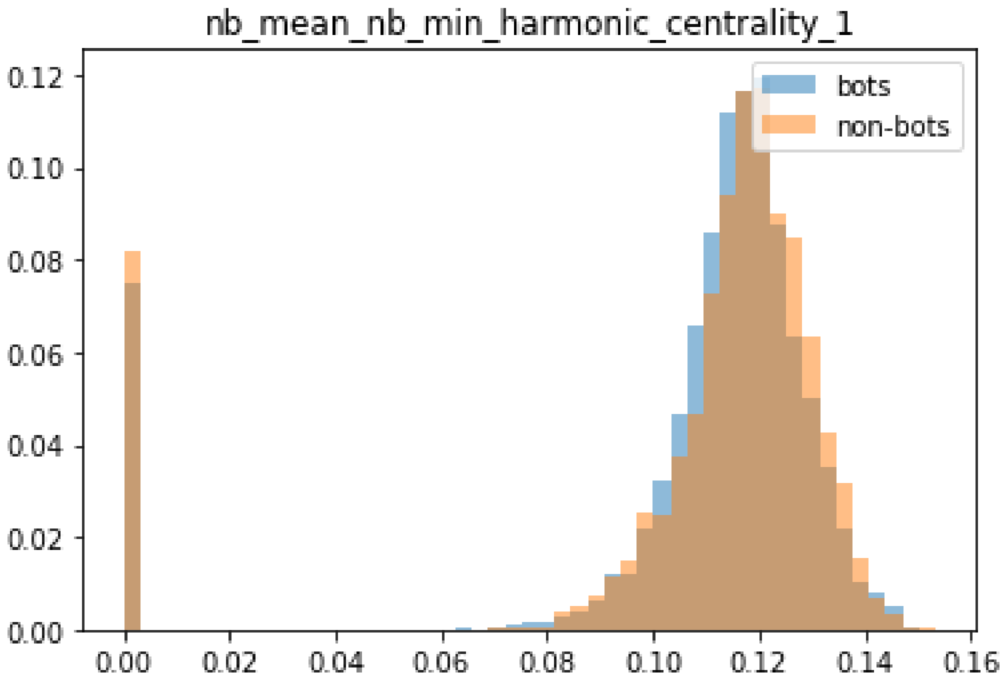
nb_mean_nb_min_harmonic_centrality_1
bots
non-bots
0.12
0.10
0.08
0.06
0.04
0.02
0.00
0.00
0.02
0.04
0.06
0.08
0.10
0.12
0.14
0.16

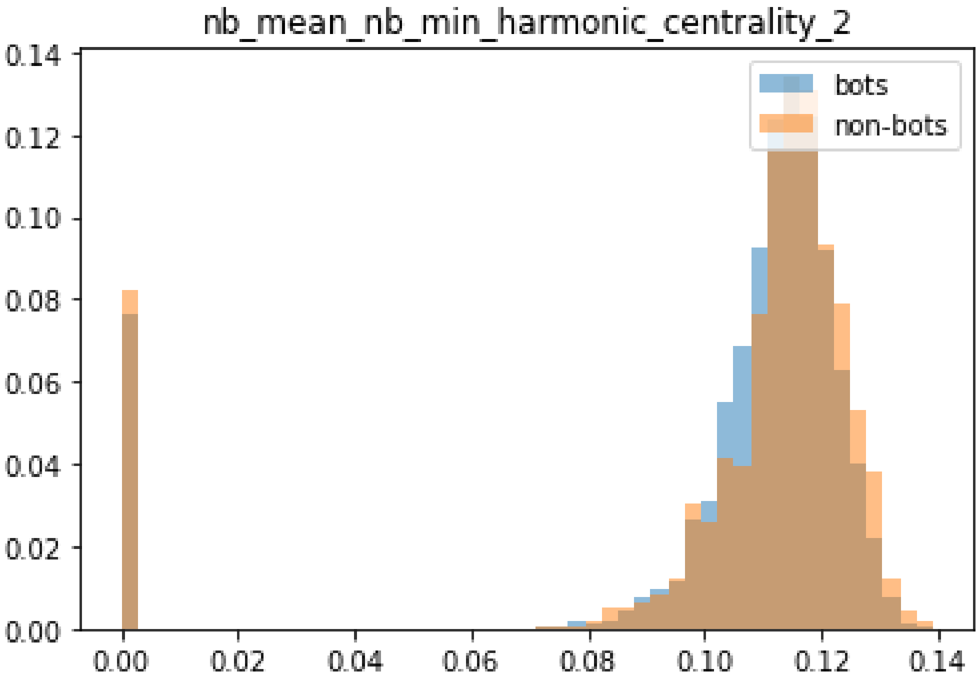
nb_mean_nb_min_harmonic_centrality_2
bots
non-bots
0.00
0.02
0.04
0.06
0.08
0.10
0.12
0.14

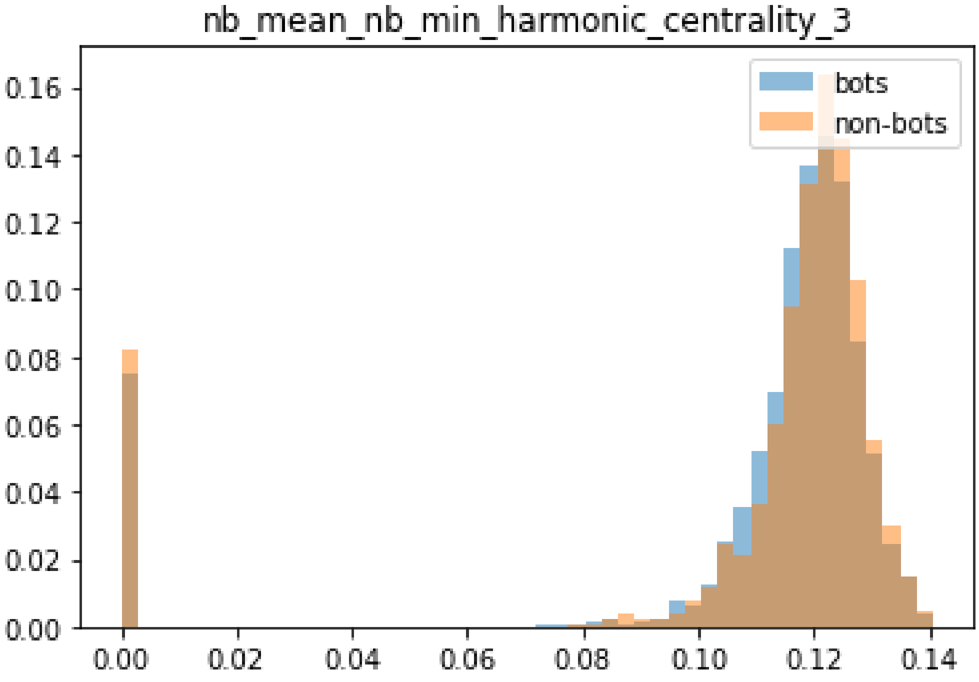
nb_mean_nb_min_harmonic_centrality_3
bots
non-bots
0.00
0.02
0.04
0.06
0.08
0.10
0.12
0.14
0.16

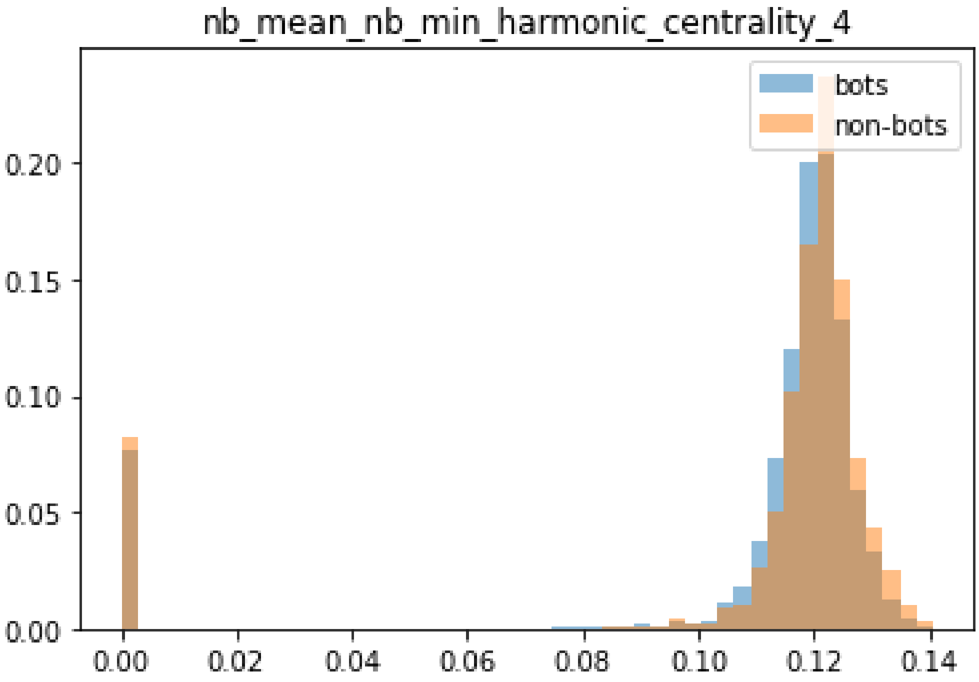
nb_mean_nb_min_harmonic_centrality_4
bots
non-bots
0.20
0.15
0.10
0.05
0.00
0.00
0.02
0.04
0.06
0.08
0.10
0.12
0.14

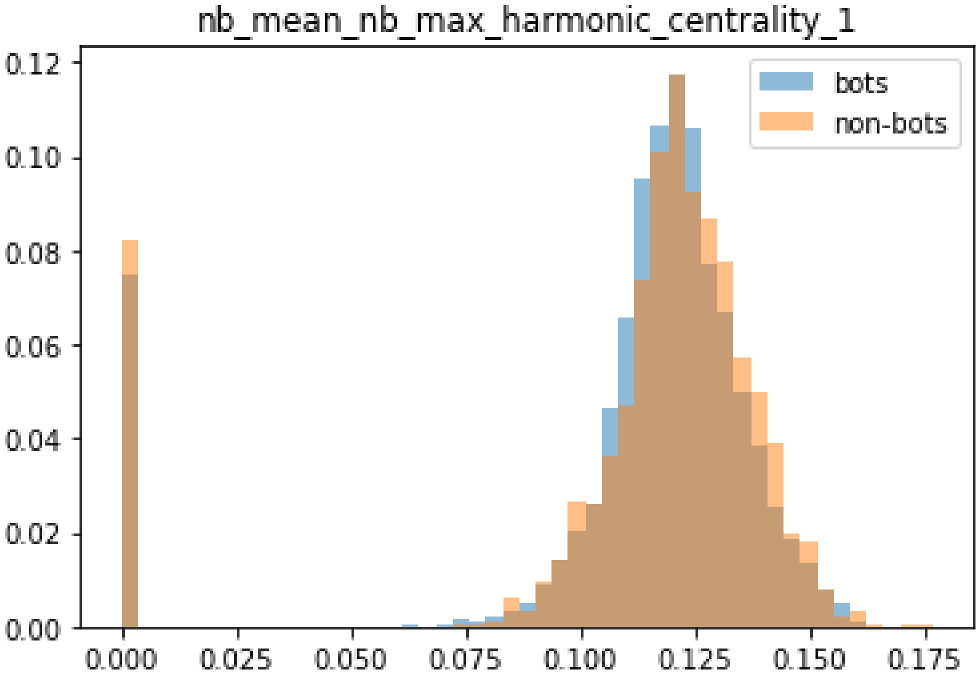
nb_mean_nb_max_harmonic_centrality_1
bots
non-bots
0.12
0.10
0.08
0.06
0.04
0.02
0.00
0.000
0.025
0.050
0.075
0.100
0.125
0.150
0.175

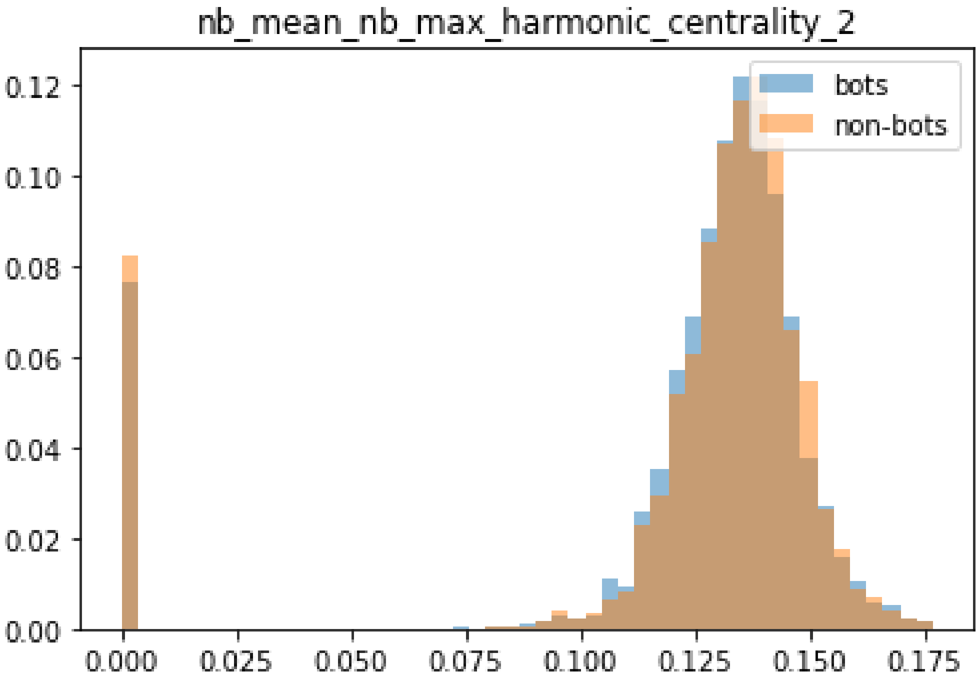
nb_mean_nb_max_harmonic_centrality_2
bots
non-bots
0.12
0.10
0.08
0.06
0.04
0.02
0.00
0.000
0.025
0.050
0.075
0.100
0.125
0.150
0.175

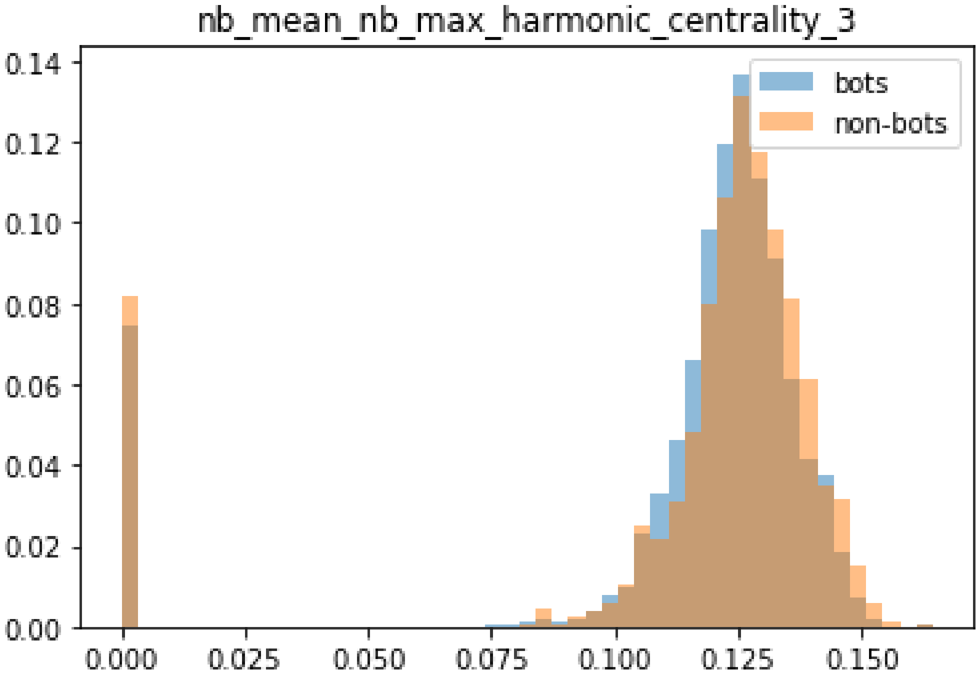
nb_mean_nb_max_harmonic_centrality_3
bots
non-bots
0.14
0.12
0.10
0.08
0.06
0.04
0.02
0.00
0.000
0.025
0.050
0.075
0.100
0.125
0.150

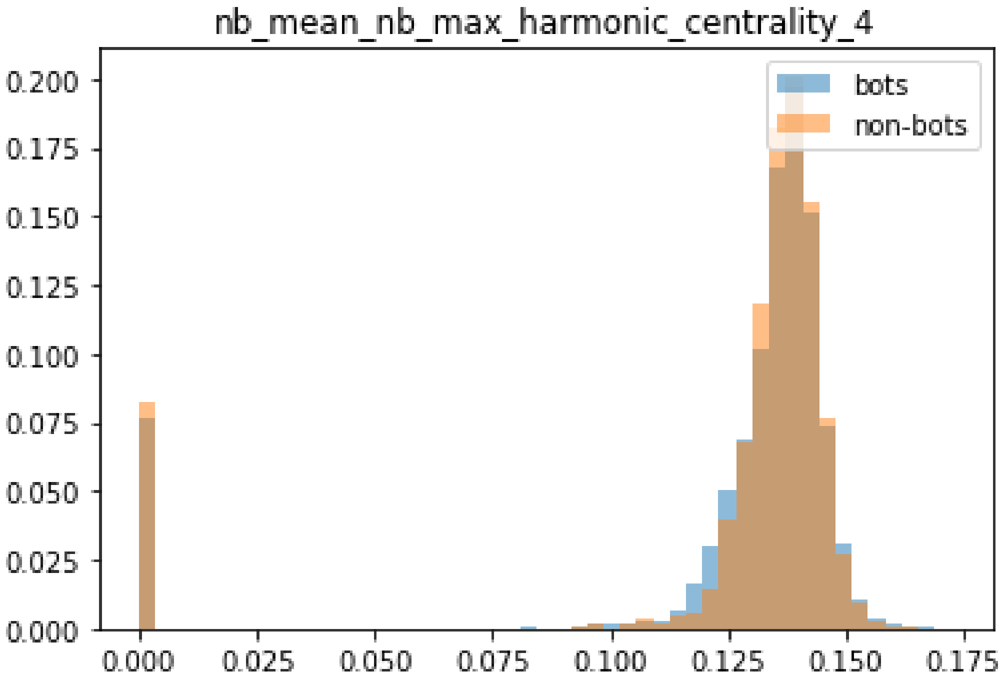
nb_mean_nb_max_harmonic_centrality_4
bots
non-bots
0.200
0.175
0.150
0.125
0.100
0.075
0.050
0.025
0.000
0.000
0.025
0.050
0.075
0.100
0.125
0.150
0.175

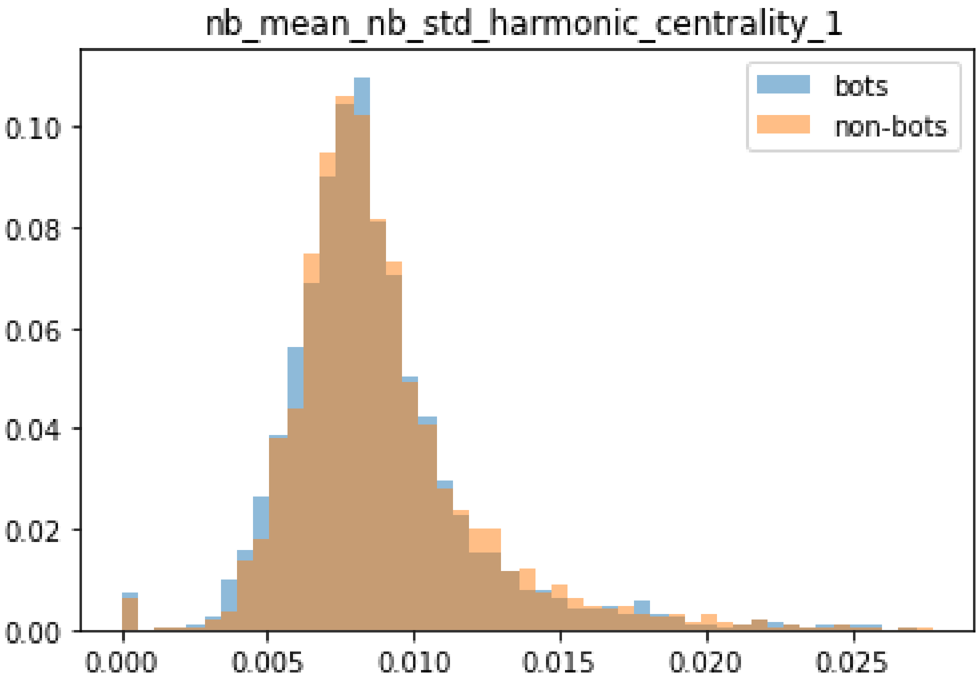
nb_mean_nb_std_harmonic_centrality_1
bots
non-bots
0.10
0.08
0.06
0.04
0.02
0.00
0.000
0.005
0.010
0.015
0.020
0.025

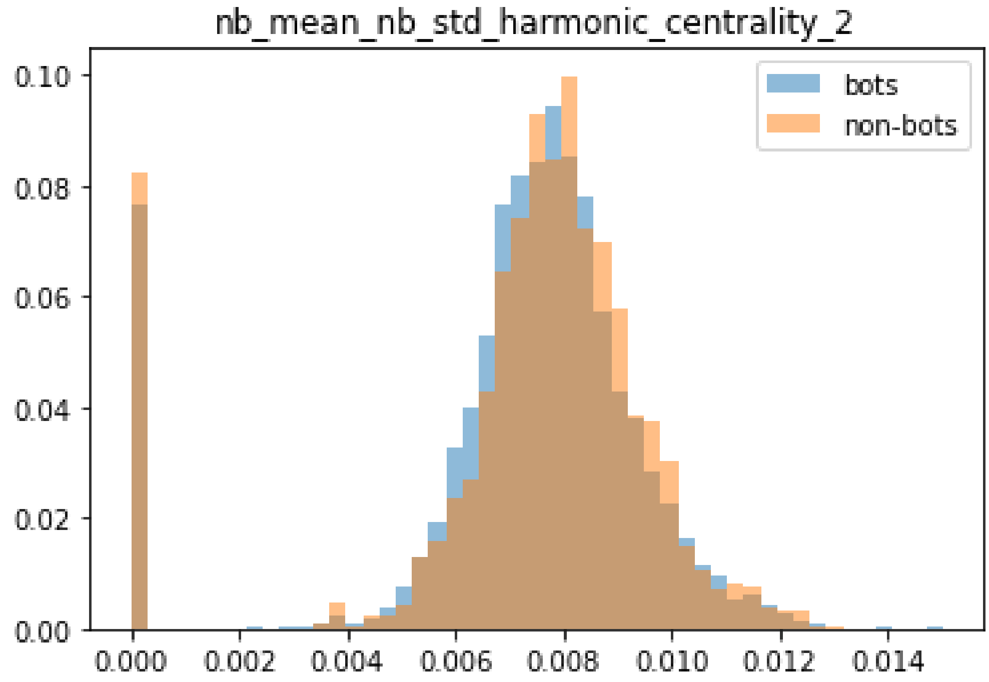
nb_mean_nb_std_harmonic_centrality_2
bots
non-bots
0.10
0.08
0.06
0.04
0.02
0.00
0.000
0.002
0.004
0.006
0.008
0.010
0.012
0.014

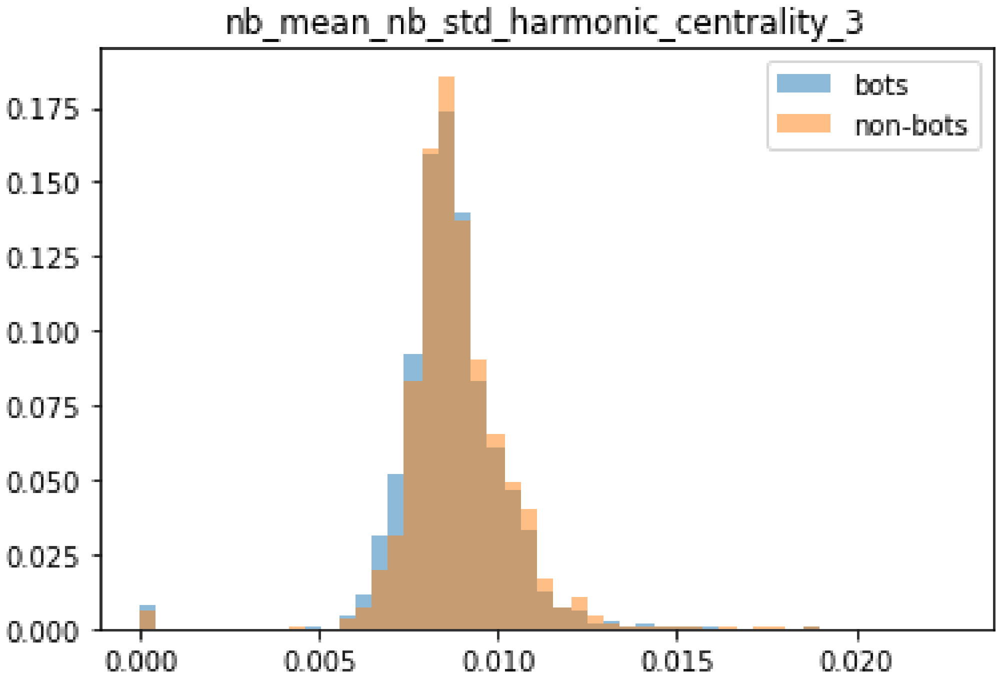
nb_mean_nb_std_harmonic_centrality_3
bots
non-bots
0.175
0.150
0.125
0.100
0.075
0.050
0.025
0.000
0.000
0.005
0.010
0.015
0.020

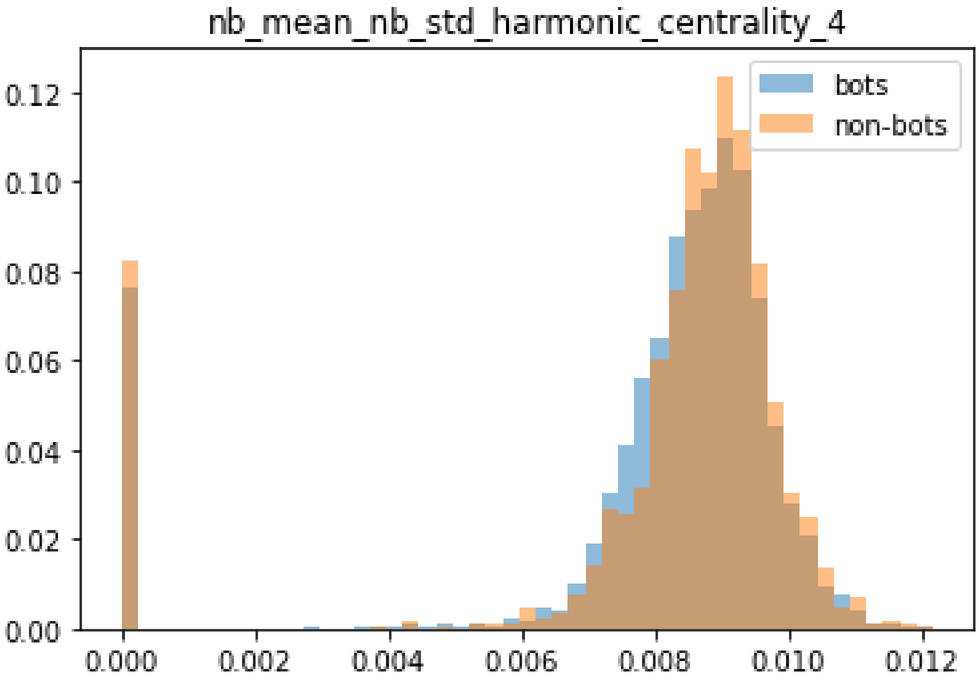
nb_mean_nb_std_harmonic_centrality_4
bots
non-bots
0.12
0.10
0.08
0.06
0.04
0.02
0.00
0.000
0.002
0.004
0.006
0.008
0.010
0.012

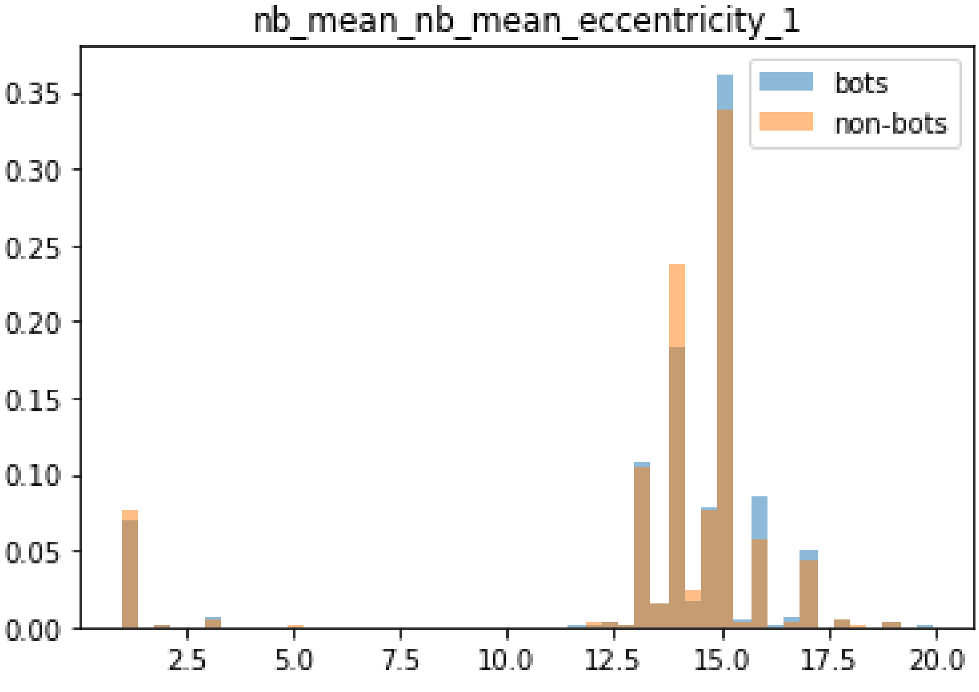
nb_mean_nb_mean_eccentricity_1
bots
non-bots
0.35
0.30
0.25
0.20
0.15
0.10
0.05
0.00
2.5
5.0
7.5
10.0
12.5
15.0
17.5
20.0

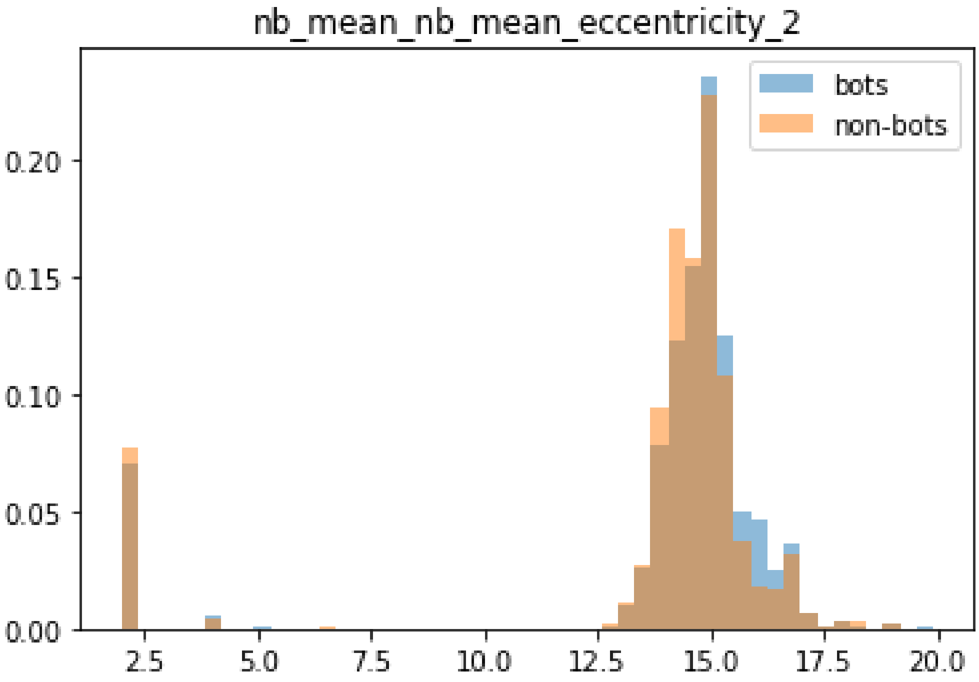
nb_mean_nb_mean_eccentricity_2
bots
non-bots
0.20
0.15
0.10
0.05
0.00
2.5
5.0
7.5
10.0
12.5
15.0
17.5
20.0

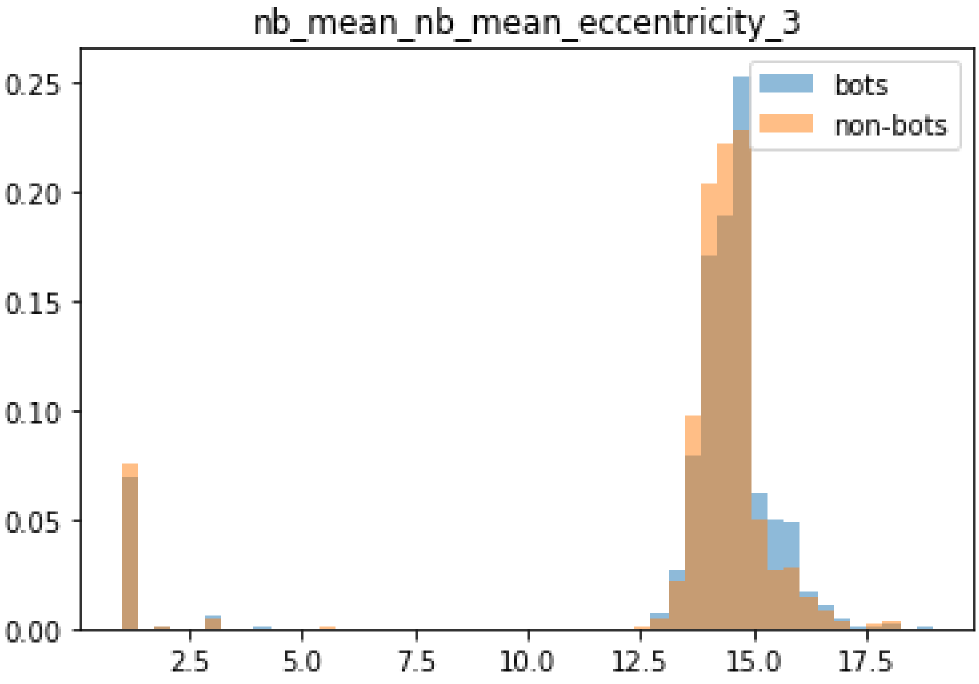
nb_mean_nb_mean_eccentricity_3
bots
non-bots
0.25
0.20
0.15
0.10
0.05
0.00
2.5
5.0
7.5
10.0
12.5
15.0
17.5

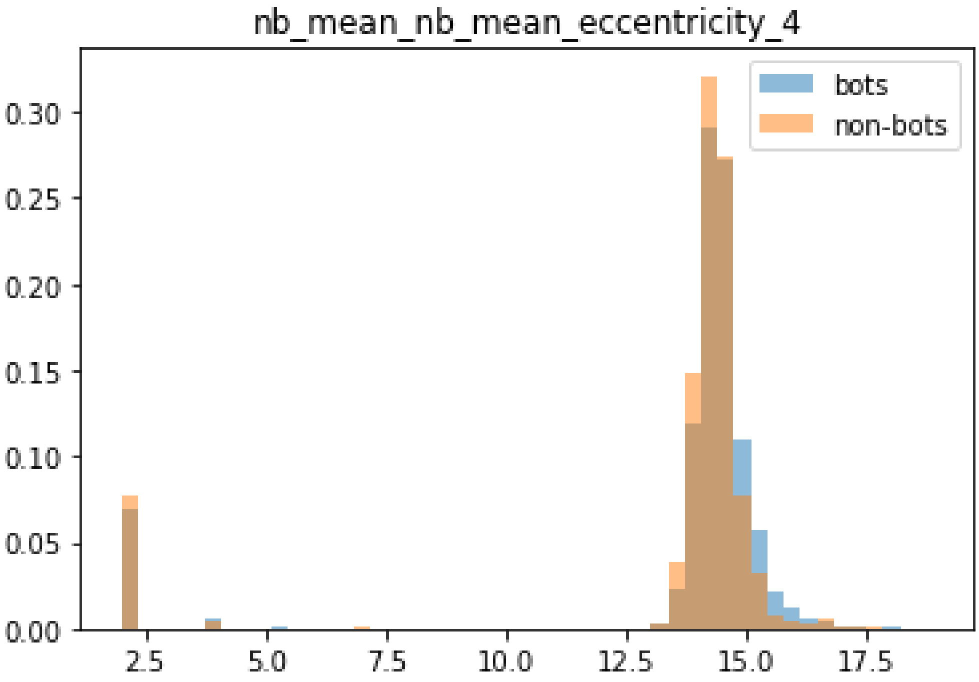
nb_mean_nb_mean_eccentricity_4
bots
non-bots
0.30
0.25
0.20
0.15
0.10
0.05
0.00
2.5
5.0
7.5
10.0
12.5
15.0
17.5

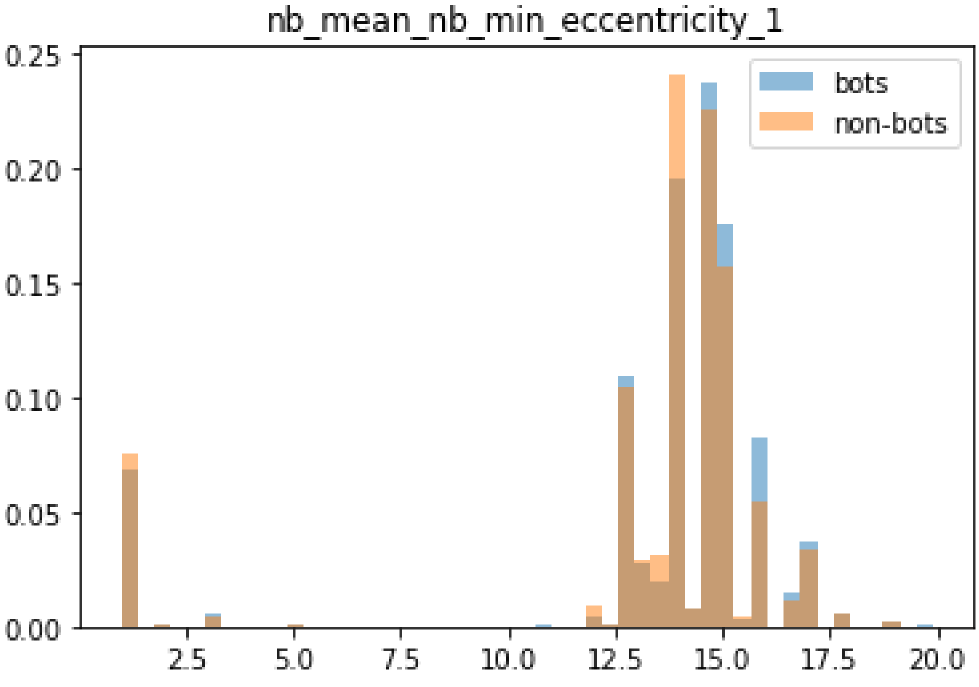
nb_mean_nb_min_eccentricity_1
bots
non-bots
0.25
0.20
0.15
0.10
0.05
0.00
2.5
5.0
7.5
10.0
12.5
15.0
17.5
20.0

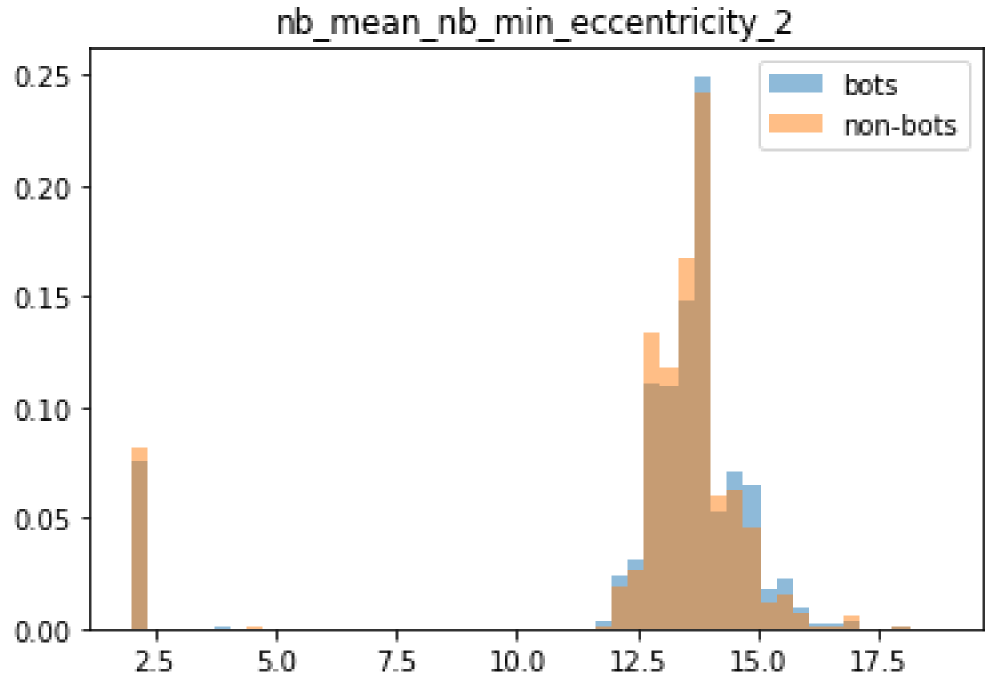
nb_mean_nb_min_eccentricity_2
bots
non-bots
0.25
0.20
0.15
0.10
0.05
0.00
2.5
5.0
7.5
10.0
12.5
15.0
17.5

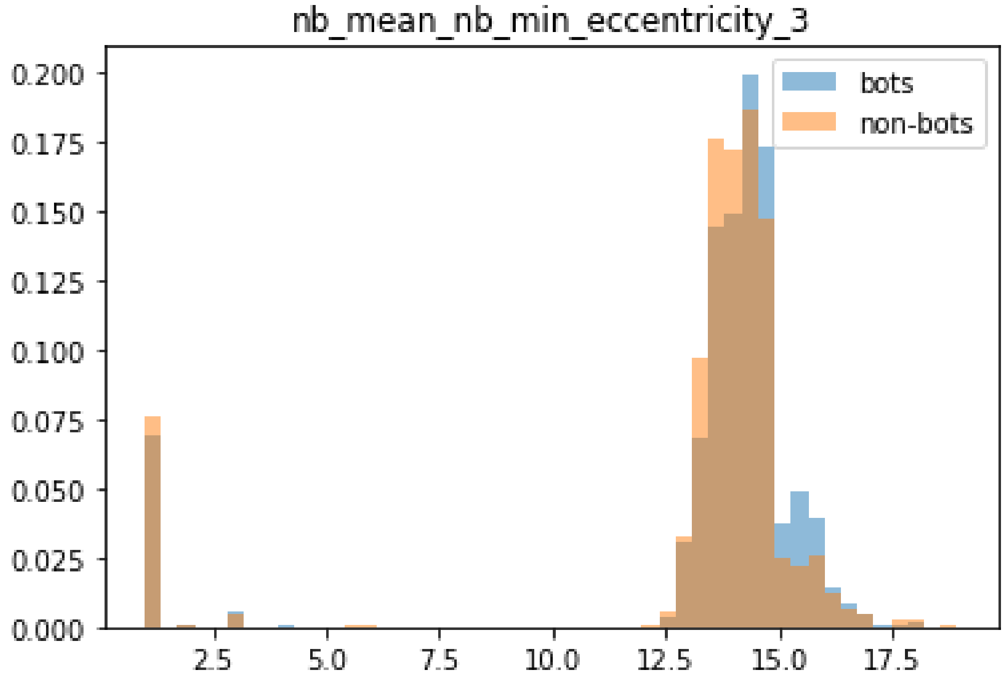
nb_mean_nb_min_eccentricity_3
bots
non-bots
0.200
0.175
0.150
0.125
0.100
0.075
0.050
0.025
0.000
2.5
5.0
7.5
10.0
12.5
15.0
17.5

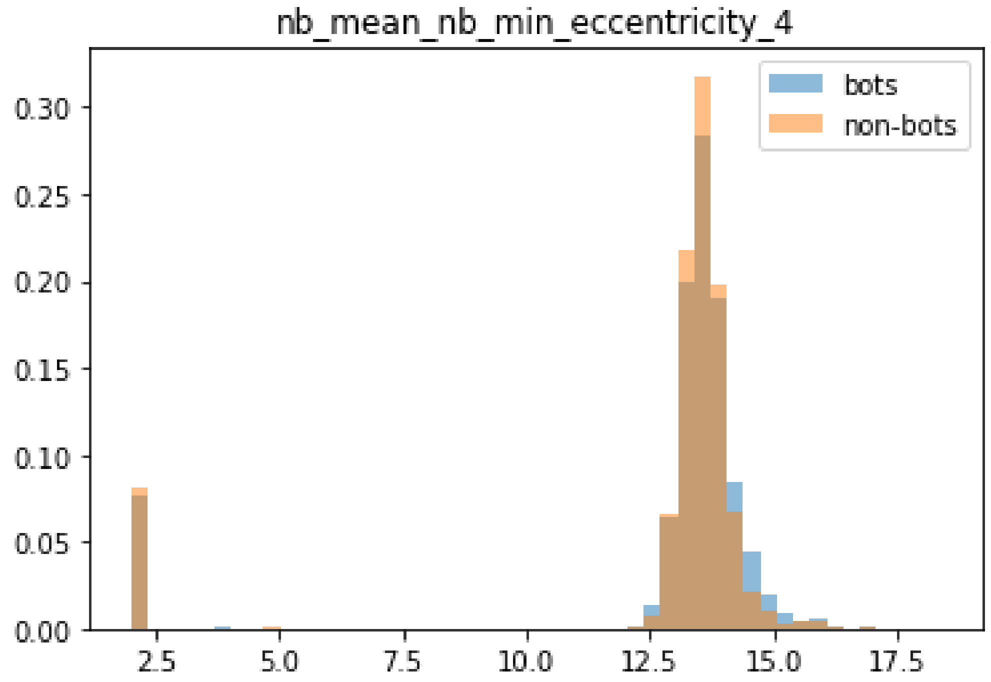
nb_mean_nb_min_eccentricity_4
bots
non-bots
0.30
0.25
0.20
0.15
0.10
0.05
0.00
2.5
5.0
7.5
10.0
12.5
15.0
17.5

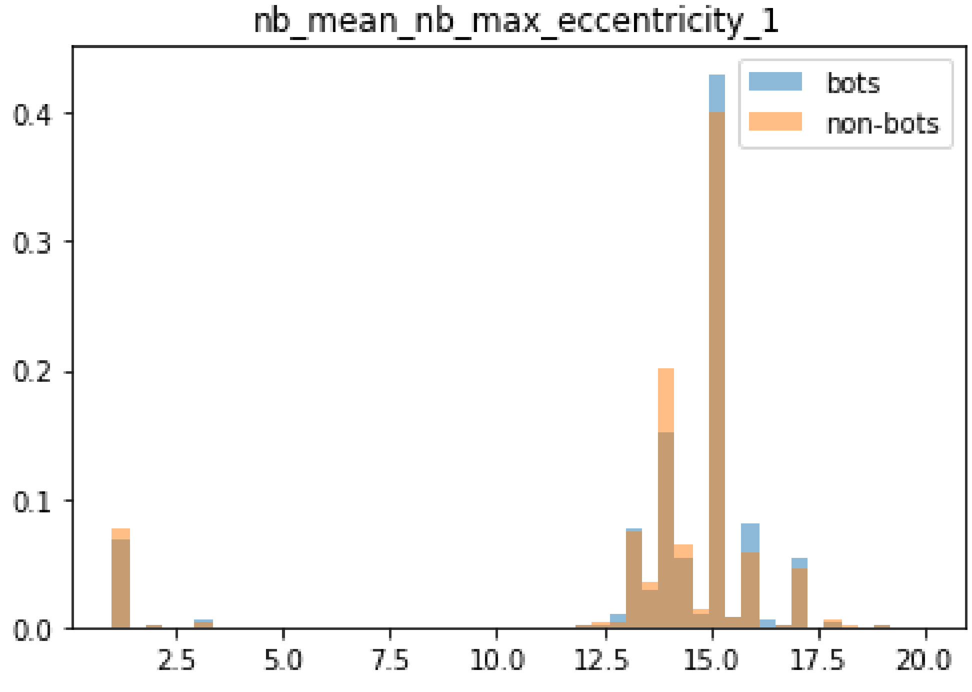
nb_mean_nb_max_eccentricity_1
bots
non-bots
0.4
0.3
0.2
0.1
0.0
2.5
5.0
7.5
10.0
12.5
15.0
17.5
20.0

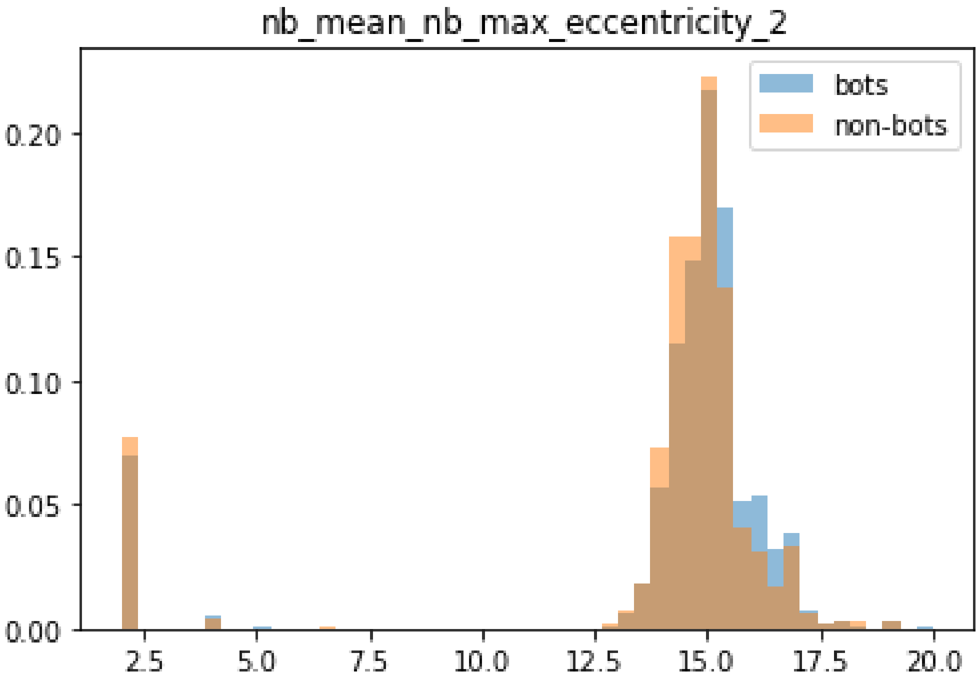
nb_mean_nb_max_eccentricity_2
bots
non-bots
0.20
0.15
0.10
0.05
0.00
2.5
5.0
7.5
10.0
12.5
15.0
17.5
20.0

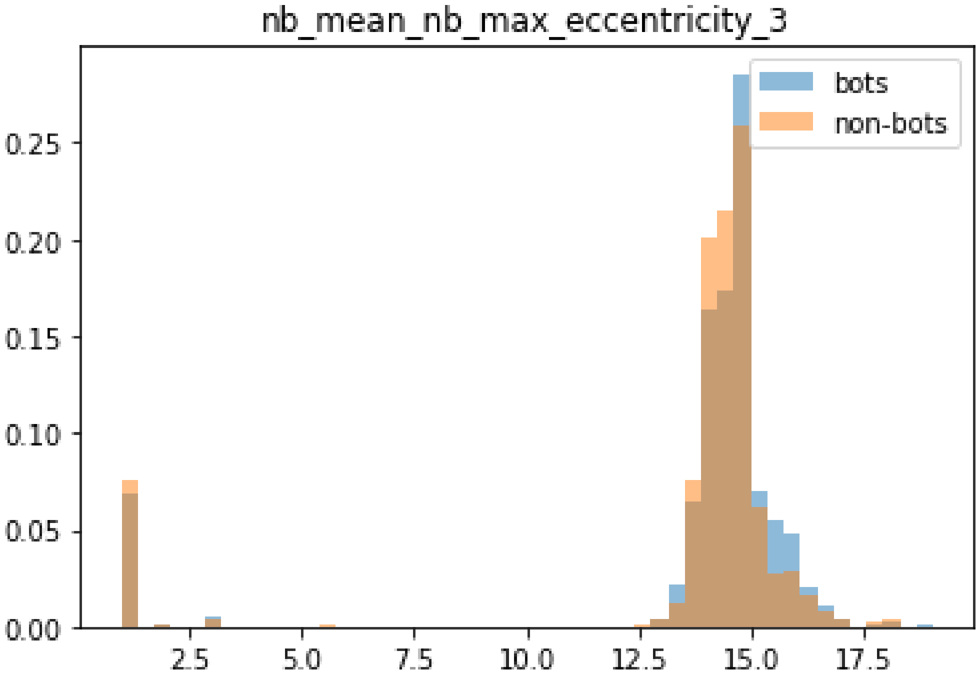
nb_mean_nb_max_eccentricity_3
bots
non-bots
0.25
0.20
0.15
0.10
0.05
0.00
2.5
5.0
7.5
10.0
12.5
15.0
17.5

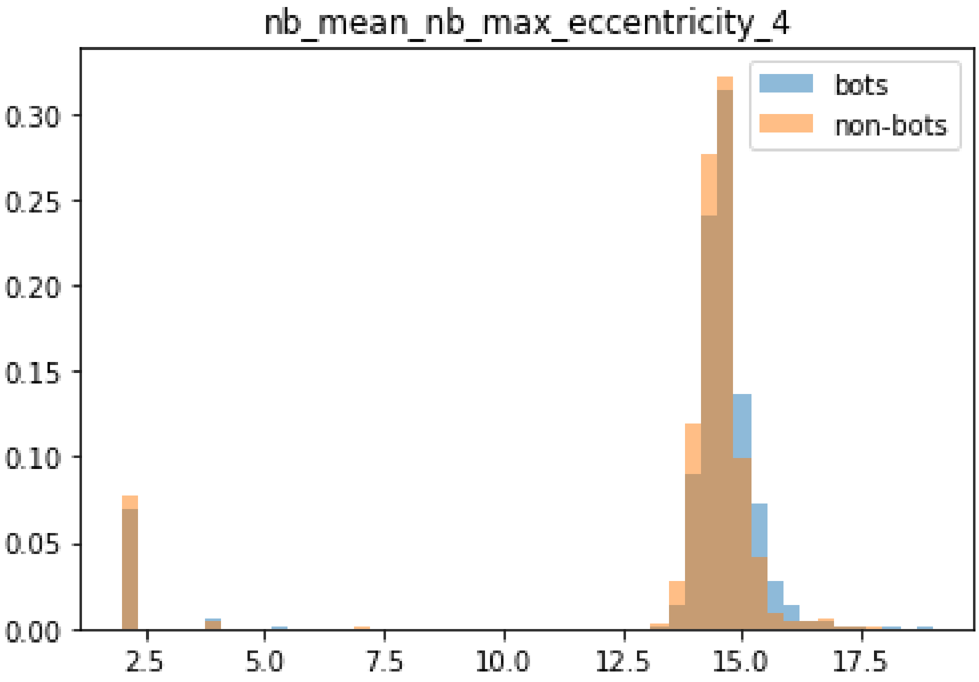
nb_mean_nb_max_eccentricity_4
bots
non-bots
0.30
0.25
0.20
0.15
0.10
0.05
0.00
2.5
5.0
7.5
10.0
12.5
15.0
17.5

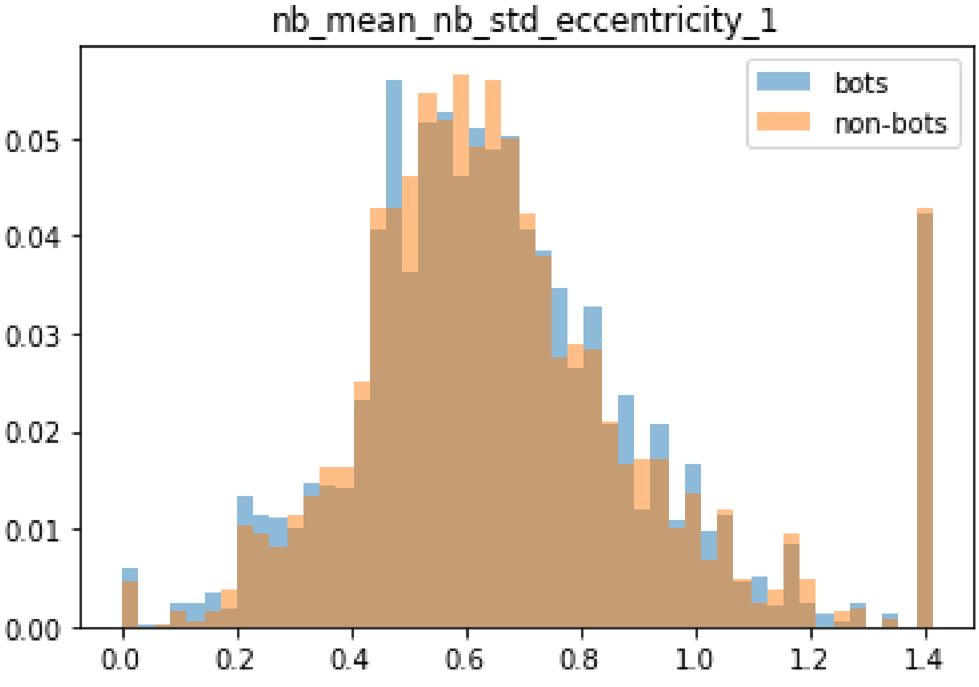
nb_mean_nb_std_eccentricity_1
bots
non-bots
0.05
0.04
0.03
0.02
0.01
0.00
0.0
0.2
0.4
0.6
0.8
1.0
1.2
1.4

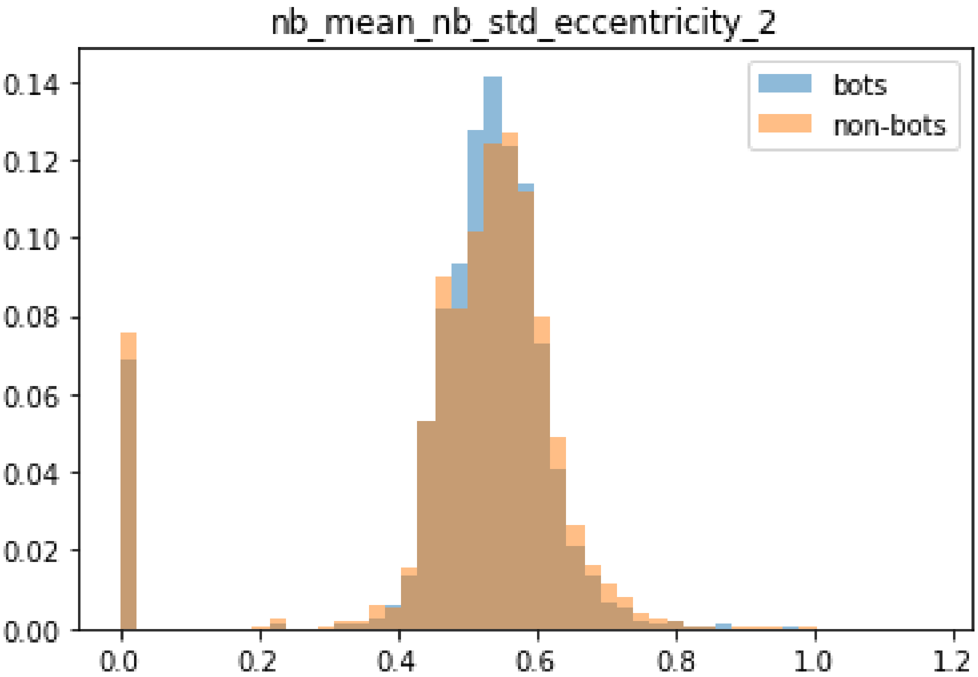
nb_mean_nb_std_eccentricity_2
bots
non-bots
0.14
0.12
0.10
0.08
0.06
0.04
0.02
0.00
0.0
0.2
0.4
0.6
0.8
1.0
1.2

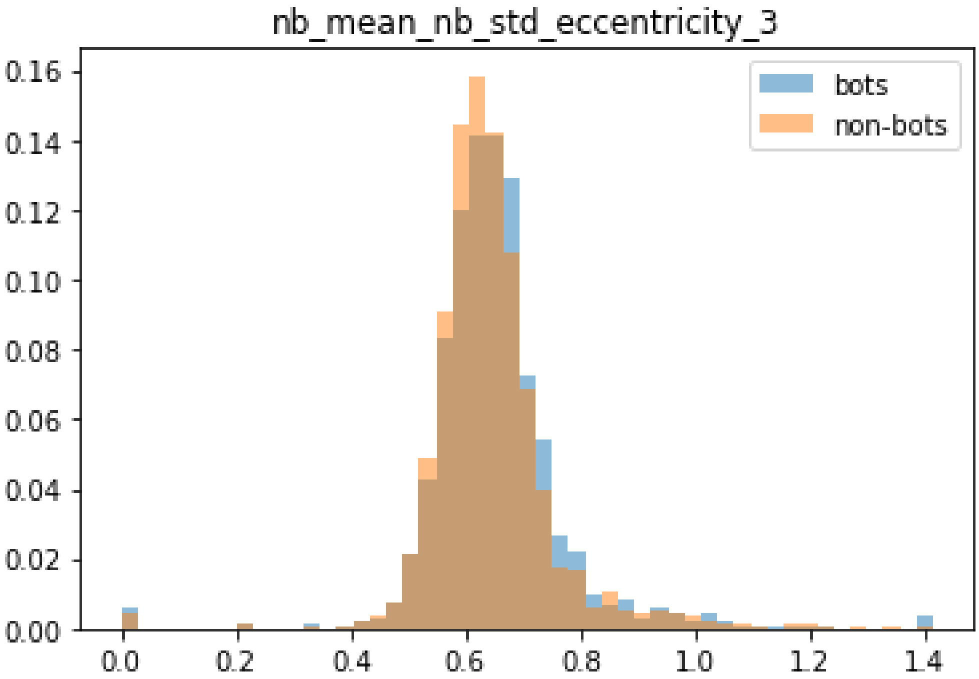
nb_mean_nb_std_eccentricity_3
bots
non-bots
0.16
0.14
0.12
0.10
0.08
0.06
0.04
0.02
0.00
0.0
0.2
0.4
0.6
0.8
1.0
1.2
1.4

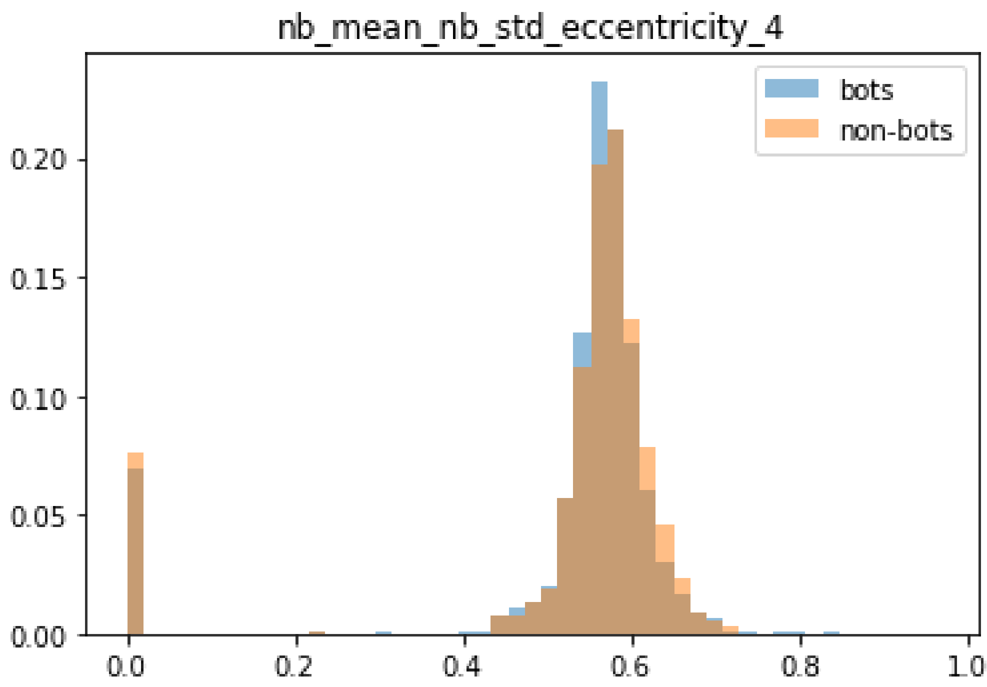
nb_mean_nb_std_eccentricity_4
bots
non-bots
0.20
0.15
0.10
0.05
0.00
0.0
0.2
0.4
0.6
0.8
1.0

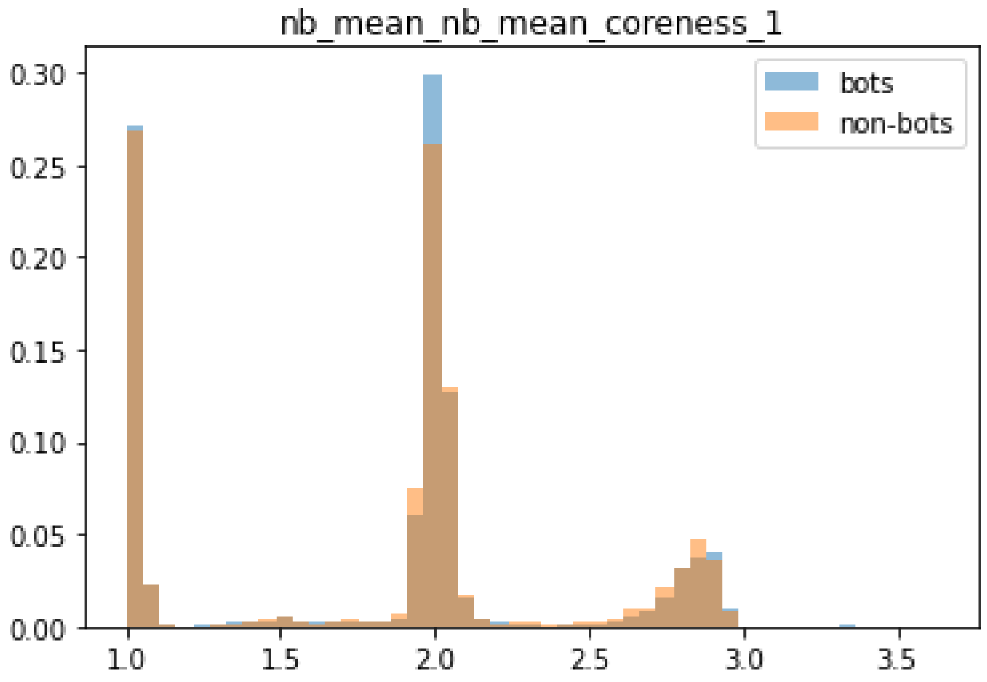
nb_mean_nb_mean_coreness_1
bots
non-bots
0.30
0.25
0.20
0.15
0.10
0.05
0.00
1.0
1.5
2.0
2.5
3.0
3.5

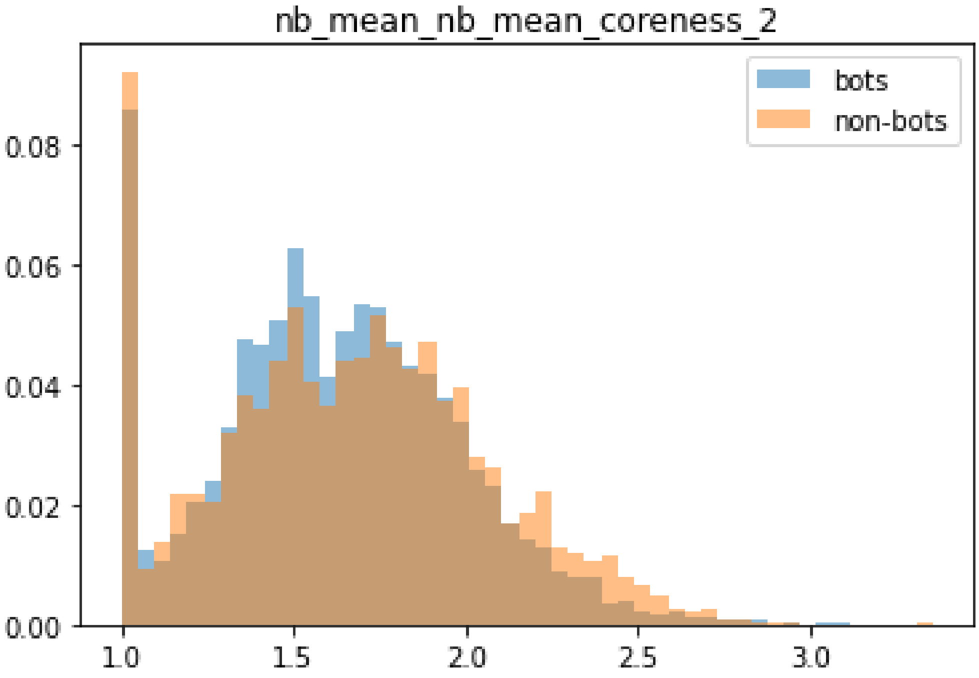

nb_mean_nb_mean_coreness_2
bots
non-bots
0.08
0.06
0.04
0.02
0.00
1.0
1.5
2.0
2.5
3.0

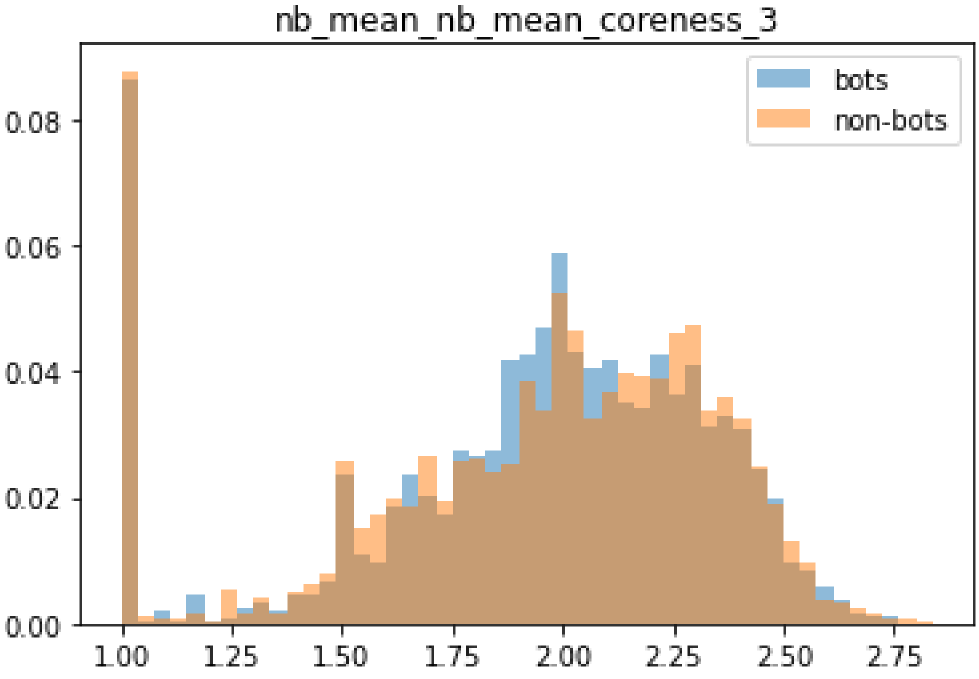

nb_mean_nb_mean_coreness_3
bots
non-bots
0.08
0.06
0.04
0.02
0.00
1.00
1.25
1.50
1.75
2.00
2.25
2.50
2.75

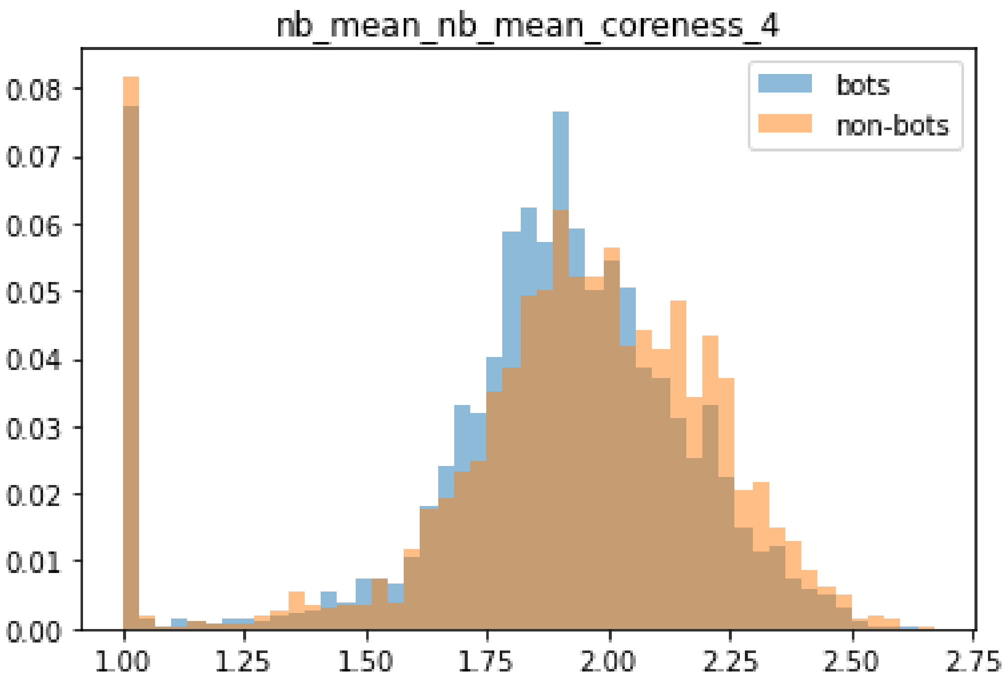
nb_mean_nb_mean_coreness_4
bots
non-bots
0.08
0.07
0.06
0.05
0.04
0.03
0.02
0.01
0.00
1.00
1.25
1.50
1.75
2.00
2.25
2.50
2.75

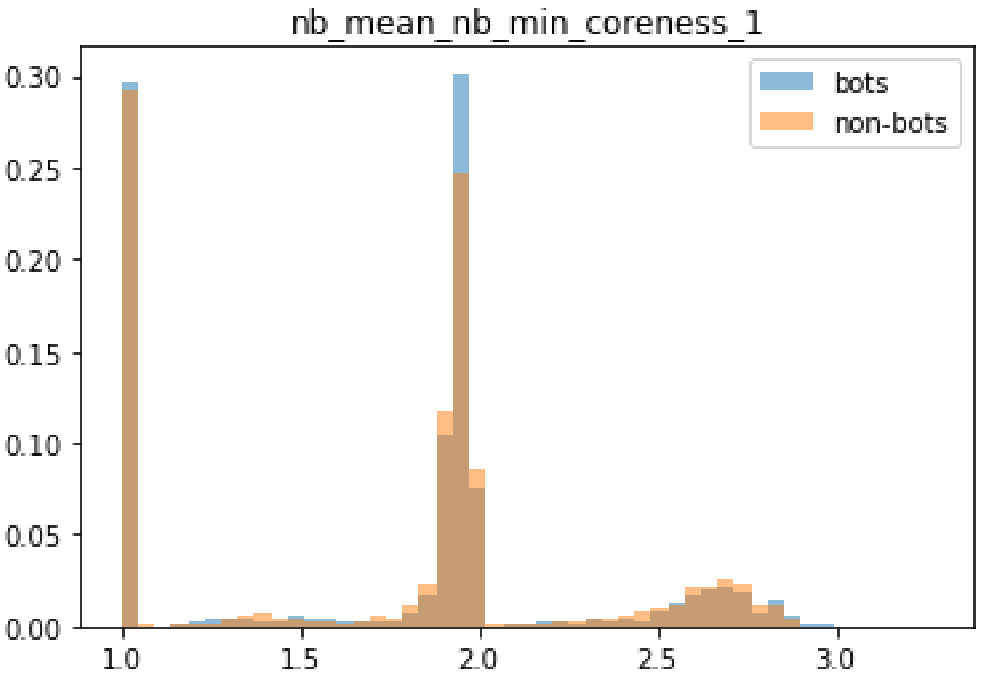
nb_mean_nb_min_coreness_1
bots
non-bots
0.30
0.25
0.20
0.15
0.10
0.05
0.00
1.0
1.5
2.0
2.5
3.0

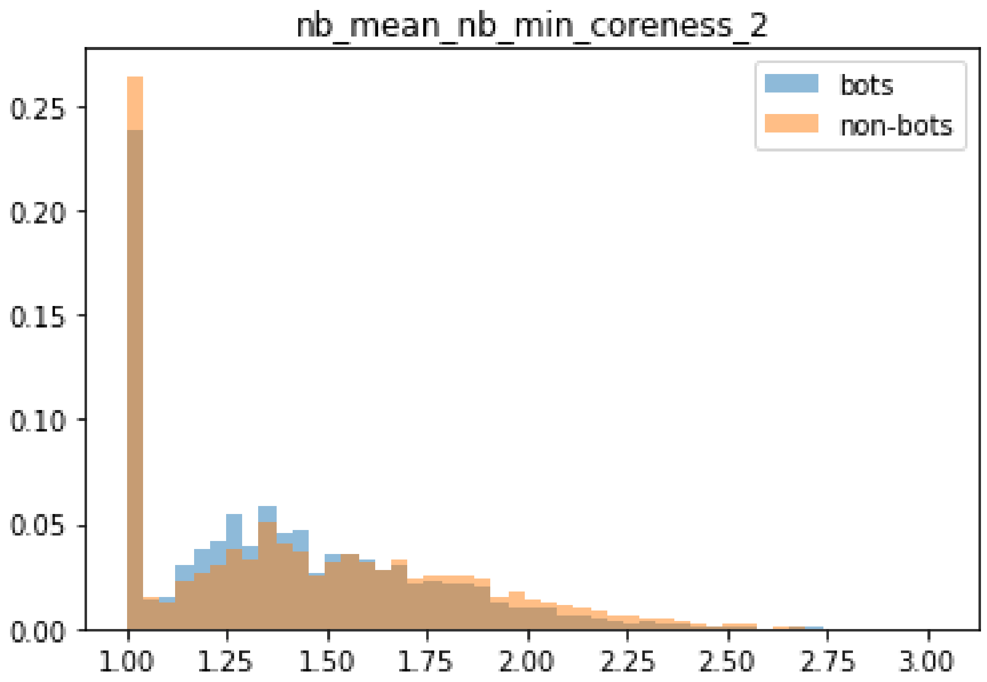
nb_mean_nb_min_coreness_2
bots
non-bots
0.25
0.20
0.15
0.10
0.05
0.00
1.00
1.25
1.50
1.75
2.00
2.25
2.50
2.75
3.00

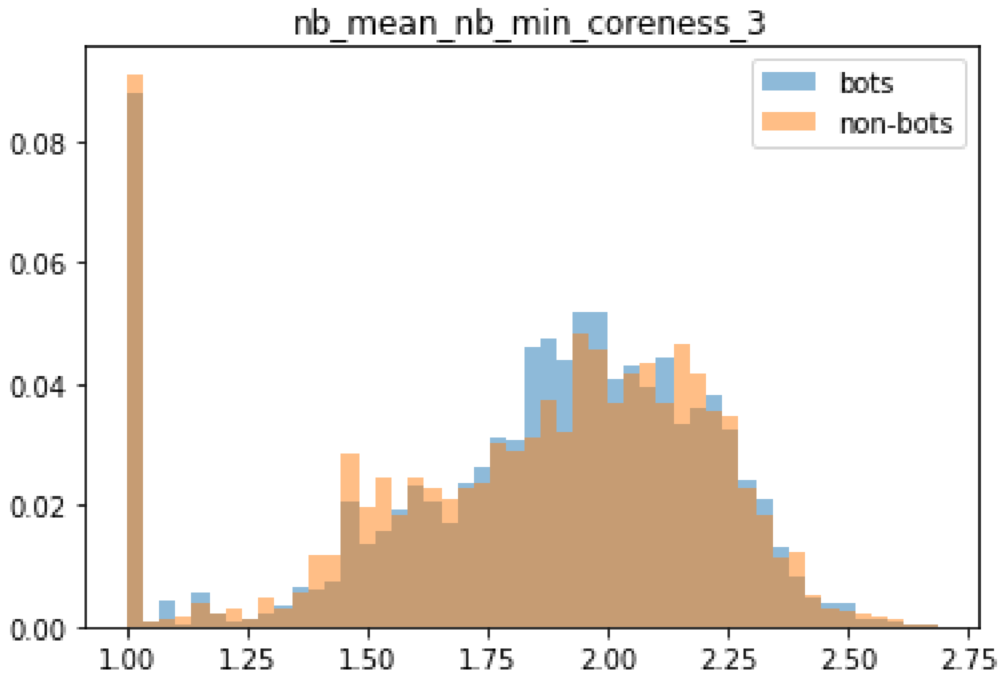
nb_mean_nb_min_coreness_3
bots
non-bots
0.08
0.06
0.04
0.02
0.00
1.00
1.25
1.50
1.75
2.00
2.25
2.50
2.75

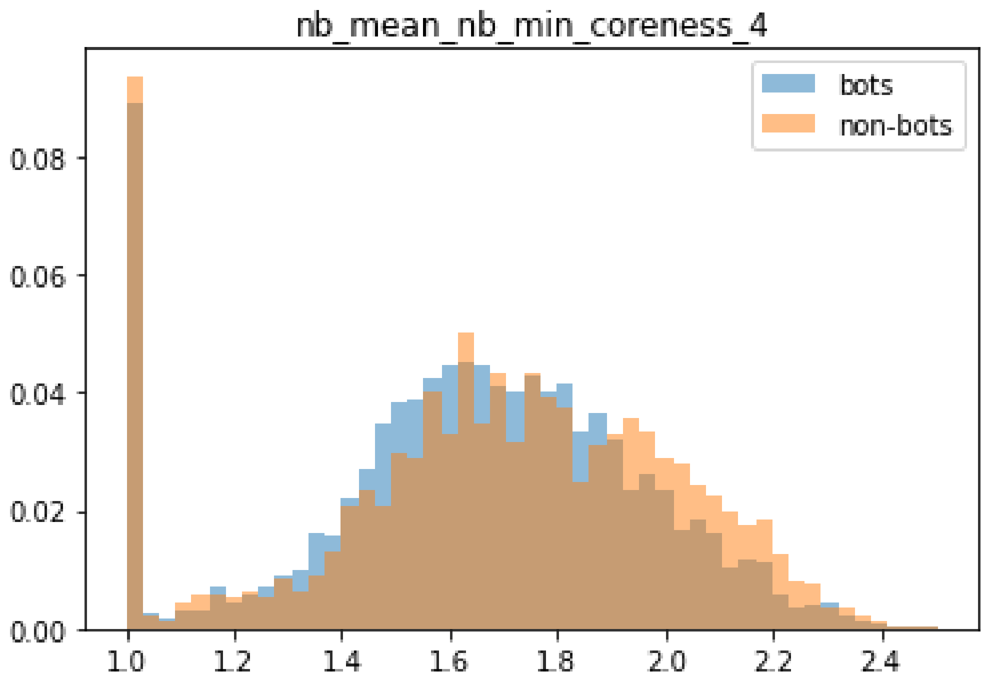
nb_mean_nb_min_coreness_4
bots
non-bots
0.00
0.02
0.04
0.06
0.08
1.0
1.2
1.4
1.6
1.8
2.0
2.2
2.4

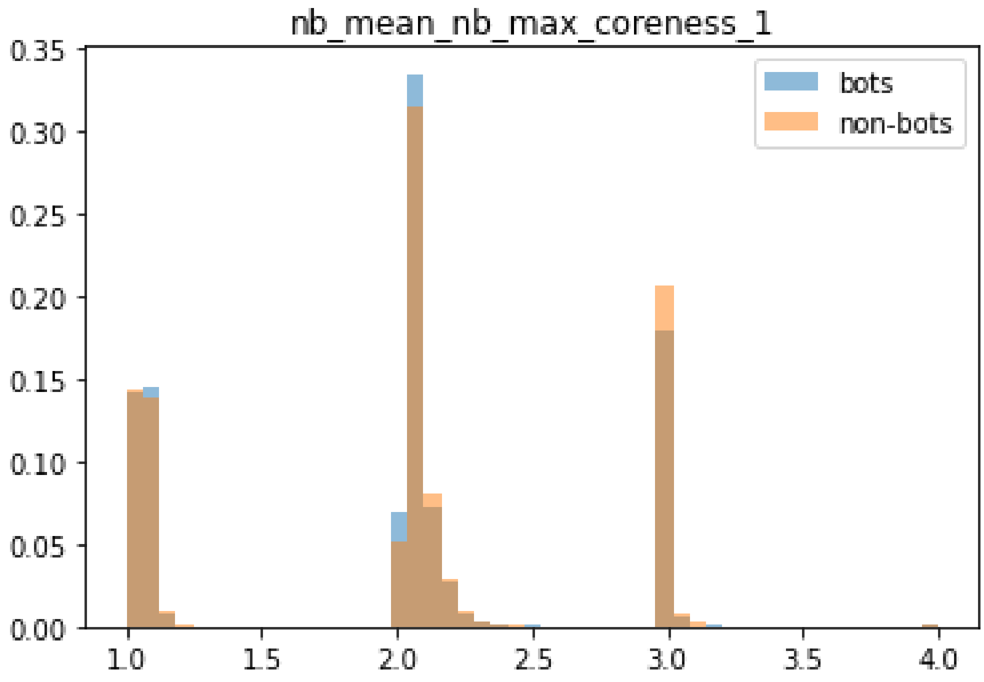
nb_mean_nb_max_coreness_1
bots
non-bots
0.00
0.05
0.10
0.15
0.20
0.25
0.30
0.35
1.0
1.5
2.0
2.5
3.0
3.5
4.0

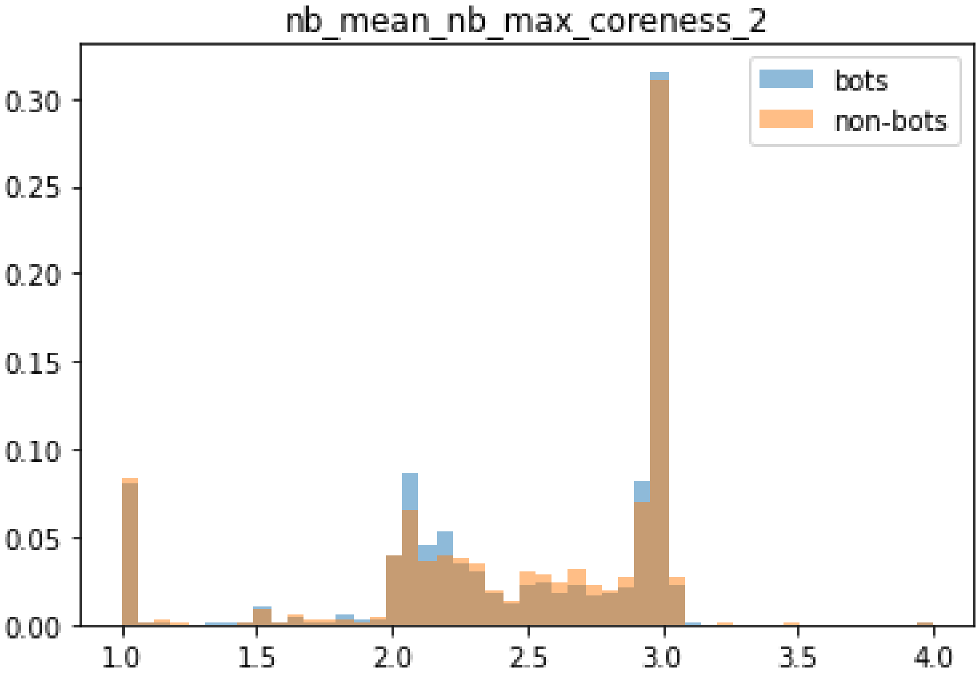
nb_mean_nb_max_coreness_2
bots
non-bots
0.30
0.25
0.20
0.15
0.10
0.05
0.00
1.0
1.5
2.0
2.5
3.0
3.5
4.0

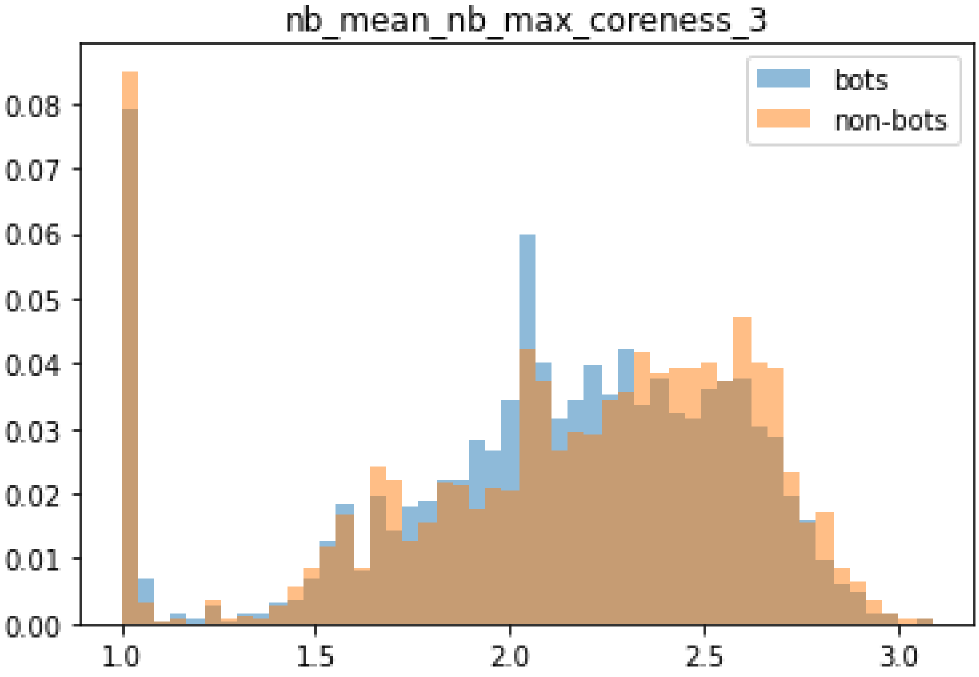
nb_mean_nb_max_coreness_3
bots
non-bots
0.08
0.07
0.06
0.05
0.04
0.03
0.02
0.01
0.00
1.0
1.5
2.0
2.5
3.0

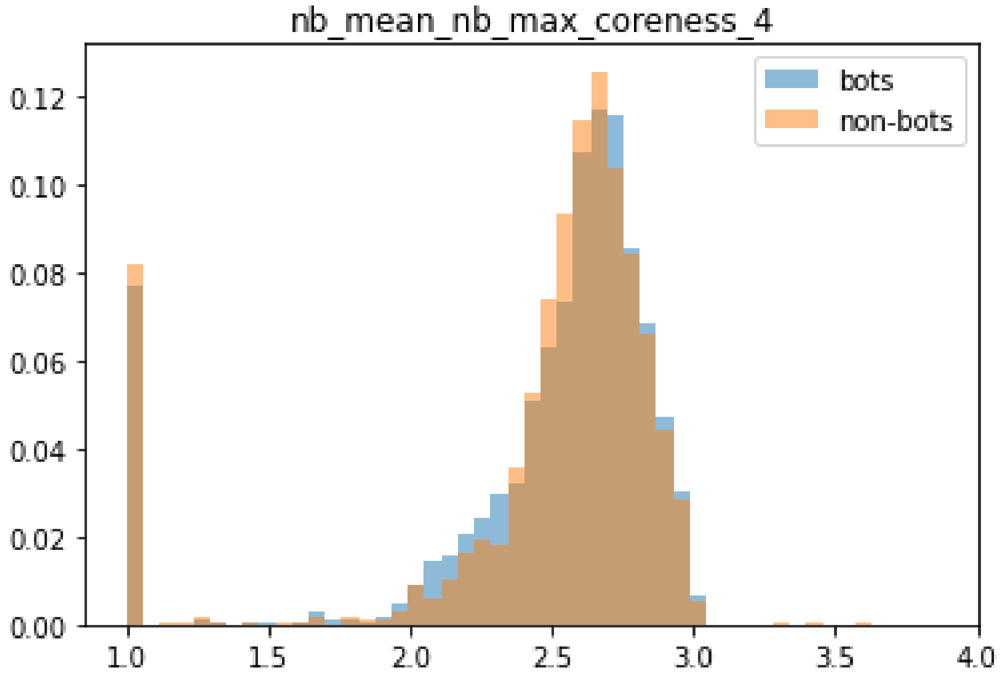
nb_mean_nb_max_coreness_4
bots
non-bots
0.12
0.10
0.08
0.06
0.04
0.02
0.00
1.0
1.5
2.0
2.5
3.0
3.5
4.0

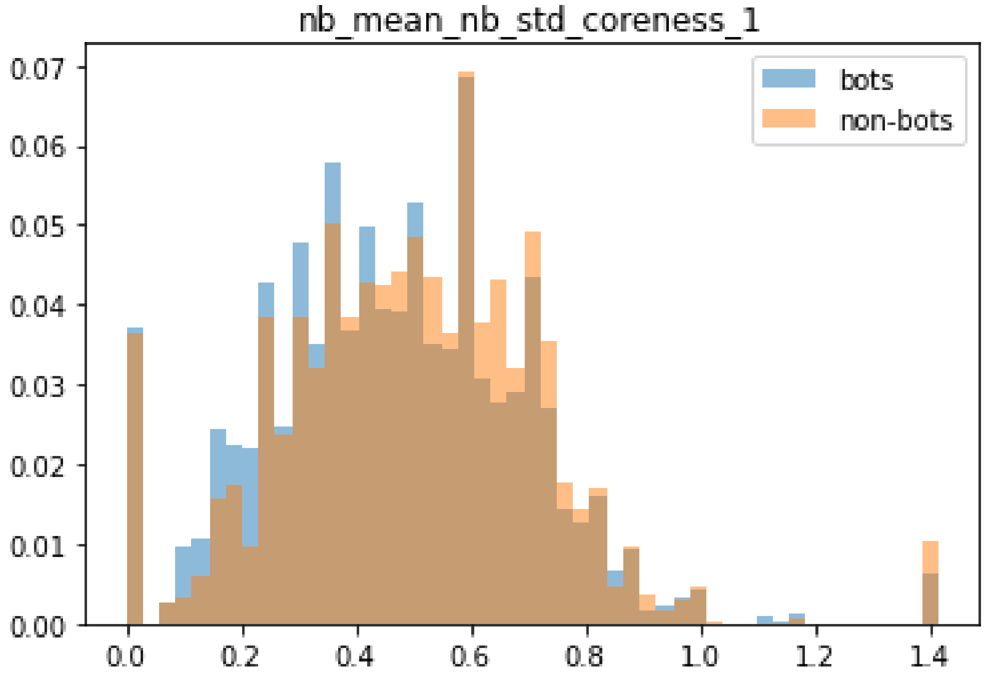
nb_mean_nb_std_coreness_1
bots
non-bots
0.07
0.06
0.05
0.04
0.03
0.02
0.01
0.00
0.0
0.2
0.4
0.6
0.8
1.0
1.2
1.4

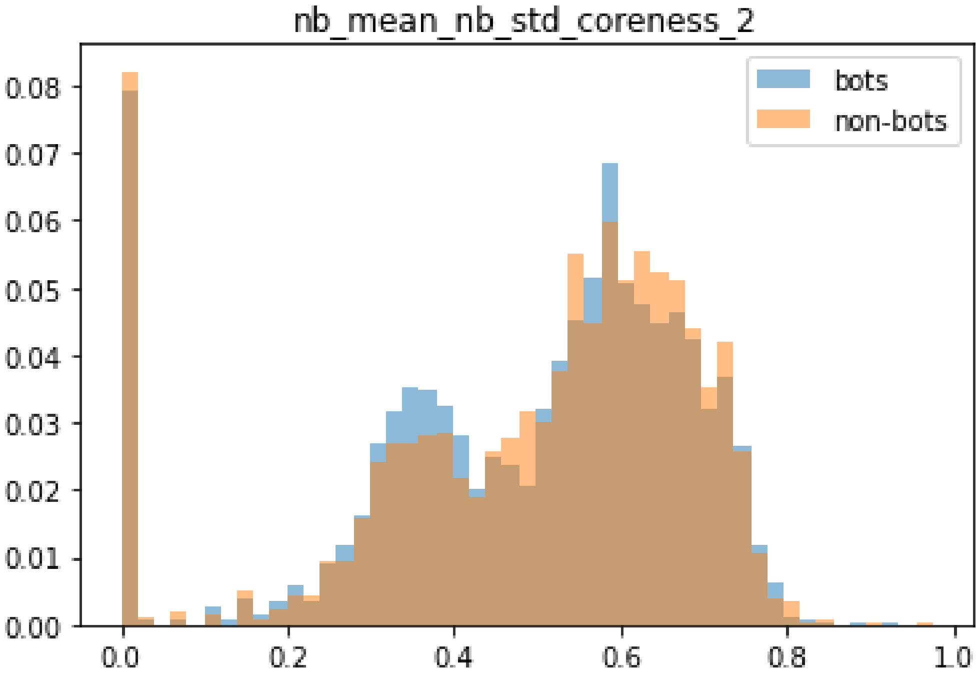
nb_mean_nb_std_coreness_2
bots
non-bots
0.08
0.07
0.06
0.05
0.04
0.03
0.02
0.01
0.00
0.0
0.2
0.4
0.6
0.8
1.0

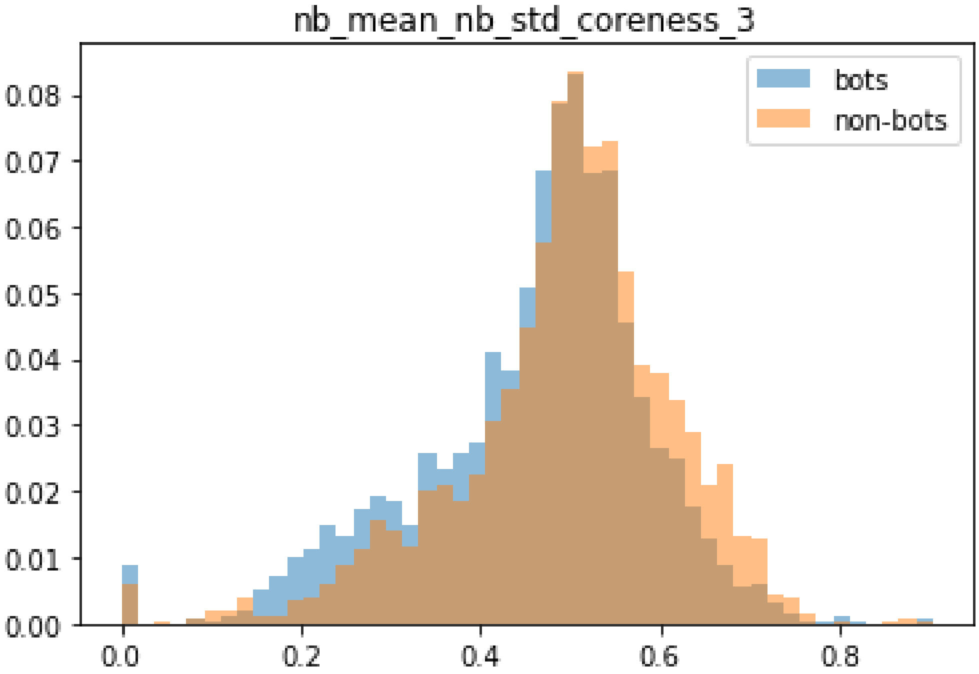
nb_mean_nb_std_coreness_3
bots
non-bots
0.08
0.07
0.06
0.05
0.04
0.03
0.02
0.01
0.00
0.0
0.2
0.4
0.6
0.8

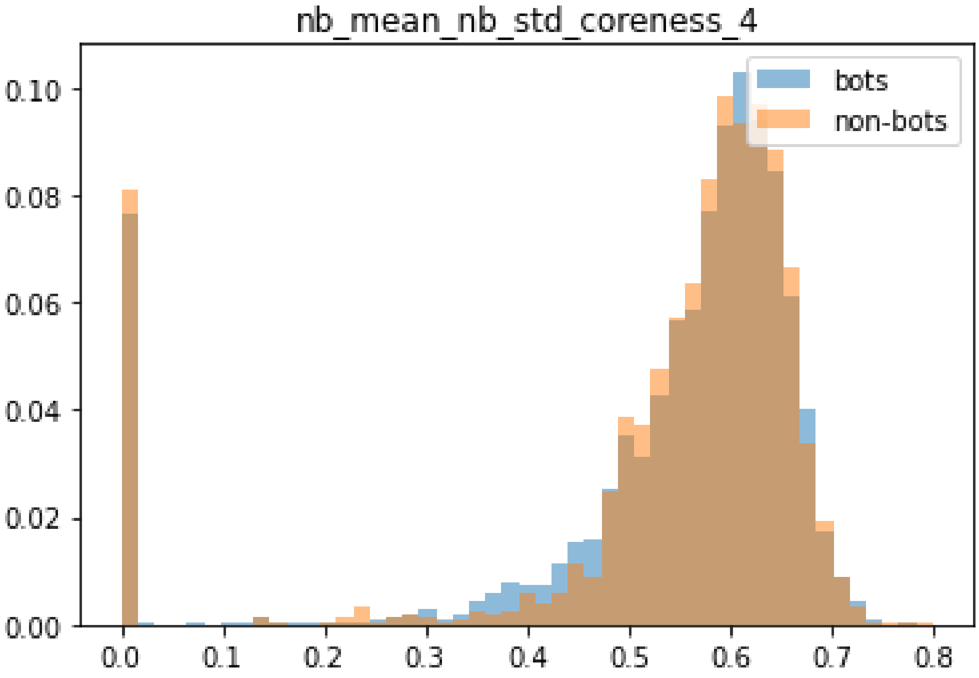

nb_mean_nb_std_coreness_4
bots
non-bots
0.00
0.02
0.04
0.06
0.08
0.10
0.0
0.1
0.2
0.3
0.4
0.5
0.6
0.7
0.8

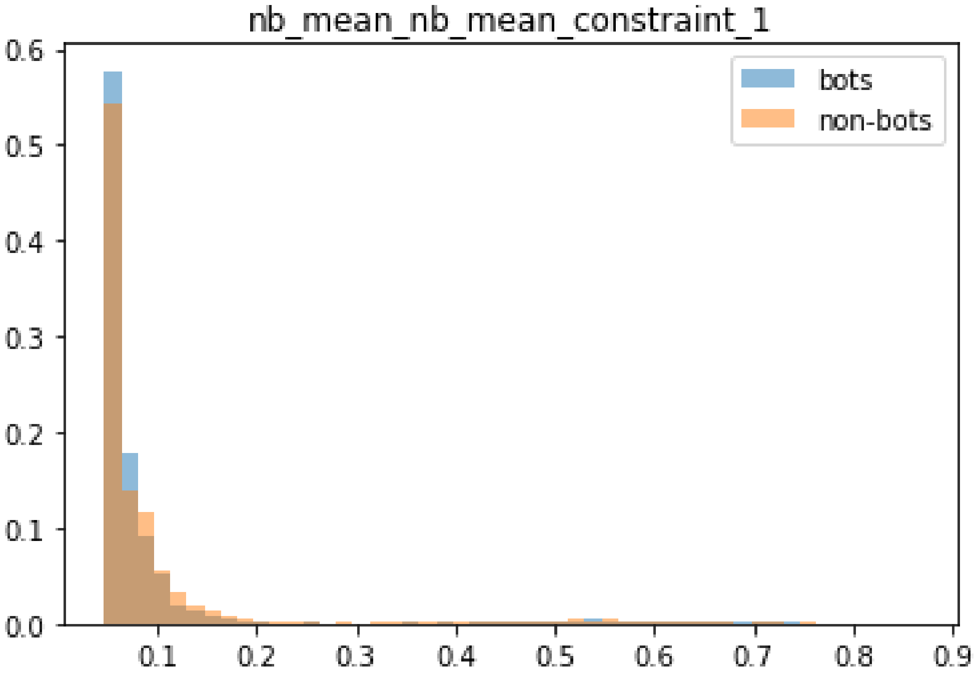

nb_mean_nb_mean_constraint_1
bots
non-bots
0.0
0.1
0.2
0.3
0.4
0.5
0.6
0.1
0.2
0.3
0.4
0.5
0.6
0.7
0.8
0.9

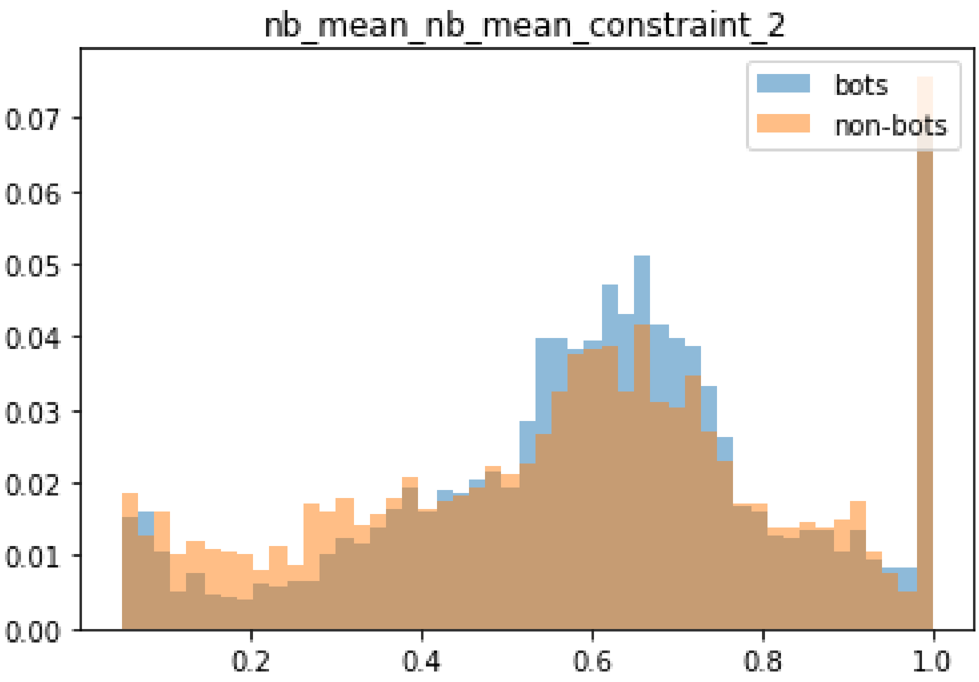
nb_mean_nb_mean_constraint_2
bots
non-bots
0.07
0.06
0.05
0.04
0.03
0.02
0.01
0.00
0.2
0.4
0.6
0.8
1.0

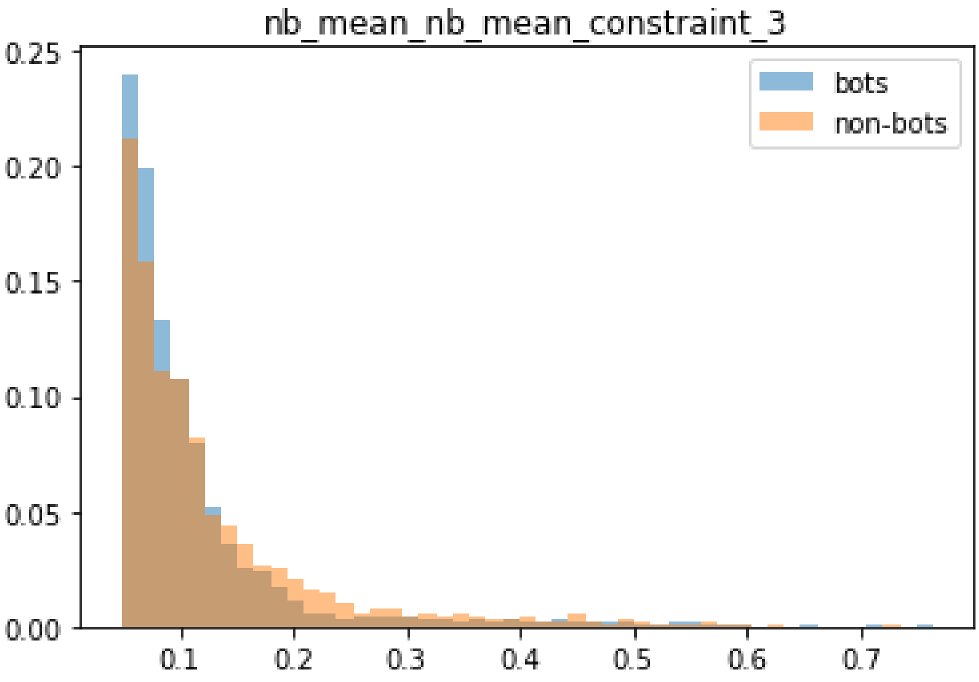
nb_mean_nb_mean_constraint_3
bots
non-bots
0.25
0.20
0.15
0.10
0.05
0.00
0.1
0.2
0.3
0.4
0.5
0.6
0.7

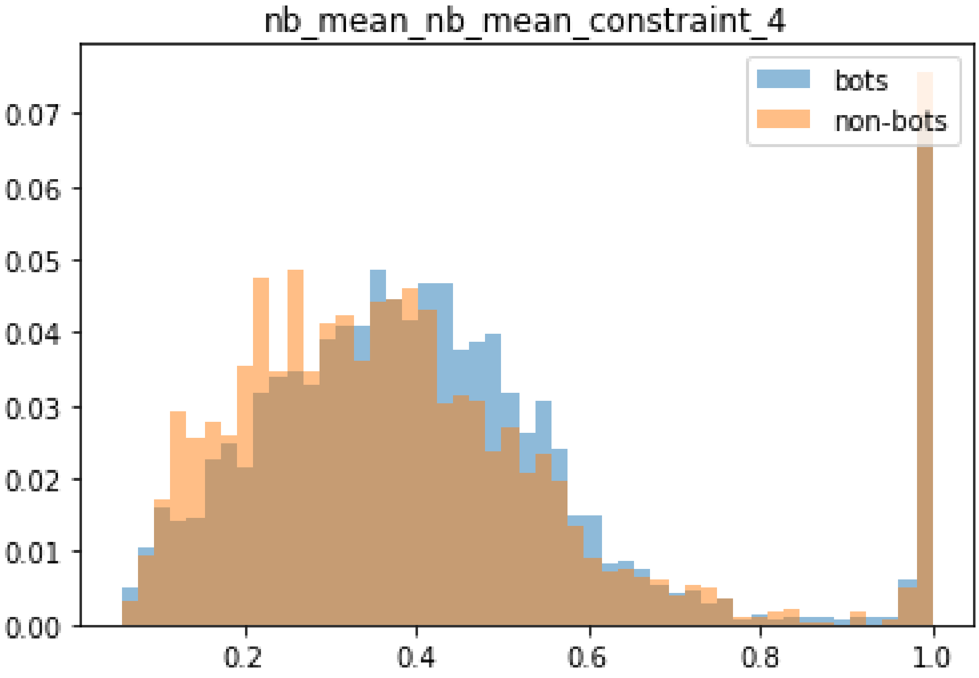
nb_mean_nb_mean_constraint_4
bots
non-bots
0.07
0.06
0.05
0.04
0.03
0.02
0.01
0.00
0.2
0.4
0.6
0.8
1.0

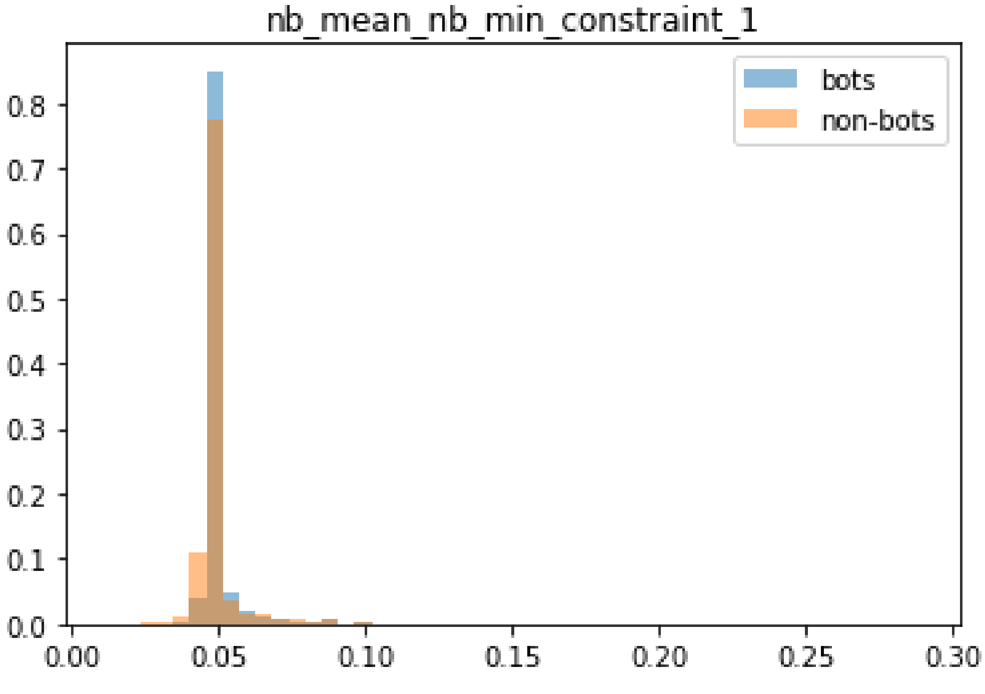
nb_mean_nb_min_constraint_1
bots
non-bots
0.8
0.7
0.6
0.5
0.4
0.3
0.2
0.1
0.0
0.00
0.05
0.10
0.15
0.20
0.25
0.30

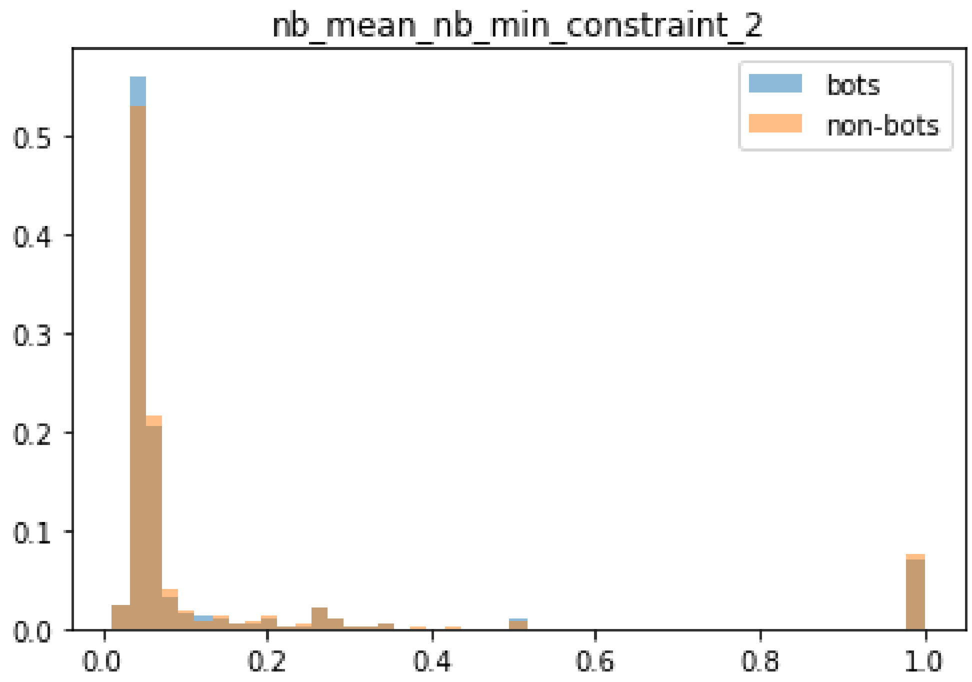
nb_mean_nb_min_constraint_2
bots
non-bots
0.0
0.1
0.2
0.3
0.4
0.5
0.0
0.2
0.4
0.6
0.8
1.0

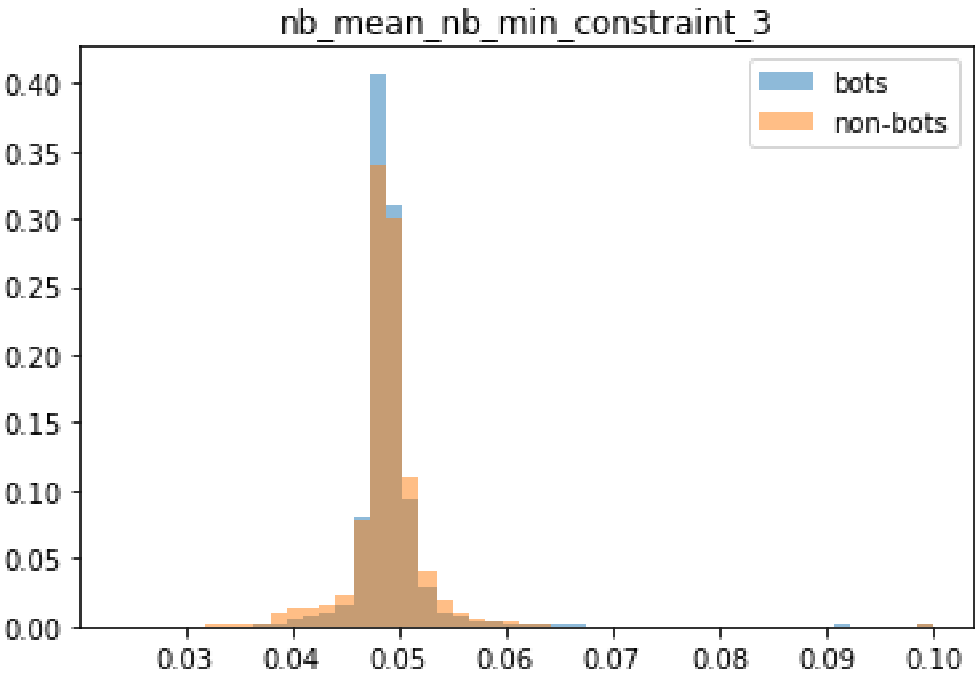
nb_mean_nb_min_constraint_3
bots
non-bots
0.00
0.05
0.10
0.15
0.20
0.25
0.30
0.35
0.40
0.03
0.04
0.05
0.06
0.07
0.08
0.09
0.10

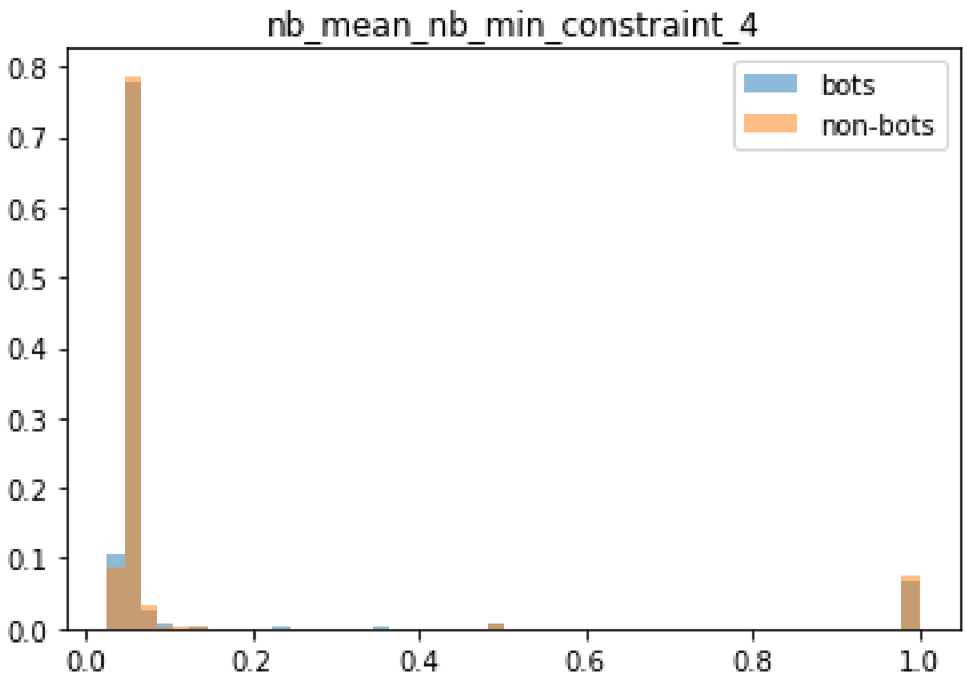
nb_mean_nb_min_constraint_4
bots
non-bots
0.0
0.1
0.2
0.3
0.4
0.5
0.6
0.7
0.8
0.0
0.2
0.4
0.6
0.8
1.0

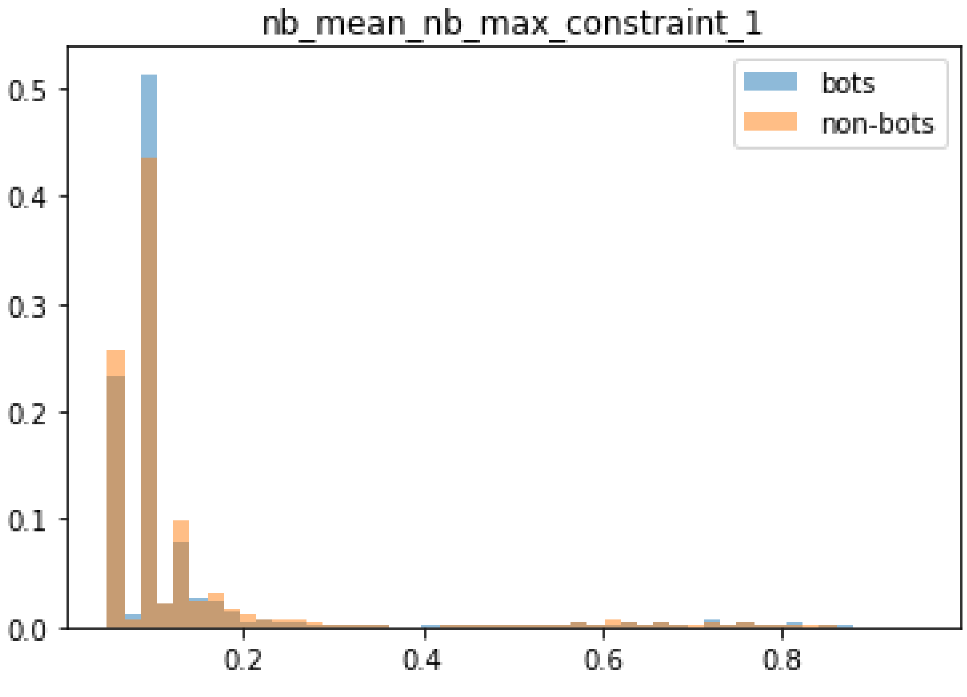
nb_mean_nb_max_constraint_1
bots
non-bots
0.0
0.1
0.2
0.3
0.4
0.5
0.2
0.4
0.6
0.8

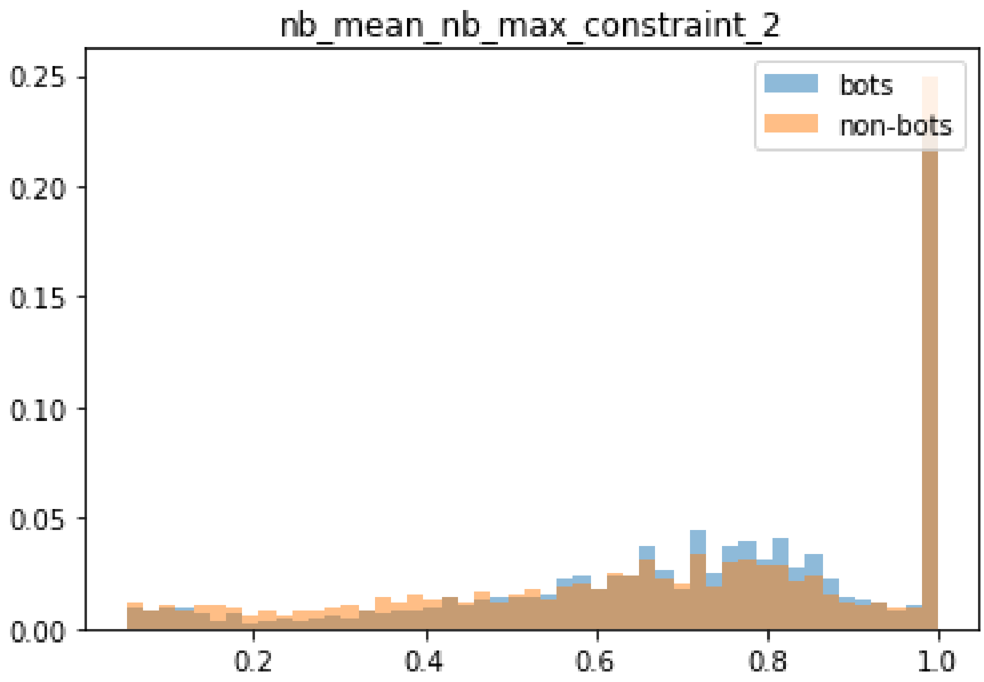
nb_mean_nb_max_constraint_2
bots
non-bots
0.25
0.20
0.15
0.10
0.05
0.00
0.2
0.4
0.6
0.8
1.0

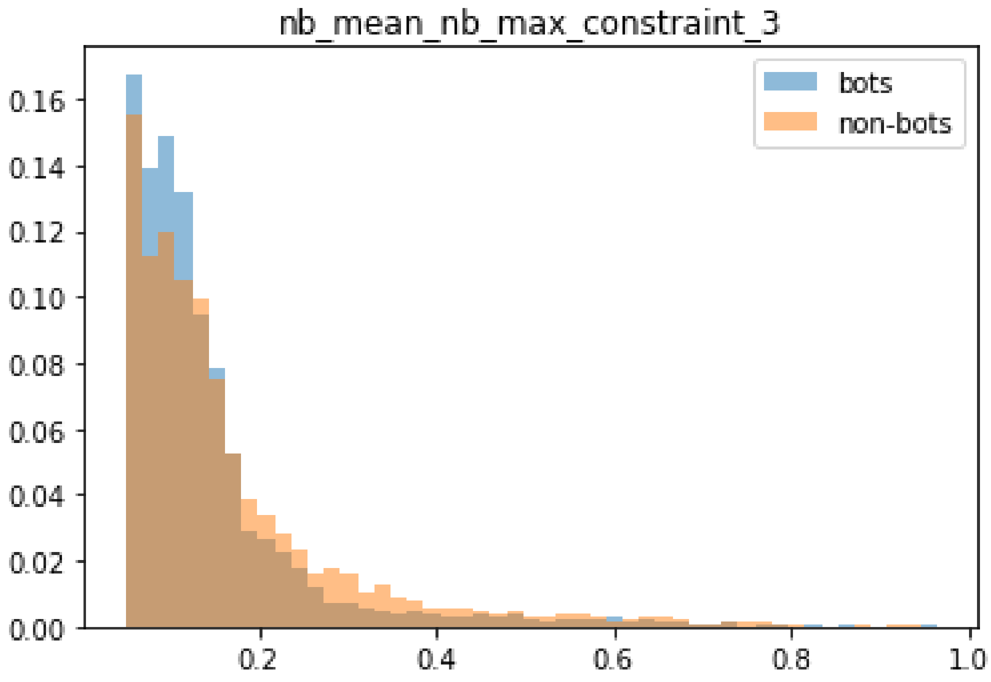
nb_mean_nb_max_constraint_3
bots
non-bots
0.16
0.14
0.12
0.10
0.08
0.06
0.04
0.02
0.00
0.2
0.4
0.6
0.8
1.0

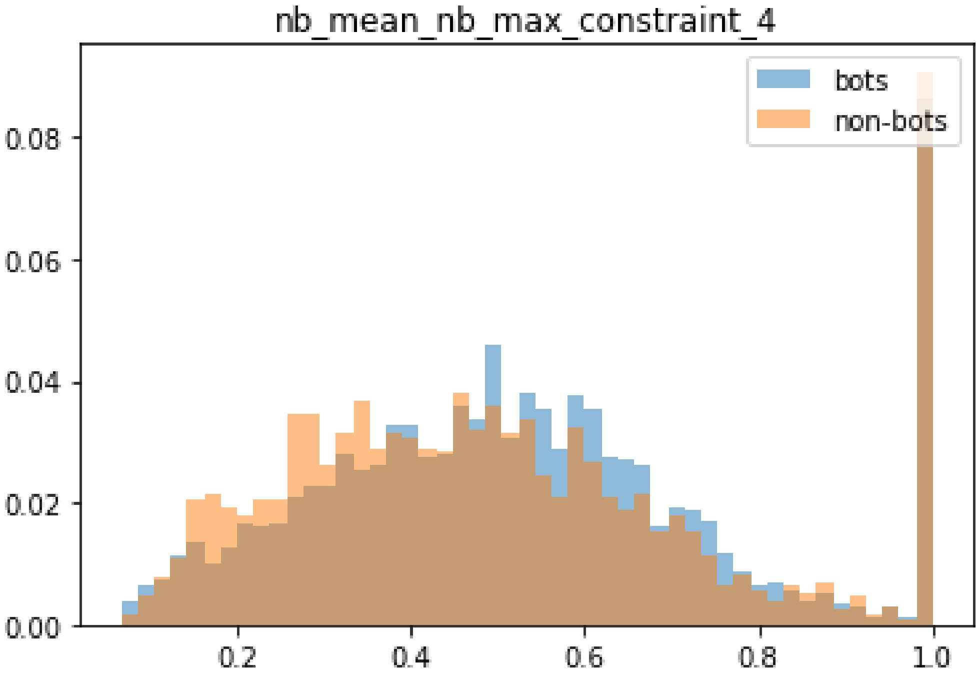
nb_mean_nb_max_constraint_4
bots
non-bots
0.08
0.06
0.04
0.02
0.00
0.2
0.4
0.6
0.8
1.0

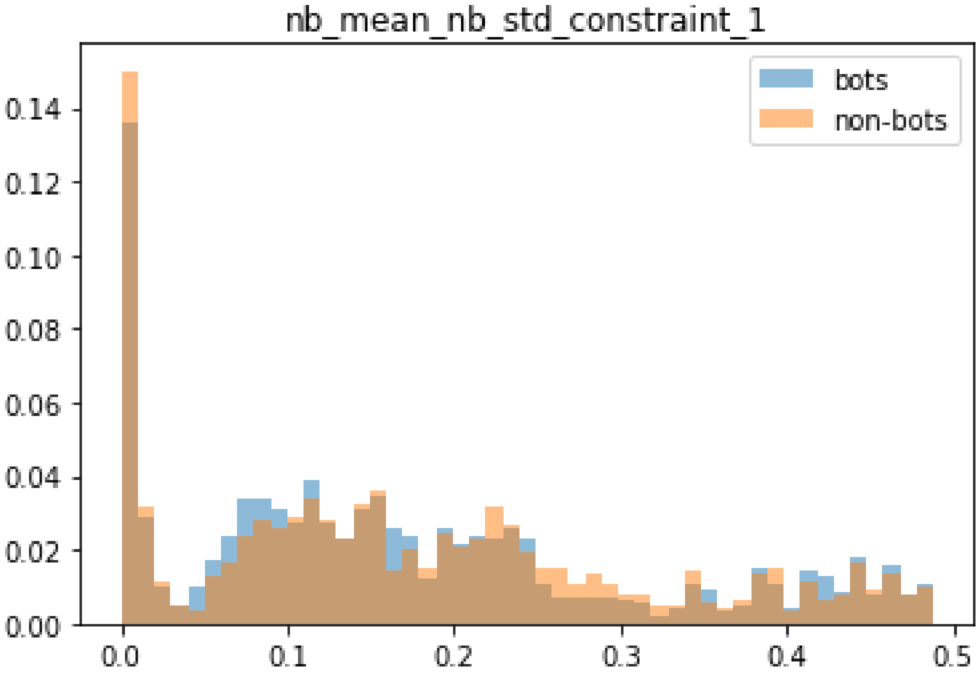
nb_mean_nb_std_constraint_1
bots
non-bots
0.14
0.12
0.10
0.08
0.06
0.04
0.02
0.00
0.0
0.1
0.2
0.3
0.4
0.5

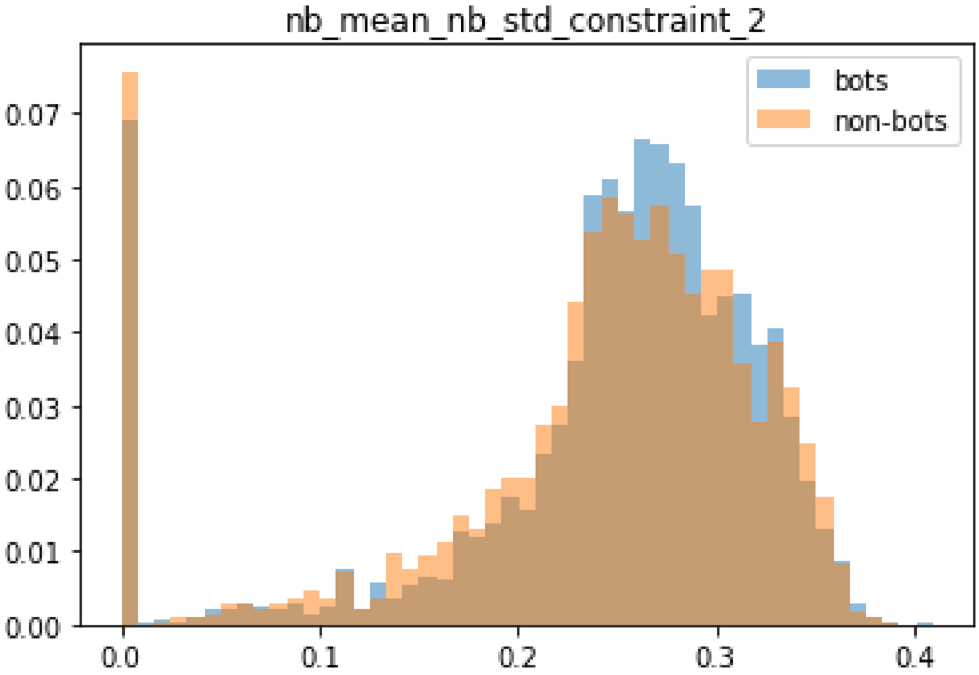
nb_mean_nb_std_constraint_2
bots
non-bots
0.07
0.06
0.05
0.04
0.03
0.02
0.01
0.00
0.0
0.1
0.2
0.3
0.4

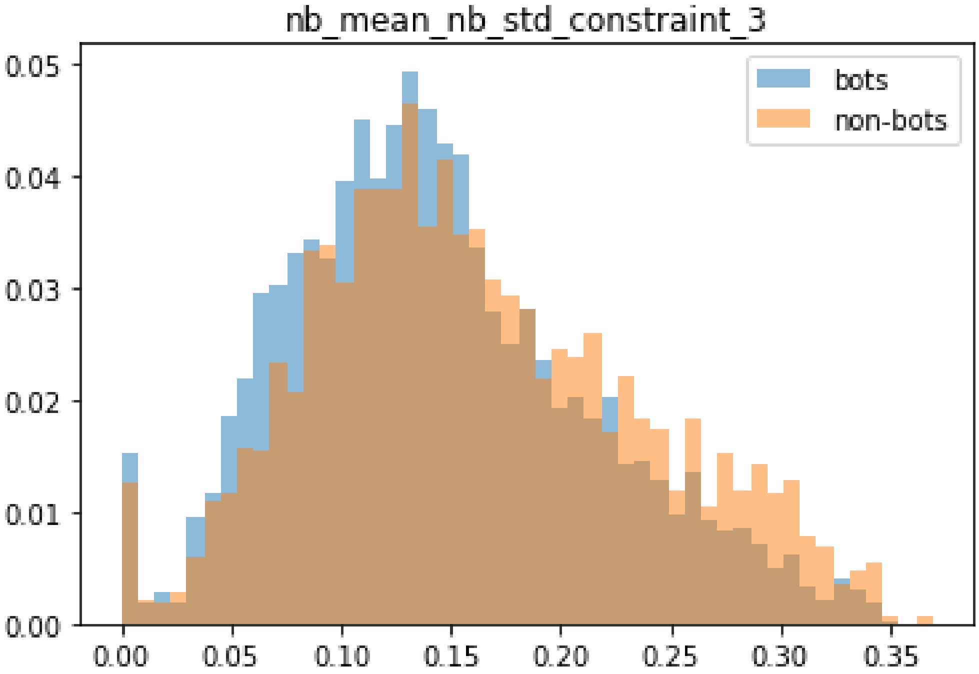
nb_mean_nb_std_constraint_3
bots
non-bots
0.05
0.04
0.03
0.02
0.01
0.00
0.00
0.05
0.10
0.15
0.20
0.25
0.30
0.35

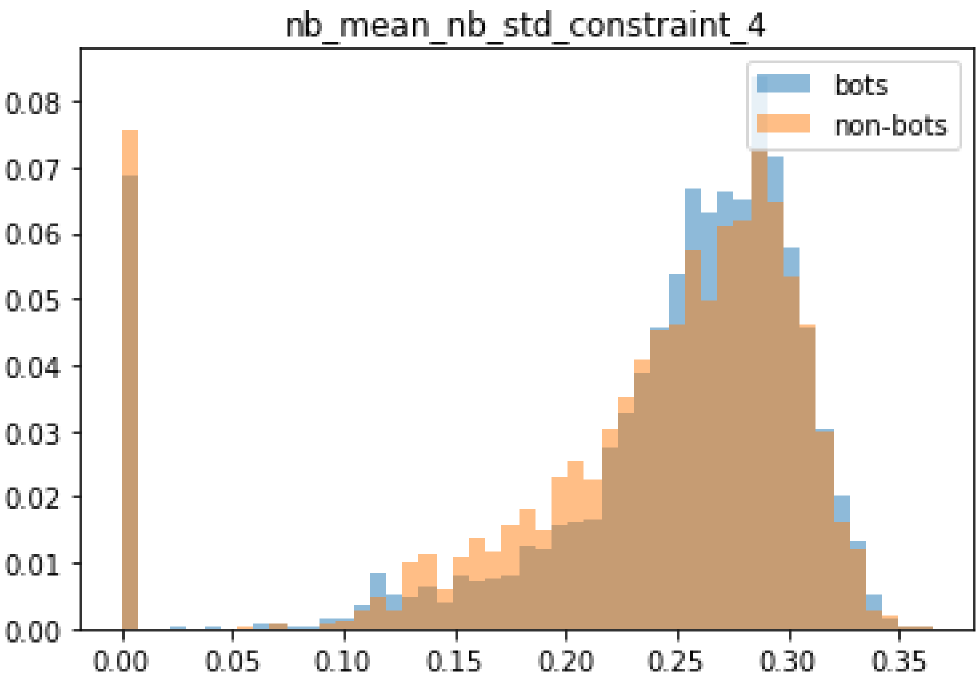
nb_mean_nb_std_constraint_4
bots
non-bots
0.00
0.01
0.02
0.03
0.04
0.05
0.06
0.07
0.08
0.00
0.05
0.10
0.15
0.20
0.25
0.30
0.35

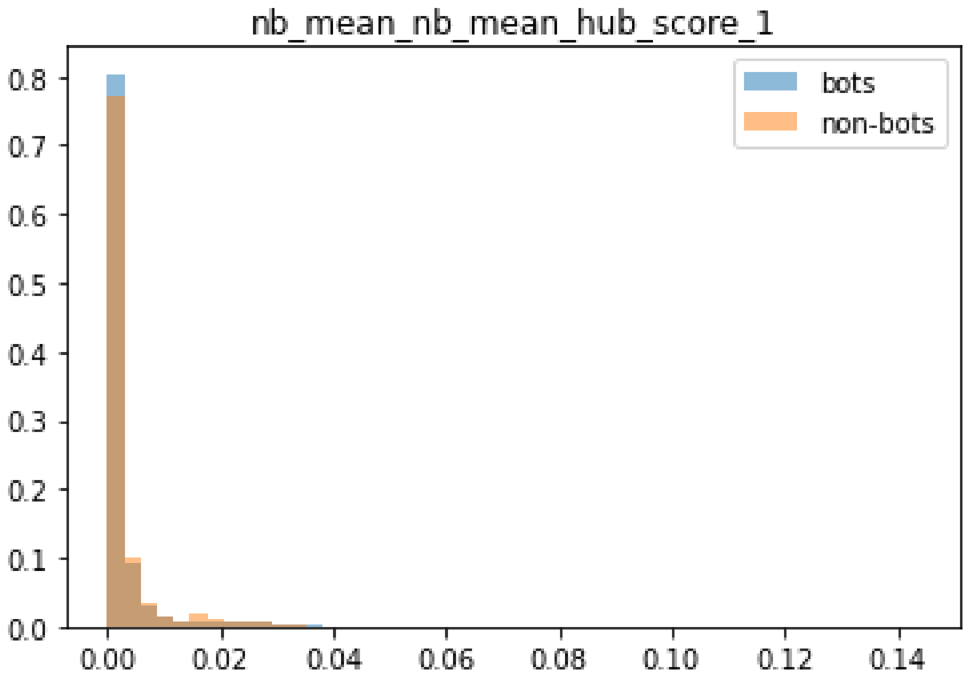
nb_mean_nb_mean_hub_score_1
bots
non-bots
0.0
0.1
0.2
0.3
0.4
0.5
0.6
0.7
0.8
0.00
0.02
0.04
0.06
0.08
0.10
0.12
0.14

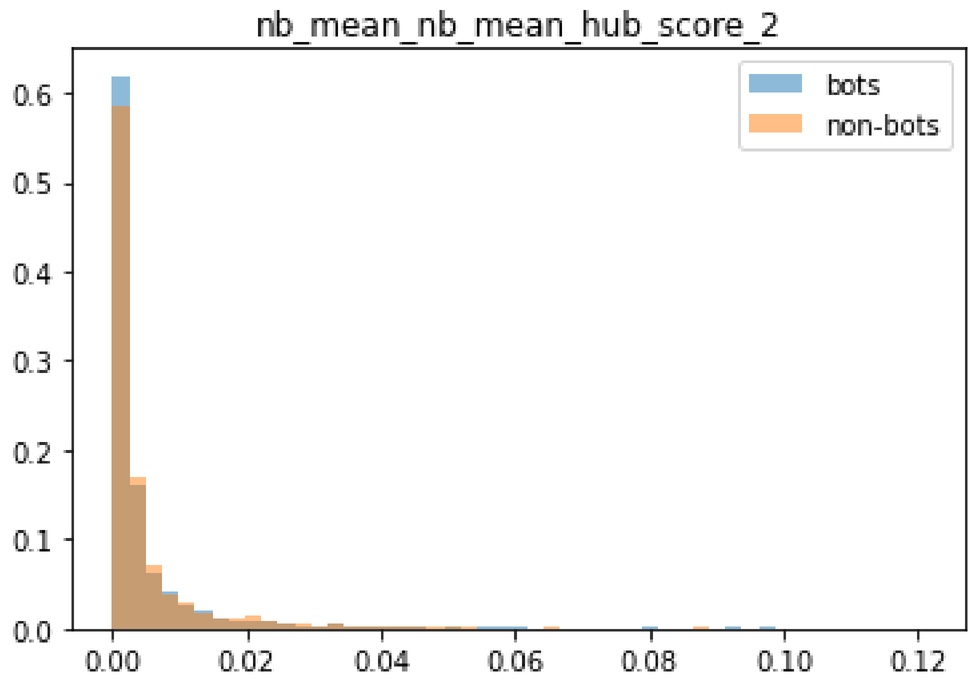
nb_mean_nb_mean_hub_score_2
bots
non-bots
0.0
0.1
0.2
0.3
0.4
0.5
0.6
0.00
0.02
0.04
0.06
0.08
0.10
0.12

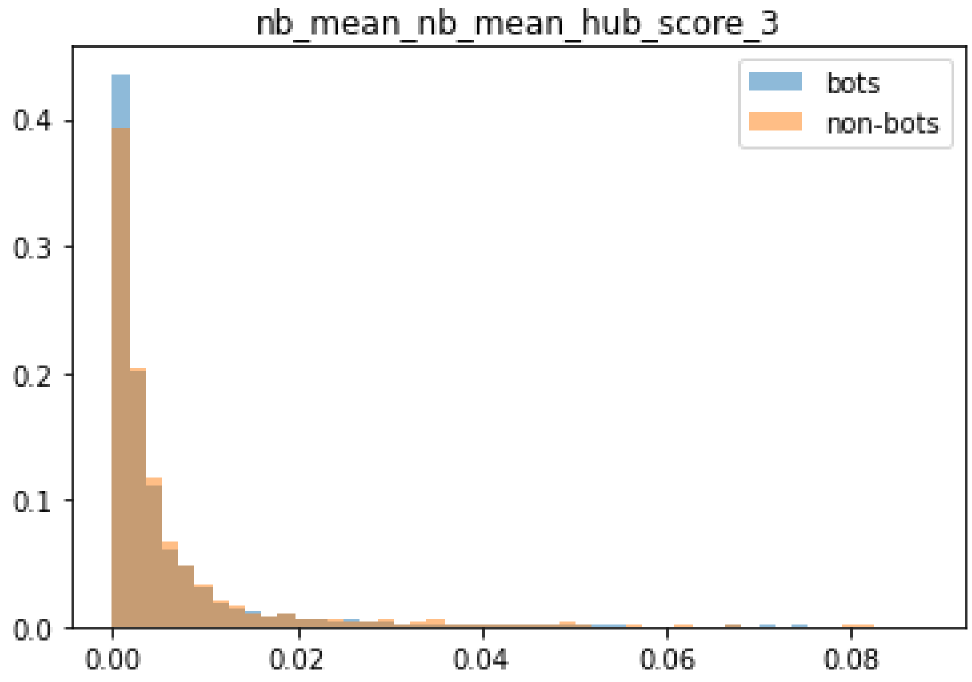
nb_mean_nb_mean_hub_score_3
bots
non-bots
0.0
0.1
0.2
0.3
0.4
0.00
0.02
0.04
0.06
0.08

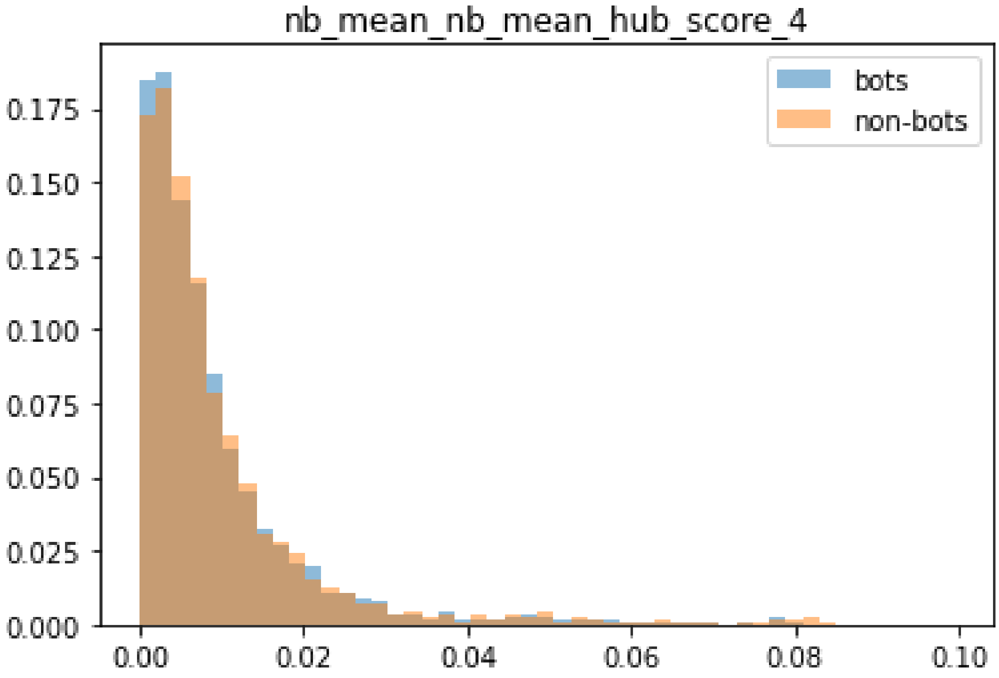
nb_mean_nb_mean_hub_score_4
bots
non-bots
0.175
0.150
0.125
0.100
0.075
0.050
0.025
0.000
0.00
0.02
0.04
0.06
0.08
0.10

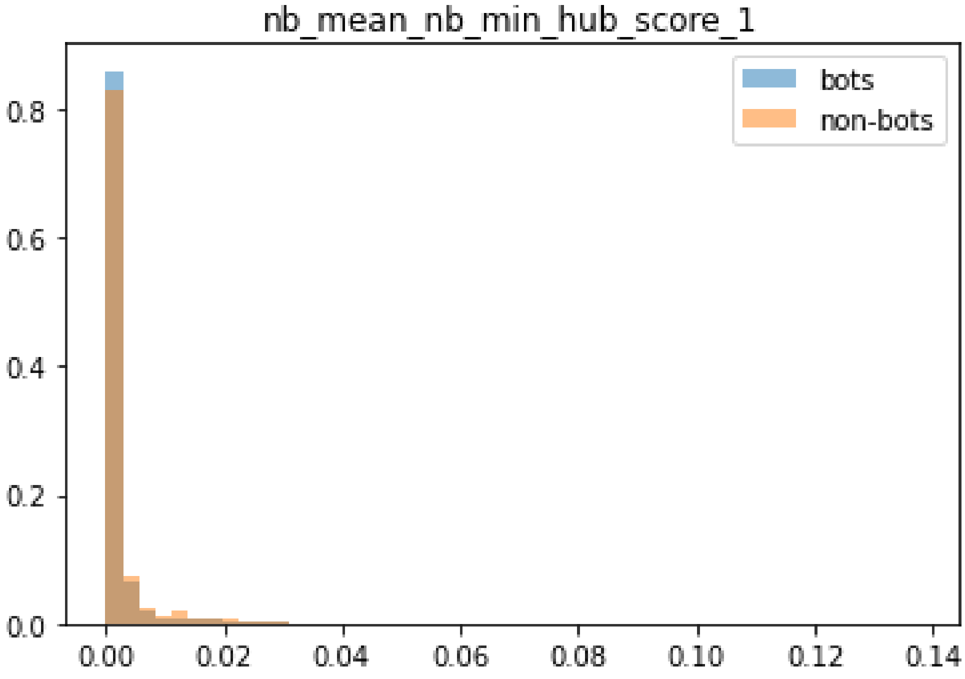
nb_mean_nb_min_hub_score_1
bots
non-bots
0.8
0.6
0.4
0.2
0.0
0.00
0.02
0.04
0.06
0.08
0.10
0.12
0.14

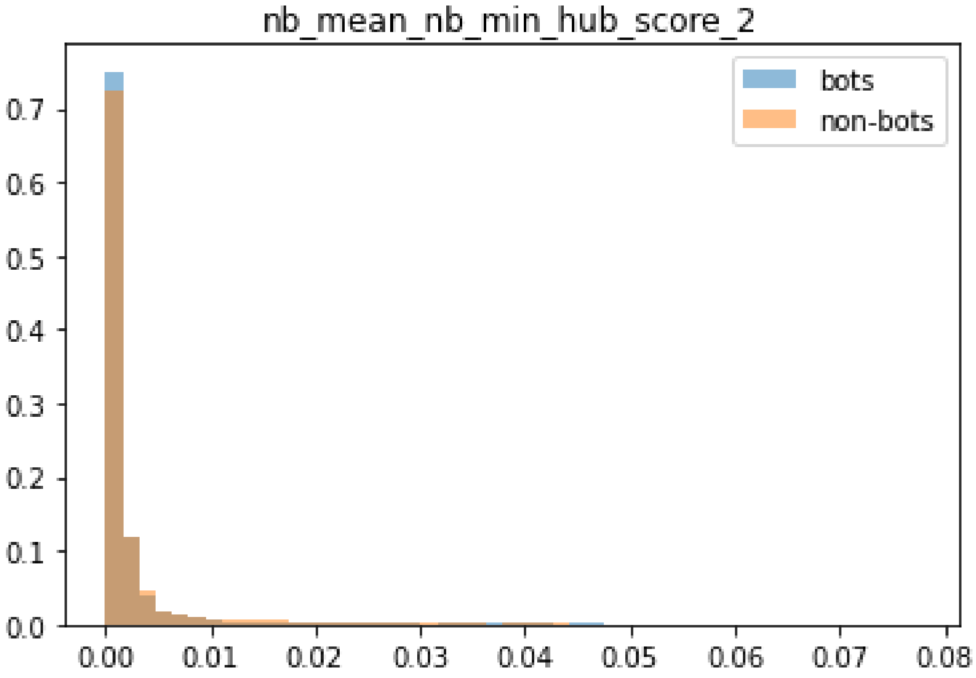

nb_mean_nb_min_hub_score_2
bots
non-bots
0.0
0.1
0.2
0.3
0.4
0.5
0.6
0.7
0.00
0.01
0.02
0.03
0.04
0.05
0.06
0.07
0.08

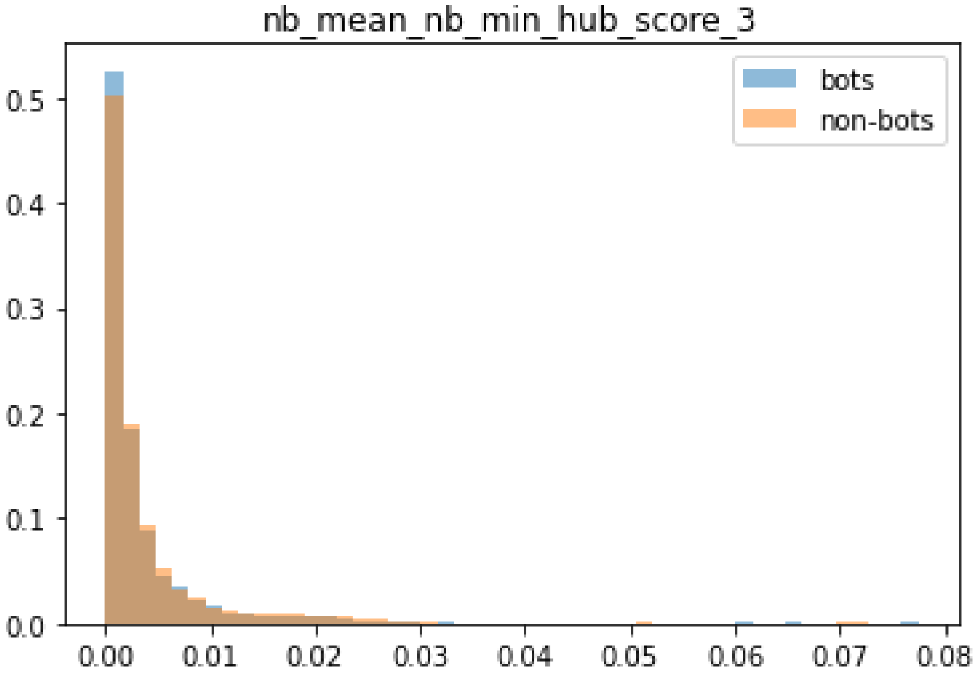

nb_mean_nb_min_hub_score_3
bots
non-bots
0.0
0.1
0.2
0.3
0.4
0.5
0.00
0.01
0.02
0.03
0.04
0.05
0.06
0.07
0.08

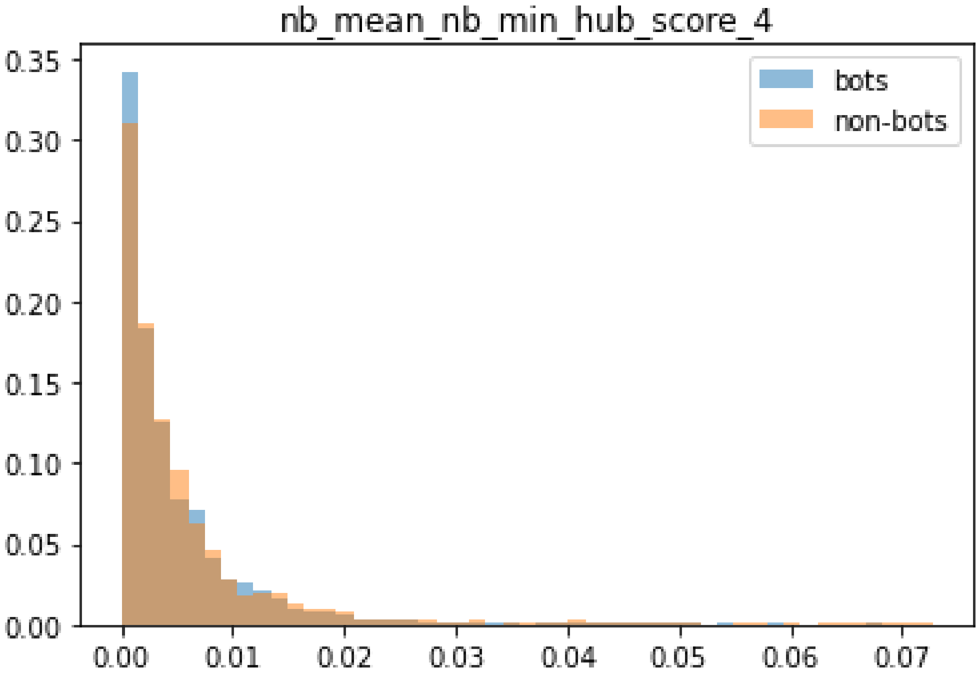
nb_mean_nb_min_hub_score_4
bots
non-bots
0.35
0.30
0.25
0.20
0.15
0.10
0.05
0.00
0.00
0.01
0.02
0.03
0.04
0.05
0.06
0.07

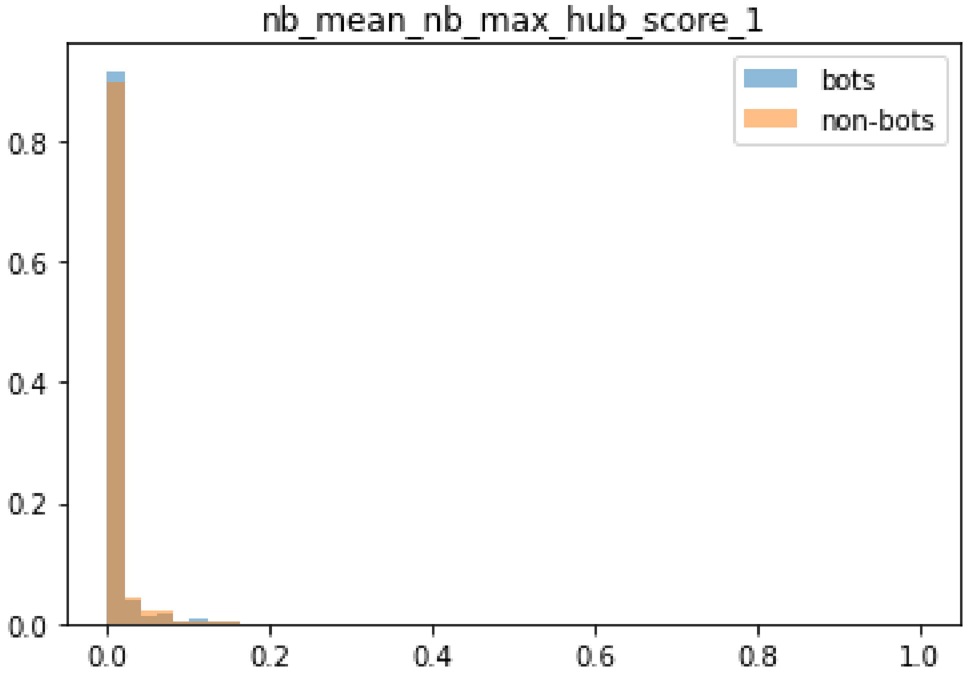
nb_mean_nb_max_hub_score_1
bots
non-bots
0.8
0.6
0.4
0.2
0.0
0.0
0.2
0.4
0.6
0.8
1.0

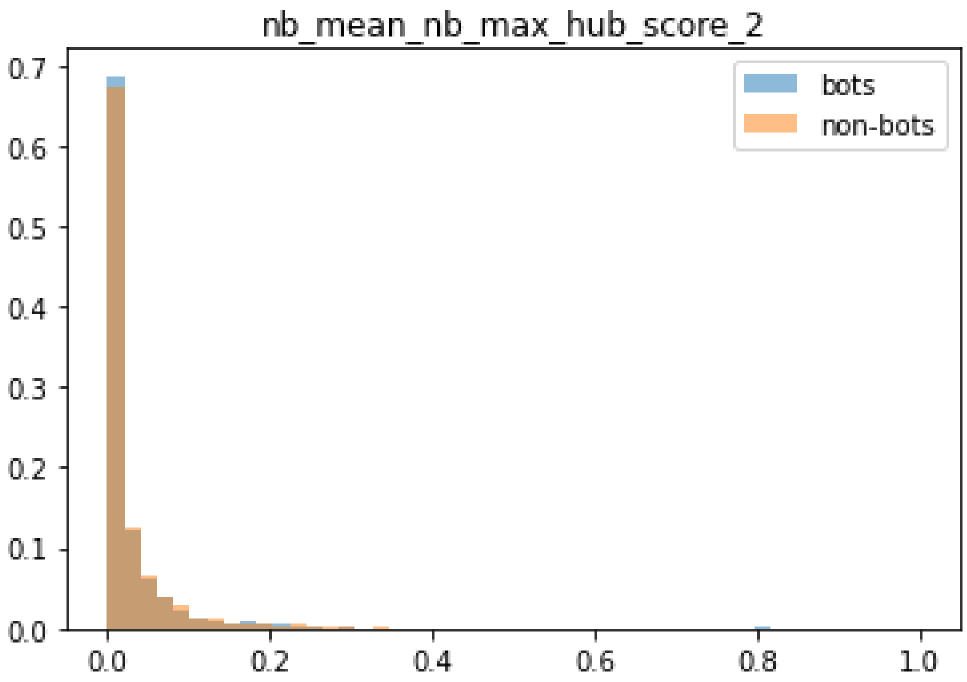

nb_mean_nb_max_hub_score_2
bots
non-bots
0.0
0.1
0.2
0.3
0.4
0.5
0.6
0.7
0.0
0.2
0.4
0.6
0.8
1.0

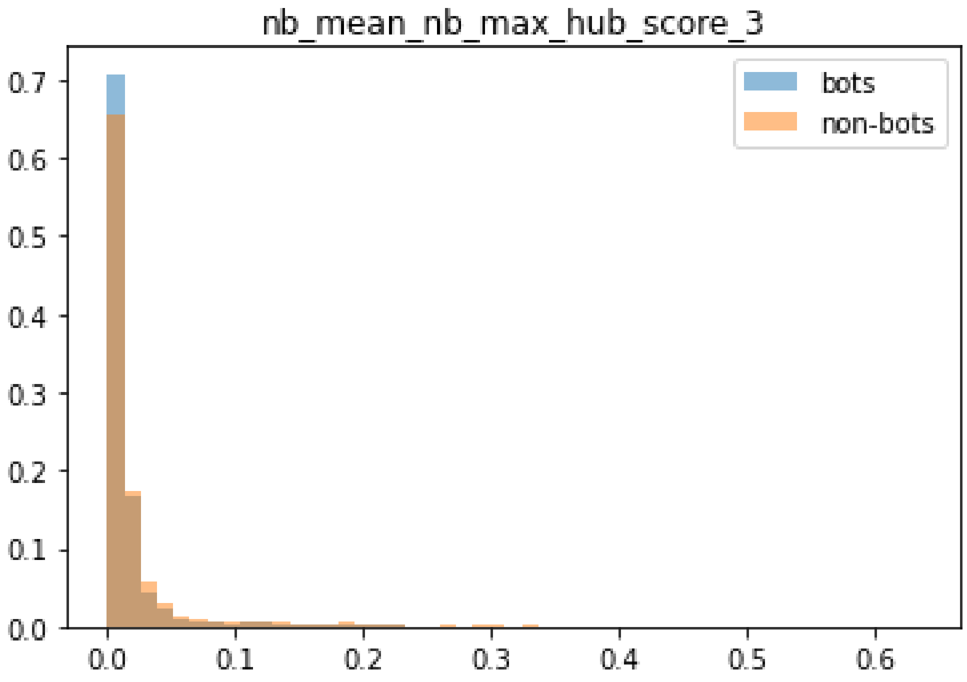

nb_mean_nb_max_hub_score_3
bots
non-bots
0.0
0.1
0.2
0.3
0.4
0.5
0.6
0.7
0.0
0.1
0.2
0.3
0.4
0.5
0.6

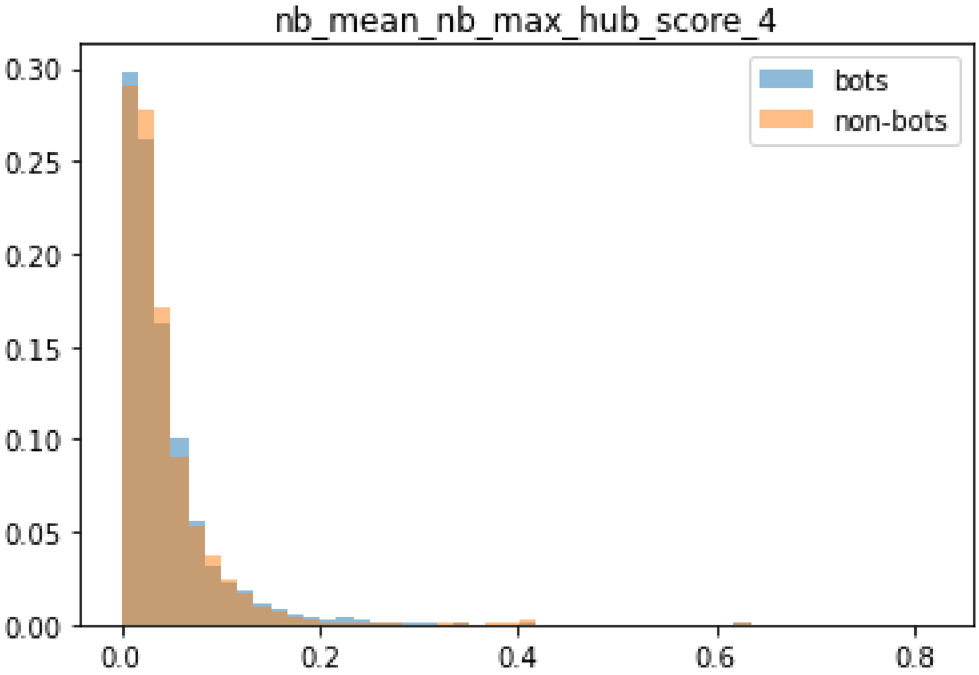
nb_mean_nb_max_hub_score_4
bots
non-bots
0.30
0.25
0.20
0.15
0.10
0.05
0.00
0.0
0.2
0.4
0.6
0.8

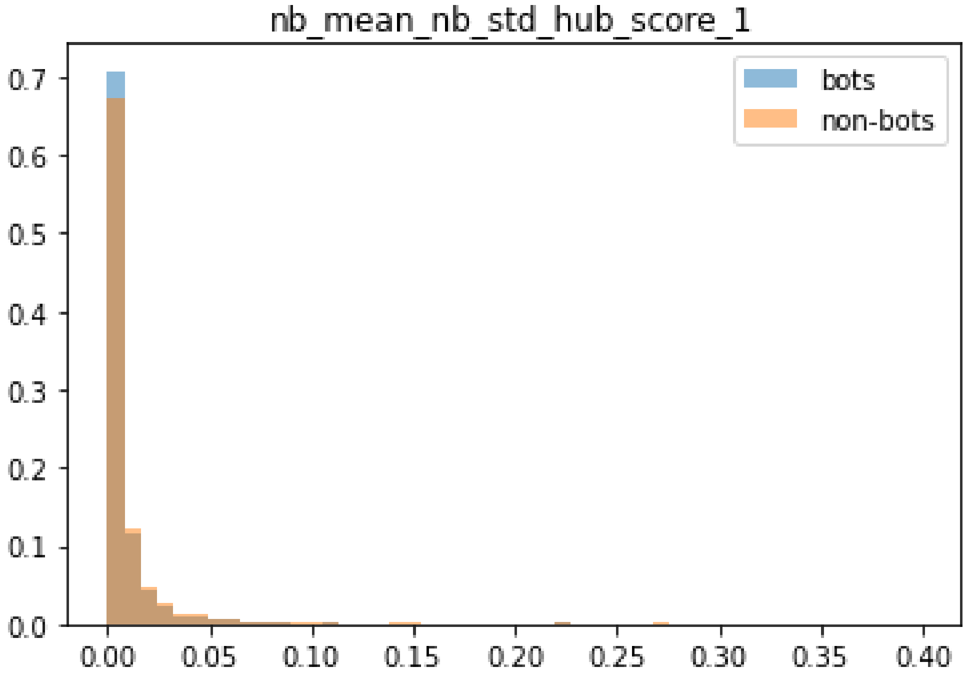
nb_mean_nb_std_hub_score_1
bots
non-bots
0.7
0.6
0.5
0.4
0.3
0.2
0.1
0.0
0.00
0.05
0.10
0.15
0.20
0.25
0.30
0.35
0.40

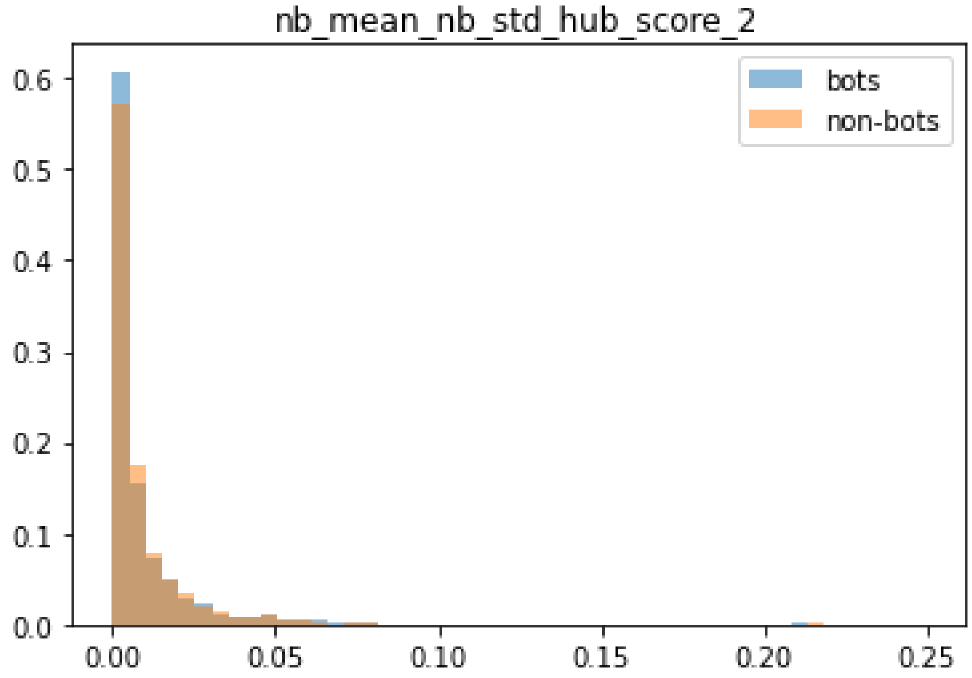
nb_mean_nb_std_hub_score_2
bots
non-bots
0.0
0.1
0.2
0.3
0.4
0.5
0.6
0.00
0.05
0.10
0.15
0.20
0.25

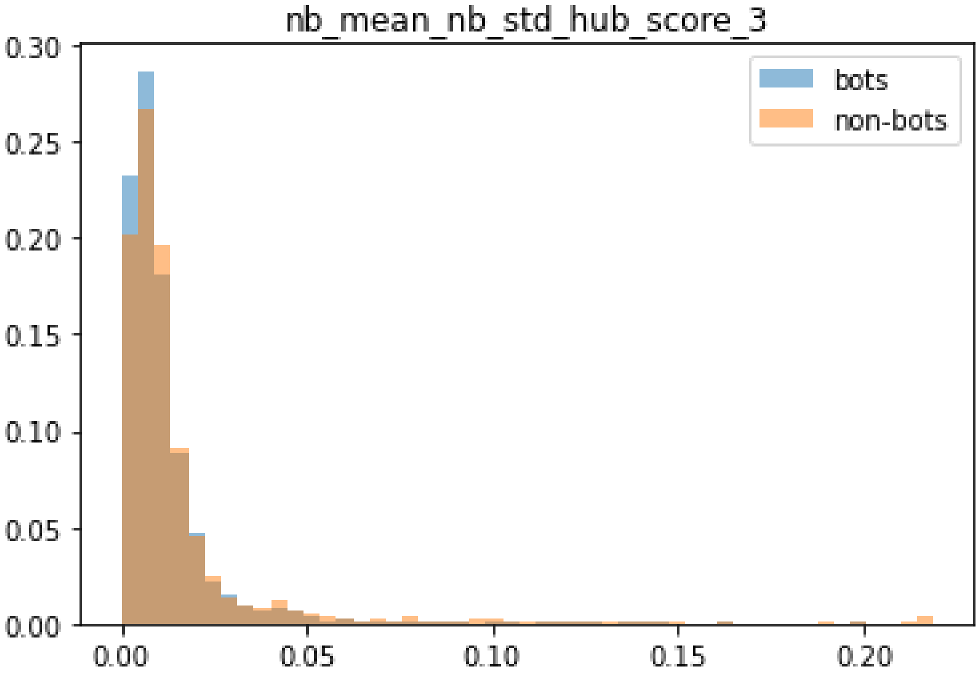
nb_mean_nb_std_hub_score_3
bots
non-bots
0.00
0.05
0.10
0.15
0.20
0.25
0.30
0.00
0.05
0.10
0.15
0.20

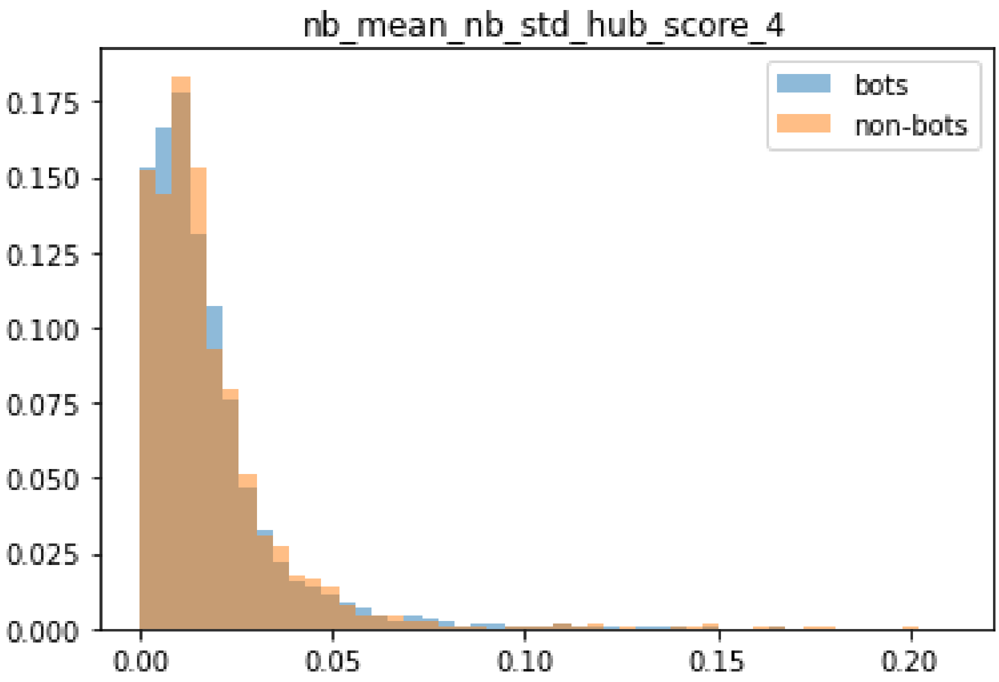
nb_mean_nb_std_hub_score_4
bots
non-bots
0.000
0.025
0.050
0.075
0.100
0.125
0.150
0.175
0.00
0.05
0.10
0.15
0.20

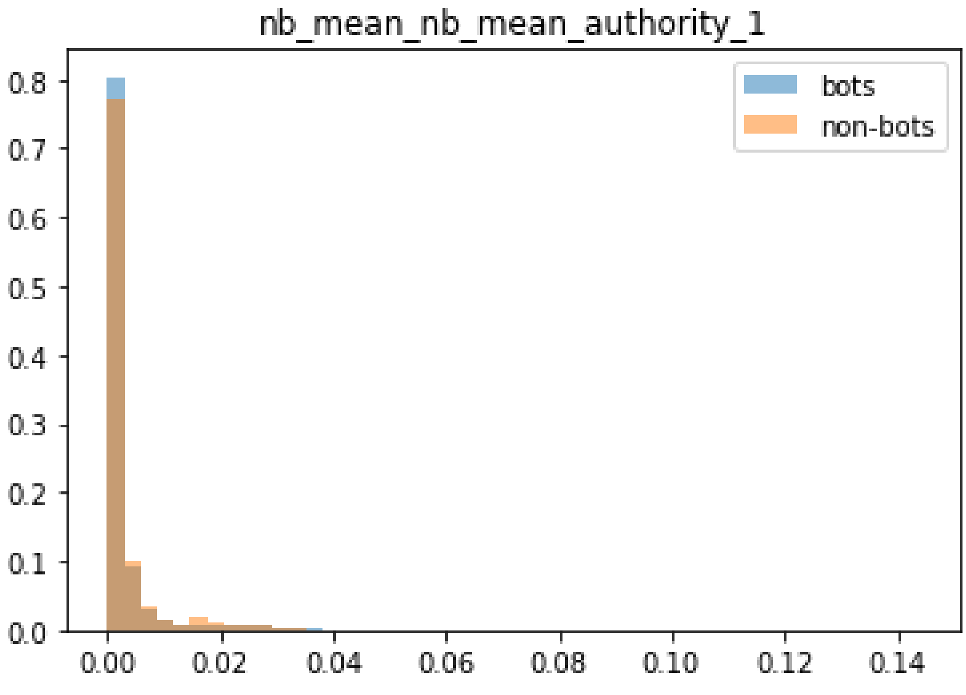
nb_mean_nb_mean_authority_1
bots
non-bots
0.0
0.1
0.2
0.3
0.4
0.5
0.6
0.7
0.8
0.00
0.02
0.04
0.06
0.08
0.10
0.12
0.14

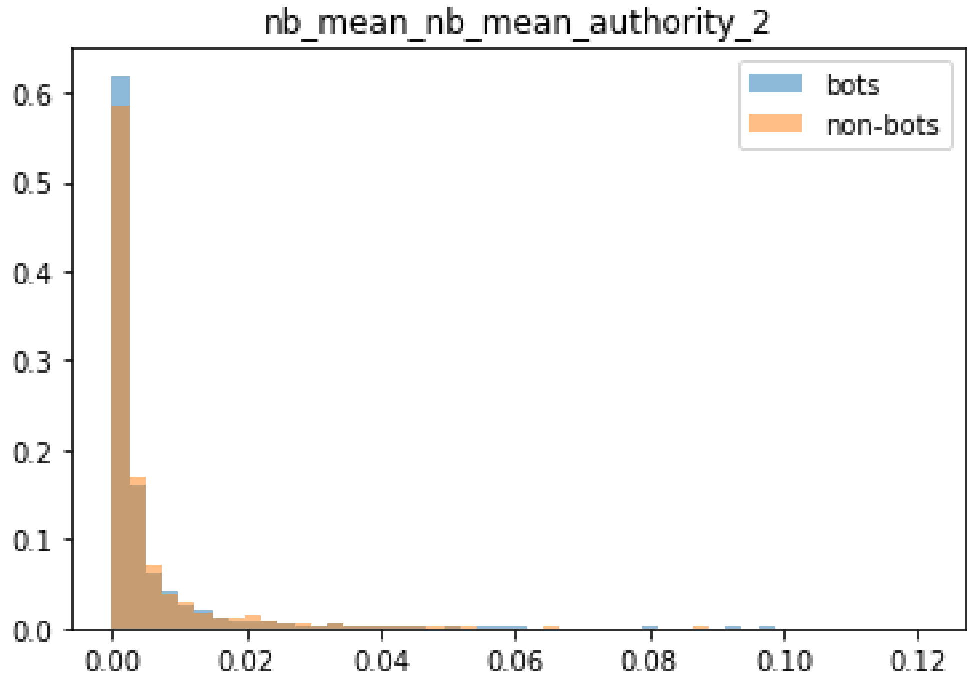
nb_mean_nb_mean_authority_2
bots
non-bots
0.6
0.5
0.4
0.3
0.2
0.1
0.0
0.00
0.02
0.04
0.06
0.08
0.10
0.12

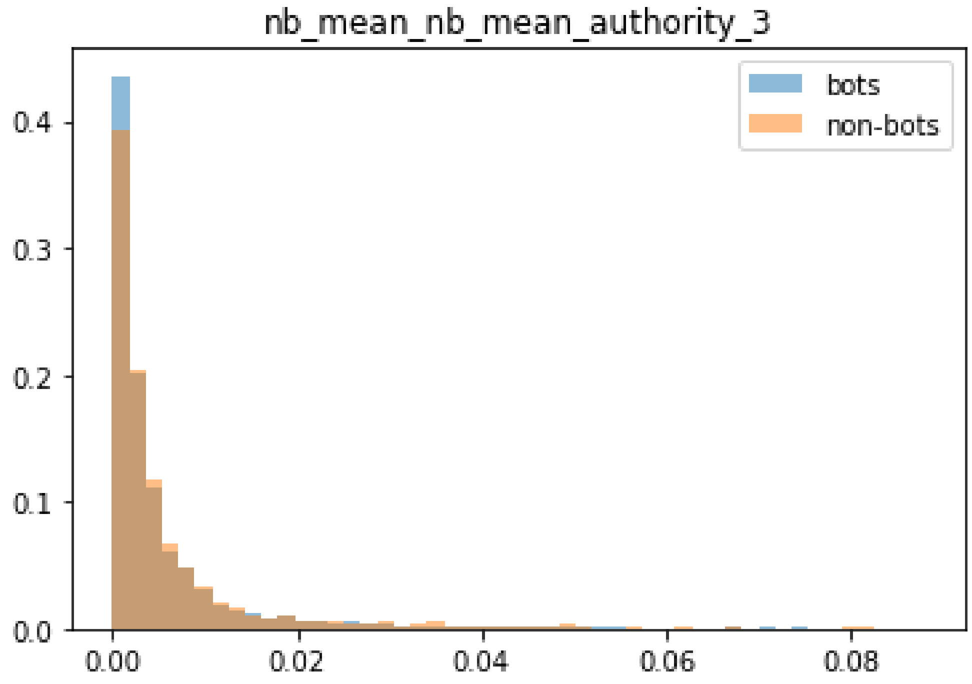
nb_mean_nb_mean_authority_3
bots
non-bots
0.4
0.3
0.2
0.1
0.0
0.00
0.02
0.04
0.06
0.08

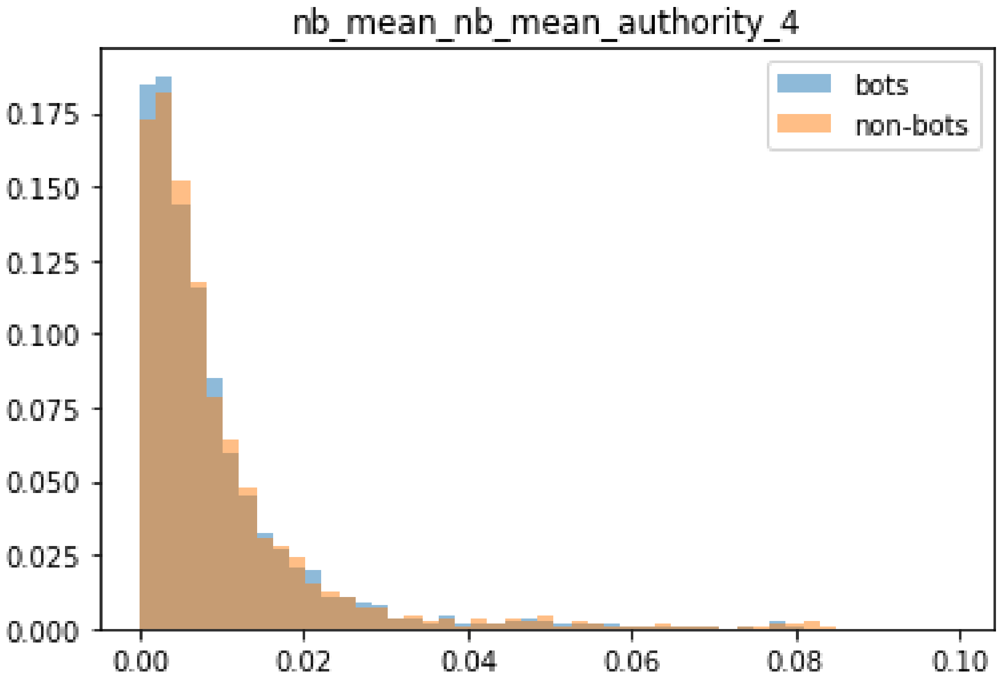
nb_mean_nb_mean_authority_4
bots
non-bots
0.175
0.150
0.125
0.100
0.075
0.050
0.025
0.000
0.00
0.02
0.04
0.06
0.08
0.10

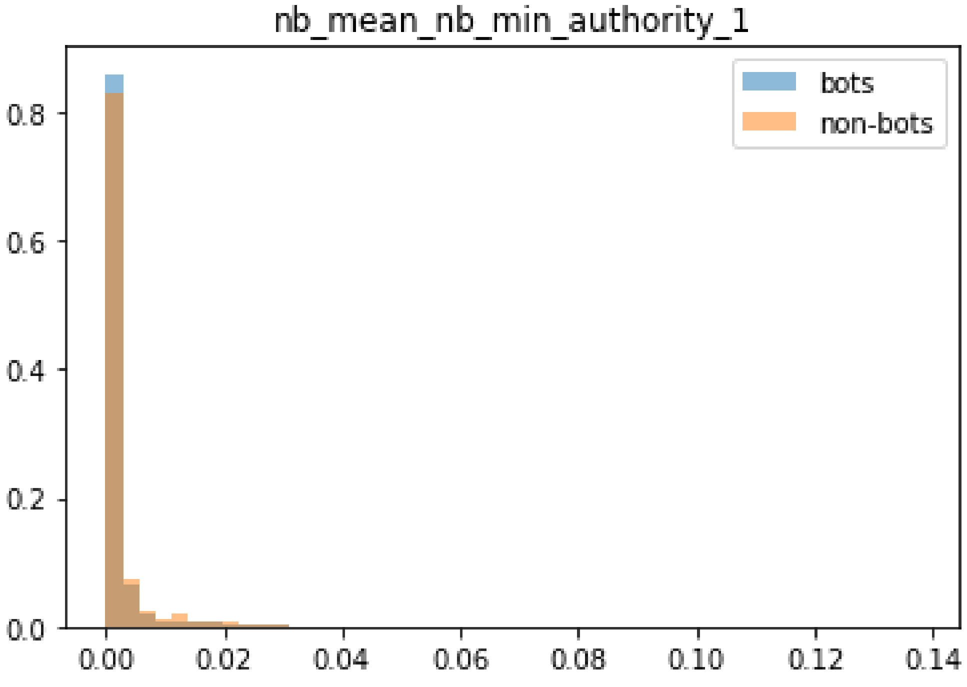
nb_mean_nb_min_authority_1
bots
non-bots
0.8
0.6
0.4
0.2
0.0
0.00
0.02
0.04
0.06
0.08
0.10
0.12
0.14

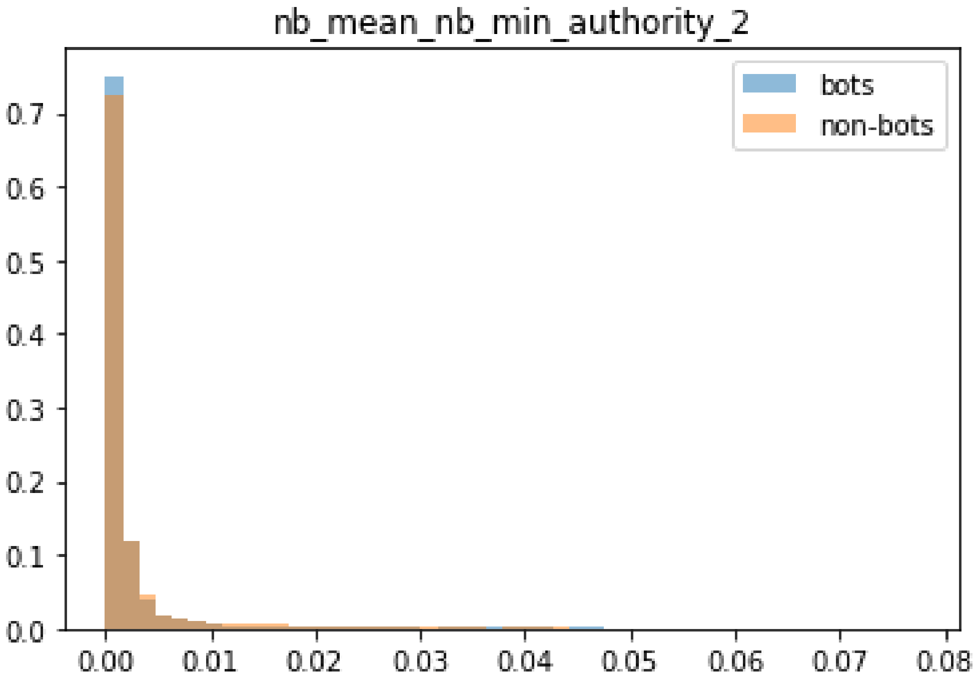
nb_mean_nb_min_authority_2
bots
non-bots
0.0
0.1
0.2
0.3
0.4
0.5
0.6
0.7
0.00
0.01
0.02
0.03
0.04
0.05
0.06
0.07
0.08

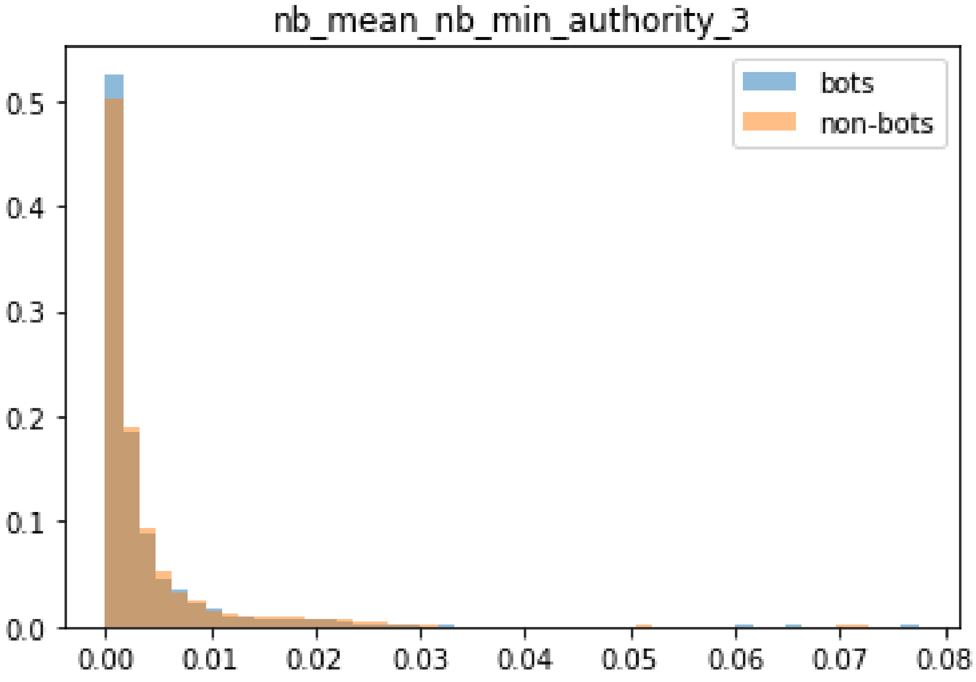
nb_mean_nb_min_authority_3
bots
non-bots
0.0
0.1
0.2
0.3
0.4
0.5
0.00
0.01
0.02
0.03
0.04
0.05
0.06
0.07
0.08

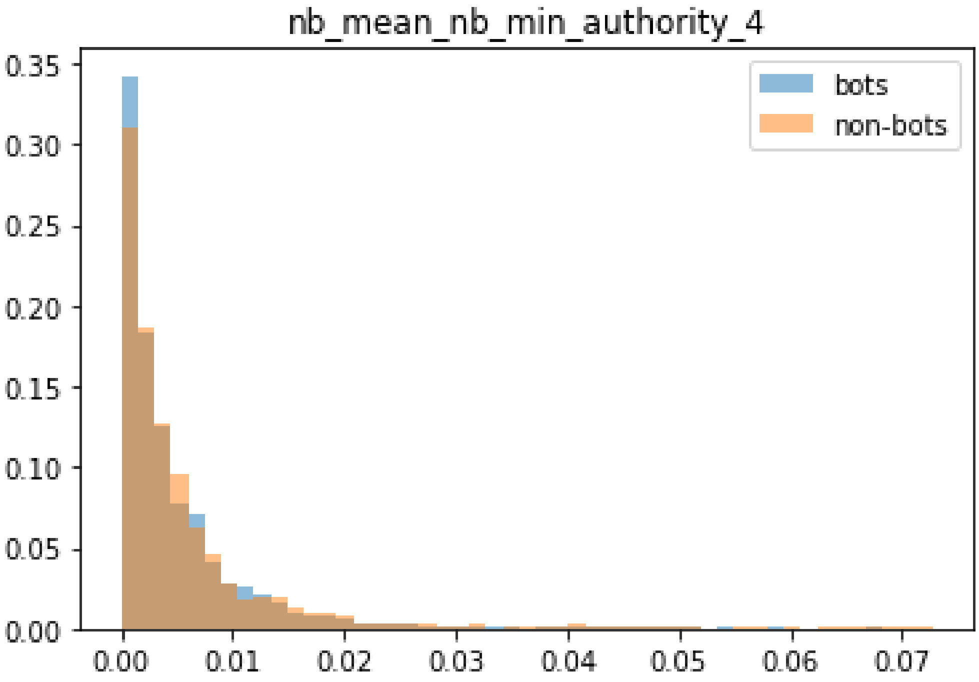
nb_mean_nb_min_authority_4
bots
non-bots
0.35
0.30
0.25
0.20
0.15
0.10
0.05
0.00
0.00
0.01
0.02
0.03
0.04
0.05
0.06
0.07

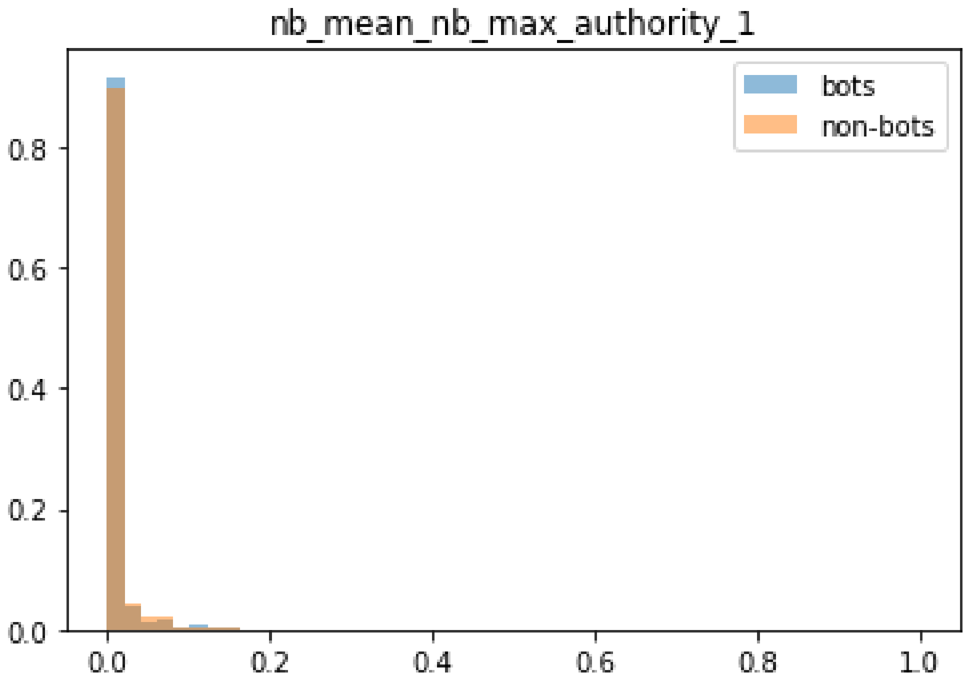
nb_mean_nb_max_authority_1
bots
non-bots
0.8
0.6
0.4
0.2
0.0
0.0
0.2
0.4
0.6
0.8
1.0

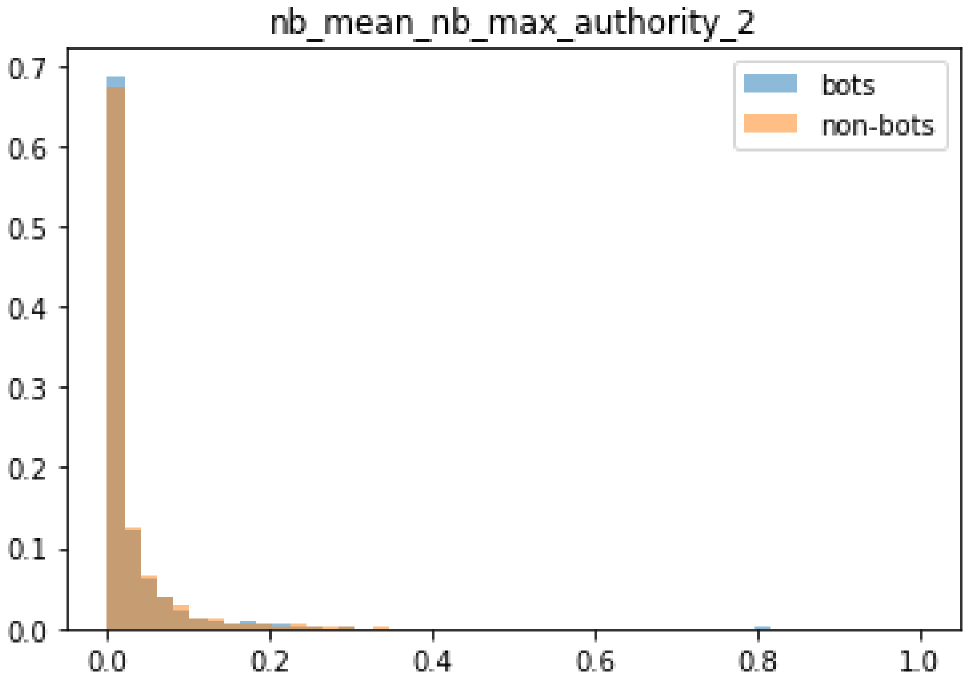
nb_mean_nb_max_authority_2
bots
non-bots
0.0
0.1
0.2
0.3
0.4
0.5
0.6
0.7
0.0
0.2
0.4
0.6
0.8
1.0

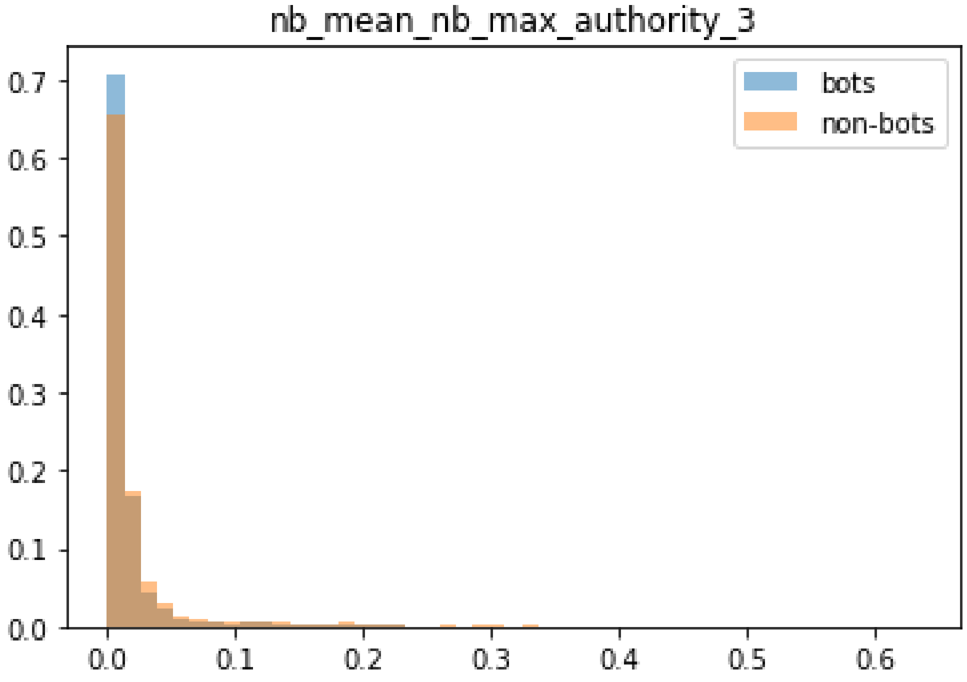
nb_mean_nb_max_authority_3
bots
non-bots
0.0
0.1
0.2
0.3
0.4
0.5
0.6
0.7
0.0
0.1
0.2
0.3
0.4
0.5
0.6

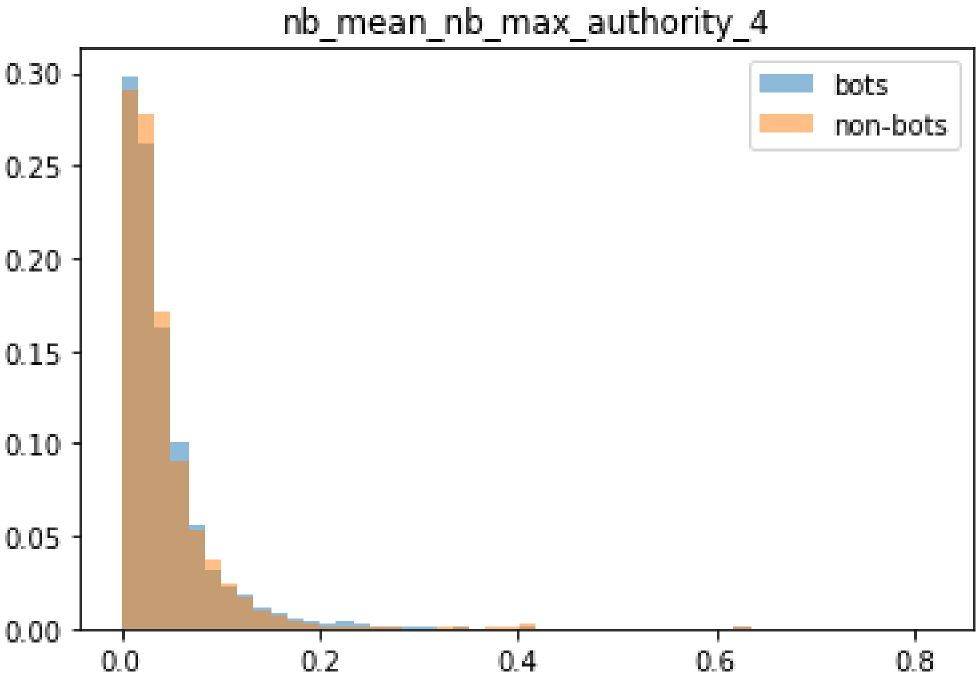
nb_mean_nb_max_authority_4
bots
non-bots
0.30
0.25
0.20
0.15
0.10
0.05
0.00
0.0
0.2
0.4
0.6
0.8

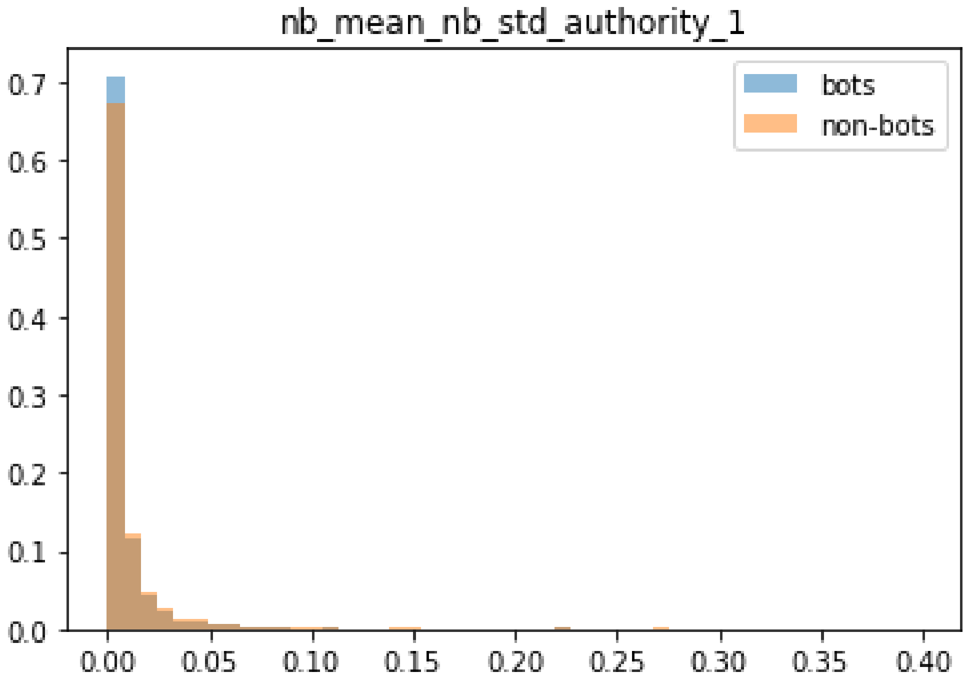
nb_mean_nb_std_authority_1
bots
non-bots
0.7
0.6
0.5
0.4
0.3
0.2
0.1
0.0
0.00
0.05
0.10
0.15
0.20
0.25
0.30
0.35
0.40

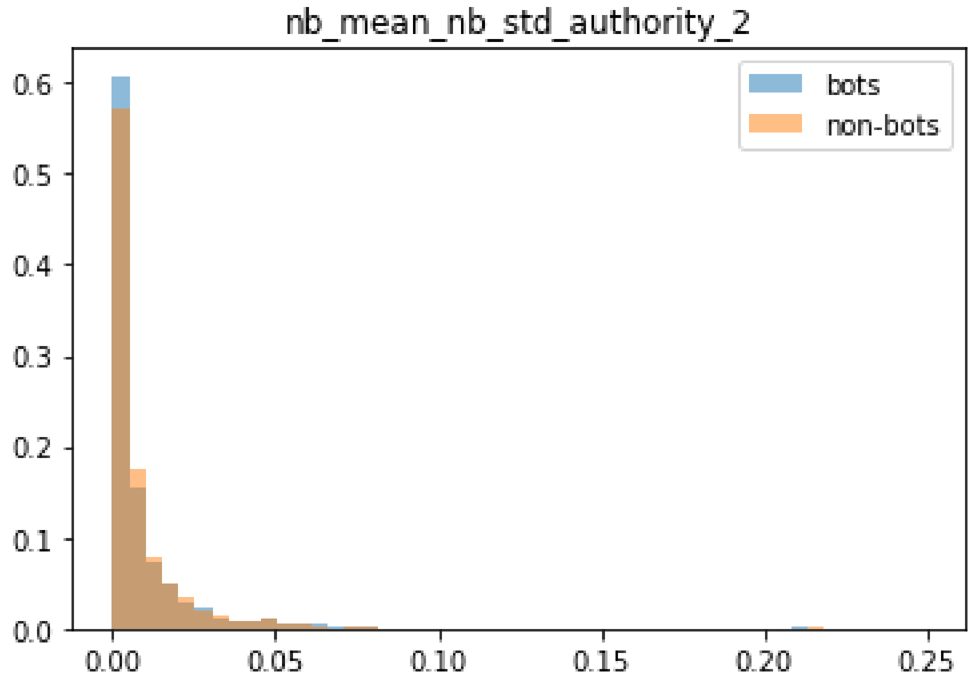
nb_mean_nb_std_authority_2
bots
non-bots
0.0
0.1
0.2
0.3
0.4
0.5
0.6
0.00
0.05
0.10
0.15
0.20
0.25

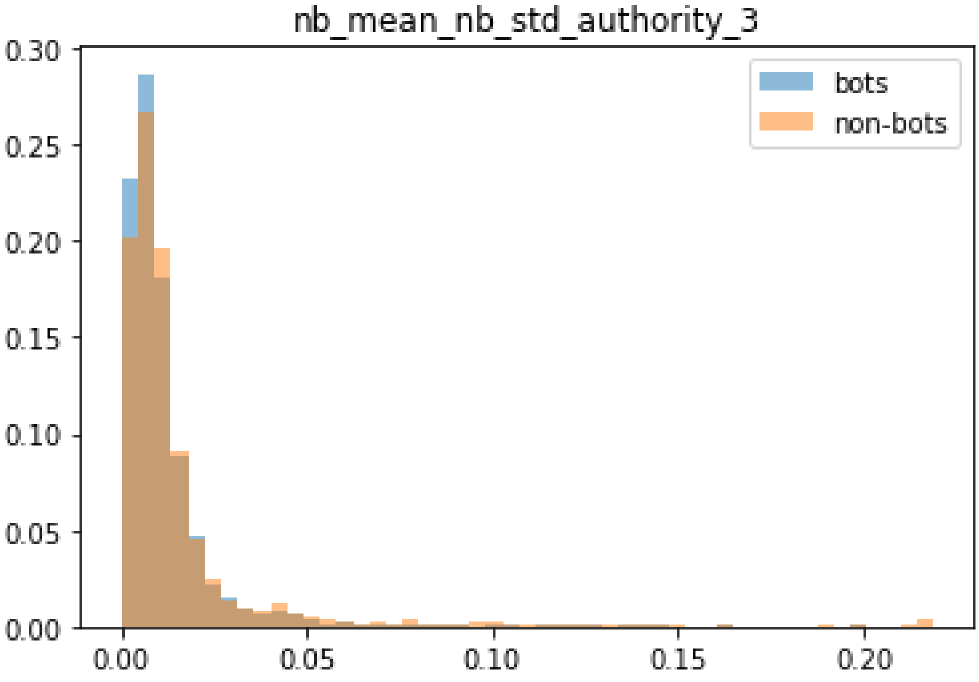
nb_mean_nb_std_authority_3
bots
non-bots
0.00
0.05
0.10
0.15
0.20
0.25
0.30
0.00
0.05
0.10
0.15
0.20

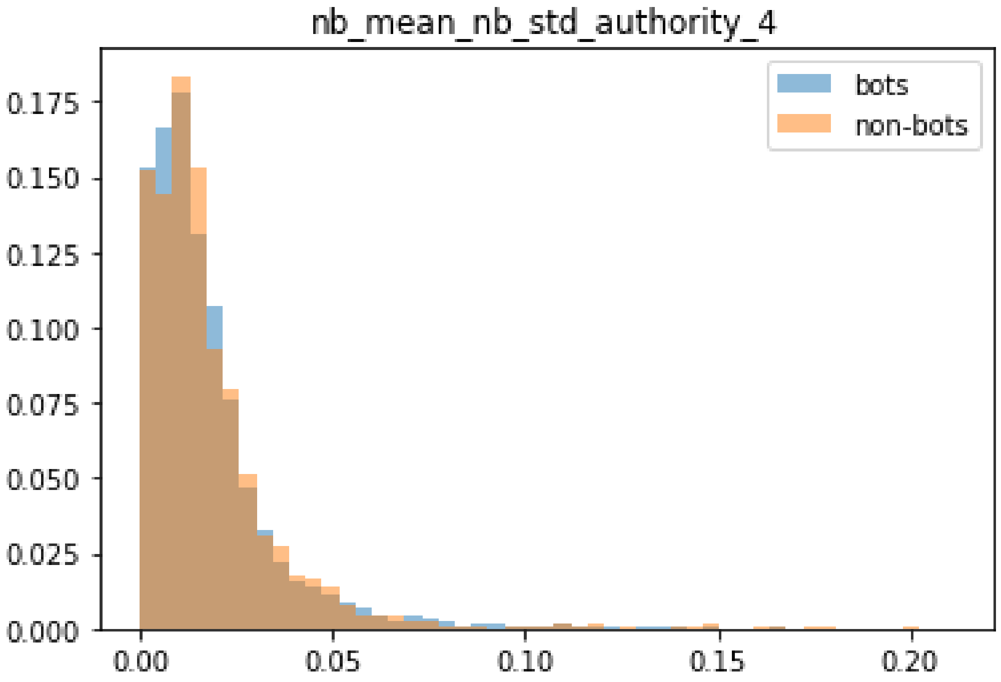
nb_mean_nb_std_authority_4
bots
non-bots
0.000
0.025
0.050
0.075
0.100
0.125
0.150
0.175
0.00
0.05
0.10
0.15
0.20

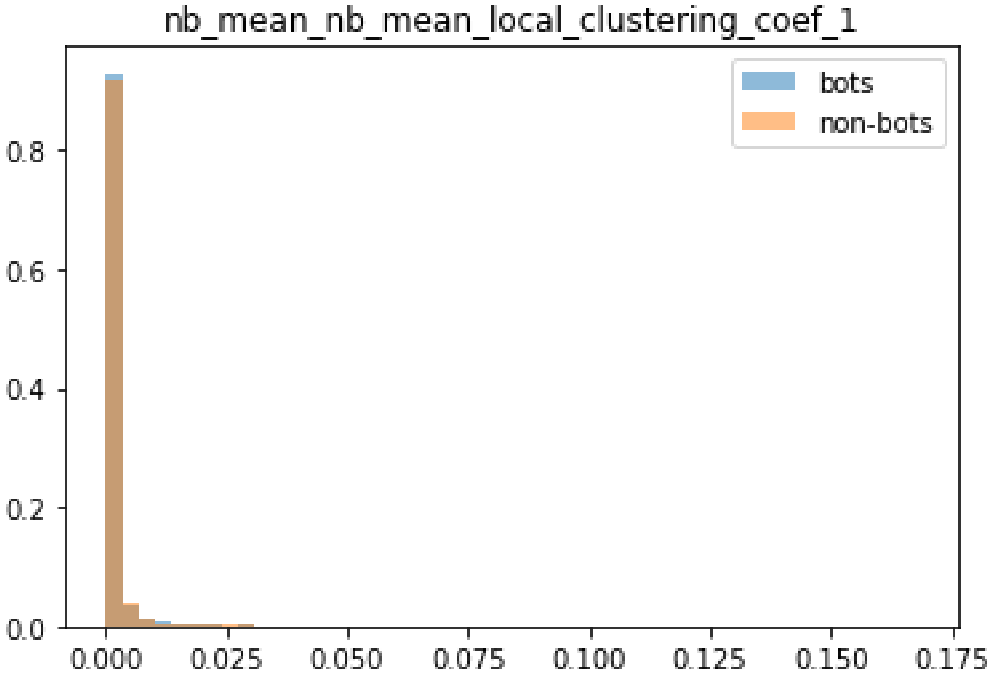
nb_mean_nb_mean_local_clustering_coef_1
bots
non-bots
0.0
0.2
0.4
0.6
0.8
0.000
0.025
0.050
0.075
0.100
0.125
0.150
0.175

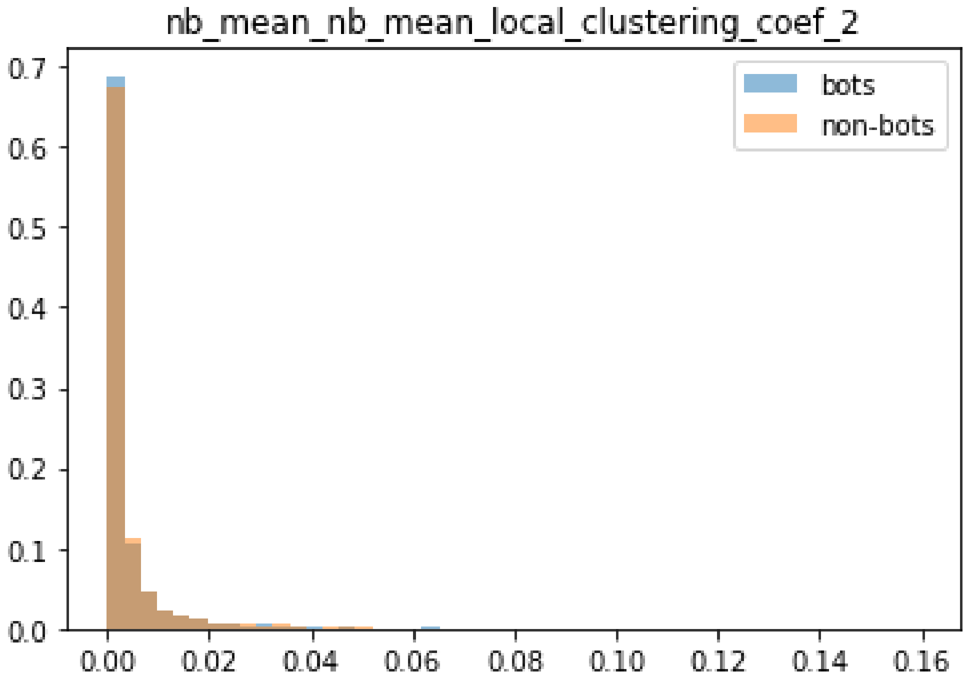

nb_mean_nb_mean_local_clustering_coef_2
bots
non-bots
0.0
0.1
0.2
0.3
0.4
0.5
0.6
0.7
0.00
0.02
0.04
0.06
0.08
0.10
0.12
0.14
0.16

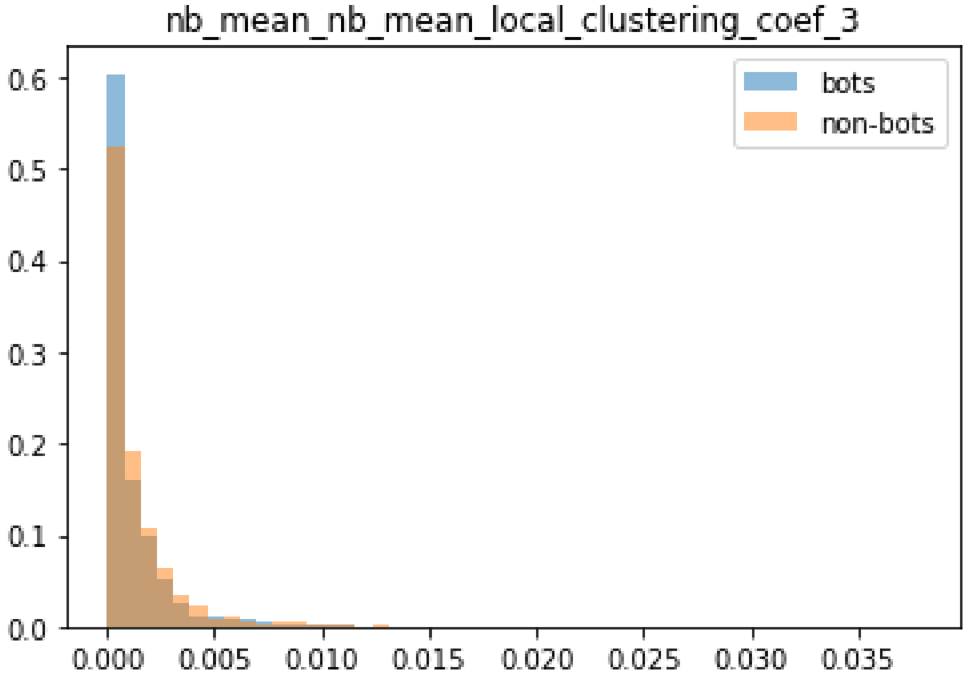

nb_mean_nb_mean_local_clustering_coef_3
bots
non-bots
0.0
0.1
0.2
0.3
0.4
0.5
0.6
0.000
0.005
0.010
0.015
0.020
0.025
0.030
0.035

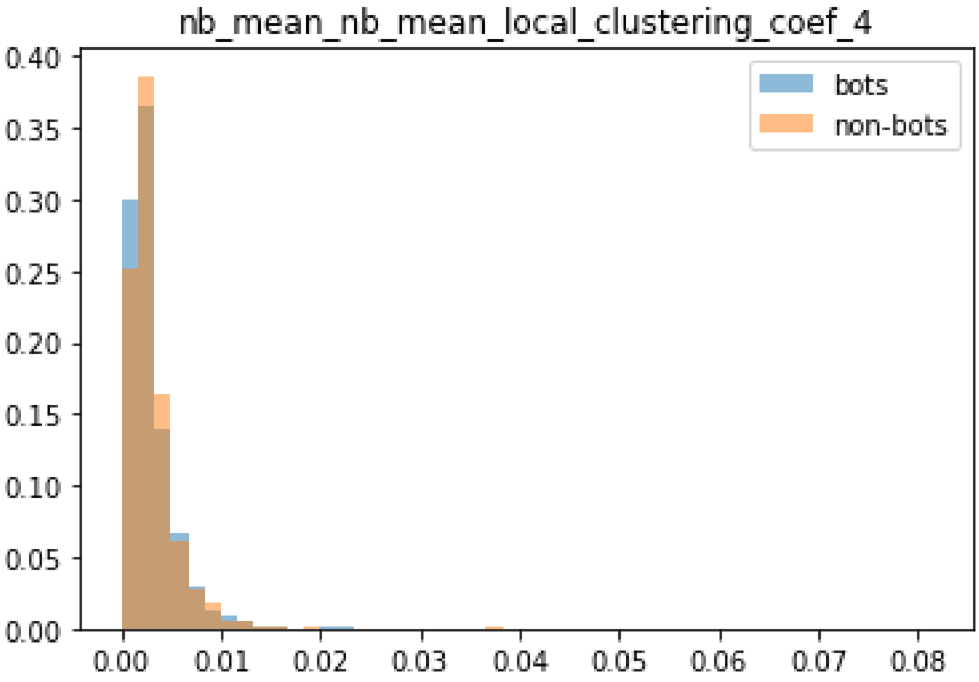
nb_mean_nb_mean_local_clustering_coef_4
bots
non-bots
0.40
0.35
0.30
0.25
0.20
0.15
0.10
0.05
0.00
0.00
0.01
0.02
0.03
0.04
0.05
0.06
0.07
0.08

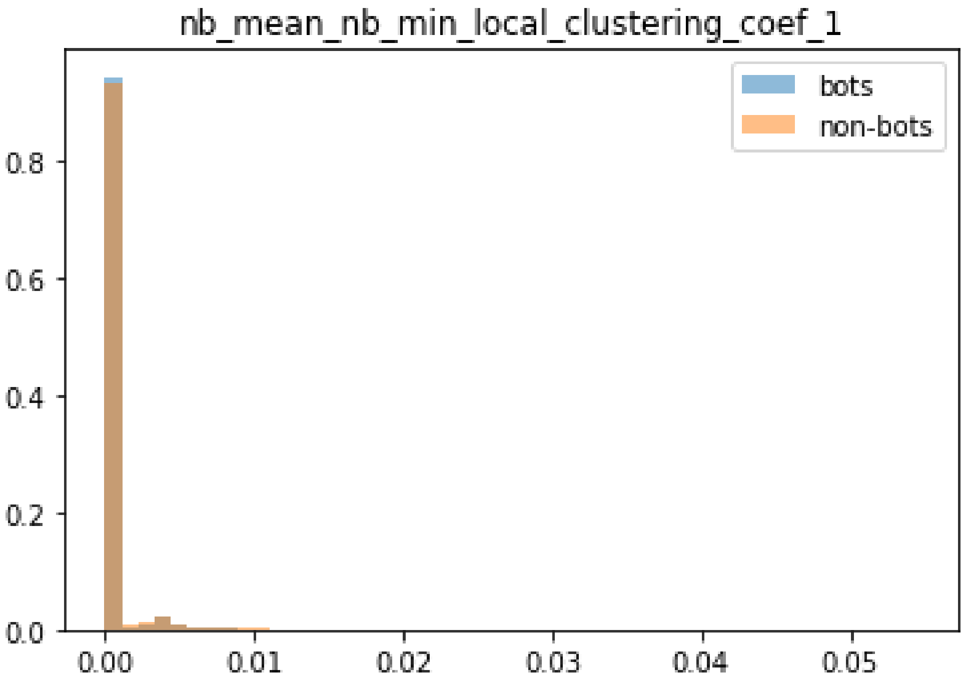
nb_mean_nb_min_local_clustering_coef_1
bots
non-bots
0.8
0.6
0.4
0.2
0.0
0.00
0.01
0.02
0.03
0.04
0.05

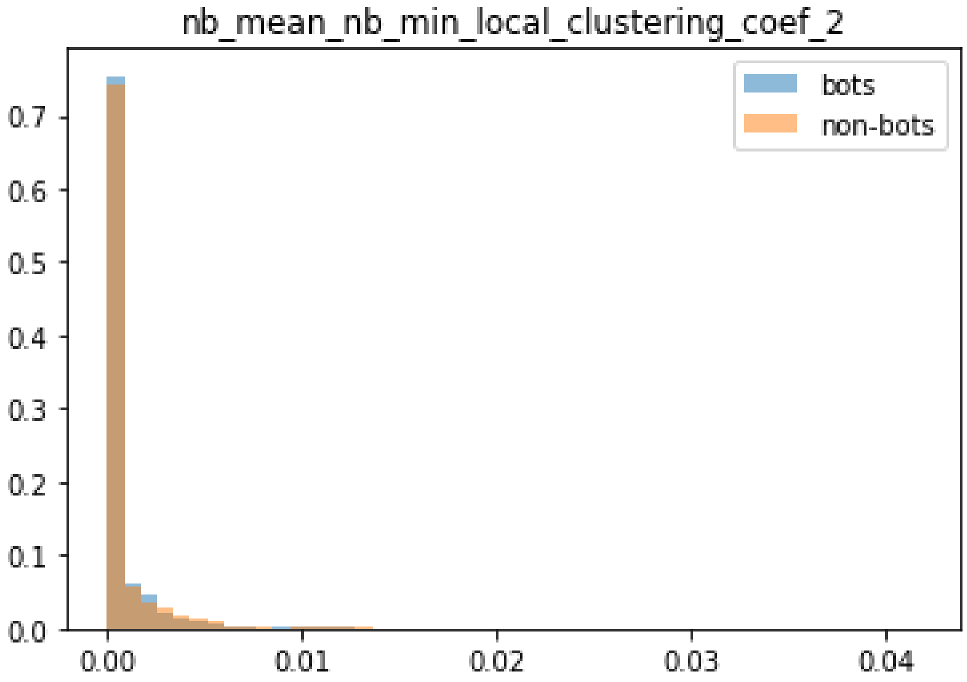
nb_mean_nb_min_local_clustering_coef_2
bots
non-bots
0.0
0.1
0.2
0.3
0.4
0.5
0.6
0.7
0.00
0.01
0.02
0.03
0.04

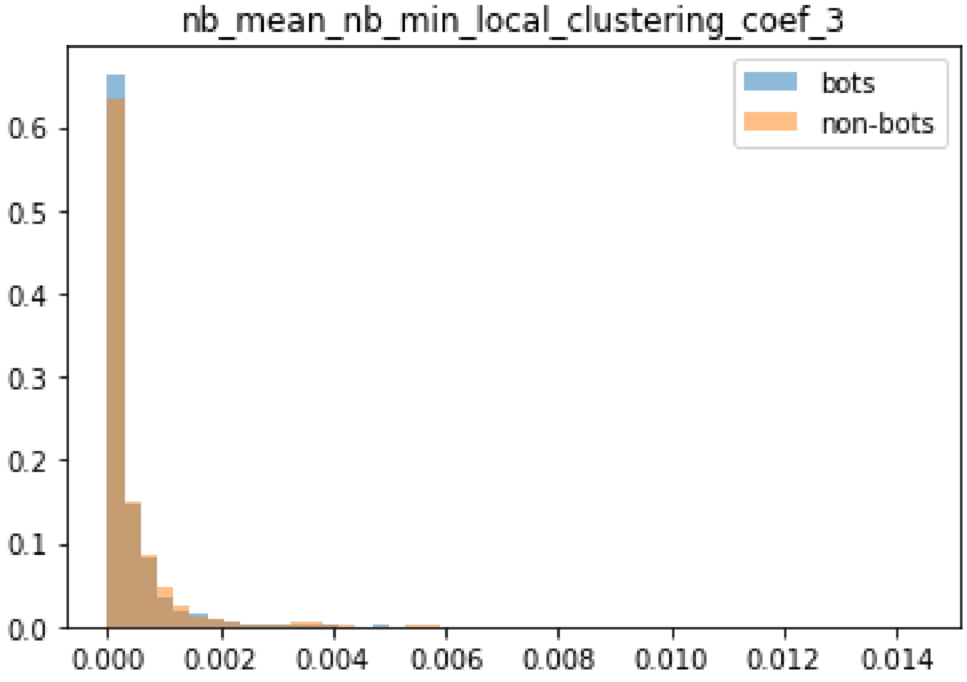
nb_mean_nb_min_local_clustering_coef_3
bots
non-bots
0.0
0.1
0.2
0.3
0.4
0.5
0.6
0.000
0.002
0.004
0.006
0.008
0.010
0.012
0.014

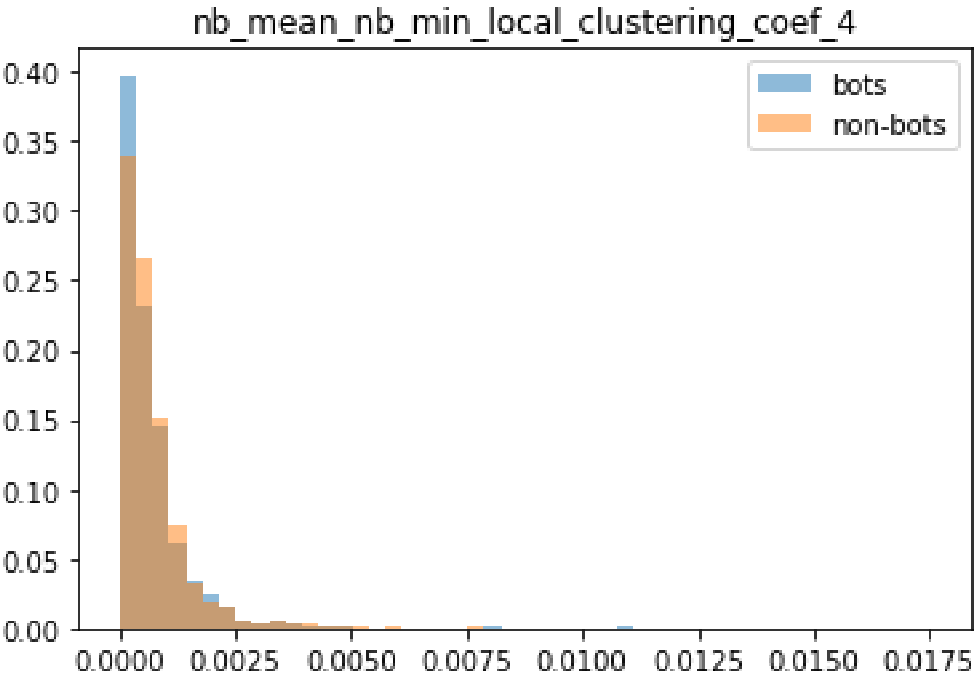
nb_mean_nb_min_local_clustering_coef_4
bots
non-bots
0.40
0.35
0.30
0.25
0.20
0.15
0.10
0.05
0.00
0.0000
0.0025
0.0050
0.0075
0.0100
0.0125
0.0150
0.0175

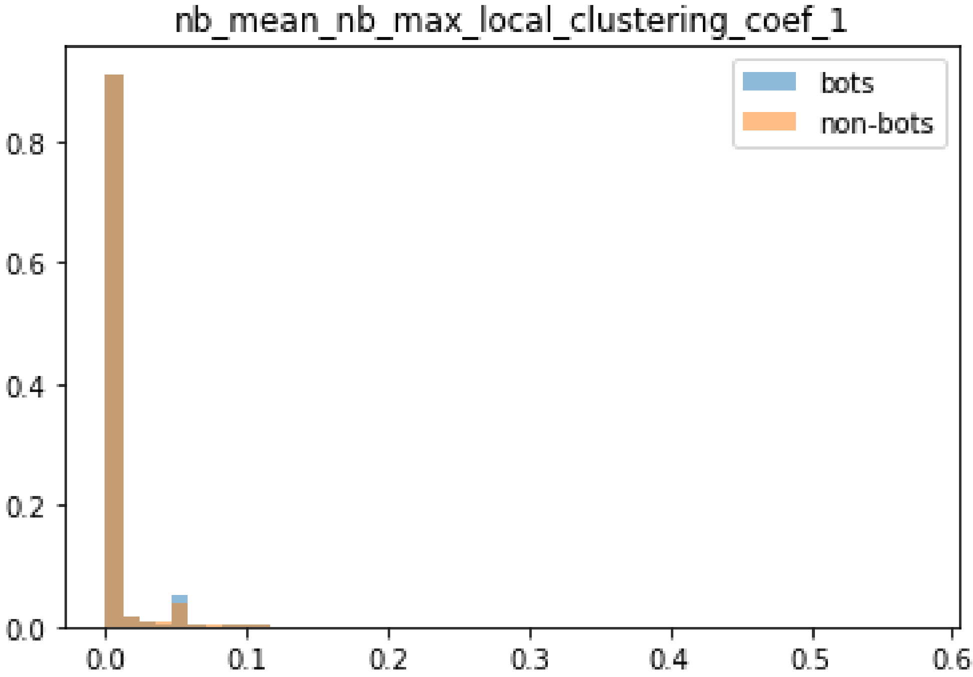
nb_mean_nb_max_local_clustering_coef_1
bots
non-bots
0.8
0.6
0.4
0.2
0.0
0.0
0.1
0.2
0.3
0.4
0.5
0.6

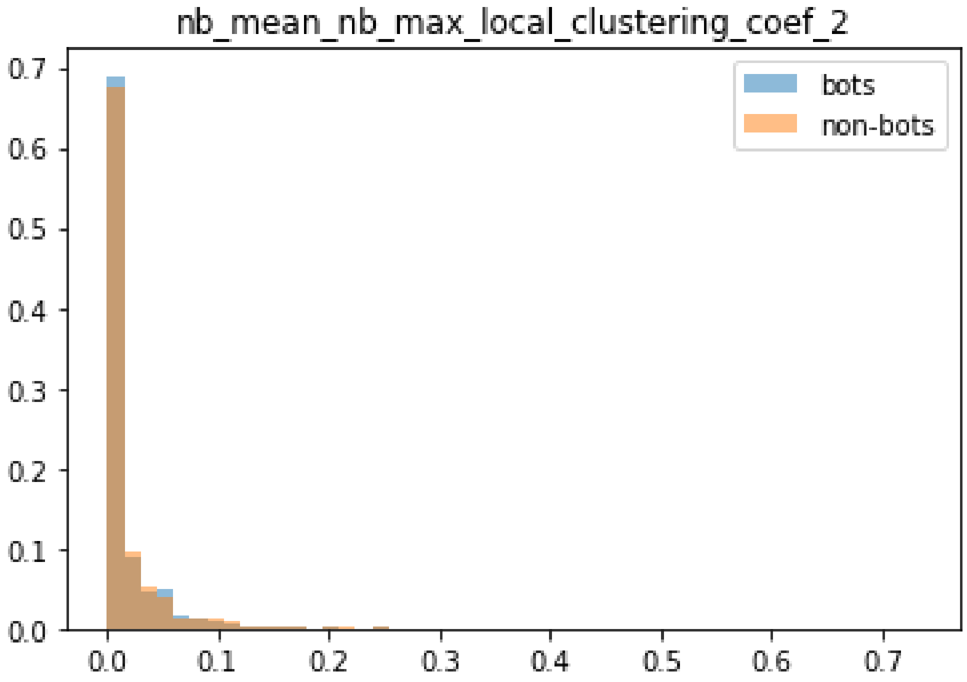
nb_mean_nb_max_local_clustering_coef_2
bots
non-bots
0.0
0.1
0.2
0.3
0.4
0.5
0.6
0.7
0.0
0.1
0.2
0.3
0.4
0.5
0.6
0.7

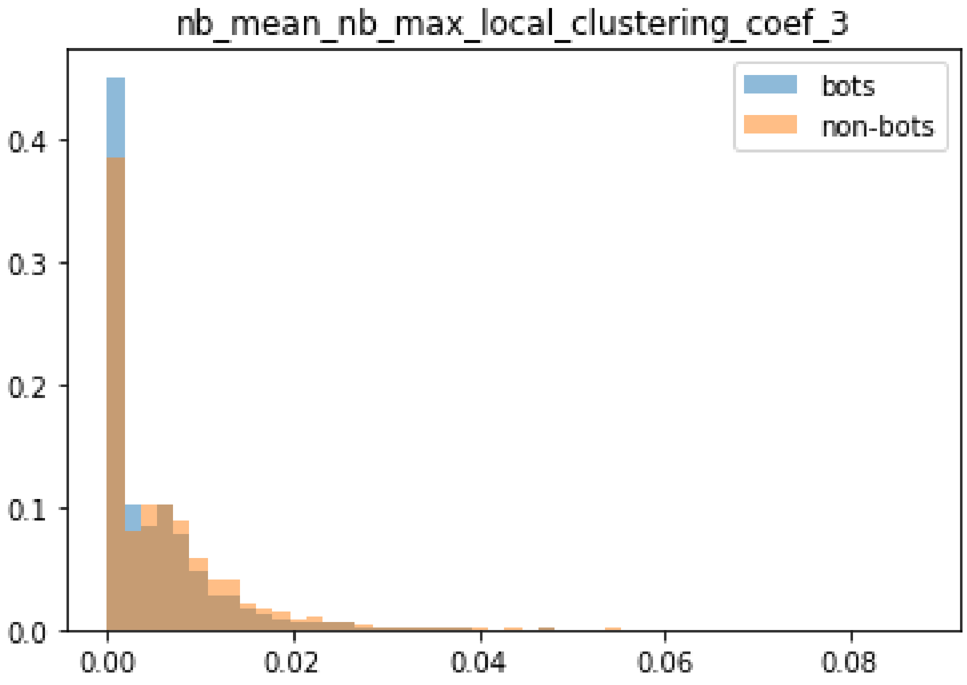
nb_mean_nb_max_local_clustering_coef_3
bots
non-bots
0.0
0.1
0.2
0.3
0.4
0.00
0.02
0.04
0.06
0.08

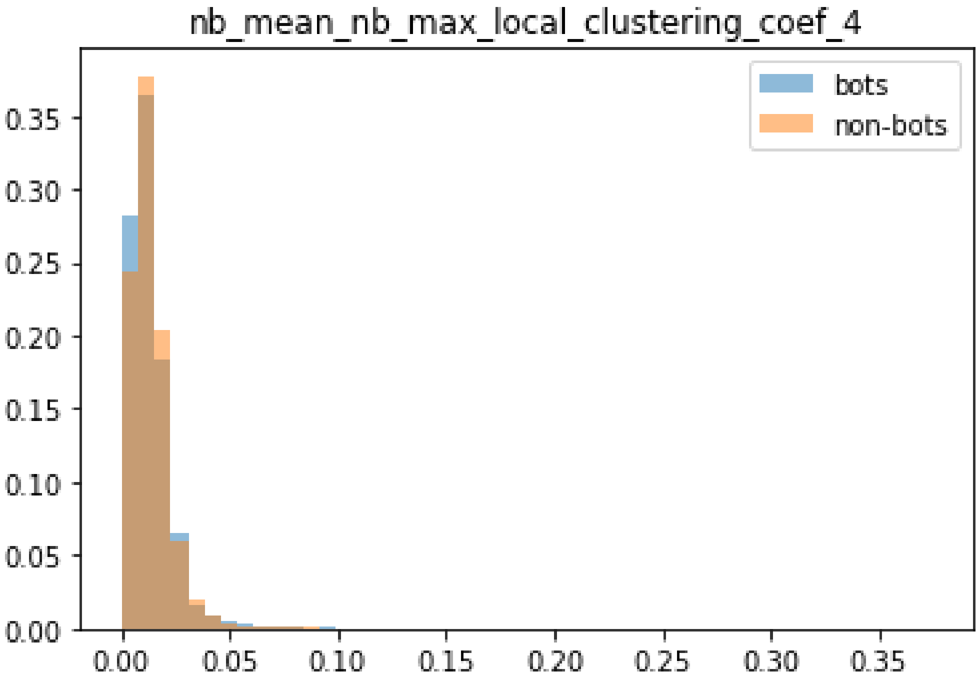
nb_mean_nb_max_local_clustering_coef_4
bots
non-bots
0.00
0.05
0.10
0.15
0.20
0.25
0.30
0.35
0.00
0.05
0.10
0.15
0.20
0.25
0.30
0.35

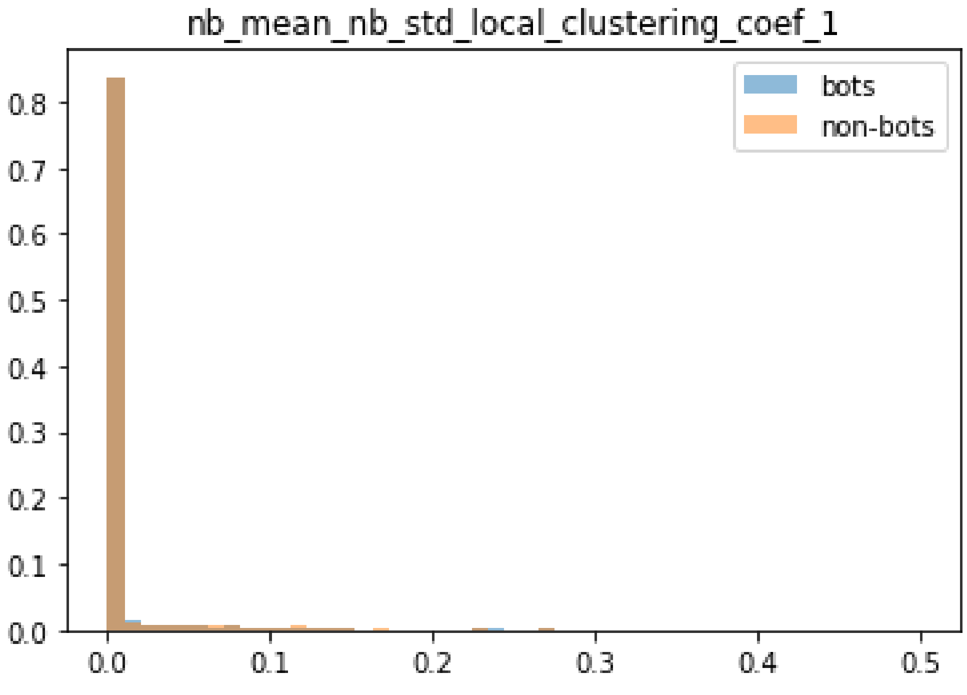
nb_mean_nb_std_local_clustering_coef_1
bots
non-bots
0.0
0.1
0.2
0.3
0.4
0.5
0.6
0.7
0.8
0.0
0.1
0.2
0.3
0.4
0.5

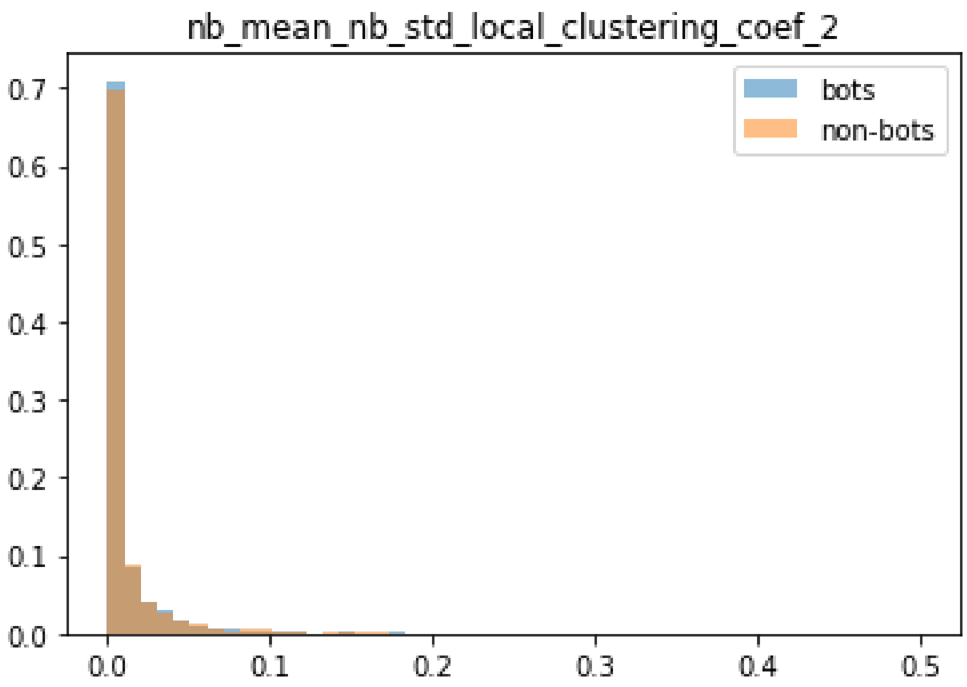
nb_mean_nb_std_local_clustering_coef_2
bots
non-bots
0.7
0.6
0.5
0.4
0.3
0.2
0.1
0.0
0.0
0.1
0.2
0.3
0.4
0.5

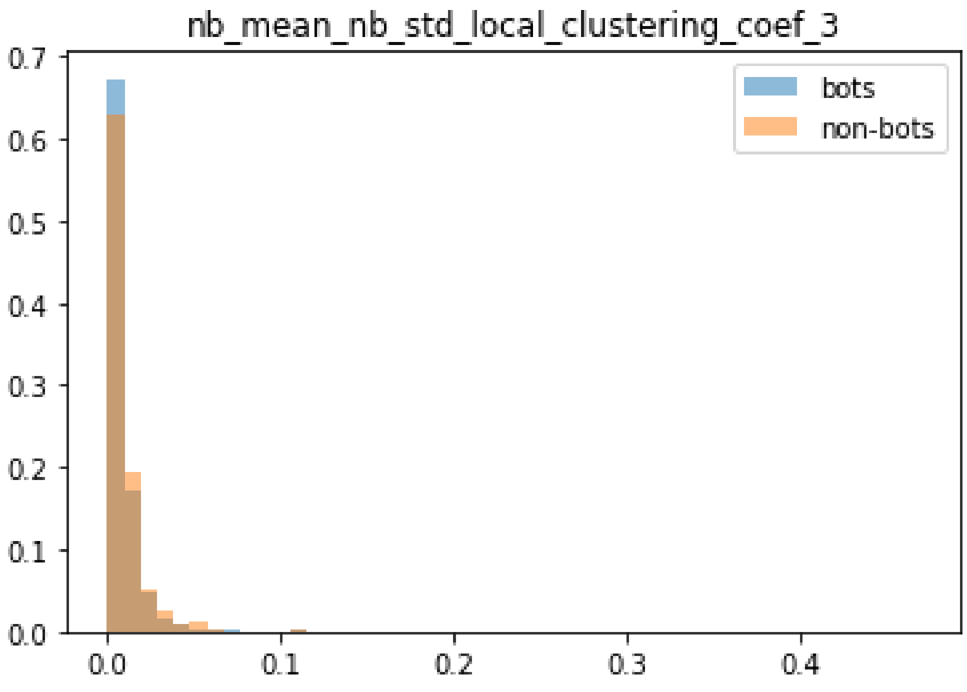
nb_mean_nb_std_local_clustering_coef_3
bots
non-bots
0.7
0.6
0.5
0.4
0.3
0.2
0.1
0.0
0.0
0.1
0.2
0.3
0.4

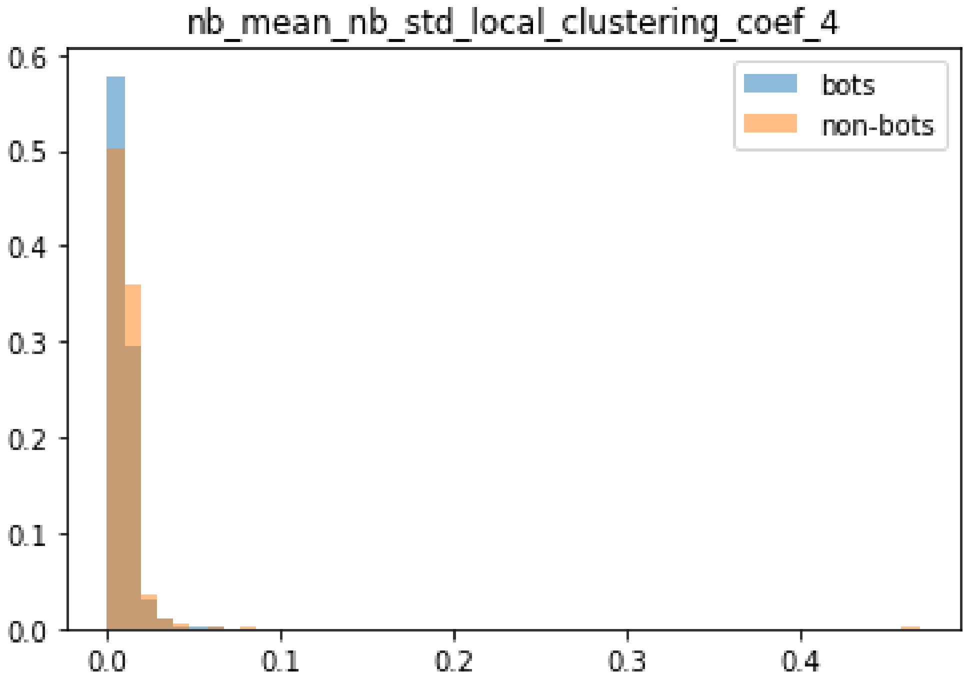

nb_mean_nb_std_local_clustering_coef_4
bots
non-bots
0.6
0.5
0.4
0.3
0.2
0.1
0.0
0.0
0.1
0.2
0.3
0.4
