# Supplementary material for: Detecting anomalies in graph networks on digital markets
Source: PLoS One. 2024 Dec 23;19(12):e0315849. doi: 10.1371/journal.pone.0315849 (PMC11666048; doi:10.1371/journal.pone.0315849)
Supplement: S1 Appendix — The Technical appendix can be found under: https://www.kaggle.com/datasets/agatasko/tech-appendix. List of supplements: plots:a. 01_TwiBot_20_histograms.htmlb. 02_Bitcoin_OTC_histograms.htmlc. 03_Bitcoin_Alpha_histograms.htmld. 04_TwiBot_20_dimensionality.htmle. 05_Bitcoin_OTC_dimensionality.htmlf. 06_Bitcoin_Alpha_dimensionality.htmltables:a. 01_TwiBot_20_statistics.csvb. 02_Bitcoin_OTC_statistics.csvc. 03_Bitcoin_Alpha_statistics.csvd. 04_TwiBot_20_results.csve. 05_Bitcoin_OTC_results.csvf. 06_Bitcoin_Alpha_results.csvg. 07_TwiBot_20_compression_results.csvh. 08_Bitcoin_OTC_compression_results.csvi. 09_Bitcoin_Alpha_compression_results.csv (ZIP) [file pone.0315849.s001.zip › tech_appendix/plots/pdf/02_Bitcoin_OTC_histograms.pdf]

02_Bitcoin_OTC_histograms

August 19, 2023

[1]:
```
import numpy as np
import pandas as pd
import matplotlib.pyplot as plt
```

0.1 Bitcoin OTC

[2]:
```
bitcoin_otc_gf = pd.read_csv("bitcoin_otc_gf.csv")
for col in bitcoin_otc_gf.columns[1:-1]:
    x = bitcoin_otc_gf.loc[bitcoin_otc_gf["label"] == 1, col]
    y = bitcoin_otc_gf.loc[bitcoin_otc_gf["label"] == 0, col]

    bins = np.linspace(min(bitcoin_otc_gf[col]), max(bitcoin_otc_gf[col]), 50)

    plt.hist(round(x, 5), bins, alpha=0.5, label='benign', weights=np.
 ↪ones(len(x))/len(x))
    plt.hist(y, bins, alpha=0.5, label='fraudulent', weights=np.ones(len(y))/
 ↪len(y))
    plt.legend(loc='upper right')
    plt.title(col)
    #plt.savefig(f'./charts/{col}.pdf')
    plt.show()
```

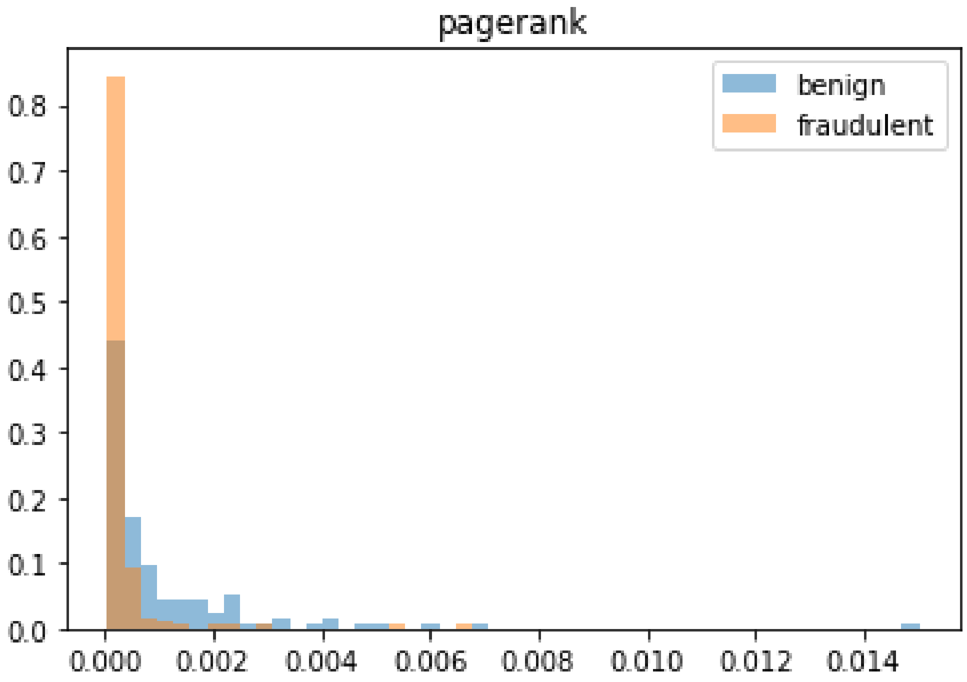
pagerank
benign
fraudulent
0.0
0.1
0.2
0.3
0.4
0.5
0.6
0.7
0.8
0.000
0.002
0.004
0.006
0.008
0.010
0.012
0.014

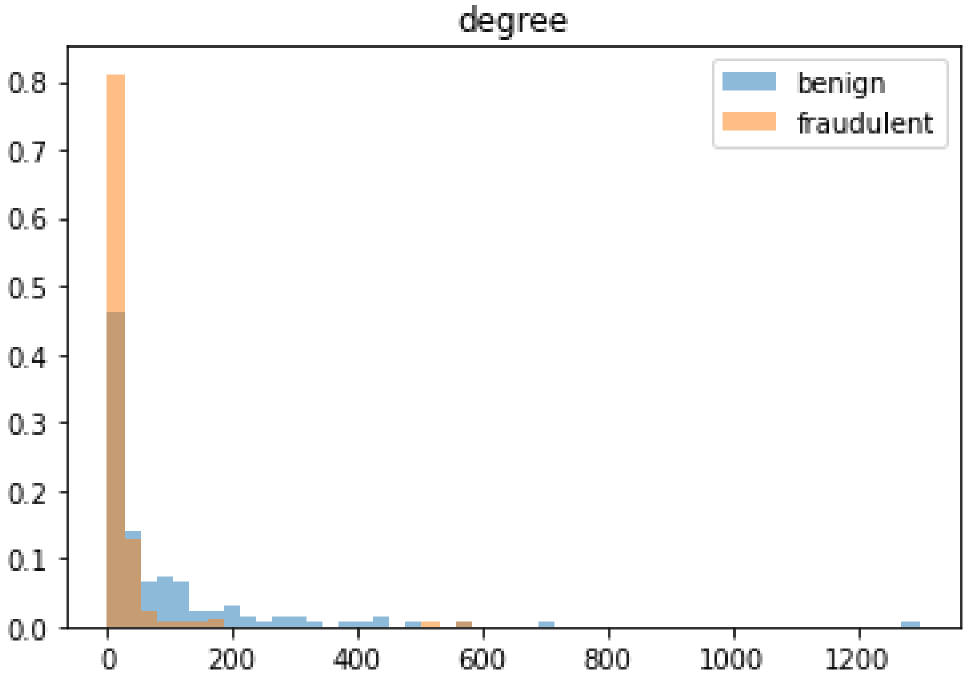
degree
benign
fraudulent
0.0
0.1
0.2
0.3
0.4
0.5
0.6
0.7
0.8
0
200
400
600
800
1000
1200

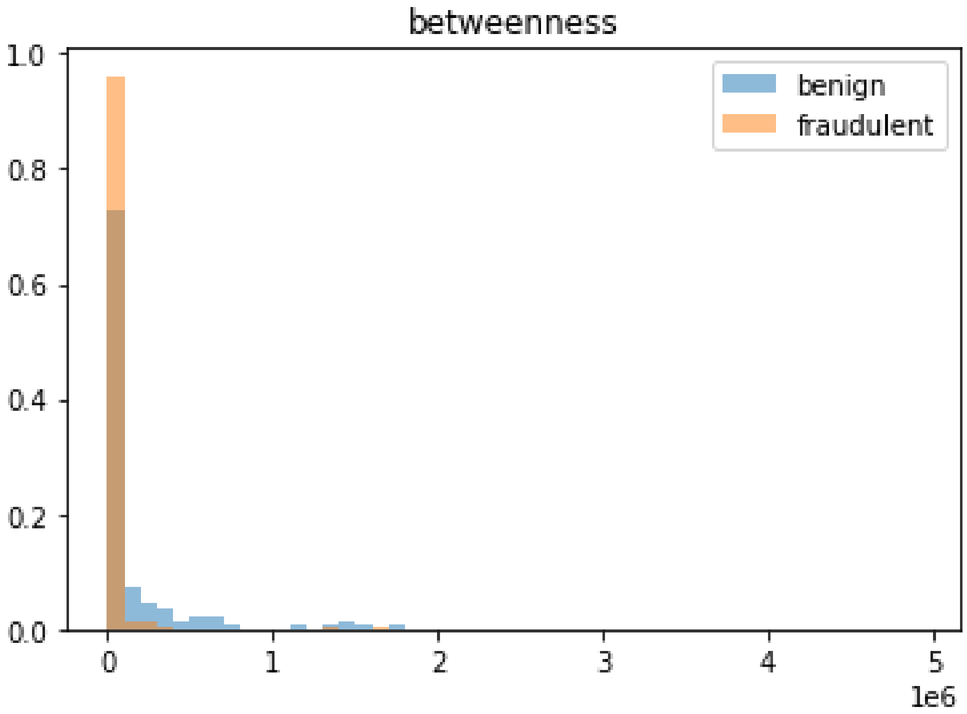
betweenness
benign
fraudulent
1.0
0.8
0.6
0.4
0.2
0.0
0
1
2
3
4
5
1e6

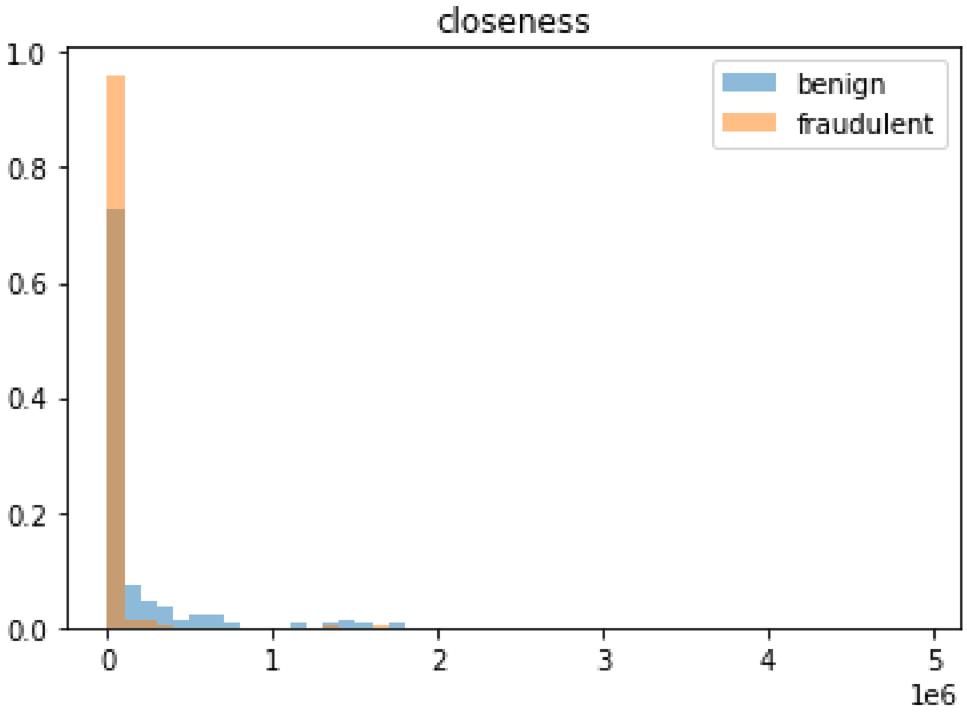
closeness
benign
fraudulent
1.0
0.8
0.6
0.4
0.2
0.0
0
1
2
3
4
5
1e6

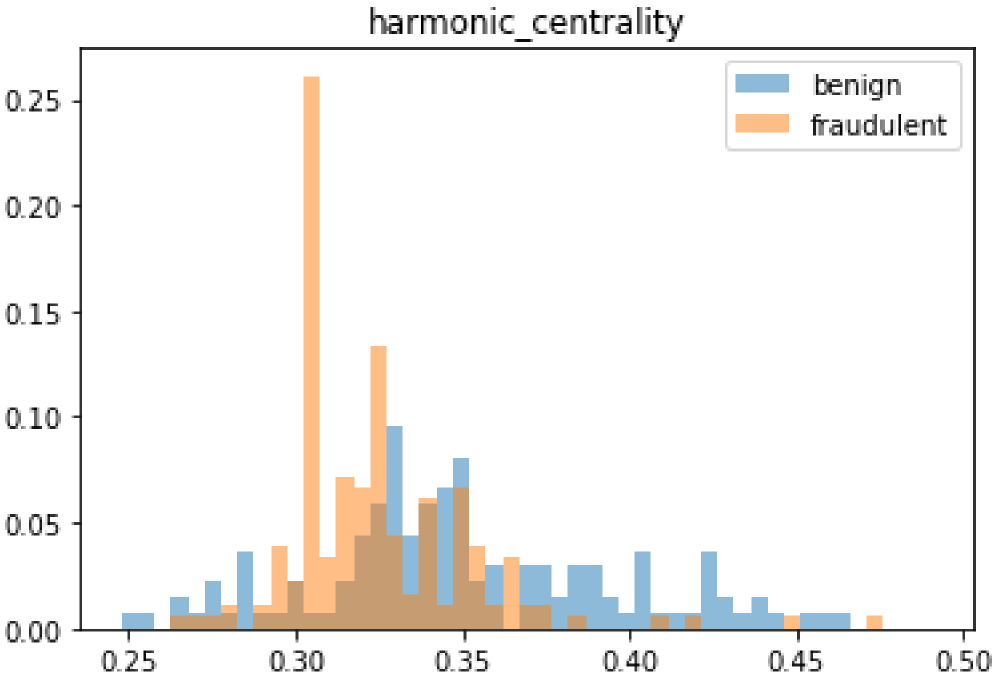
harmonic_centrality
benign
fraudulent
0.00
0.05
0.10
0.15
0.20
0.25
0.25
0.30
0.35
0.40
0.45
0.50

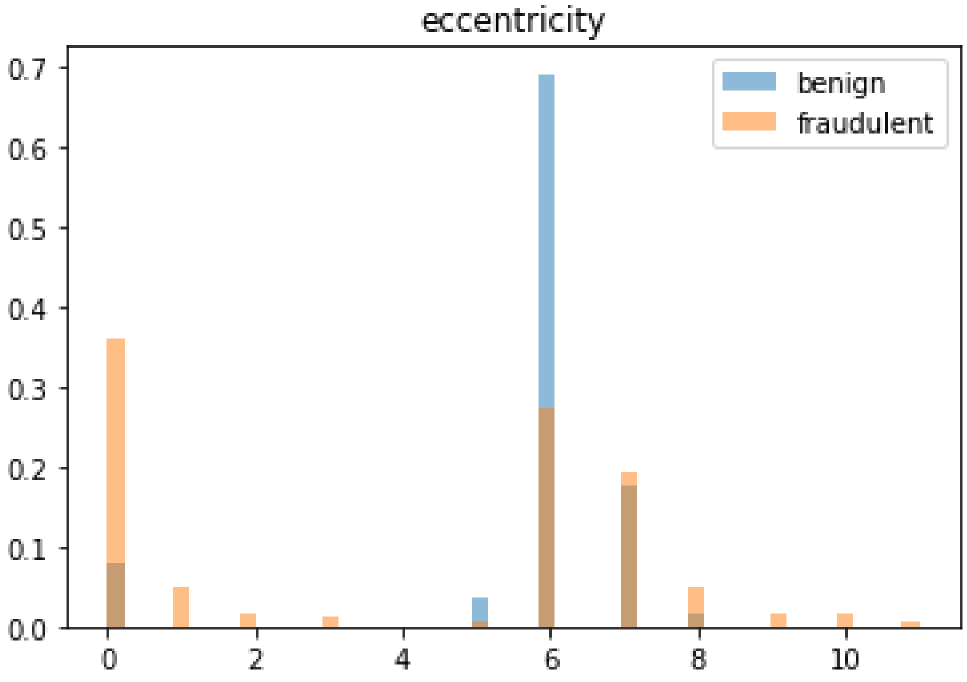
eccentricity
benign
fraudulent
0.0
0.1
0.2
0.3
0.4
0.5
0.6
0.7
0
2
4
6
8
10

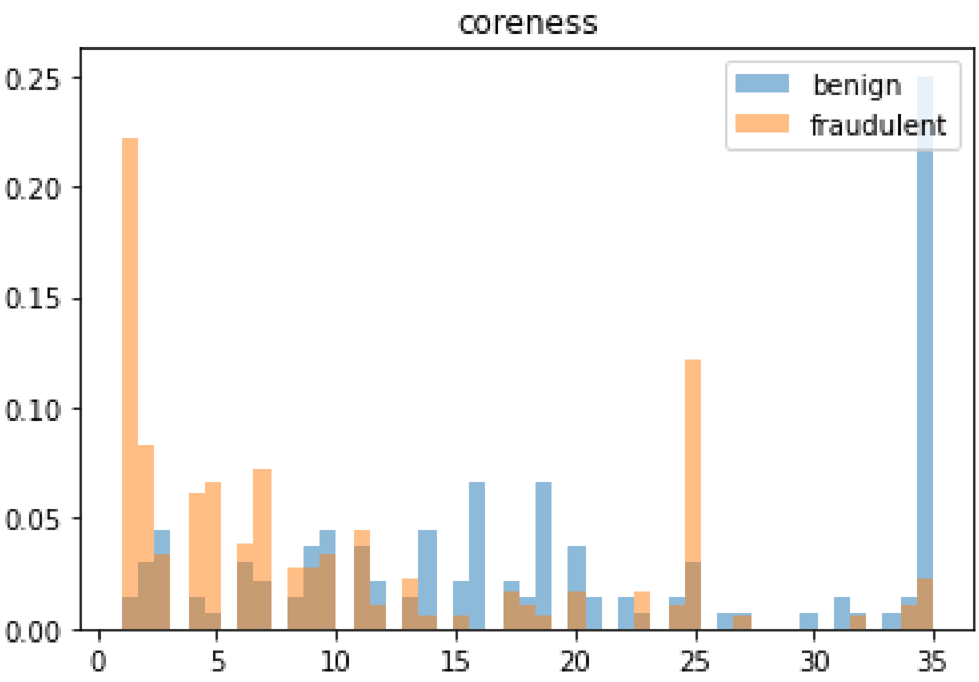
coreness
benign
fraudulent
0.25
0.20
0.15
0.10
0.05
0.00
0
5
10
15
20
25
30
35

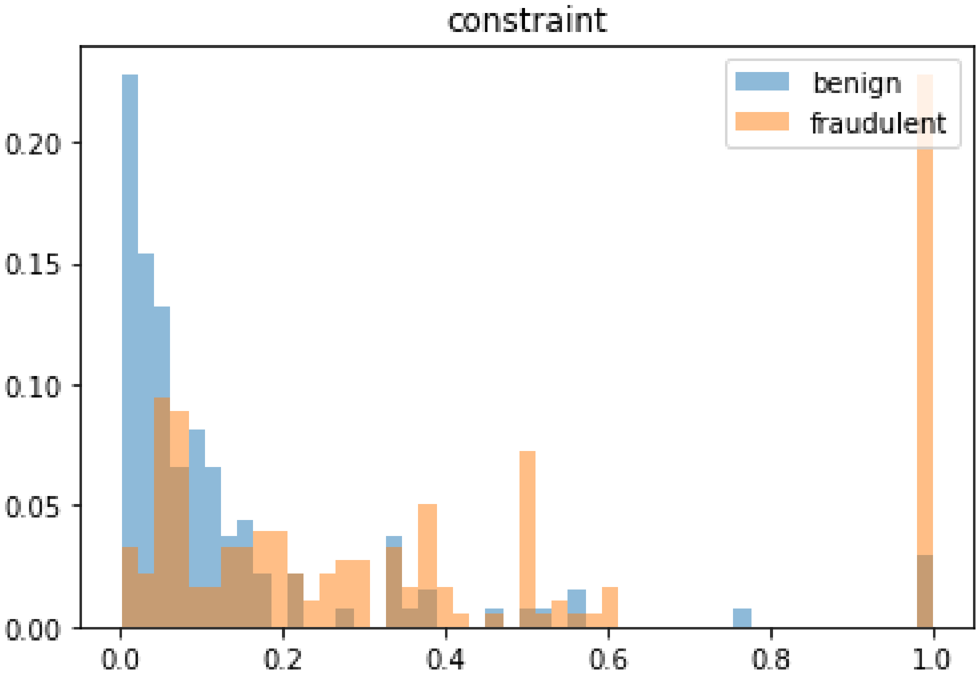
constraint
benign
fraudulent
0.20
0.15
0.10
0.05
0.00
0.0
0.2
0.4
0.6
0.8
1.0

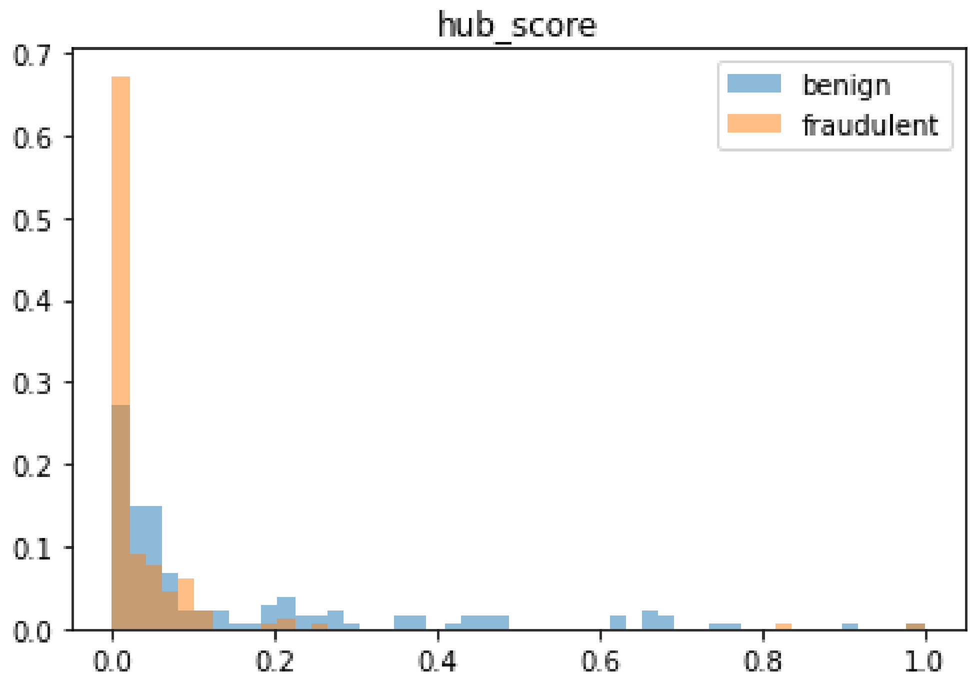
hub_score
benign
fraudulent
0.7
0.6
0.5
0.4
0.3
0.2
0.1
0.0
0.0
0.2
0.4
0.6
0.8
1.0

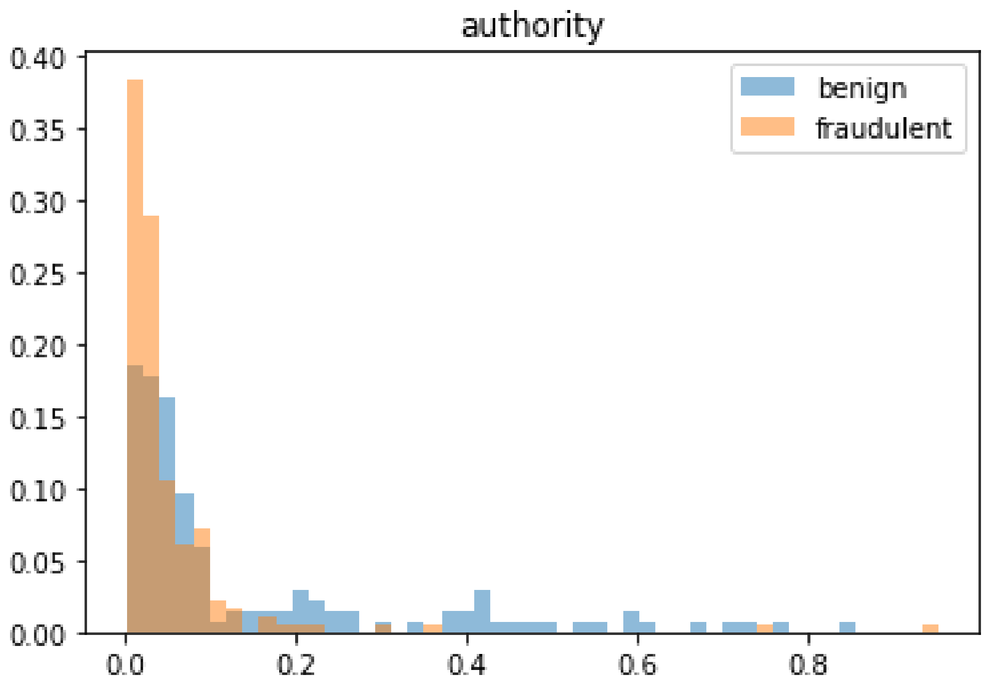
authority
benign
fraudulent
0.40
0.35
0.30
0.25
0.20
0.15
0.10
0.05
0.00
0.0
0.2
0.4
0.6
0.8

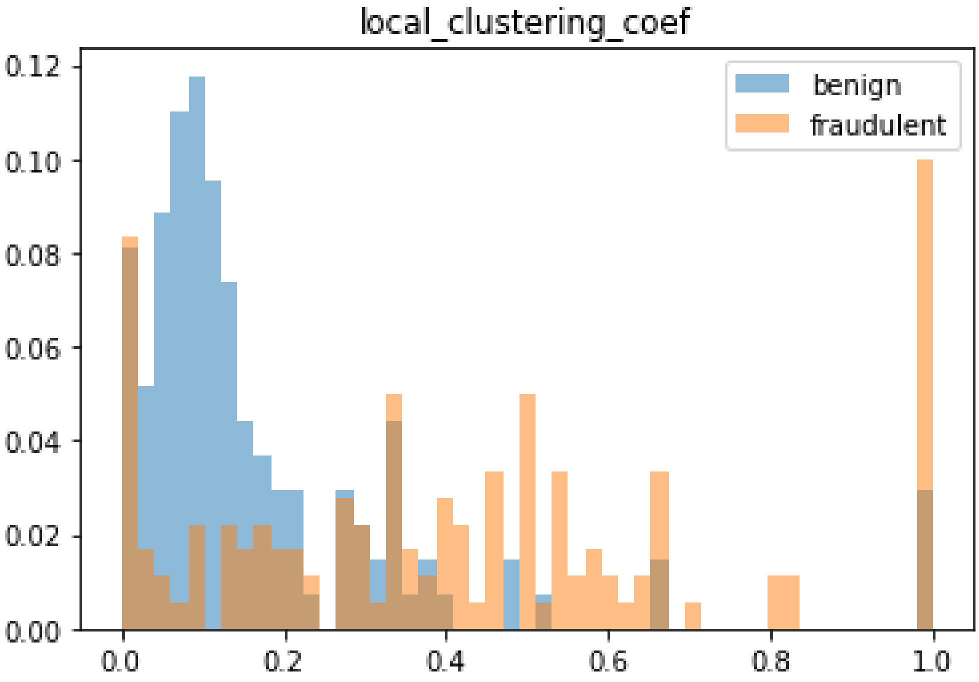
local_clustering_coef
benign
fraudulent
0.00
0.02
0.04
0.06
0.08
0.10
0.12
0.0
0.2
0.4
0.6
0.8
1.0

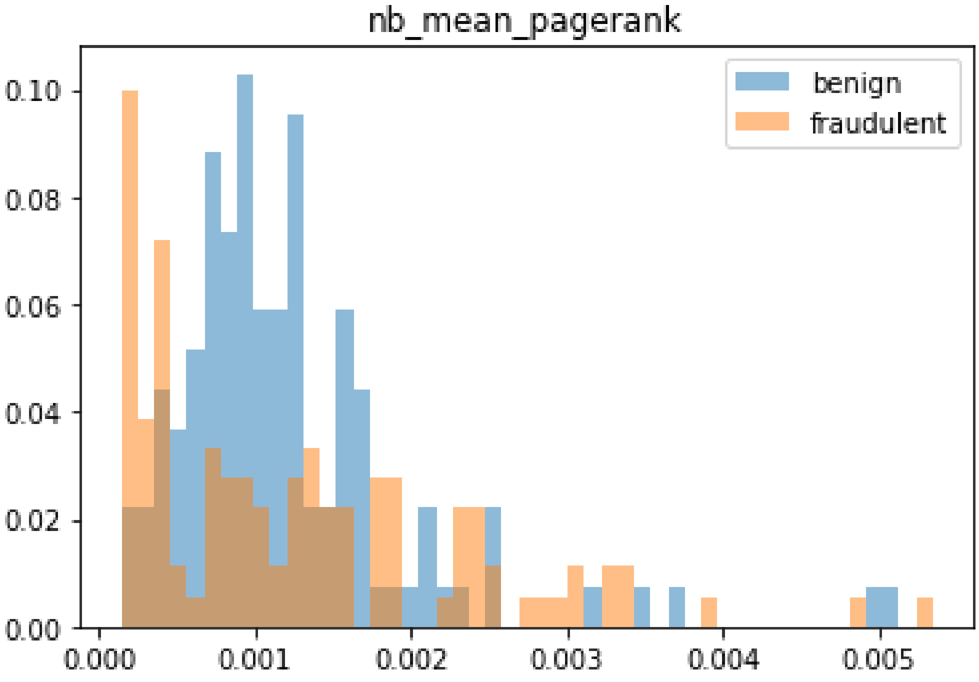
nb_mean_pagerank
benign
fraudulent
0.00
0.02
0.04
0.06
0.08
0.10
0.000
0.001
0.002
0.003
0.004
0.005

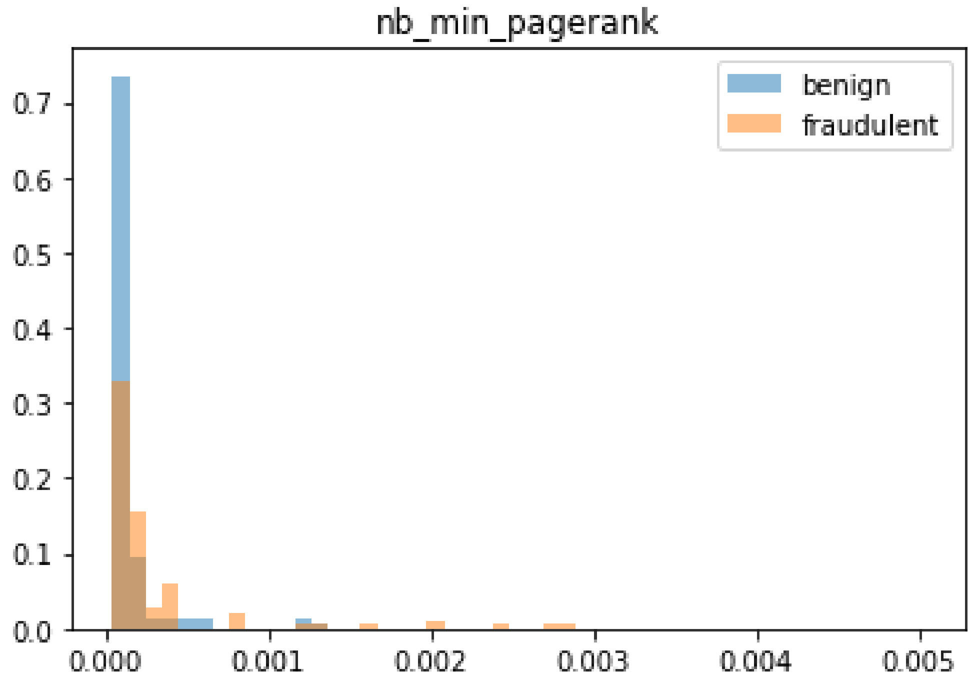
nb_min_pagerank
benign
fraudulent
0.7
0.6
0.5
0.4
0.3
0.2
0.1
0.0
0.000
0.001
0.002
0.003
0.004
0.005

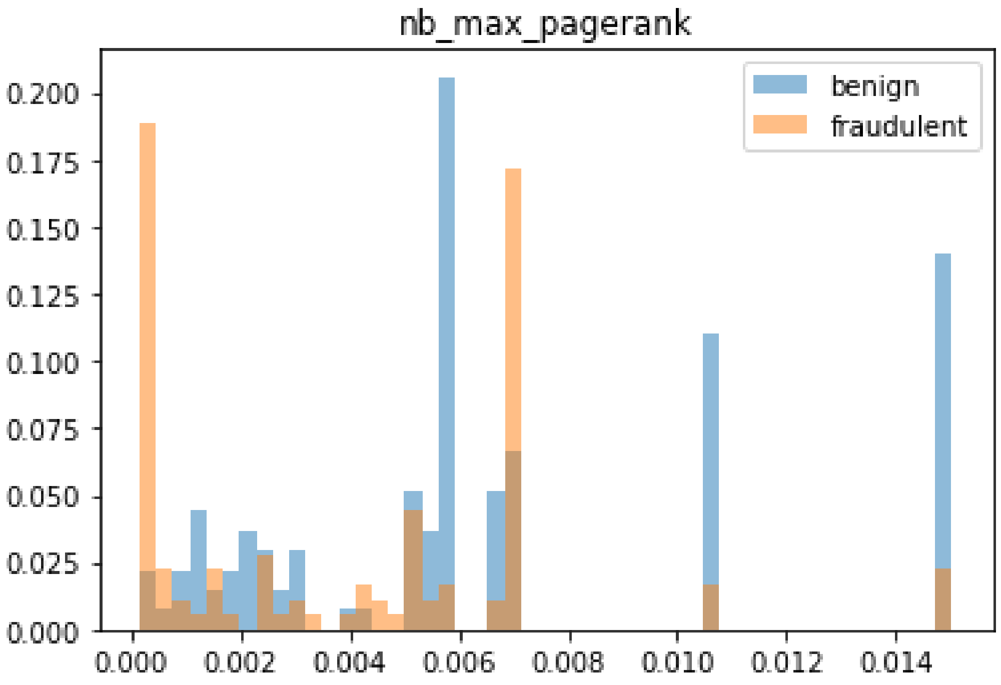
nb_max_pagerank
benign
fraudulent
0.200
0.175
0.150
0.125
0.100
0.075
0.050
0.025
0.000
0.000
0.002
0.004
0.006
0.008
0.010
0.012
0.014

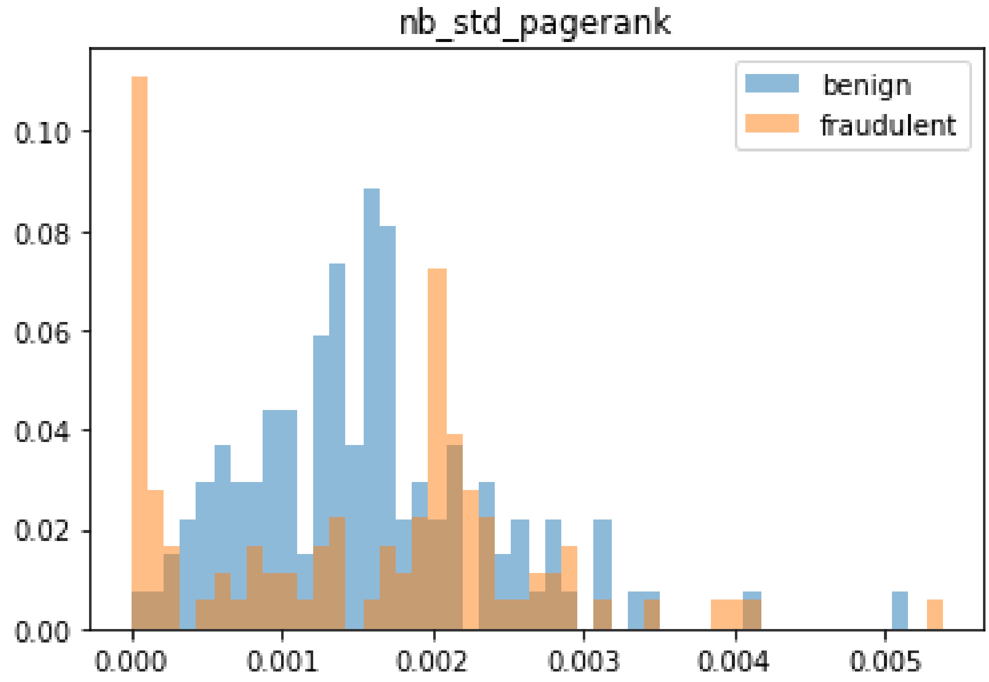
nb_std_pagerank
benign
fraudulent
0.10
0.08
0.06
0.04
0.02
0.00
0.000
0.001
0.002
0.003
0.004
0.005

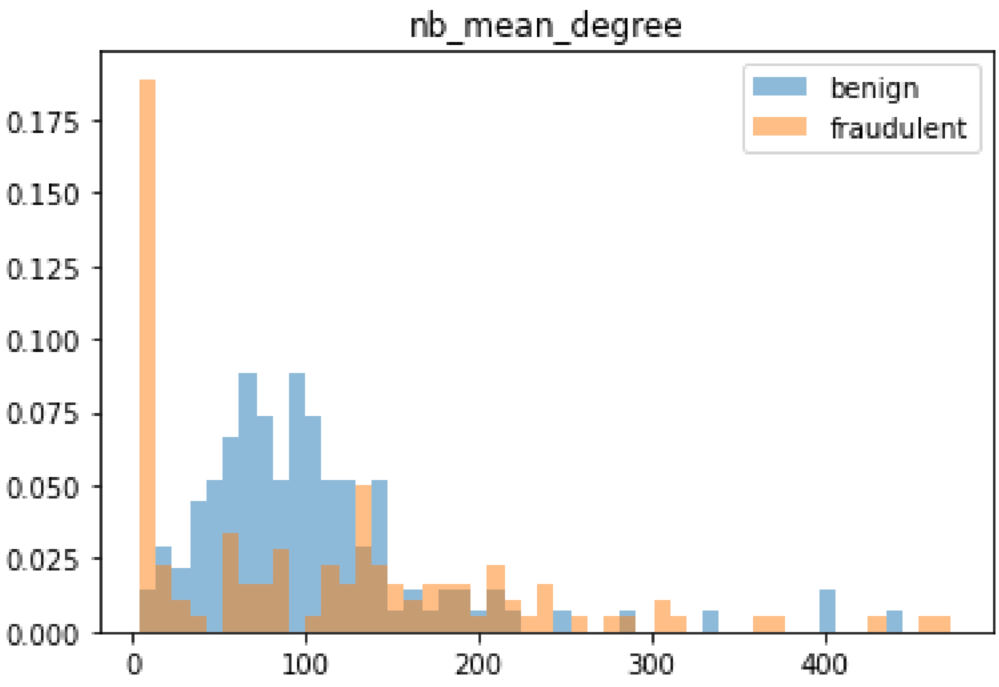
nb_mean_degree
benign
fraudulent
0.175
0.150
0.125
0.100
0.075
0.050
0.025
0.000
0
100
200
300
400

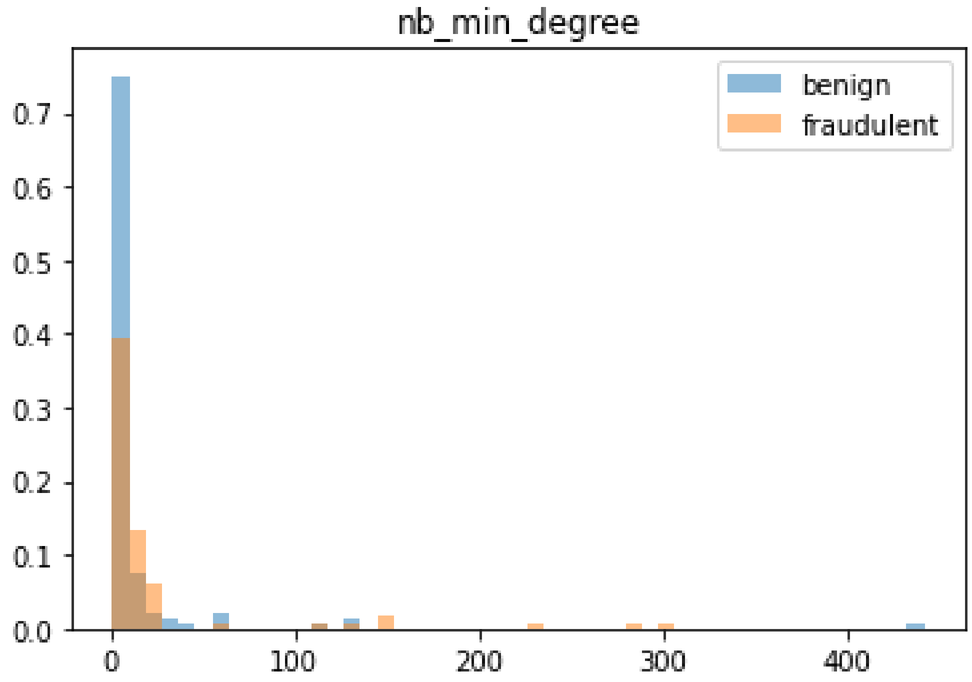
nb_min_degree
benign
fraudulent
0.7
0.6
0.5
0.4
0.3
0.2
0.1
0.0
0
100
200
300
400

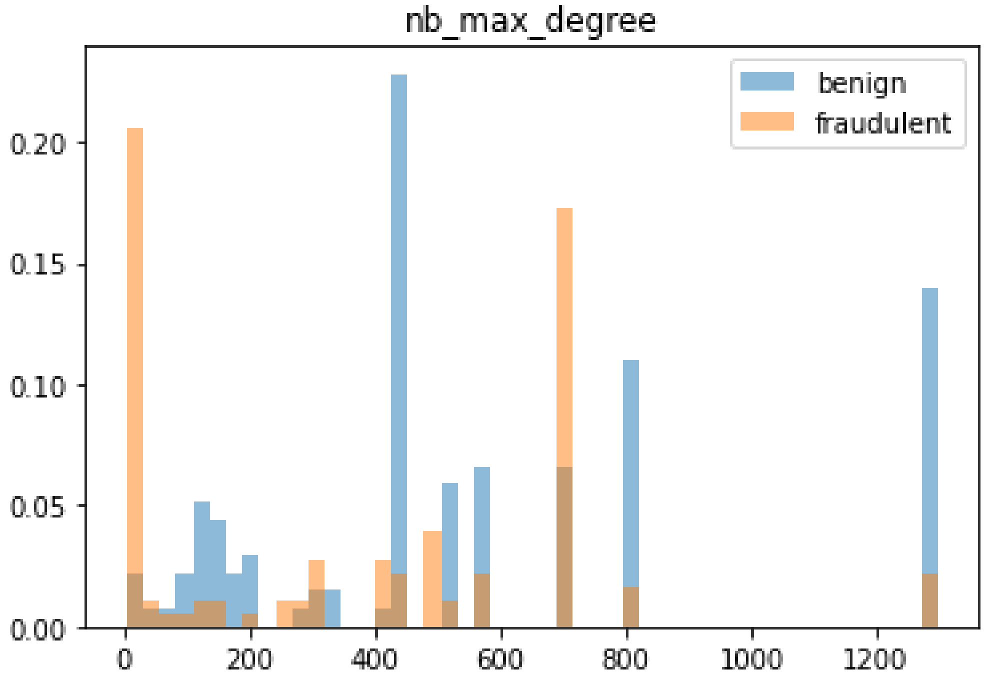
nb_max_degree
benign
fraudulent
0.20
0.15
0.10
0.05
0.00
0
200
400
600
800
1000
1200

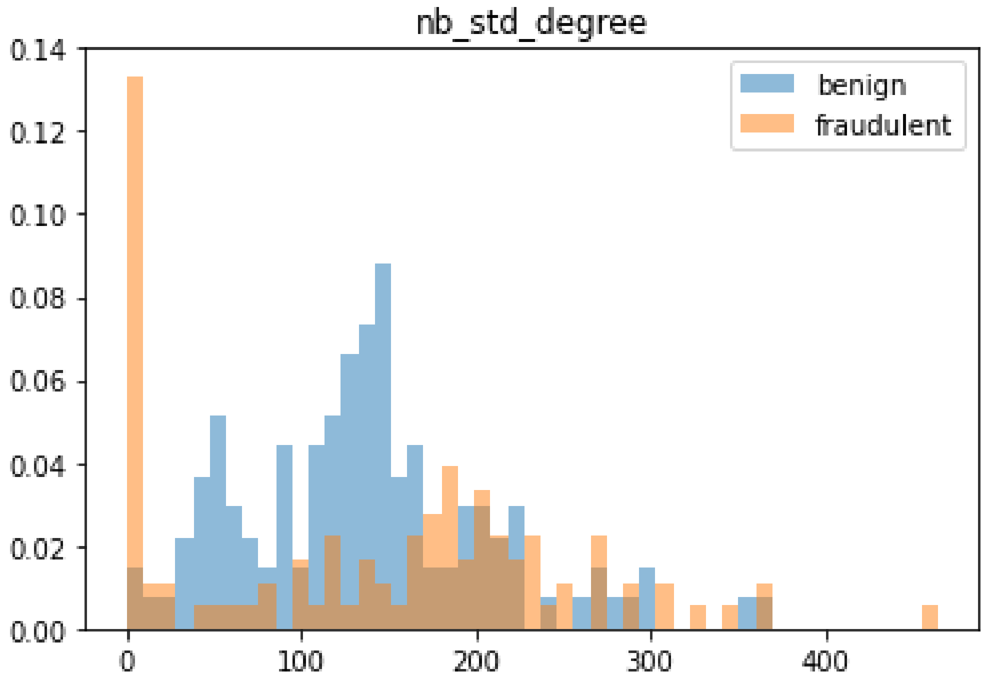
nb_std_degree
benign
fraudulent
0.14
0.12
0.10
0.08
0.06
0.04
0.02
0.00
0
100
200
300
400

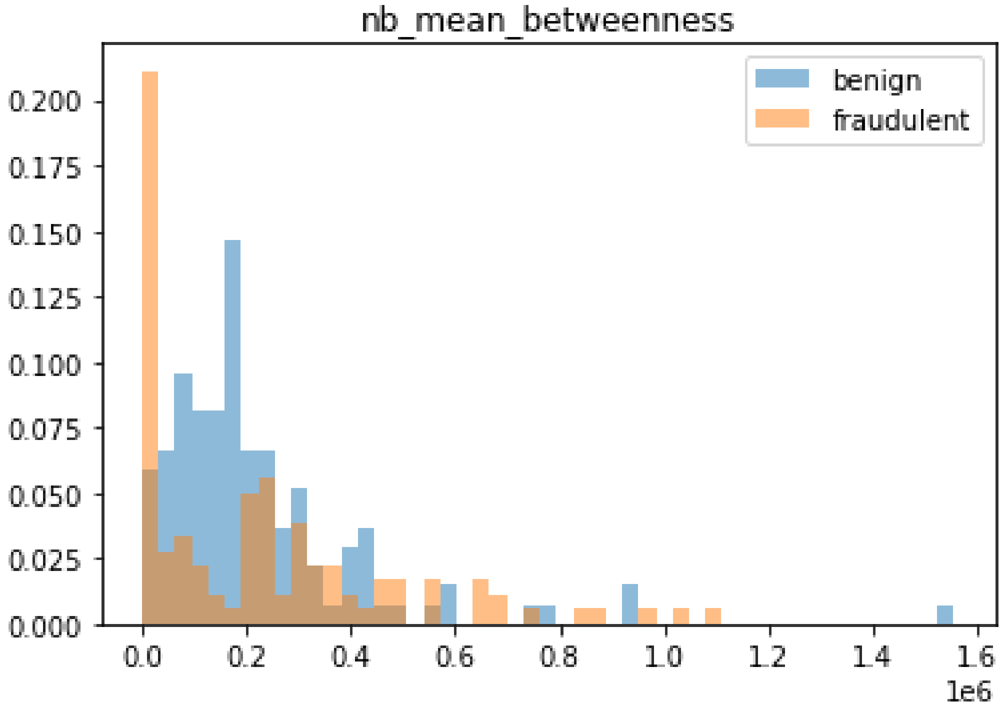
nb_mean_betweenness
benign
fraudulent
0.200
0.175
0.150
0.125
0.100
0.075
0.050
0.025
0.000
0.0
0.2
0.4
0.6
0.8
1.0
1.2
1.4
1.6
1e6

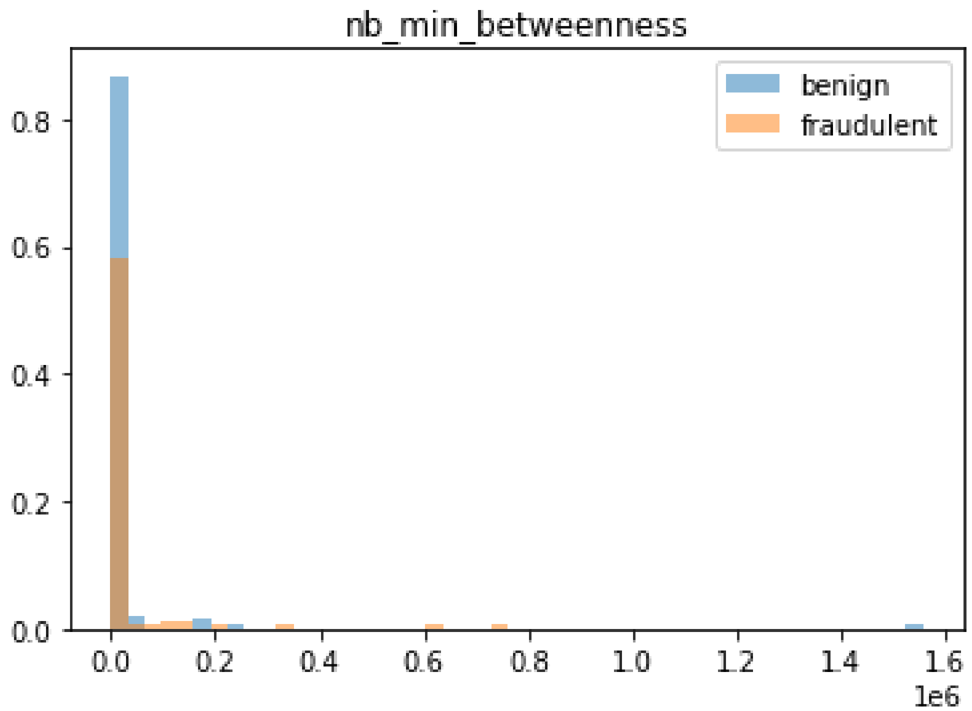
nb_min_betweenness
benign
fraudulent
0.0
0.2
0.4
0.6
0.8
0.0
0.2
0.4
0.6
0.8
1.0
1.2
1.4
1.6
1e6

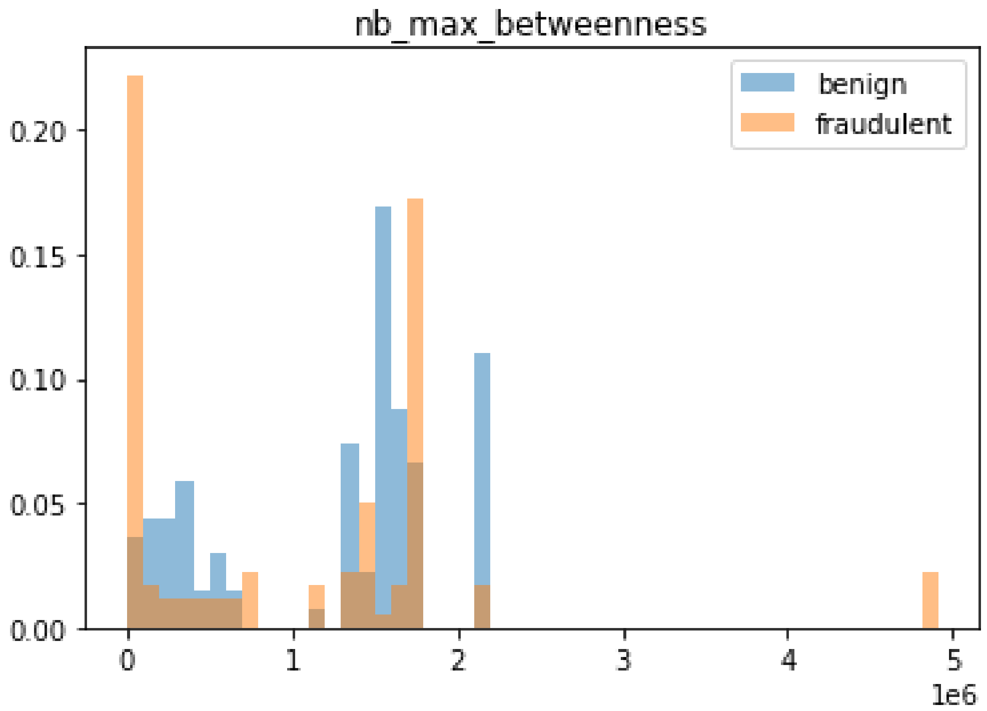
nb_max_betweenness
benign
fraudulent
0.00
0.05
0.10
0.15
0.20
0
1
2
3
4
5
1e6

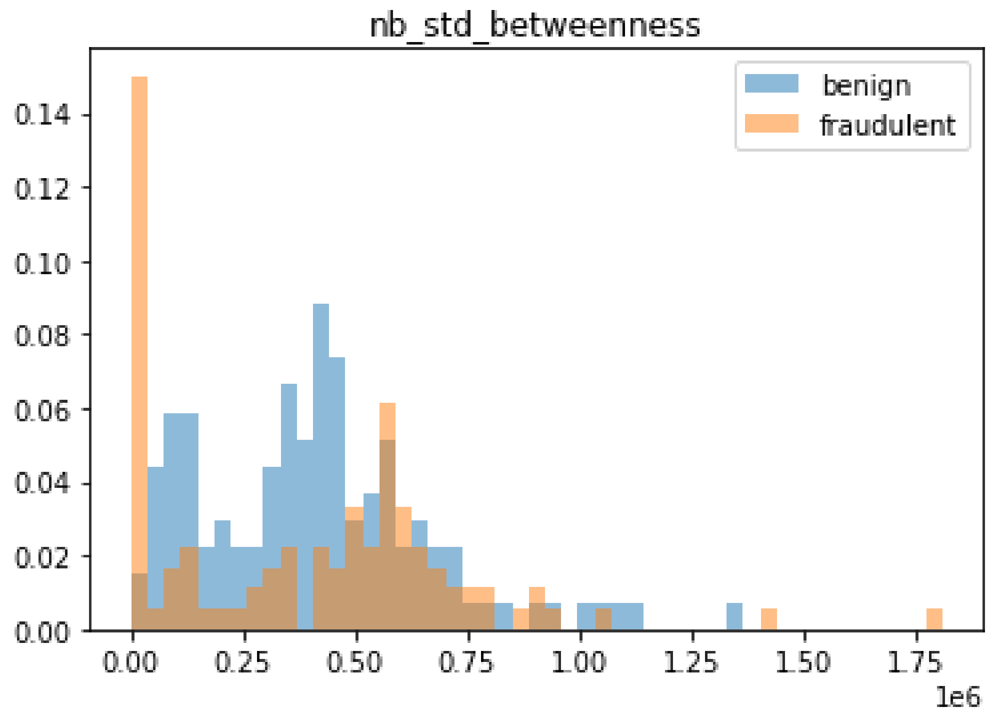
nb_std_betweenness
benign
fraudulent
0.14
0.12
0.10
0.08
0.06
0.04
0.02
0.00
0.00
0.25
0.50
0.75
1.00
1.25
1.50
1.75
1e6

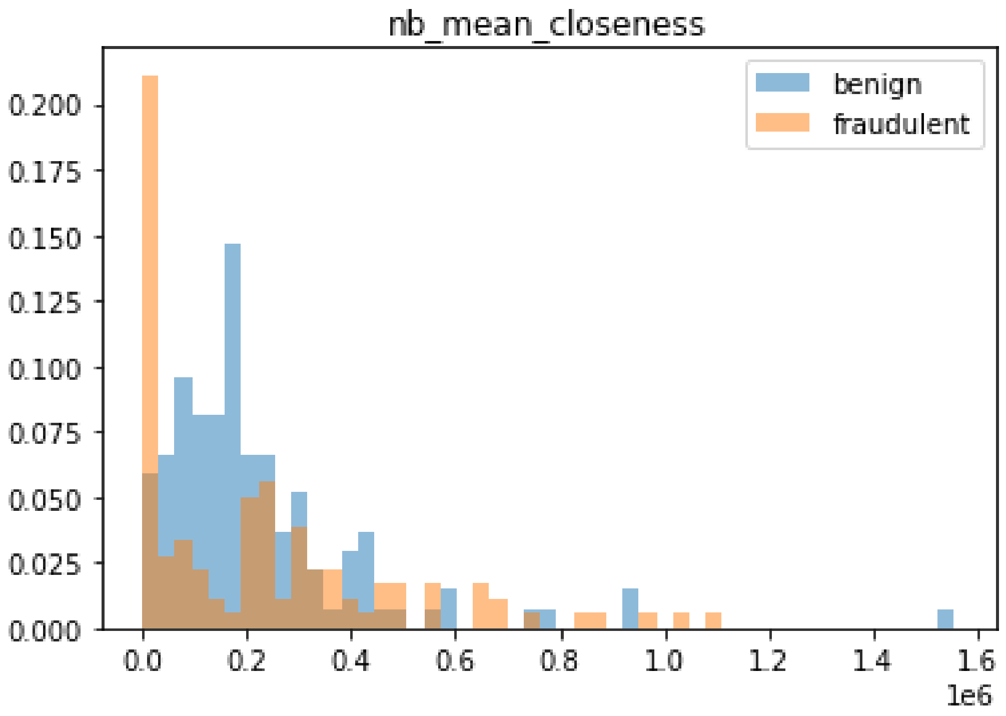
nb_mean_closeness
benign
fraudulent
0.200
0.175
0.150
0.125
0.100
0.075
0.050
0.025
0.000
0.0
0.2
0.4
0.6
0.8
1.0
1.2
1.4
1.6
1e6

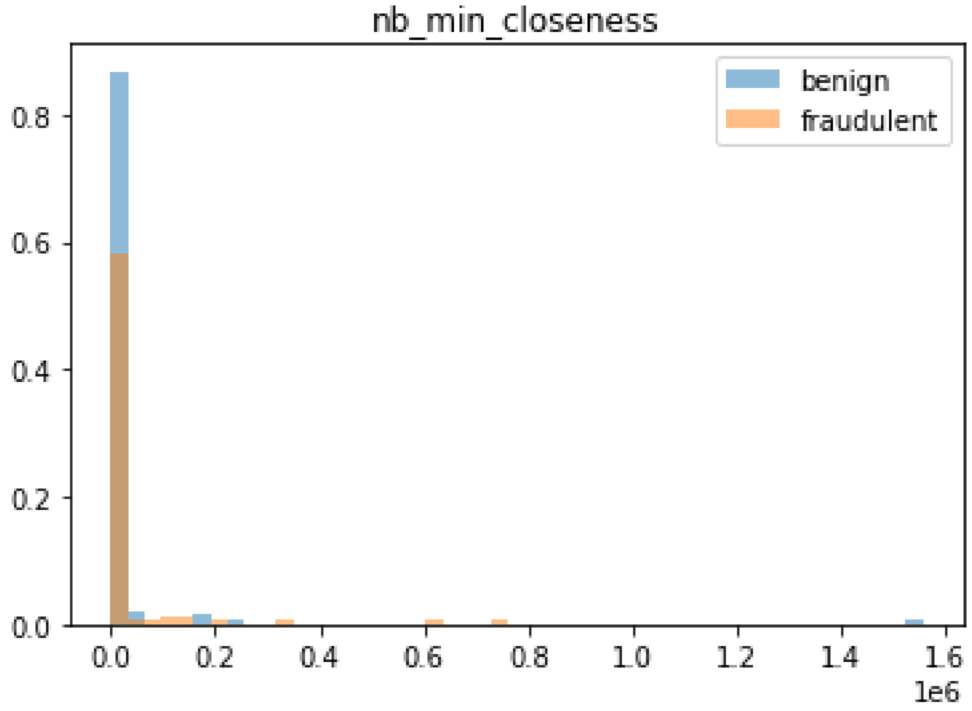
nb_min_closeness
benign
fraudulent
0.0
0.2
0.4
0.6
0.8
0.0
0.2
0.4
0.6
0.8
1.0
1.2
1.4
1.6
1e6

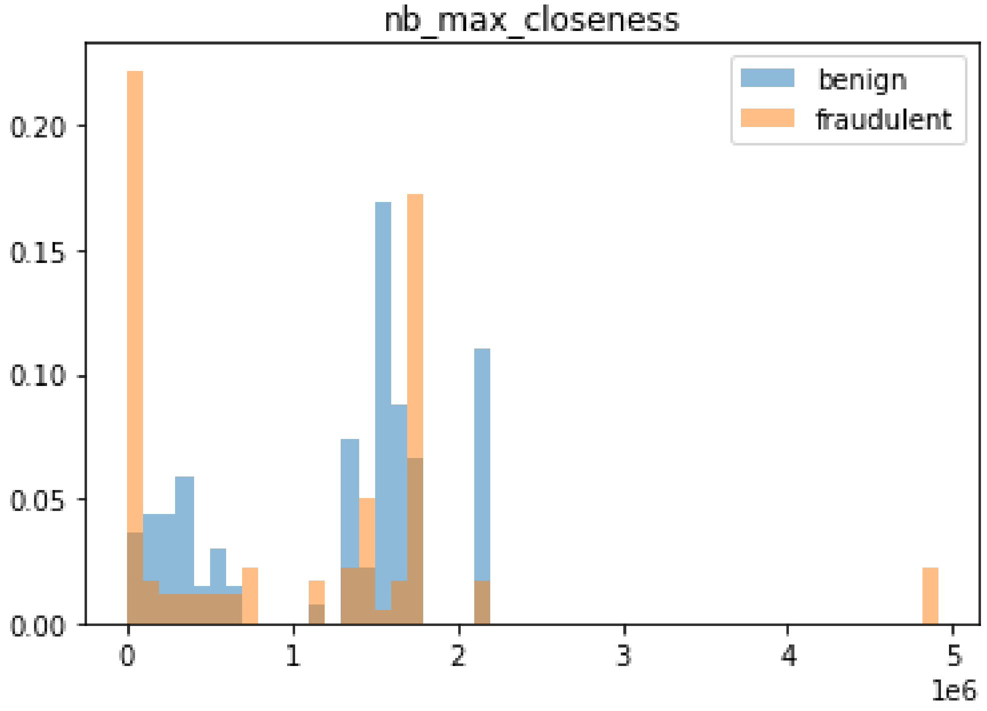
nb_max_closeness
benign
fraudulent
0.00
0.05
0.10
0.15
0.20
0
1
2
3
4
5
1e6

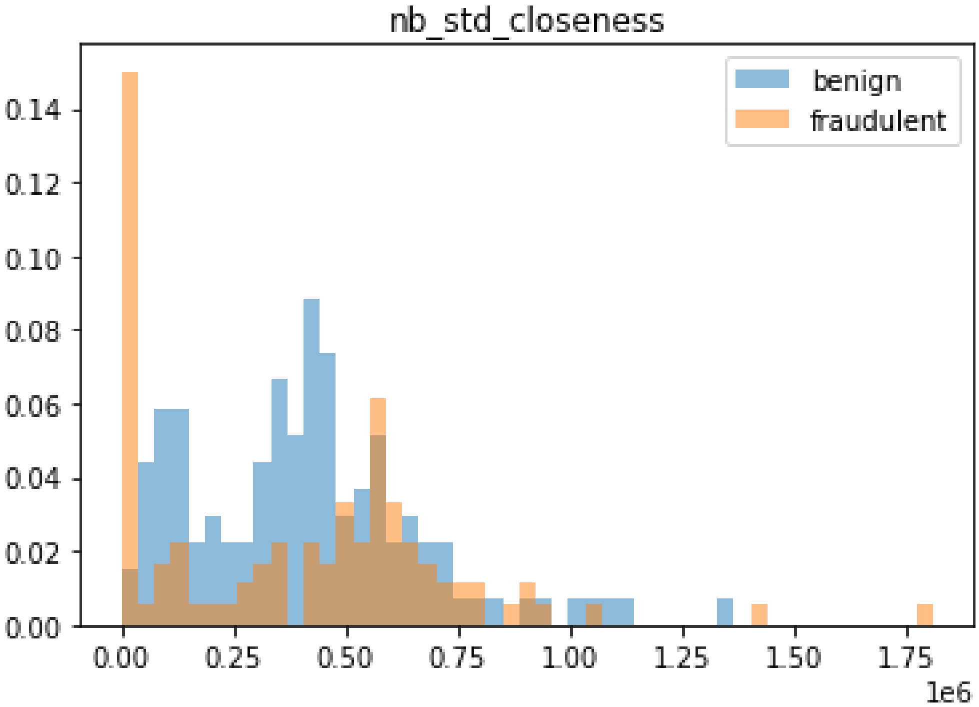
nb_std_closeness
benign
fraudulent
0.14
0.12
0.10
0.08
0.06
0.04
0.02
0.00
0.00
0.25
0.50
0.75
1.00
1.25
1.50
1.75
1e6

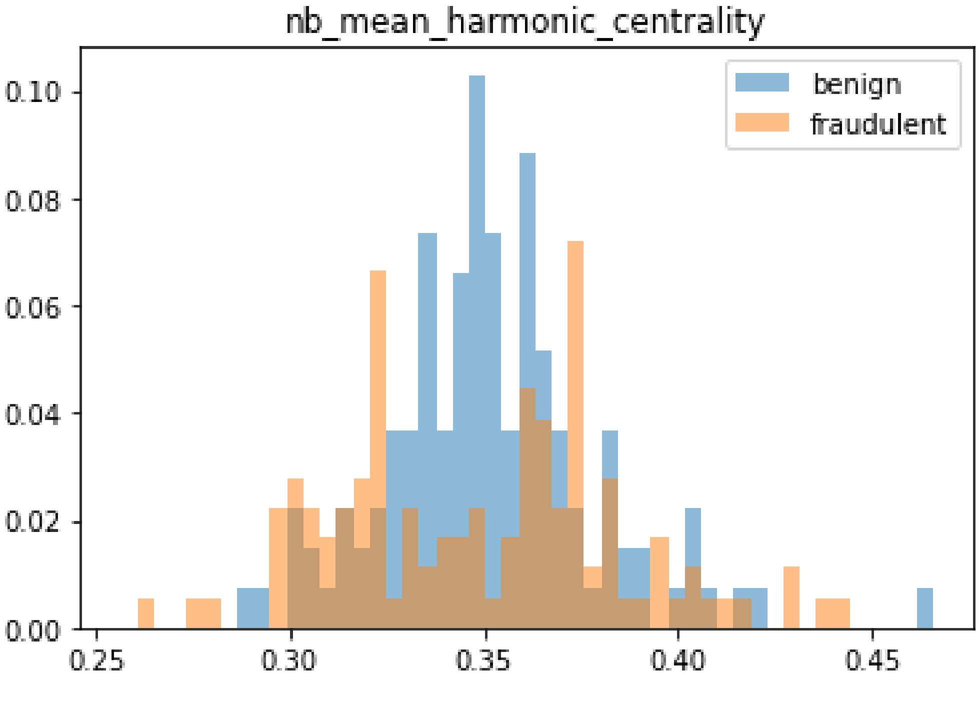
nb_mean_harmonic_centrality
benign
fraudulent
0.10
0.08
0.06
0.04
0.02
0.00
0.25
0.30
0.35
0.40
0.45

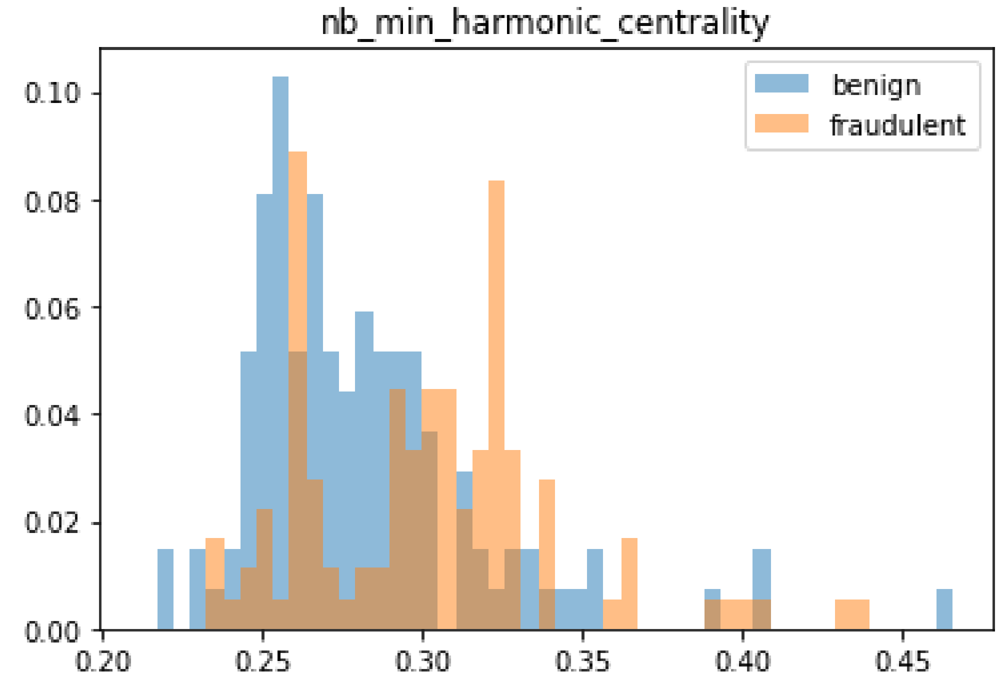
nb_min_harmonic_centrality
benign
fraudulent
0.10
0.08
0.06
0.04
0.02
0.00
0.20
0.25
0.30
0.35
0.40
0.45

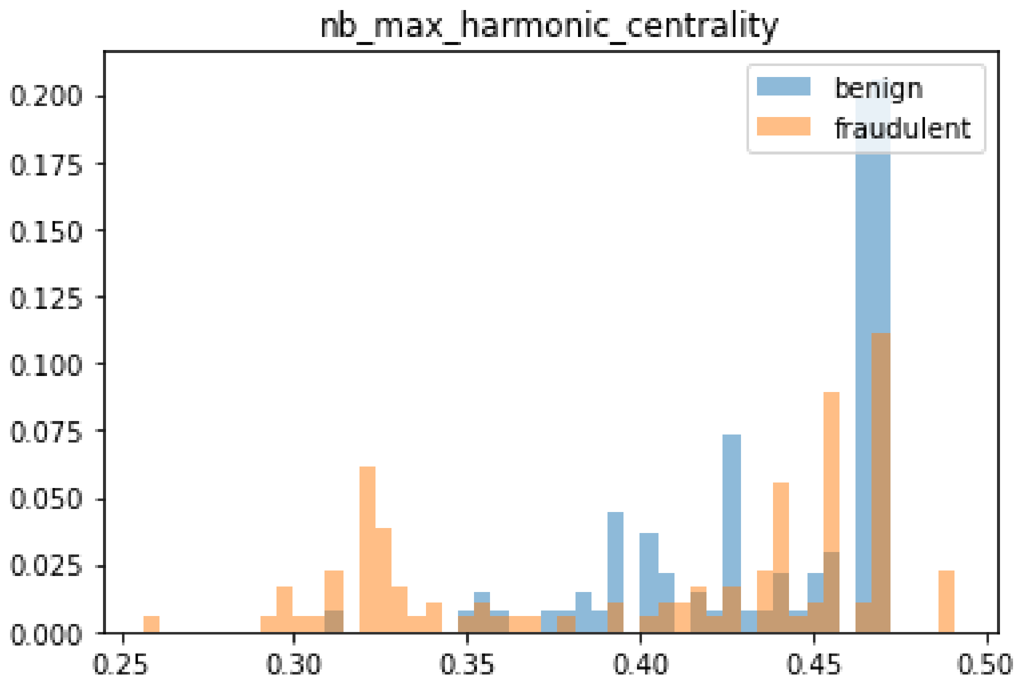
nb_max_harmonic_centrality
benign
fraudulent
0.200
0.175
0.150
0.125
0.100
0.075
0.050
0.025
0.000
0.25
0.30
0.35
0.40
0.45
0.50

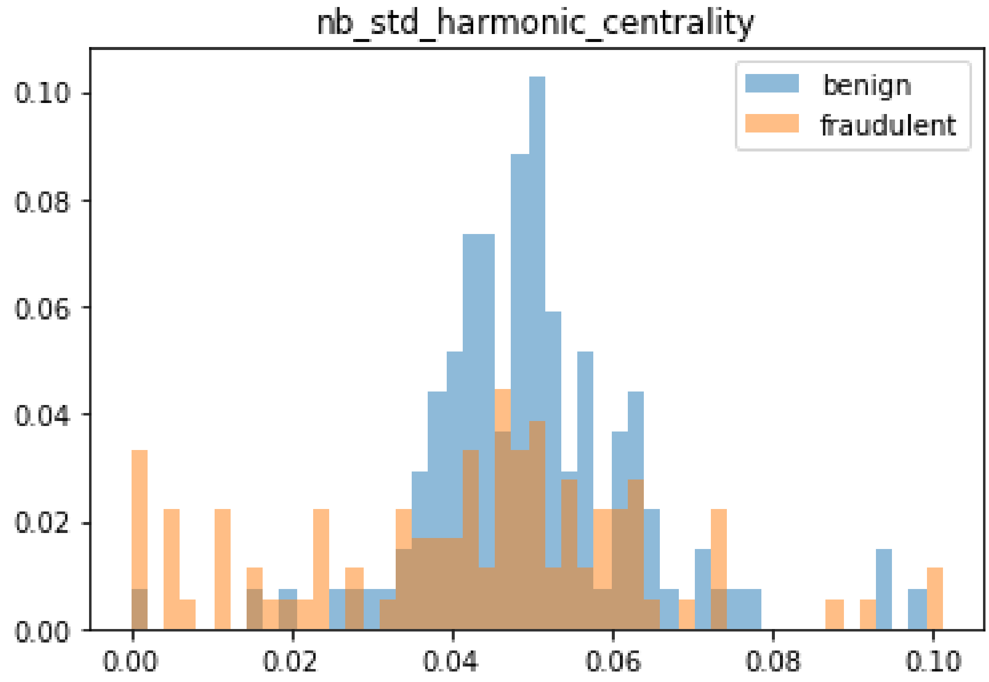
nb_std_harmonic_centrality
benign
fraudulent
0.10
0.08
0.06
0.04
0.02
0.00
0.00
0.02
0.04
0.06
0.08
0.10

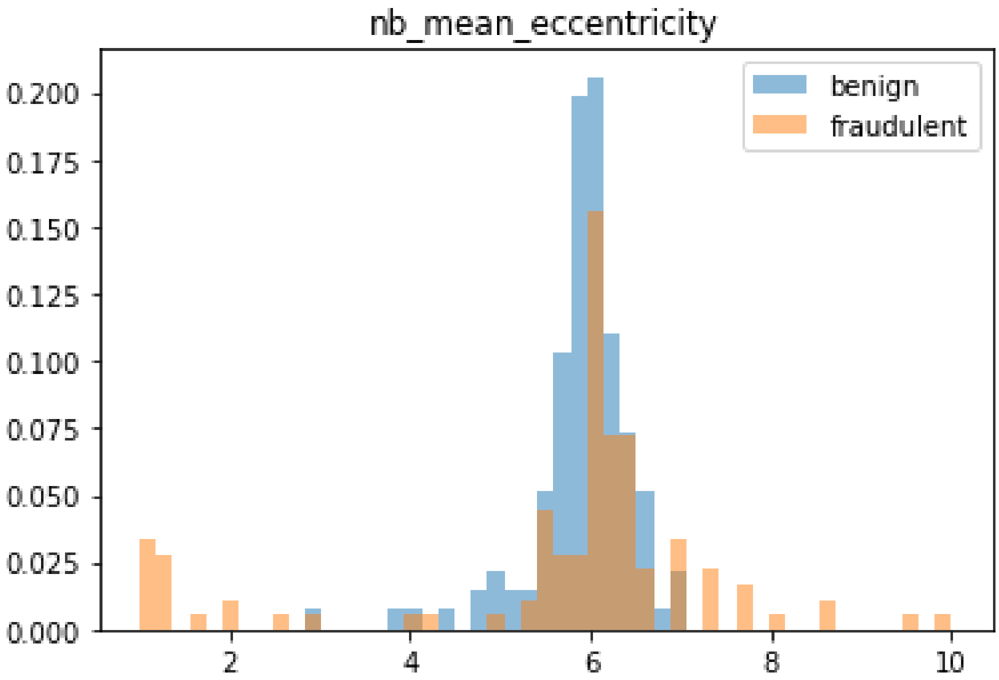
nb_mean_eccentricity
benign
fraudulent
0.200
0.175
0.150
0.125
0.100
0.075
0.050
0.025
0.000
2
4
6
8
10

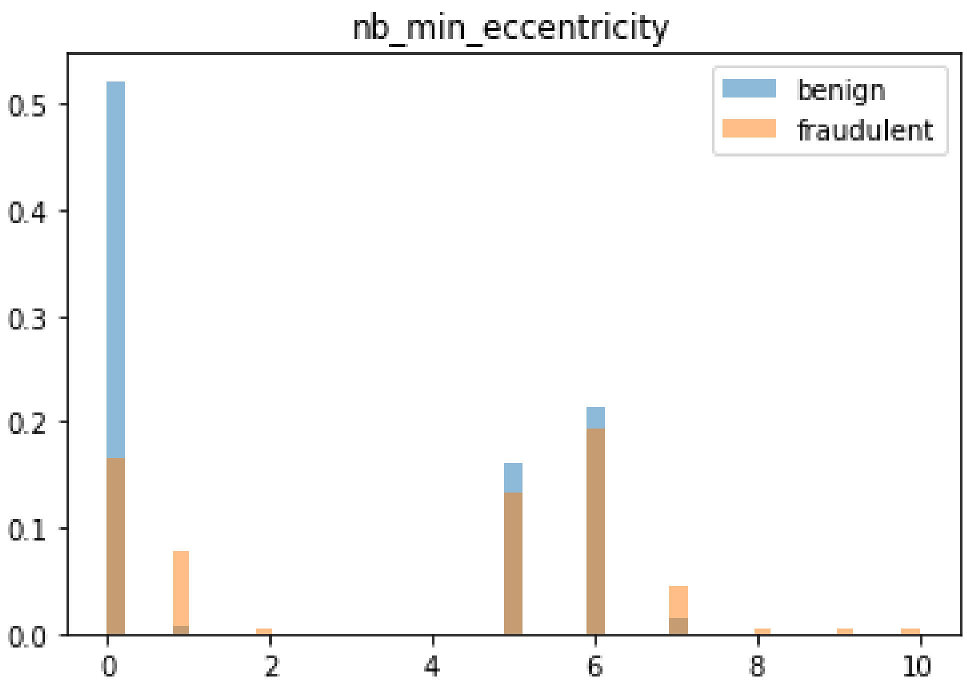
nb_min_eccentricity
benign
fraudulent
0.0
0.1
0.2
0.3
0.4
0.5
0
2
4
6
8
10

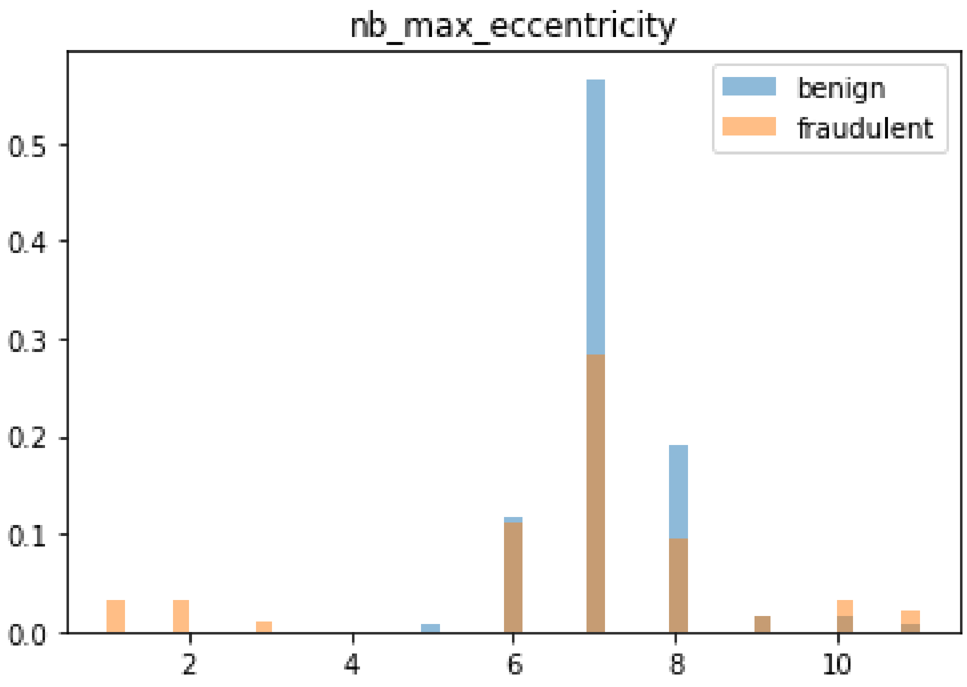
nb_max_eccentricity
benign
fraudulent
0.0
0.1
0.2
0.3
0.4
0.5
2
4
6
8
10

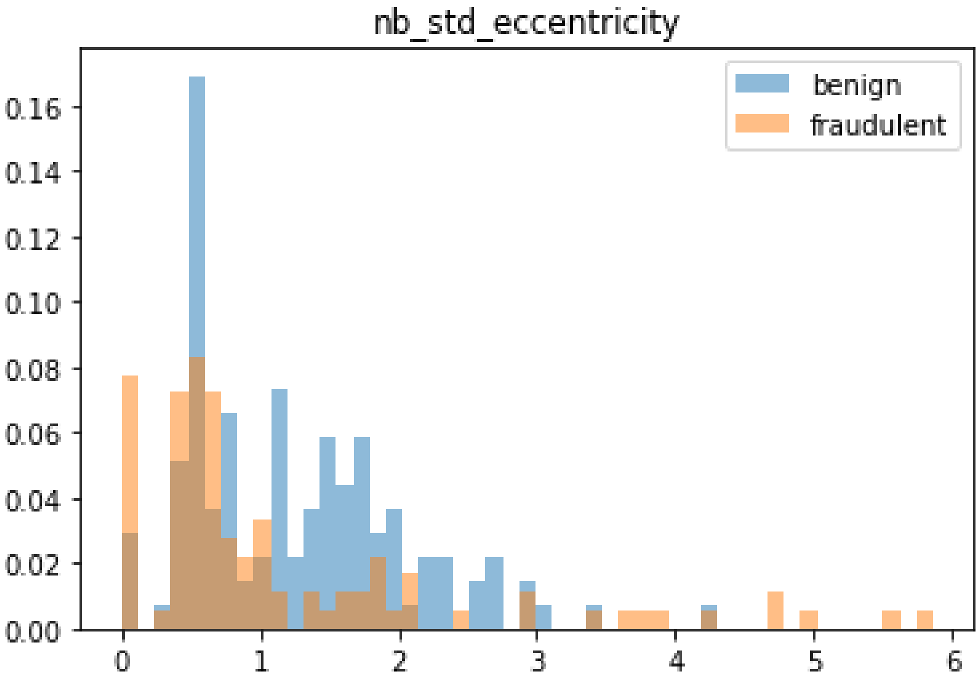
nb_std_eccentricity
benign
fraudulent
0.16
0.14
0.12
0.10
0.08
0.06
0.04
0.02
0.00
0
1
2
3
4
5
6

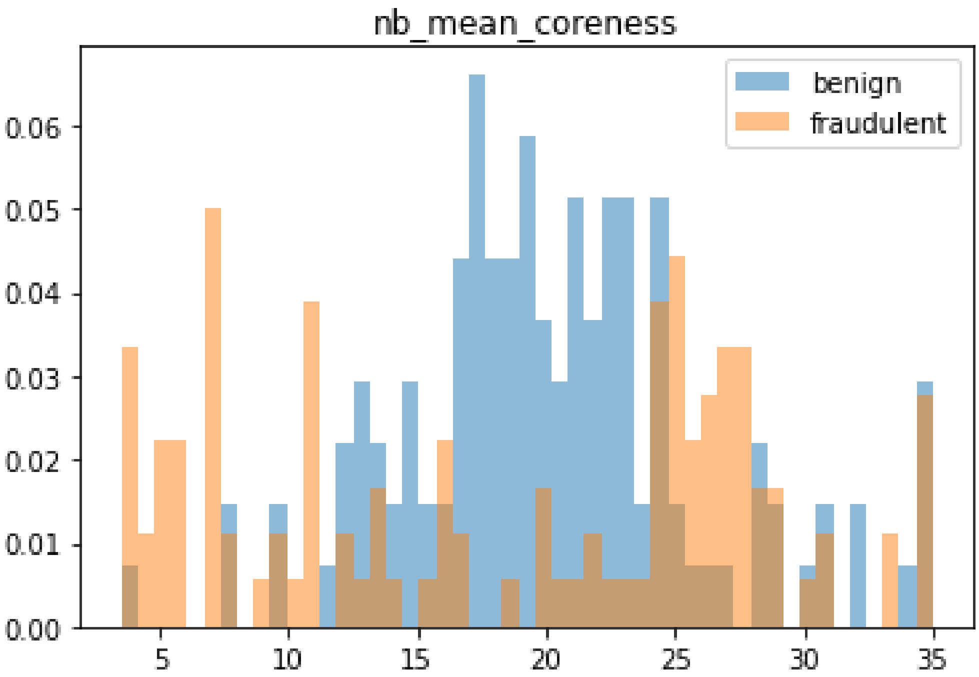
nb_mean_coreness
benign
fraudulent
0.06
0.05
0.04
0.03
0.02
0.01
0.00
5
10
15
20
25
30
35

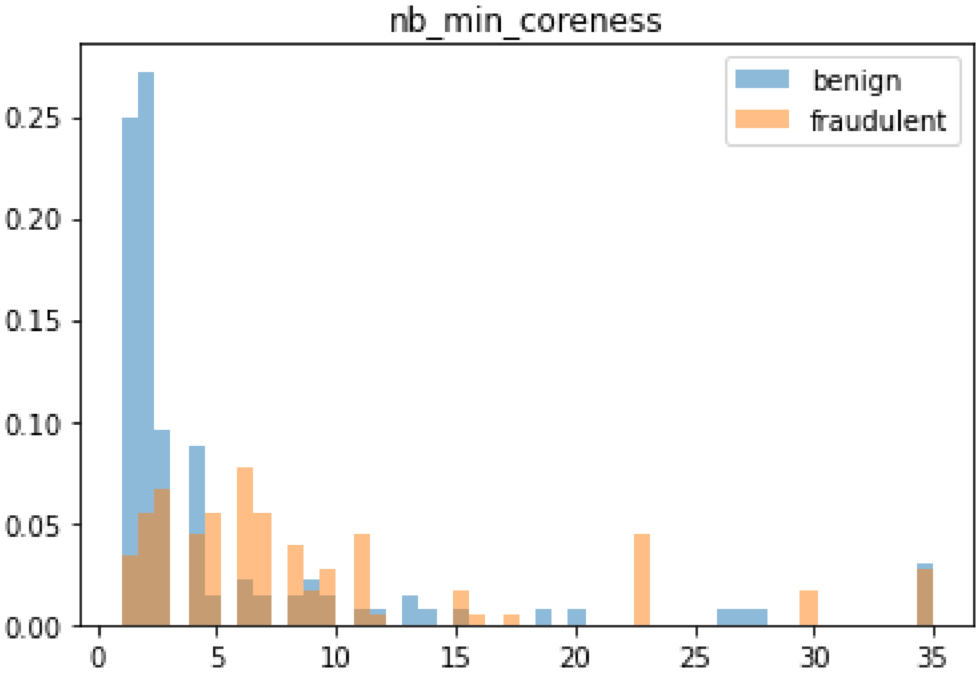
nb_min_coreness
benign
fraudulent
0.25
0.20
0.15
0.10
0.05
0.00
0
5
10
15
20
25
30
35

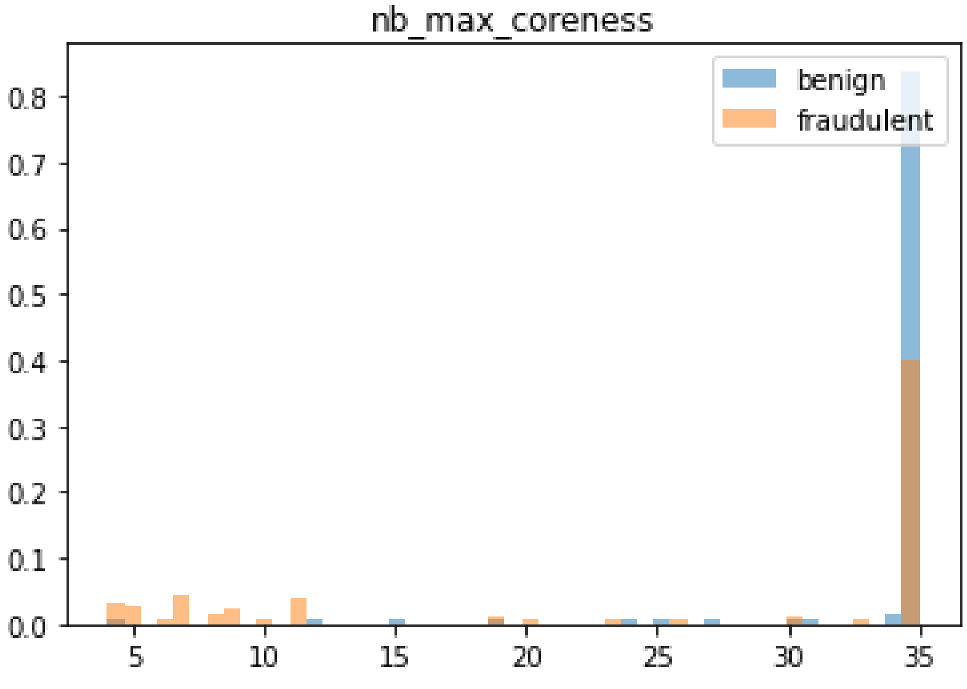
nb_max_coreness
benign
fraudulent
0.8
0.7
0.6
0.5
0.4
0.3
0.2
0.1
0.0
5
10
15
20
25
30
35

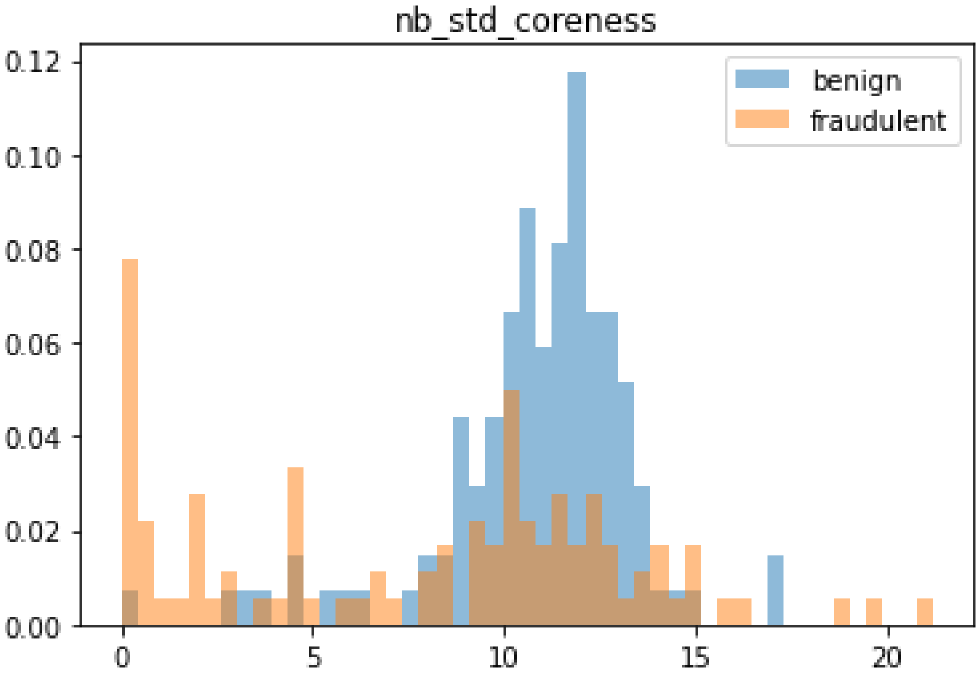
nb_std_coreness
benign
fraudulent
0.12
0.10
0.08
0.06
0.04
0.02
0.00
0
5
10
15
20

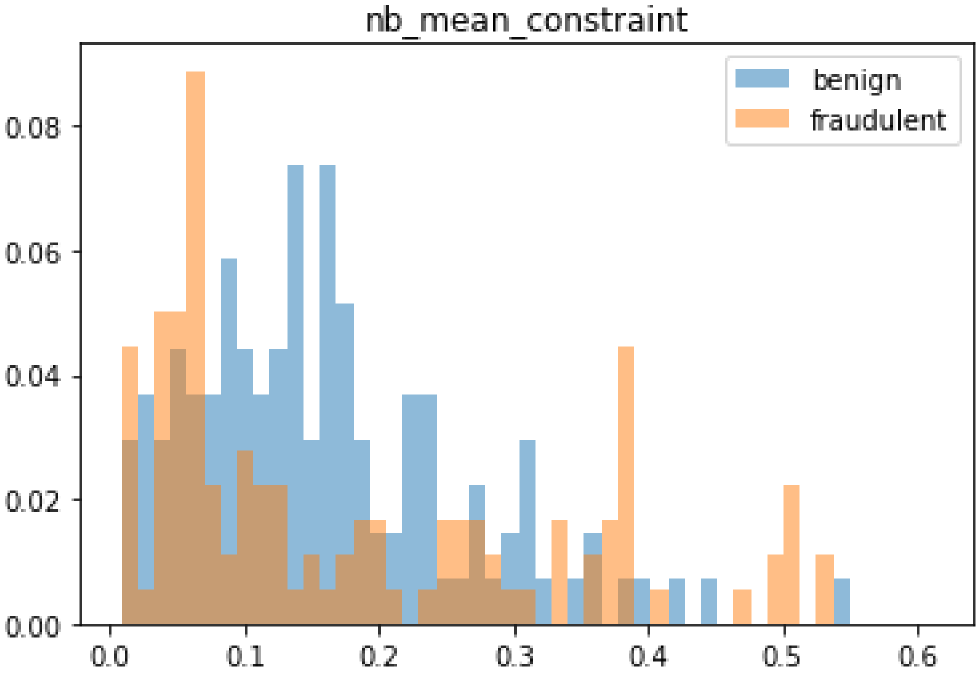
nb_mean_constraint
benign
fraudulent
0.08
0.06
0.04
0.02
0.00
0.0
0.1
0.2
0.3
0.4
0.5
0.6

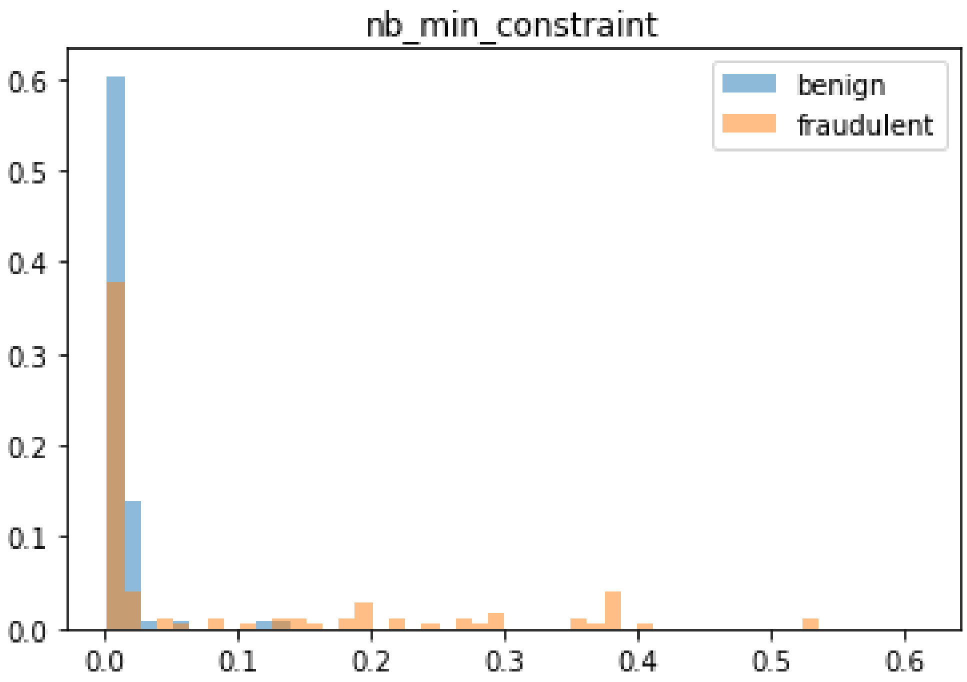

nb_min_constraint
benign
fraudulent
0.0
0.1
0.2
0.3
0.4
0.5
0.6

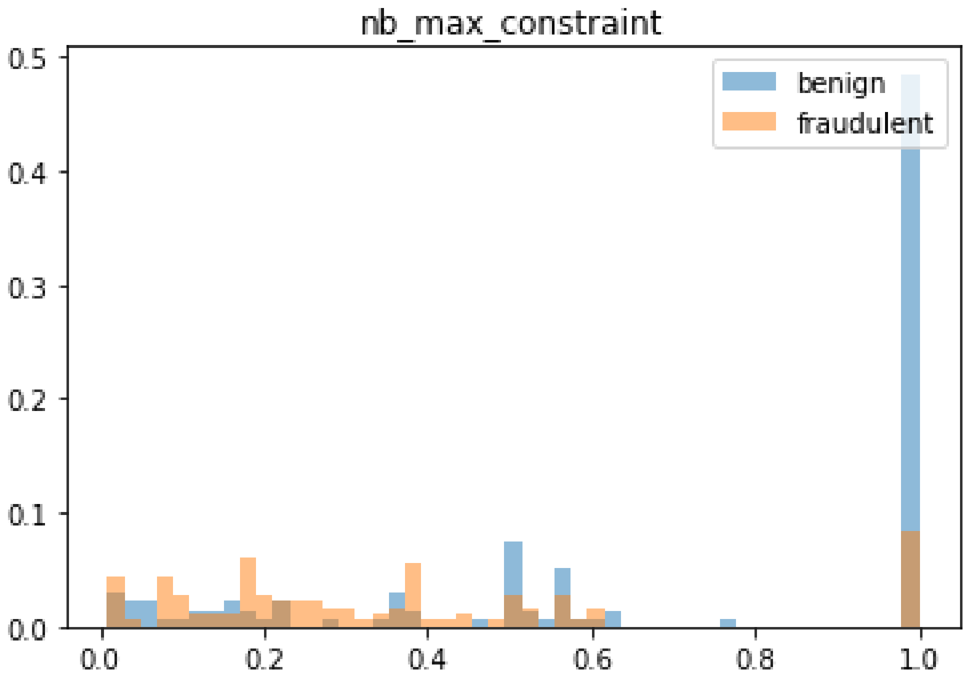

nb_max_constraint
benign
fraudulent
0.0
0.1
0.2
0.3
0.4
0.5
0.6
0.8
1.0

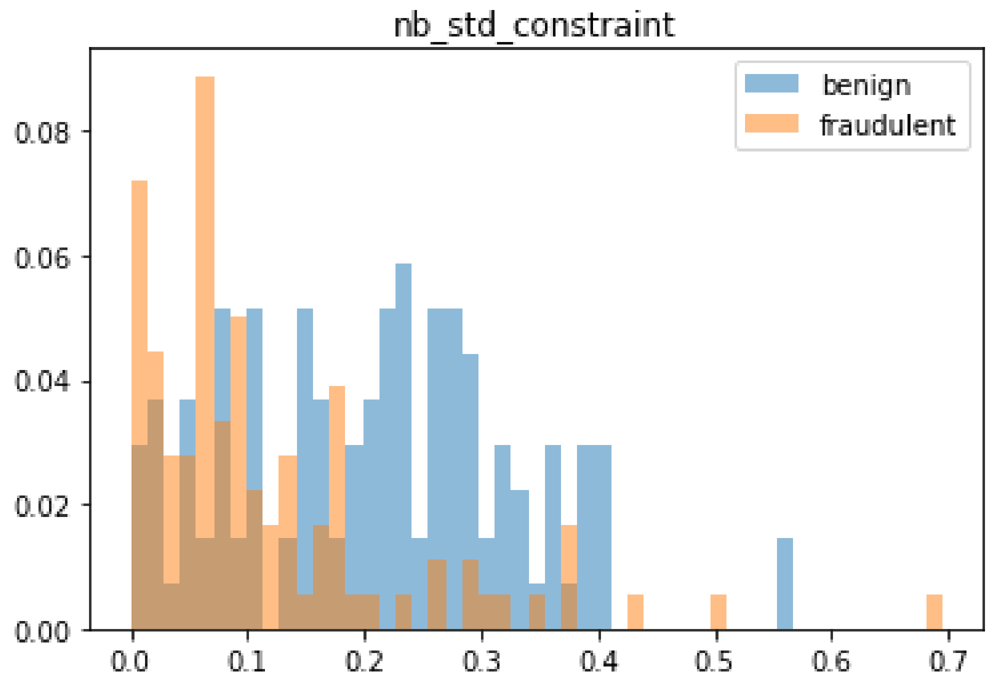
nb_std_constraint
benign
fraudulent
0.08
0.06
0.04
0.02
0.00
0.0
0.1
0.2
0.3
0.4
0.5
0.6
0.7

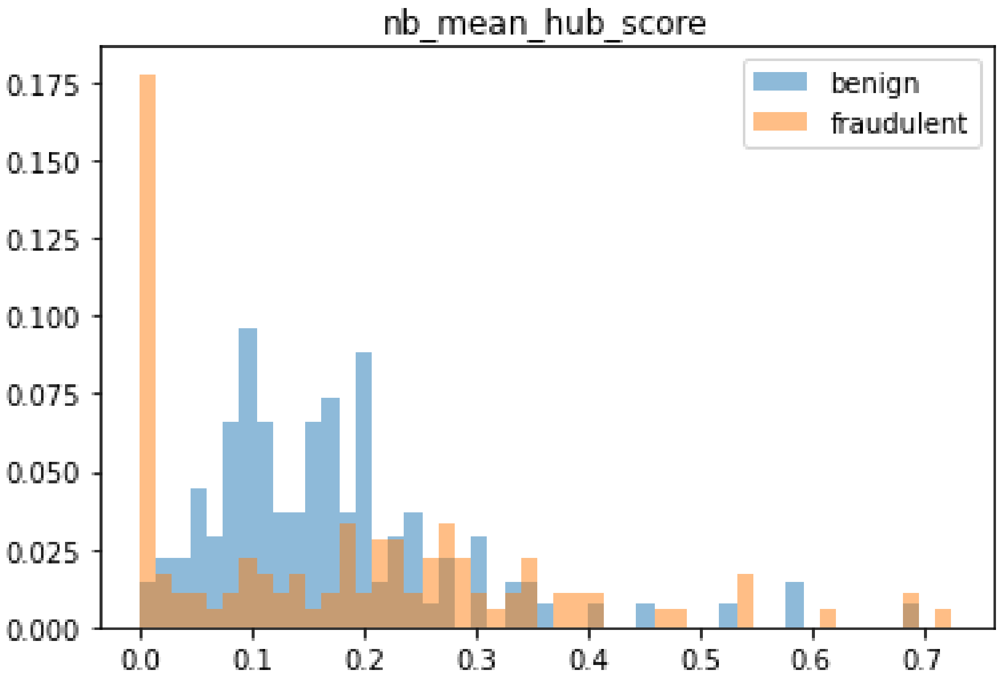
nb_mean_hub_score
benign
fraudulent
0.175
0.150
0.125
0.100
0.075
0.050
0.025
0.000
0.0
0.1
0.2
0.3
0.4
0.5
0.6
0.7

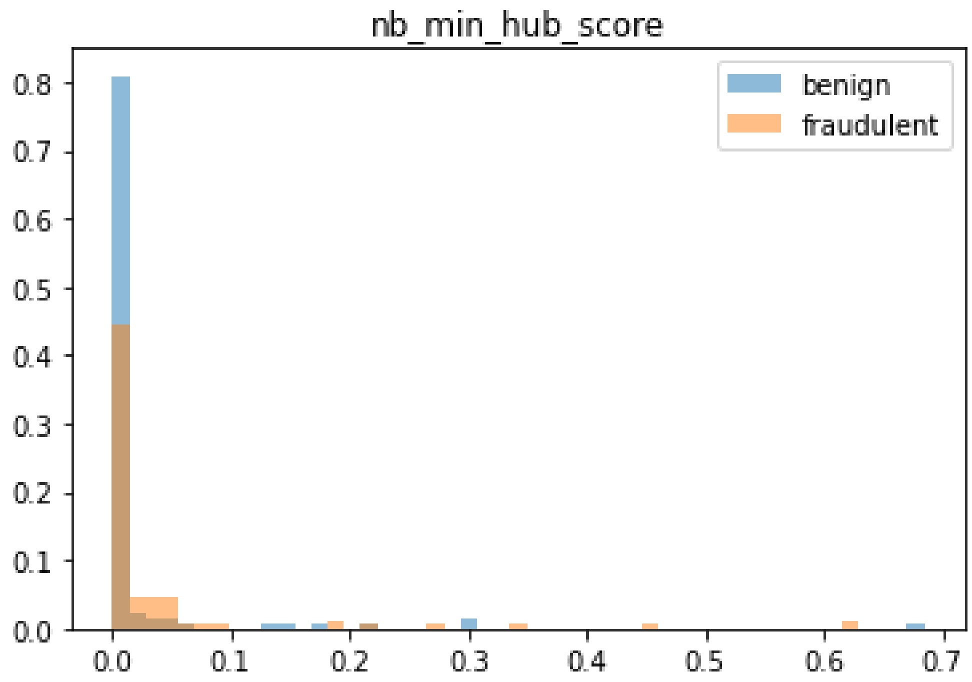
nb_min_hub_score
benign
fraudulent
0.0
0.1
0.2
0.3
0.4
0.5
0.6
0.7
0.8
0.0
0.1
0.2
0.3
0.4
0.5
0.6
0.7

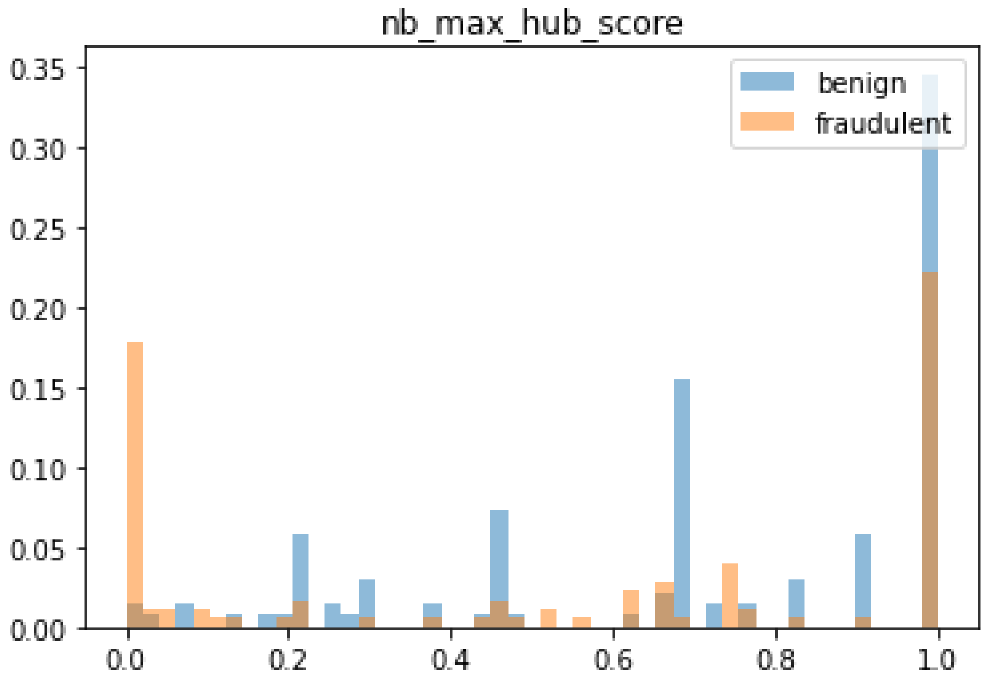
nb_max_hub_score
benign
fraudulent
0.00
0.05
0.10
0.15
0.20
0.25
0.30
0.35
0.0
0.2
0.4
0.6
0.8
1.0

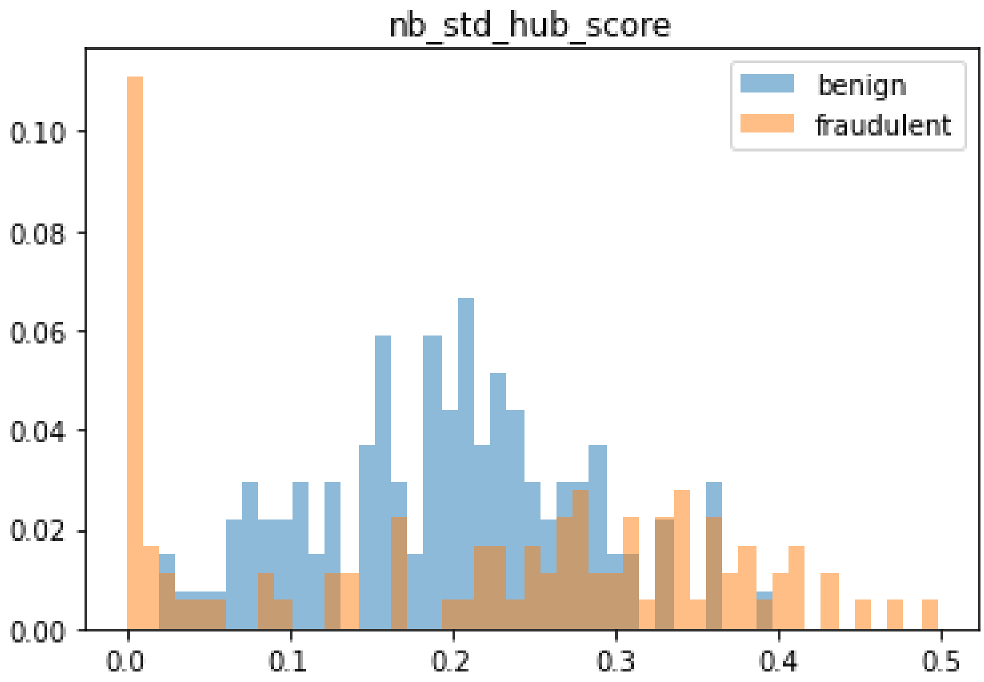
nb_std_hub_score
benign
fraudulent
0.00
0.02
0.04
0.06
0.08
0.10
0.0
0.1
0.2
0.3
0.4
0.5

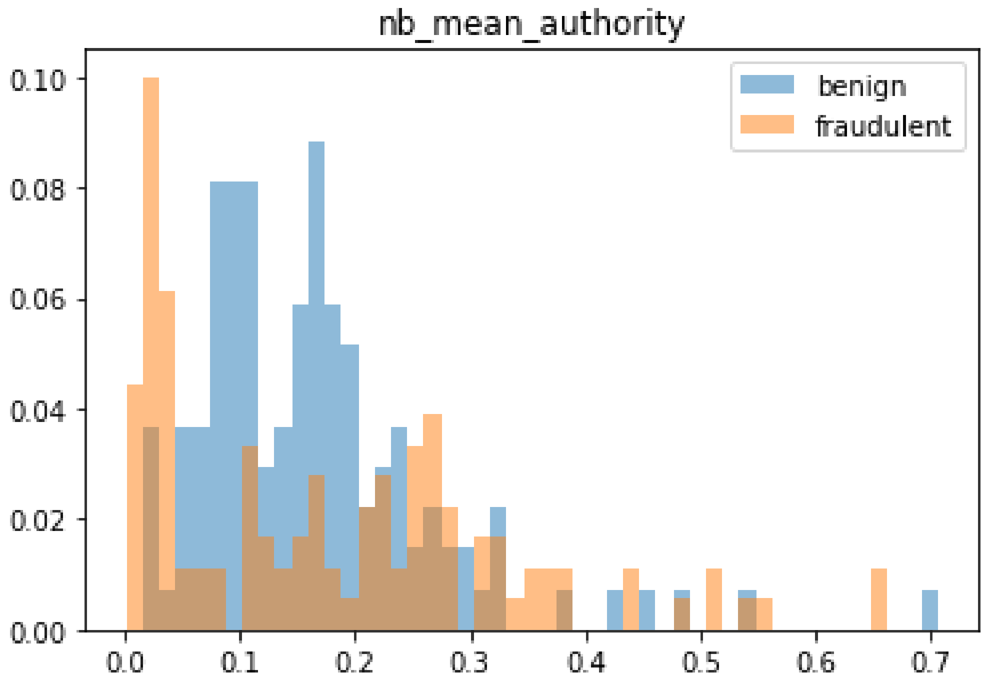
nb_mean_authority
benign
fraudulent
0.00
0.02
0.04
0.06
0.08
0.10
0.0
0.1
0.2
0.3
0.4
0.5
0.6
0.7

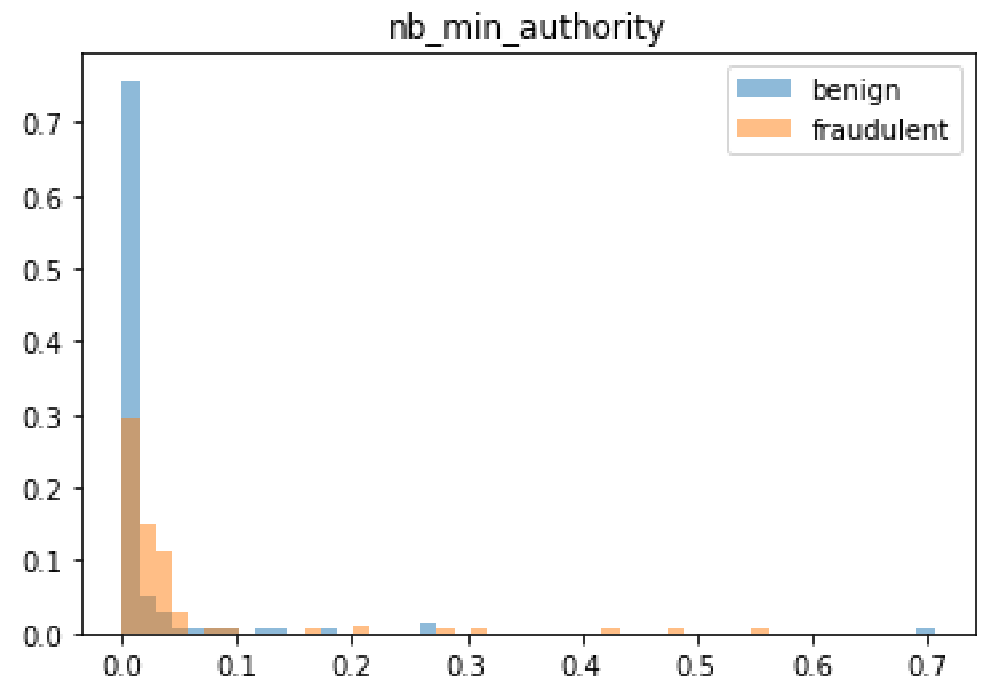
nb_min_authority
benign
fraudulent
0.7
0.6
0.5
0.4
0.3
0.2
0.1
0.0
0.0
0.1
0.2
0.3
0.4
0.5
0.6
0.7

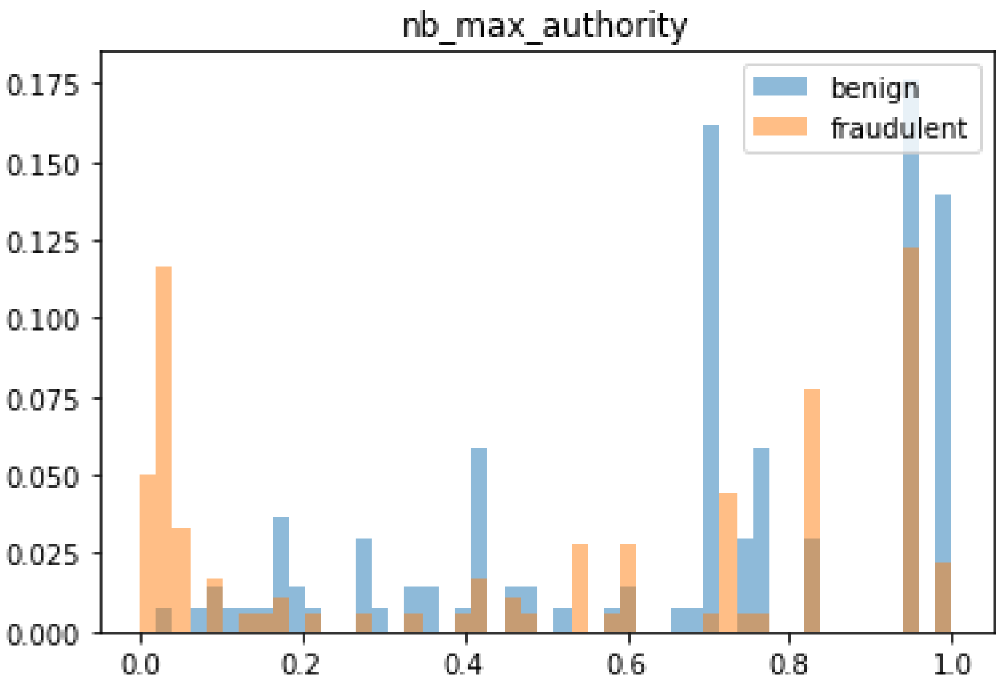
nb_max_authority
benign
fraudulent
0.175
0.150
0.125
0.100
0.075
0.050
0.025
0.000
0.0
0.2
0.4
0.6
0.8
1.0

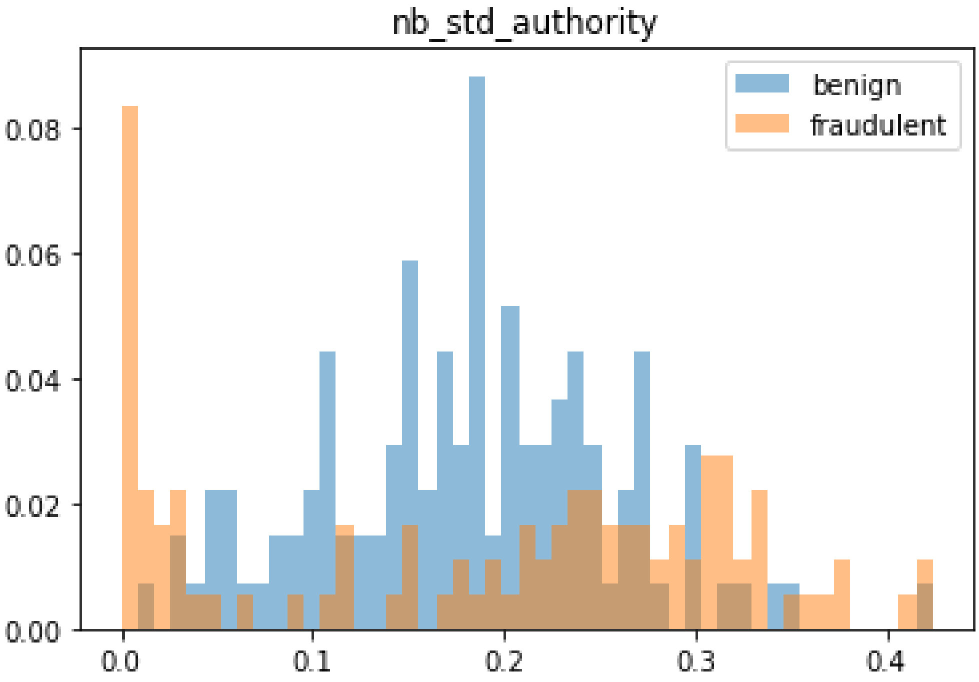
nb_std_authority
benign
fraudulent
0.08
0.06
0.04
0.02
0.00
0.0
0.1
0.2
0.3
0.4

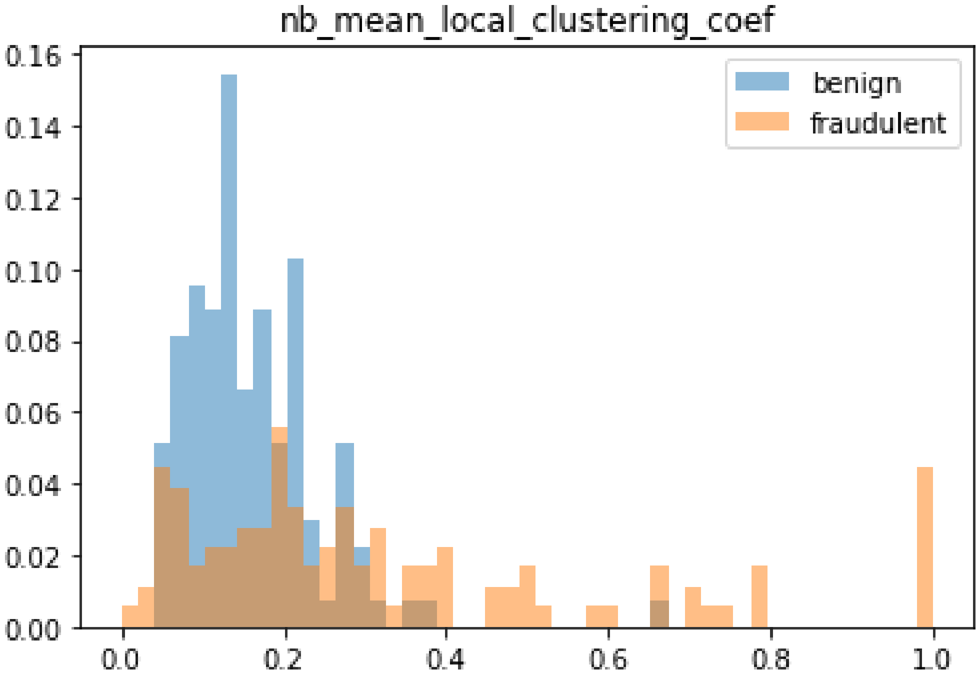
nb_mean_local_clustering_coef
benign
fraudulent
0.16
0.14
0.12
0.10
0.08
0.06
0.04
0.02
0.00
0.0
0.2
0.4
0.6
0.8
1.0

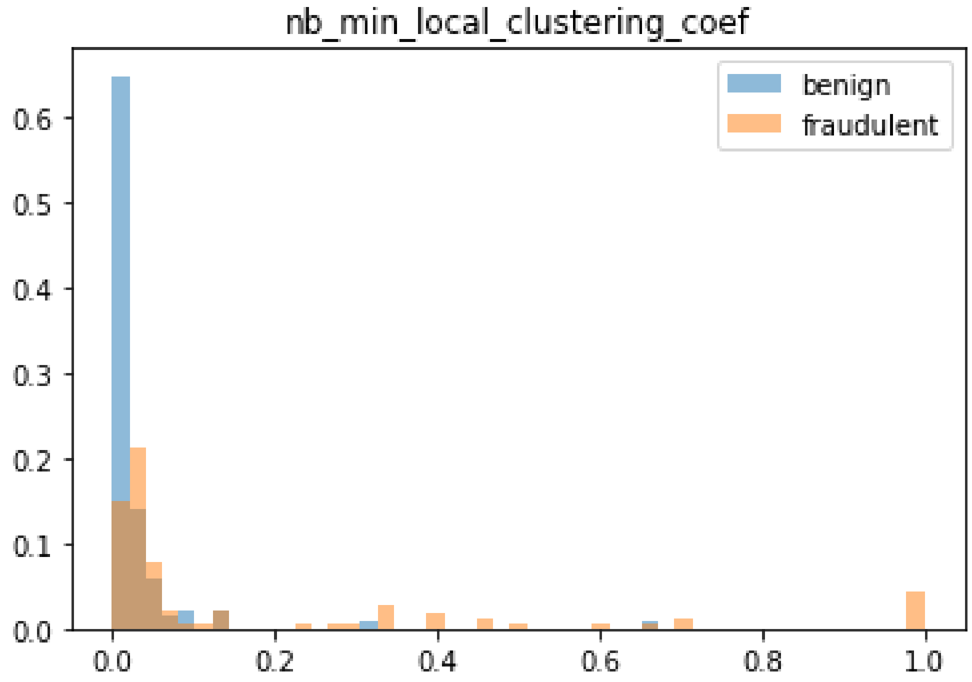
nb_min_local_clustering_coef
benign
fraudulent
0.6
0.5
0.4
0.3
0.2
0.1
0.0
0.0
0.2
0.4
0.6
0.8
1.0

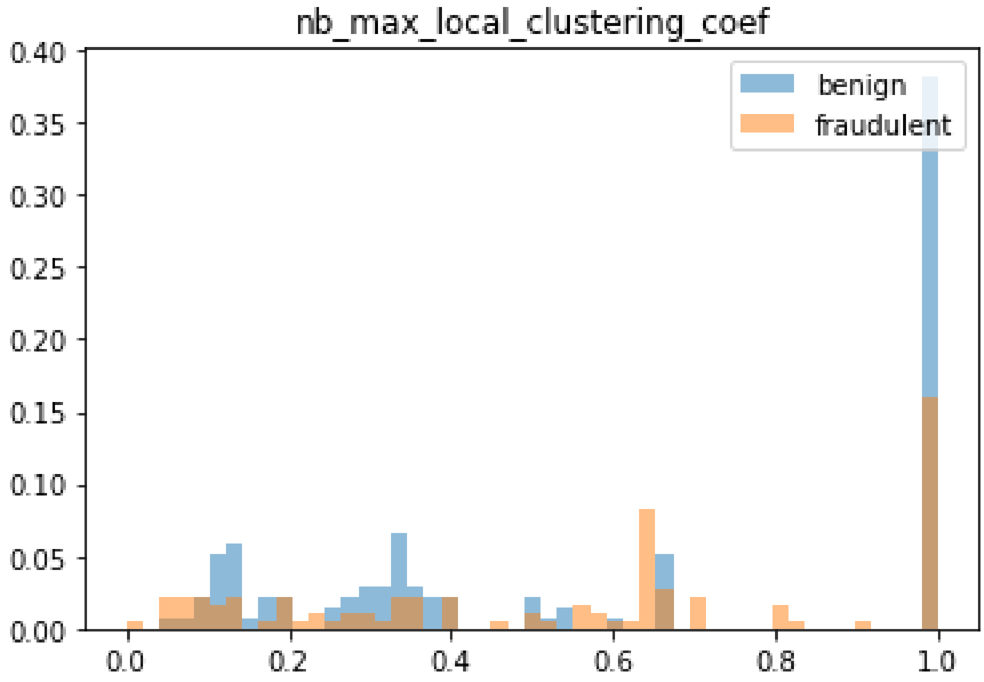
nb_max_local_clustering_coef
benign
fraudulent
0.40
0.35
0.30
0.25
0.20
0.15
0.10
0.05
0.00
0.0
0.2
0.4
0.6
0.8
1.0

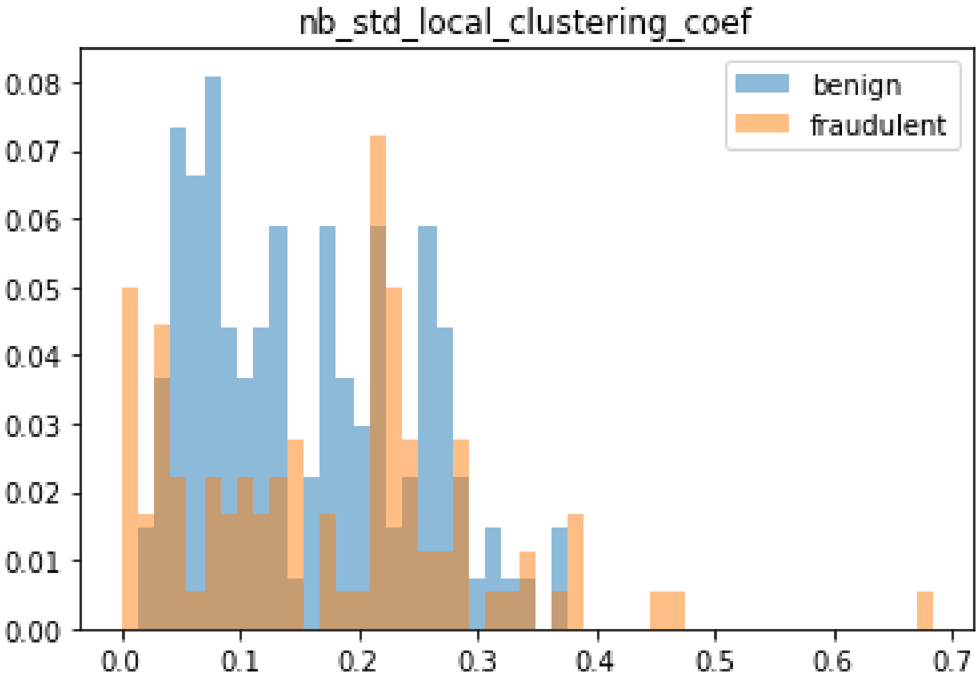
nb_std_local_clustering_coef
benign
fraudulent
0.00
0.01
0.02
0.03
0.04
0.05
0.06
0.07
0.08
0.0
0.1
0.2
0.3
0.4
0.5
0.6
0.7

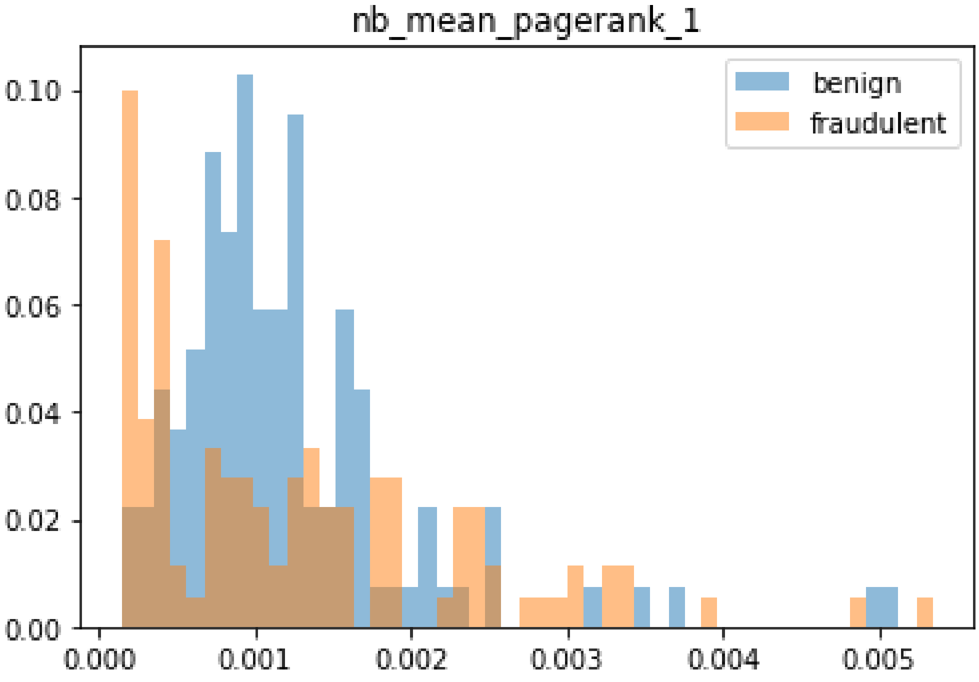
nb_mean_pagerank_1
benign
fraudulent
0.00
0.02
0.04
0.06
0.08
0.10
0.000
0.001
0.002
0.003
0.004
0.005

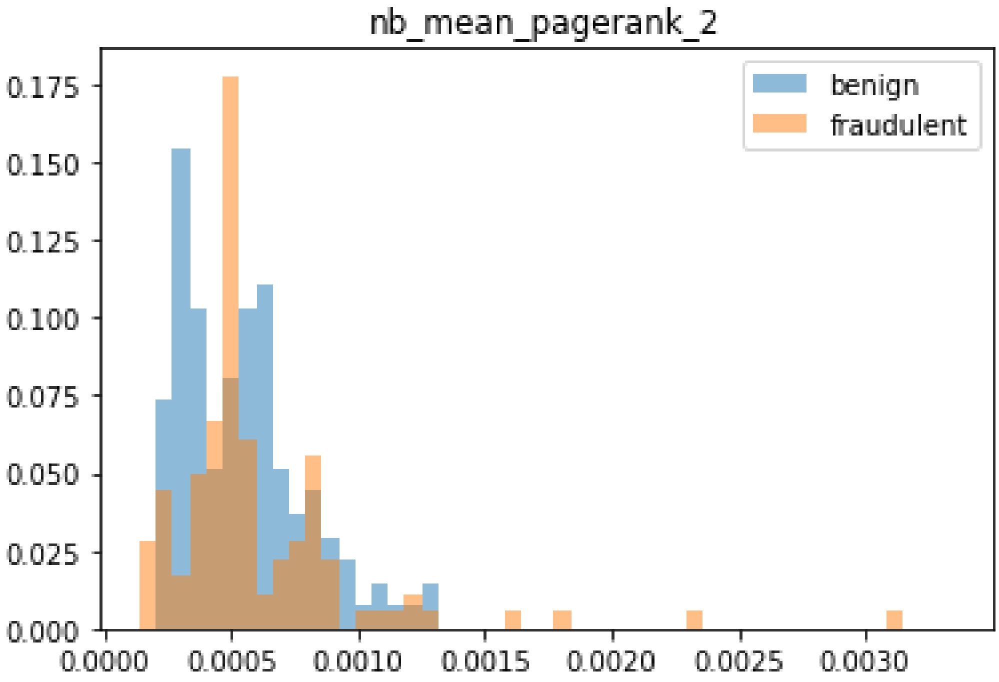
nb_mean_pagerank_2
benign
fraudulent
0.175
0.150
0.125
0.100
0.075
0.050
0.025
0.000
0.0000
0.0005
0.0010
0.0015
0.0020
0.0025
0.0030

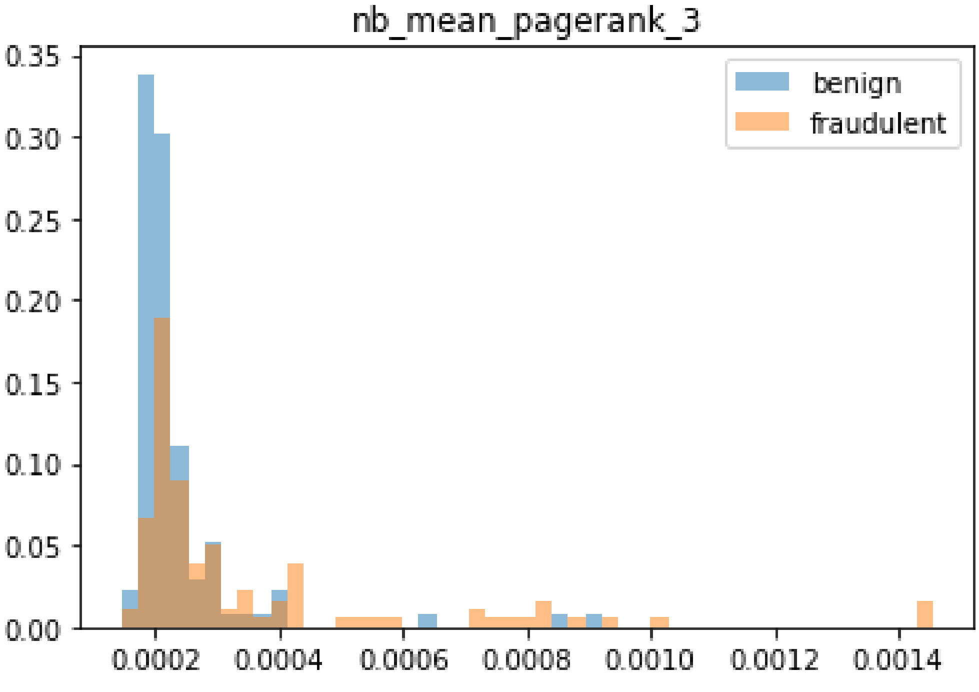
nb_mean_pagerank_3
benign
fraudulent
0.35
0.30
0.25
0.20
0.15
0.10
0.05
0.00
0.0002
0.0004
0.0006
0.0008
0.0010
0.0012
0.0014

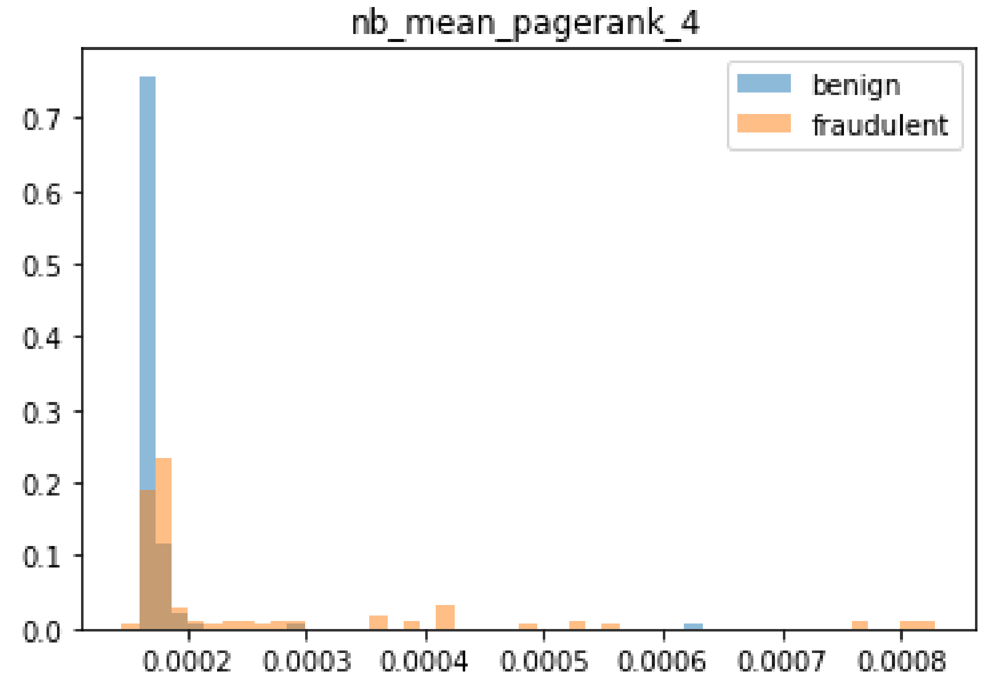
nb_mean_pagerank_4
benign
fraudulent
0.7
0.6
0.5
0.4
0.3
0.2
0.1
0.0
0.0002
0.0003
0.0004
0.0005
0.0006
0.0007
0.0008

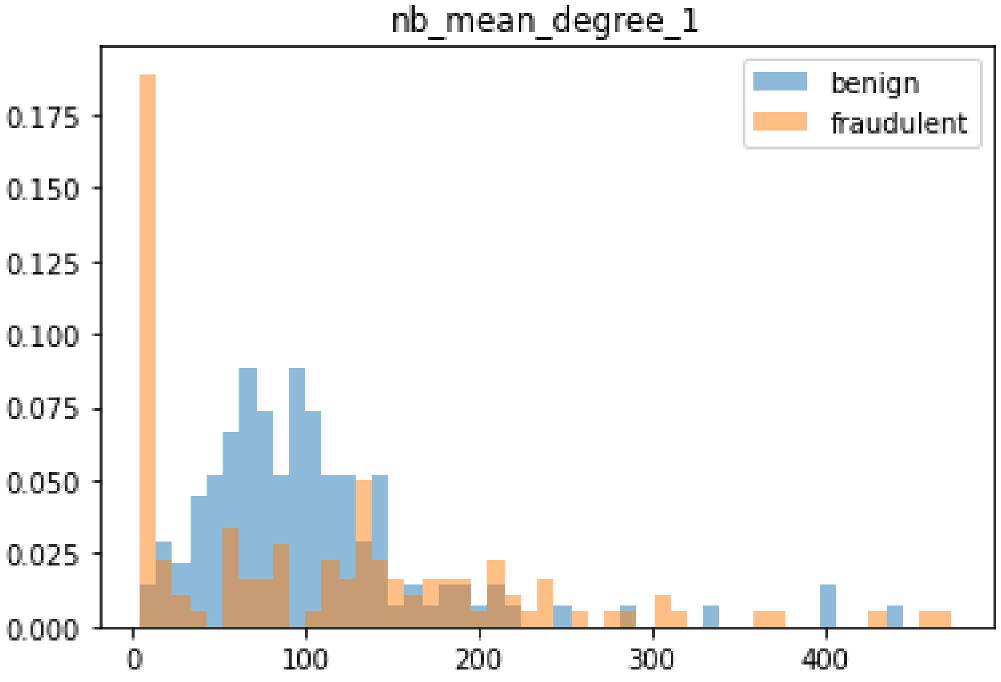
nb_mean_degree_1
benign
fraudulent
0.175
0.150
0.125
0.100
0.075
0.050
0.025
0.000
0
100
200
300
400

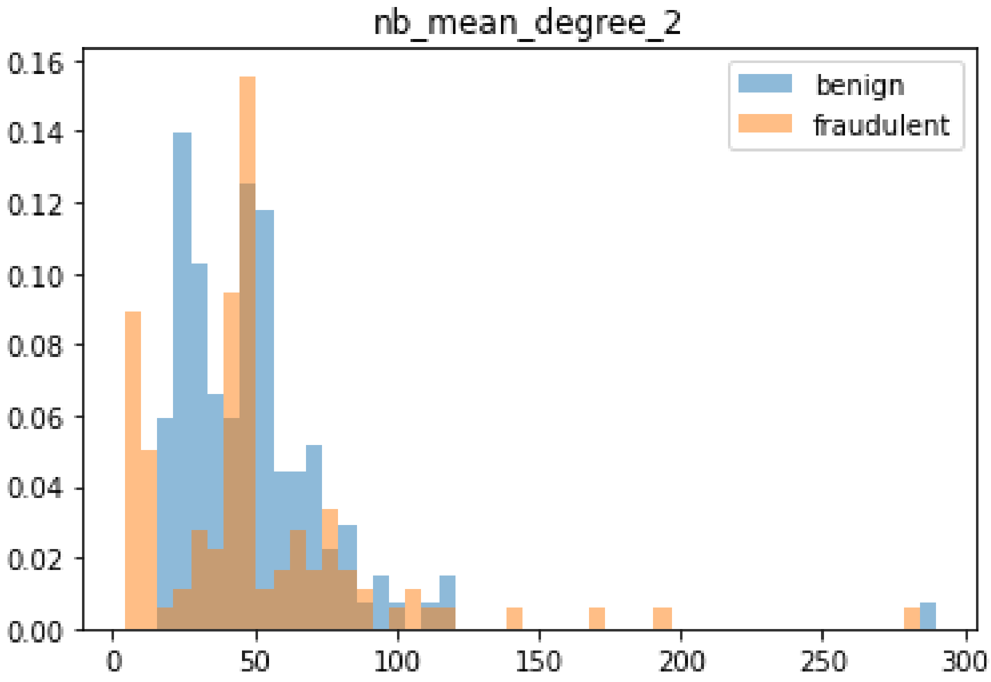
nb_mean_degree_2
benign
fraudulent
0.16
0.14
0.12
0.10
0.08
0.06
0.04
0.02
0.00
0
50
100
150
200
250
300

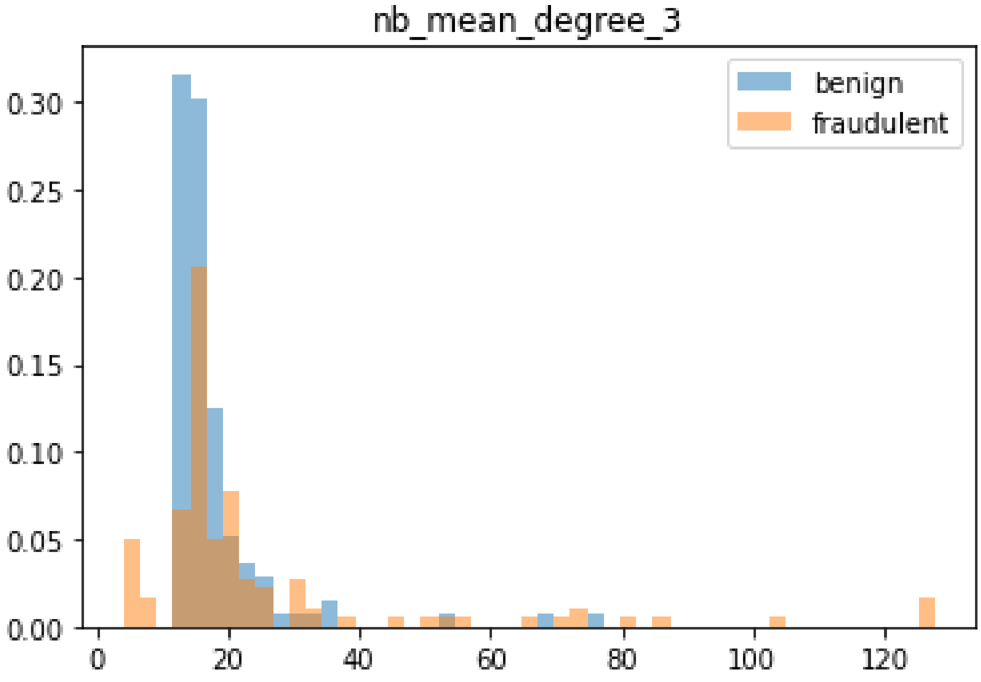
nb_mean_degree_3
benign
fraudulent
0.30
0.25
0.20
0.15
0.10
0.05
0.00
0
20
40
60
80
100
120

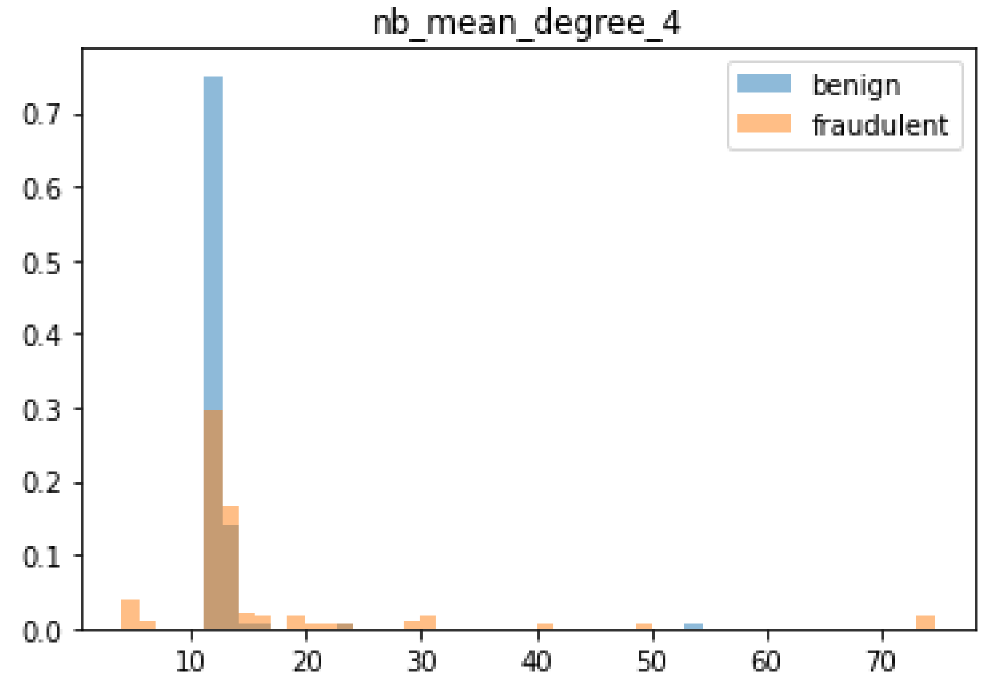
nb_mean_degree_4
benign
fraudulent
0.7
0.6
0.5
0.4
0.3
0.2
0.1
0.0
10
20
30
40
50
60
70

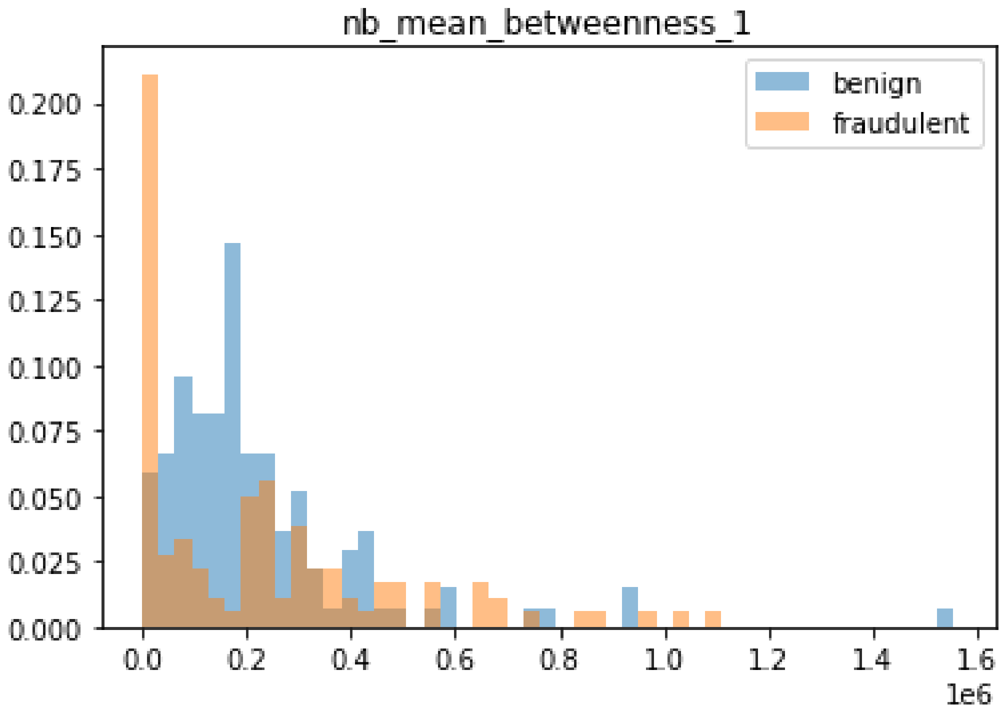
nb_mean_betweenness_1
benign
fraudulent
0.200
0.175
0.150
0.125
0.100
0.075
0.050
0.025
0.000
0.0
0.2
0.4
0.6
0.8
1.0
1.2
1.4
1.6
1e6

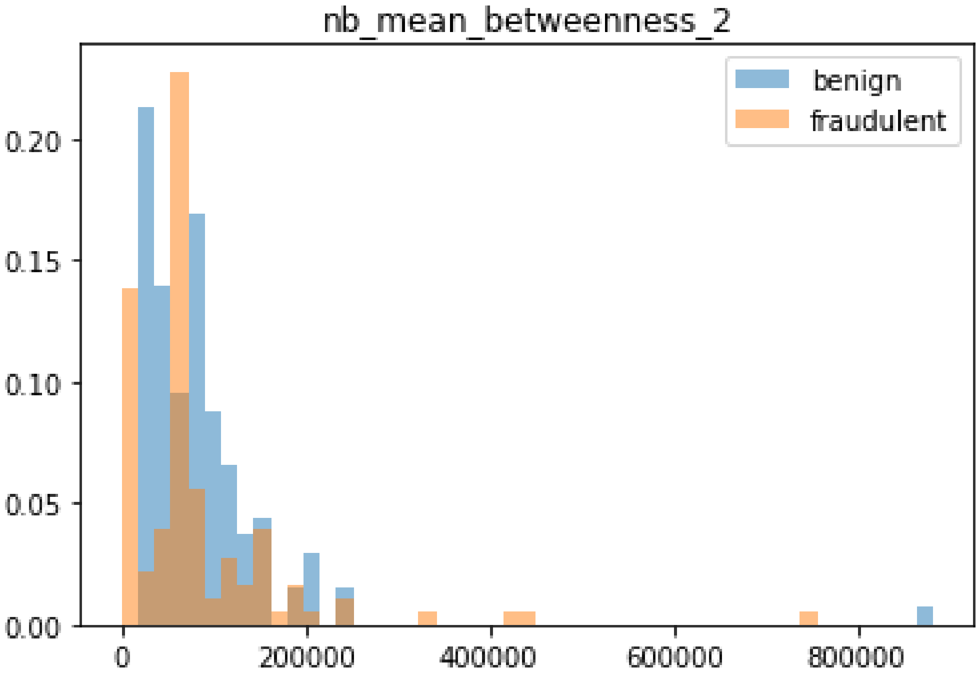
nb_mean_betweenness_2
benign
fraudulent
0.20
0.15
0.10
0.05
0.00
0
200000
400000
600000
800000

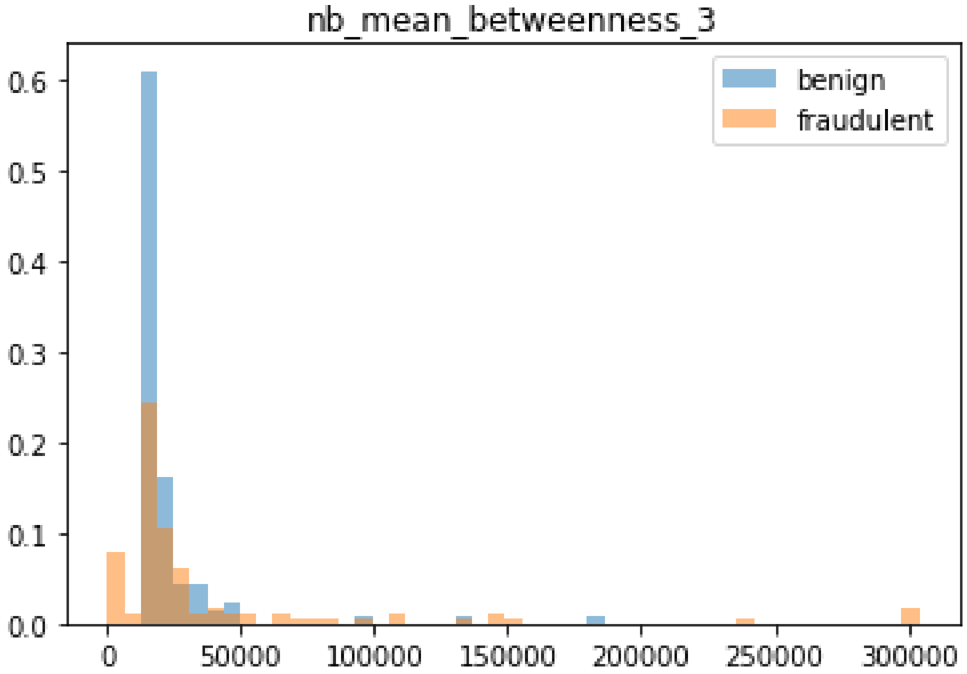
nb_mean_betweenness_3
benign
fraudulent
0.6
0.5
0.4
0.3
0.2
0.1
0.0
0
50000
100000
150000
200000
250000
300000

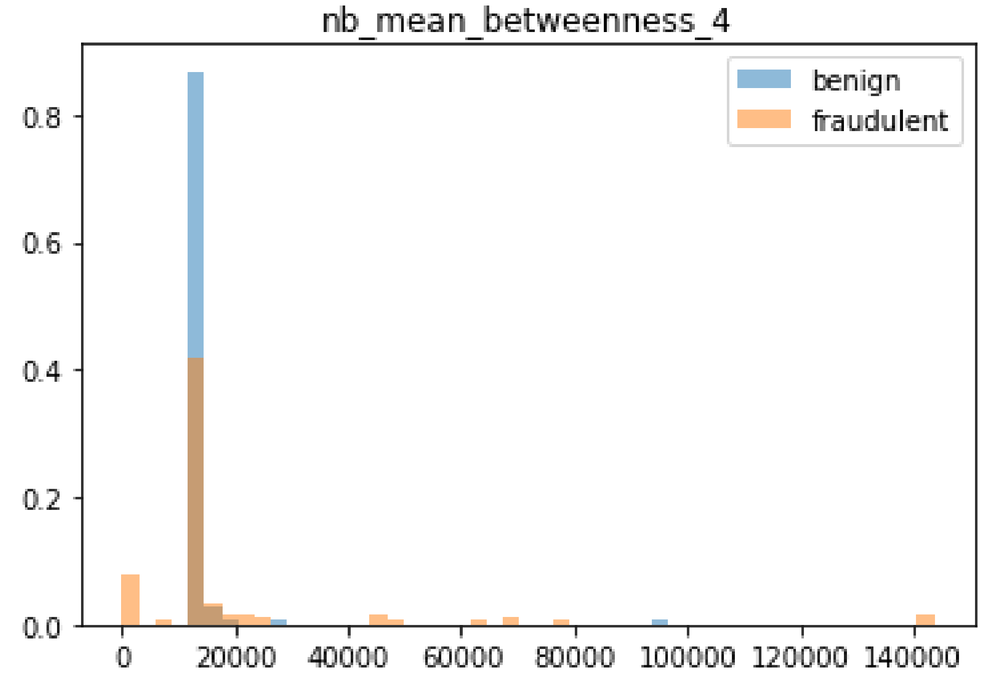
nb_mean_betweenness_4
benign
fraudulent
0.8
0.6
0.4
0.2
0.0
0
20000
40000
60000
80000
100000
120000
140000

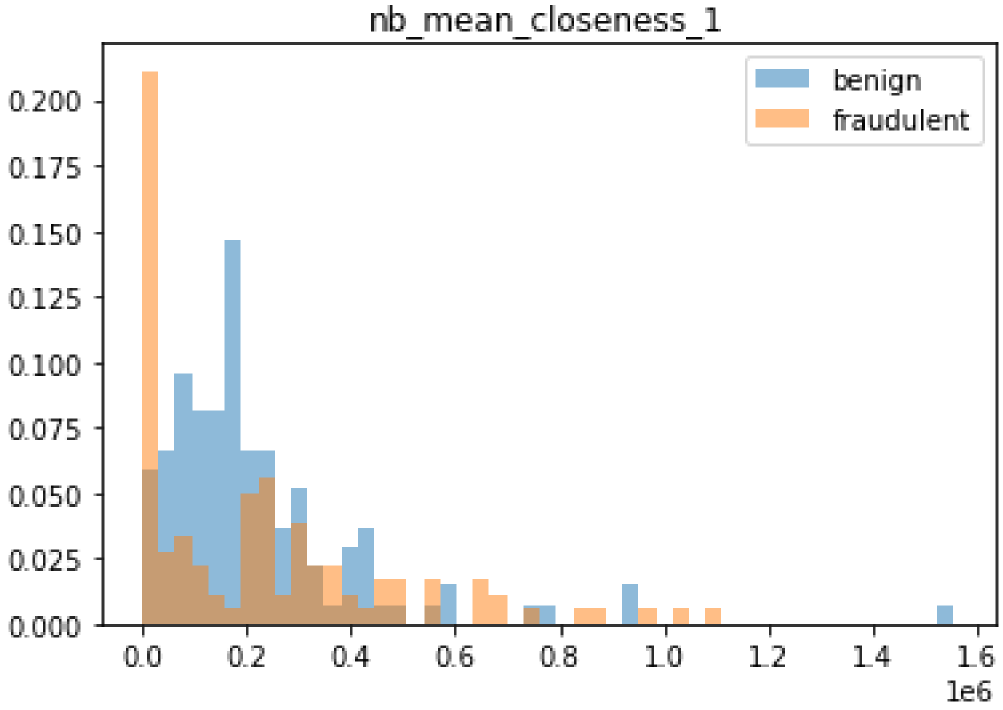
nb_mean_closeness_1
benign
fraudulent
0.200
0.175
0.150
0.125
0.100
0.075
0.050
0.025
0.000
0.0
0.2
0.4
0.6
0.8
1.0
1.2
1.4
1.6
1e6

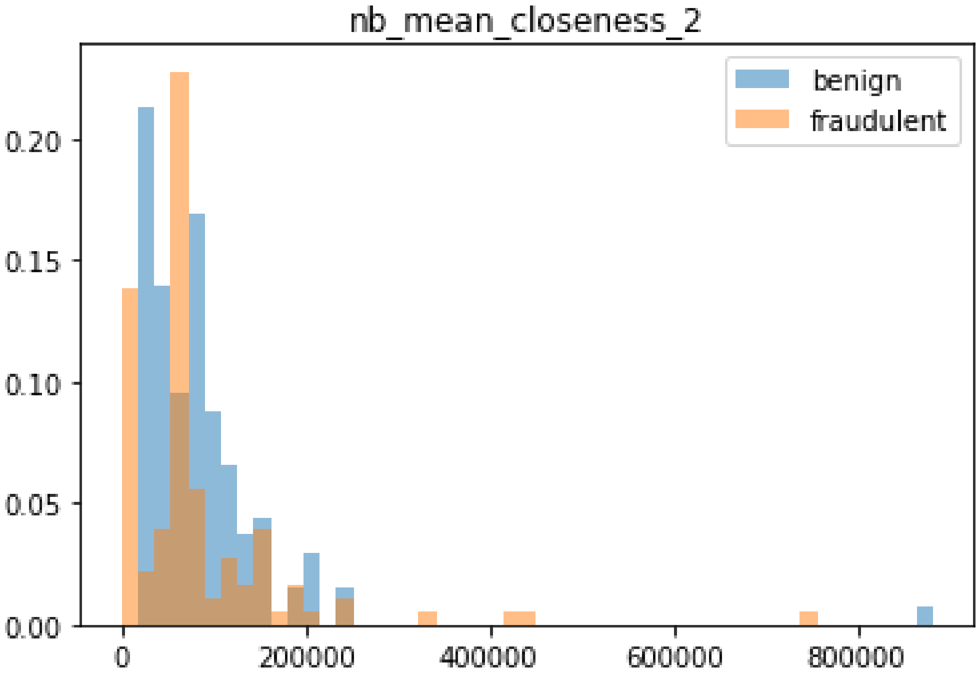
nb_mean_closeness_2
benign
fraudulent
0.20
0.15
0.10
0.05
0.00
0
200000
400000
600000
800000

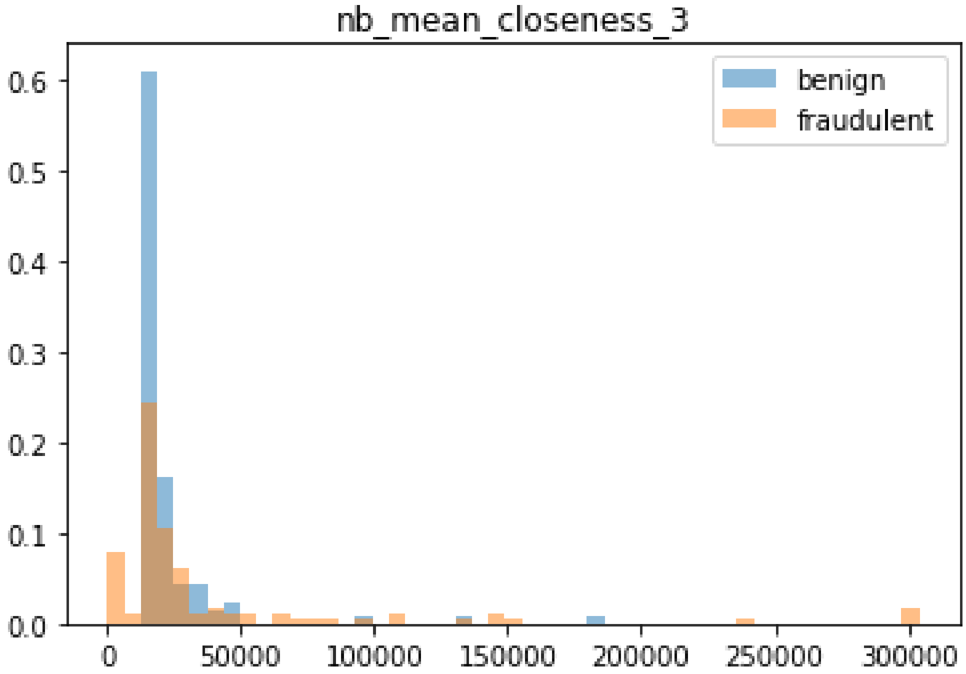
nb_mean_closeness_3
benign
fraudulent
0.6
0.5
0.4
0.3
0.2
0.1
0.0
0
50000
100000
150000
200000
250000
300000

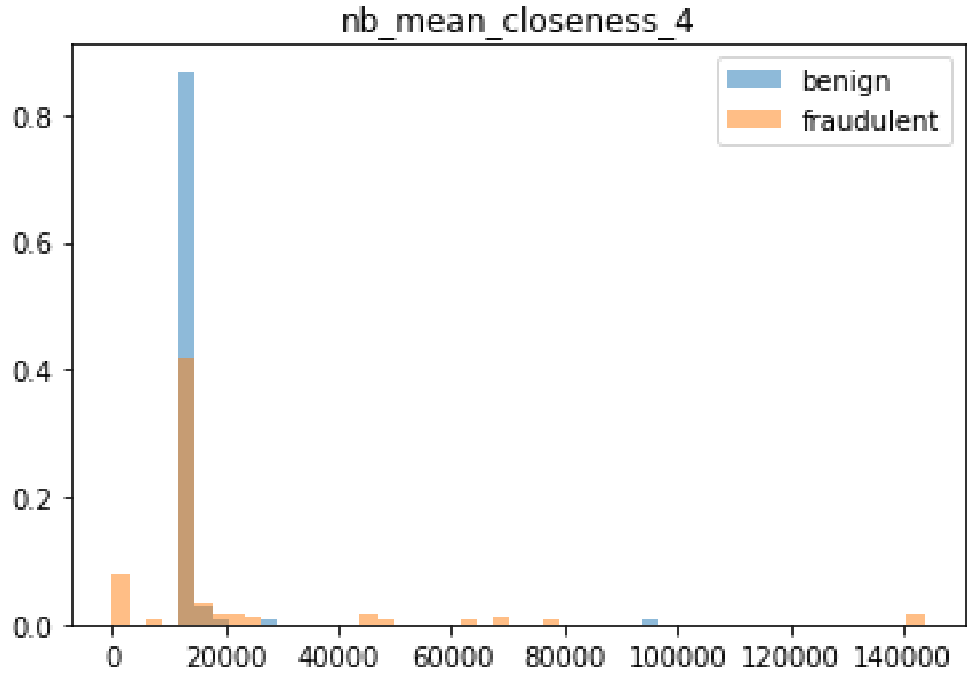

nb_mean_closeness_4
benign
fraudulent
0.0
0.2
0.4
0.6
0.8
0
20000
40000
60000
80000
100000
120000
140000

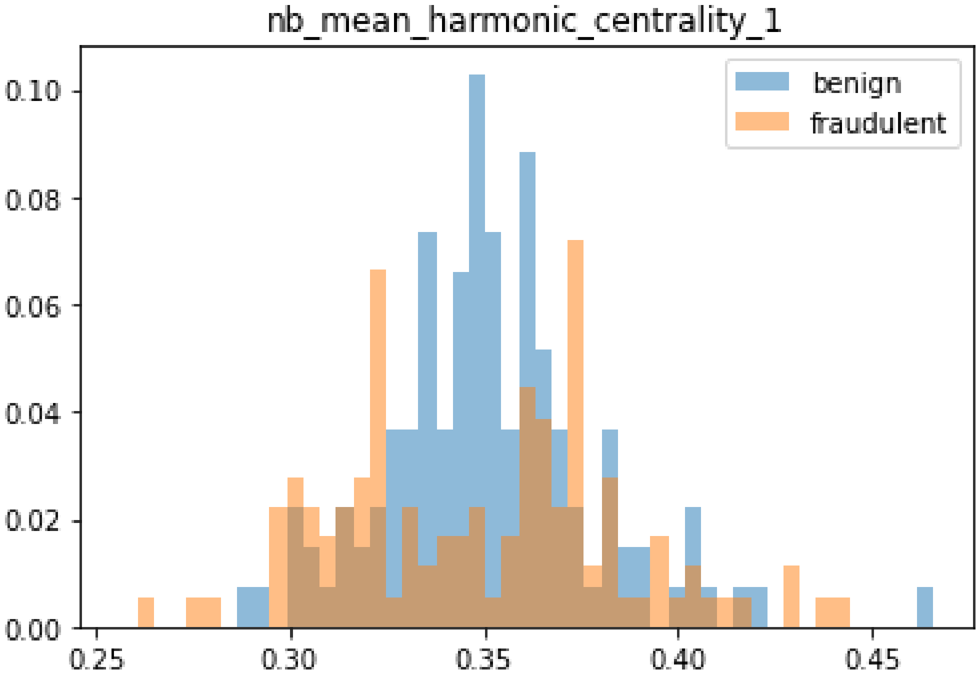

nb_mean_harmonic_centrality_1
benign
fraudulent
0.00
0.02
0.04
0.06
0.08
0.10
0.25
0.30
0.35
0.40
0.45

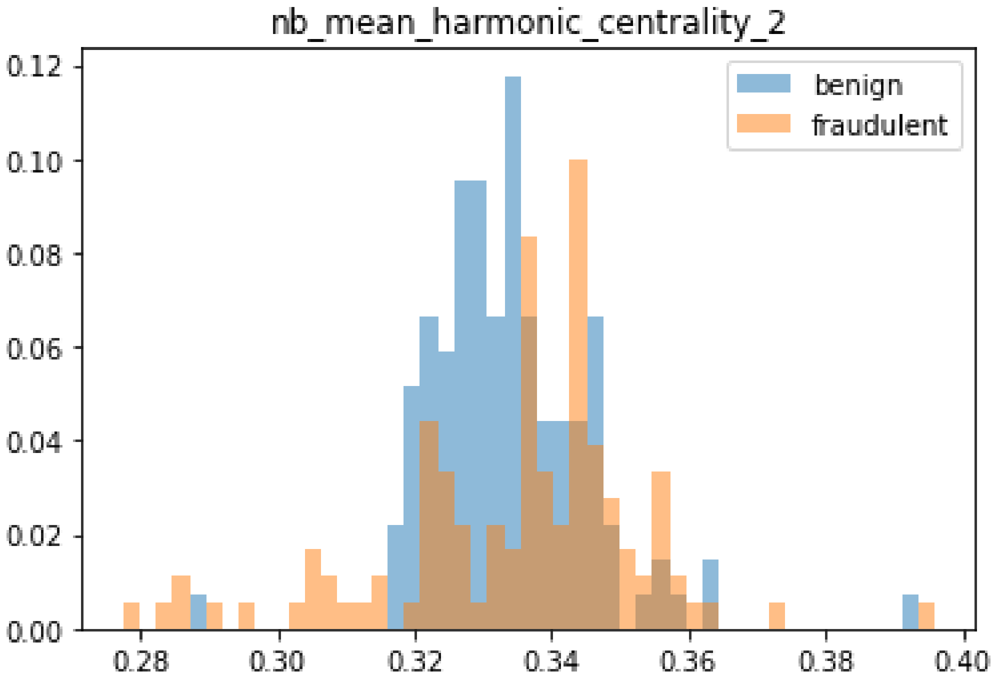
nb_mean_harmonic_centrality_2
benign
fraudulent
0.12
0.10
0.08
0.06
0.04
0.02
0.00
0.28
0.30
0.32
0.34
0.36
0.38
0.40

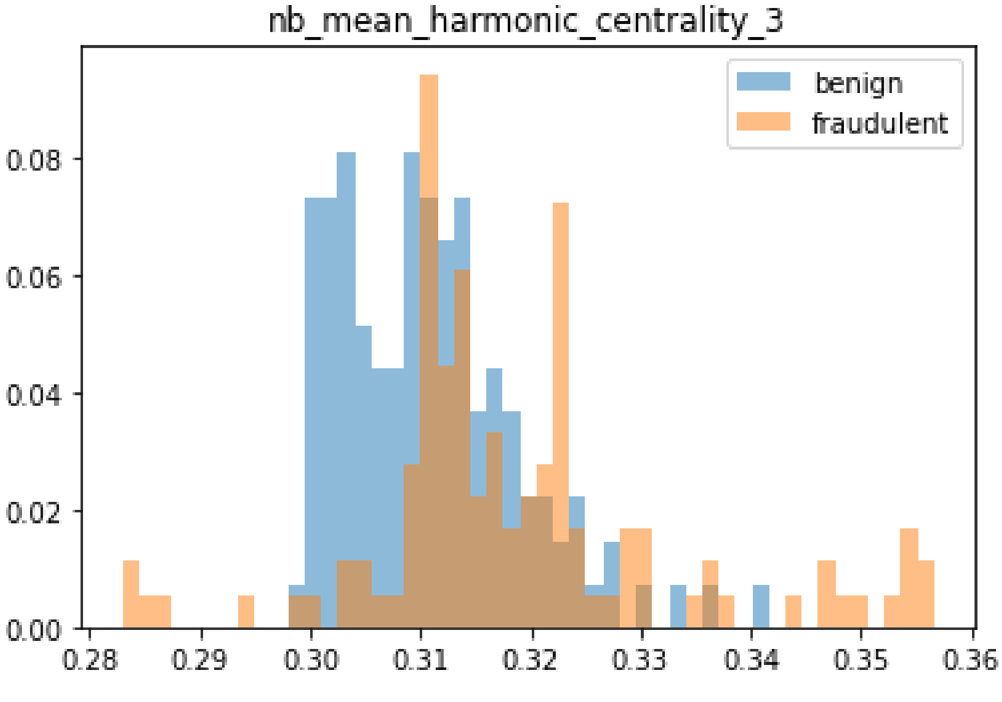
nb_mean_harmonic_centrality_3
benign
fraudulent
0.08
0.06
0.04
0.02
0.00
0.28
0.29
0.30
0.31
0.32
0.33
0.34
0.35
0.36

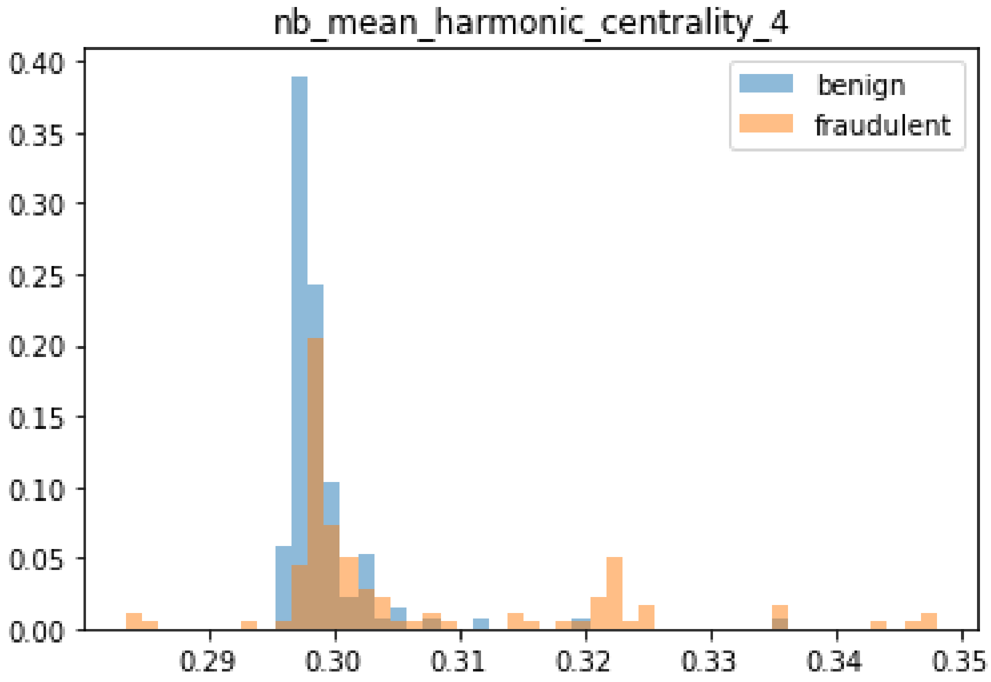

nb_mean_harmonic_centrality_4
0.40
0.35
0.30
0.25
0.20
0.15
0.10
0.05
0.00
0.29
0.30
0.31
0.32
0.33
0.34
0.35
benign
fraudulent

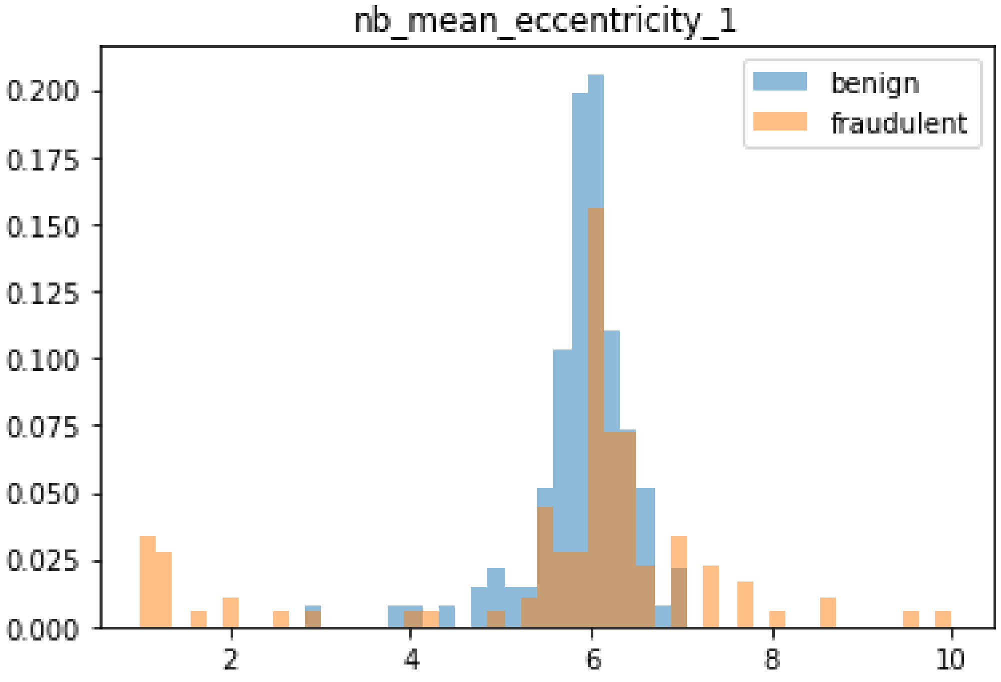

nb_mean_eccentricity_1
0.200
0.175
0.150
0.125
0.100
0.075
0.050
0.025
0.000
2
4
6
8
10
benign
fraudulent

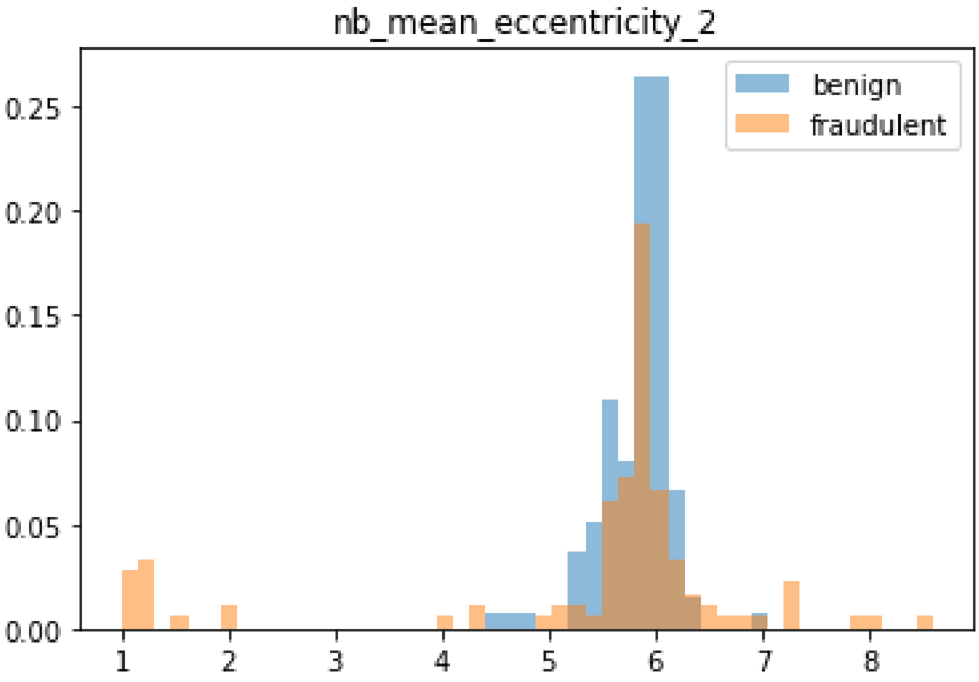
nb_mean_eccentricity_2
benign
fraudulent
0.25
0.20
0.15
0.10
0.05
0.00
1
2
3
4
5
6
7
8

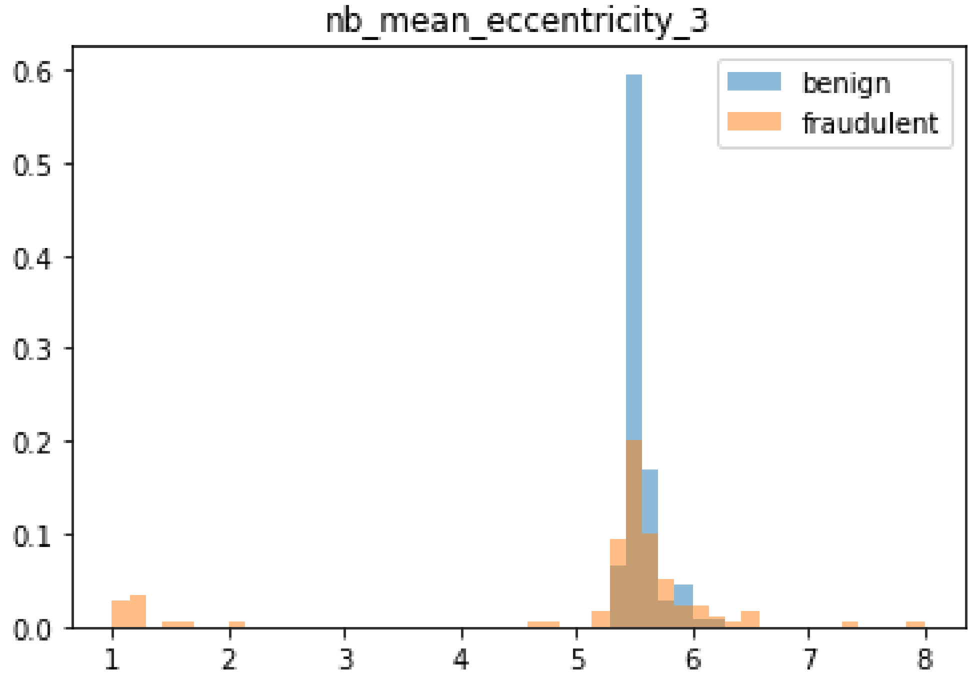
nb_mean_eccentricity_3
benign
fraudulent
0.6
0.5
0.4
0.3
0.2
0.1
0.0
1
2
3
4
5
6
7
8

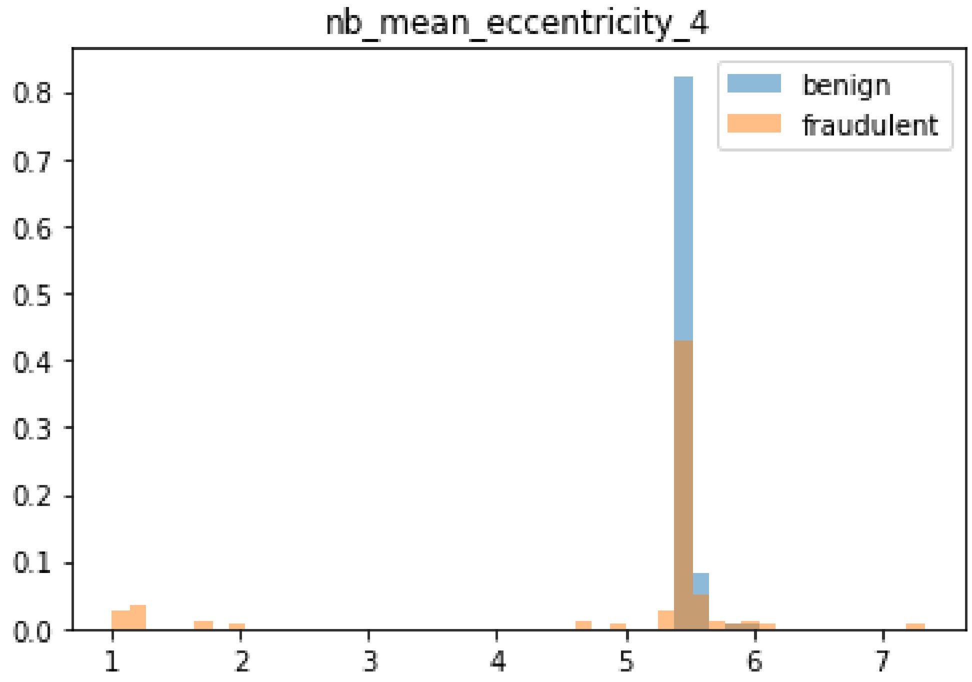
nb_mean_eccentricity_4
benign
fraudulent
0.8
0.7
0.6
0.5
0.4
0.3
0.2
0.1
0.0
1
2
3
4
5
6
7

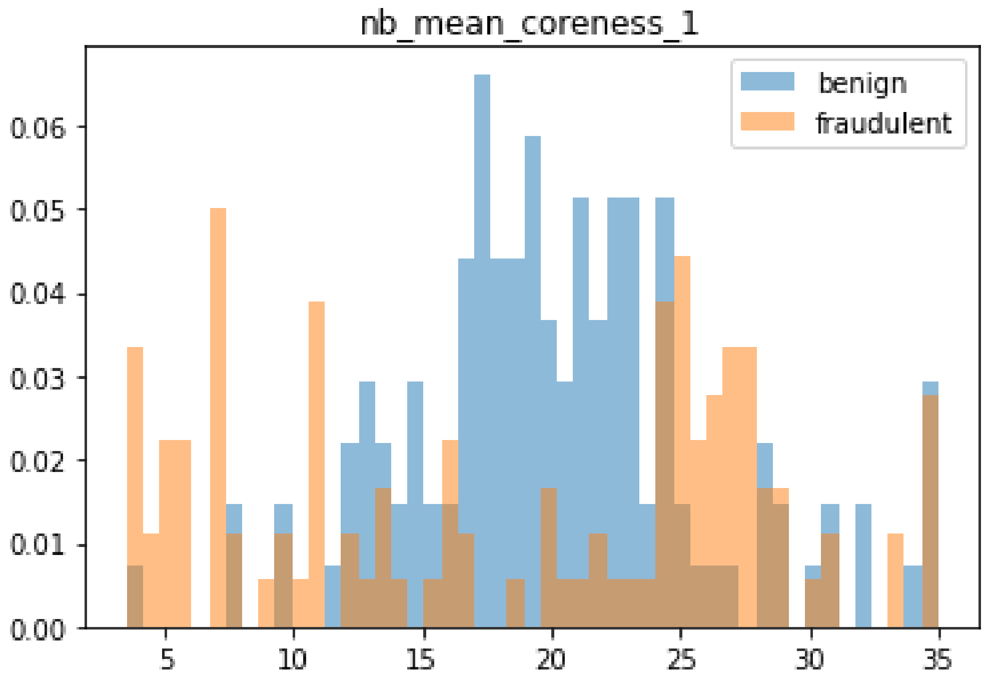
nb_mean_coreness_1
benign
fraudulent
0.06
0.05
0.04
0.03
0.02
0.01
0.00
5
10
15
20
25
30
35

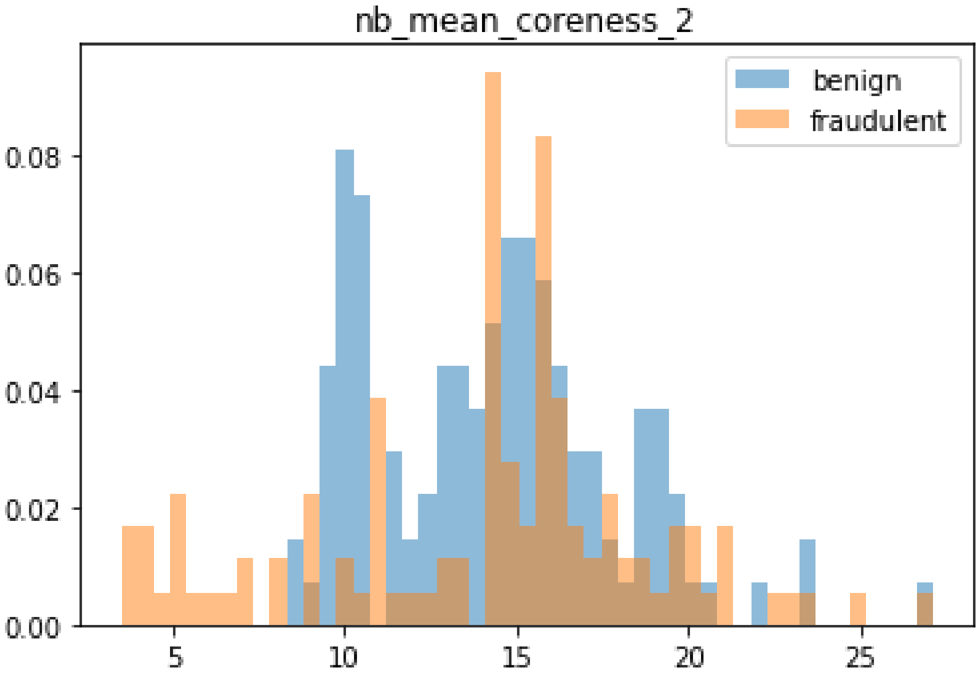
nb_mean_coreness_2
benign
fraudulent
0.08
0.06
0.04
0.02
0.00
5
10
15
20
25

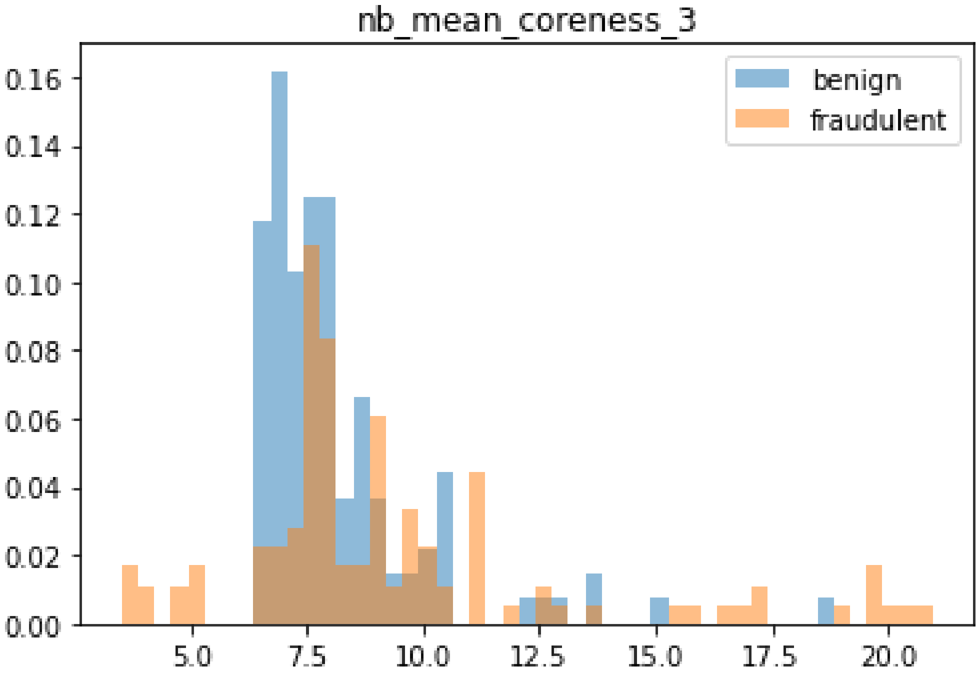
nb_mean_coreness_3
benign
fraudulent
0.16
0.14
0.12
0.10
0.08
0.06
0.04
0.02
0.00
5.0
7.5
10.0
12.5
15.0
17.5
20.0

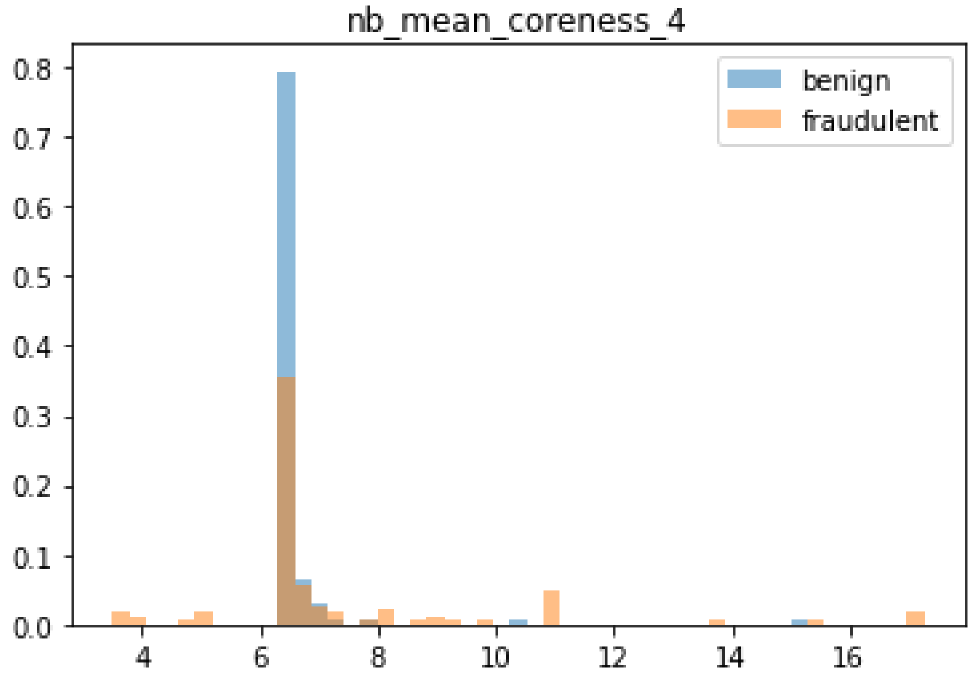
nb_mean_coreness_4
benign
fraudulent
0.8
0.7
0.6
0.5
0.4
0.3
0.2
0.1
0.0
4
6
8
10
12
14
16

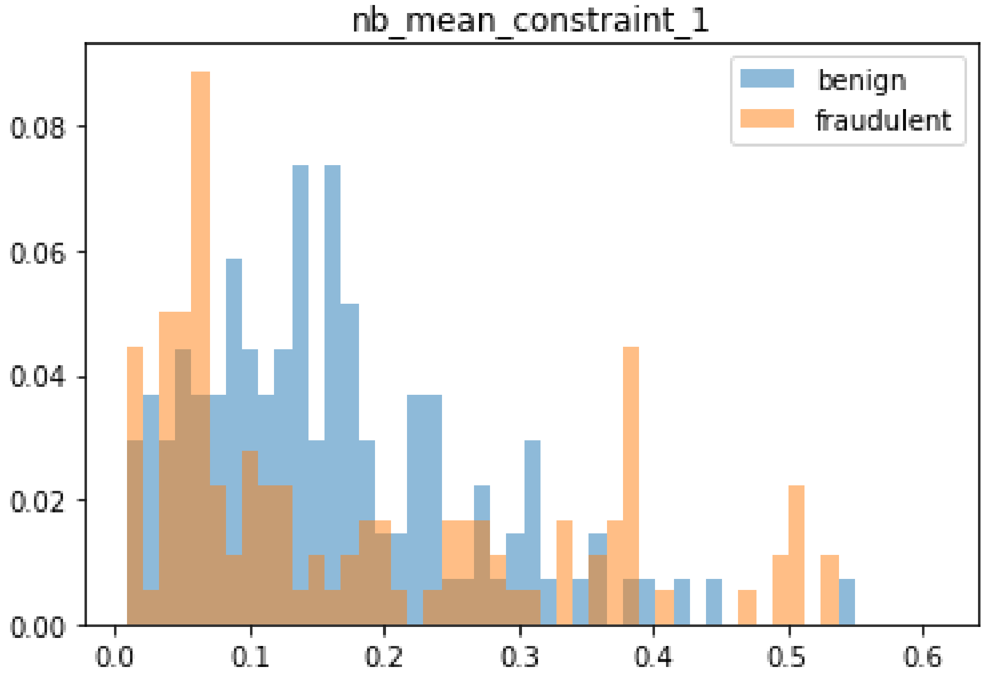
nb_mean_constraint_1
benign
fraudulent
0.08
0.06
0.04
0.02
0.00
0.0
0.1
0.2
0.3
0.4
0.5
0.6

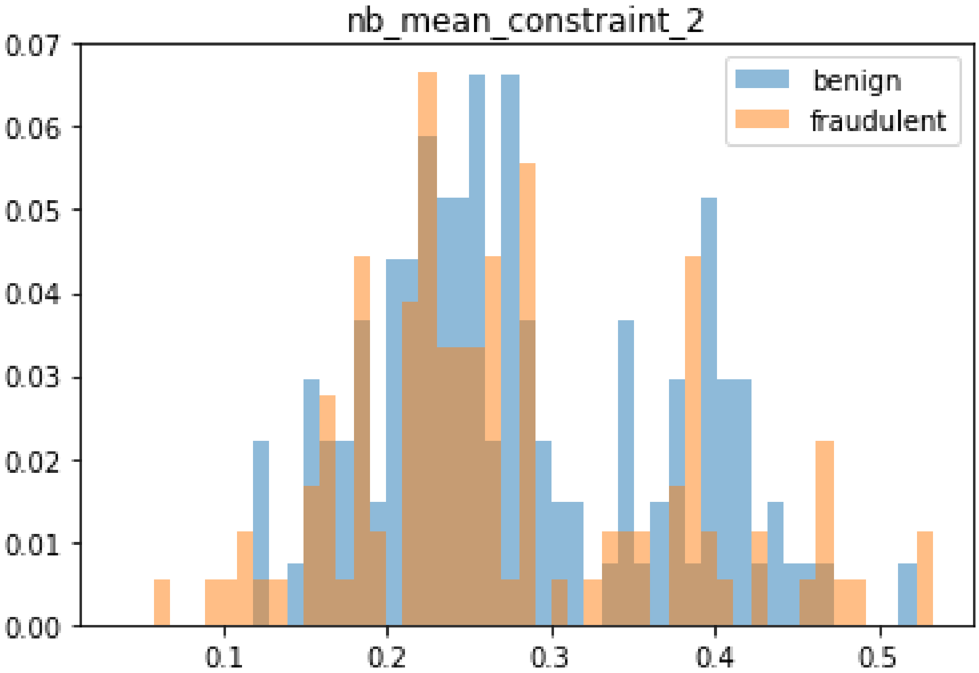
nb_mean_constraint_2
benign
fraudulent
0.07
0.06
0.05
0.04
0.03
0.02
0.01
0.00
0.1
0.2
0.3
0.4
0.5

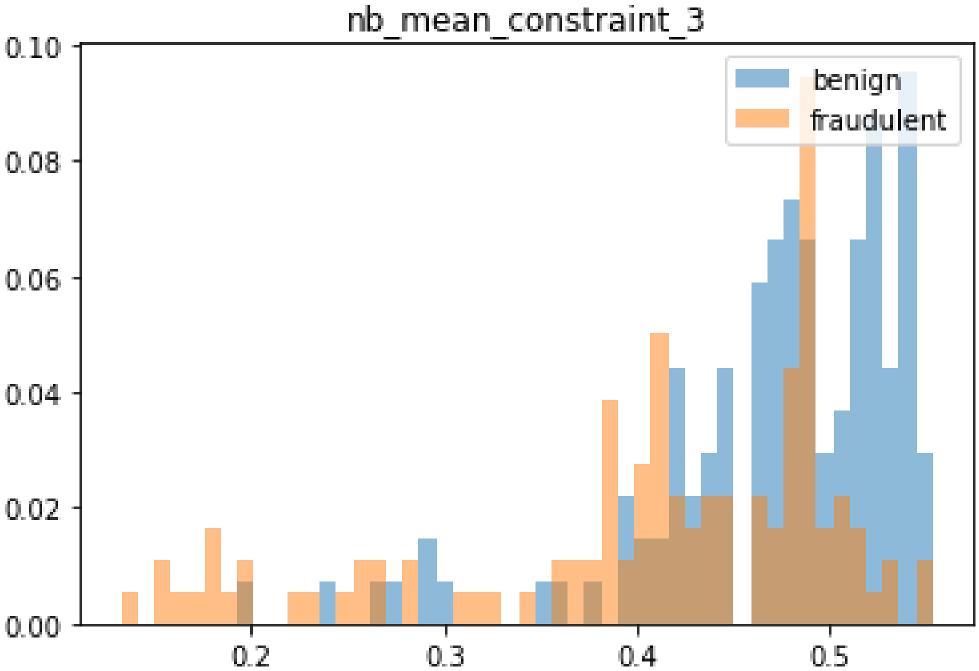
nb_mean_constraint_3
benign
fraudulent
0.10
0.08
0.06
0.04
0.02
0.00
0.2
0.3
0.4
0.5

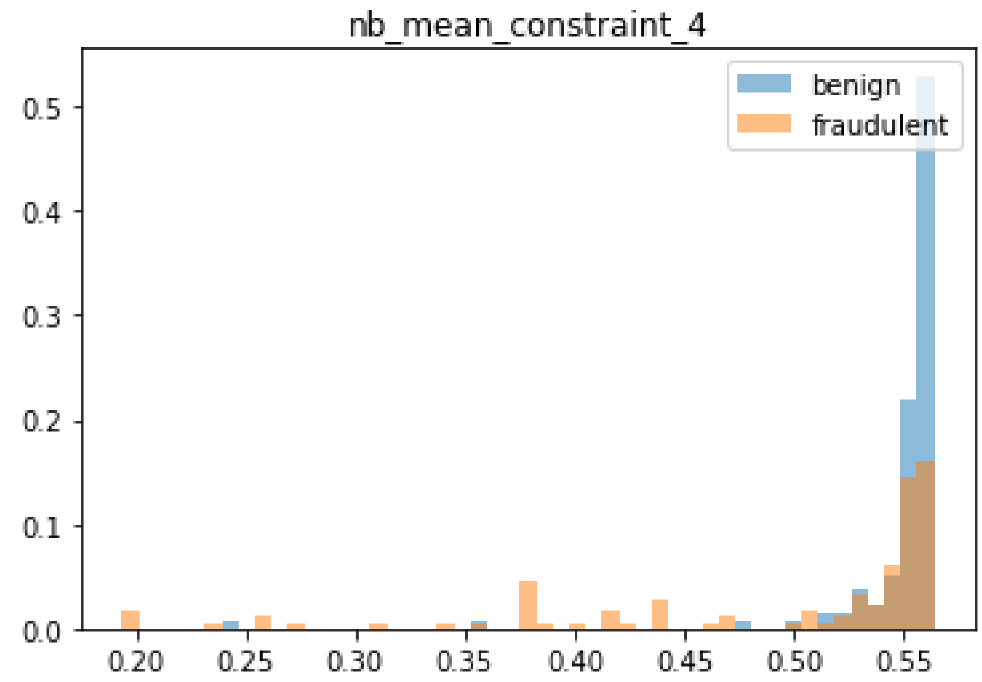

nb_mean_constraint_4
benign
fraudulent
0.0
0.1
0.2
0.3
0.4
0.5
0.20
0.25
0.30
0.35
0.40
0.45
0.50
0.55

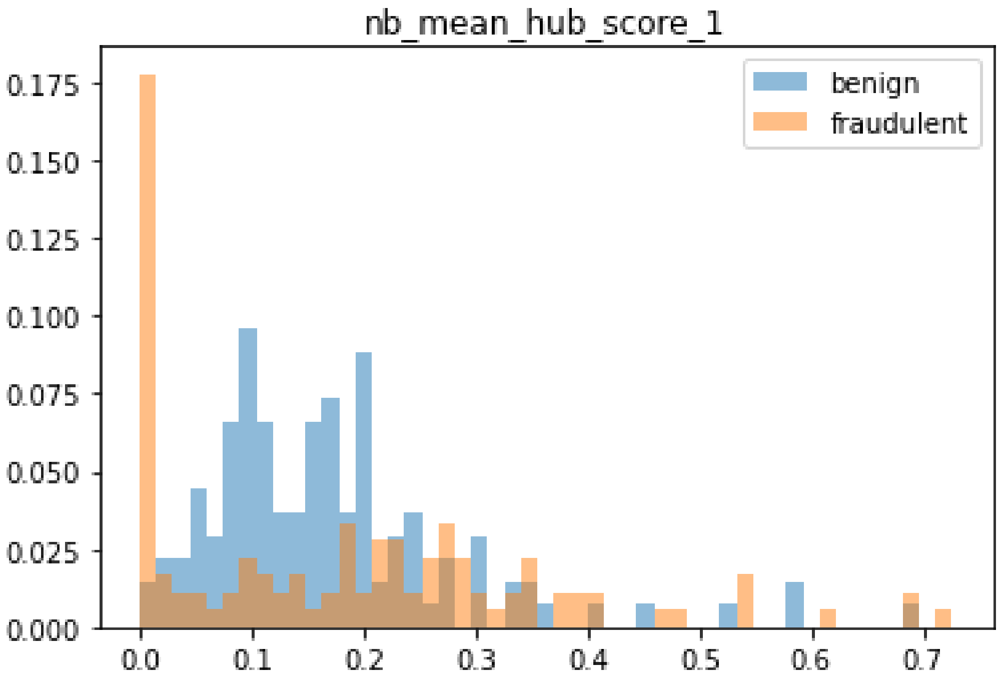

nb_mean_hub_score_1
benign
fraudulent
0.000
0.025
0.050
0.075
0.100
0.125
0.150
0.175
0.0
0.1
0.2
0.3
0.4
0.5
0.6
0.7

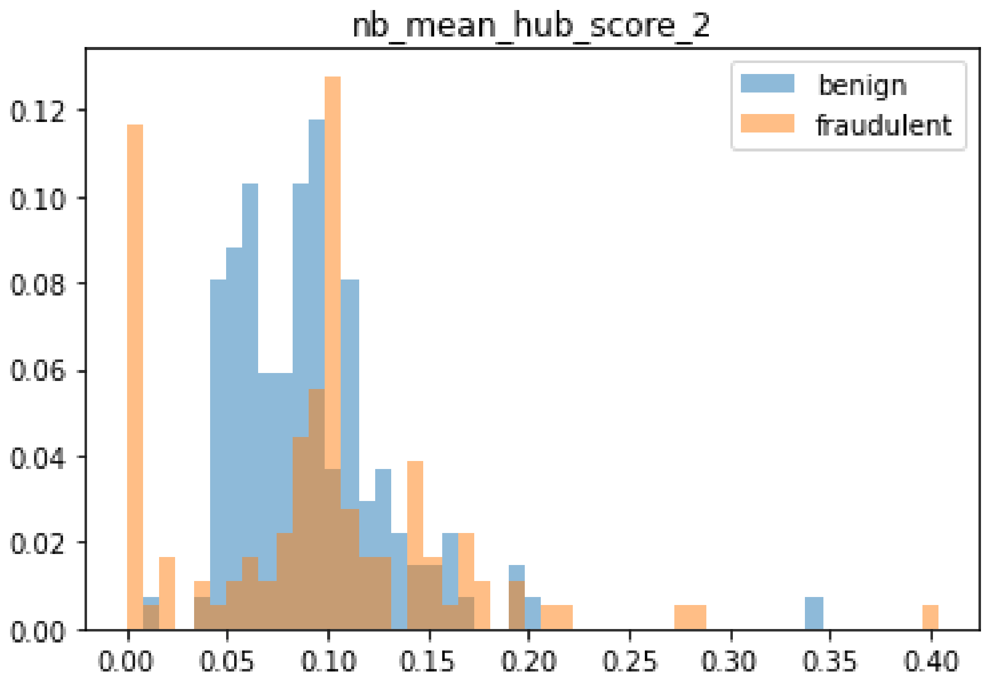
nb_mean_hub_score_2
benign
fraudulent
0.00
0.02
0.04
0.06
0.08
0.10
0.12
0.00
0.05
0.10
0.15
0.20
0.25
0.30
0.35
0.40

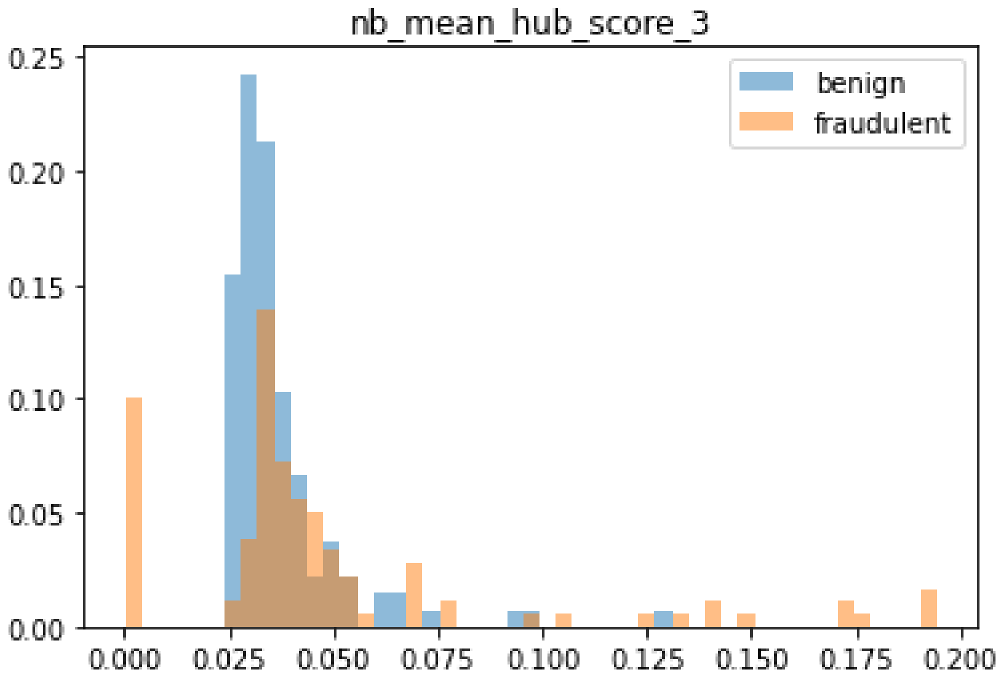
nb_mean_hub_score_3
benign
fraudulent
0.00
0.05
0.10
0.15
0.20
0.25
0.000
0.025
0.050
0.075
0.100
0.125
0.150
0.175
0.200

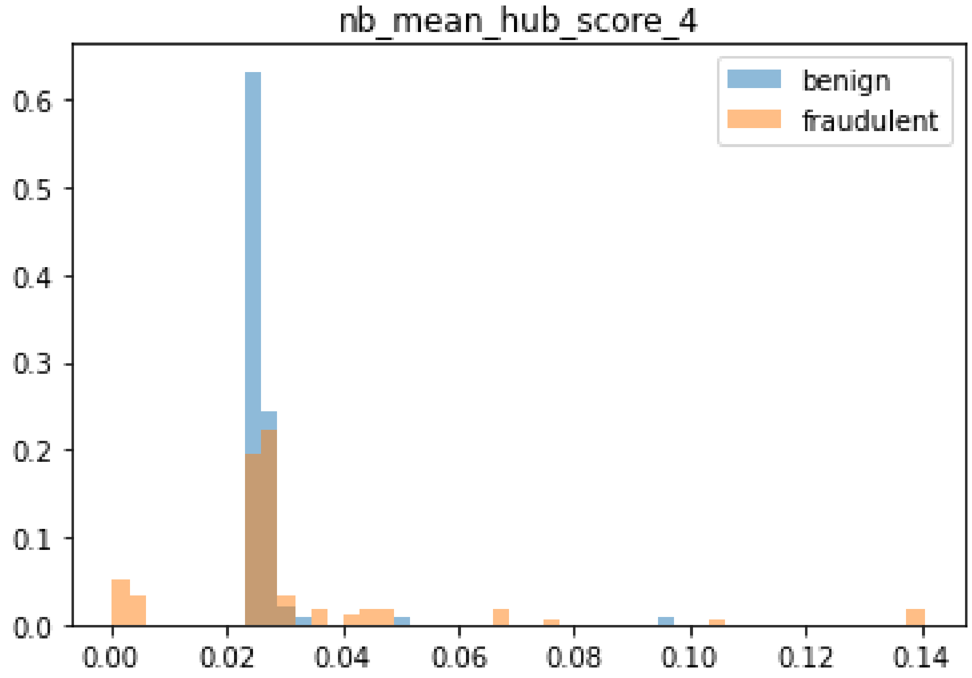
nb_mean_hub_score_4
benign
fraudulent
0.6
0.5
0.4
0.3
0.2
0.1
0.0
0.00
0.02
0.04
0.06
0.08
0.10
0.12
0.14

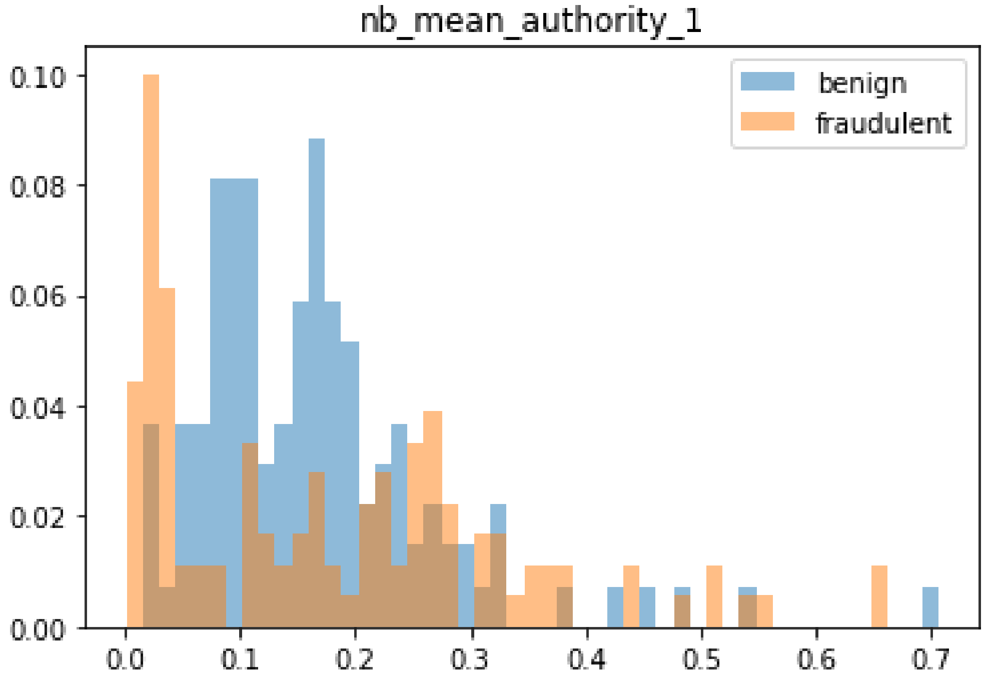
nb_mean_authority_1
benign
fraudulent
0.10
0.08
0.06
0.04
0.02
0.00
0.0
0.1
0.2
0.3
0.4
0.5
0.6
0.7

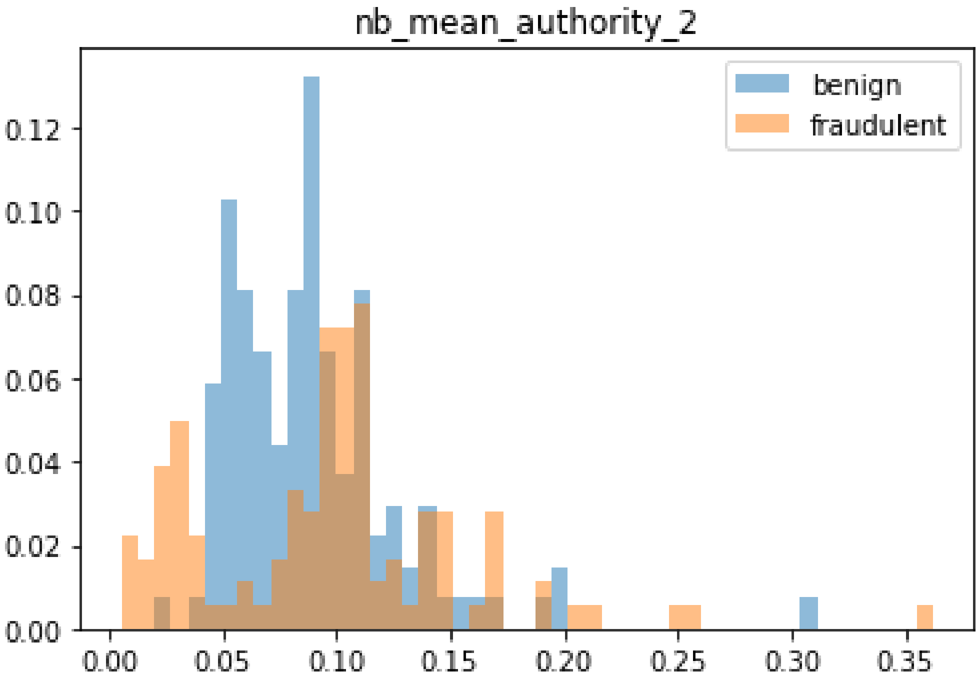
nb_mean_authority_2
benign
fraudulent
0.12
0.10
0.08
0.06
0.04
0.02
0.00
0.00
0.05
0.10
0.15
0.20
0.25
0.30
0.35

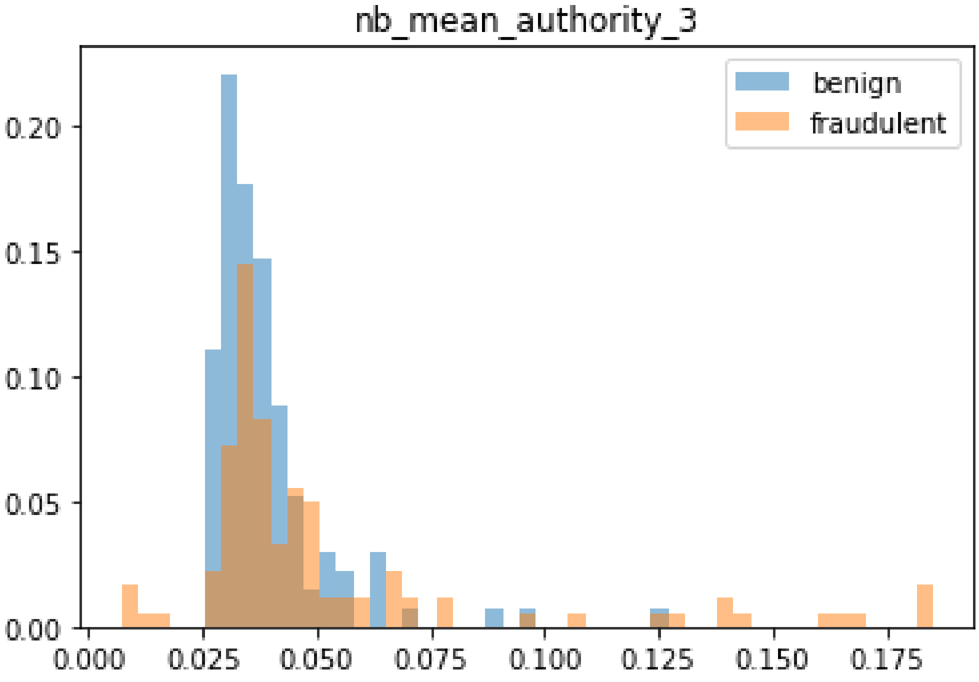
nb_mean_authority_3
benign
fraudulent
0.20
0.15
0.10
0.05
0.00
0.000
0.025
0.050
0.075
0.100
0.125
0.150
0.175

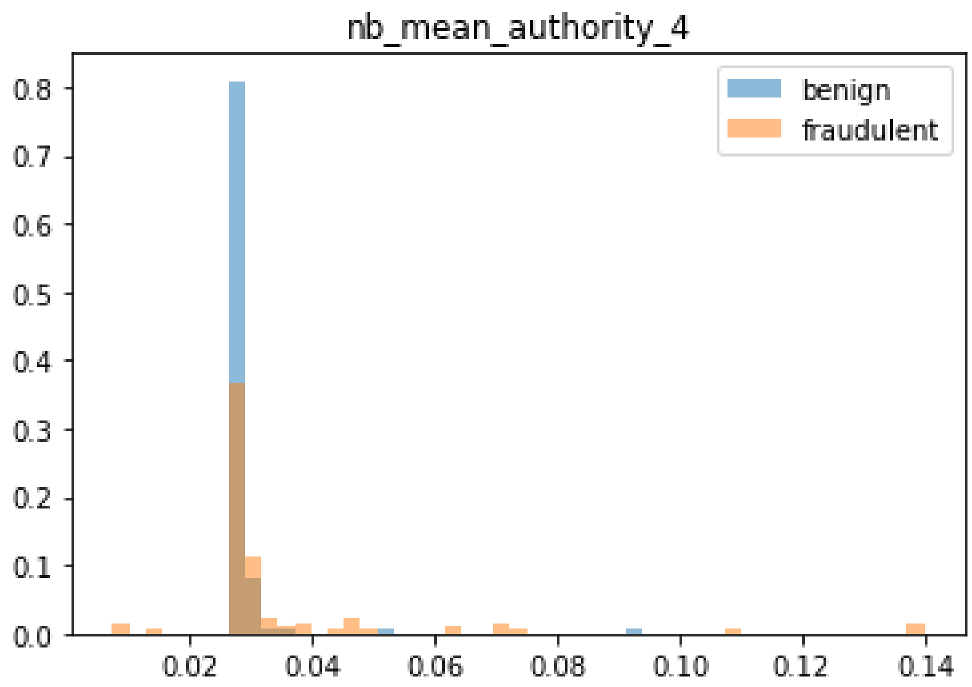
nb_mean_authority_4
benign
fraudulent
0.8
0.7
0.6
0.5
0.4
0.3
0.2
0.1
0.0
0.02
0.04
0.06
0.08
0.10
0.12
0.14

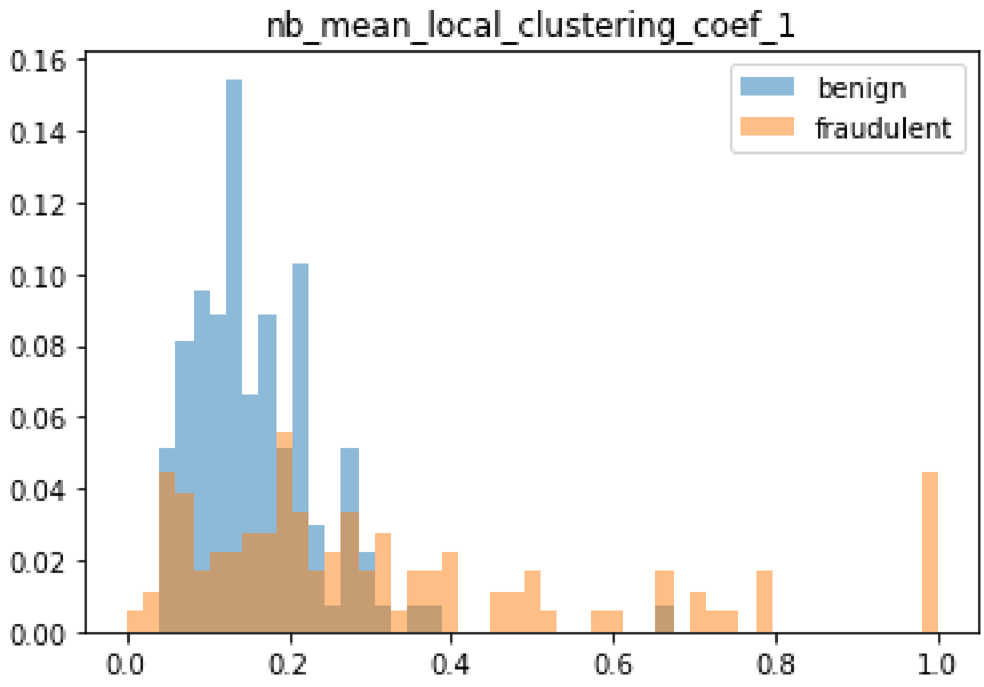
nb_mean_local_clustering_coef_1
benign
fraudulent
0.16
0.14
0.12
0.10
0.08
0.06
0.04
0.02
0.00
0.0
0.2
0.4
0.6
0.8
1.0

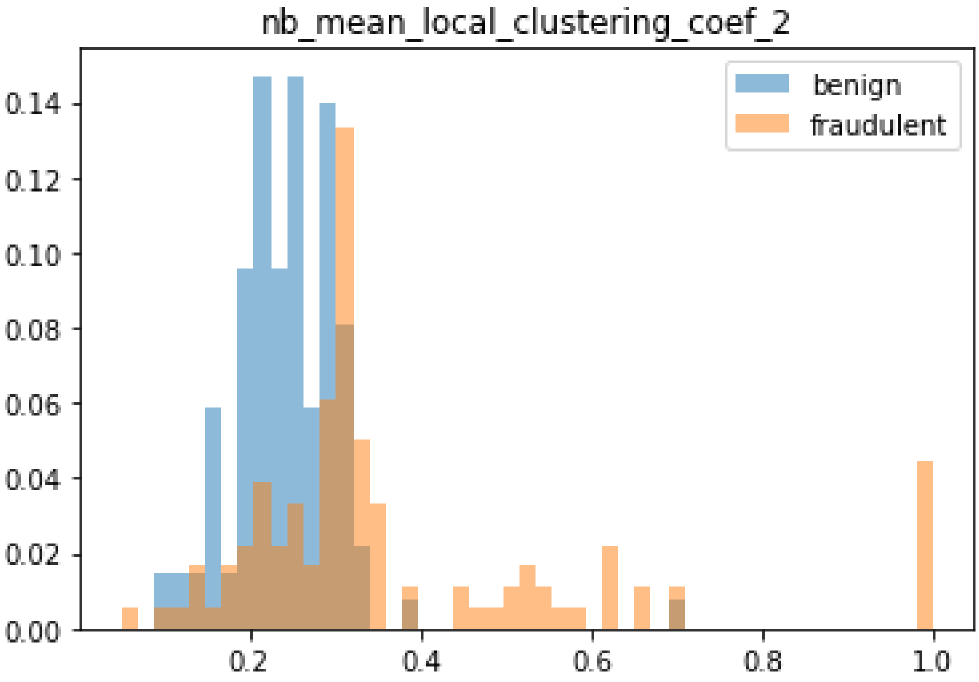
nb_mean_local_clustering_coef_2
benign
fraudulent
0.00
0.02
0.04
0.06
0.08
0.10
0.12
0.14
0.2
0.4
0.6
0.8
1.0

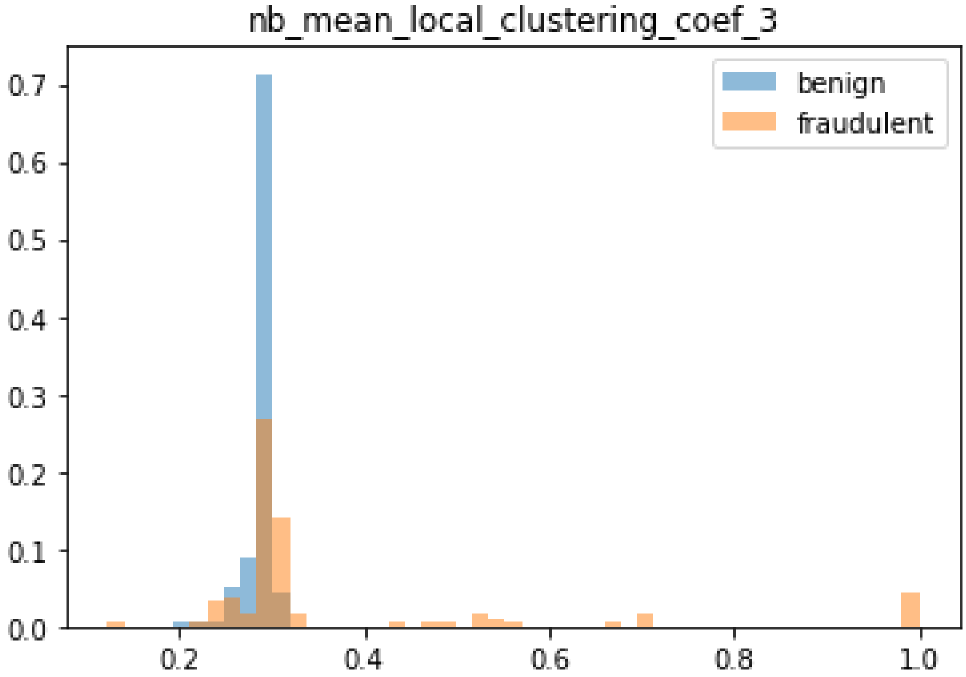
nb_mean_local_clustering_coef_3
benign
fraudulent
0.0
0.1
0.2
0.3
0.4
0.5
0.6
0.7
0.2
0.4
0.6
0.8
1.0

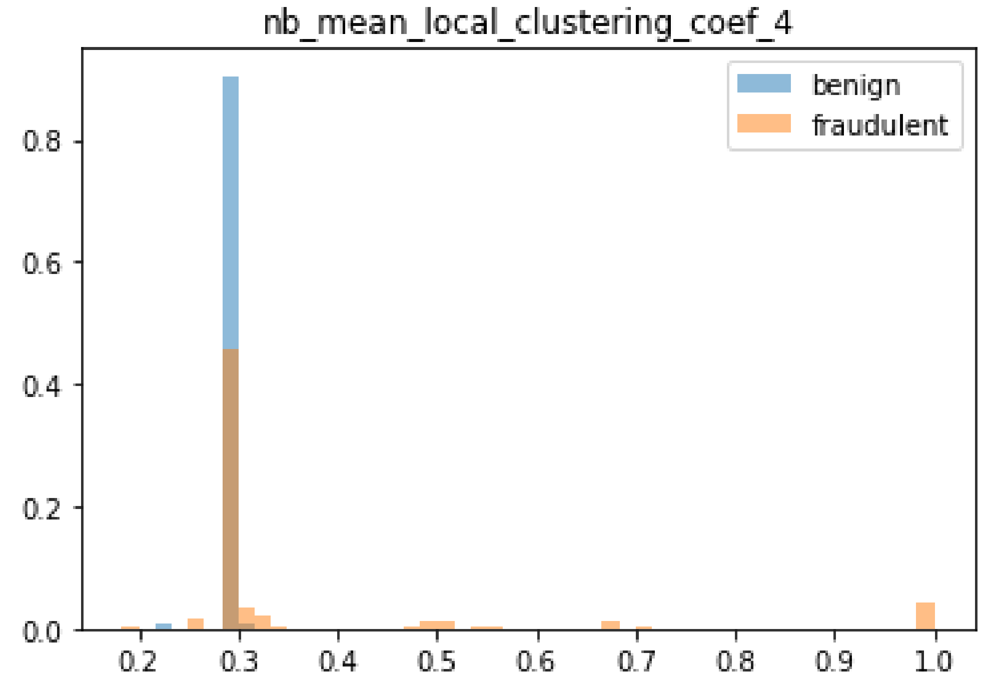
nb_mean_local_clustering_coef_4
benign
fraudulent
0.8
0.6
0.4
0.2
0.0
0.2
0.3
0.4
0.5
0.6
0.7
0.8
0.9
1.0

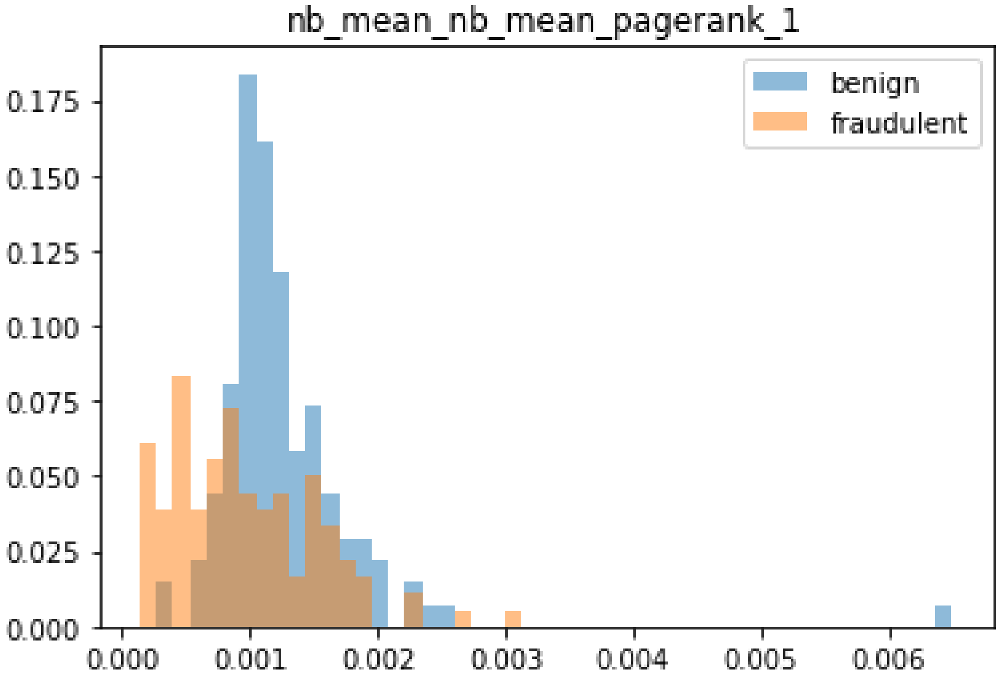
nb_mean_nb_mean_pagerank_1
benign
fraudulent
0.175
0.150
0.125
0.100
0.075
0.050
0.025
0.000
0.000
0.001
0.002
0.003
0.004
0.005
0.006

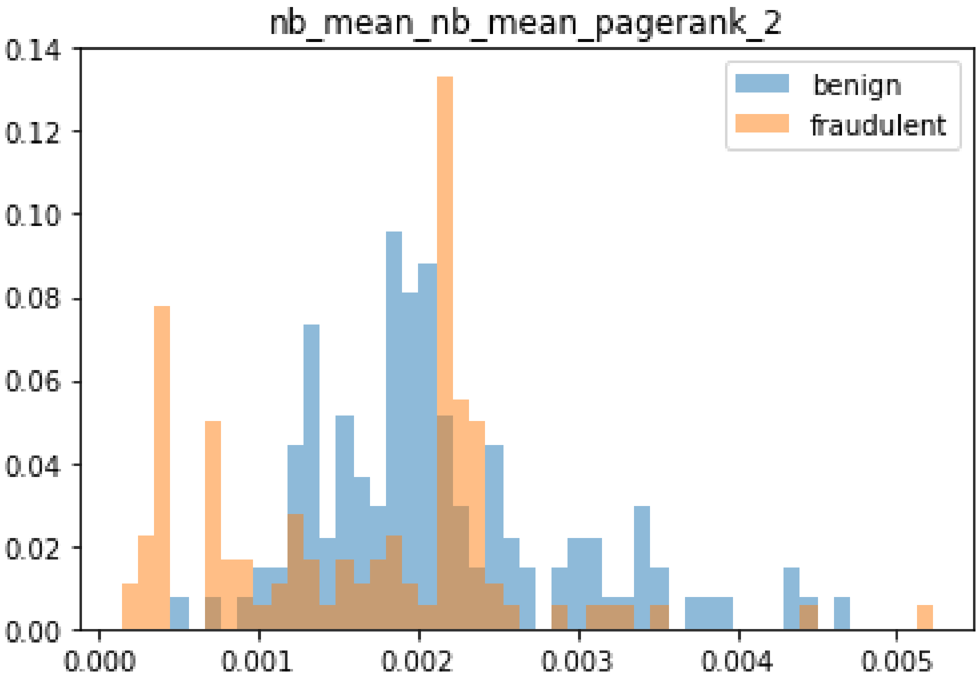
nb_mean_nb_mean_pagerank_2
benign
fraudulent
0.00
0.02
0.04
0.06
0.08
0.10
0.12
0.14
0.000
0.001
0.002
0.003
0.004
0.005

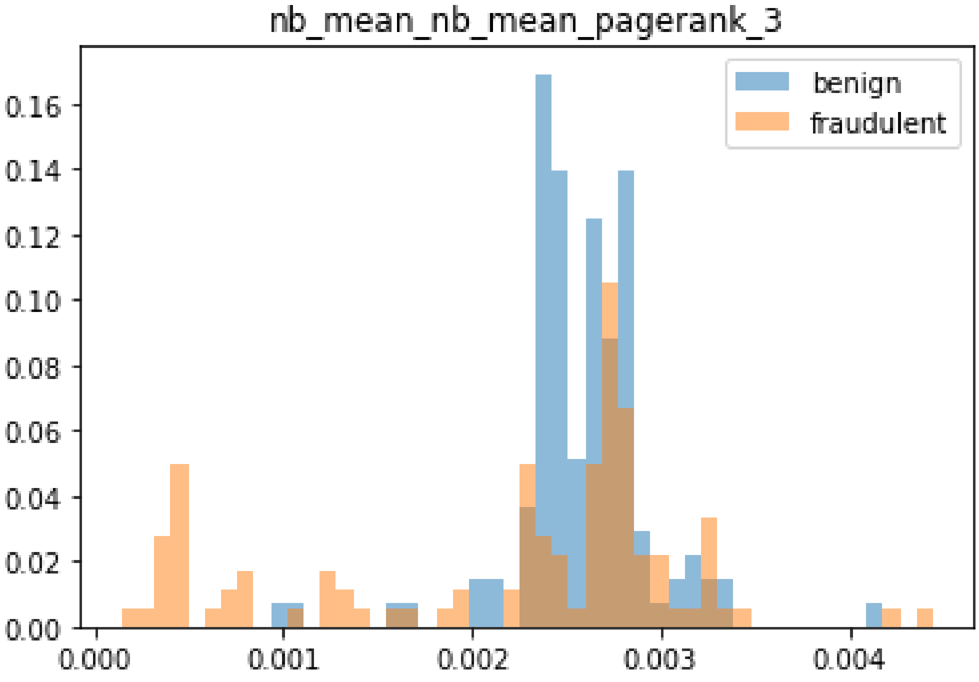
nb_mean_nb_mean_pagerank_3
benign
fraudulent
0.00
0.02
0.04
0.06
0.08
0.10
0.12
0.14
0.16
0.000
0.001
0.002
0.003
0.004

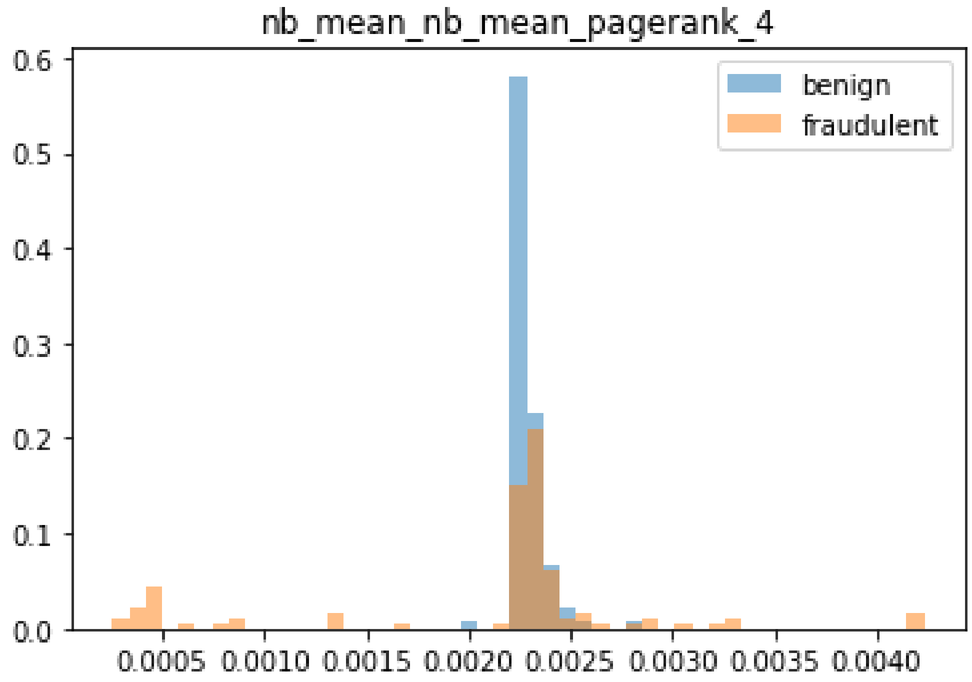
nb_mean_nb_mean_pagerank_4
benign
fraudulent
0.6
0.5
0.4
0.3
0.2
0.1
0.0
0.0005 0.0010 0.0015 0.0020 0.0025 0.0030 0.0035 0.0040

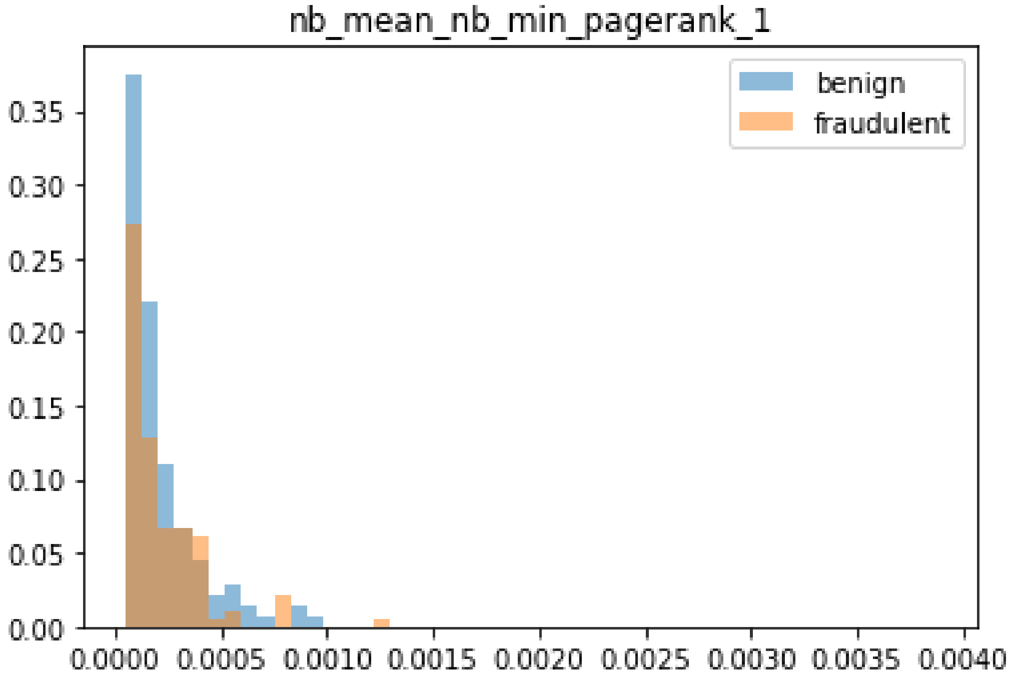
nb_mean_nb_min_pagerank_1
benign
fraudulent
0.35
0.30
0.25
0.20
0.15
0.10
0.05
0.00
0.0000 0.0005 0.0010 0.0015 0.0020 0.0025 0.0030 0.0035 0.0040

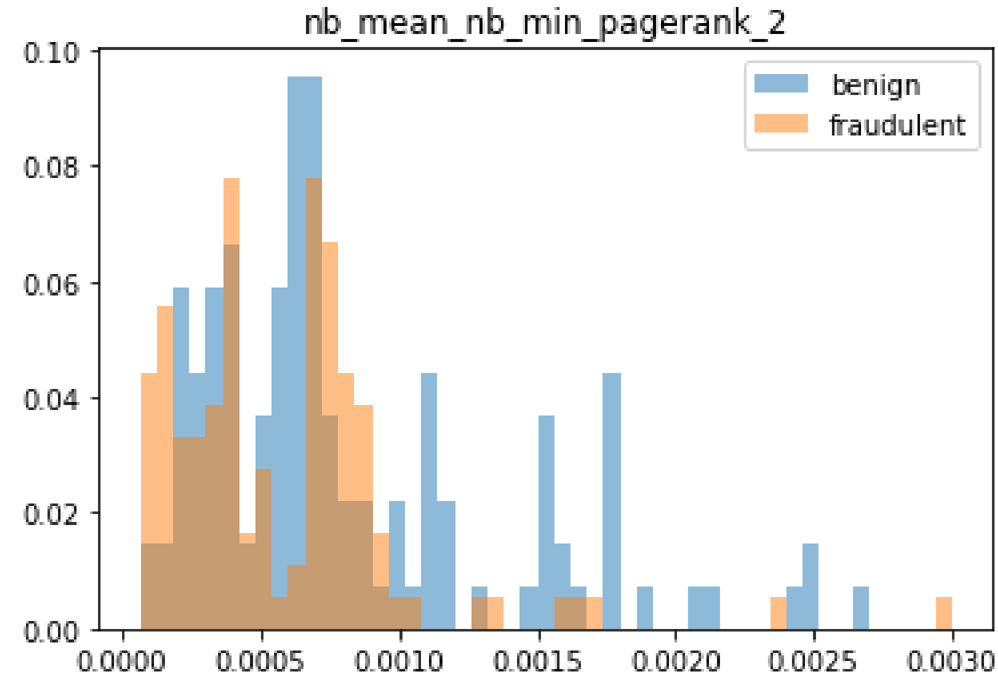
nb_mean_nb_min_pagerank_2
benign
fraudulent
0.10
0.08
0.06
0.04
0.02
0.00
0.0000
0.0005
0.0010
0.0015
0.0020
0.0025
0.0030

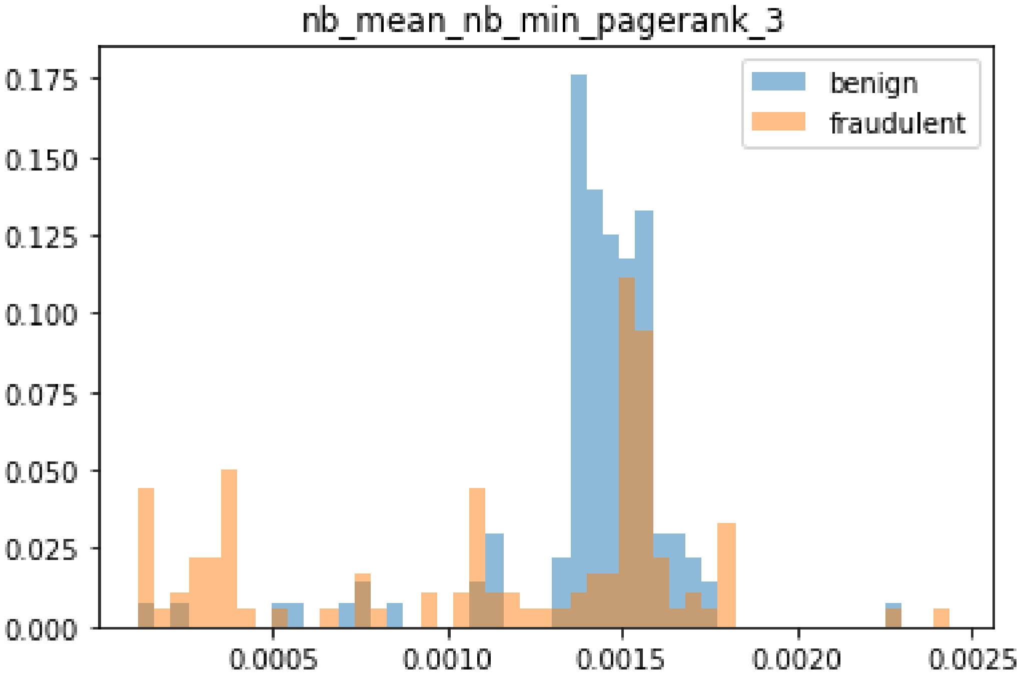
nb_mean_nb_min_pagerank_3
benign
fraudulent
0.175
0.150
0.125
0.100
0.075
0.050
0.025
0.000
0.0005
0.0010
0.0015
0.0020
0.0025

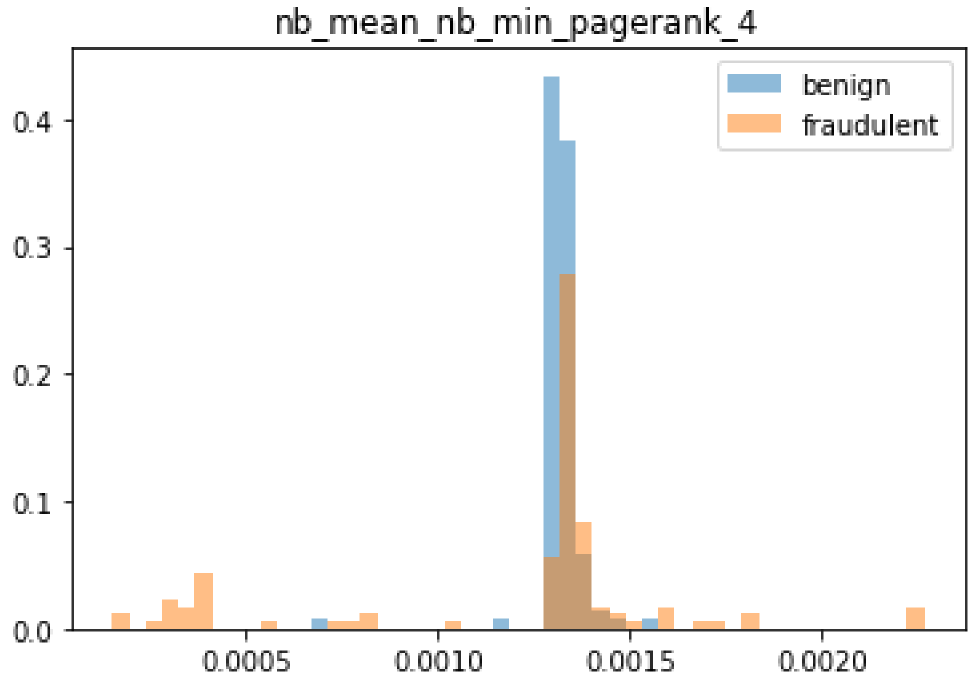
nb_mean_nb_min_pagerank_4
benign
fraudulent
0.4
0.3
0.2
0.1
0.0
0.0005
0.0010
0.0015
0.0020

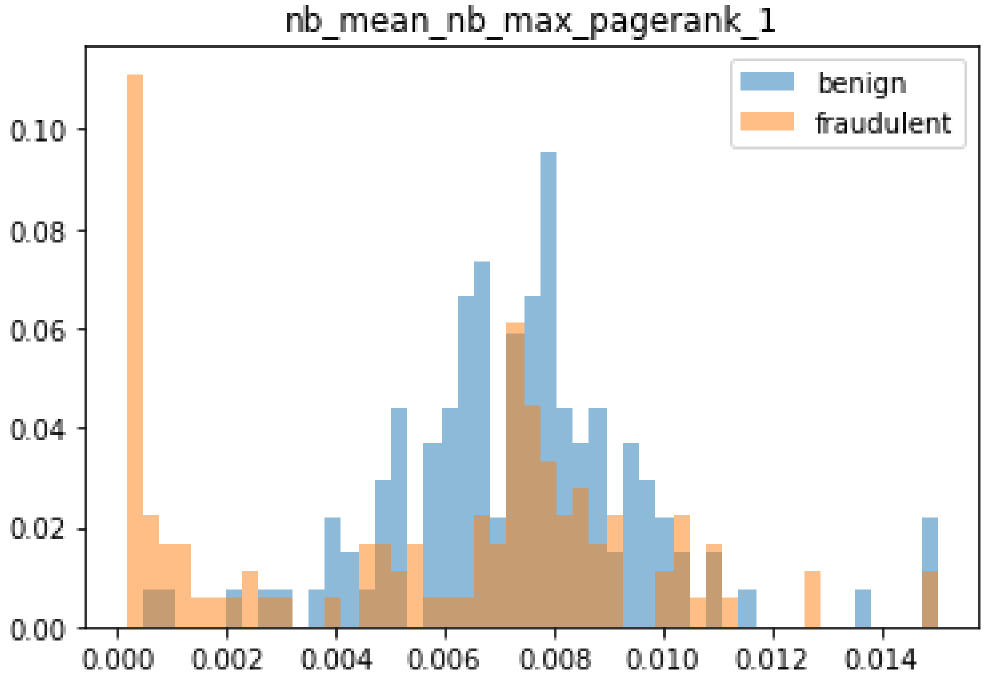
nb_mean_nb_max_pagerank_1
benign
fraudulent
0.10
0.08
0.06
0.04
0.02
0.00
0.000
0.002
0.004
0.006
0.008
0.010
0.012
0.014

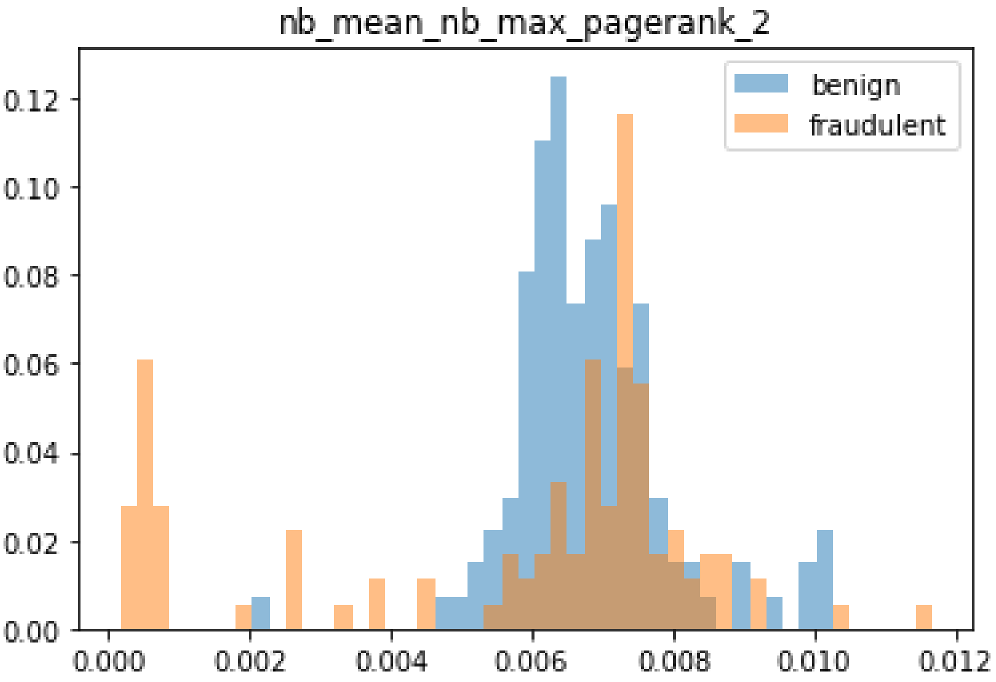
nb_mean_nb_max_pagerank_2
benign
fraudulent
0.12
0.10
0.08
0.06
0.04
0.02
0.00
0.000
0.002
0.004
0.006
0.008
0.010
0.012

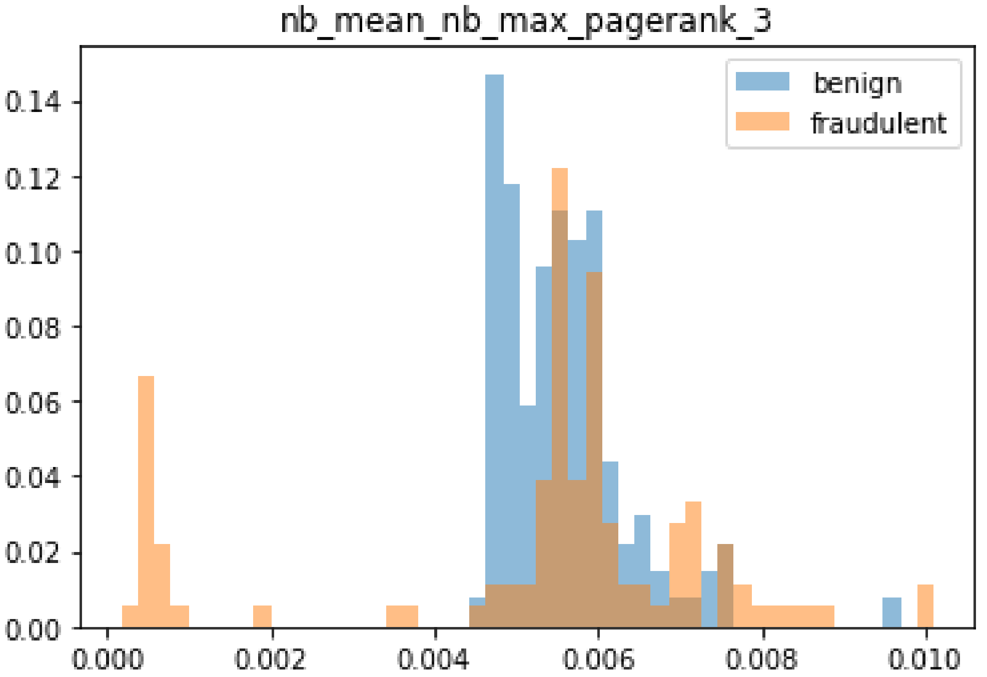
nb_mean_nb_max_pagerank_3
benign
fraudulent
0.14
0.12
0.10
0.08
0.06
0.04
0.02
0.00
0.000
0.002
0.004
0.006
0.008
0.010

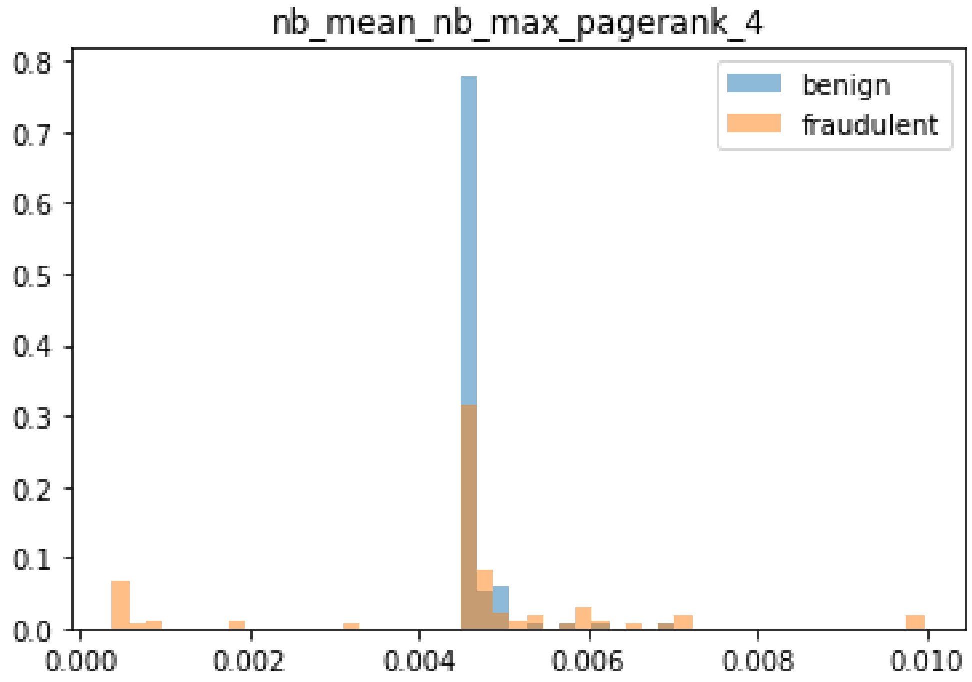
nb_mean_nb_max_pagerank_4
benign
fraudulent
0.0
0.1
0.2
0.3
0.4
0.5
0.6
0.7
0.8
0.000
0.002
0.004
0.006
0.008
0.010

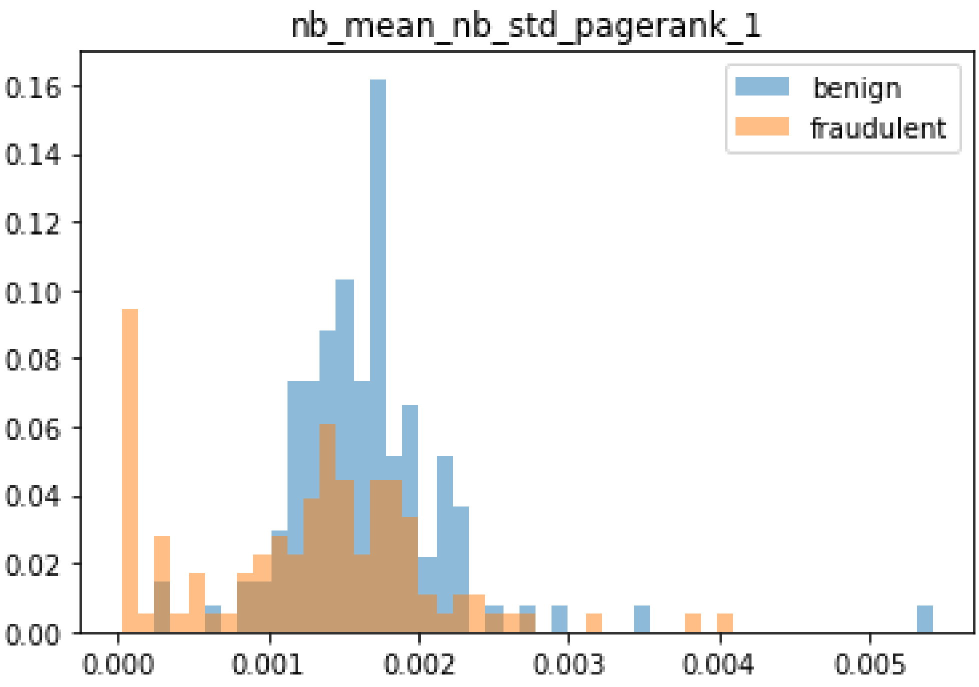
nb_mean_nb_std_pagerank_1
benign
fraudulent
0.00
0.02
0.04
0.06
0.08
0.10
0.12
0.14
0.16
0.000
0.001
0.002
0.003
0.004
0.005

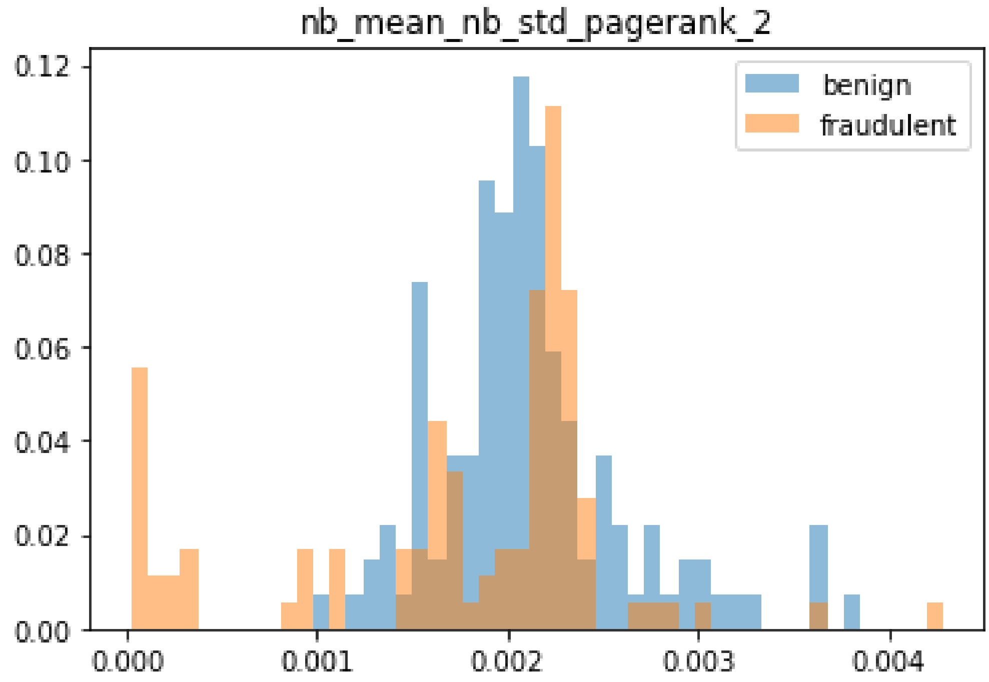
nb_mean_nb_std_pagerank_2
benign
fraudulent
0.12
0.10
0.08
0.06
0.04
0.02
0.00
0.000
0.001
0.002
0.003
0.004

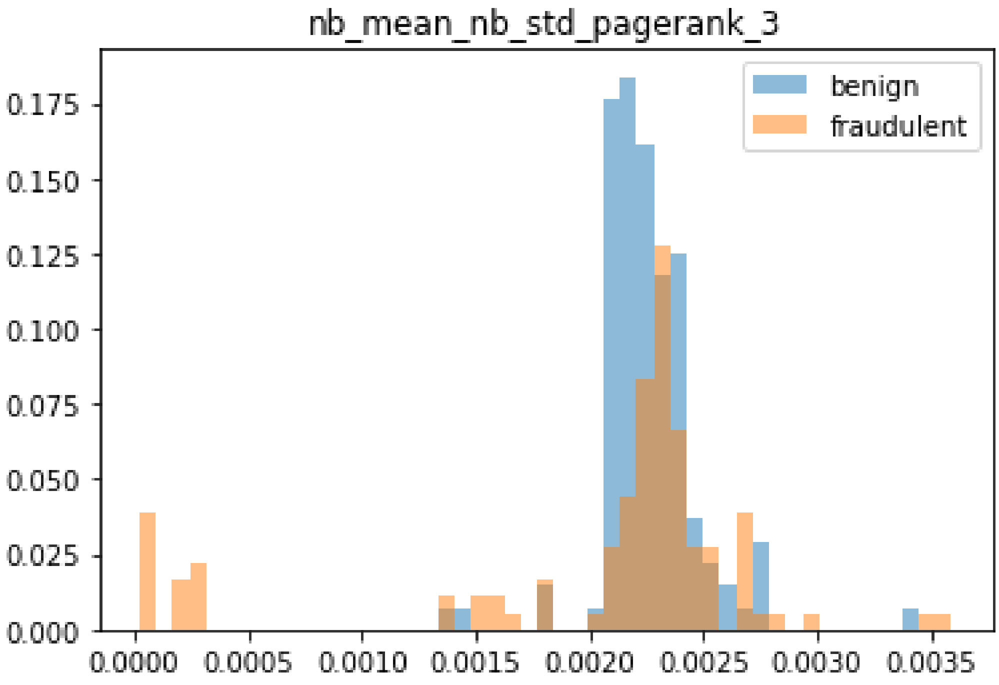
nb_mean_nb_std_pagerank_3
benign
fraudulent
0.175
0.150
0.125
0.100
0.075
0.050
0.025
0.000
0.0000
0.0005
0.0010
0.0015
0.0020
0.0025
0.0030
0.0035

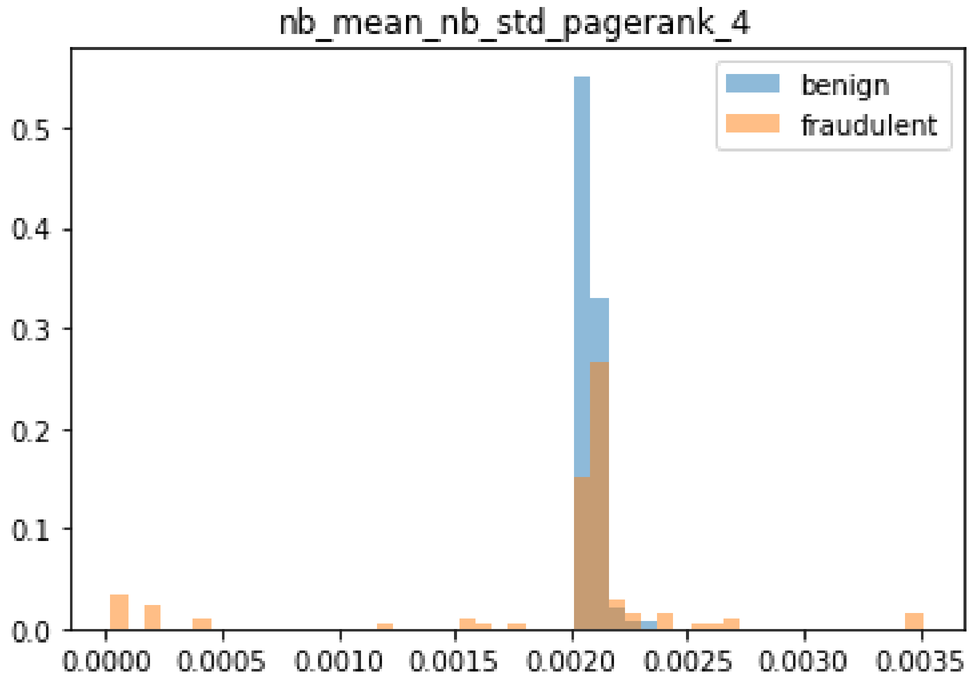
nb_mean_nb_std_pagerank_4
benign
fraudulent
0.0
0.1
0.2
0.3
0.4
0.5
0.0000
0.0005
0.0010
0.0015
0.0020
0.0025
0.0030
0.0035

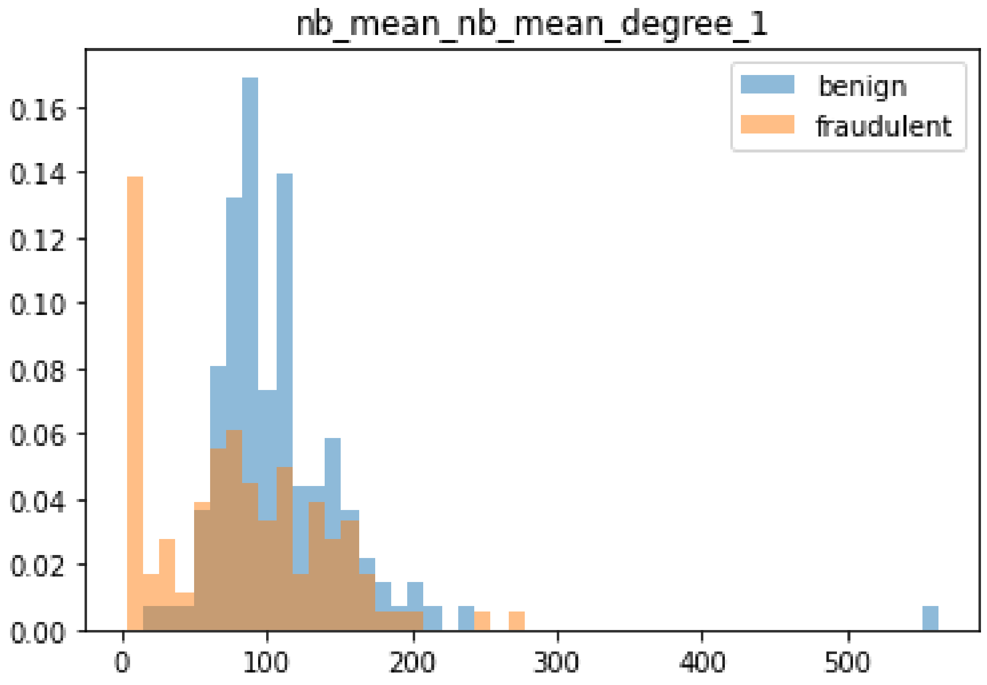
nb_mean_nb_mean_degree_1
benign
fraudulent
0.00
0.02
0.04
0.06
0.08
0.10
0.12
0.14
0.16
0
100
200
300
400
500

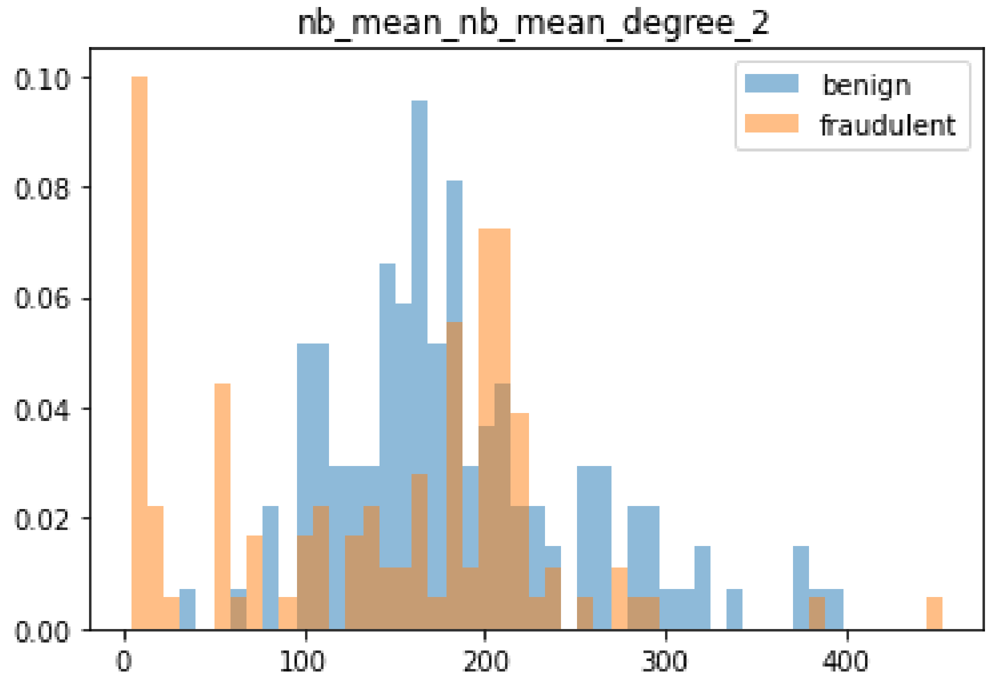
nb_mean_nb_mean_degree_2
benign
fraudulent
0.00
0.02
0.04
0.06
0.08
0.10
0
100
200
300
400

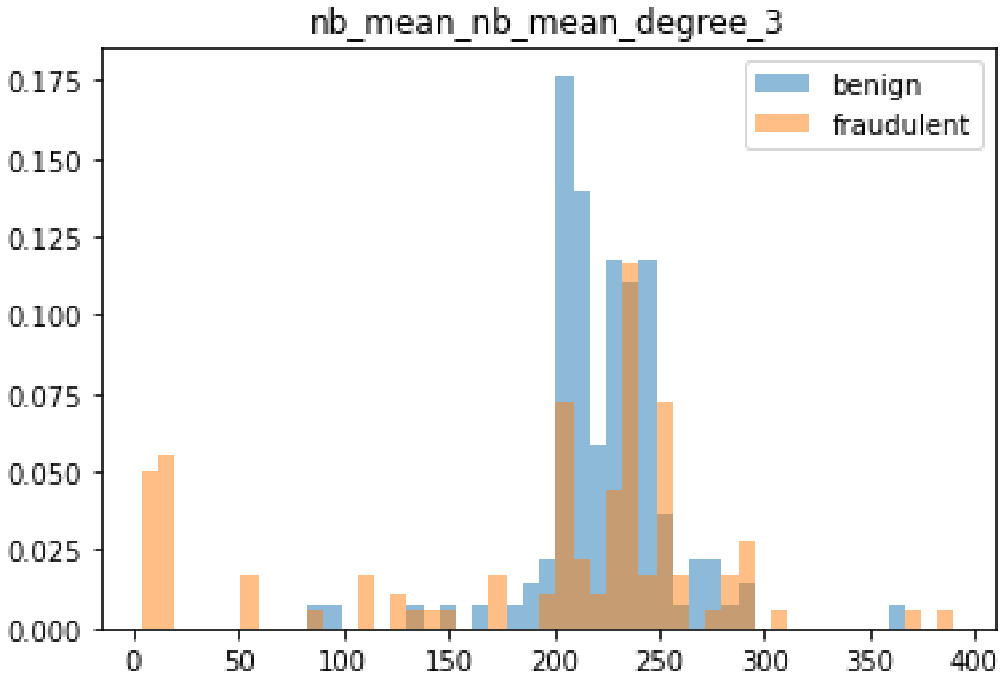
nb_mean_nb_mean_degree_3
benign
fraudulent
0.000
0.025
0.050
0.075
0.100
0.125
0.150
0.175
0
50
100
150
200
250
300
350
400

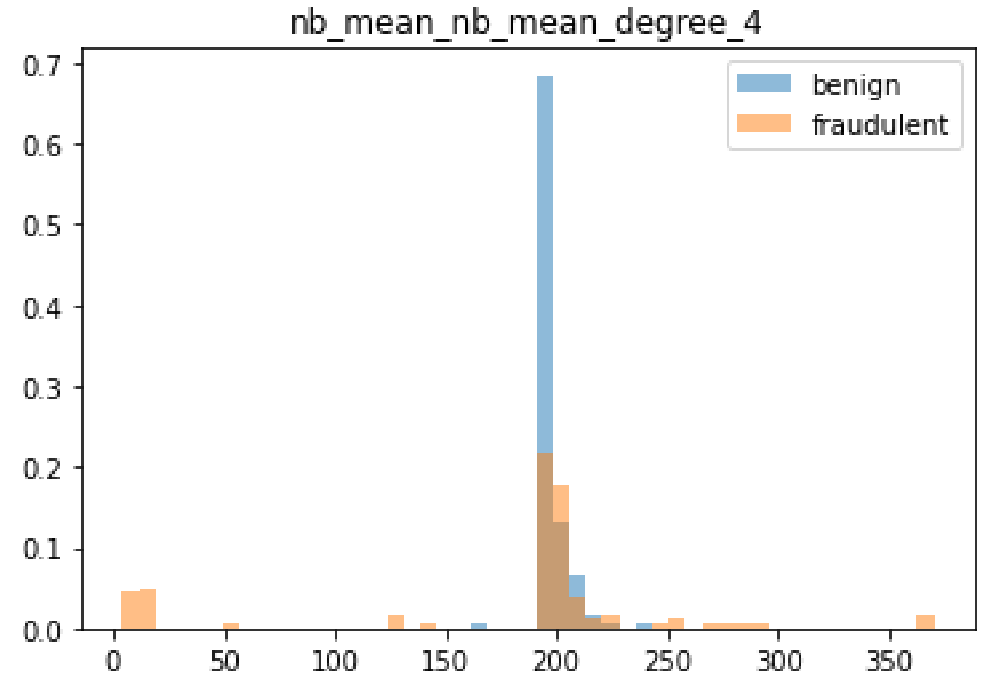
nb_mean_nb_mean_degree_4
benign
fraudulent
0.7
0.6
0.5
0.4
0.3
0.2
0.1
0.0
0
50
100
150
200
250
300
350

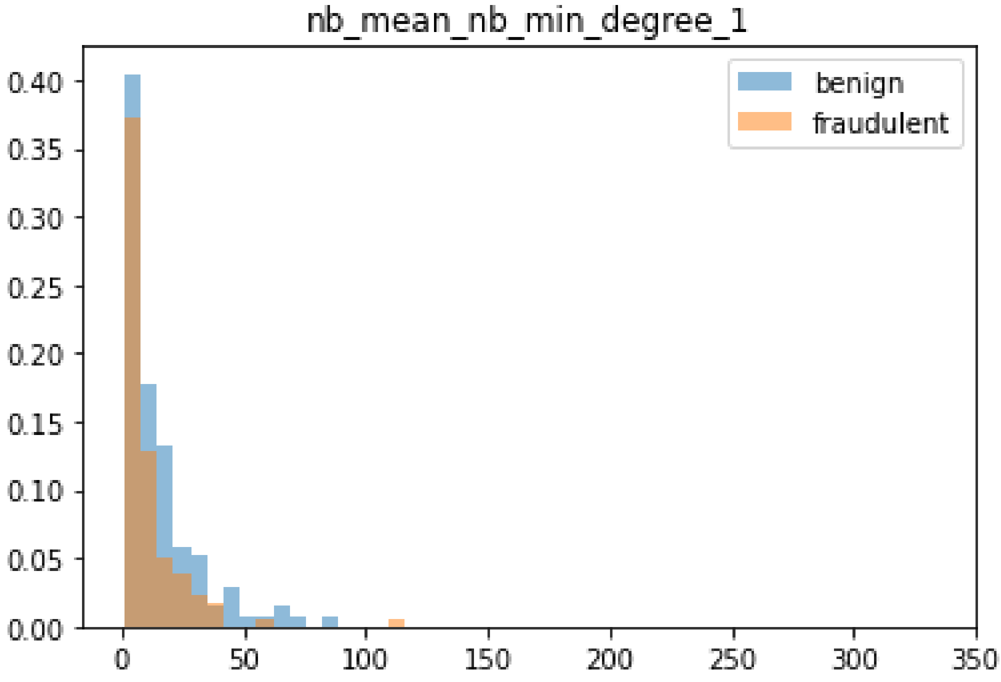
nb_mean_nb_min_degree_1
benign
fraudulent
0.40
0.35
0.30
0.25
0.20
0.15
0.10
0.05
0.00
0
50
100
150
200
250
300
350

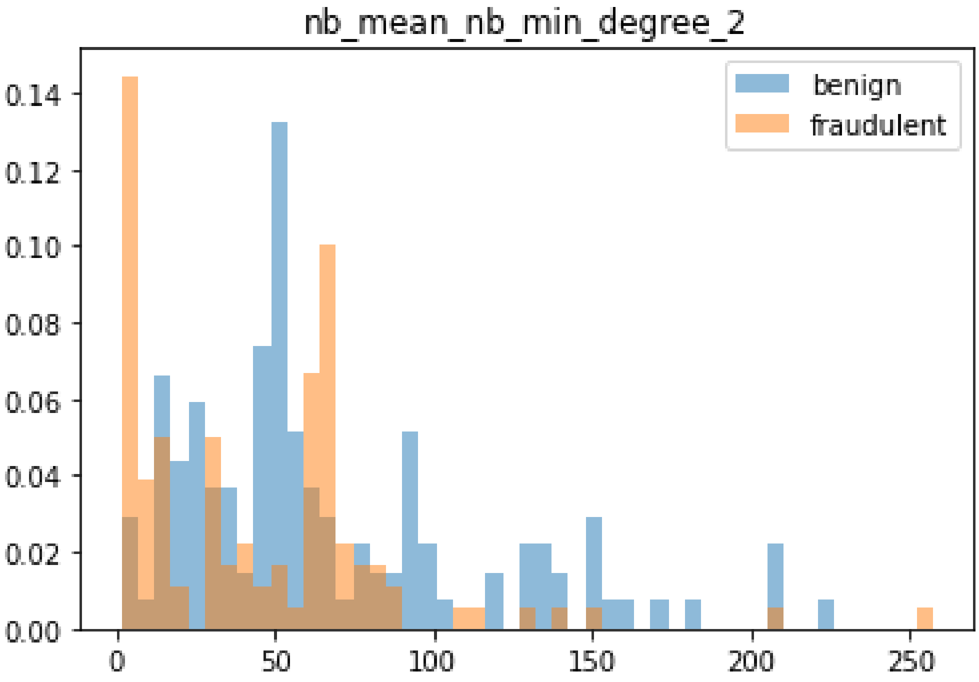
nb_mean_nb_min_degree_2
benign
fraudulent
0.00
0.02
0.04
0.06
0.08
0.10
0.12
0.14
0
50
100
150
200
250

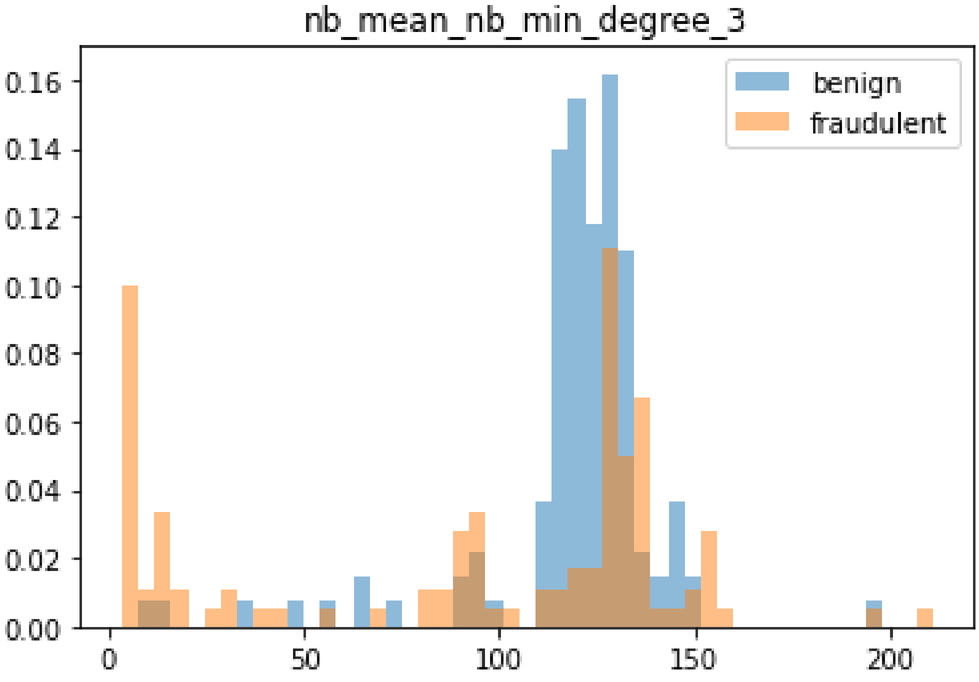
nb_mean_nb_min_degree_3
benign
fraudulent
0.00
0.02
0.04
0.06
0.08
0.10
0.12
0.14
0.16
0
50
100
150
200

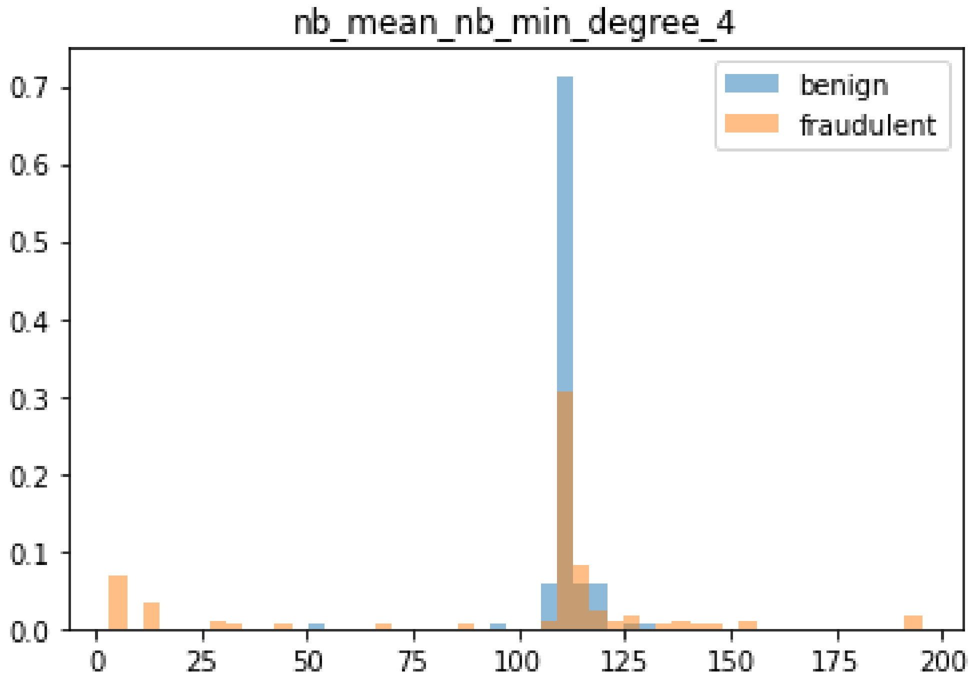
nb_mean_nb_min_degree_4
benign
fraudulent
0.7
0.6
0.5
0.4
0.3
0.2
0.1
0.0
0
25
50
75
100
125
150
175
200

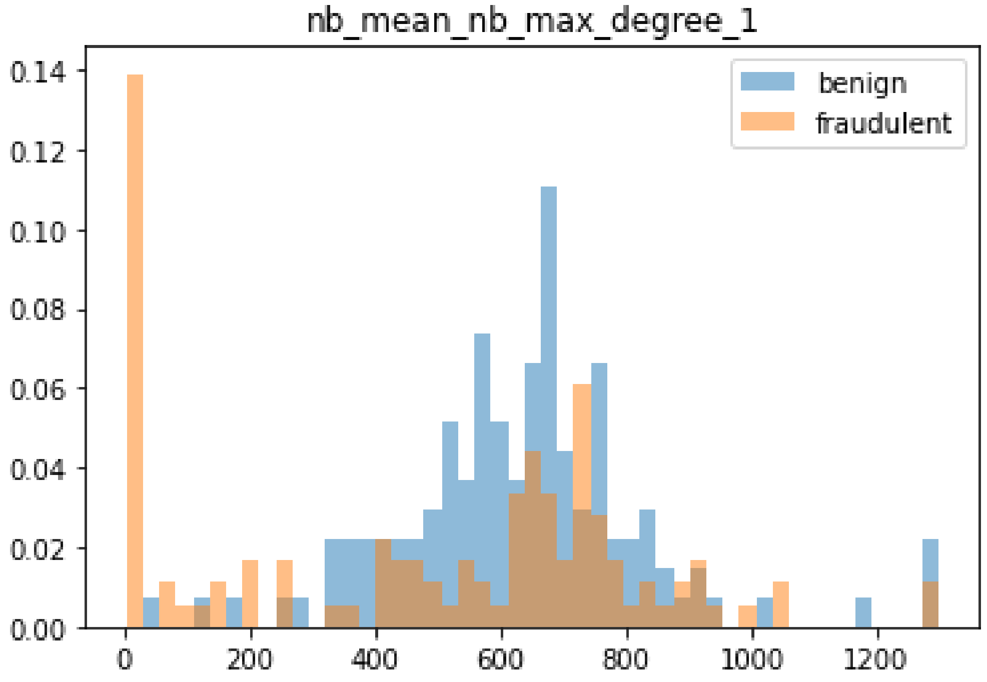
nb_mean_nb_max_degree_1
benign
fraudulent
0.14
0.12
0.10
0.08
0.06
0.04
0.02
0.00
0
200
400
600
800
1000
1200

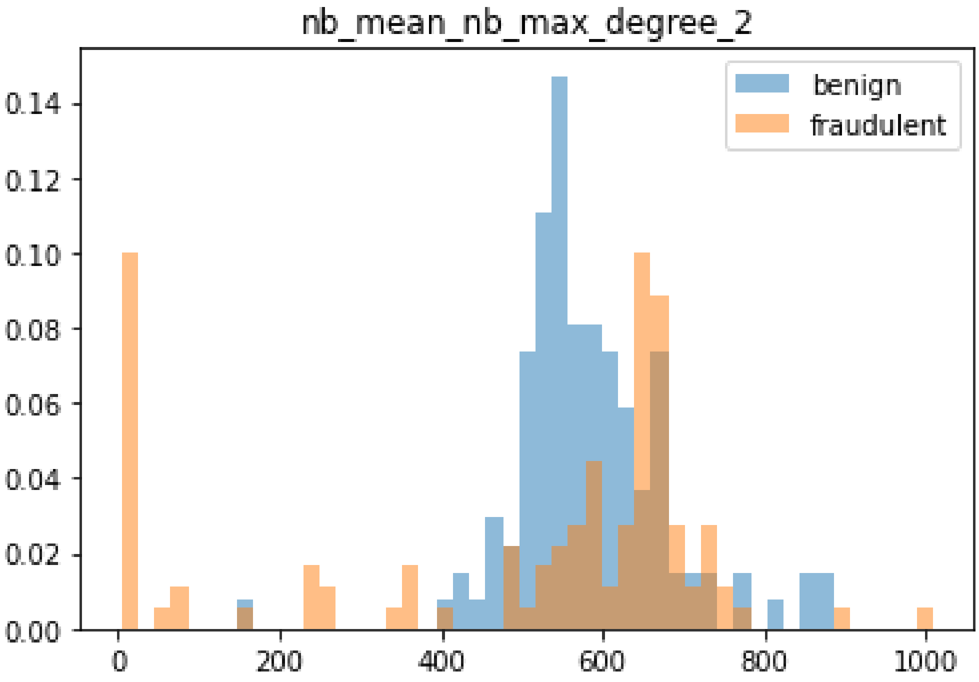
nb_mean_nb_max_degree_2
benign
fraudulent
0.00
0.02
0.04
0.06
0.08
0.10
0.12
0.14
0
200
400
600
800
1000

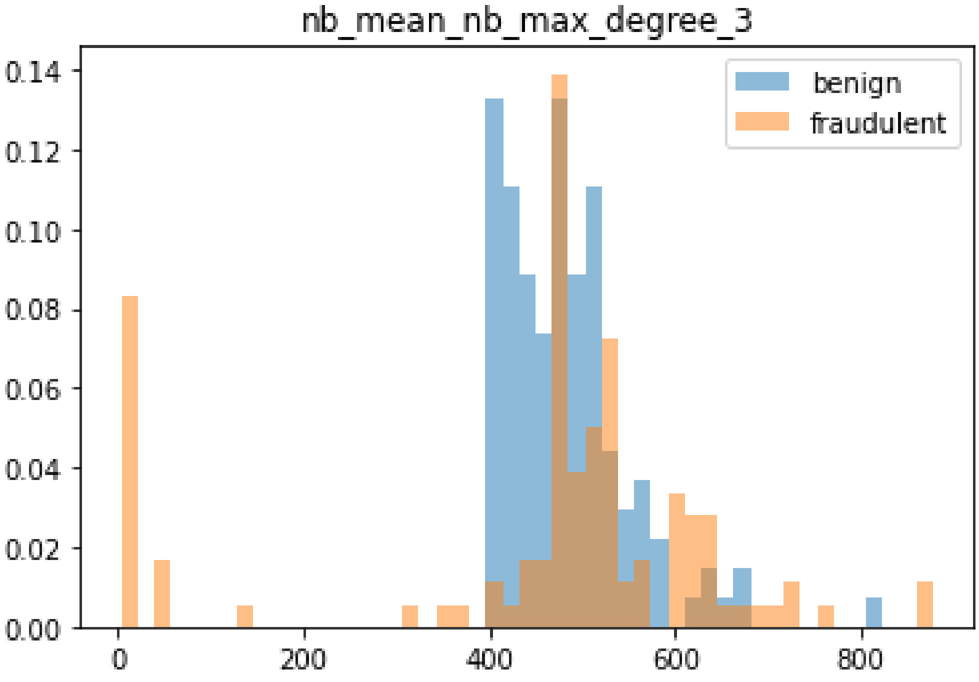
nb_mean_nb_max_degree_3
benign
fraudulent
0.00
0.02
0.04
0.06
0.08
0.10
0.12
0.14
0
200
400
600
800

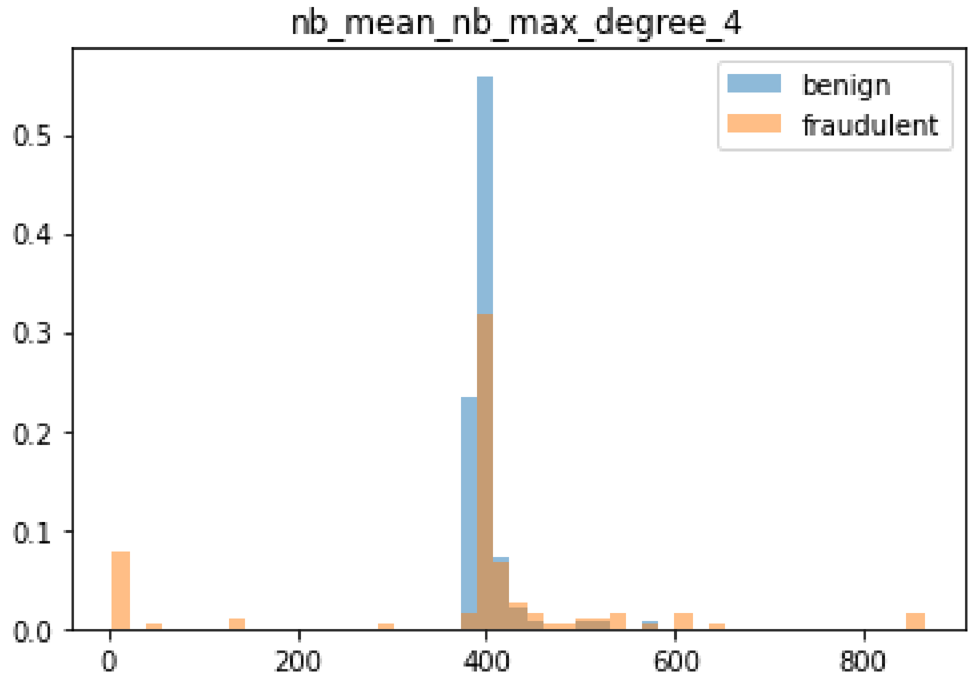
nb_mean_nb_max_degree_4
benign
fraudulent
0.5
0.4
0.3
0.2
0.1
0.0
0
200
400
600
800

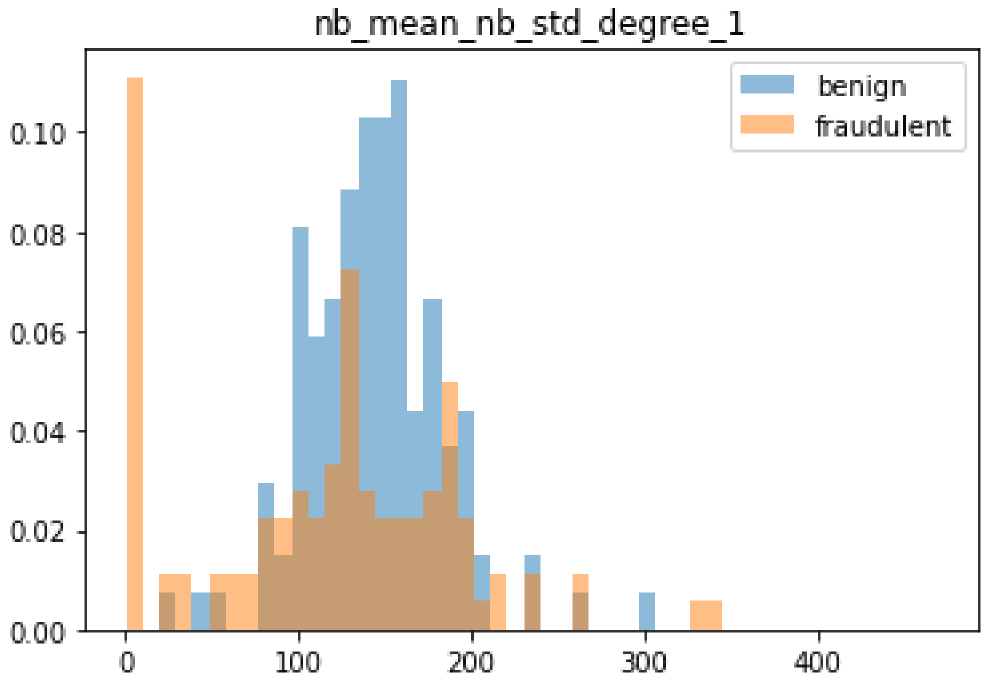
nb_mean_nb_std_degree_1
benign
fraudulent
0.10
0.08
0.06
0.04
0.02
0.00
0
100
200
300
400

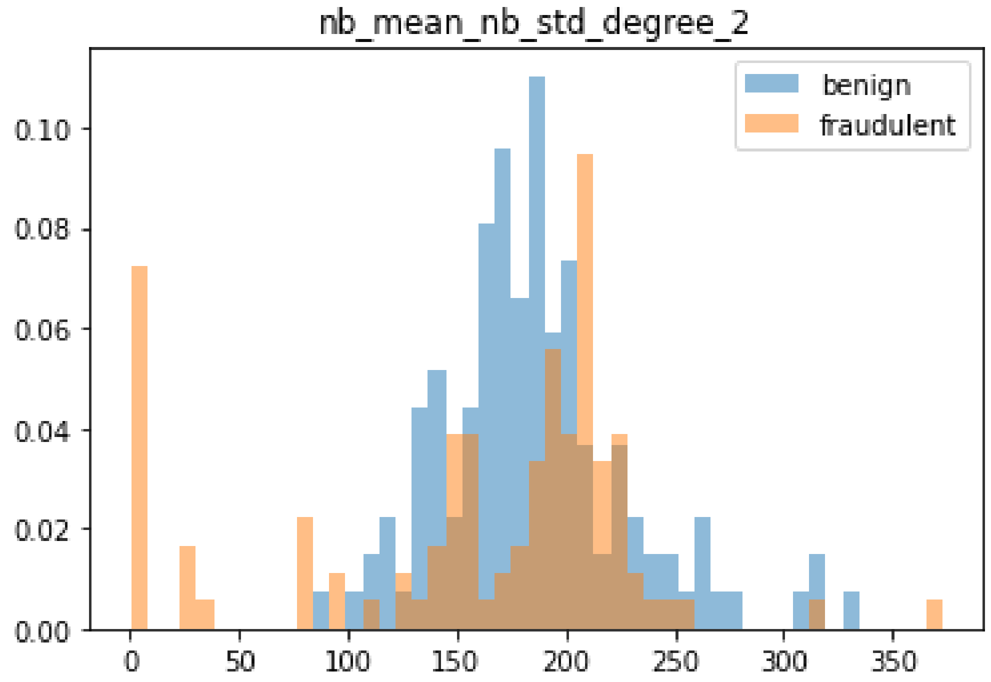
nb_mean_nb_std_degree_2
benign
fraudulent
0.10
0.08
0.06
0.04
0.02
0.00
0
50
100
150
200
250
300
350

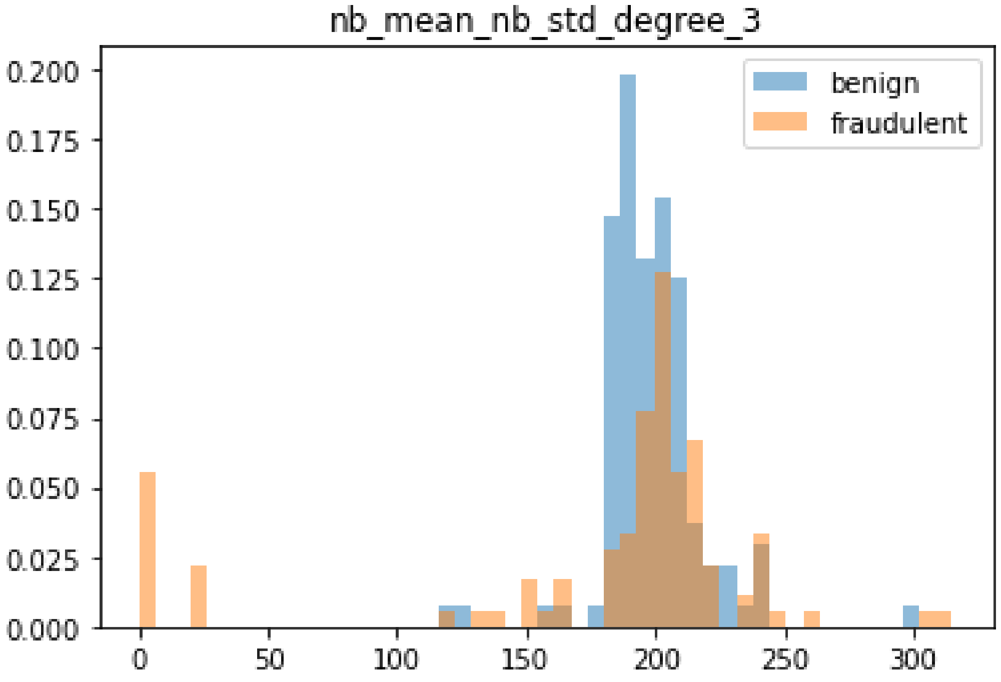
nb_mean_nb_std_degree_3
benign
fraudulent
0.200
0.175
0.150
0.125
0.100
0.075
0.050
0.025
0.000
0
50
100
150
200
250
300

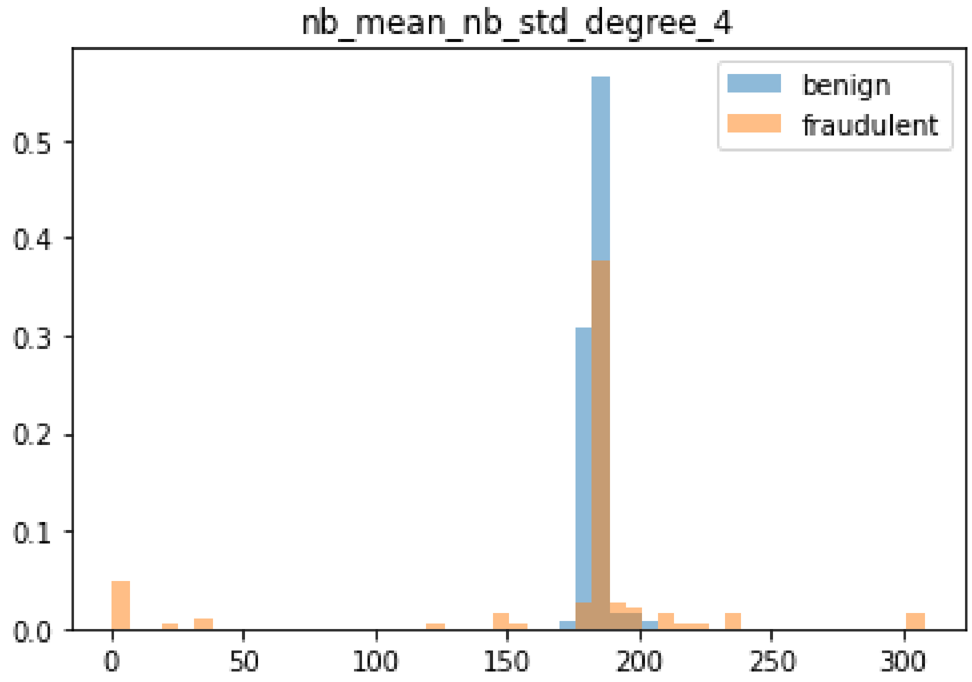
nb_mean_nb_std_degree_4
benign
fraudulent
0.0
0.1
0.2
0.3
0.4
0.5
0
50
100
150
200
250
300

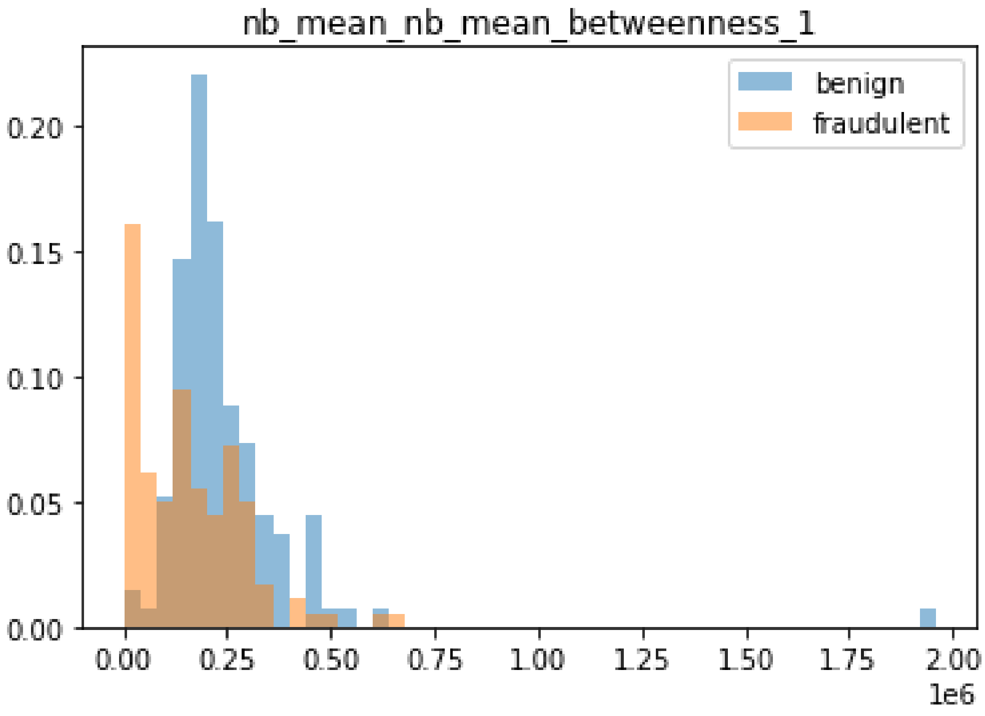
nb_mean_nb_mean_betweenness_1
benign
fraudulent
0.00
0.05
0.10
0.15
0.20
0.00
0.25
0.50
0.75
1.00
1.25
1.50
1.75
2.00
1e6

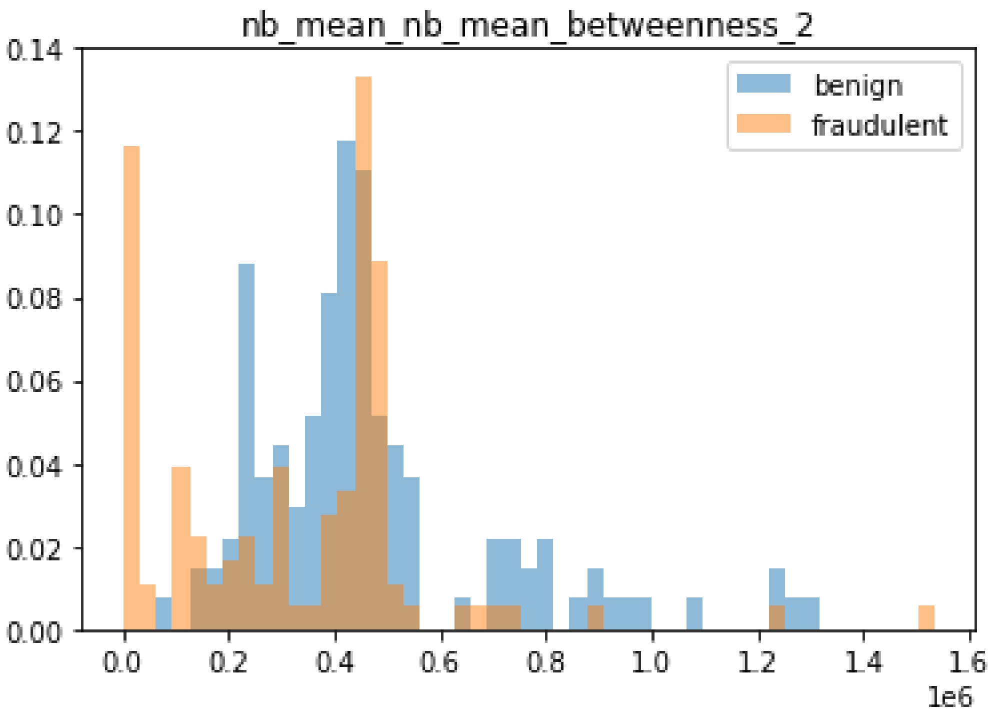
nb_mean_nb_mean_betweenness_2
0.14
0.12
0.10
0.08
0.06
0.04
0.02
0.00
0.0
0.2
0.4
0.6
0.8
1.0
1.2
1.4
1.6
1e6
benign
fraudulent

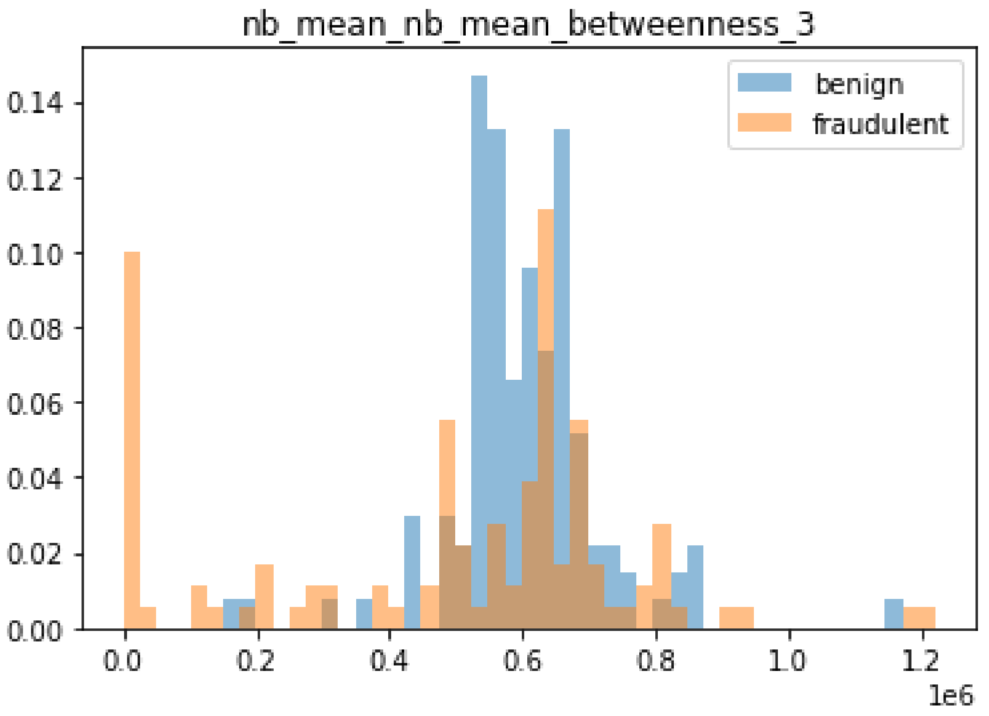
nb_mean_nb_mean_betweenness_3
0.14
0.12
0.10
0.08
0.06
0.04
0.02
0.00
0.0
0.2
0.4
0.6
0.8
1.0
1.2
1e6
benign
fraudulent

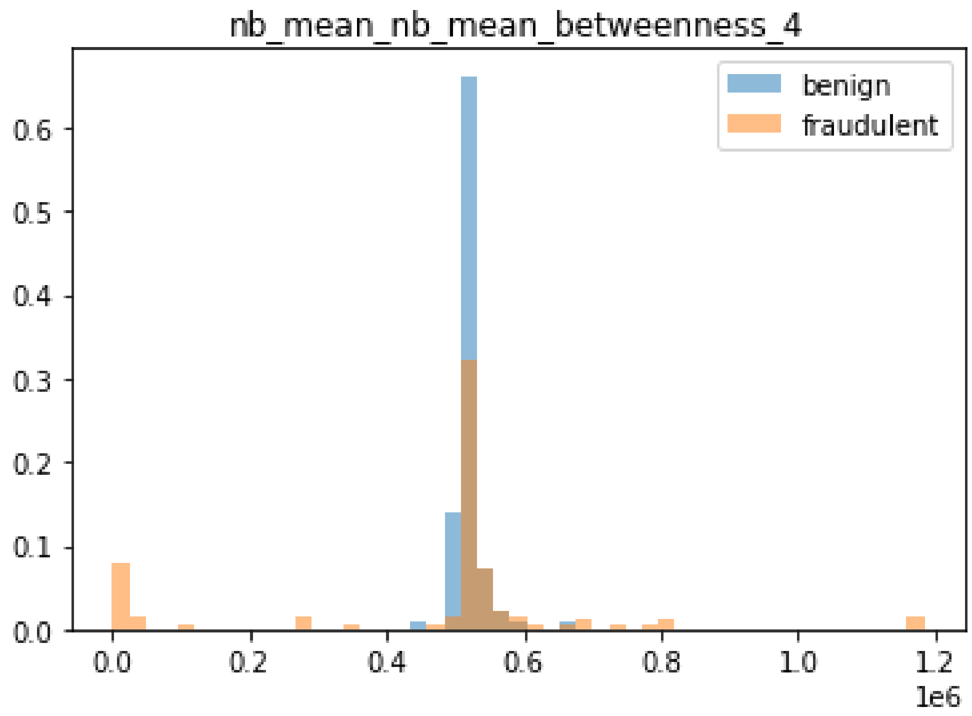
nb_mean_nb_mean_betweenness_4
benign
fraudulent
0.0
0.1
0.2
0.3
0.4
0.5
0.6
0.0
0.2
0.4
0.6
0.8
1.0
1.2
1e6

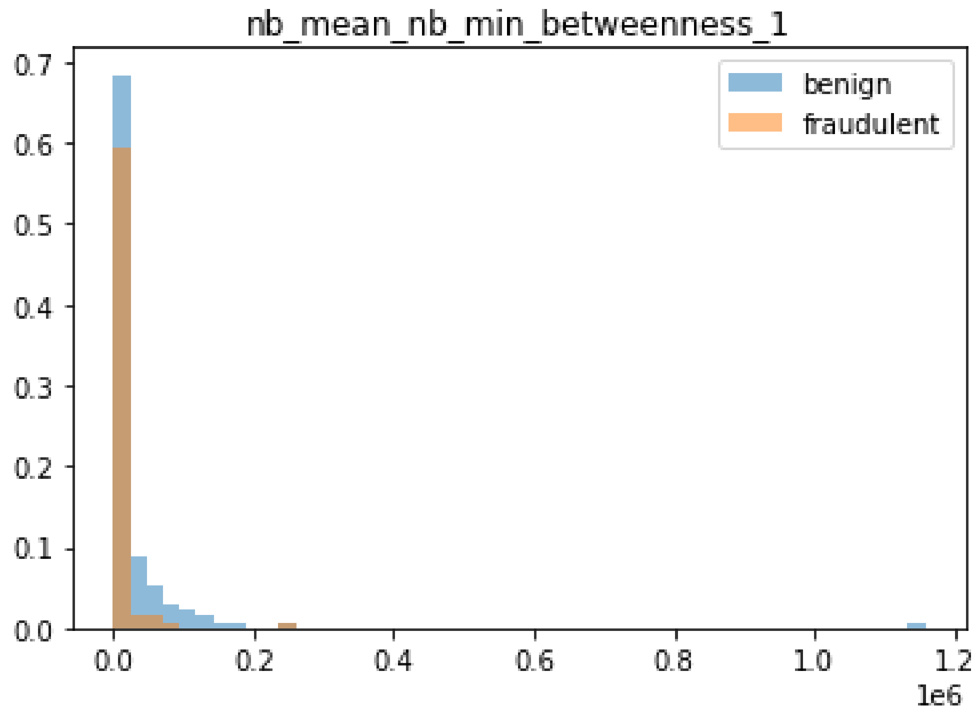
nb_mean_nb_min_betweenness_1
benign
fraudulent
0.0
0.1
0.2
0.3
0.4
0.5
0.6
0.7
0.0
0.2
0.4
0.6
0.8
1.0
1.2
1e6

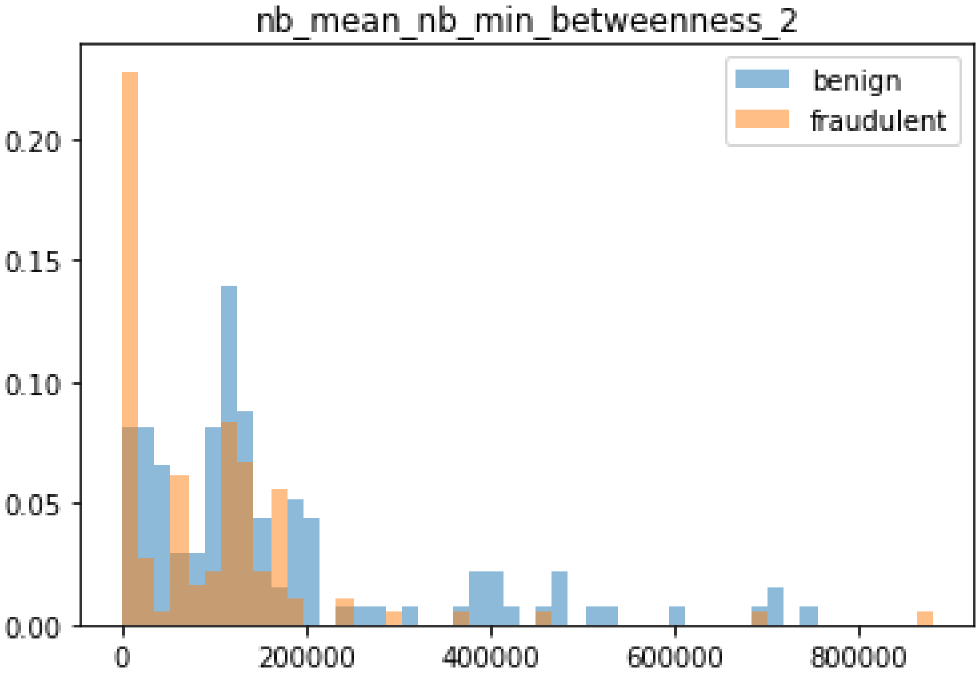
nb_mean_nb_min_betweenness_2
benign
fraudulent
0.20
0.15
0.10
0.05
0.00
0
200000
400000
600000
800000

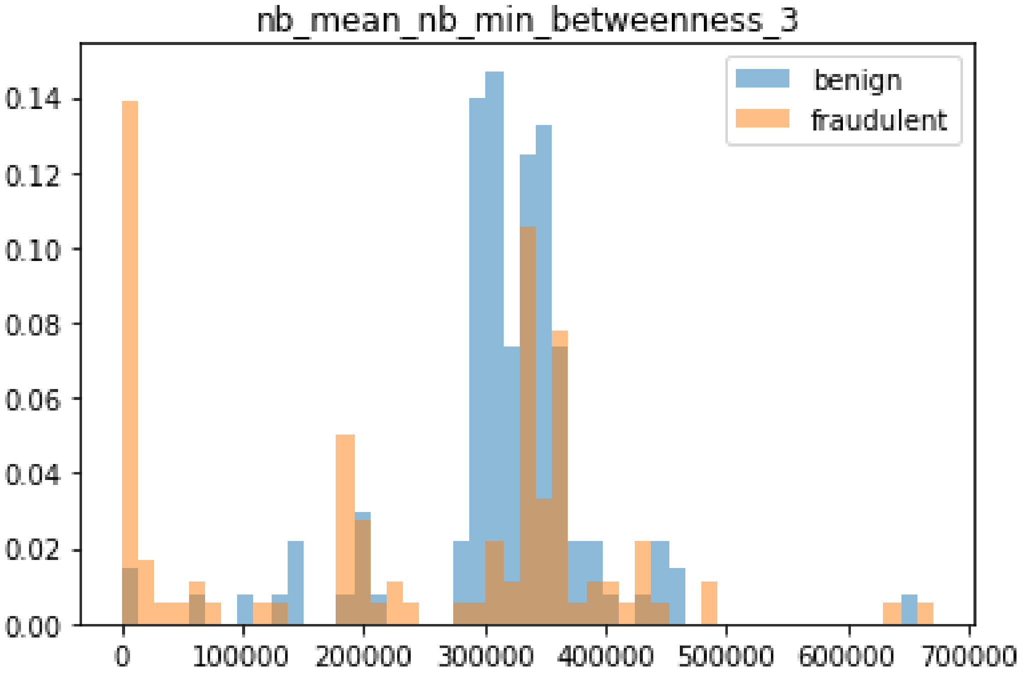
nb_mean_nb_min_betweenness_3
benign
fraudulent
0.14
0.12
0.10
0.08
0.06
0.04
0.02
0.00
0
100000
200000
300000
400000
500000
600000
700000

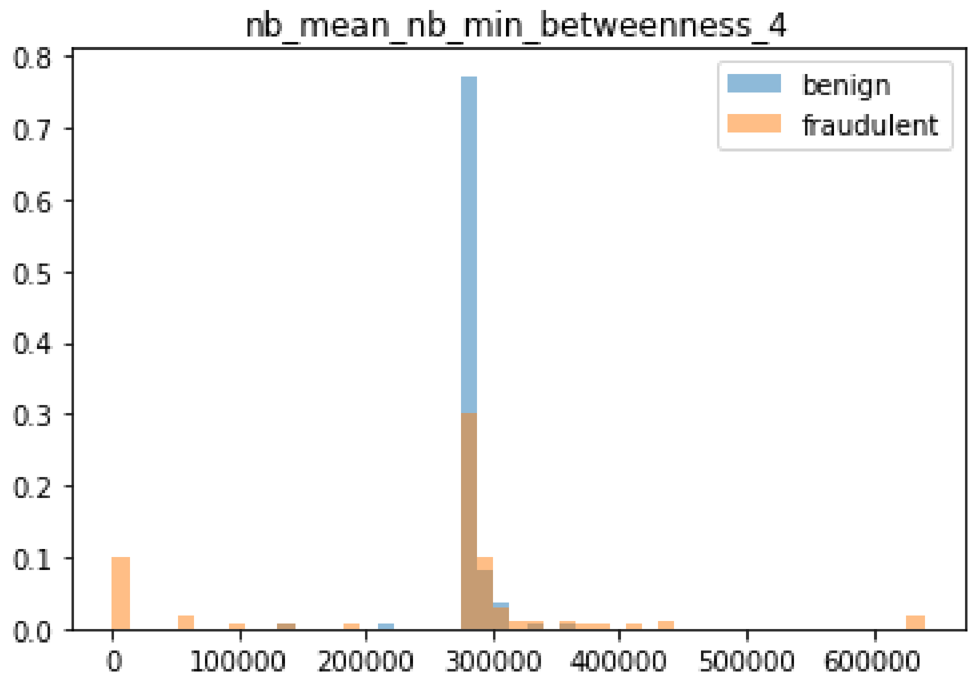
nb_mean_nb_min_betweenness_4
benign
fraudulent
0.8
0.7
0.6
0.5
0.4
0.3
0.2
0.1
0.0
0
100000
200000
300000
400000
500000
600000

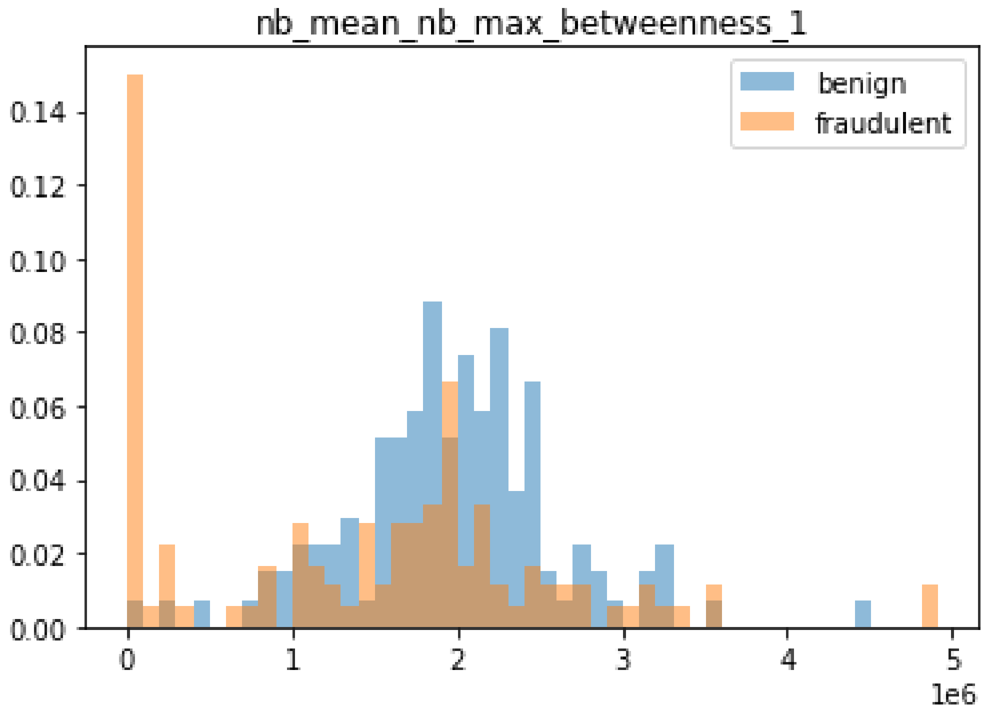
nb_mean_nb_max_betweenness_1
benign
fraudulent
0.14
0.12
0.10
0.08
0.06
0.04
0.02
0.00
0
1
2
3
4
5
1e6

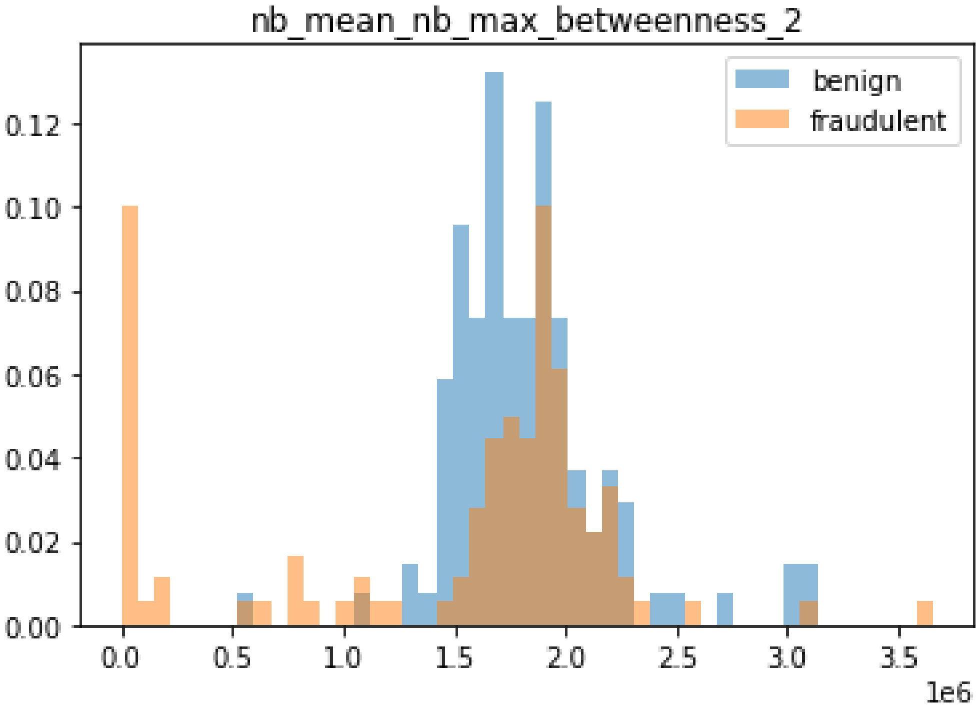
nb_mean_nb_max_betweenness_2
benign
fraudulent
0.12
0.10
0.08
0.06
0.04
0.02
0.00
0.0
0.5
1.0
1.5
2.0
2.5
3.0
3.5
1e6

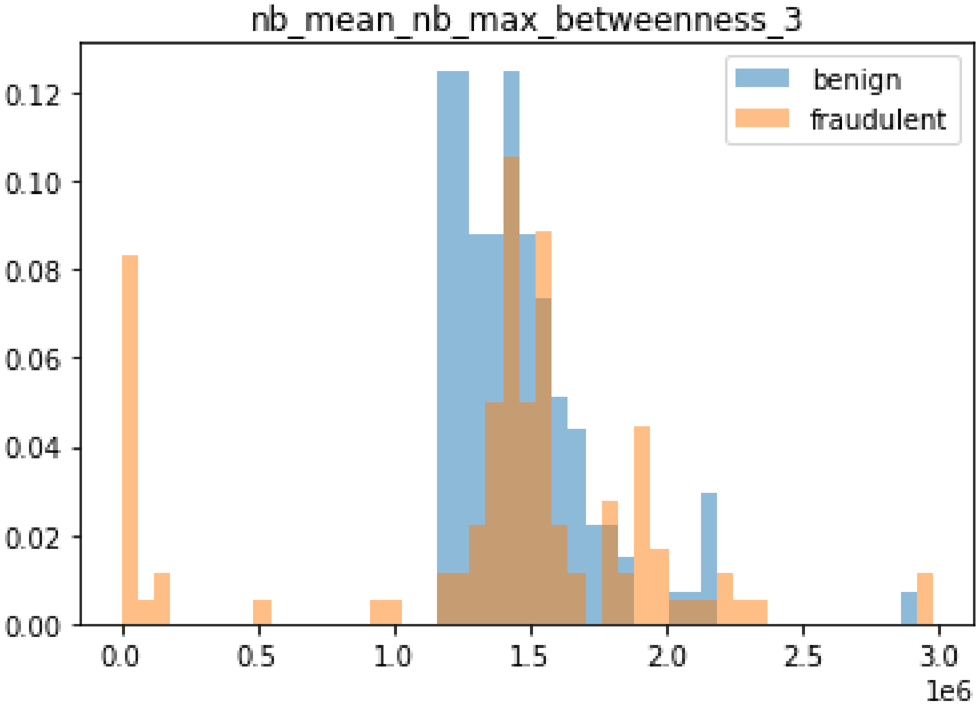
nb_mean_nb_max_betweenness_3
benign
fraudulent
0.12
0.10
0.08
0.06
0.04
0.02
0.00
0.0
0.5
1.0
1.5
2.0
2.5
3.0
1e6

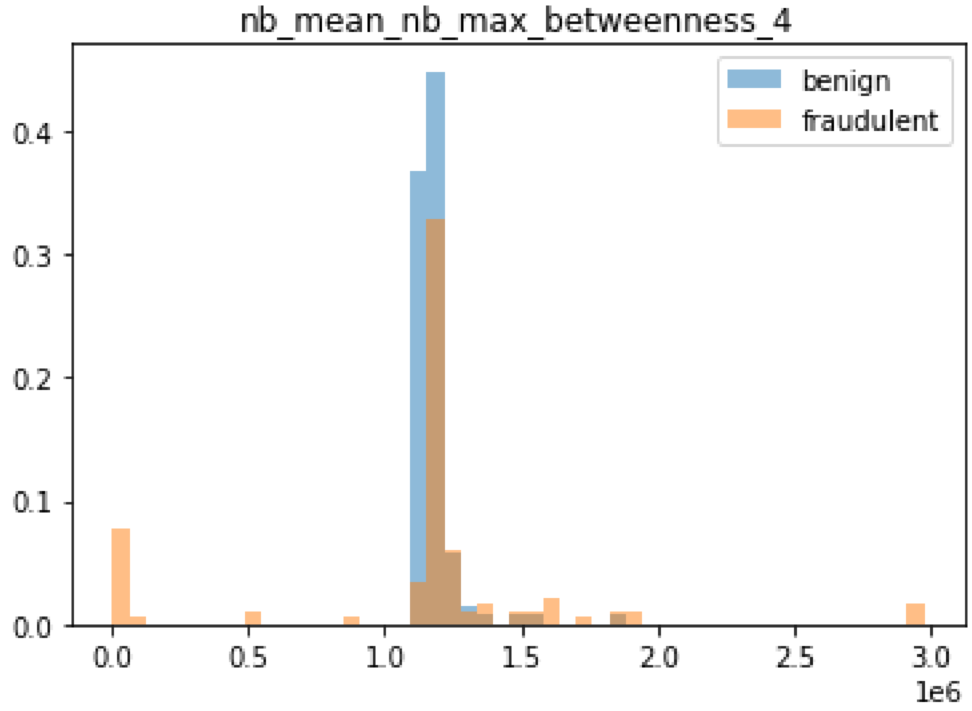

nb_mean_nb_max_betweenness_4
benign
fraudulent
0.0
0.1
0.2
0.3
0.4
0.0
0.5
1.0
1.5
2.0
2.5
3.0
1e6

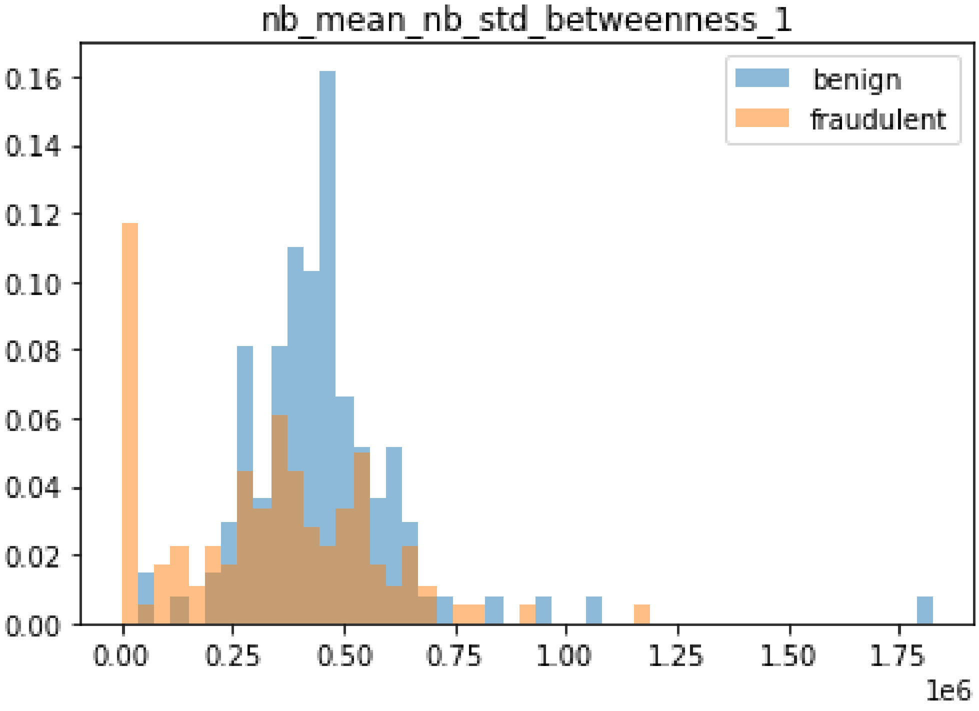

nb_mean_nb_std_betweenness_1
benign
fraudulent
0.00
0.02
0.04
0.06
0.08
0.10
0.12
0.14
0.16
0.00
0.25
0.50
0.75
1.00
1.25
1.50
1.75
1e6

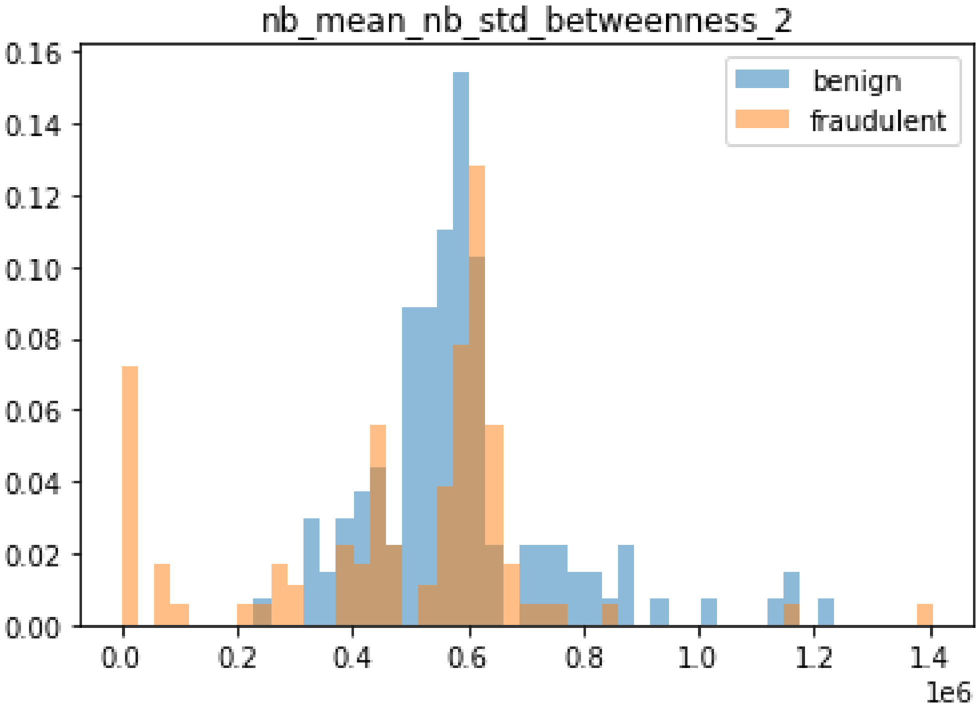
nb_mean_nb_std_betweenness_2
benign
fraudulent
0.16
0.14
0.12
0.10
0.08
0.06
0.04
0.02
0.00
0.0
0.2
0.4
0.6
0.8
1.0
1.2
1.4
1e6

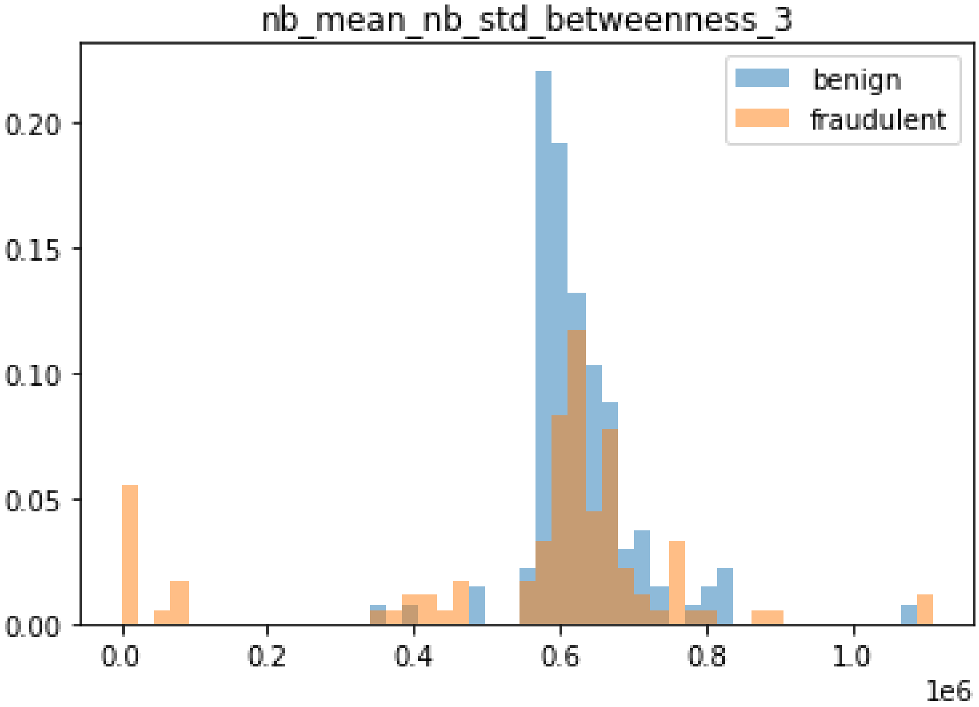
nb_mean_nb_std_betweenness_3
benign
fraudulent
0.20
0.15
0.10
0.05
0.00
0.0
0.2
0.4
0.6
0.8
1.0
1e6

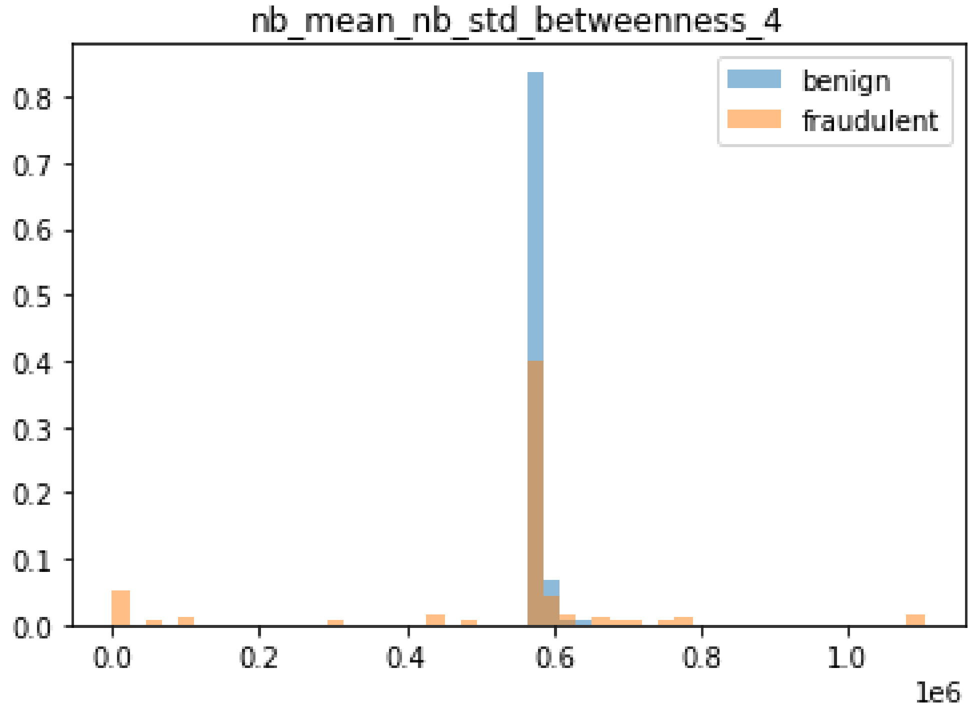
nb_mean_nb_std_betweenness_4
benign
fraudulent
0.0
0.1
0.2
0.3
0.4
0.5
0.6
0.7
0.8
0.0
0.2
0.4
0.6
0.8
1.0
1e6

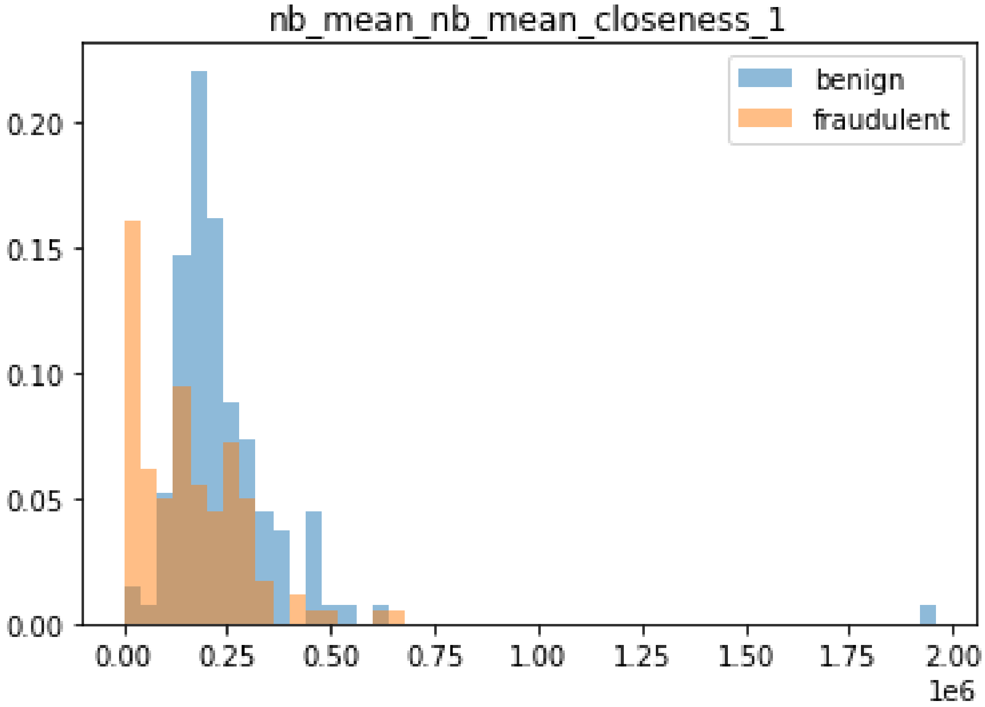
nb_mean_nb_mean_closeness_1
benign
fraudulent
0.00
0.05
0.10
0.15
0.20
0.00
0.25
0.50
0.75
1.00
1.25
1.50
1.75
2.00
1e6

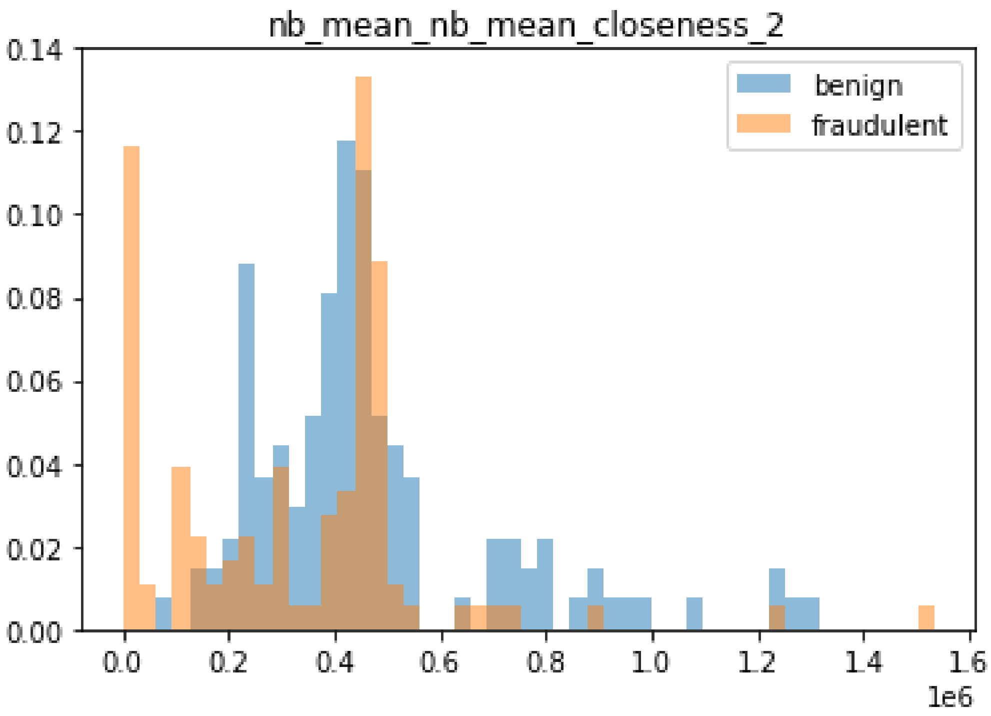
nb_mean_nb_mean_closeness_2
benign
fraudulent
0.14
0.12
0.10
0.08
0.06
0.04
0.02
0.00
0.0
0.2
0.4
0.6
0.8
1.0
1.2
1.4
1.6
1e6

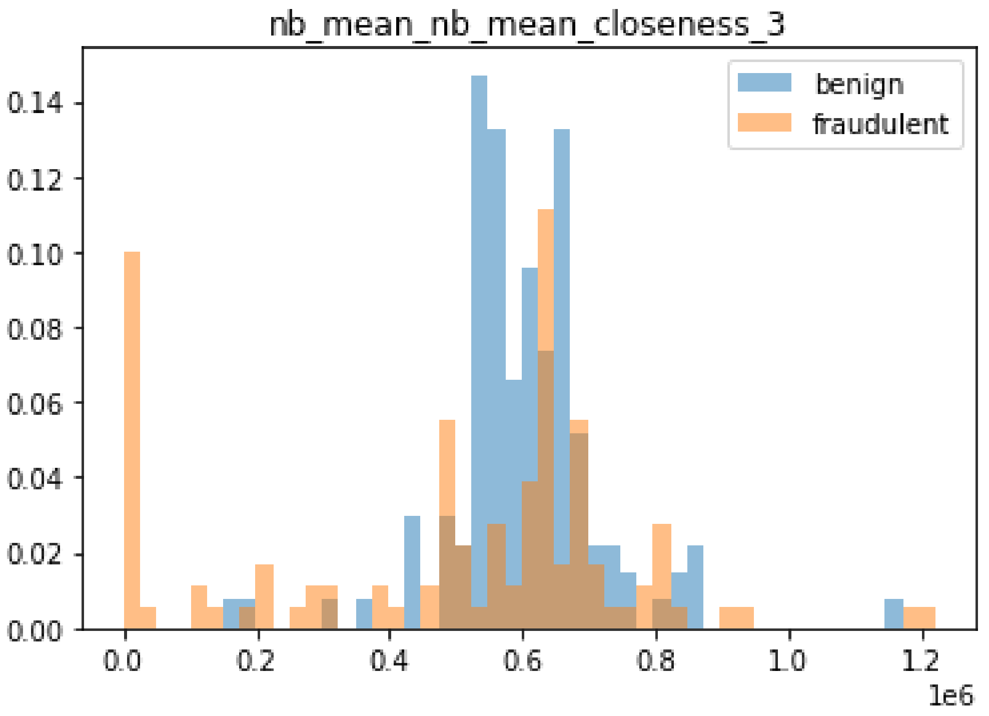
nb_mean_nb_mean_closeness_3
benign
fraudulent
0.14
0.12
0.10
0.08
0.06
0.04
0.02
0.00
0.0
0.2
0.4
0.6
0.8
1.0
1.2
1e6

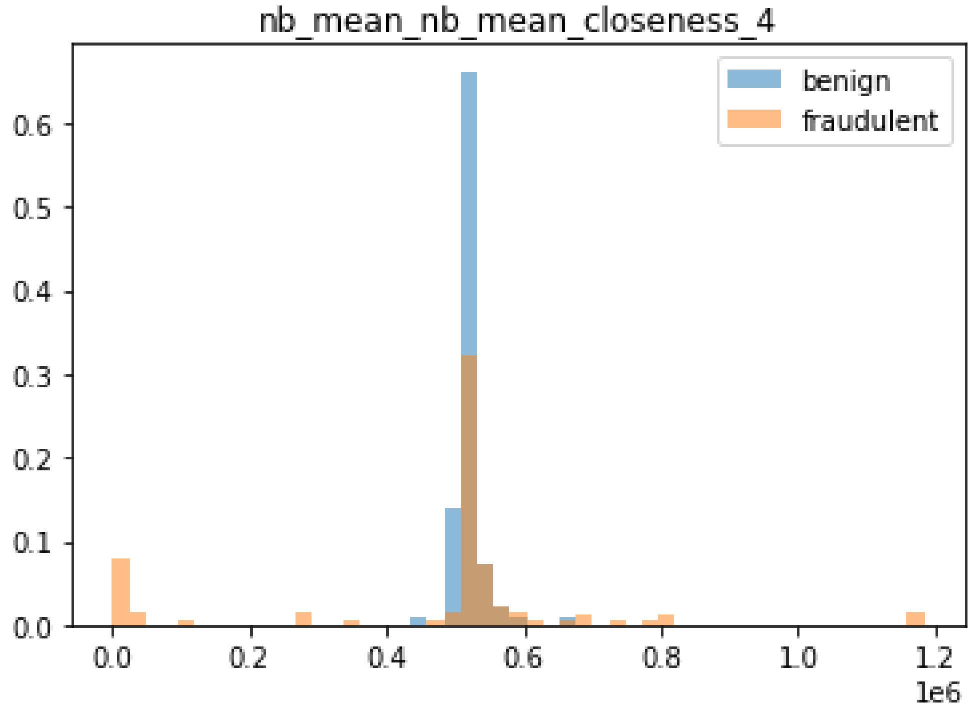
nb_mean_nb_mean_closeness_4
benign
fraudulent
0.0
0.1
0.2
0.3
0.4
0.5
0.6
0.0
0.2
0.4
0.6
0.8
1.0
1.2
1e6

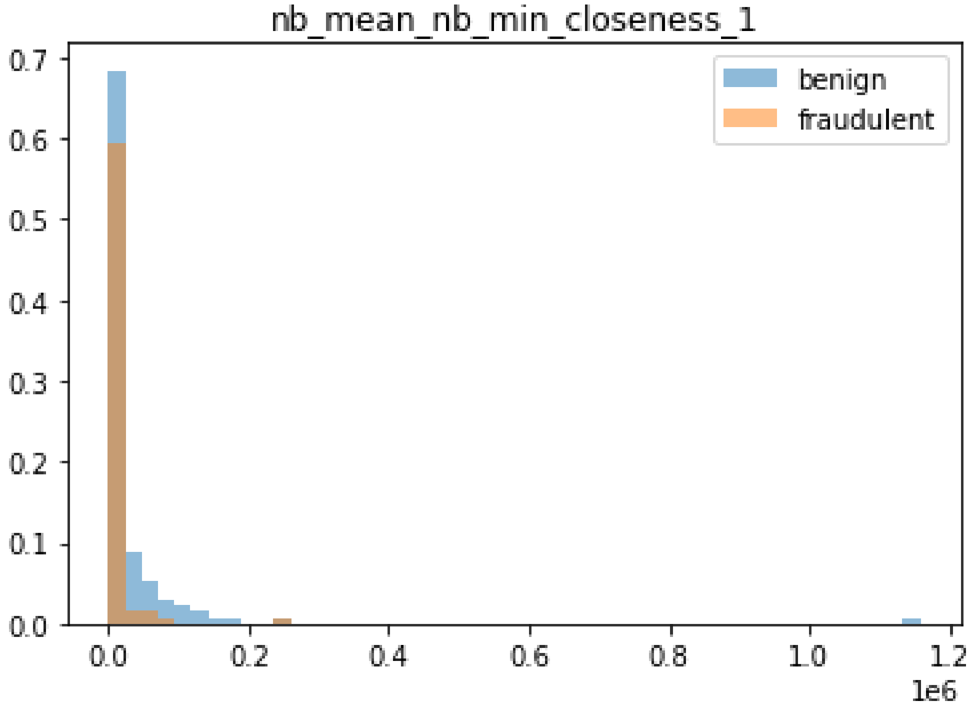
nb_mean_nb_min_closeness_1
benign
fraudulent
0.0
0.1
0.2
0.3
0.4
0.5
0.6
0.7
0.0
0.2
0.4
0.6
0.8
1.0
1.2
1e6

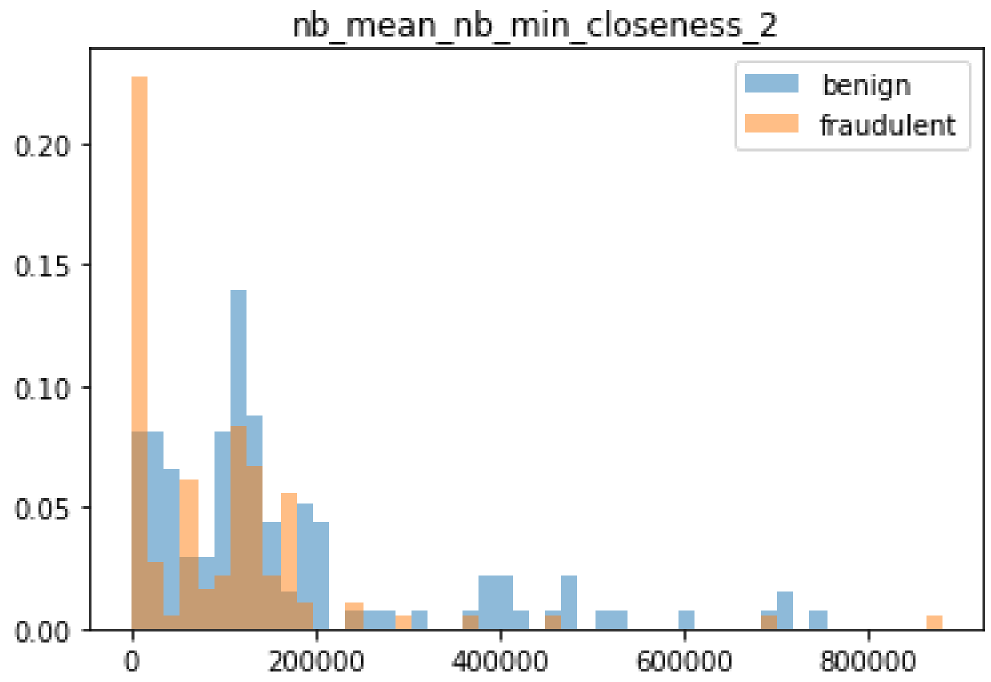
nb_mean_nb_min_closeness_2
benign
fraudulent
0.20
0.15
0.10
0.05
0.00
0
200000
400000
600000
800000

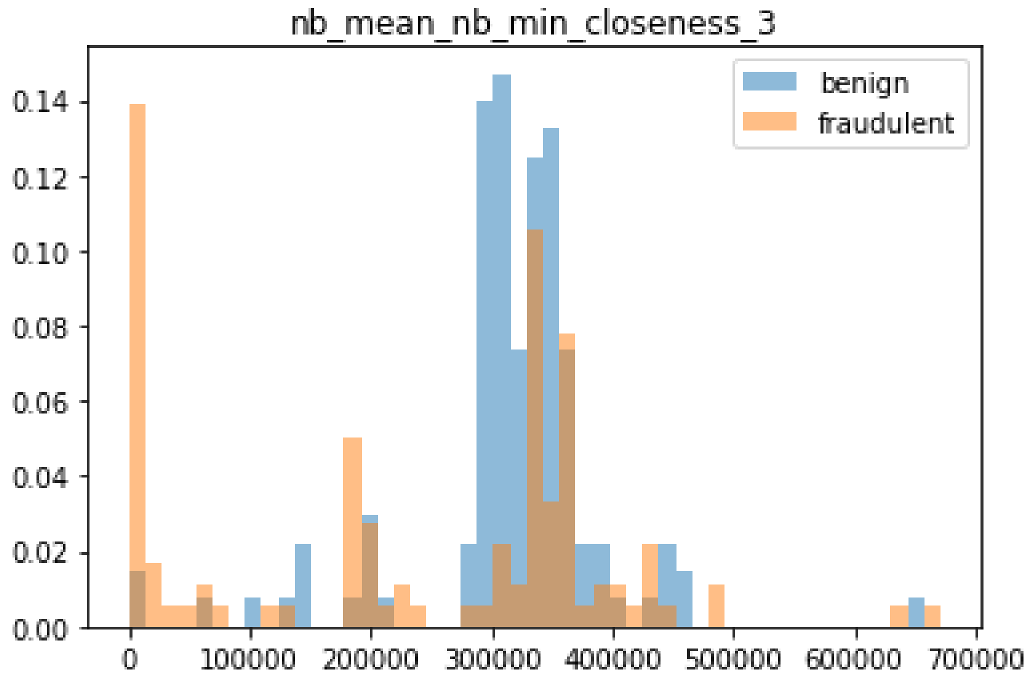
nb_mean_nb_min_closeness_3
benign
fraudulent
0.14
0.12
0.10
0.08
0.06
0.04
0.02
0.00
0
100000
200000
300000
400000
500000
600000
700000

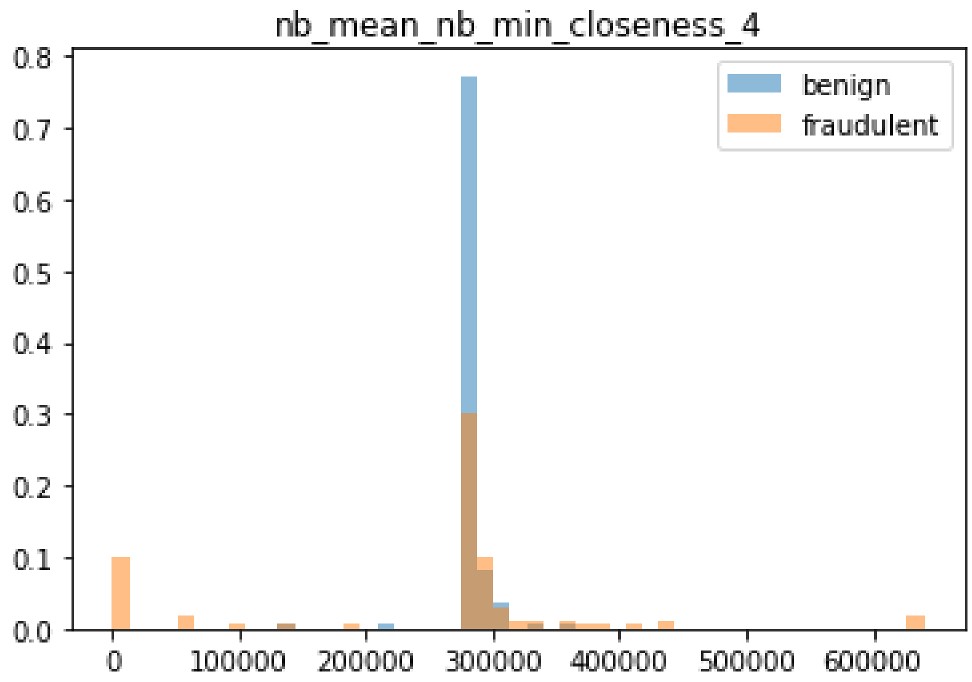
nb_mean_nb_min_closeness_4
benign
fraudulent
0.8
0.7
0.6
0.5
0.4
0.3
0.2
0.1
0.0
0
100000
200000
300000
400000
500000
600000

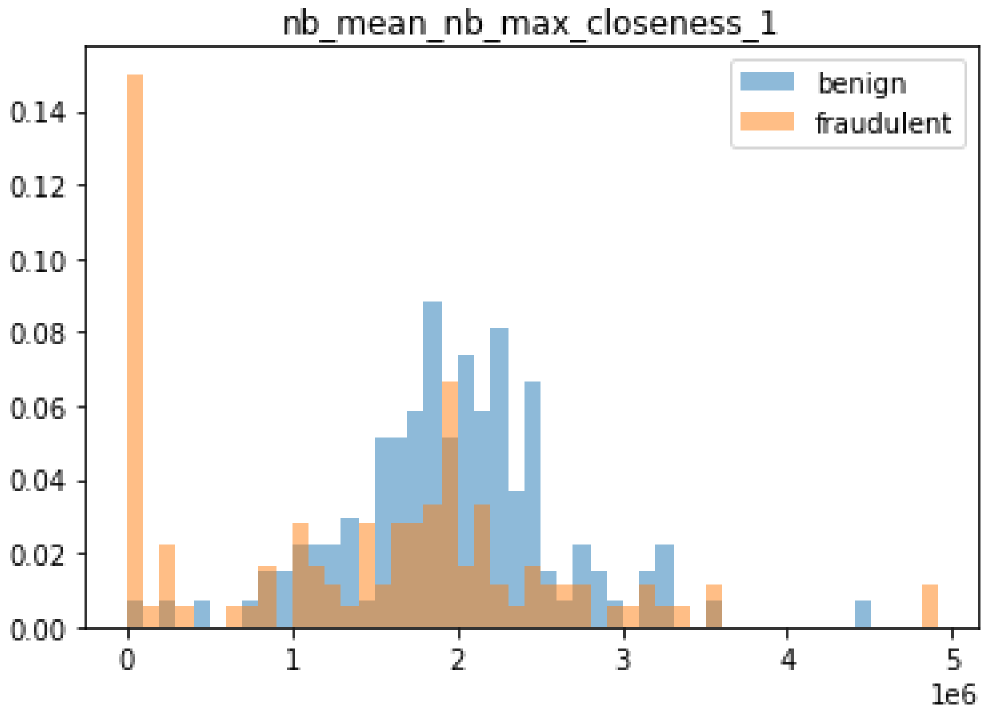
nb_mean_nb_max_closeness_1
benign
fraudulent
0.14
0.12
0.10
0.08
0.06
0.04
0.02
0.00
0
1
2
3
4
5
1e6

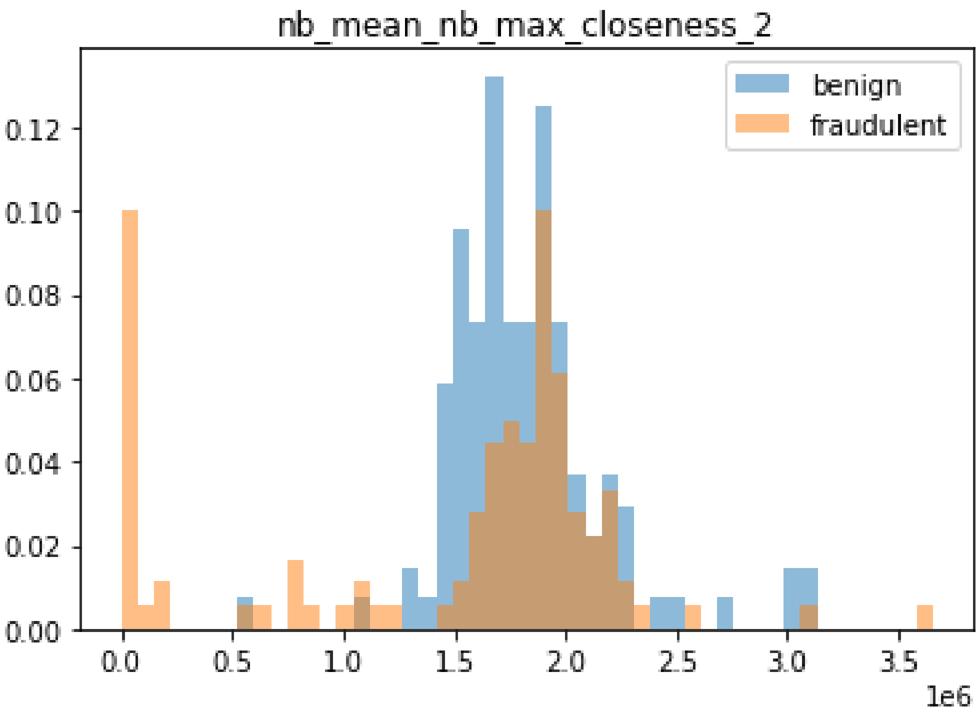

nb_mean_nb_max_closeness_2
benign
fraudulent
0.12
0.10
0.08
0.06
0.04
0.02
0.00
0.0
0.5
1.0
1.5
2.0
2.5
3.0
3.5
1e6

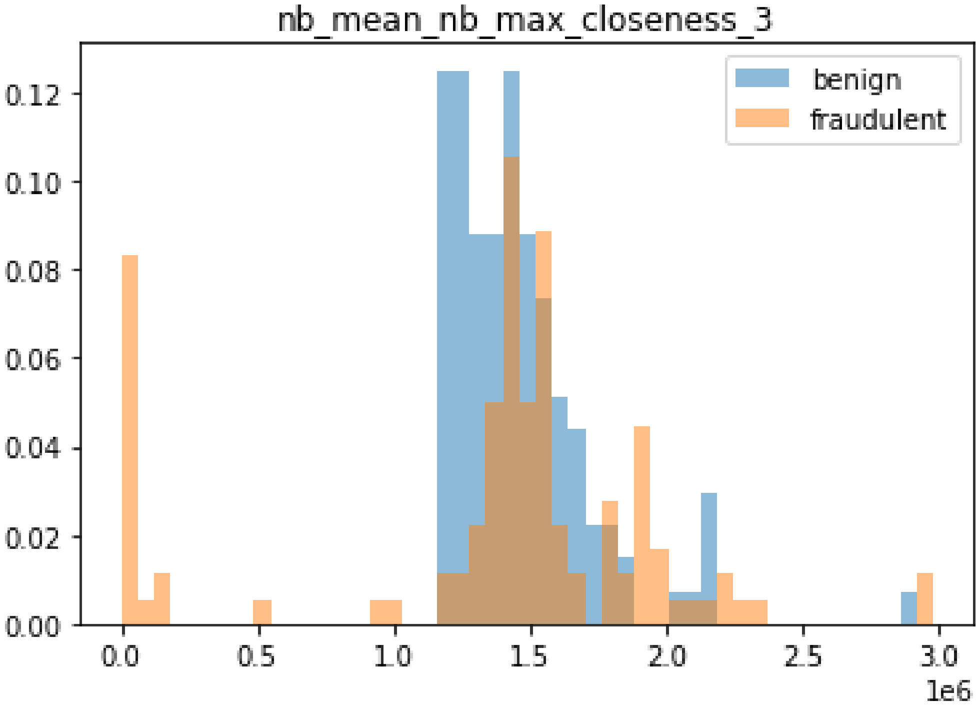

nb_mean_nb_max_closeness_3
benign
fraudulent
0.12
0.10
0.08
0.06
0.04
0.02
0.00
0.0
0.5
1.0
1.5
2.0
2.5
3.0
1e6

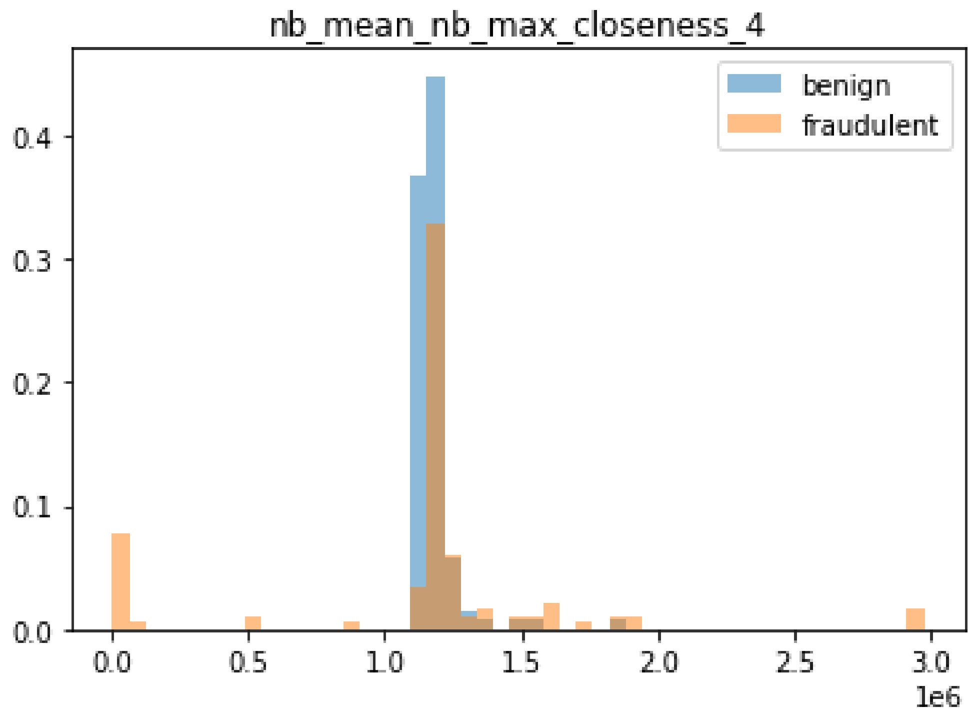
nb_mean_nb_max_closeness_4
benign
fraudulent
0.0
0.1
0.2
0.3
0.4
0.0
0.5
1.0
1.5
2.0
2.5
3.0
1e6

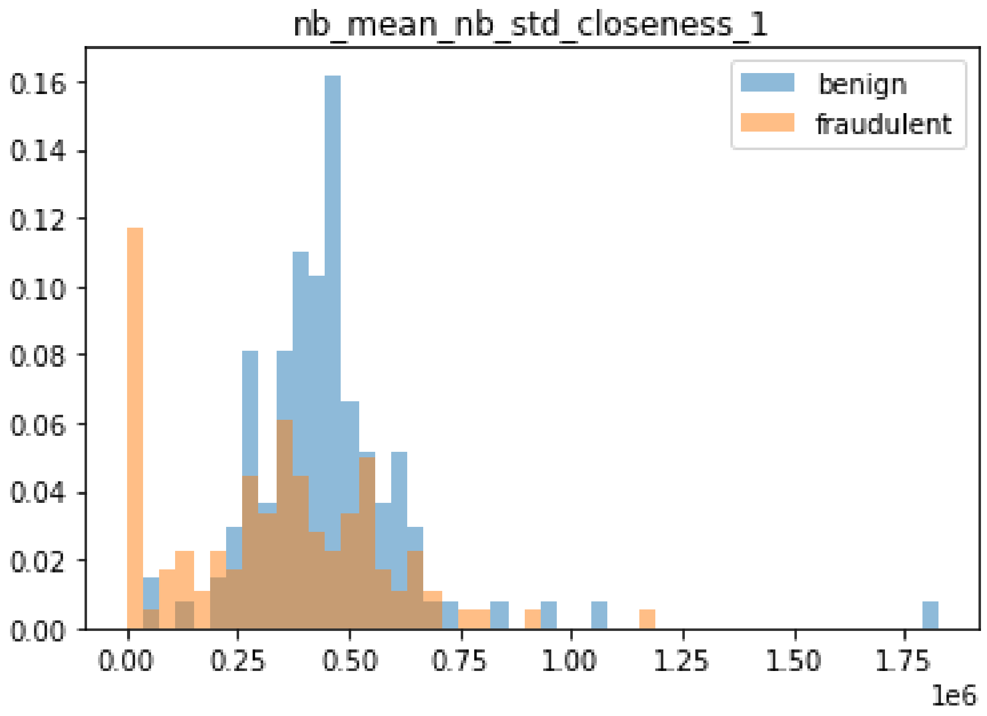
nb_mean_nb_std_closeness_1
benign
fraudulent
0.00
0.02
0.04
0.06
0.08
0.10
0.12
0.14
0.16
0.00
0.25
0.50
0.75
1.00
1.25
1.50
1.75
1e6

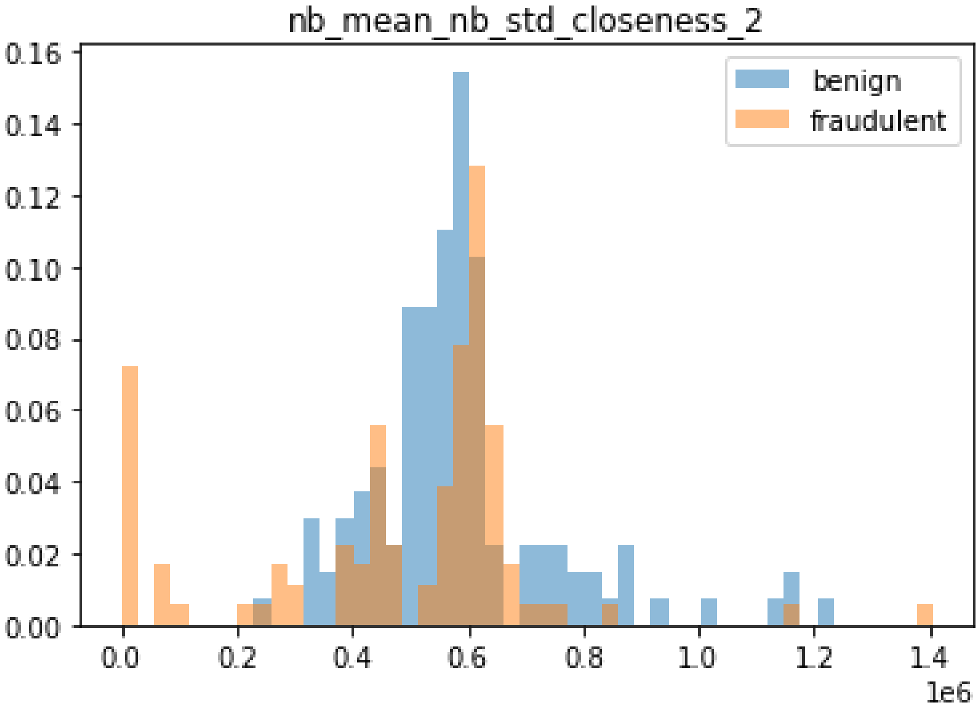
nb_mean_nb_std_closeness_2
benign
fraudulent
0.16
0.14
0.12
0.10
0.08
0.06
0.04
0.02
0.00
0.0
0.2
0.4
0.6
0.8
1.0
1.2
1.4
1e6

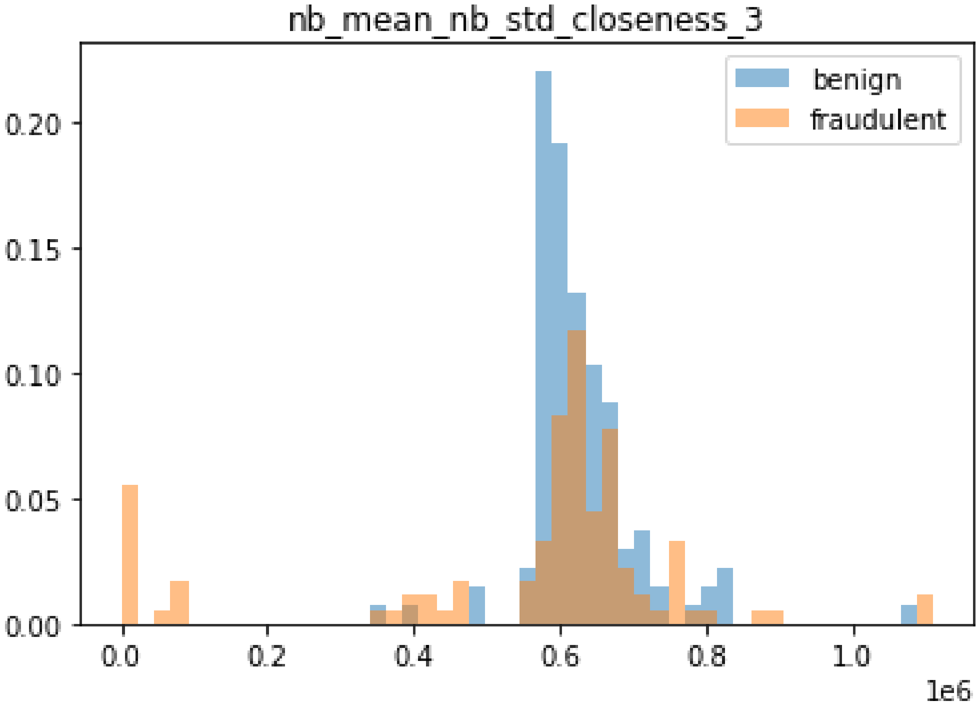
nb_mean_nb_std_closeness_3
benign
fraudulent
0.20
0.15
0.10
0.05
0.00
0.0
0.2
0.4
0.6
0.8
1.0
1e6

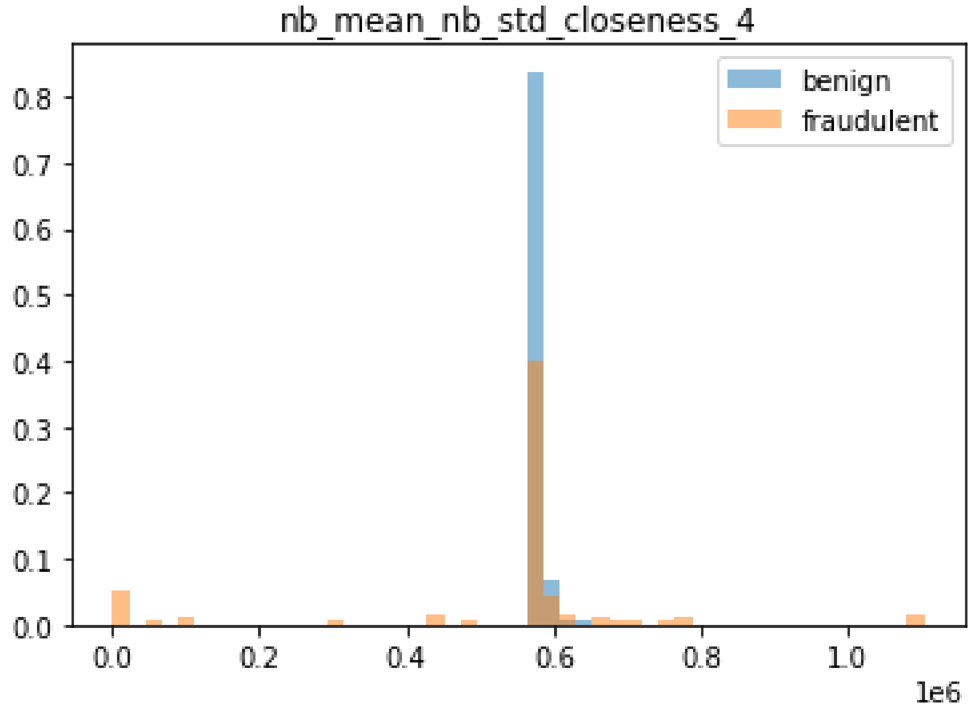
nb_mean_nb_std_closeness_4
benign
fraudulent
0.0
0.1
0.2
0.3
0.4
0.5
0.6
0.7
0.8
0.0
0.2
0.4
0.6
0.8
1.0
1e6

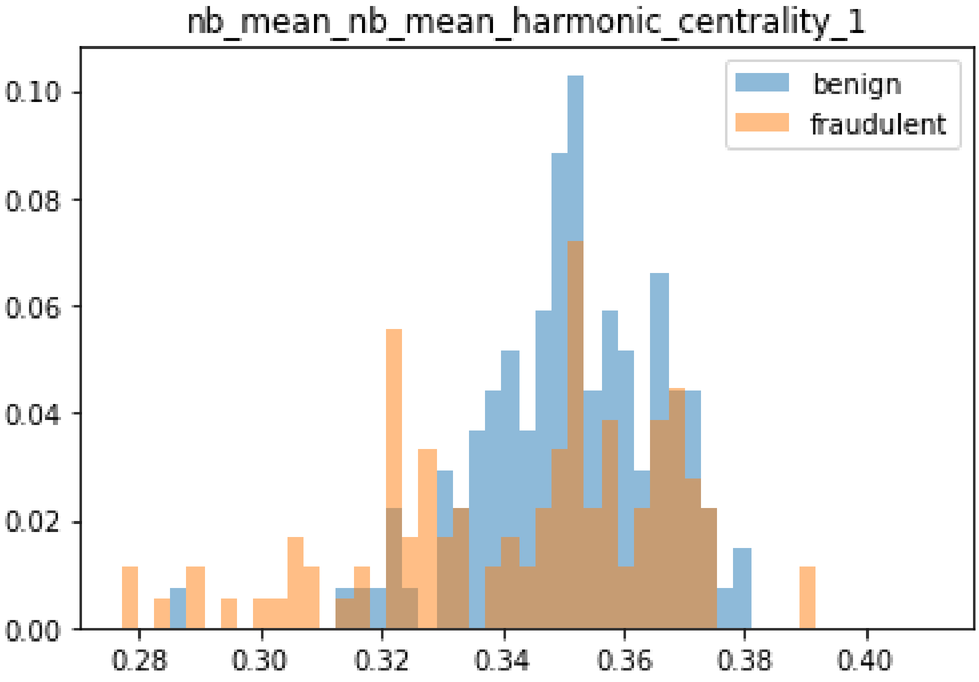
nb_mean_nb_mean_harmonic_centrality_1
benign
fraudulent
0.00
0.02
0.04
0.06
0.08
0.10
0.28
0.30
0.32
0.34
0.36
0.38
0.40

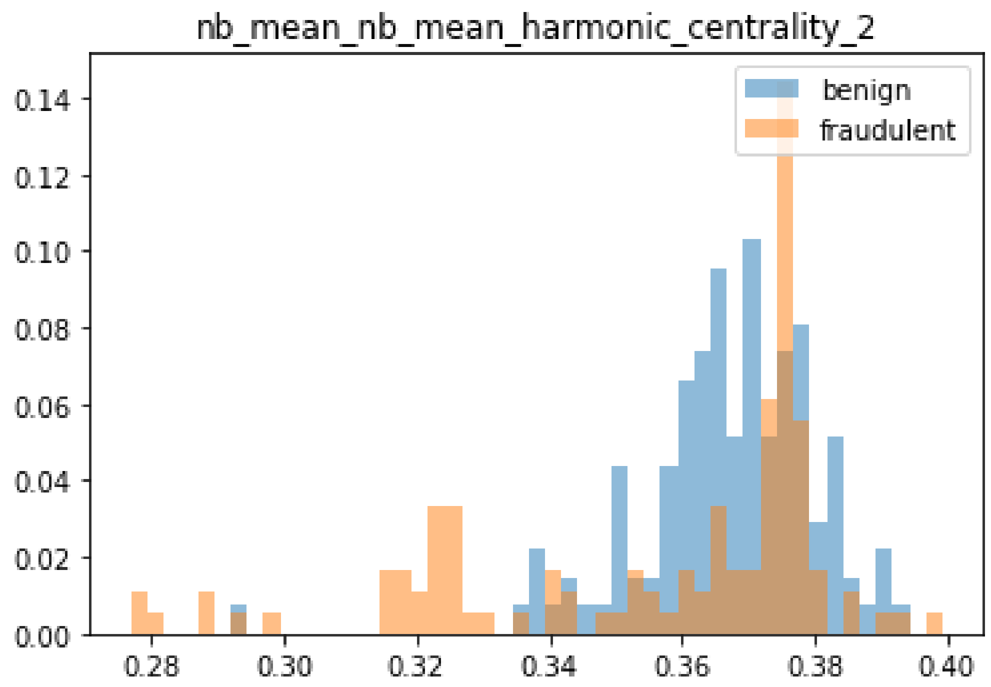
nb_mean_nb_mean_harmonic_centrality_2
benign
fraudulent
0.14
0.12
0.10
0.08
0.06
0.04
0.02
0.00
0.28
0.30
0.32
0.34
0.36
0.38
0.40

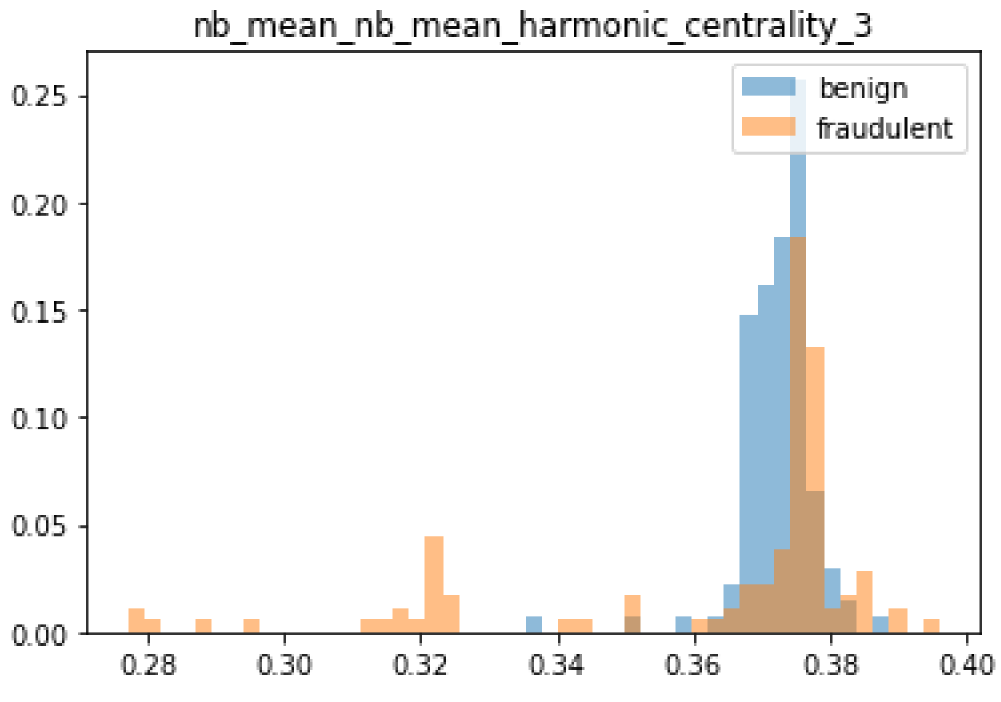
nb_mean_nb_mean_harmonic_centrality_3
benign
fraudulent
0.25
0.20
0.15
0.10
0.05
0.00
0.28
0.30
0.32
0.34
0.36
0.38
0.40

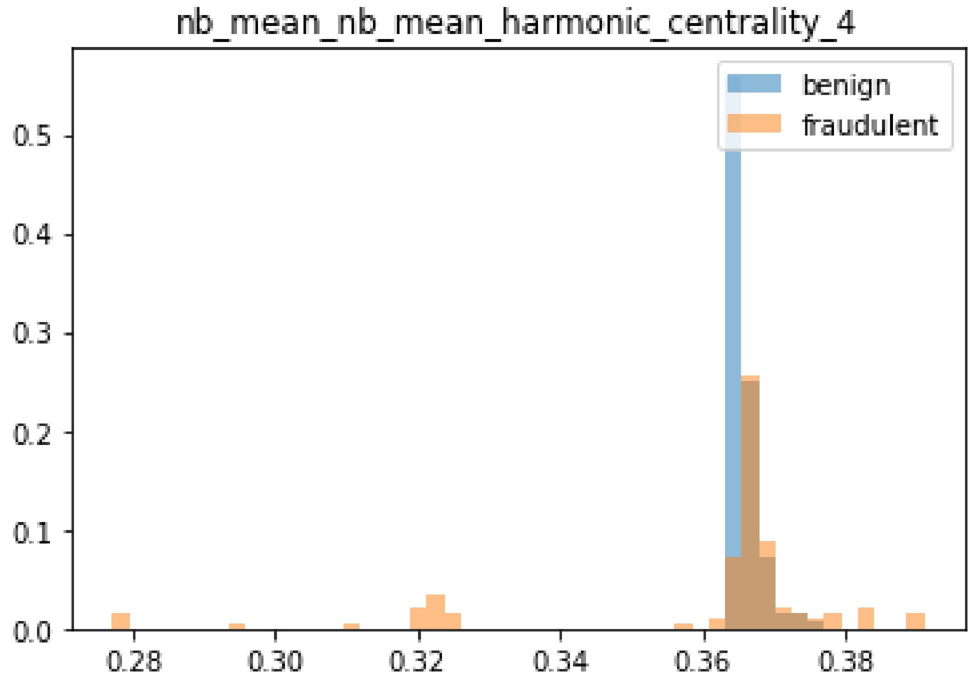
nb_mean_nb_mean_harmonic_centrality_4
benign
fraudulent
0.0
0.1
0.2
0.3
0.4
0.5
0.28
0.30
0.32
0.34
0.36
0.38

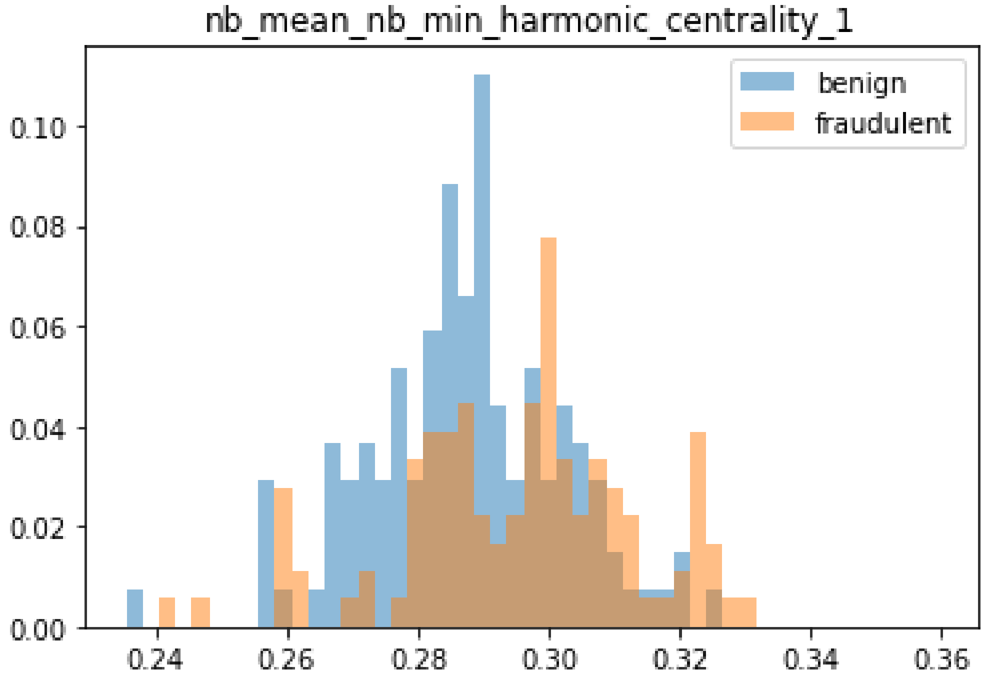
nb_mean_nb_min_harmonic_centrality_1
benign
fraudulent
0.00
0.02
0.04
0.06
0.08
0.10
0.24
0.26
0.28
0.30
0.32
0.34
0.36

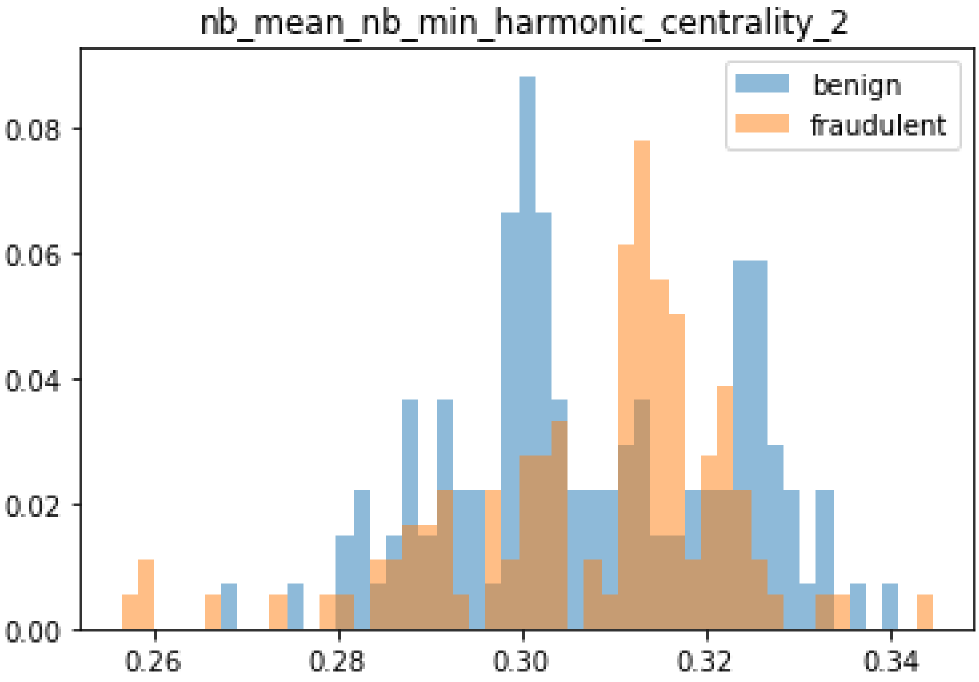
nb_mean_nb_min_harmonic_centrality_2
benign
fraudulent
0.00
0.02
0.04
0.06
0.08
0.26
0.28
0.30
0.32
0.34

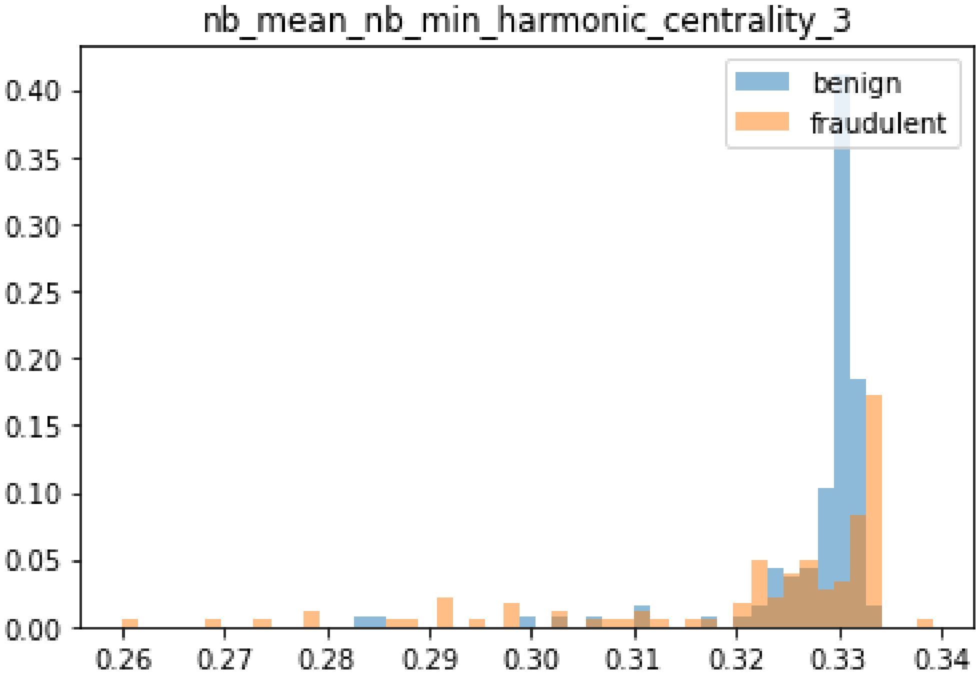
nb_mean_nb_min_harmonic_centrality_3
benign
fraudulent
0.00
0.05
0.10
0.15
0.20
0.25
0.30
0.35
0.40
0.26
0.27
0.28
0.29
0.30
0.31
0.32
0.33
0.34

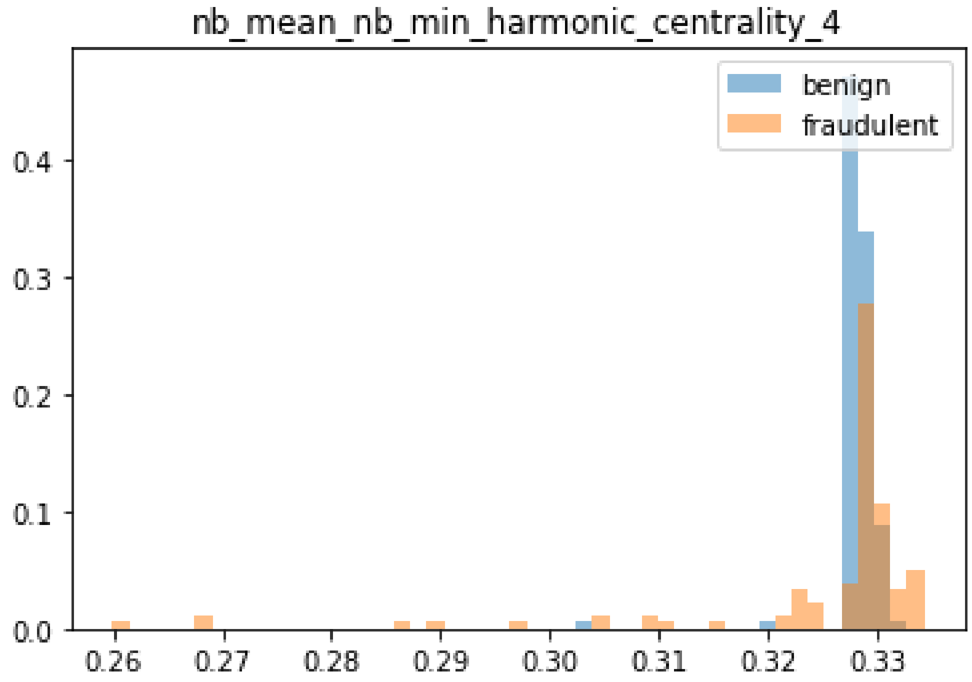
nb_mean_nb_min_harmonic_centrality_4
benign
fraudulent
0.4
0.3
0.2
0.1
0.0
0.26
0.27
0.28
0.29
0.30
0.31
0.32
0.33

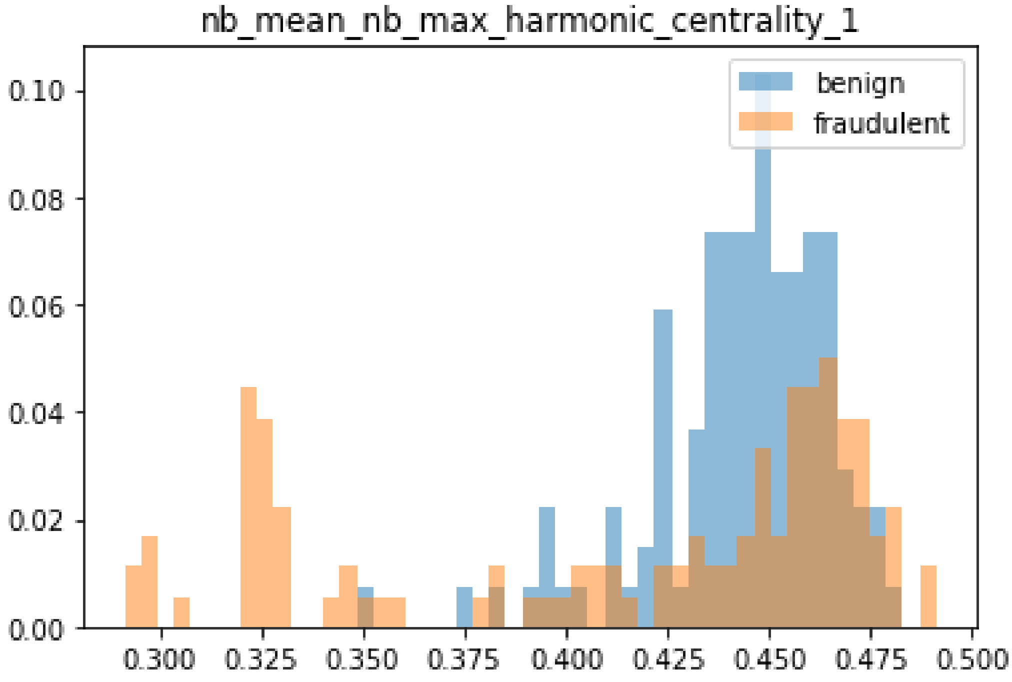
nb_mean_nb_max_harmonic_centrality_1
benign
fraudulent
0.10
0.08
0.06
0.04
0.02
0.00
0.300
0.325
0.350
0.375
0.400
0.425
0.450
0.475
0.500

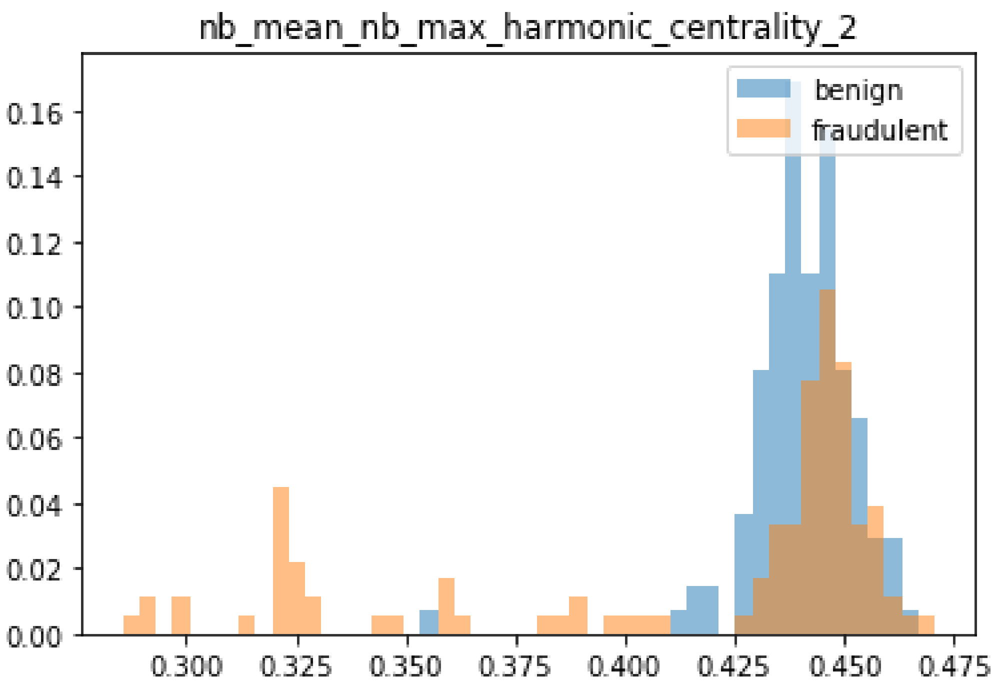
nb_mean_nb_max_harmonic_centrality_2
benign
fraudulent
0.16
0.14
0.12
0.10
0.08
0.06
0.04
0.02
0.00
0.300
0.325
0.350
0.375
0.400
0.425
0.450
0.475

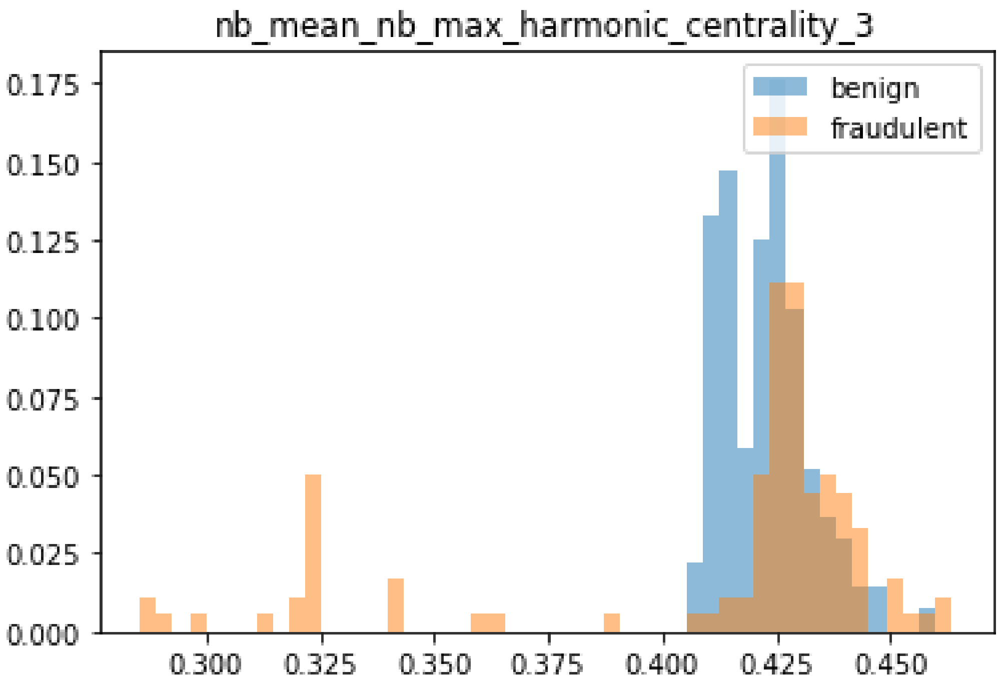
nb_mean_nb_max_harmonic_centrality_3
benign
fraudulent
0.175
0.150
0.125
0.100
0.075
0.050
0.025
0.000
0.300
0.325
0.350
0.375
0.400
0.425
0.450

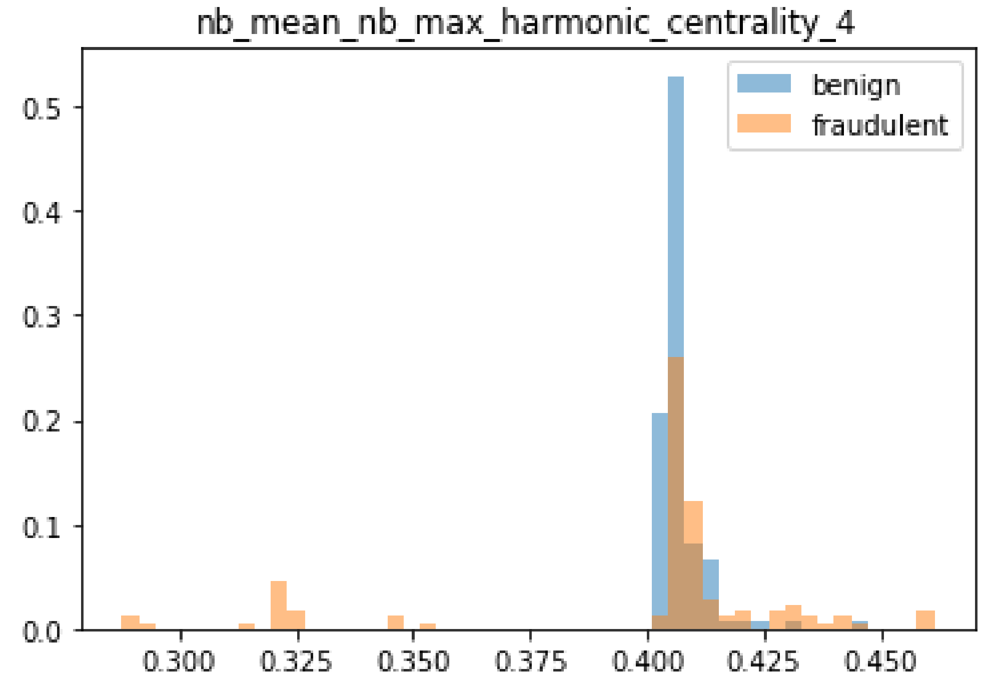
nb_mean_nb_max_harmonic_centrality_4
benign
fraudulent
0.0
0.1
0.2
0.3
0.4
0.5
0.300
0.325
0.350
0.375
0.400
0.425
0.450

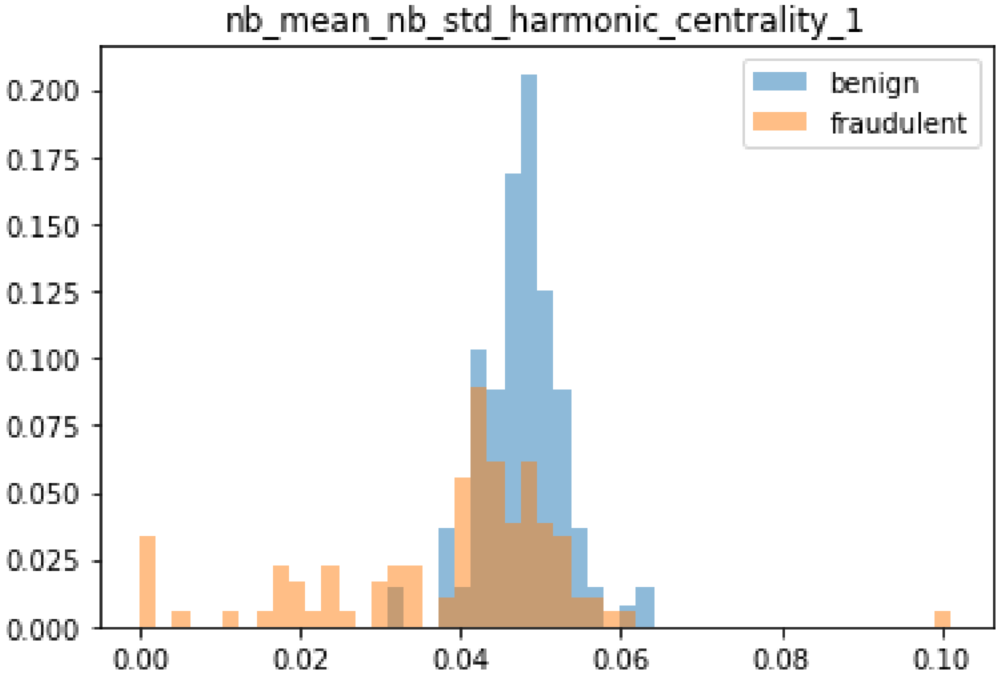
nb_mean_nb_std_harmonic_centrality_1
benign
fraudulent
0.000
0.025
0.050
0.075
0.100
0.125
0.150
0.175
0.200
0.00
0.02
0.04
0.06
0.08
0.10

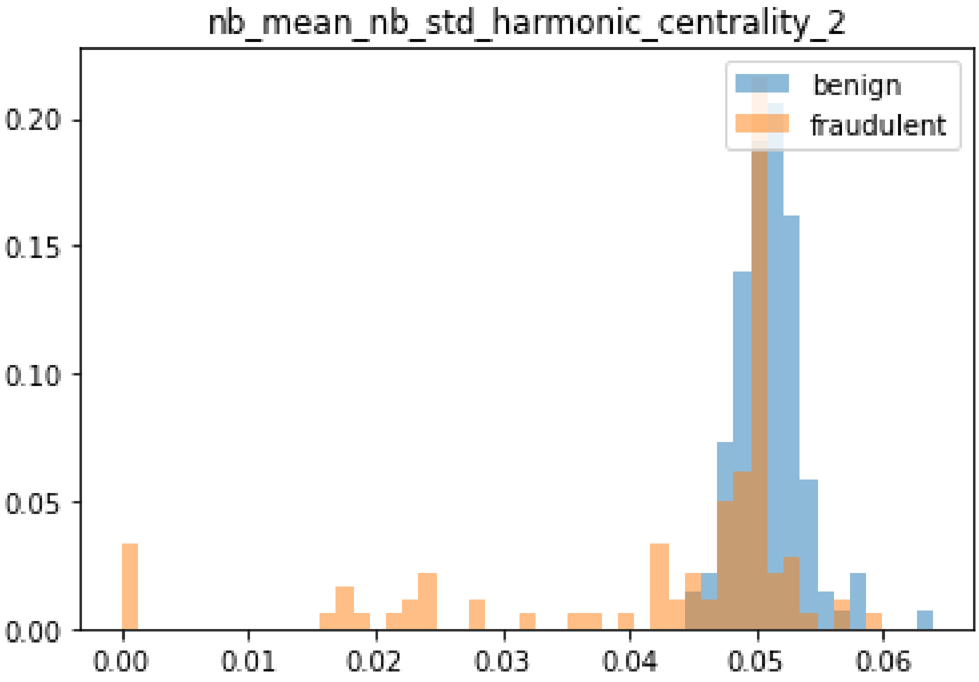
nb_mean_nb_std_harmonic_centrality_2
benign
fraudulent
0.20
0.15
0.10
0.05
0.00
0.00
0.01
0.02
0.03
0.04
0.05
0.06

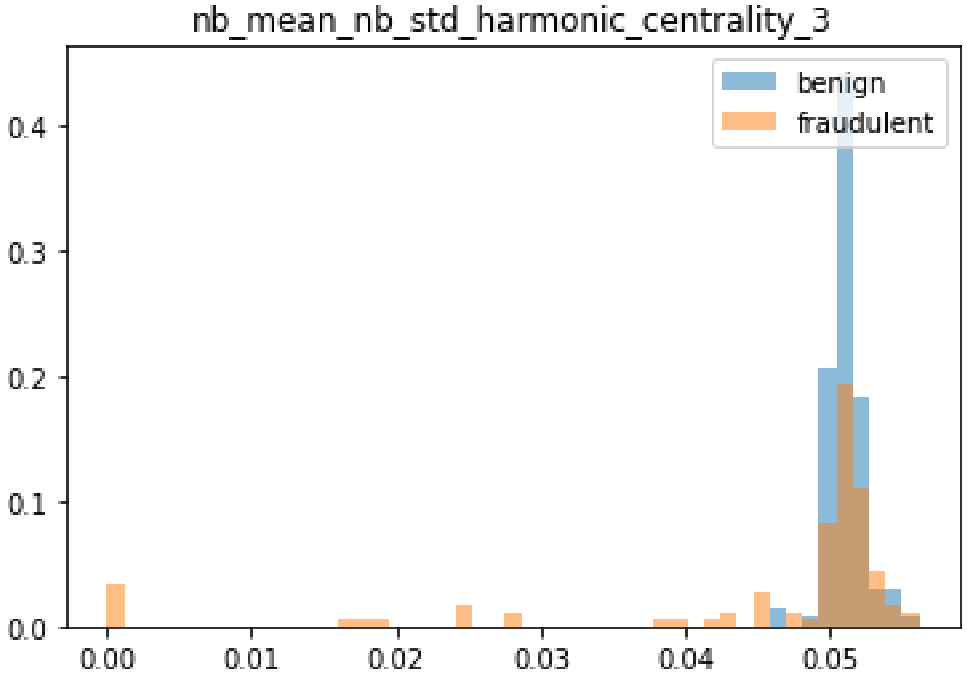
nb_mean_nb_std_harmonic_centrality_3
benign
fraudulent
0.4
0.3
0.2
0.1
0.0
0.00
0.01
0.02
0.03
0.04
0.05

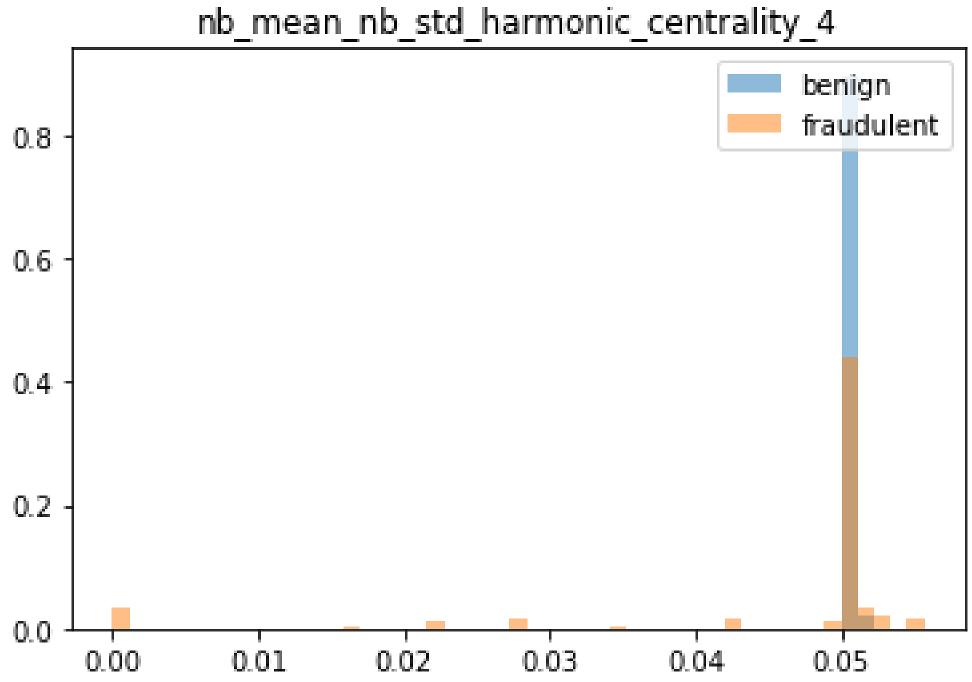
nb_mean_nb_std_harmonic_centrality_4
benign
fraudulent
0.8
0.6
0.4
0.2
0.0
0.00
0.01
0.02
0.03
0.04
0.05

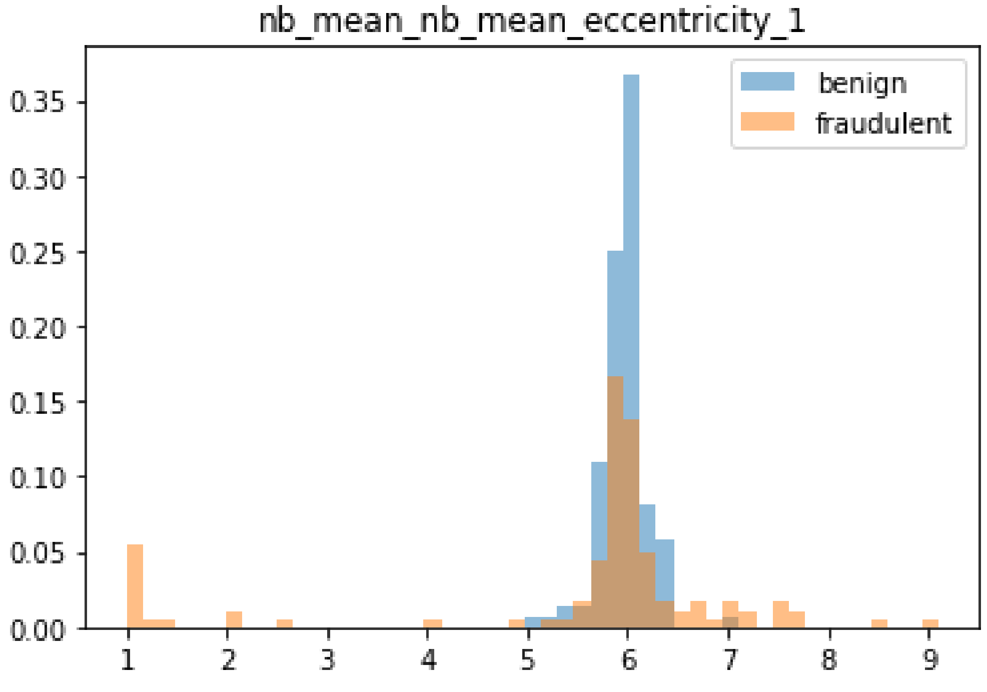
nb_mean_nb_mean_eccentricity_1
benign
fraudulent
0.35
0.30
0.25
0.20
0.15
0.10
0.05
0.00
1
2
3
4
5
6
7
8
9

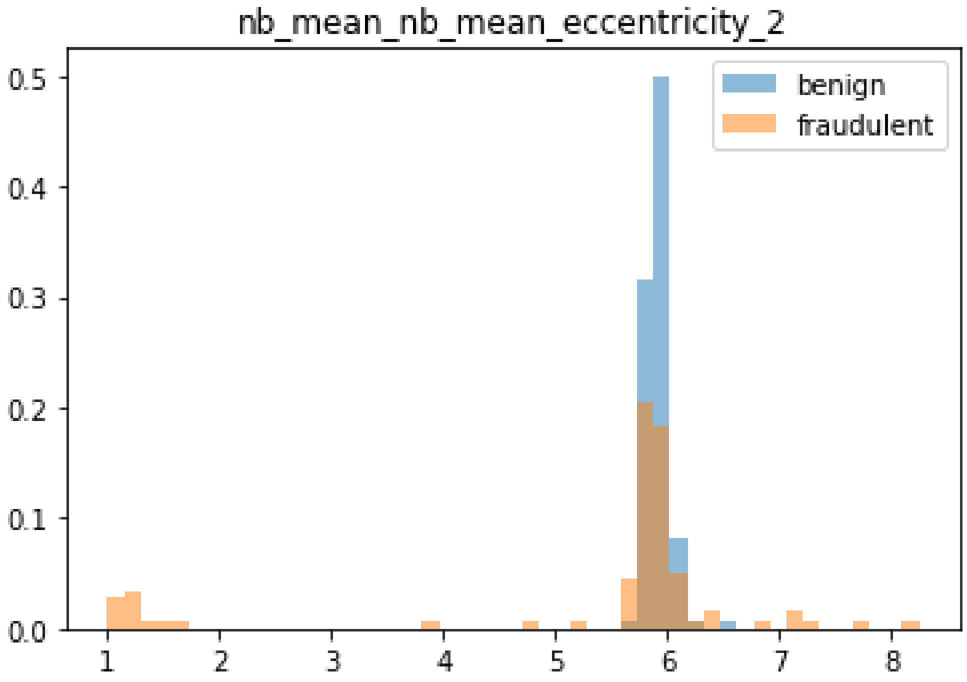
nb_mean_nb_mean_eccentricity_2
benign
fraudulent
0.0
0.1
0.2
0.3
0.4
0.5
1
2
3
4
5
6
7
8

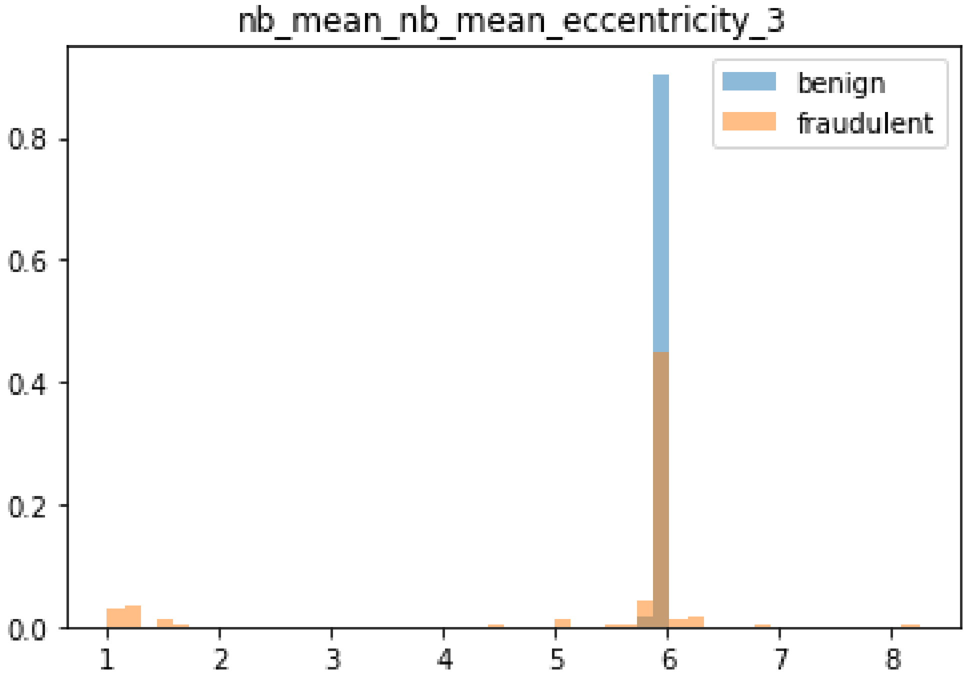
nb_mean_nb_mean_eccentricity_3
benign
fraudulent
0.0
0.2
0.4
0.6
0.8
1
2
3
4
5
6
7
8

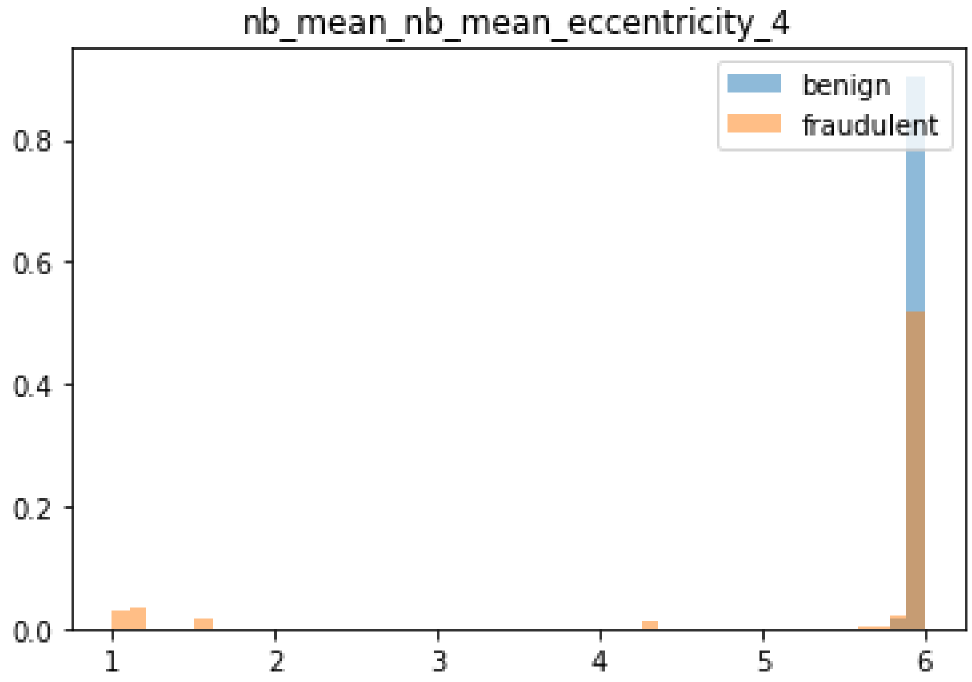
nb_mean_nb_mean_eccentricity_4
benign
fraudulent
0.0
0.2
0.4
0.6
0.8
1
2
3
4
5
6

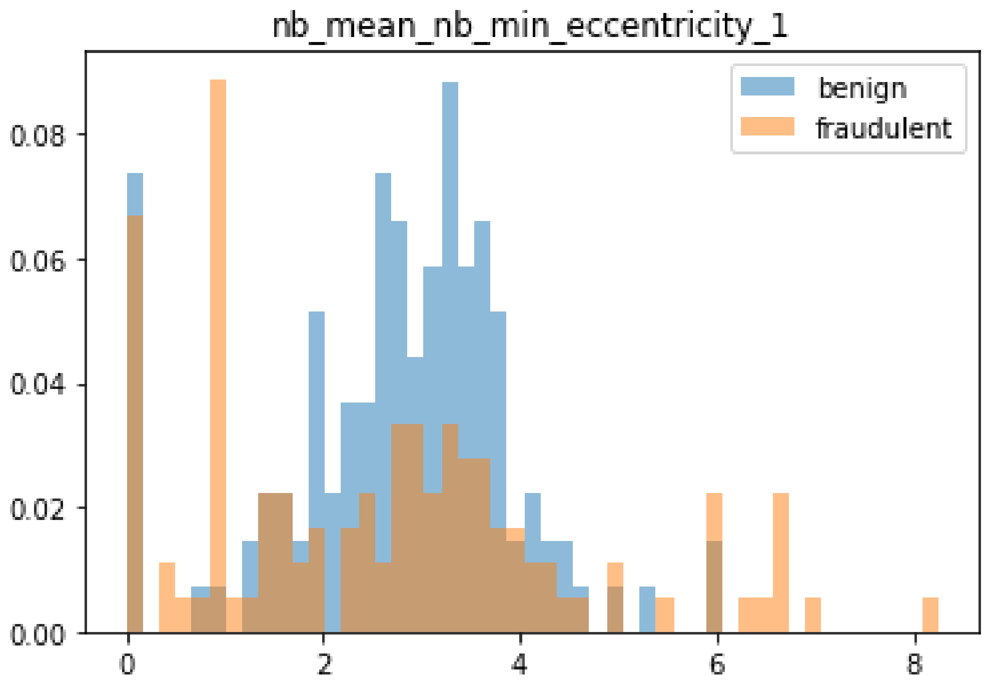
nb_mean_nb_min_eccentricity_1
benign
fraudulent
0.00
0.02
0.04
0.06
0.08
0
2
4
6
8

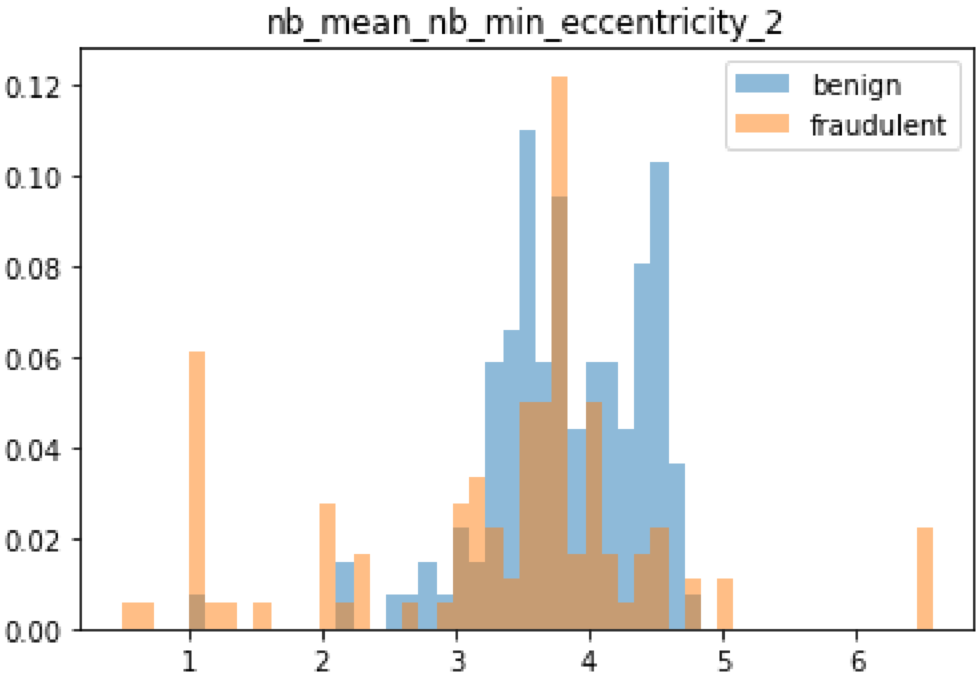
nb_mean_nb_min_eccentricity_2
benign
fraudulent
0.12
0.10
0.08
0.06
0.04
0.02
0.00
1
2
3
4
5
6

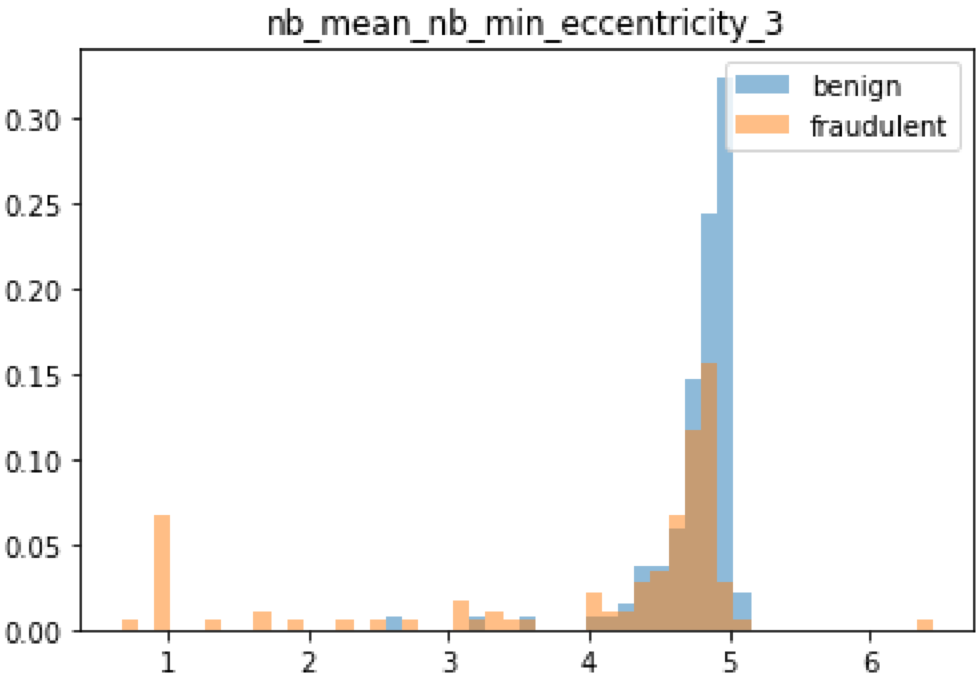
nb_mean_nb_min_eccentricity_3
benign
fraudulent
0.30
0.25
0.20
0.15
0.10
0.05
0.00
1
2
3
4
5
6

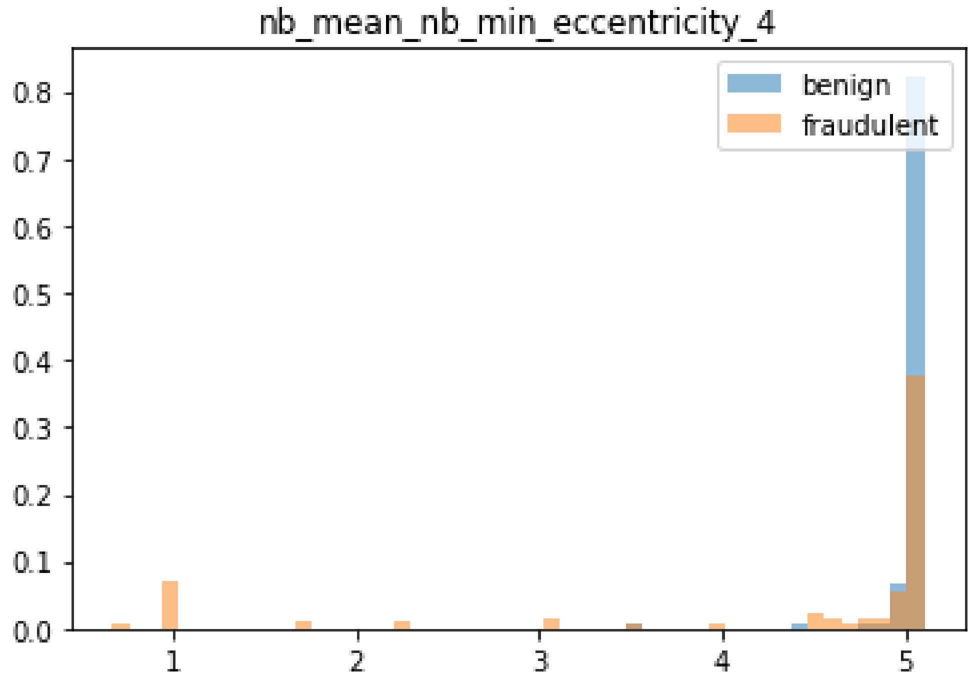
nb_mean_nb_min_eccentricity_4
benign
fraudulent
0.8
0.7
0.6
0.5
0.4
0.3
0.2
0.1
0.0
1
2
3
4
5

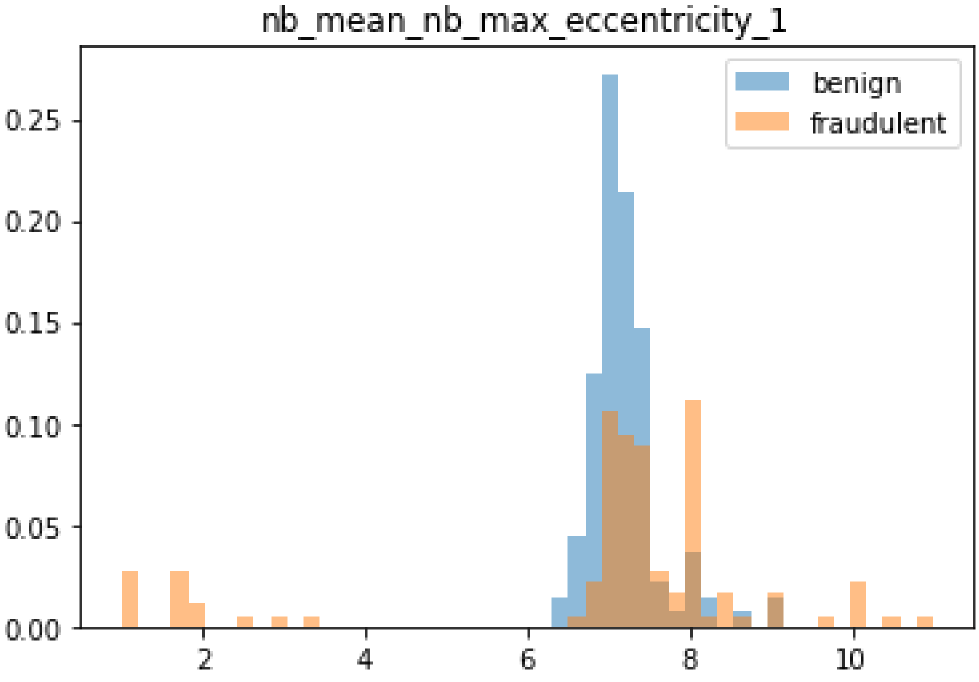
nb_mean_nb_max_eccentricity_1
benign
fraudulent
0.25
0.20
0.15
0.10
0.05
0.00
2
4
6
8
10

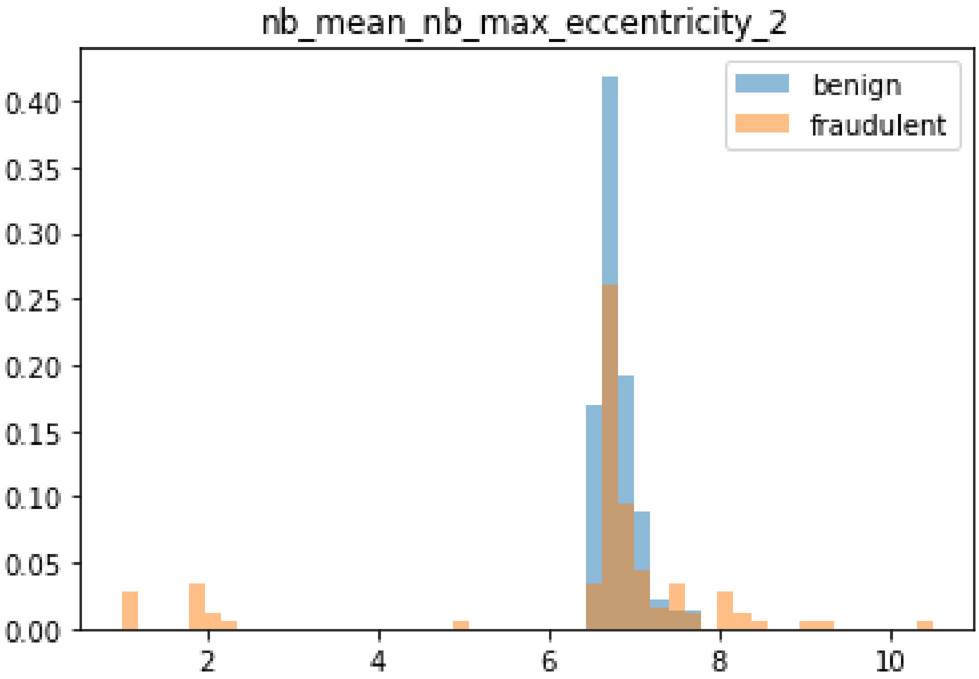
nb_mean_nb_max_eccentricity_2
benign
fraudulent
0.40
0.35
0.30
0.25
0.20
0.15
0.10
0.05
0.00
2
4
6
8
10

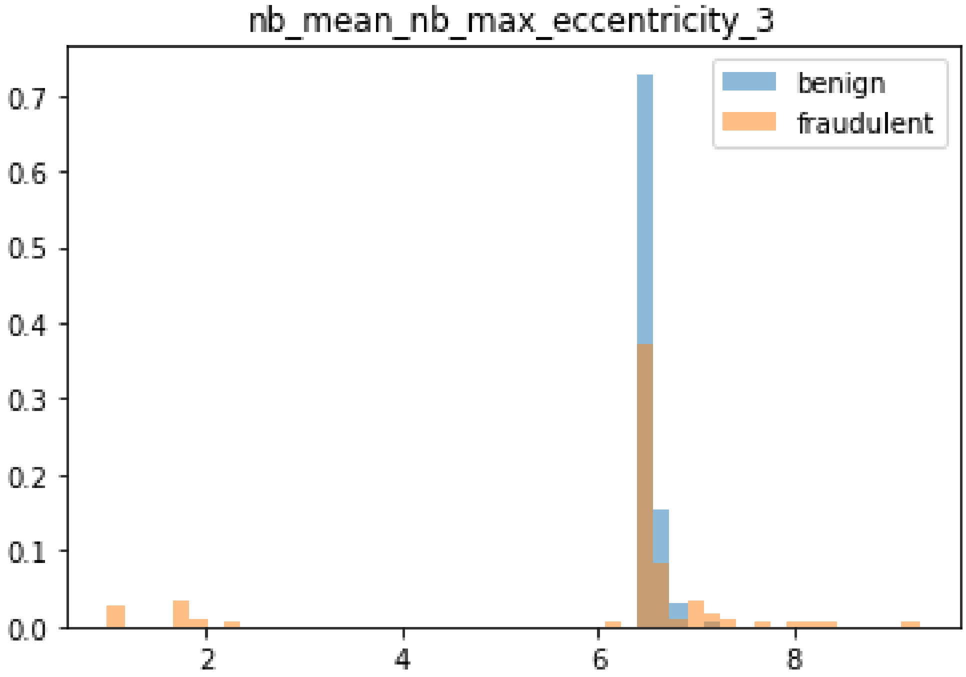
nb_mean_nb_max_eccentricity_3
benign
fraudulent
0.7
0.6
0.5
0.4
0.3
0.2
0.1
0.0
2
4
6
8

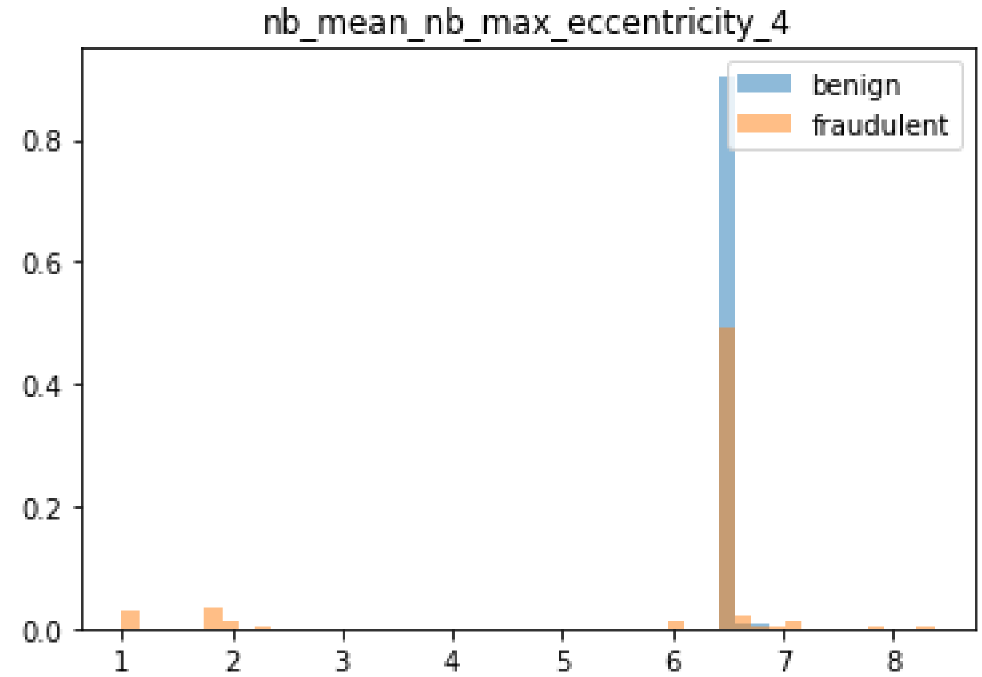
nb_mean_nb_max_eccentricity_4
benign
fraudulent
0.0
0.2
0.4
0.6
0.8
1
2
3
4
5
6
7
8

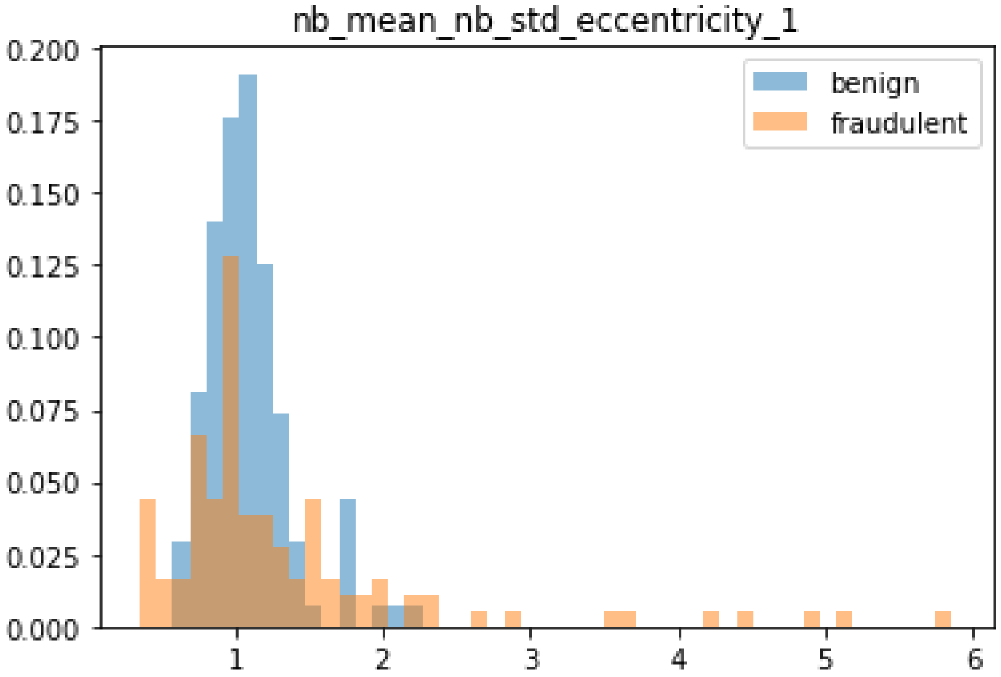
nb_mean_nb_std_eccentricity_1
benign
fraudulent
0.000
0.025
0.050
0.075
0.100
0.125
0.150
0.175
0.200
1
2
3
4
5
6

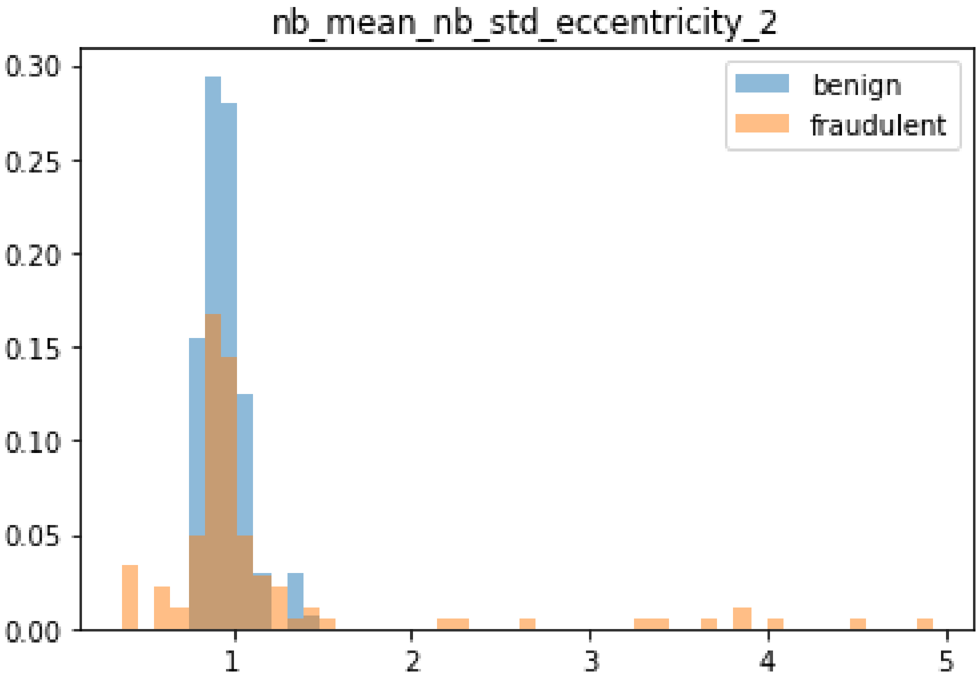
nb_mean_nb_std_eccentricity_2
benign
fraudulent
0.30
0.25
0.20
0.15
0.10
0.05
0.00
1
2
3
4
5

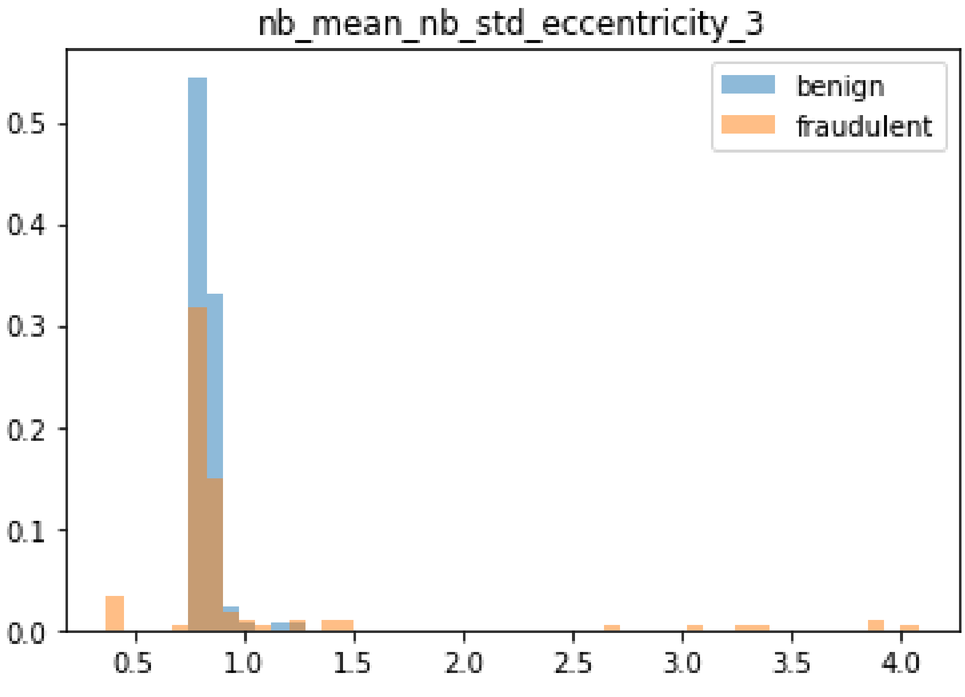
nb_mean_nb_std_eccentricity_3
benign
fraudulent
0.5
0.4
0.3
0.2
0.1
0.0
0.5
1.0
1.5
2.0
2.5
3.0
3.5
4.0

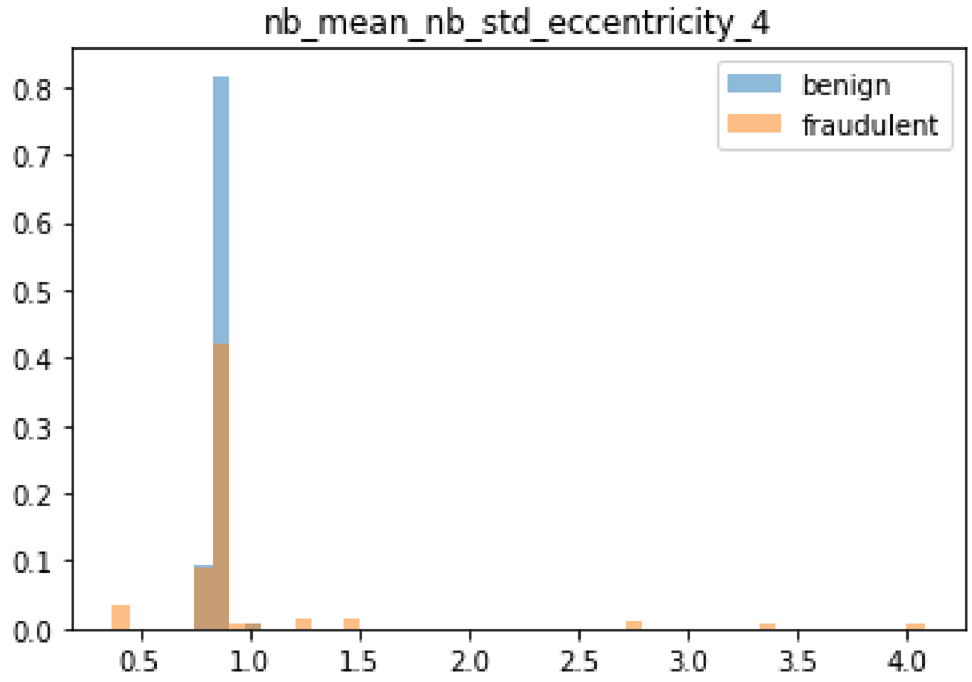
nb_mean_nb_std_eccentricity_4
benign
fraudulent
0.0
0.1
0.2
0.3
0.4
0.5
0.6
0.7
0.8
0.5
1.0
1.5
2.0
2.5
3.0
3.5
4.0

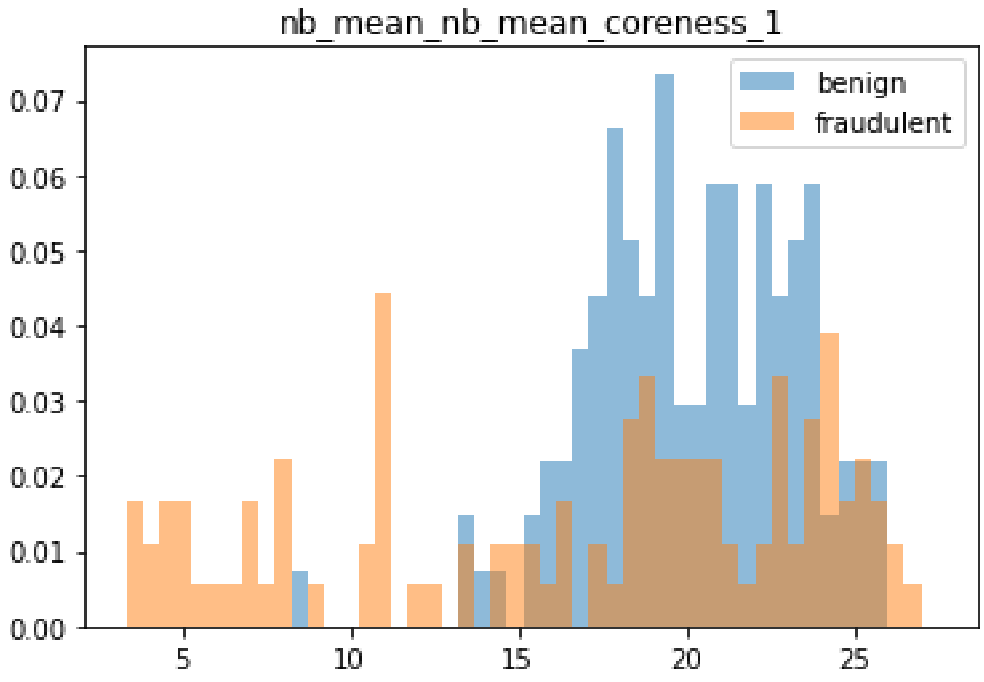
nb_mean_nb_mean_coreness_1
benign
fraudulent
0.00
0.01
0.02
0.03
0.04
0.05
0.06
0.07
5
10
15
20
25

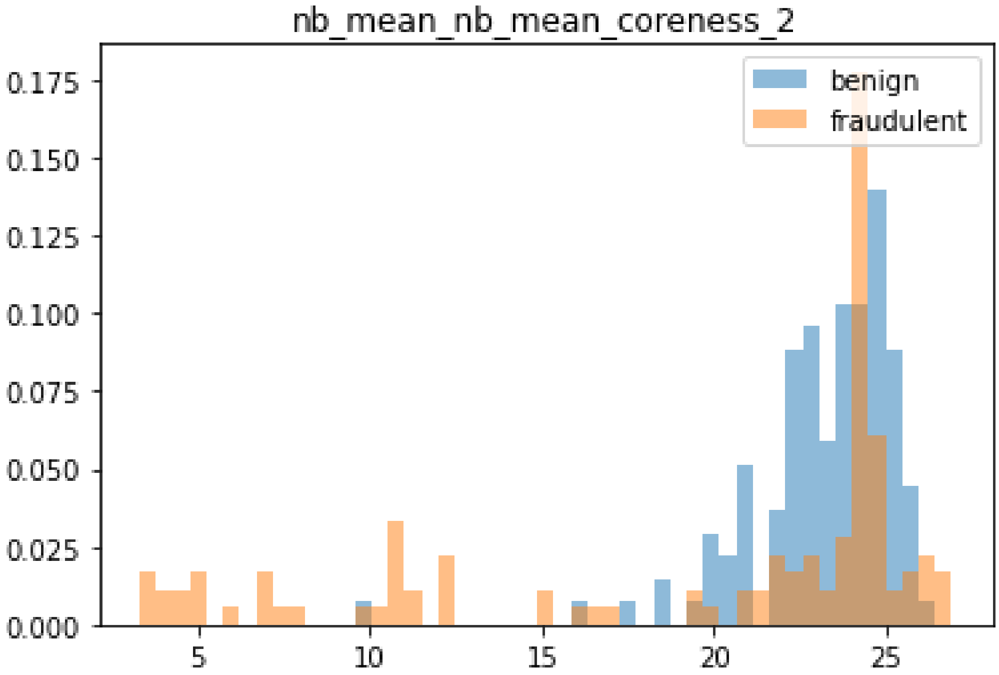
nb_mean_nb_mean_coreness_2
benign
fraudulent
0.175
0.150
0.125
0.100
0.075
0.050
0.025
0.000
5
10
15
20
25

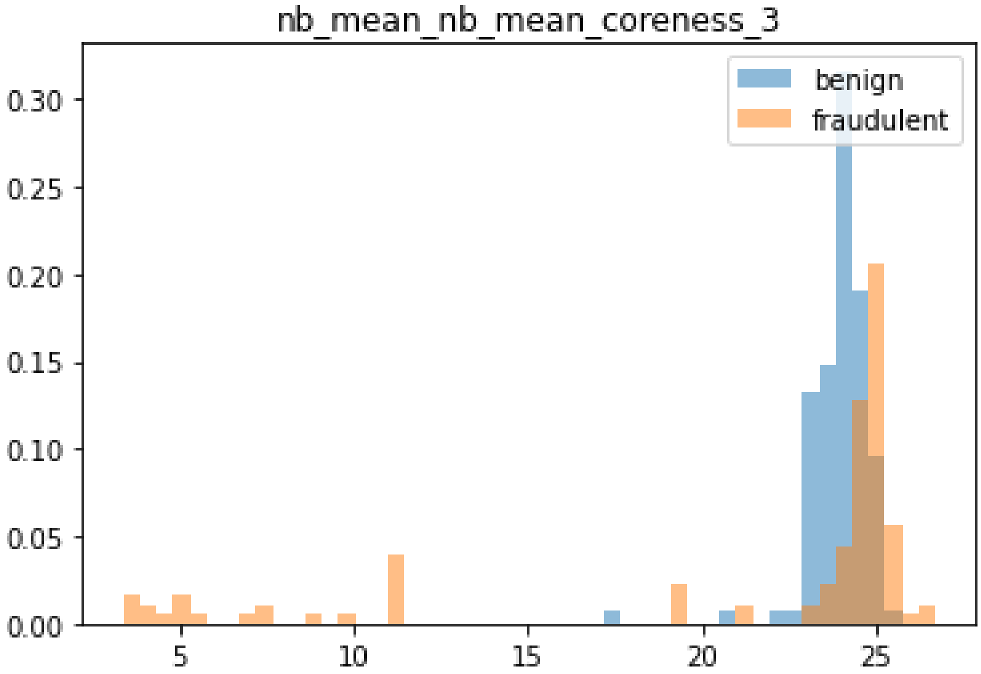
nb_mean_nb_mean_coreness_3
benign
fraudulent
0.30
0.25
0.20
0.15
0.10
0.05
0.00
5
10
15
20
25

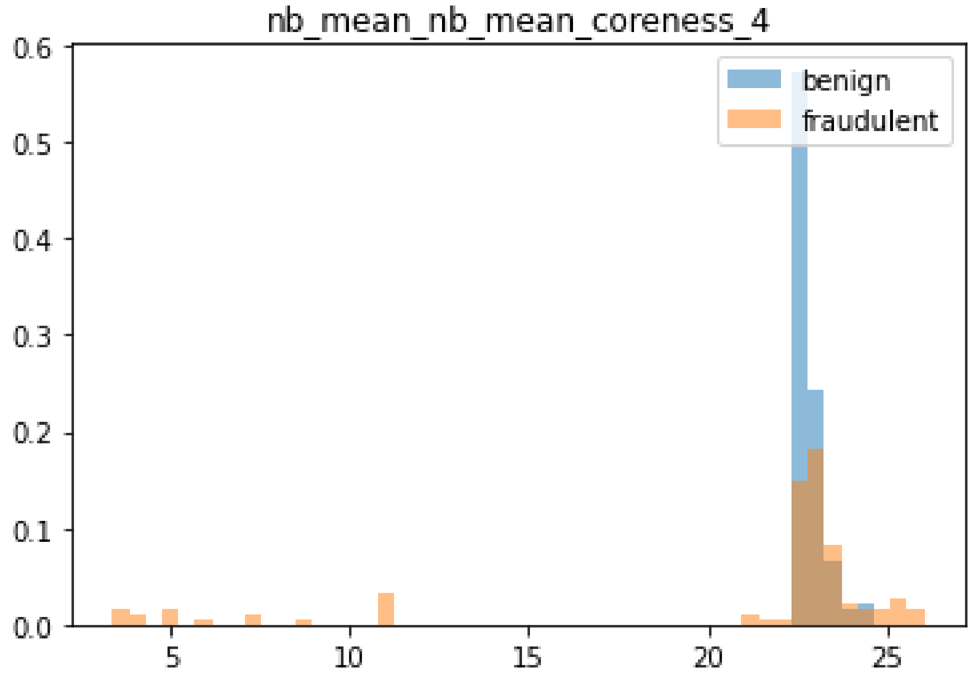
nb_mean_nb_mean_coreness_4
benign
fraudulent
0.0
0.1
0.2
0.3
0.4
0.5
0.6
5
10
15
20
25

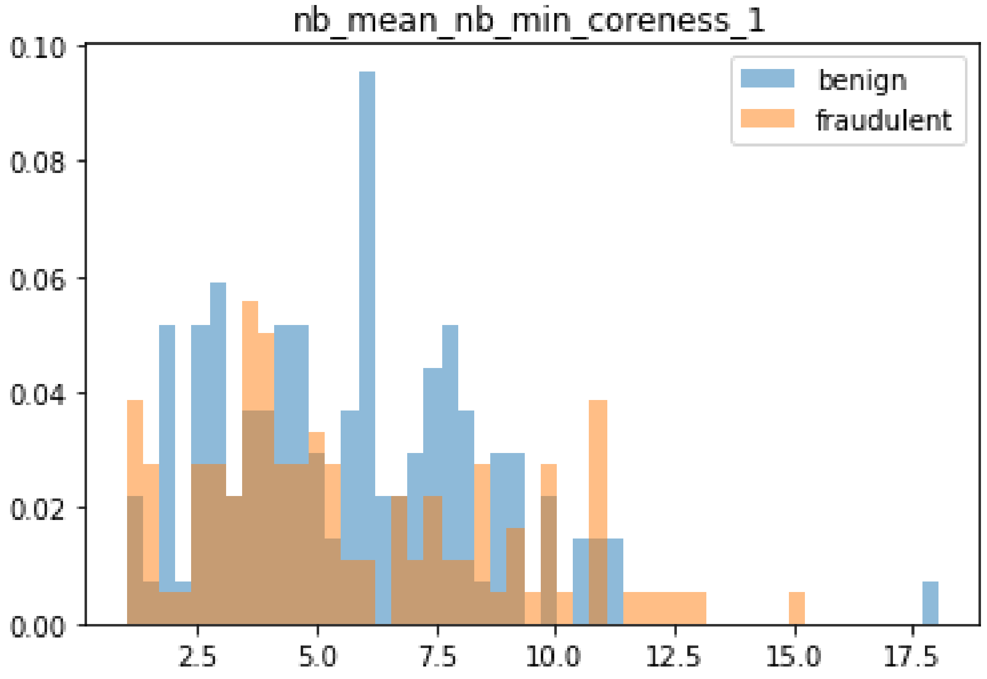
nb_mean_nb_min_coreness_1
benign
fraudulent
0.00
0.02
0.04
0.06
0.08
0.10
2.5
5.0
7.5
10.0
12.5
15.0
17.5

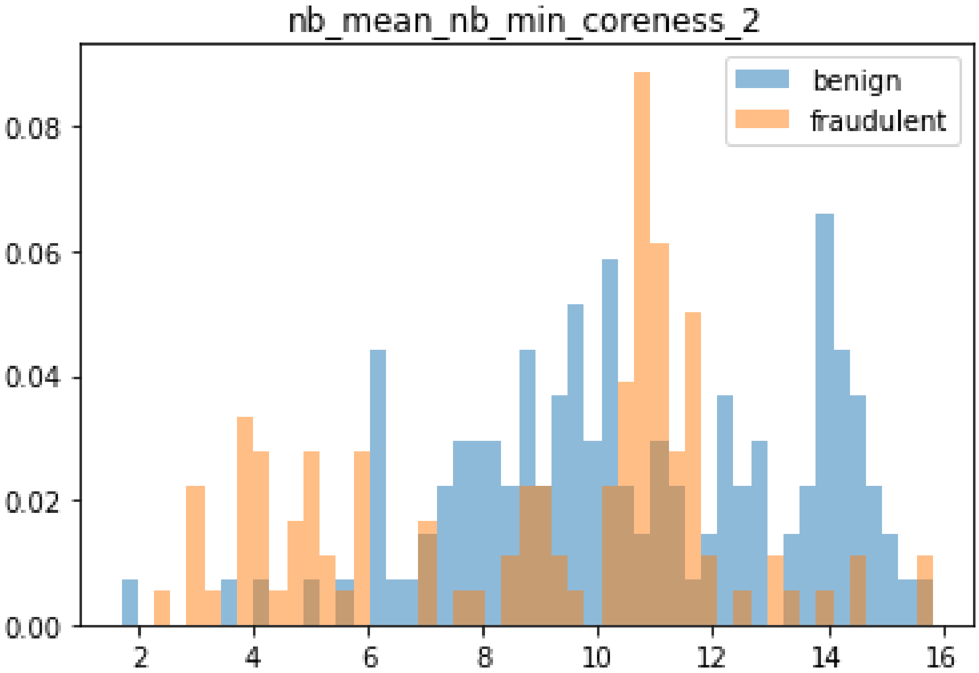
nb_mean_nb_min_coreness_2
benign
fraudulent
0.00
0.02
0.04
0.06
0.08
2
4
6
8
10
12
14
16

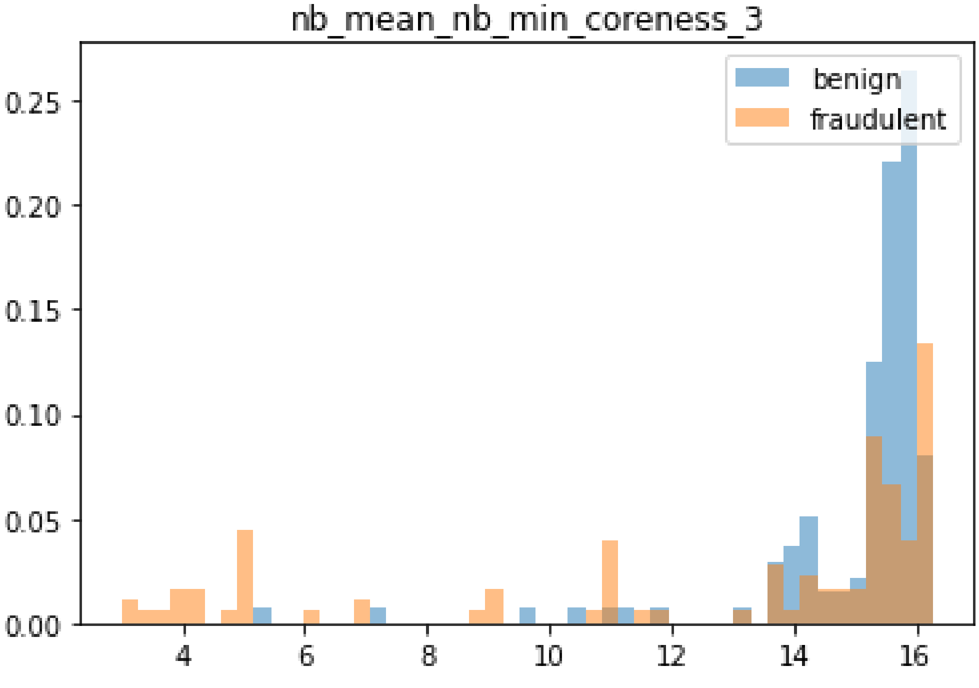
nb_mean_nb_min_coreness_3
benign
fraudulent
0.00
0.05
0.10
0.15
0.20
0.25
4
6
8
10
12
14
16

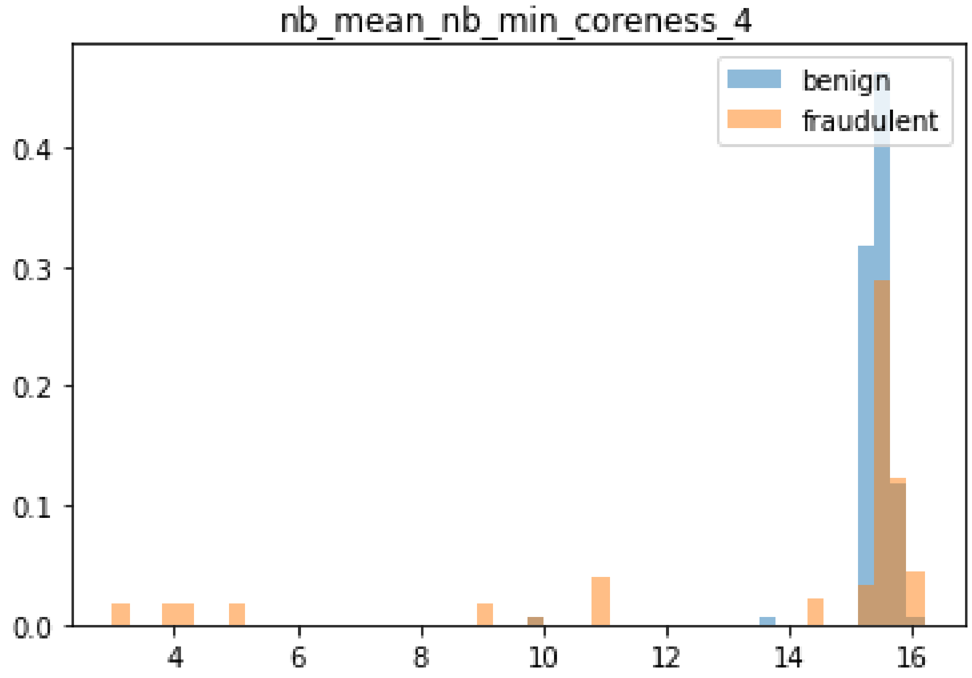
nb_mean_nb_min_coreness_4
benign
fraudulent
0.0
0.1
0.2
0.3
0.4
4
6
8
10
12
14
16

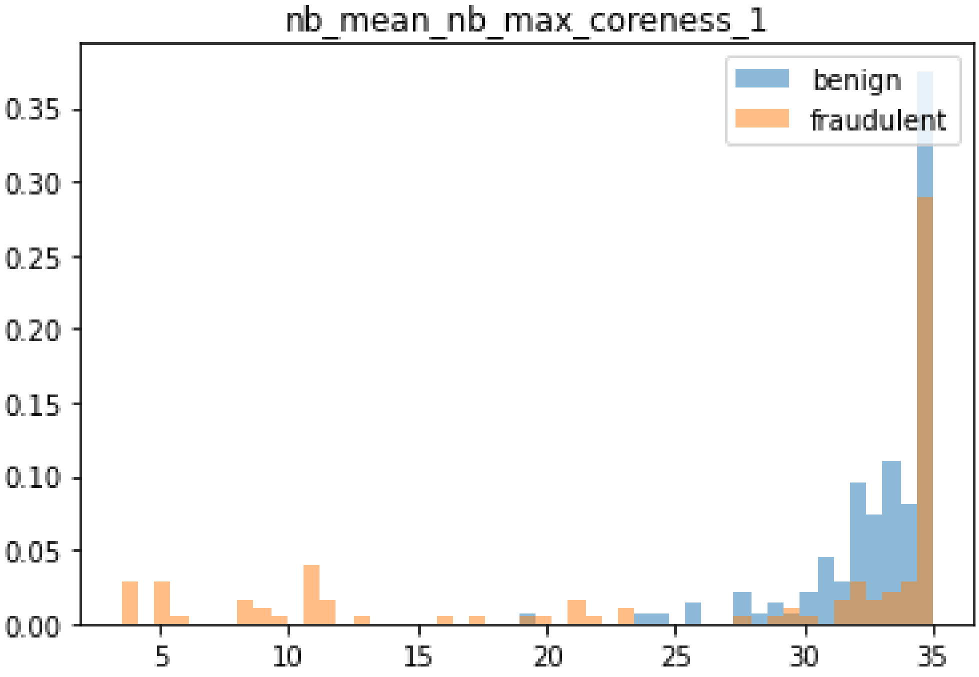
nb_mean_nb_max_coreness_1
benign
fraudulent
0.00
0.05
0.10
0.15
0.20
0.25
0.30
0.35
5
10
15
20
25
30
35

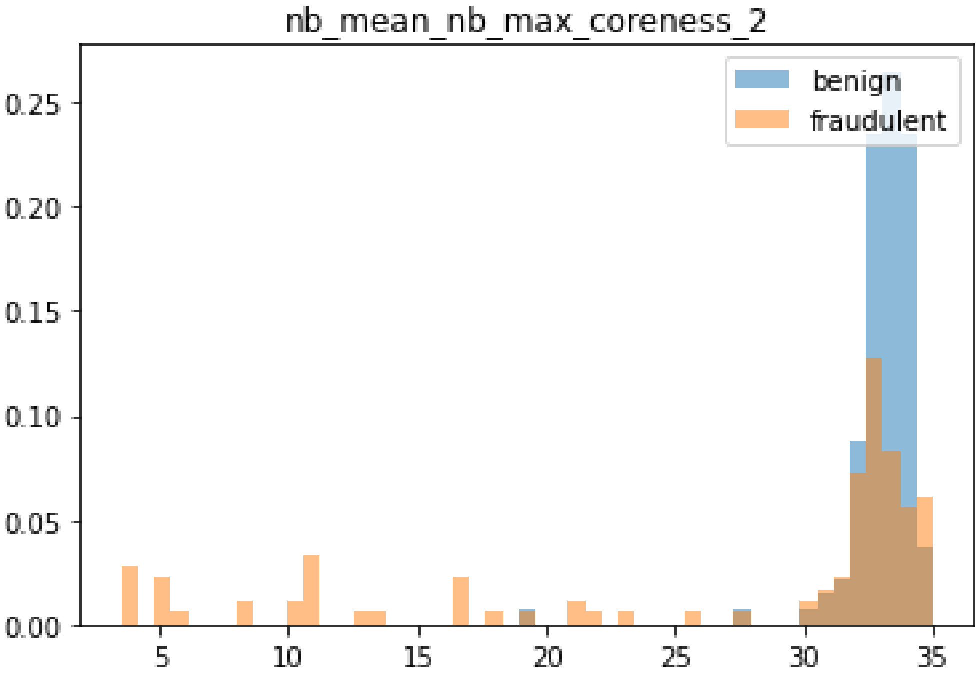
nb_mean_nb_max_coreness_2
benign
fraudulent
0.25
0.20
0.15
0.10
0.05
0.00
5
10
15
20
25
30
35

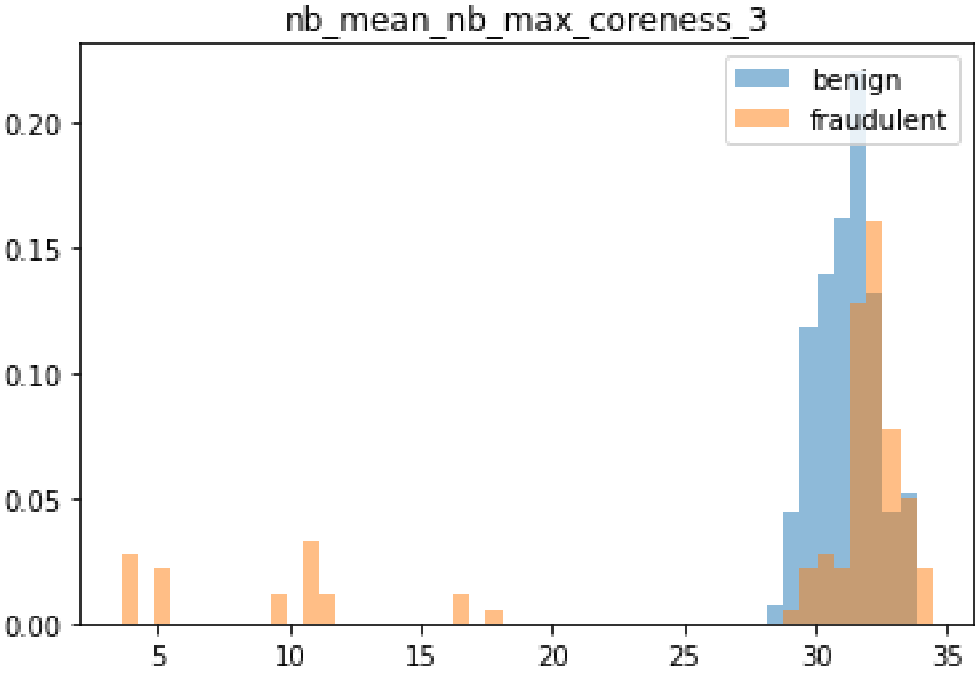
nb_mean_nb_max_coreness_3
benign
fraudulent
0.20
0.15
0.10
0.05
0.00
5
10
15
20
25
30
35

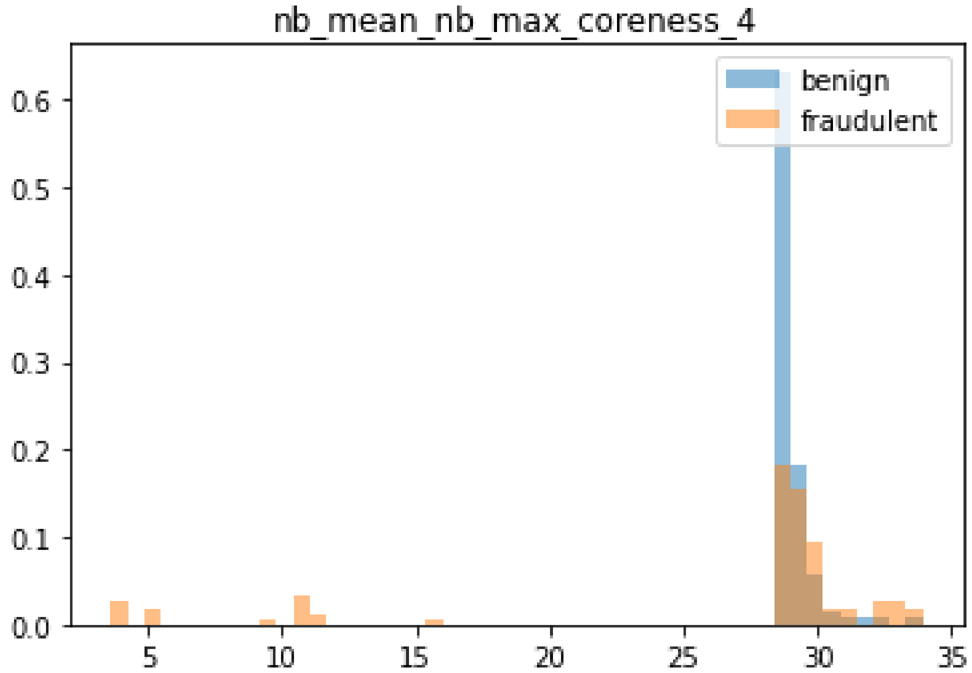
nb_mean_nb_max_coreness_4
benign
fraudulent
0.0
0.1
0.2
0.3
0.4
0.5
0.6
5
10
15
20
25
30
35

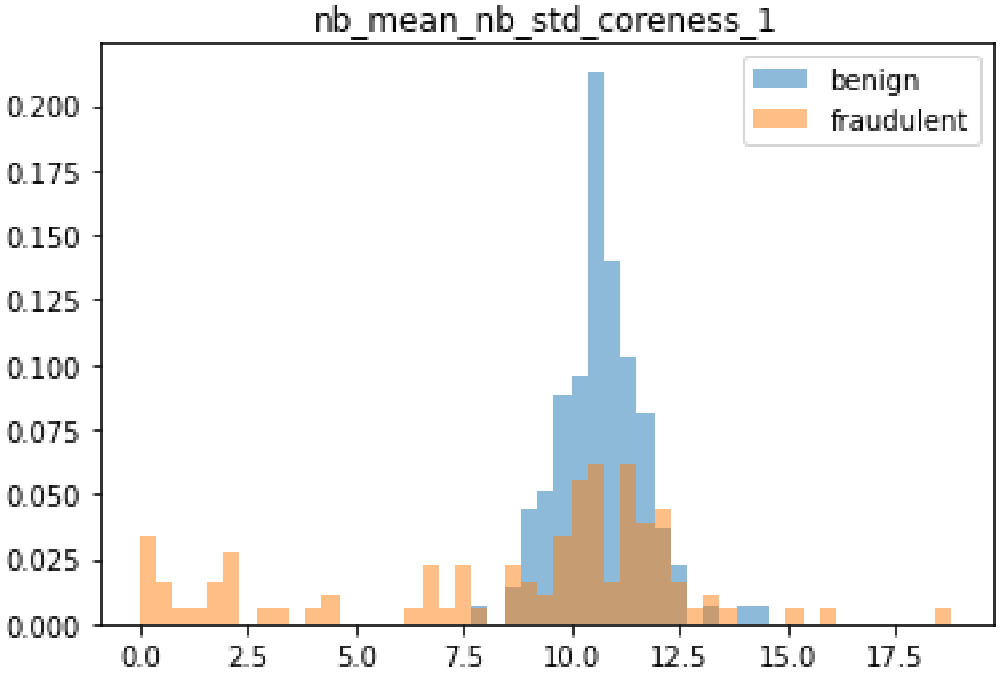
nb_mean_nb_std_coreness_1
benign
fraudulent
0.000
0.025
0.050
0.075
0.100
0.125
0.150
0.175
0.200
0.0
2.5
5.0
7.5
10.0
12.5
15.0
17.5

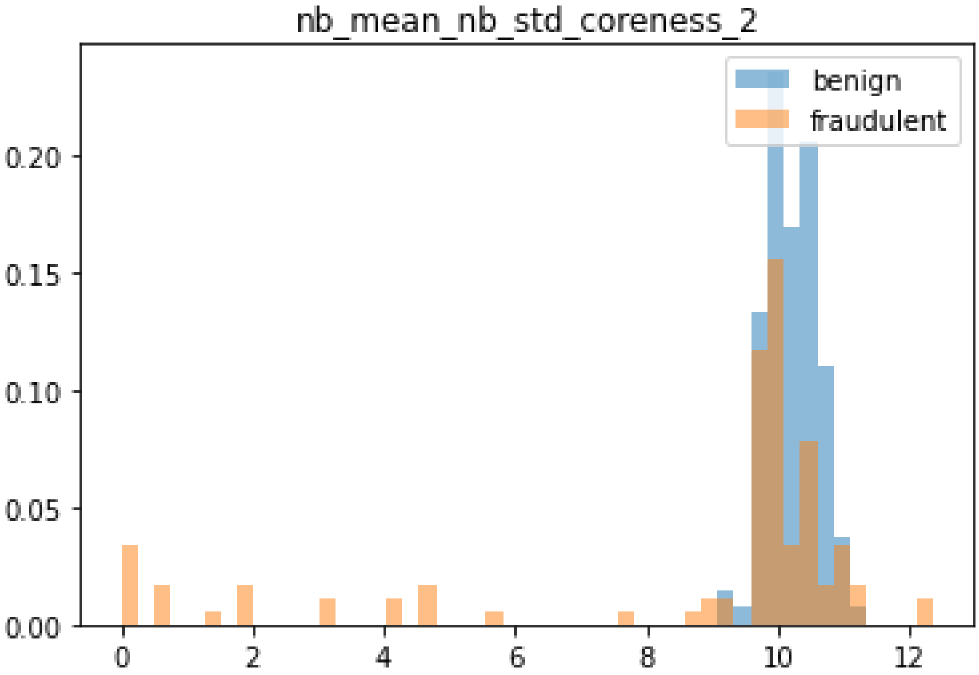
nb_mean_nb_std_coreness_2
benign
fraudulent
0.20
0.15
0.10
0.05
0.00
0
2
4
6
8
10
12

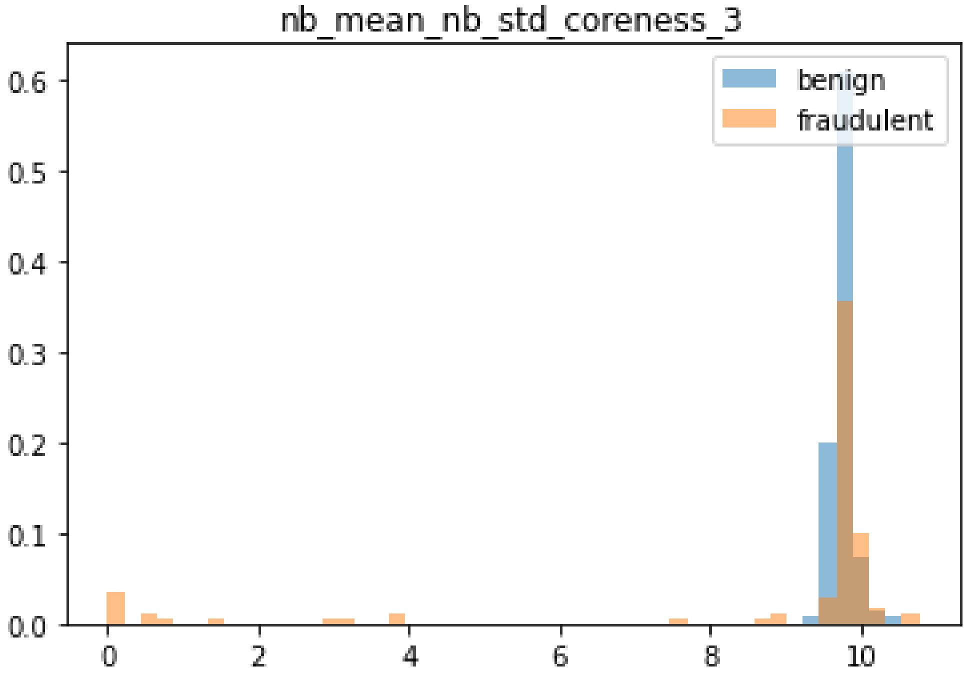
nb_mean_nb_std_coreness_3
benign
fraudulent
0.6
0.5
0.4
0.3
0.2
0.1
0.0
0
2
4
6
8
10

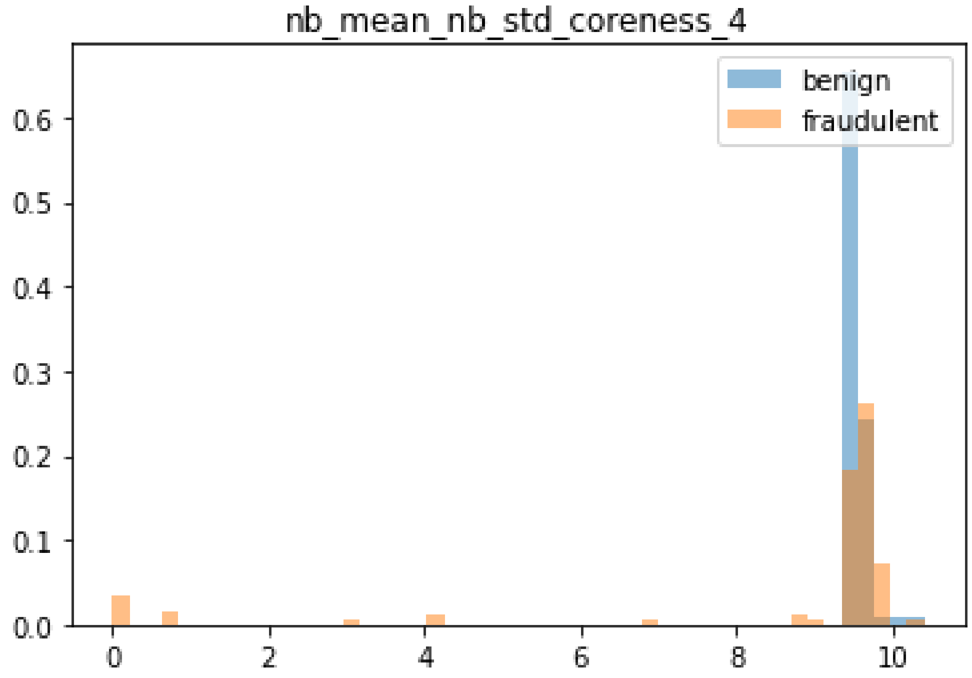
nb_mean_nb_std_coreness_4
benign
fraudulent
0.6
0.5
0.4
0.3
0.2
0.1
0.0
0
2
4
6
8
10

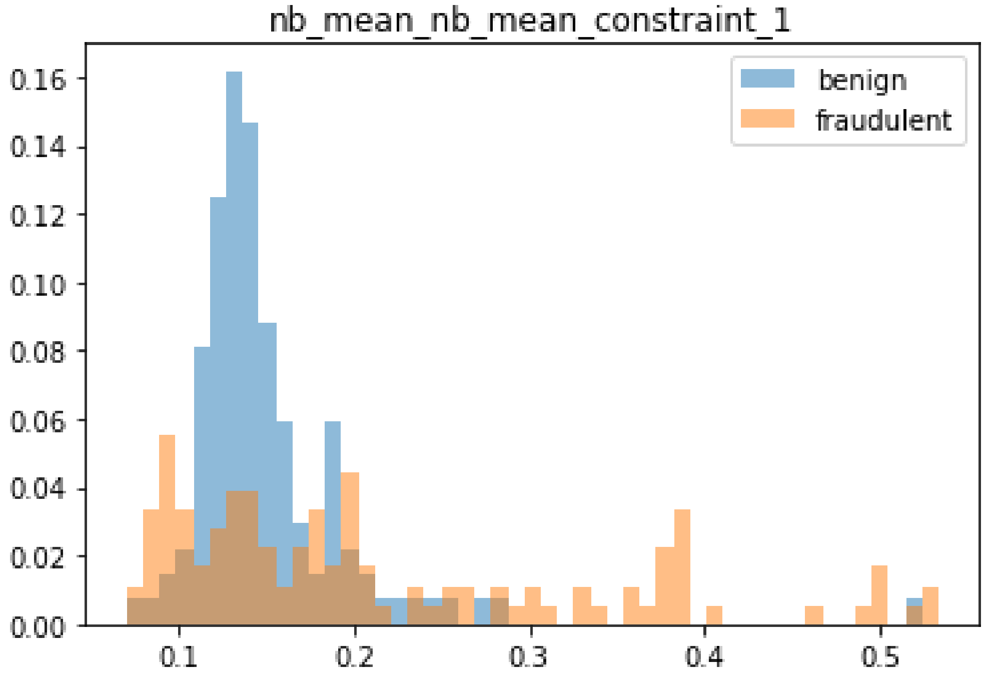
nb_mean_nb_mean_constraint_1
benign
fraudulent
0.16
0.14
0.12
0.10
0.08
0.06
0.04
0.02
0.00
0.1
0.2
0.3
0.4
0.5

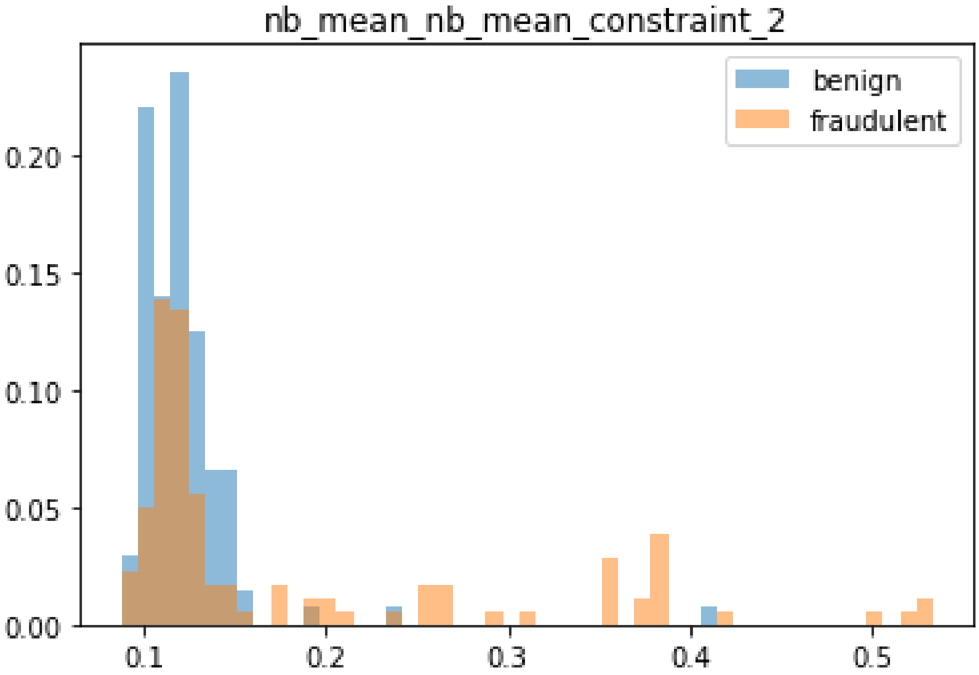
nb_mean_nb_mean_constraint_2
benign
fraudulent
0.00
0.05
0.10
0.15
0.20
0.1
0.2
0.3
0.4
0.5

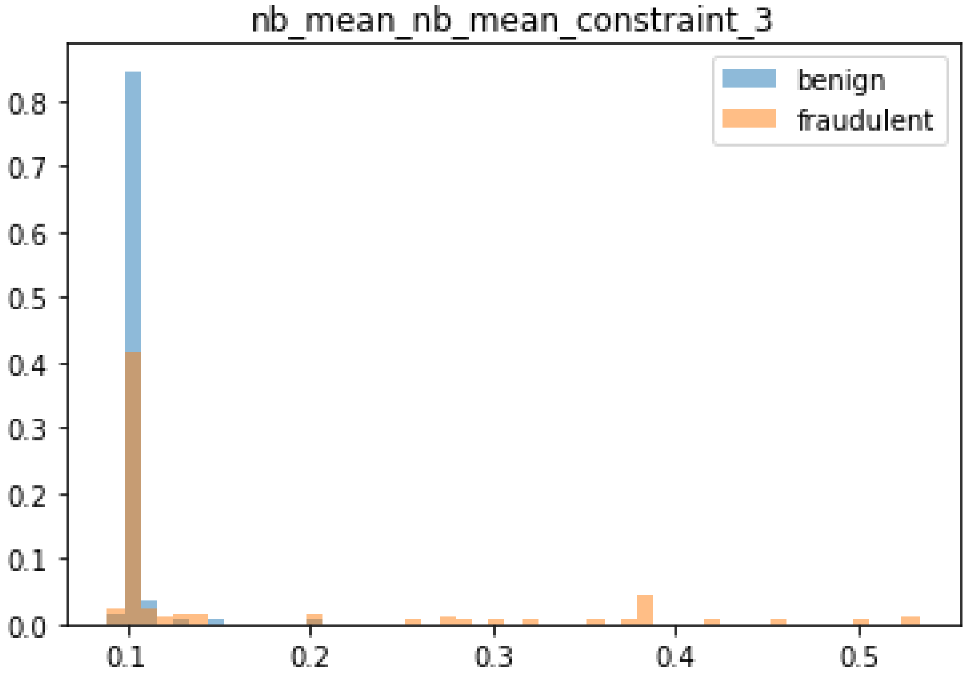
nb_mean_nb_mean_constraint_3
benign
fraudulent
0.0
0.1
0.2
0.3
0.4
0.5
0.6
0.7
0.8
0.1
0.2
0.3
0.4
0.5

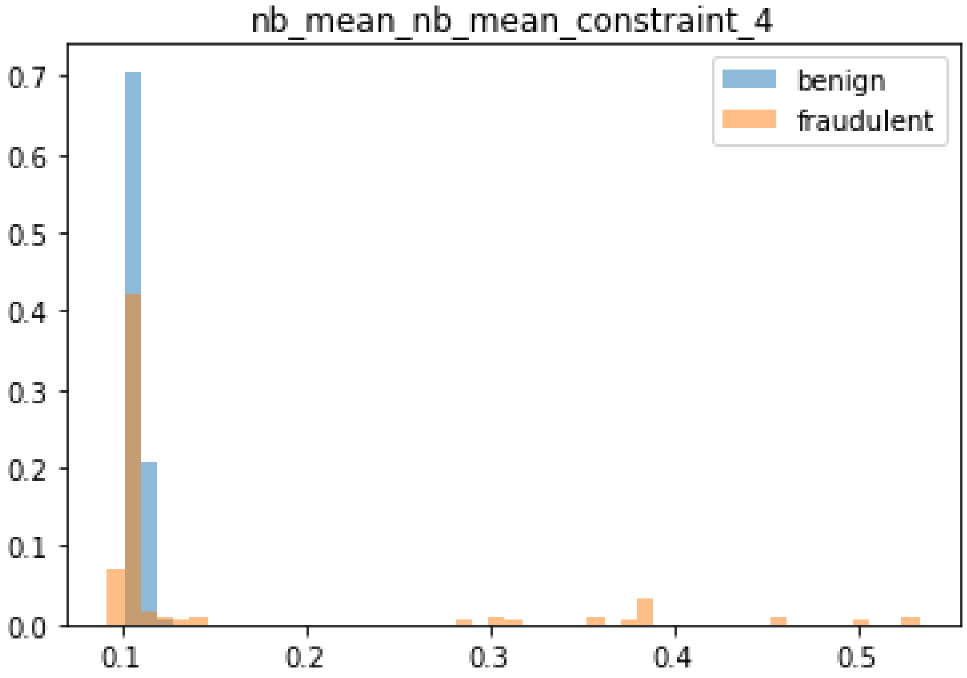
nb_mean_nb_mean_constraint_4
benign
fraudulent
0.0
0.1
0.2
0.3
0.4
0.5
0.6
0.7
0.1
0.2
0.3
0.4
0.5

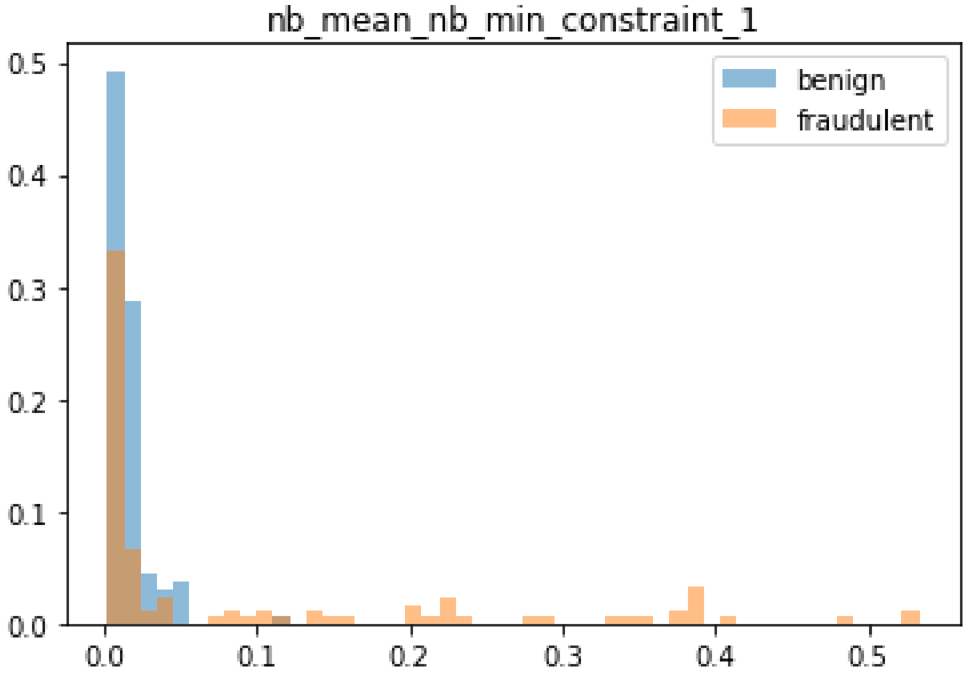
nb_mean_nb_min_constraint_1
benign
fraudulent
0.0
0.1
0.2
0.3
0.4
0.5
0.0
0.1
0.2
0.3
0.4
0.5

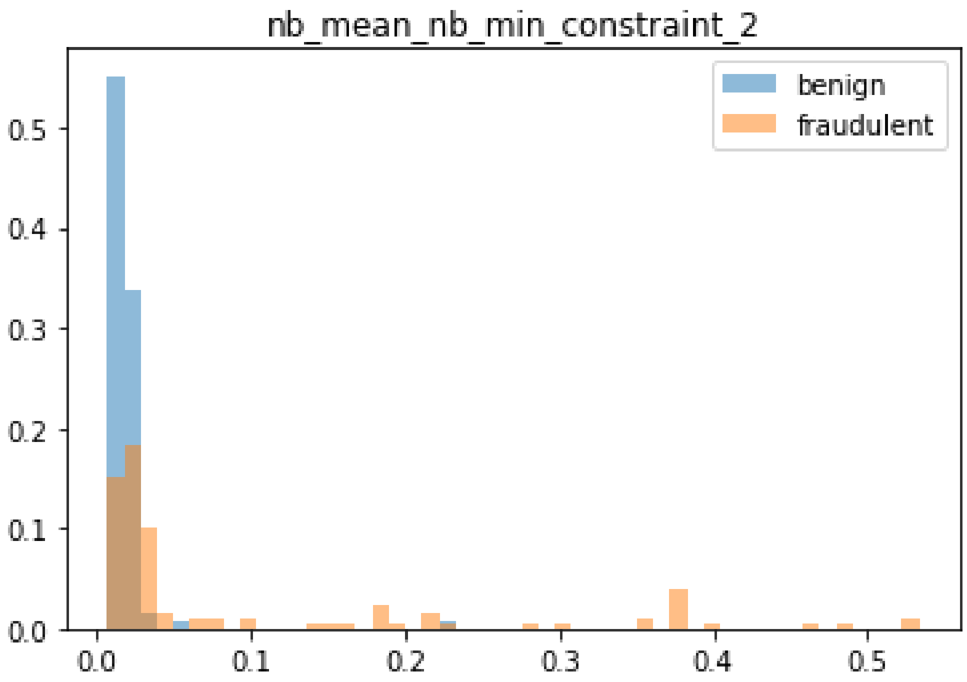
nb_mean_nb_min_constraint_2
benign
fraudulent
0.5
0.4
0.3
0.2
0.1
0.0
0.0
0.1
0.2
0.3
0.4
0.5

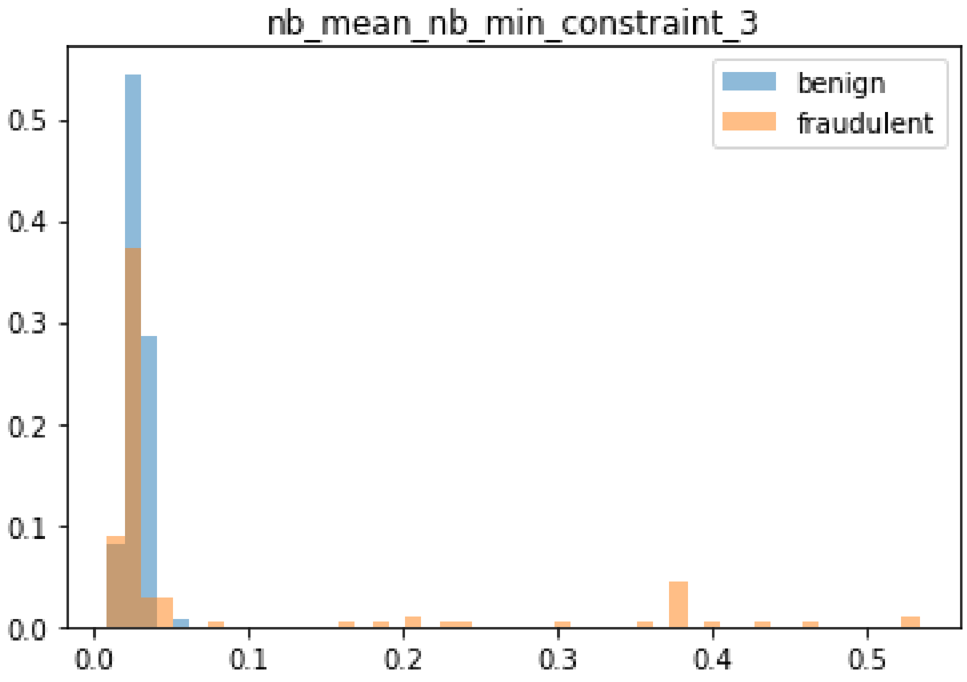
nb_mean_nb_min_constraint_3
benign
fraudulent
0.5
0.4
0.3
0.2
0.1
0.0
0.0
0.1
0.2
0.3
0.4
0.5

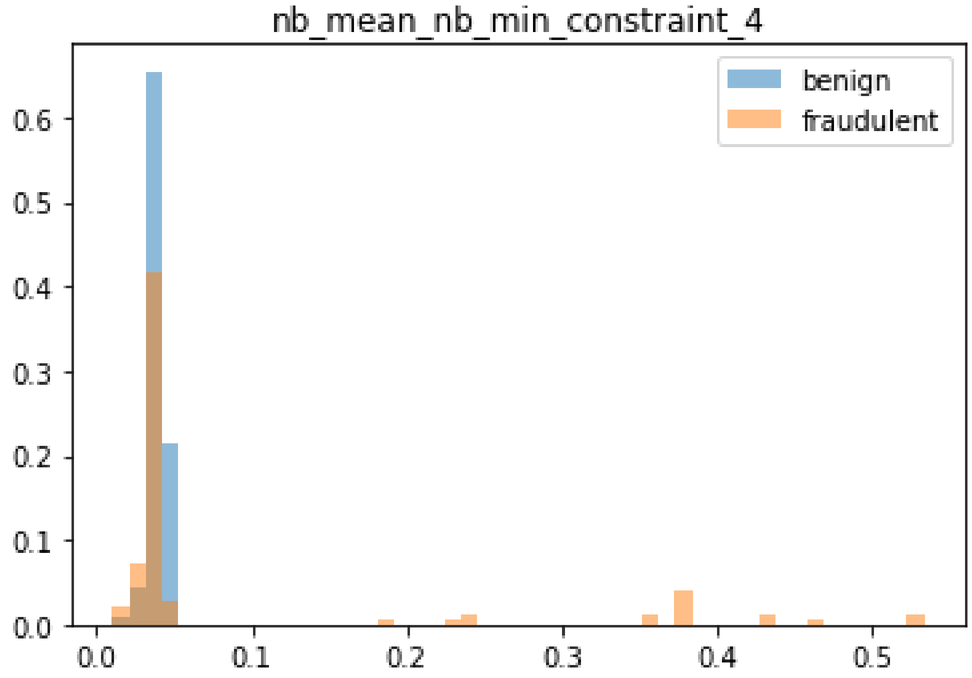
nb_mean_nb_min_constraint_4
benign
fraudulent
0.0
0.1
0.2
0.3
0.4
0.5
0.6

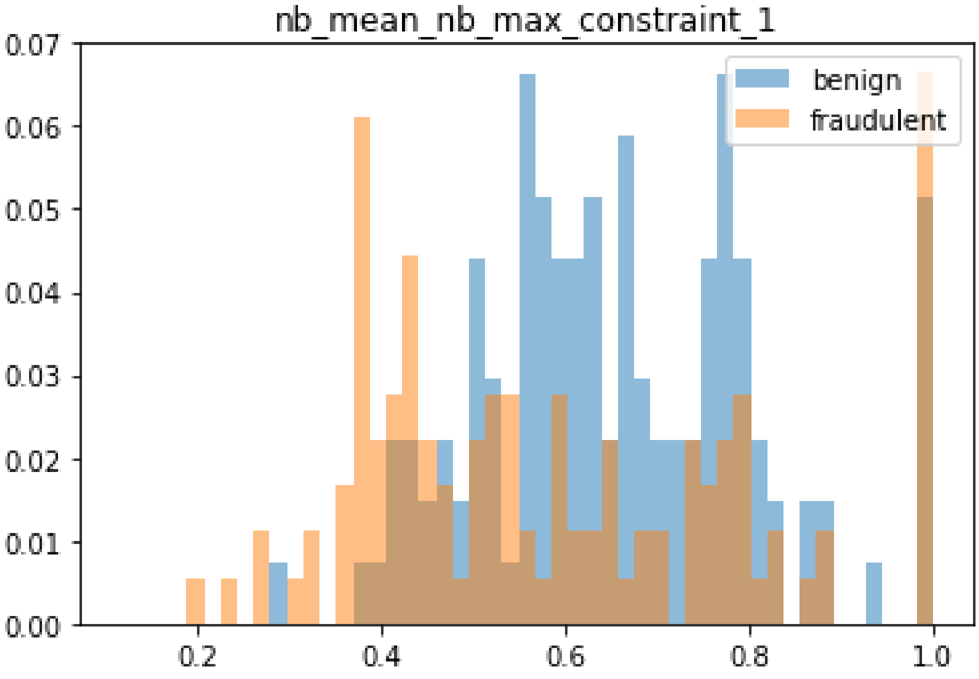
nb_mean_nb_max_constraint_1
benign
fraudulent
0.00
0.01
0.02
0.03
0.04
0.05
0.06
0.07
0.2
0.4
0.6
0.8
1.0

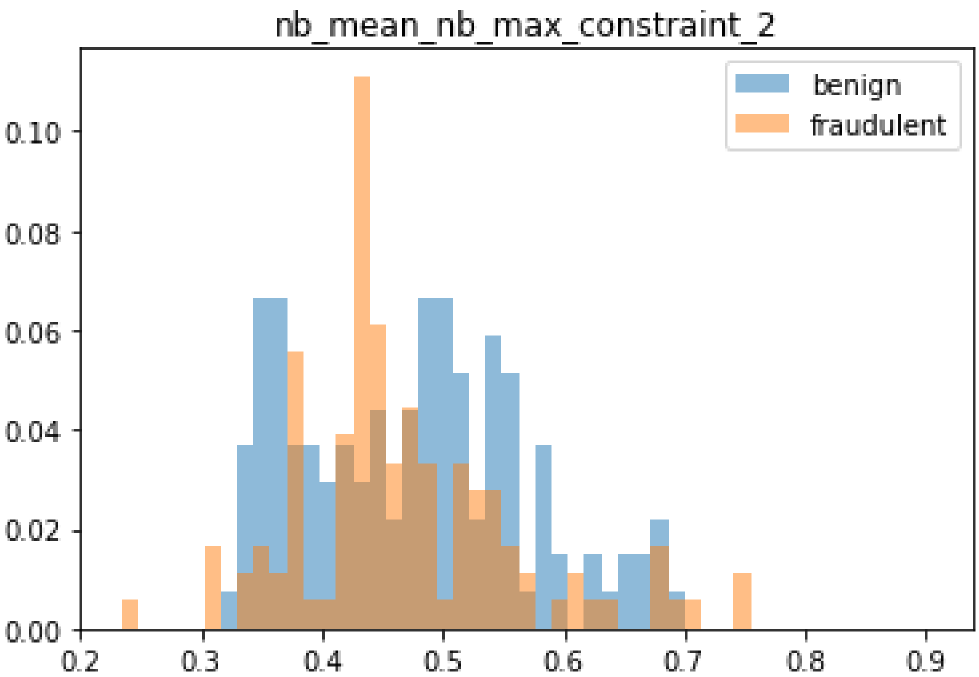
nb_mean_nb_max_constraint_2
benign
fraudulent
0.00
0.02
0.04
0.06
0.08
0.10
0.2
0.3
0.4
0.5
0.6
0.7
0.8
0.9

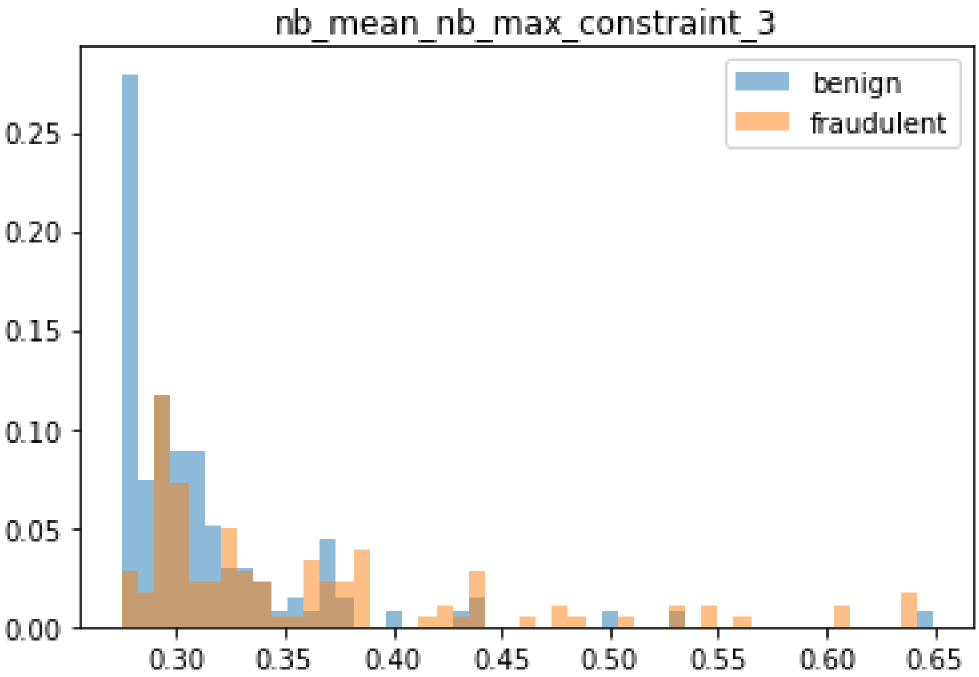
nb_mean_nb_max_constraint_3
benign
fraudulent
0.00
0.05
0.10
0.15
0.20
0.25
0.30
0.35
0.40
0.45
0.50
0.55
0.60
0.65

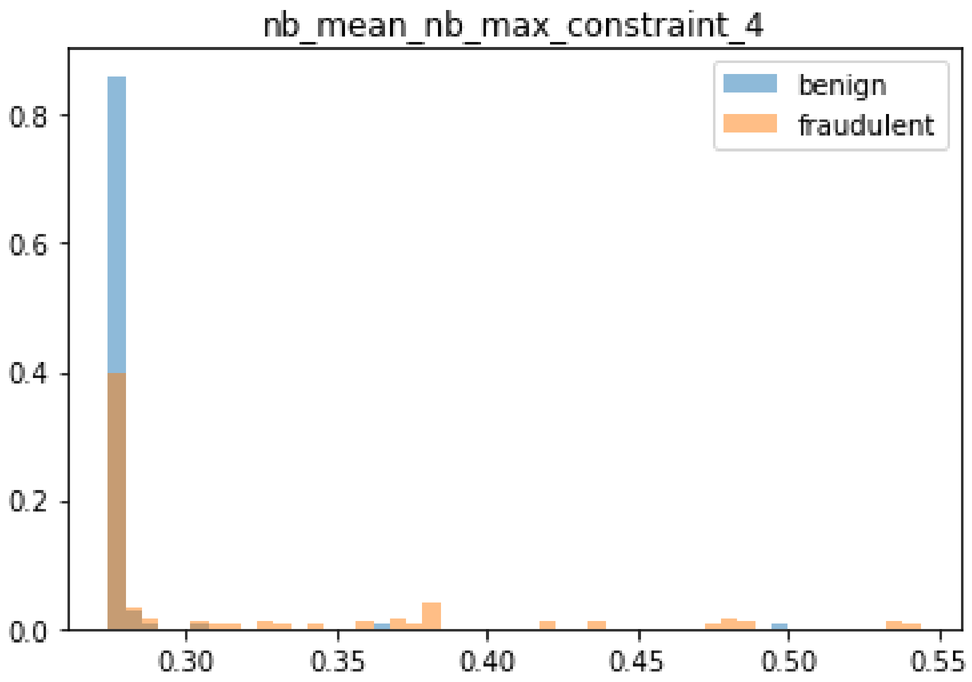

nb_mean_nb_max_constraint_4
benign
fraudulent
0.8
0.6
0.4
0.2
0.0
0.30
0.35
0.40
0.45
0.50
0.55

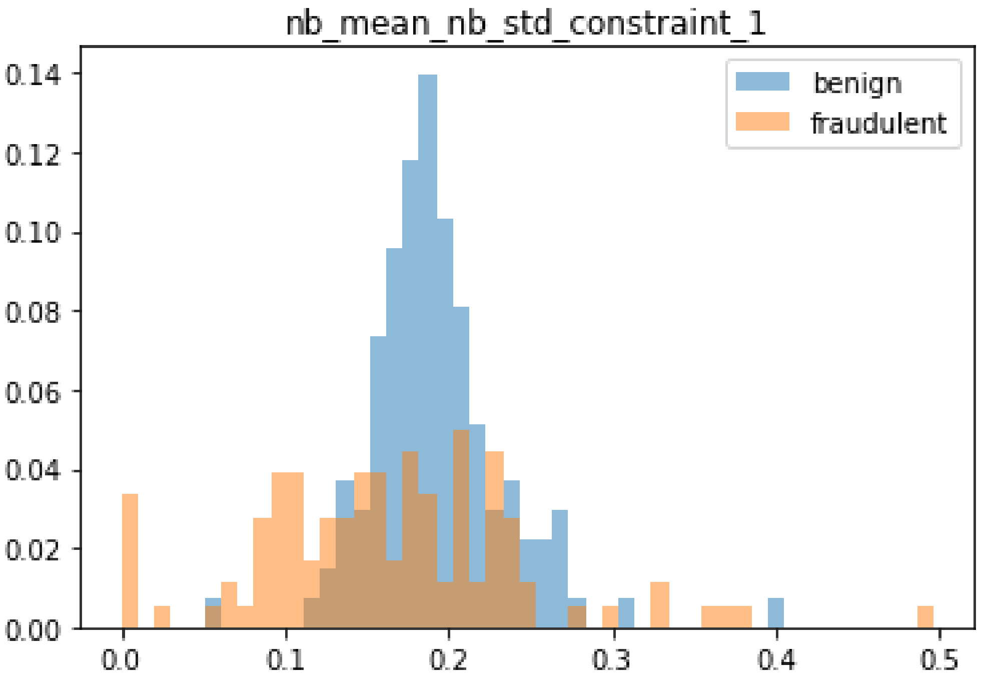

nb_mean_nb_std_constraint_1
benign
fraudulent
0.14
0.12
0.10
0.08
0.06
0.04
0.02
0.00
0.0
0.1
0.2
0.3
0.4
0.5

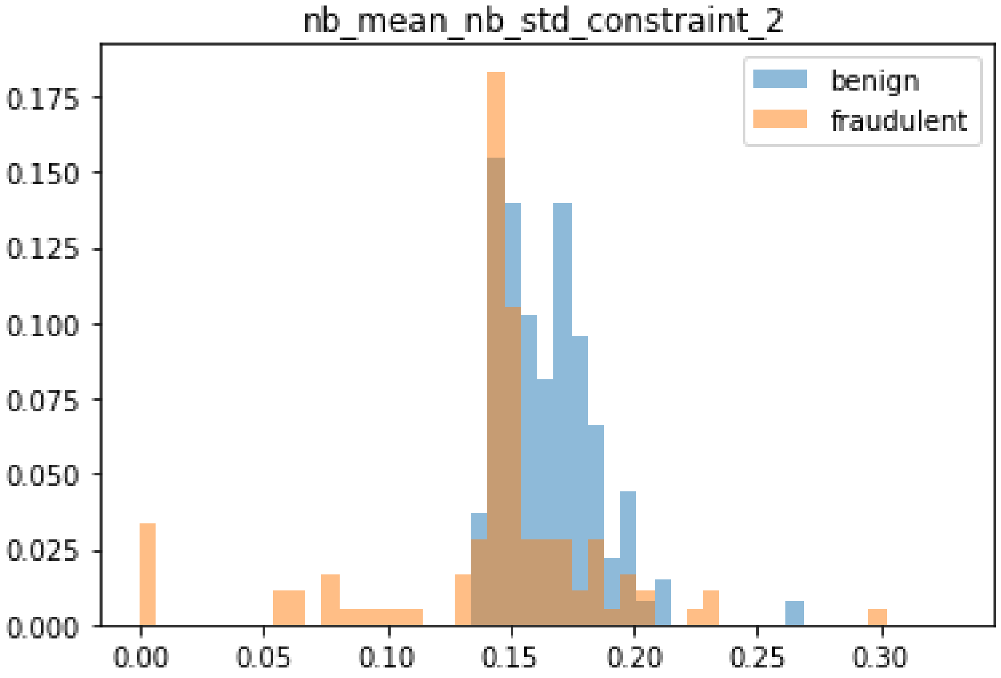
nb_mean_nb_std_constraint_2
benign
fraudulent
0.000
0.025
0.050
0.075
0.100
0.125
0.150
0.175
0.00
0.05
0.10
0.15
0.20
0.25
0.30

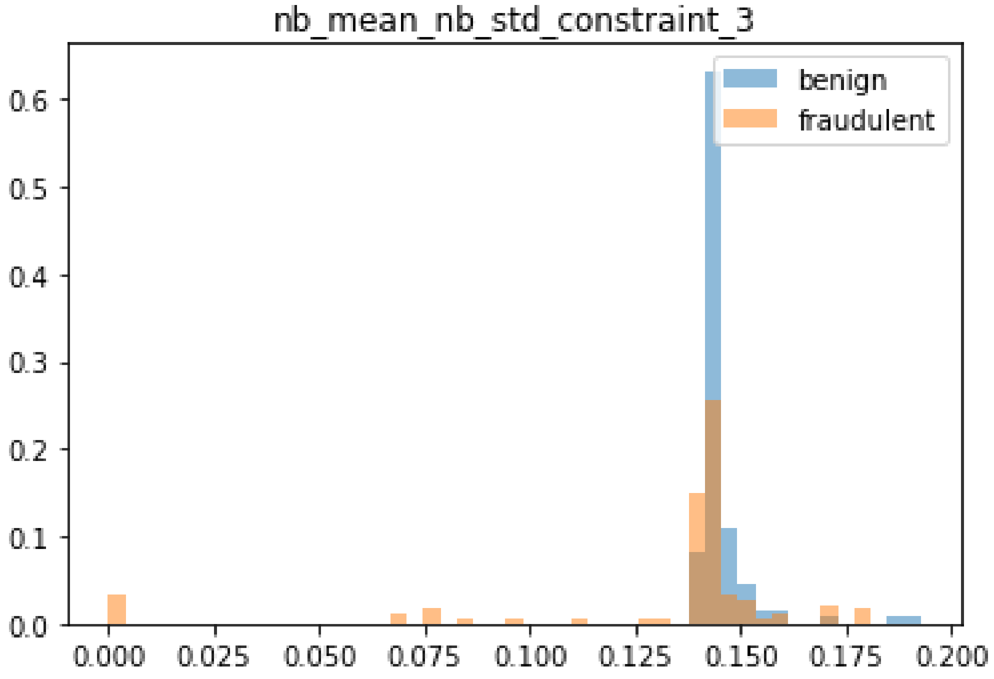
nb_mean_nb_std_constraint_3
benign
fraudulent
0.0
0.1
0.2
0.3
0.4
0.5
0.6
0.000
0.025
0.050
0.075
0.100
0.125
0.150
0.175
0.200

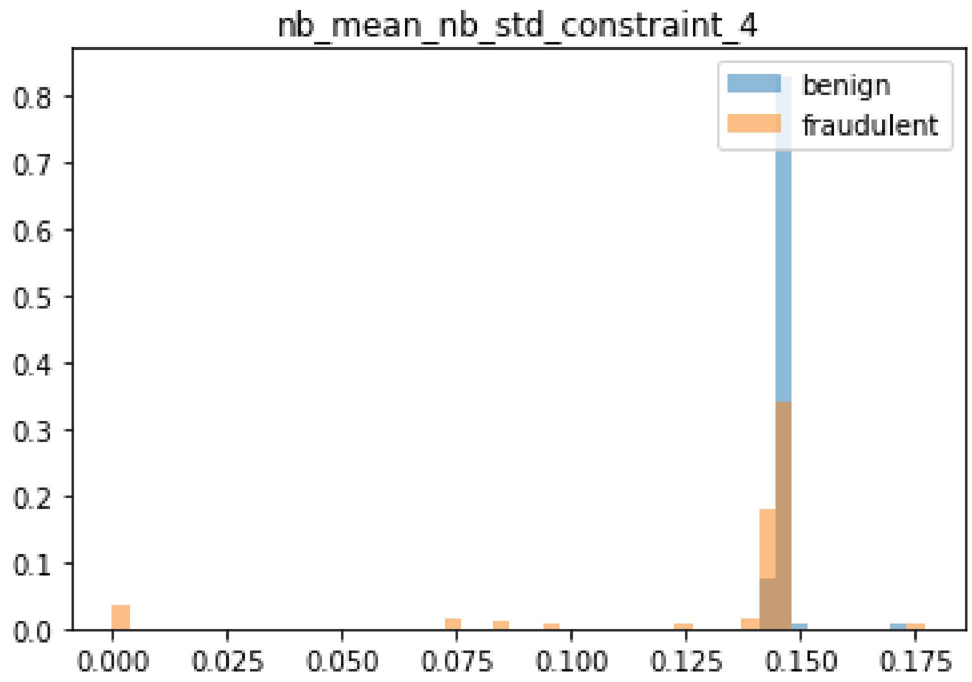
nb_mean_nb_std_constraint_4
benign
fraudulent
0.0
0.1
0.2
0.3
0.4
0.5
0.6
0.7
0.8
0.000
0.025
0.050
0.075
0.100
0.125
0.150
0.175

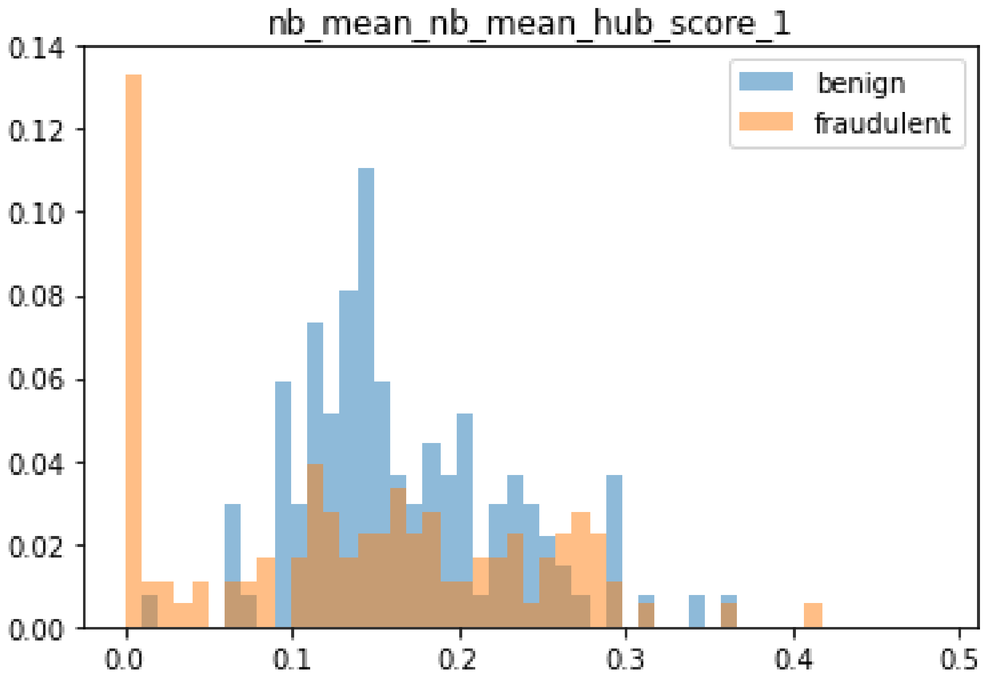
nb_mean_nb_mean_hub_score_1
benign
fraudulent
0.00
0.02
0.04
0.06
0.08
0.10
0.12
0.14
0.0
0.1
0.2
0.3
0.4
0.5

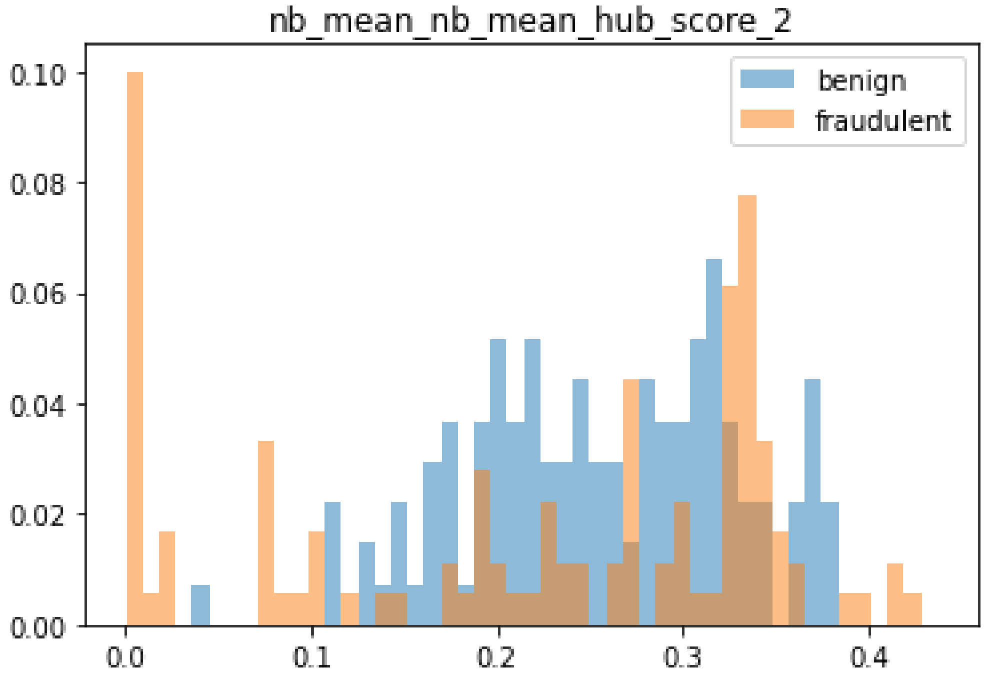
nb_mean_nb_mean_hub_score_2
benign
fraudulent
0.00
0.02
0.04
0.06
0.08
0.10
0.0
0.1
0.2
0.3
0.4

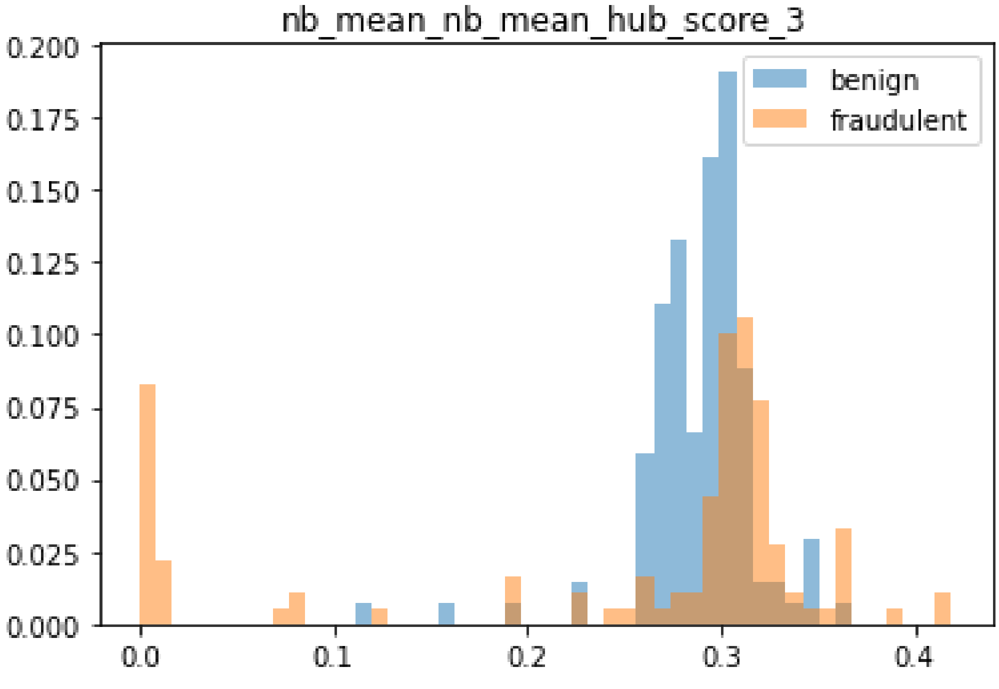
nb_mean_nb_mean_hub_score_3
benign
fraudulent
0.000
0.025
0.050
0.075
0.100
0.125
0.150
0.175
0.200
0.0
0.1
0.2
0.3
0.4

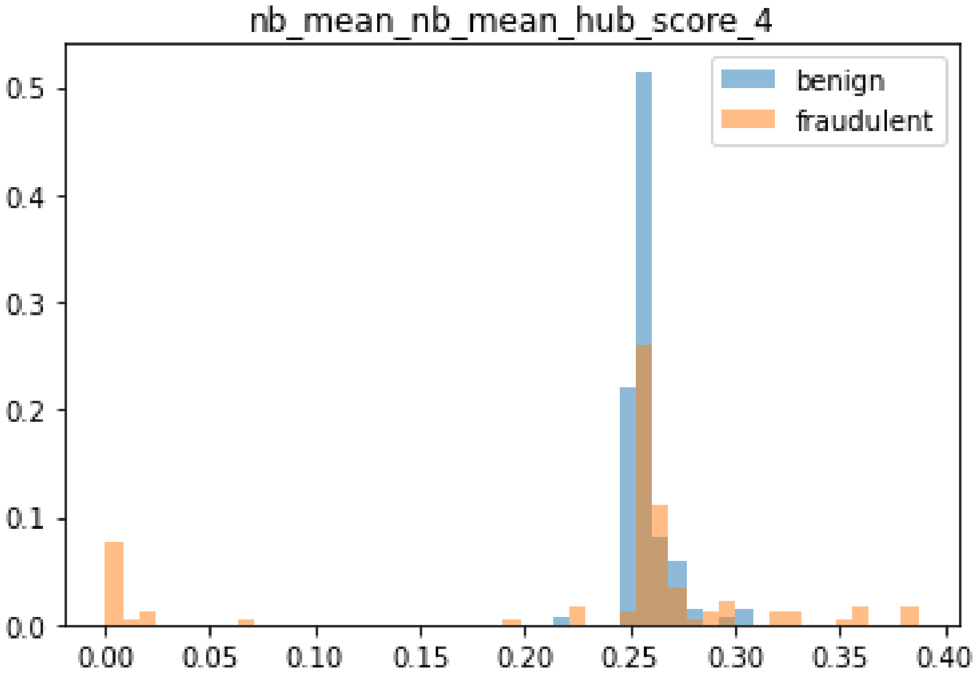
nb_mean_nb_mean_hub_score_4
benign
fraudulent
0.0
0.1
0.2
0.3
0.4
0.5
0.00
0.05
0.10
0.15
0.20
0.25
0.30
0.35
0.40

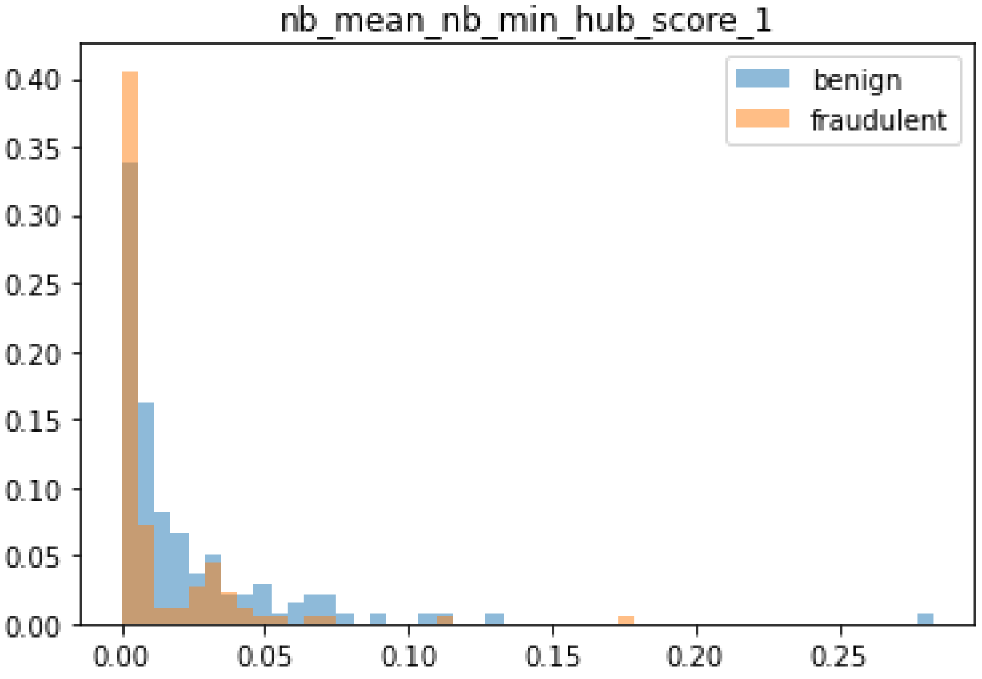
nb_mean_nb_min_hub_score_1
benign
fraudulent
0.00
0.05
0.10
0.15
0.20
0.25
0.30
0.35
0.40
0.00
0.05
0.10
0.15
0.20
0.25

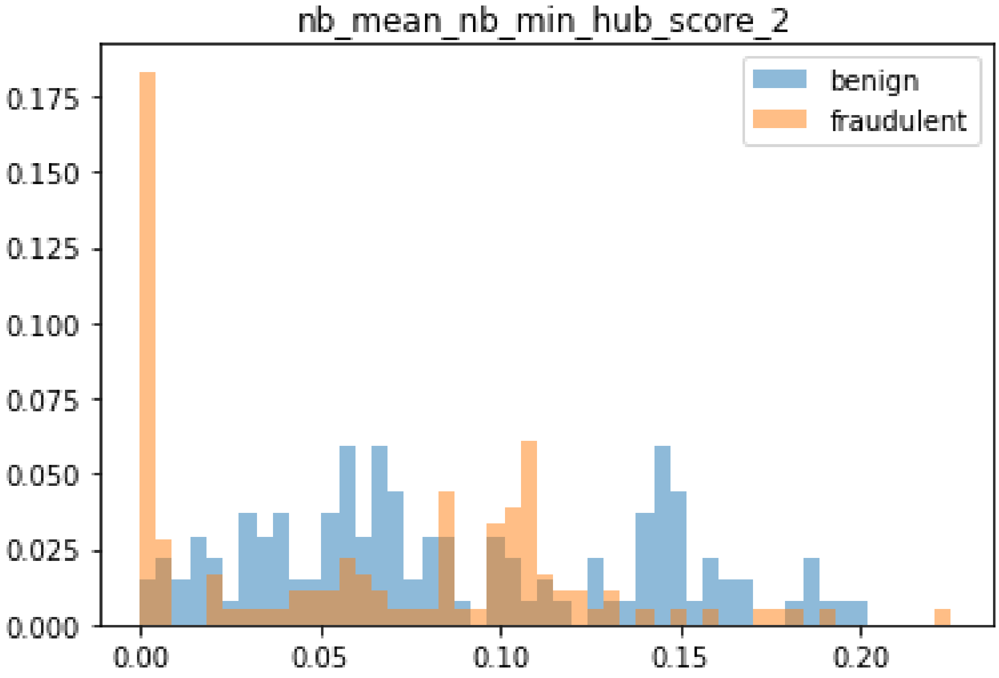
nb_mean_nb_min_hub_score_2
benign
fraudulent
0.175
0.150
0.125
0.100
0.075
0.050
0.025
0.000
0.00
0.05
0.10
0.15
0.20

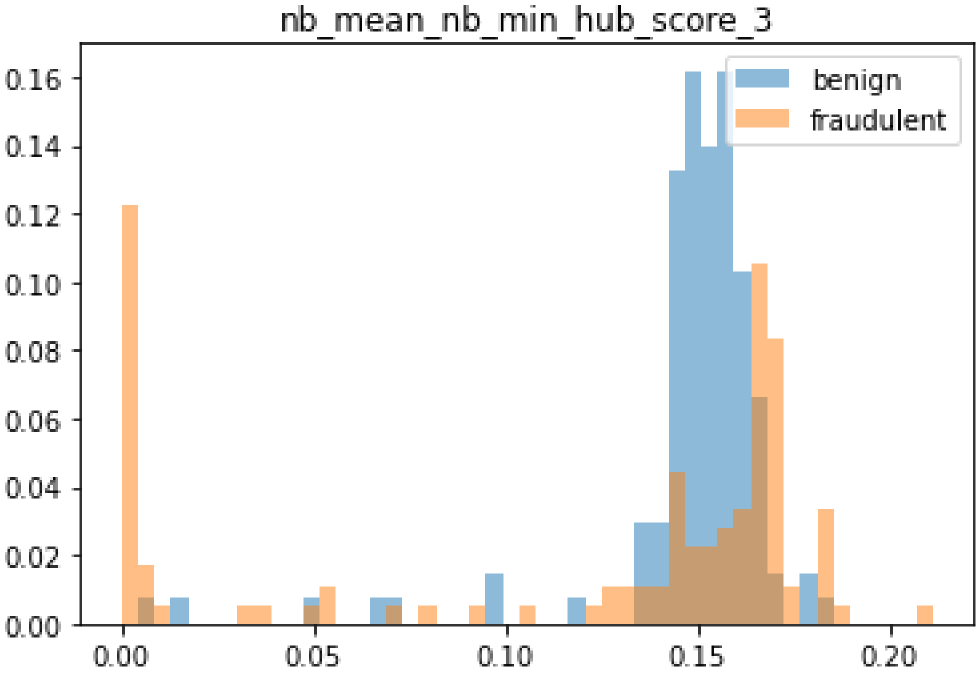
nb_mean_nb_min_hub_score_3
benign
fraudulent
0.16
0.14
0.12
0.10
0.08
0.06
0.04
0.02
0.00
0.00
0.05
0.10
0.15
0.20

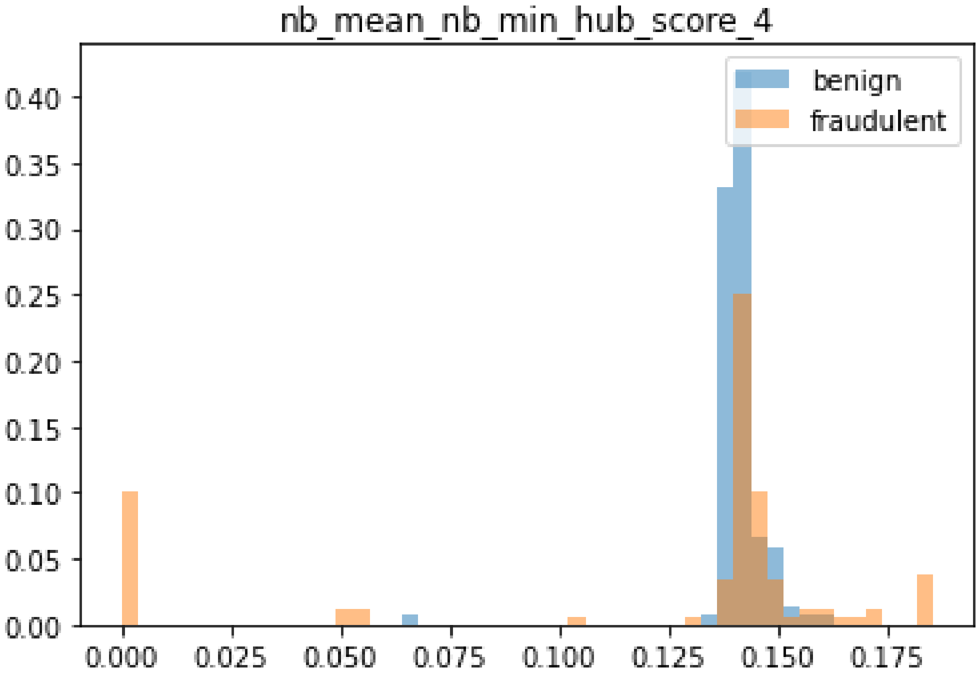
nb_mean_nb_min_hub_score_4
benign
fraudulent
0.40
0.35
0.30
0.25
0.20
0.15
0.10
0.05
0.00
0.000
0.025
0.050
0.075
0.100
0.125
0.150
0.175

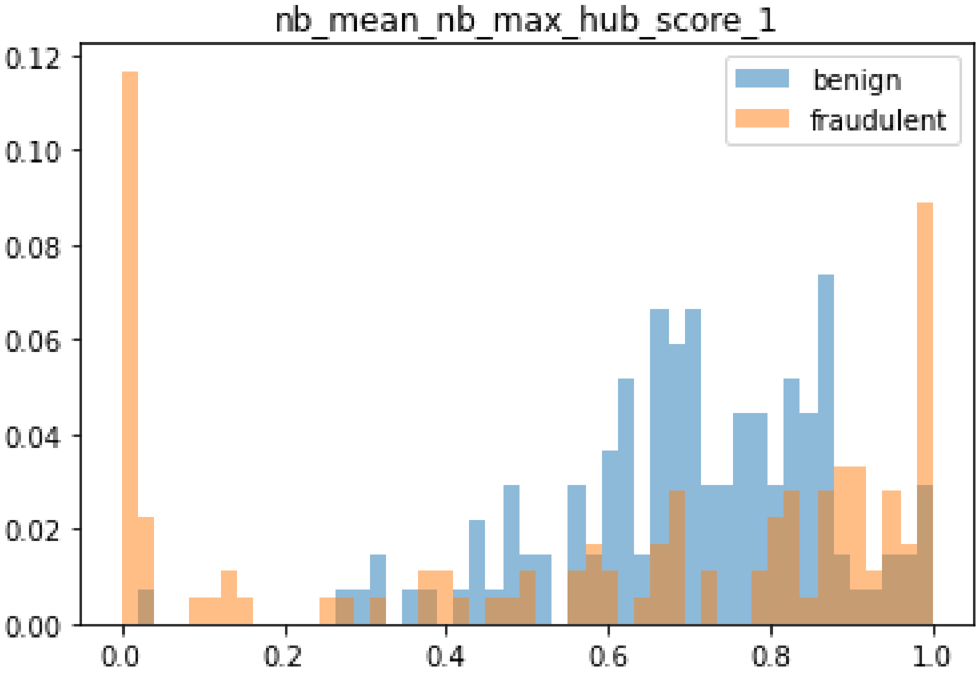
nb_mean_nb_max_hub_score_1
benign
fraudulent
0.12
0.10
0.08
0.06
0.04
0.02
0.00
0.0
0.2
0.4
0.6
0.8
1.0

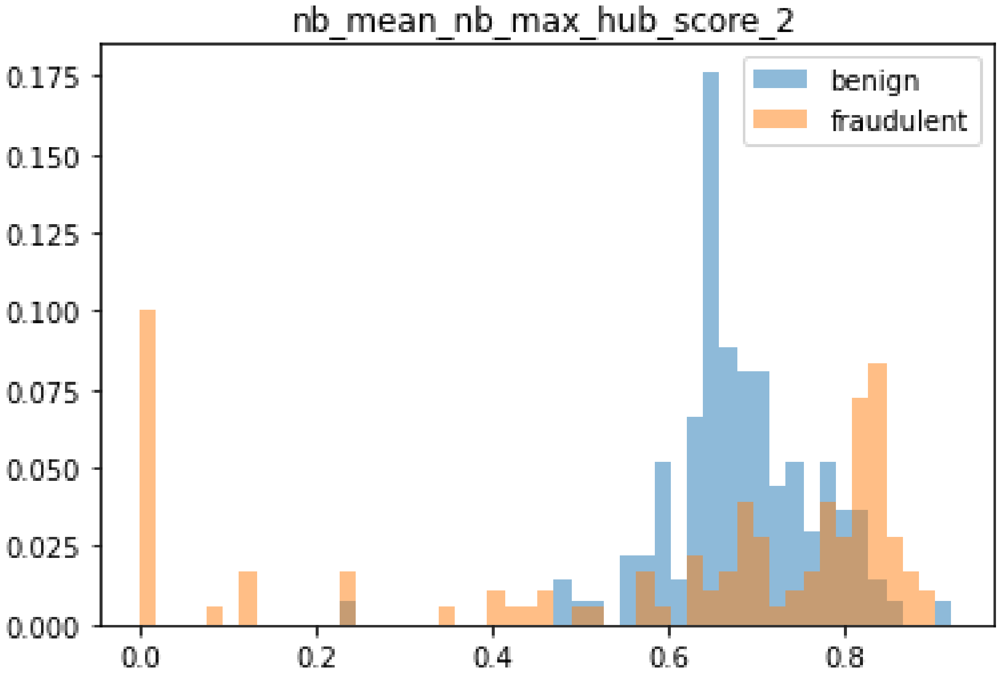
nb_mean_nb_max_hub_score_2
benign
fraudulent
0.175
0.150
0.125
0.100
0.075
0.050
0.025
0.000
0.0
0.2
0.4
0.6
0.8

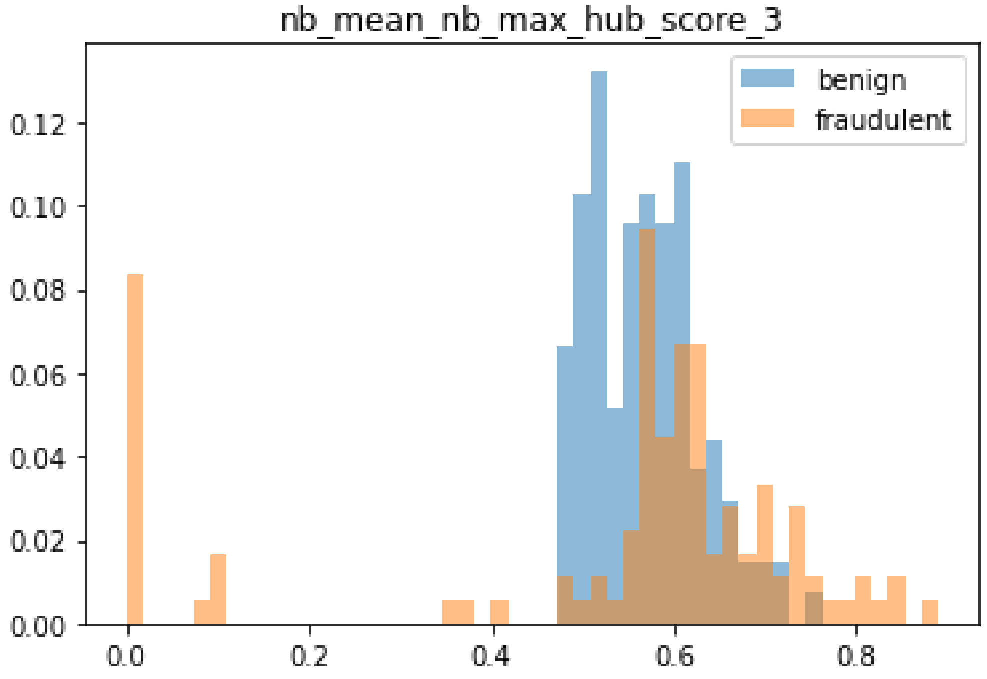
nb_mean_nb_max_hub_score_3
benign
fraudulent
0.12
0.10
0.08
0.06
0.04
0.02
0.00
0.0
0.2
0.4
0.6
0.8

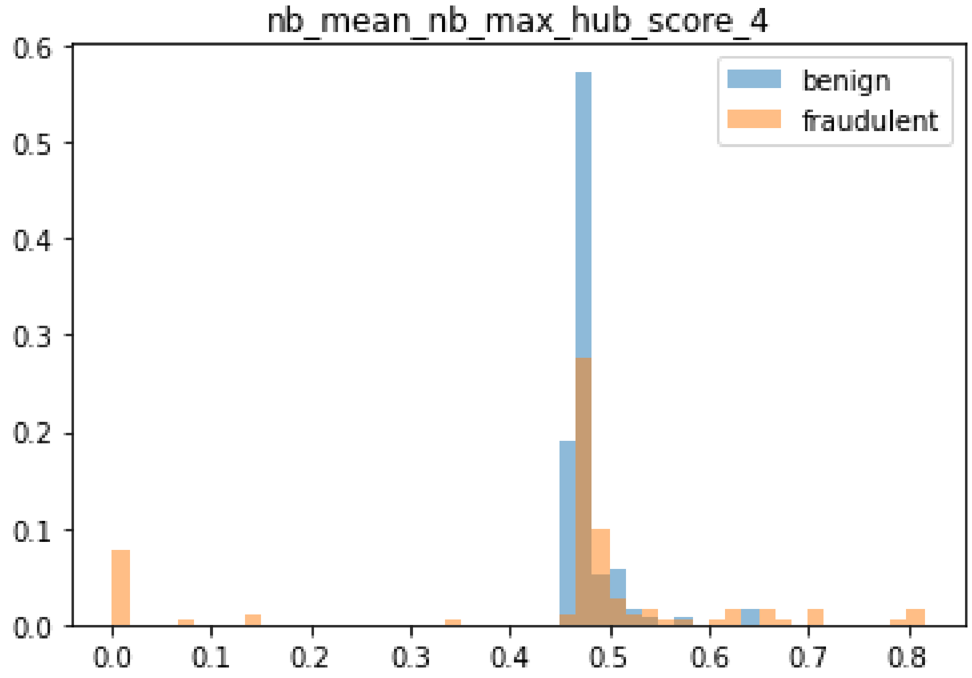

nb_mean_nb_max_hub_score_4
benign
fraudulent
0.6
0.5
0.4
0.3
0.2
0.1
0.0
0.0
0.1
0.2
0.3
0.4
0.5
0.6
0.7
0.8

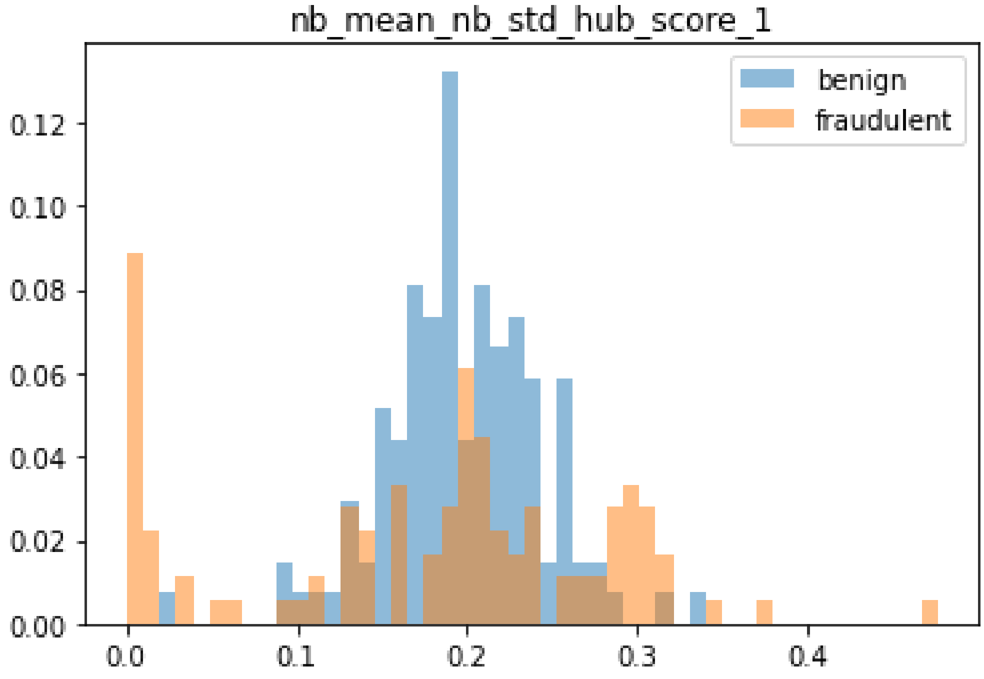

nb_mean_nb_std_hub_score_1
benign
fraudulent
0.12
0.10
0.08
0.06
0.04
0.02
0.00
0.0
0.1
0.2
0.3
0.4

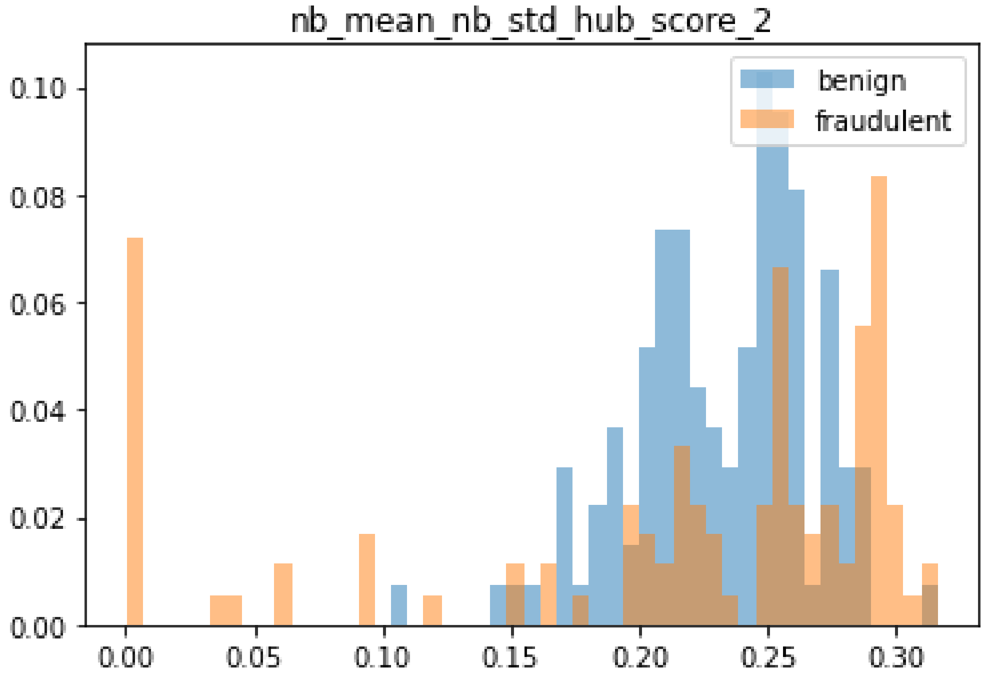
nb_mean_nb_std_hub_score_2
benign
fraudulent
0.00
0.02
0.04
0.06
0.08
0.10
0.00
0.05
0.10
0.15
0.20
0.25
0.30

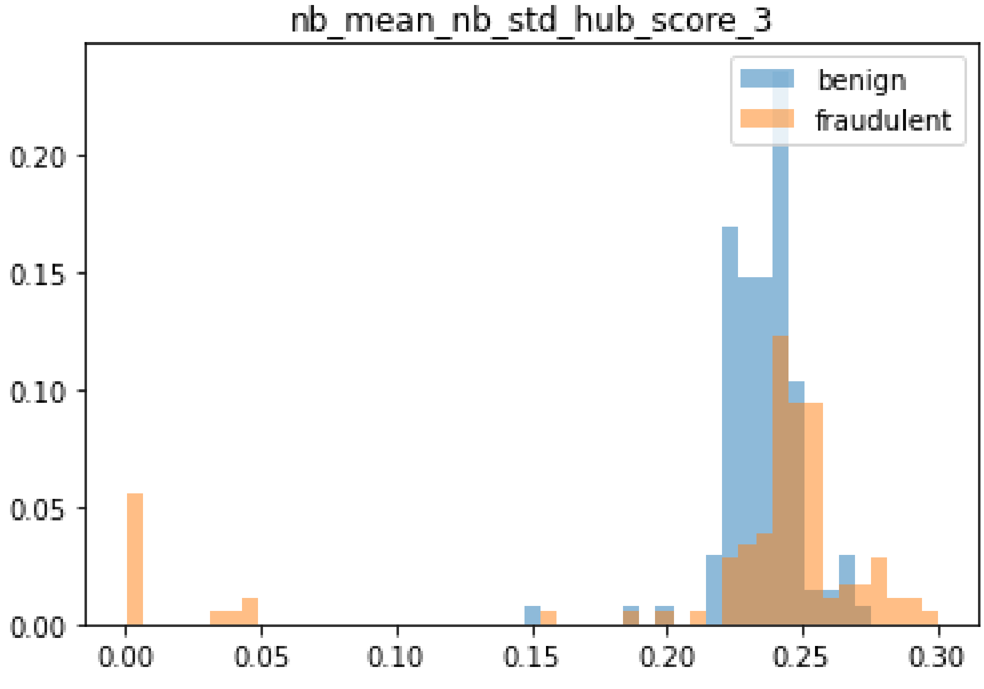
nb_mean_nb_std_hub_score_3
benign
fraudulent
0.00
0.05
0.10
0.15
0.20
0.00
0.05
0.10
0.15
0.20
0.25
0.30

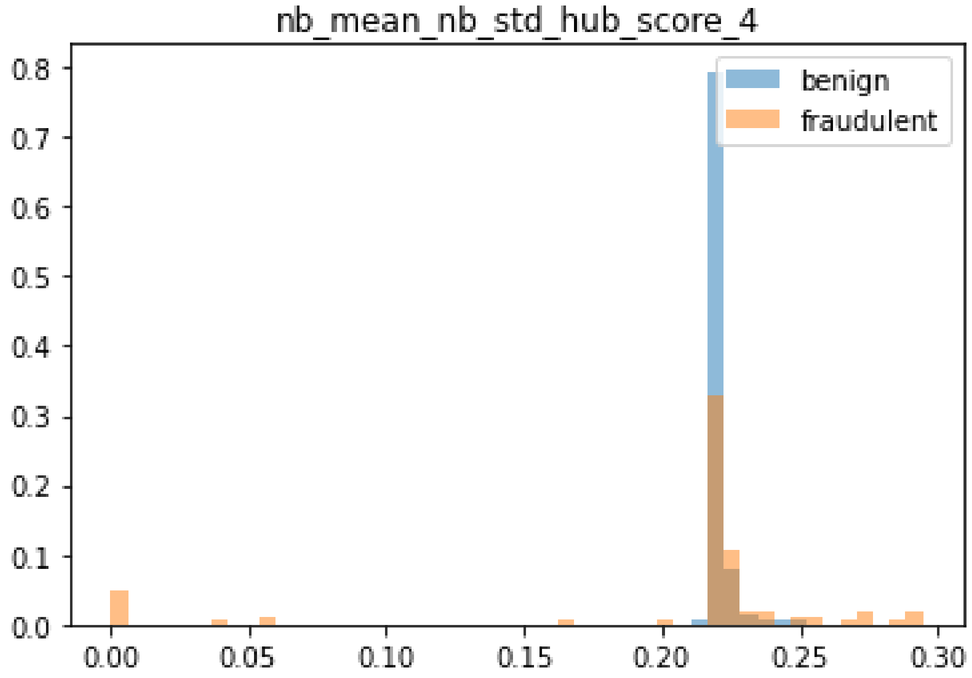
nb_mean_nb_std_hub_score_4
benign
fraudulent
0.8
0.7
0.6
0.5
0.4
0.3
0.2
0.1
0.0
0.00
0.05
0.10
0.15
0.20
0.25
0.30

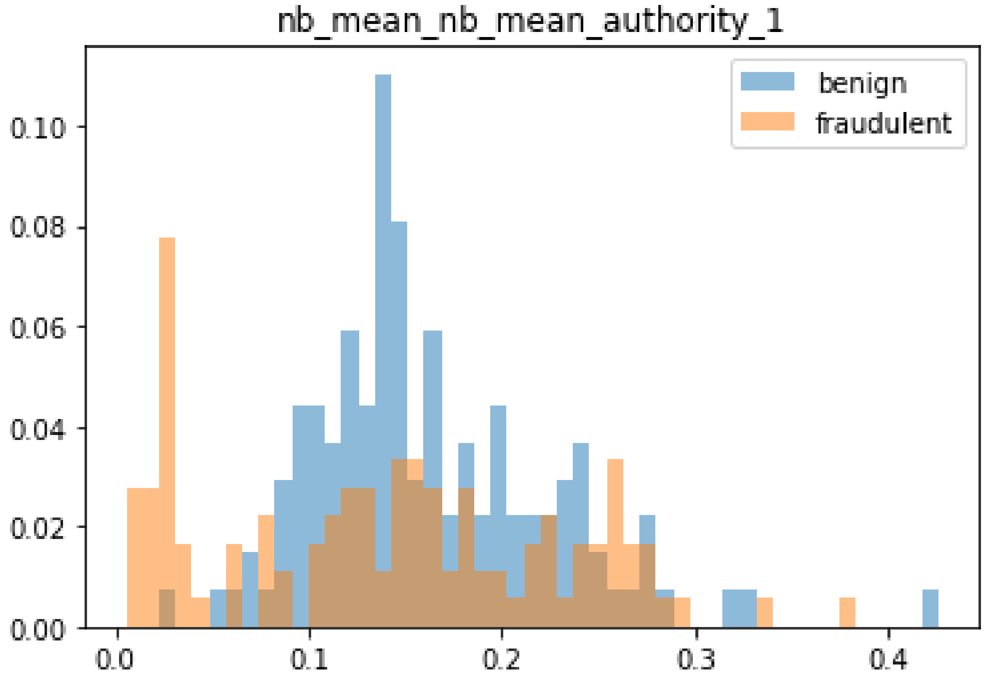
nb_mean_nb_mean_authority_1
benign
fraudulent
0.10
0.08
0.06
0.04
0.02
0.00
0.0
0.1
0.2
0.3
0.4

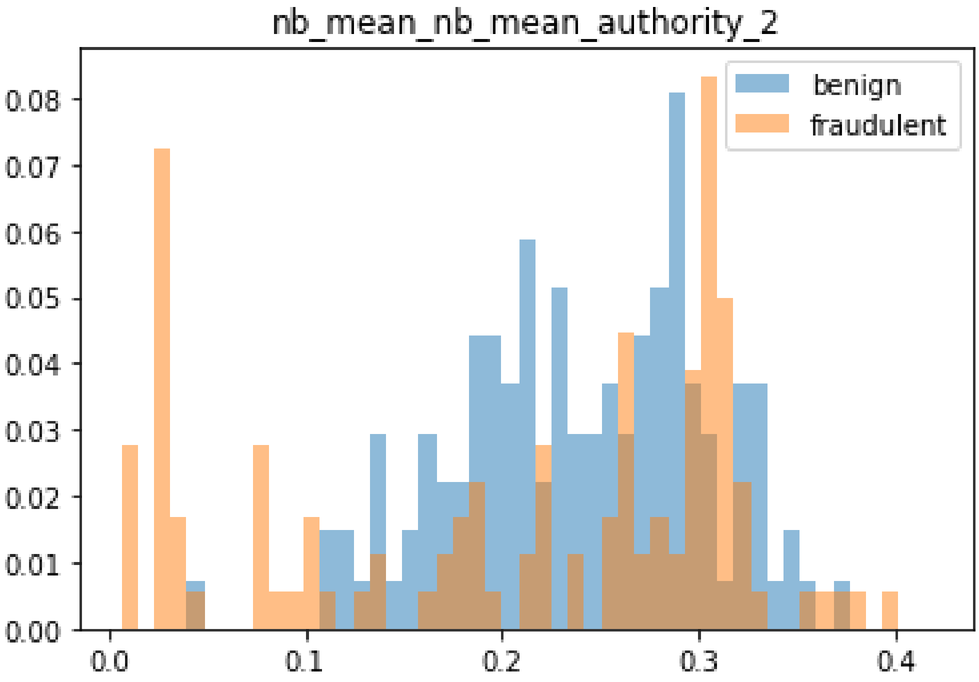
nb_mean_nb_mean_authority_2
benign
fraudulent
0.00
0.01
0.02
0.03
0.04
0.05
0.06
0.07
0.08
0.0
0.1
0.2
0.3
0.4

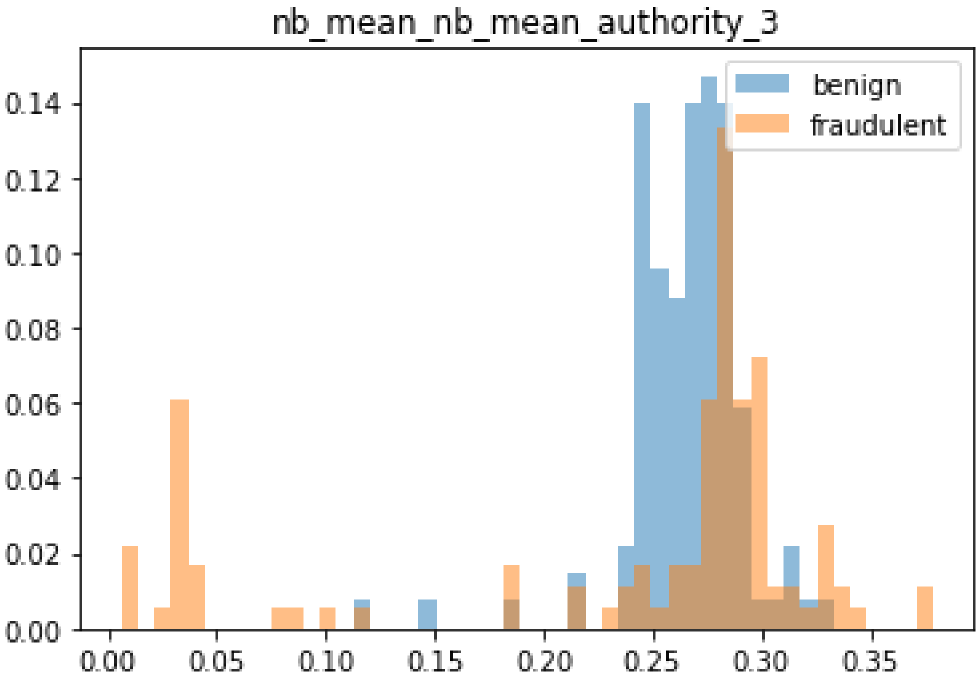
nb_mean_nb_mean_authority_3
benign
fraudulent
0.00
0.02
0.04
0.06
0.08
0.10
0.12
0.14
0.00
0.05
0.10
0.15
0.20
0.25
0.30
0.35

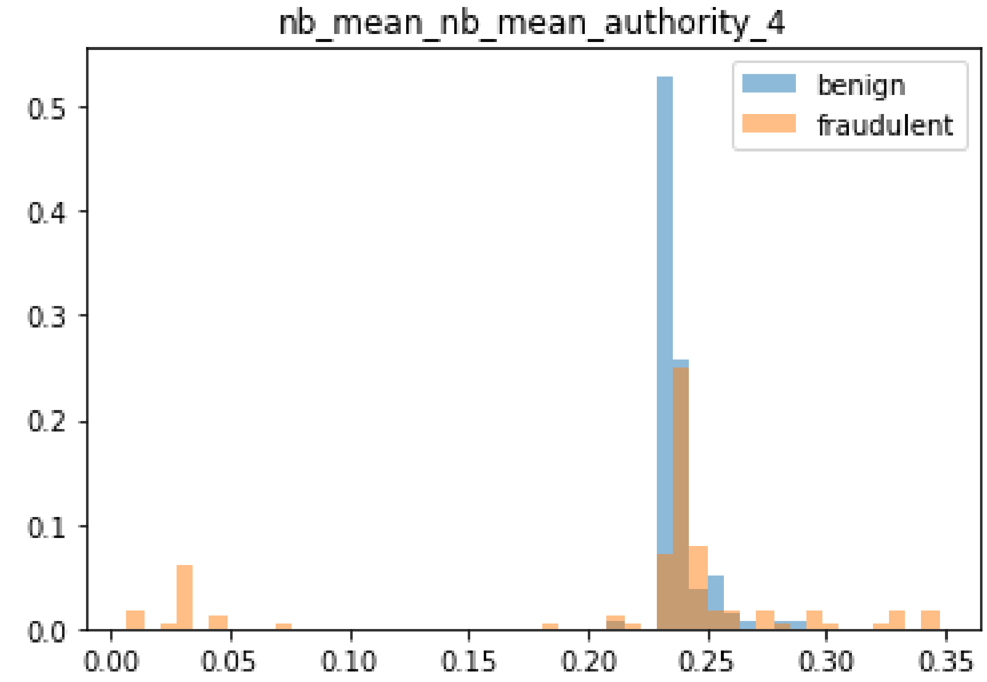
nb_mean_nb_mean_authority_4
benign
fraudulent
0.0
0.1
0.2
0.3
0.4
0.5
0.00
0.05
0.10
0.15
0.20
0.25
0.30
0.35

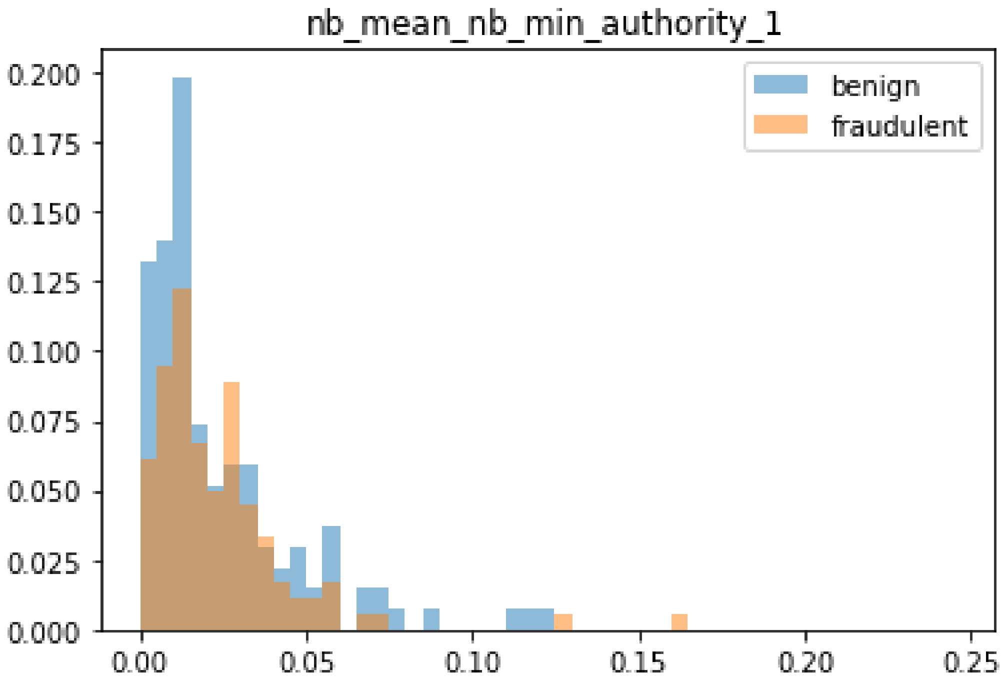
nb_mean_nb_min_authority_1
benign
fraudulent
0.000
0.025
0.050
0.075
0.100
0.125
0.150
0.175
0.200
0.00
0.05
0.10
0.15
0.20
0.25

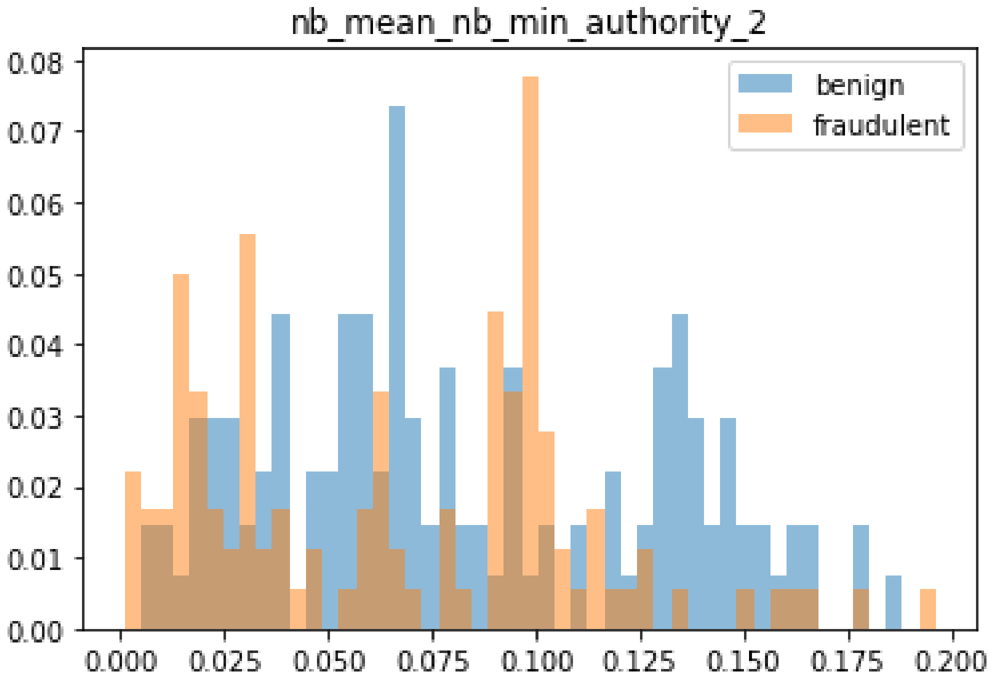
nb_mean_nb_min_authority_2
benign
fraudulent
0.08
0.07
0.06
0.05
0.04
0.03
0.02
0.01
0.00
0.000
0.025
0.050
0.075
0.100
0.125
0.150
0.175
0.200

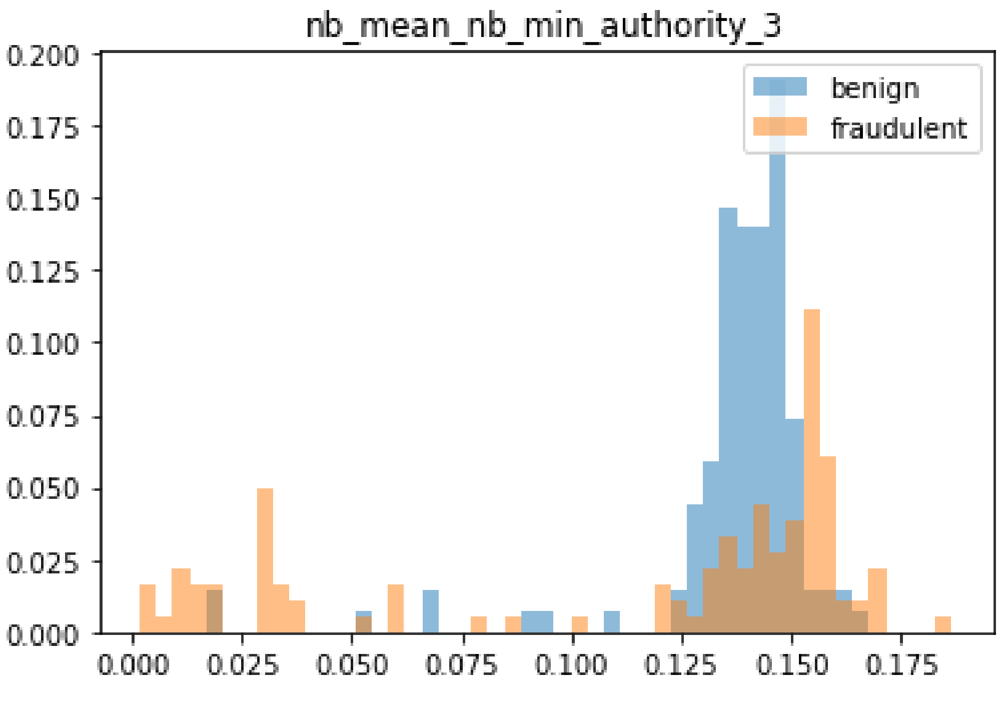
nb_mean_nb_min_authority_3
benign
fraudulent
0.200
0.175
0.150
0.125
0.100
0.075
0.050
0.025
0.000
0.000
0.025
0.050
0.075
0.100
0.125
0.150
0.175

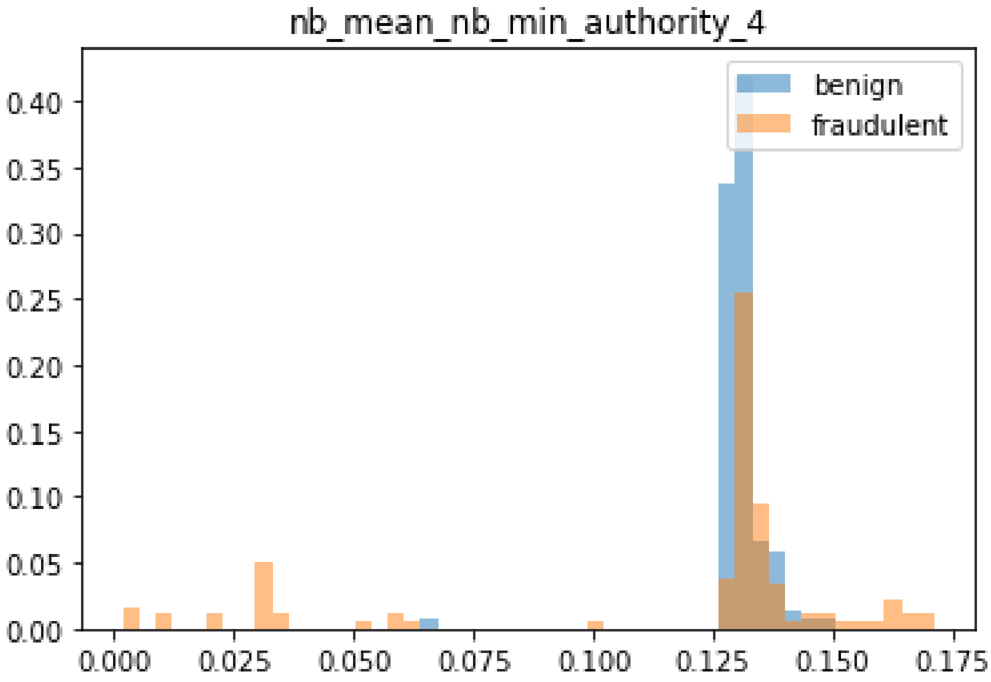
nb_mean_nb_min_authority_4
benign
fraudulent
0.40
0.35
0.30
0.25
0.20
0.15
0.10
0.05
0.00
0.000
0.025
0.050
0.075
0.100
0.125
0.150
0.175

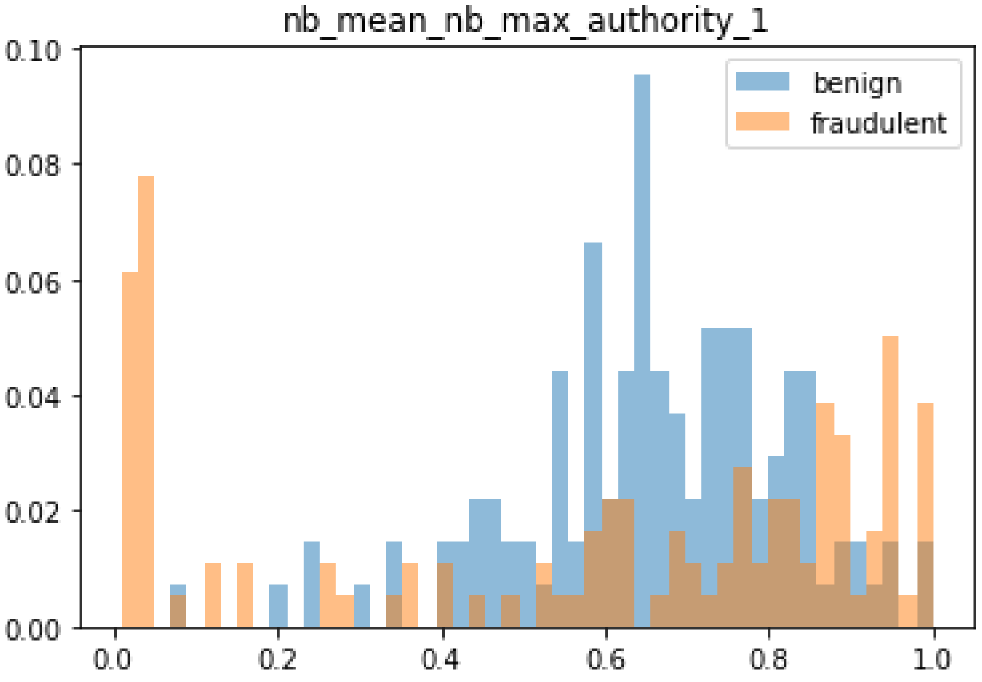
nb_mean_nb_max_authority_1
benign
fraudulent
0.10
0.08
0.06
0.04
0.02
0.00
0.0
0.2
0.4
0.6
0.8
1.0

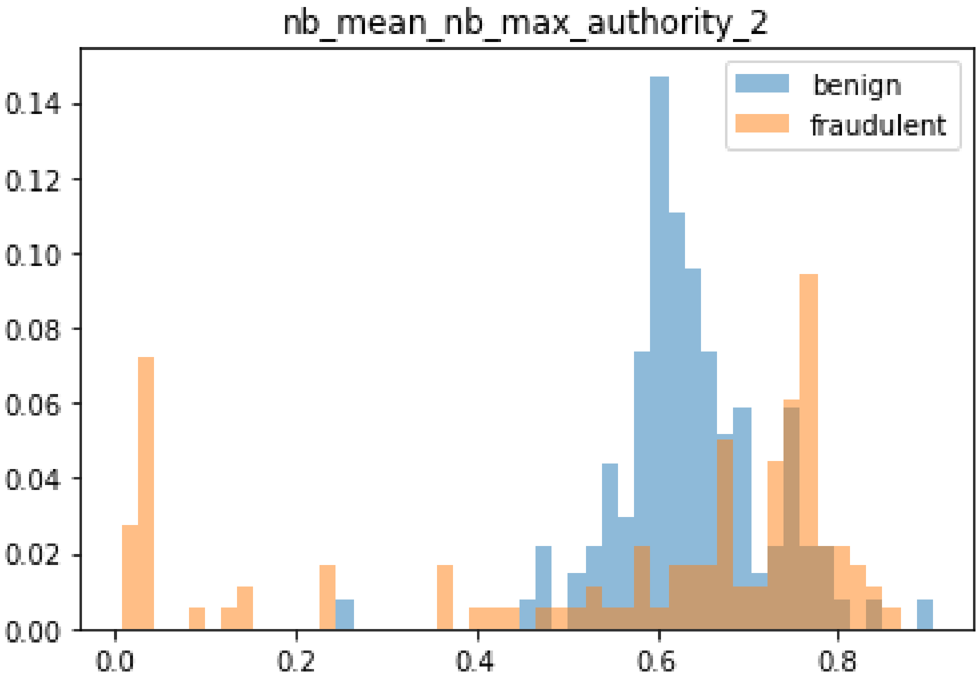
nb_mean_nb_max_authority_2
benign
fraudulent
0.14
0.12
0.10
0.08
0.06
0.04
0.02
0.00
0.0
0.2
0.4
0.6
0.8

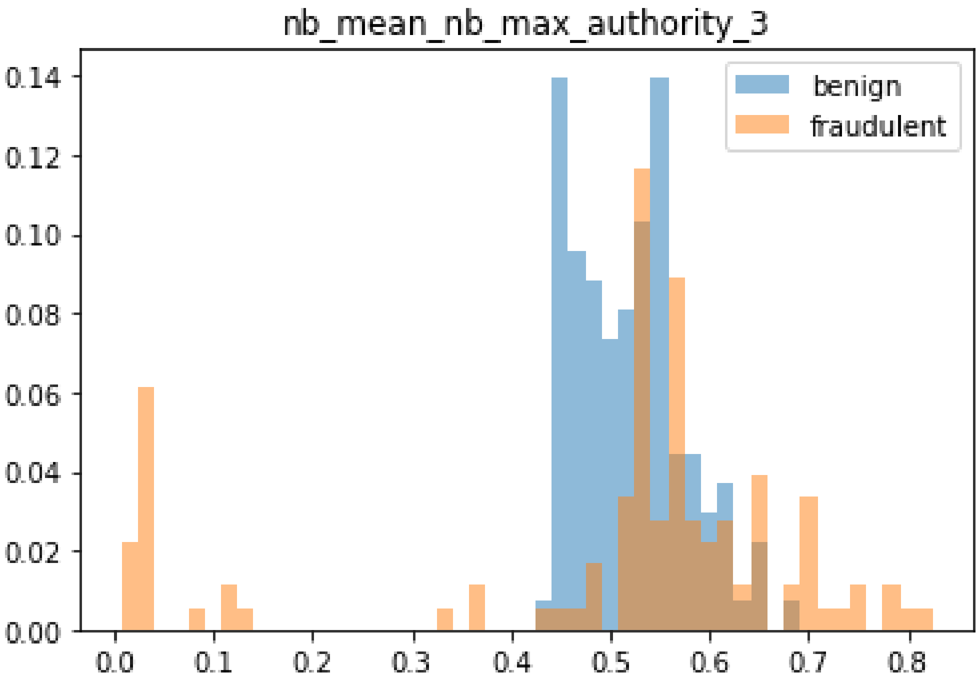
nb_mean_nb_max_authority_3
benign
fraudulent
0.14
0.12
0.10
0.08
0.06
0.04
0.02
0.00
0.0
0.1
0.2
0.3
0.4
0.5
0.6
0.7
0.8

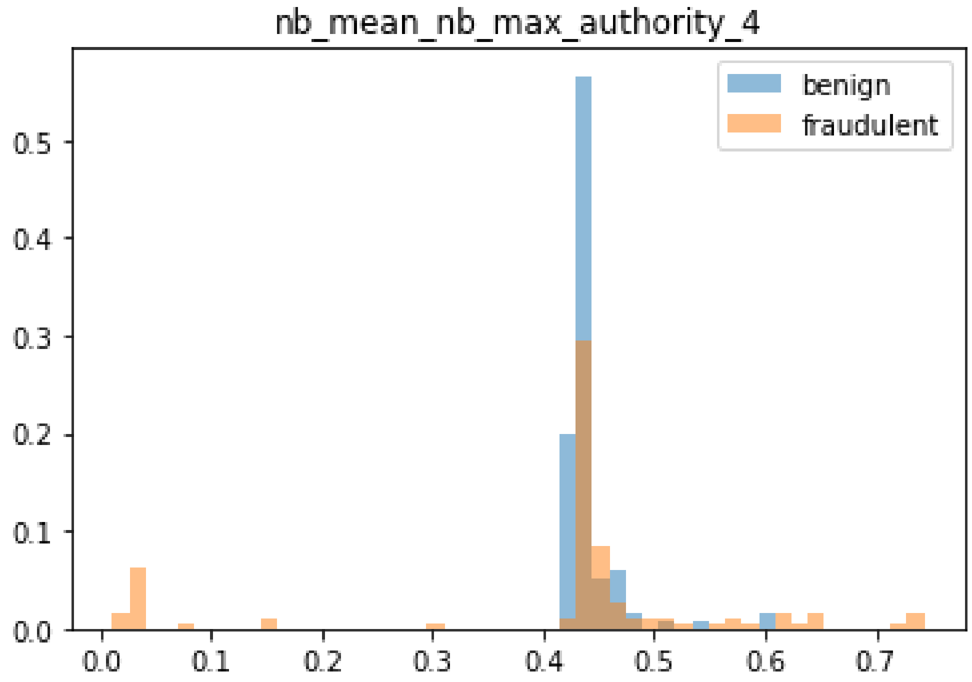
nb_mean_nb_max_authority_4
benign
fraudulent
0.0
0.1
0.2
0.3
0.4
0.5
0.0
0.1
0.2
0.3
0.4
0.5
0.6
0.7

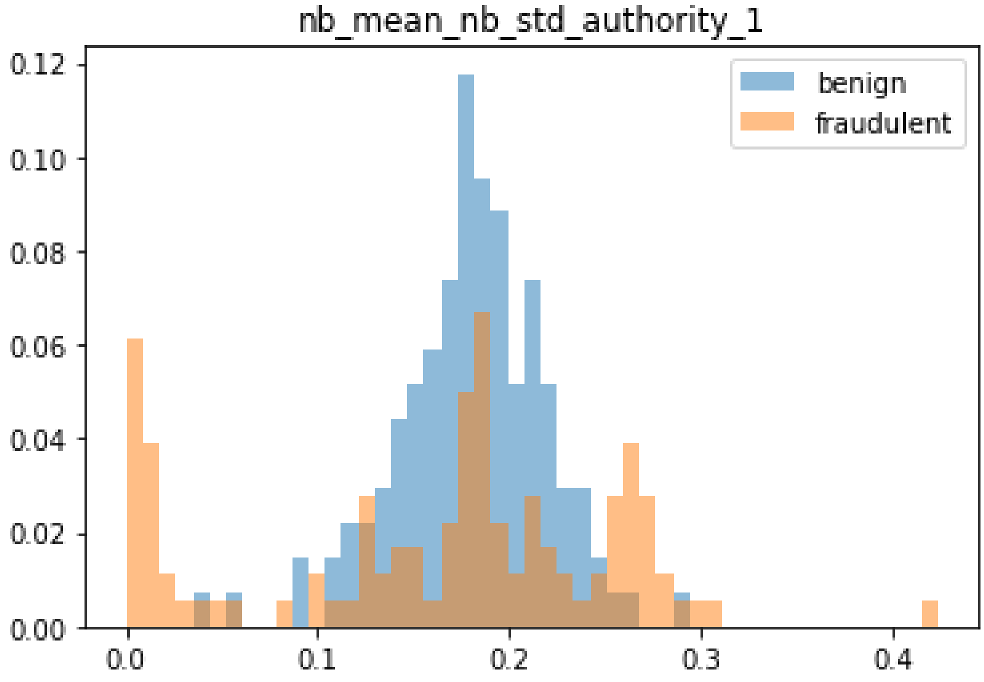
nb_mean_nb_std_authority_1
benign
fraudulent
0.00
0.02
0.04
0.06
0.08
0.10
0.12
0.0
0.1
0.2
0.3
0.4

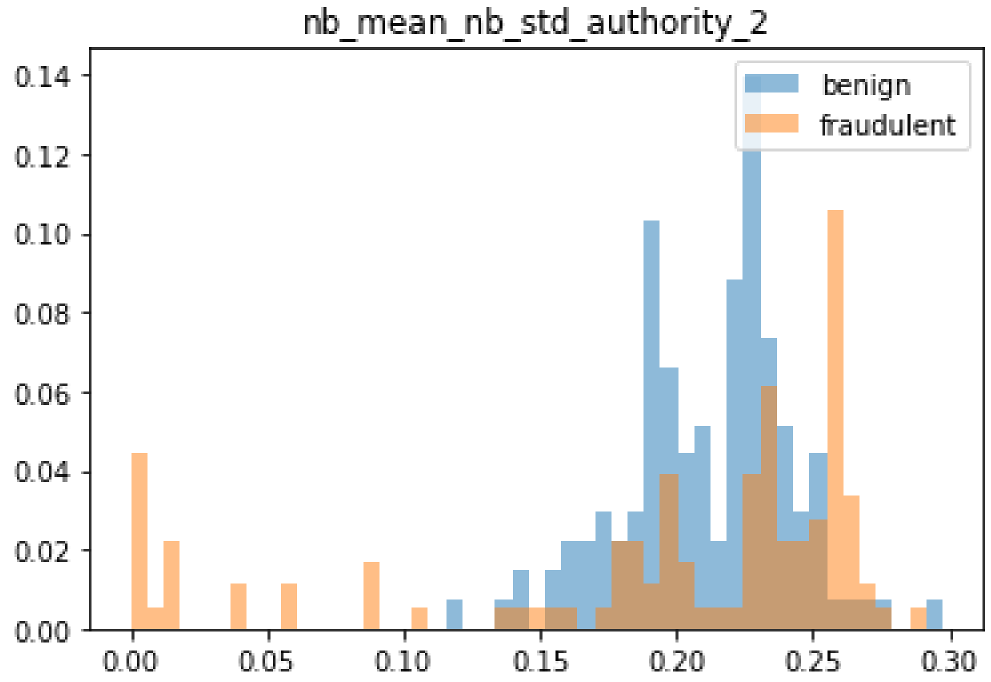

nb_mean_nb_std_authority_2
benign
fraudulent
0.14
0.12
0.10
0.08
0.06
0.04
0.02
0.00
0.00
0.05
0.10
0.15
0.20
0.25
0.30

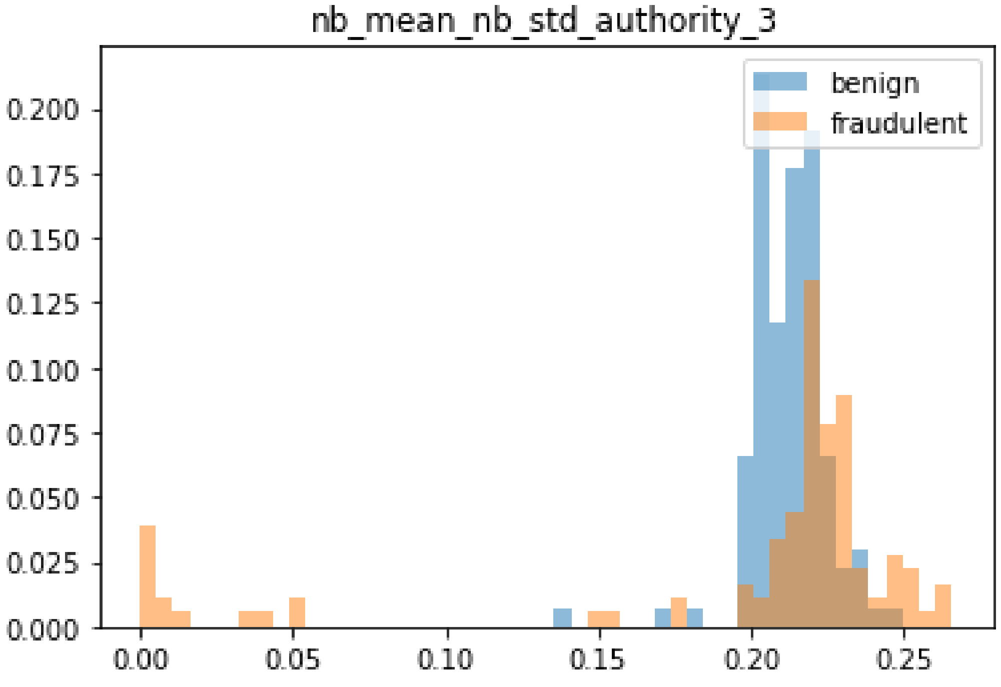

nb_mean_nb_std_authority_3
benign
fraudulent
0.200
0.175
0.150
0.125
0.100
0.075
0.050
0.025
0.000
0.00
0.05
0.10
0.15
0.20
0.25

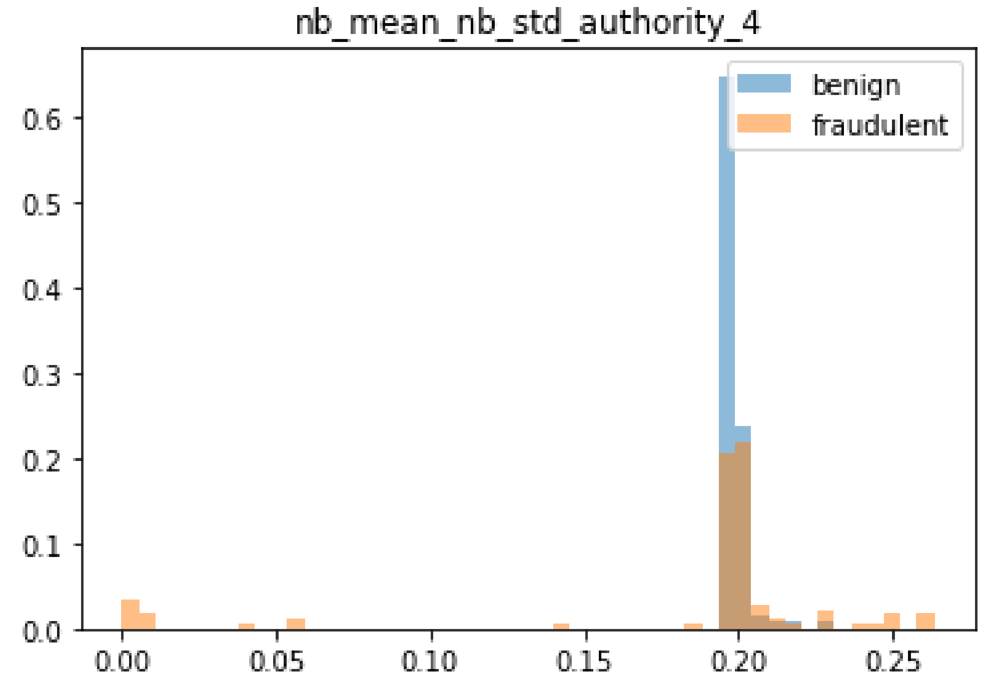
nb_mean_nb_std_authority_4
benign
fraudulent
0.0
0.1
0.2
0.3
0.4
0.5
0.6
0.00
0.05
0.10
0.15
0.20
0.25

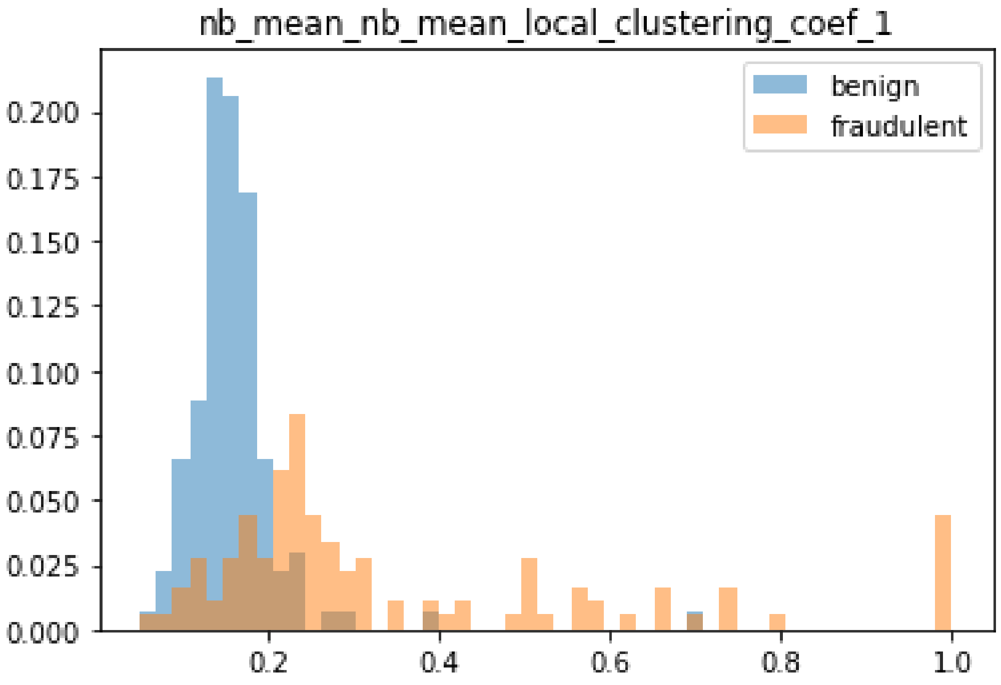
nb_mean_nb_mean_local_clustering_coef_1
benign
fraudulent
0.000
0.025
0.050
0.075
0.100
0.125
0.150
0.175
0.200
0.2
0.4
0.6
0.8
1.0

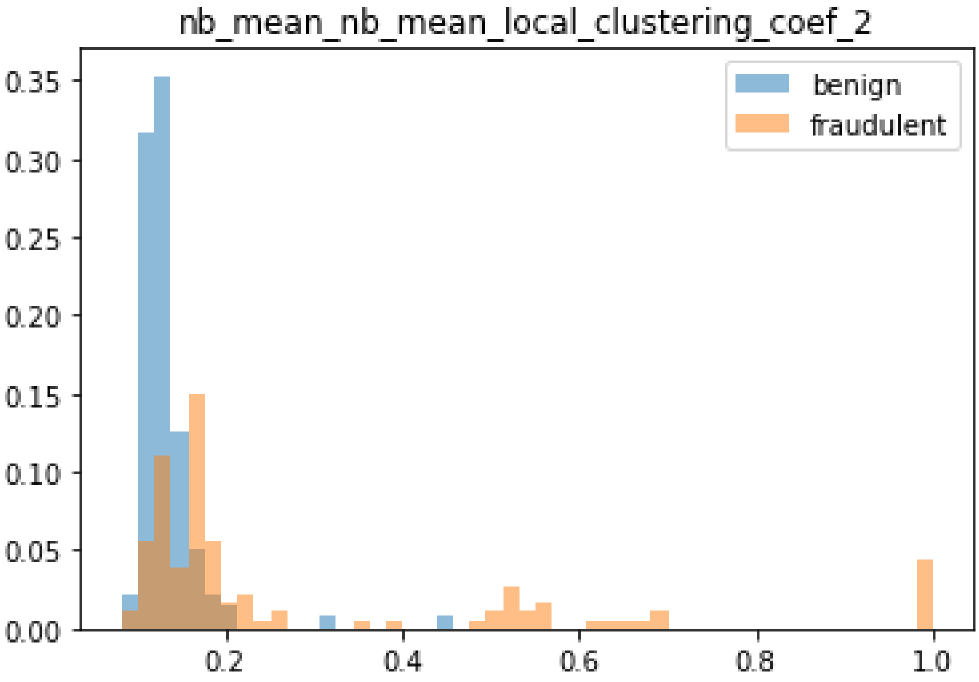
nb_mean_nb_mean_local_clustering_coef_2
benign
fraudulent
0.35
0.30
0.25
0.20
0.15
0.10
0.05
0.00
0.2
0.4
0.6
0.8
1.0

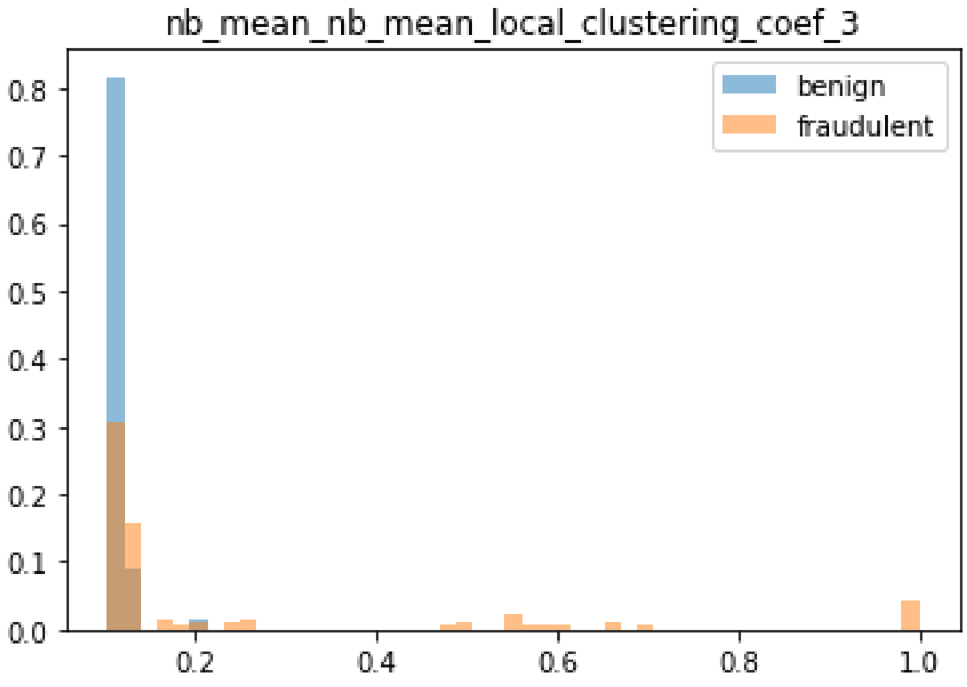
nb_mean_nb_mean_local_clustering_coef_3
benign
fraudulent
0.8
0.7
0.6
0.5
0.4
0.3
0.2
0.1
0.0
0.2
0.4
0.6
0.8
1.0

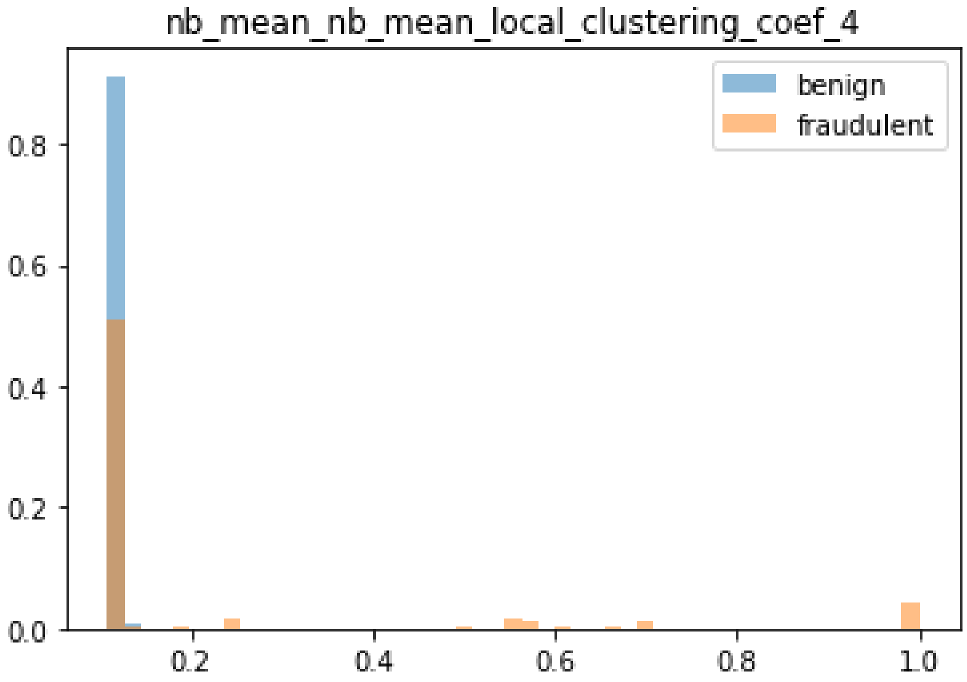
nb_mean_nb_mean_local_clustering_coef_4
benign
fraudulent
0.8
0.6
0.4
0.2
0.0
0.2
0.4
0.6
0.8
1.0

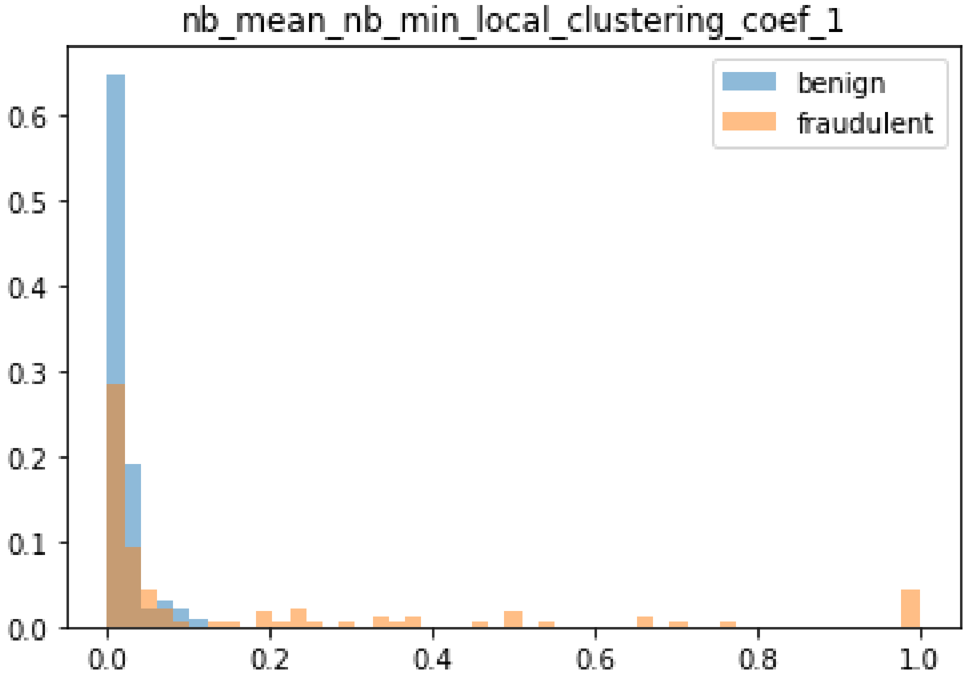
nb_mean_nb_min_local_clustering_coef_1
benign
fraudulent
0.6
0.5
0.4
0.3
0.2
0.1
0.0
0.0
0.2
0.4
0.6
0.8
1.0

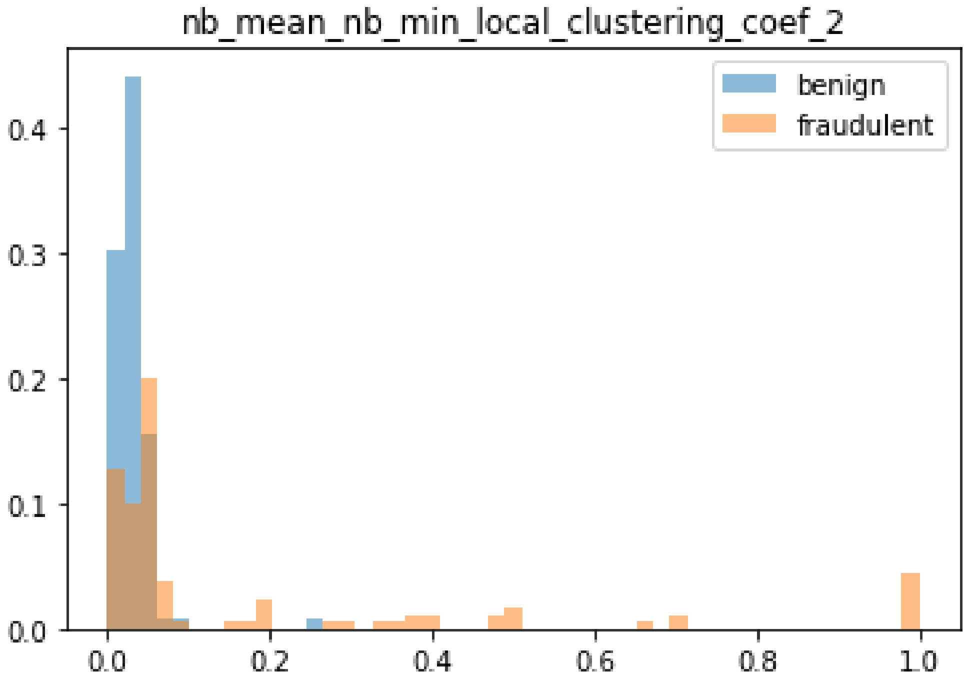
nb_mean_nb_min_local_clustering_coef_2
benign
fraudulent
0.0
0.1
0.2
0.3
0.4
0.0
0.2
0.4
0.6
0.8
1.0

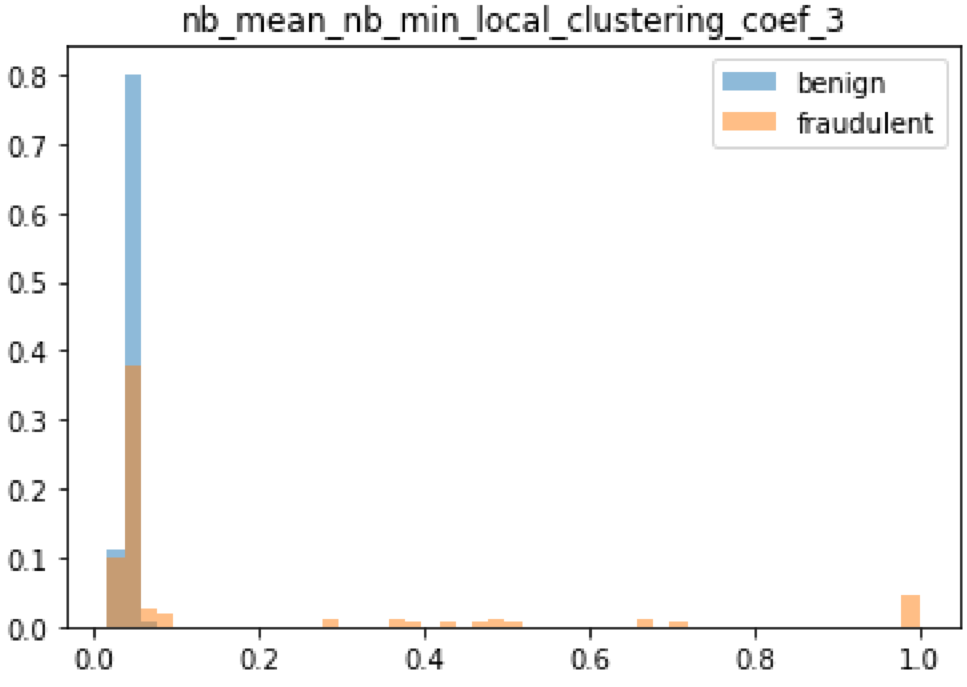
nb_mean_nb_min_local_clustering_coef_3
benign
fraudulent
0.0
0.1
0.2
0.3
0.4
0.5
0.6
0.7
0.8
0.0
0.2
0.4
0.6
0.8
1.0

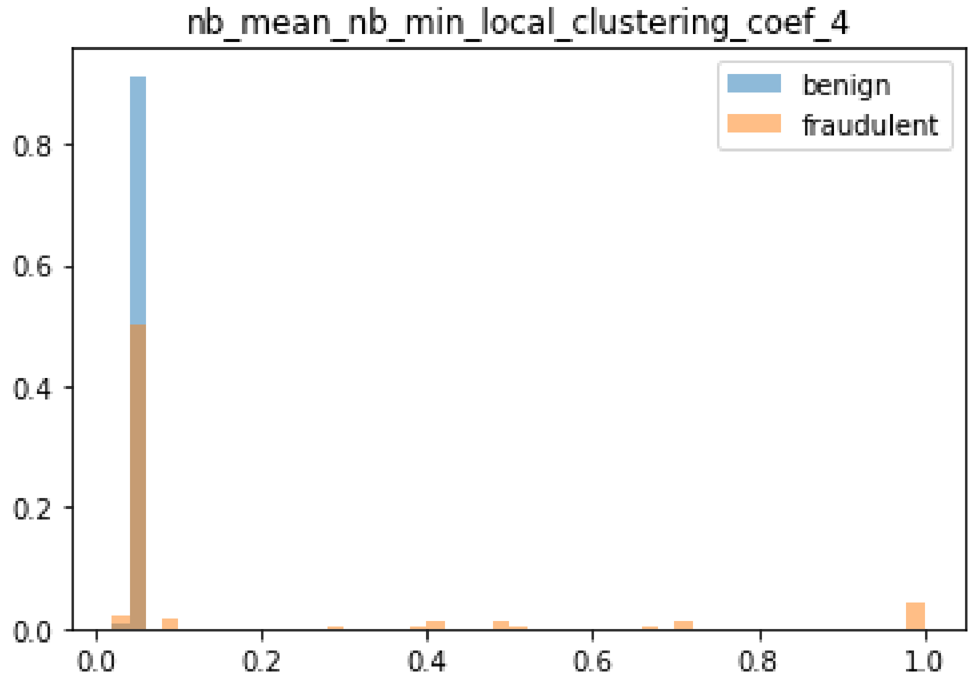
nb_mean_nb_min_local_clustering_coef_4
benign
fraudulent
0.0
0.2
0.4
0.6
0.8
1.0

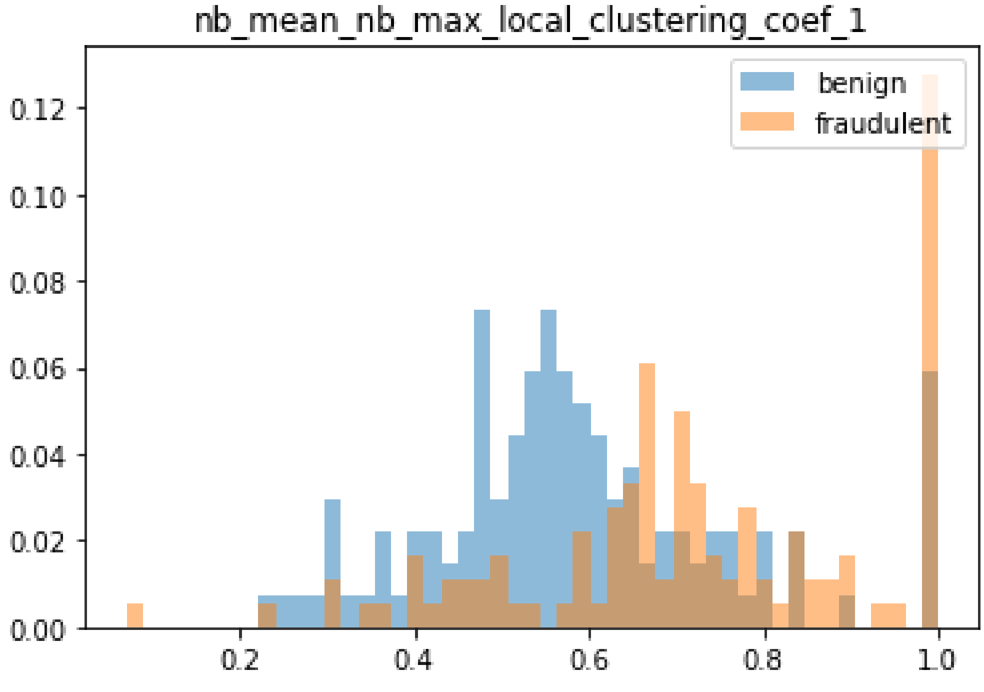
nb_mean_nb_max_local_clustering_coef_1
benign
fraudulent
0.00
0.02
0.04
0.06
0.08
0.10
0.12
0.2
0.4
0.6
0.8
1.0

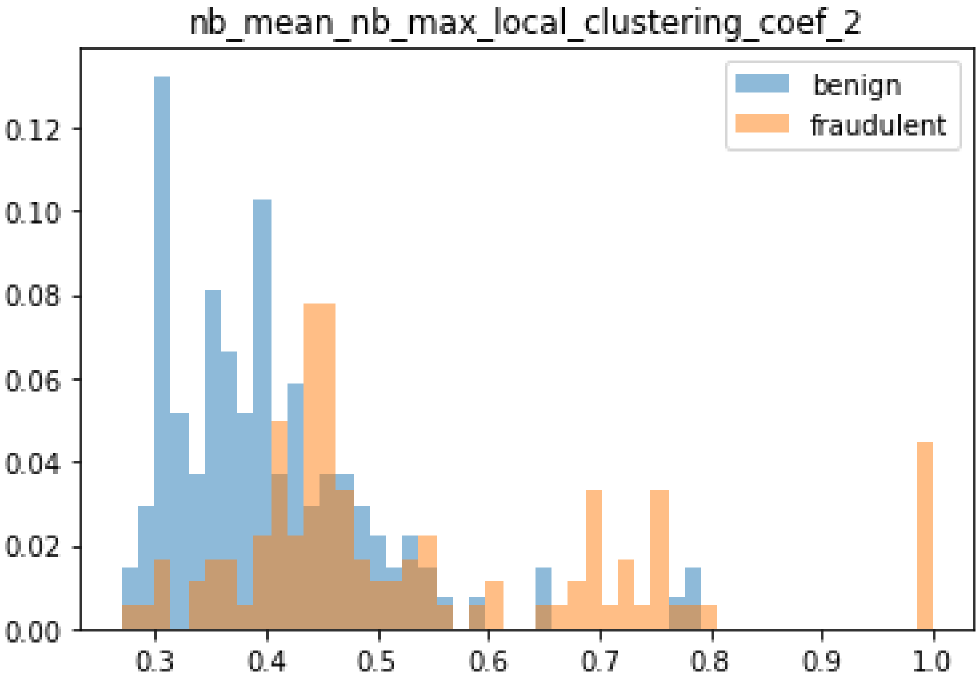
nb_mean_nb_max_local_clustering_coef_2
benign
fraudulent
0.00
0.02
0.04
0.06
0.08
0.10
0.12
0.3
0.4
0.5
0.6
0.7
0.8
0.9
1.0

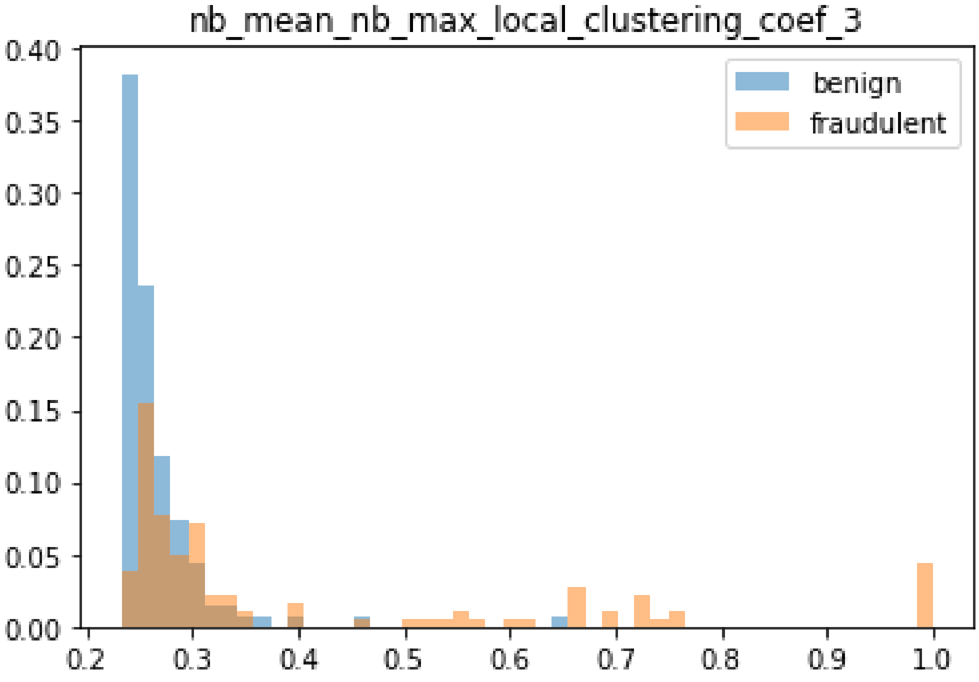
nb_mean_nb_max_local_clustering_coef_3
benign
fraudulent
0.00
0.05
0.10
0.15
0.20
0.25
0.30
0.35
0.40
0.2
0.3
0.4
0.5
0.6
0.7
0.8
0.9
1.0

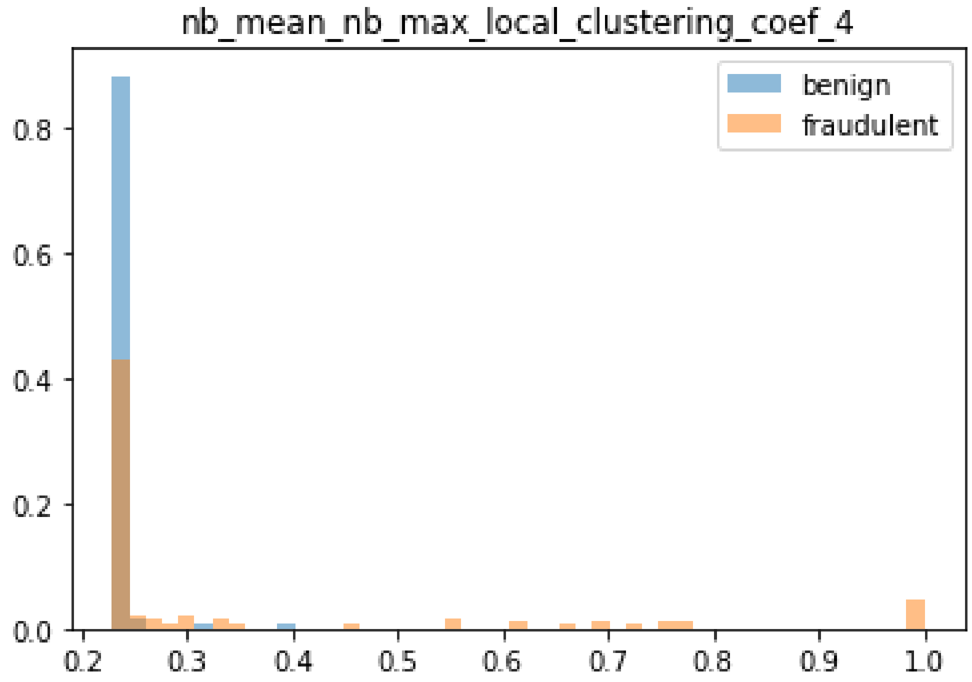
nb_mean_nb_max_local_clustering_coef_4
benign
fraudulent
0.0
0.2
0.4
0.6
0.8
0.2 0.3 0.4 0.5 0.6 0.7 0.8 0.9 1.0

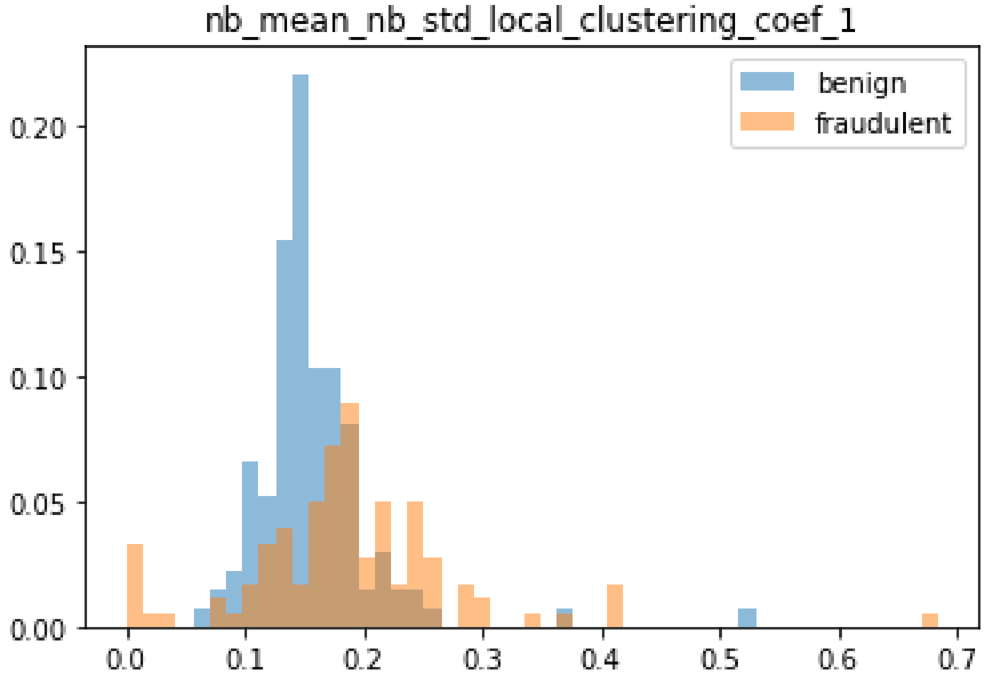
nb_mean_nb_std_local_clustering_coef_1
benign
fraudulent
0.00
0.05
0.10
0.15
0.20
0.0 0.1 0.2 0.3 0.4 0.5 0.6 0.7

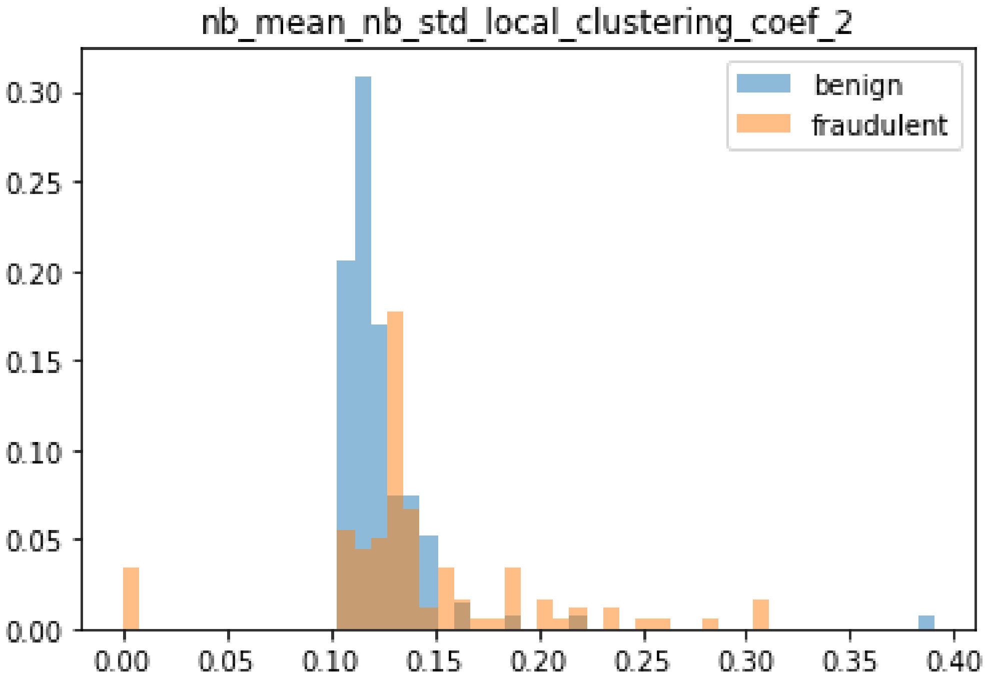

nb_mean_nb_std_local_clustering_coef_2
benign
fraudulent
0.30
0.25
0.20
0.15
0.10
0.05
0.00
0.00
0.05
0.10
0.15
0.20
0.25
0.30
0.35
0.40

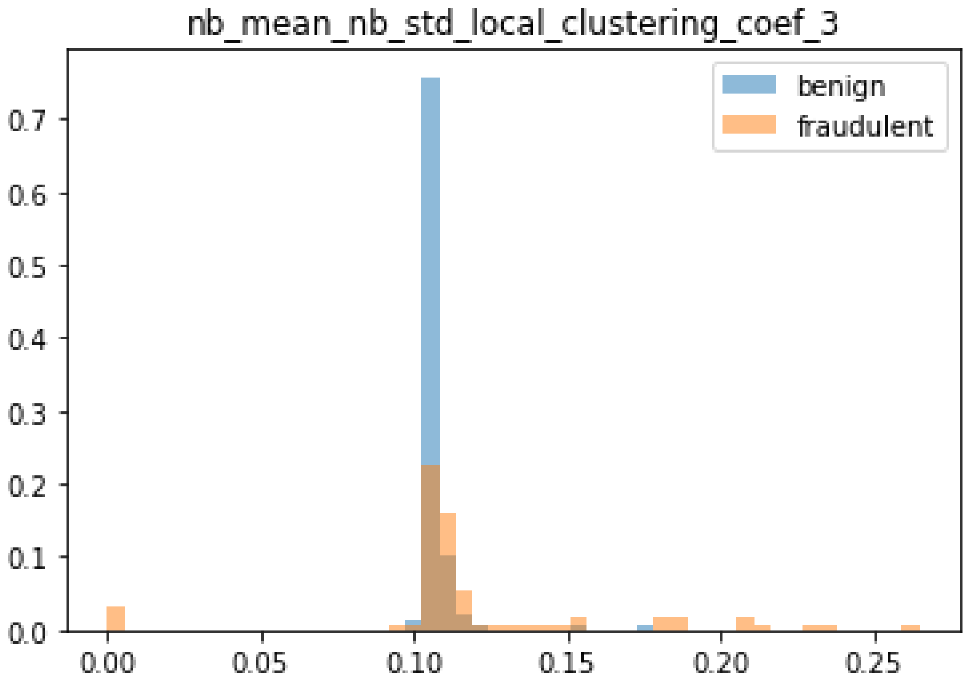

nb_mean_nb_std_local_clustering_coef_3
benign
fraudulent
0.7
0.6
0.5
0.4
0.3
0.2
0.1
0.0
0.00
0.05
0.10
0.15
0.20
0.25

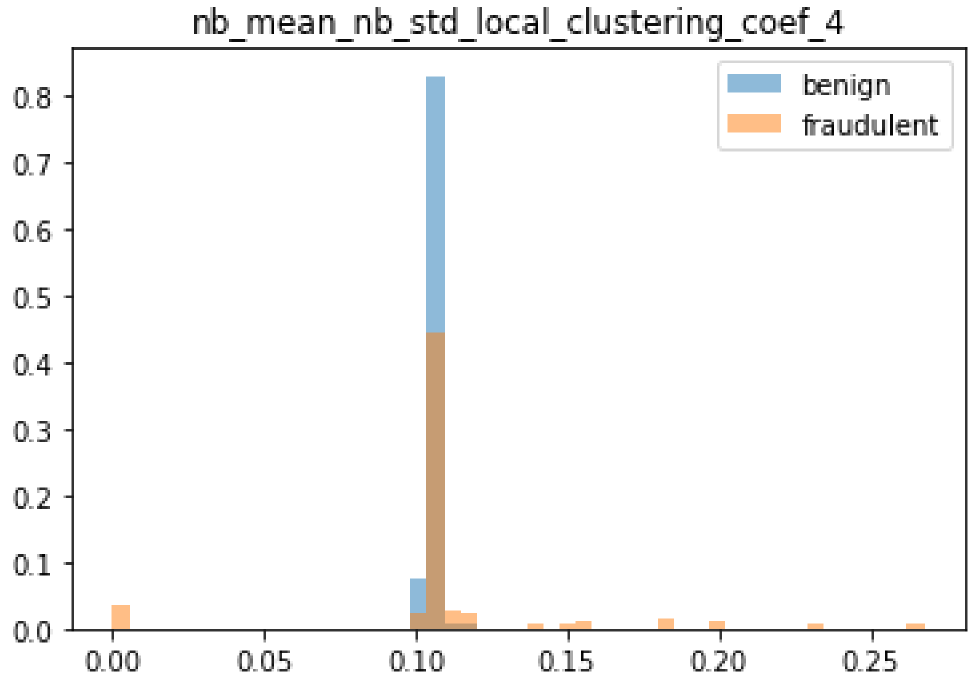
nb_mean_nb_std_local_clustering_coef_4
benign
fraudulent
0.0
0.1
0.2
0.3
0.4
0.5
0.6
0.7
0.8
0.00
0.05
0.10
0.15
0.20
0.25
