# Supplementary material for: Detecting anomalies in graph networks on digital markets
Source: PLoS One. 2024 Dec 23;19(12):e0315849. doi: 10.1371/journal.pone.0315849 (PMC11666048; doi:10.1371/journal.pone.0315849)

02_Bitcoin_OTC_dimensionality

August 19, 2023

[1]:
```
import pandas as pd
import matplotlib.pyplot as plt
import itertools
```

1 Dimension and compression vs model performance

1.1 Bitcoin OTC

[2]:
```
dataset_nam = "bitcoin_otc"

y_test = pd.read_excel(f"./dimensionality_input/{dataset_nam}/results/ytest.
 ↪xlsx"
                      ).drop(columns=["Unnamed: 0", "index"], errors="ignore")

results = pd.read_csv(f"./dimensionality_input/{dataset_nam}/results/
 ↪results_aggregated.csv",
                          index_col = 0)
```

[3]:
```
# UMAP: only seed 0 for readability

a = results["embedding_type"].unique().tolist()
a.remove("graph_features")
a.remove("all_embeddings")
a

for model, metric, embedding_type, compression_name in list(
    itertools.product(results["model"].unique(),
                      #["acc", "f1", "mcc", "auc"],
                      ["f1"],
                      a,
                      results["compression_name"].unique())):

    if compression_name == "no_compression":
        pass
    else:
        print(f"------------- \n {model}, {metric}, {embedding_type},␣
 ↪{compression_name}")
```

```
    temp = results[results["compression_name"].isin(["no_compression",␣
↪compression_name])]
    temp = temp[(temp["embedding_type"] == embedding_type) & (temp["model"]␣
↪== model)]
    if compression_name == "umap":
        temp = temp[(temp["seed"] == 0) | (temp["seed"].isna())]

    temp2 = temp.sort_values(by="original_dim_number", ascending=True)

    fig, ax = plt.subplots(figsize=(12,8))
    plt.scatter(temp2["original_dim_number"], temp2[metric], label =␣
↪temp2["embedding_name"])
    plt.title(f"Metric: {metric} vs embedding dimension ({embedding_type},␣
↪{compression_name}, {model} model)")
    plt.xlabel("original dim number")
    plt.ylabel(metric)

    for index in range(len(temp2["original_dim_number"])):
        ax.text(temp2["original_dim_number"].iloc[index],
                temp2[metric].iloc[index],
                temp2["embedding_name"].iloc[index], size=10)
    plt.show()
```

```
-------------
 h2o, f1, rolx, pca
```

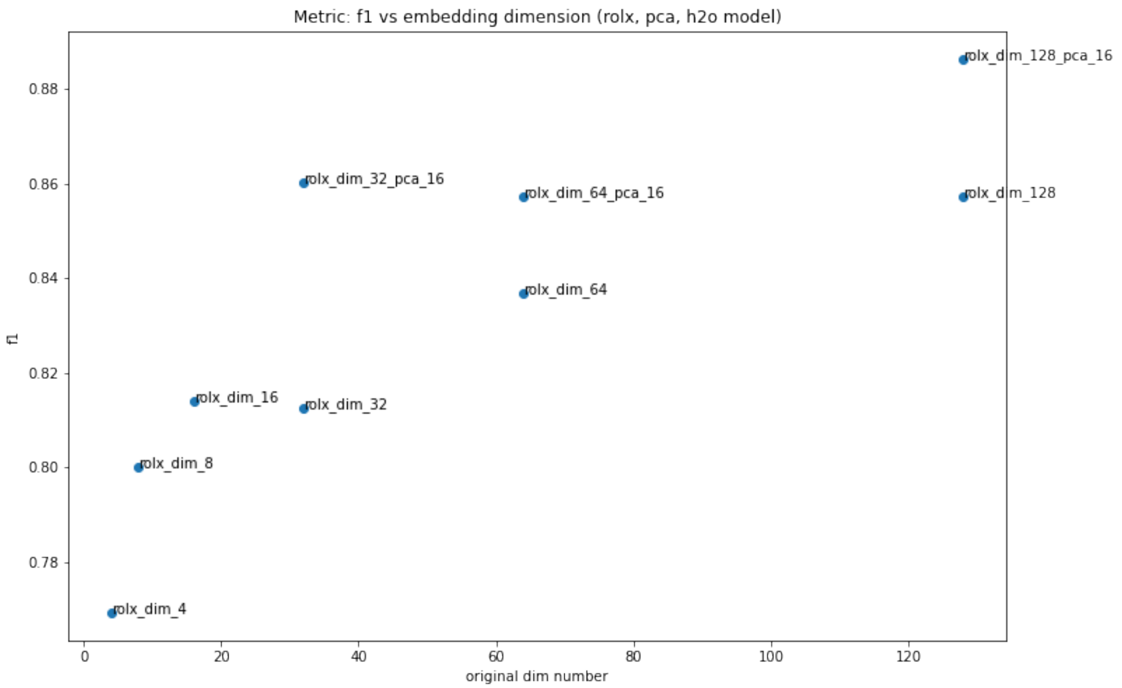

----

h2o, f1, rolx, umap

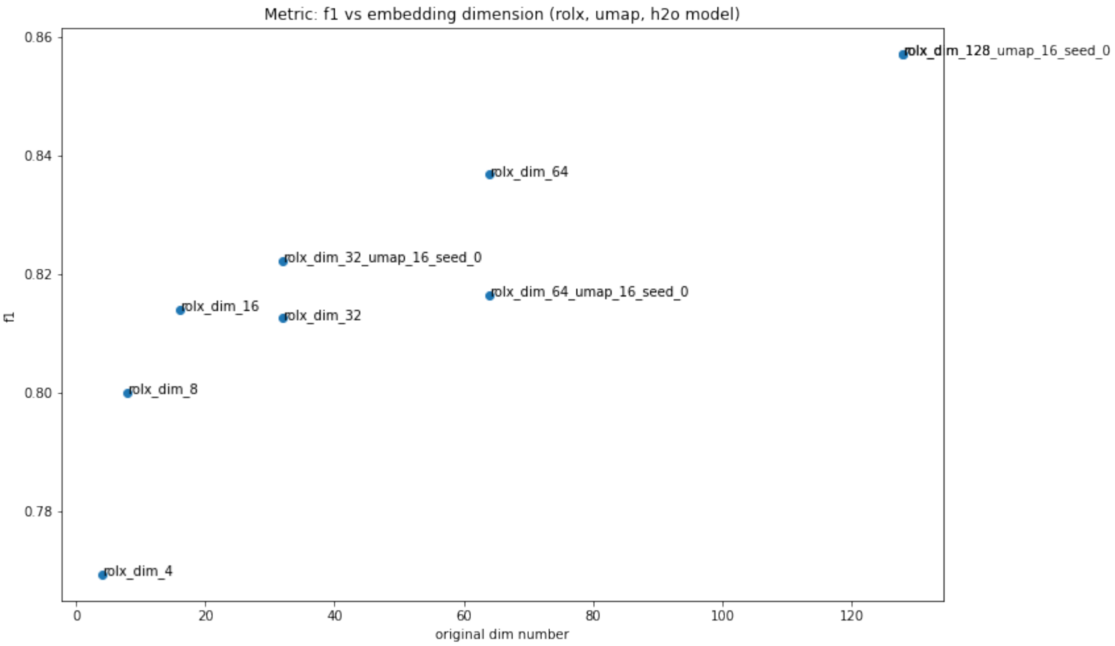

----

h2o, f1, struc2vec, pca

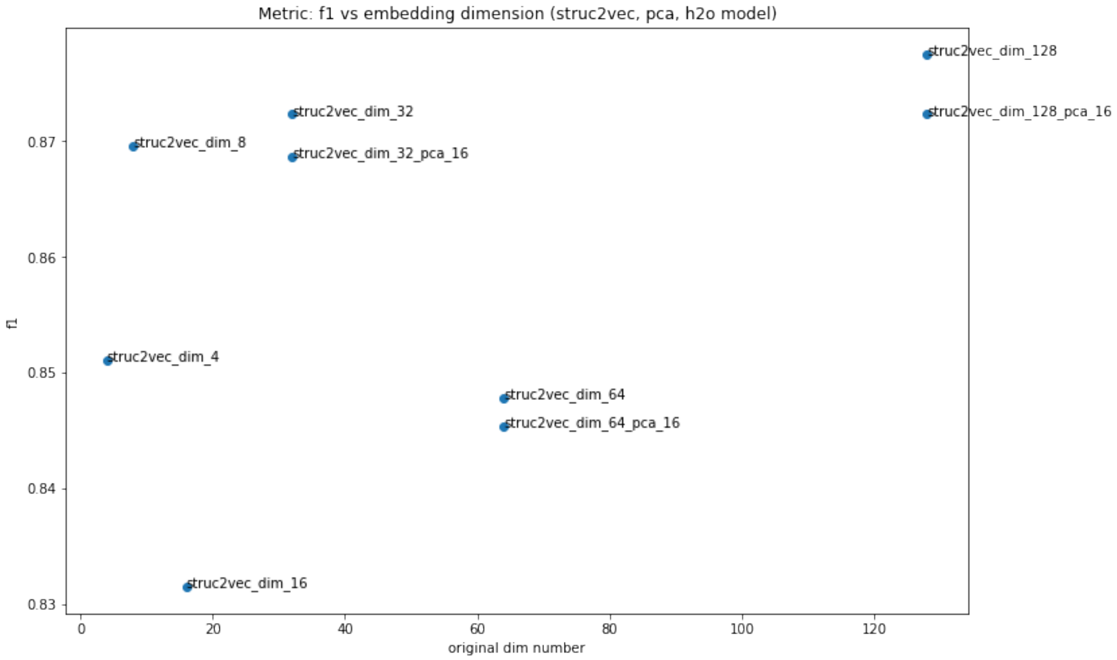

----

h2o, f1, struc2vec, umap

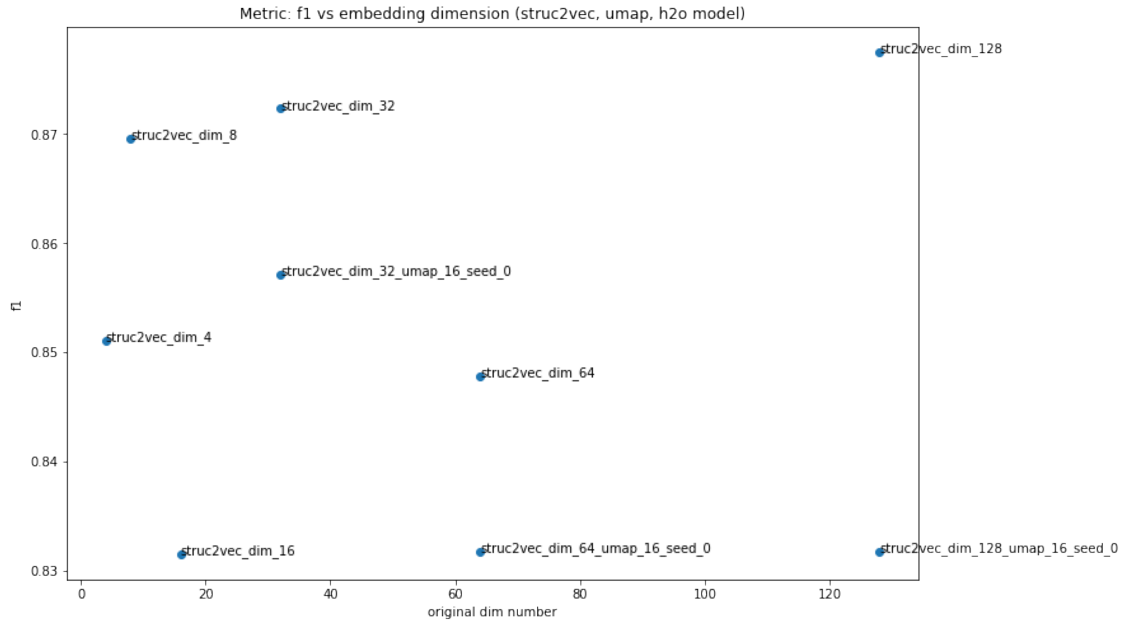

----

h2o, f1, pygn_degree, pca

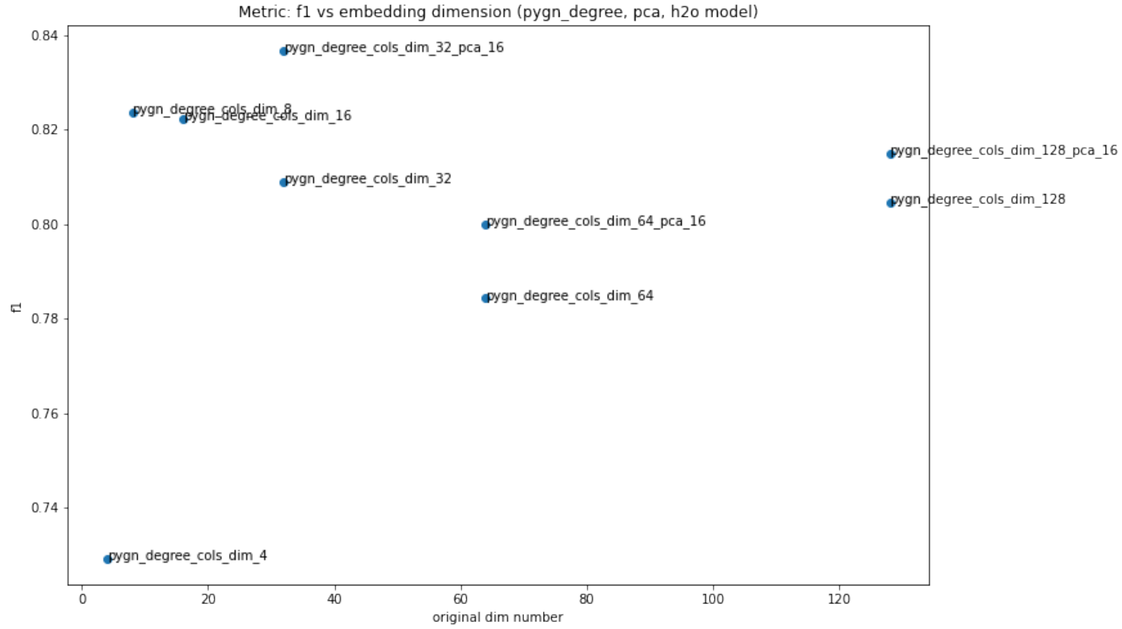

----

h2o, f1, pygn_degree, umap

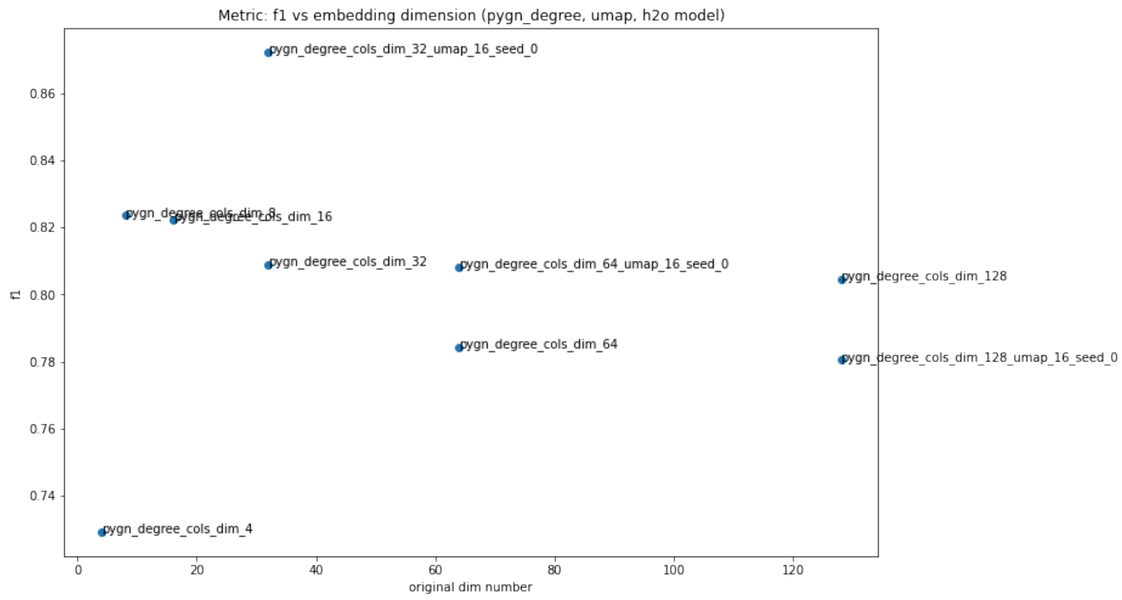

----

h2o, f1, pygn_gf, pca

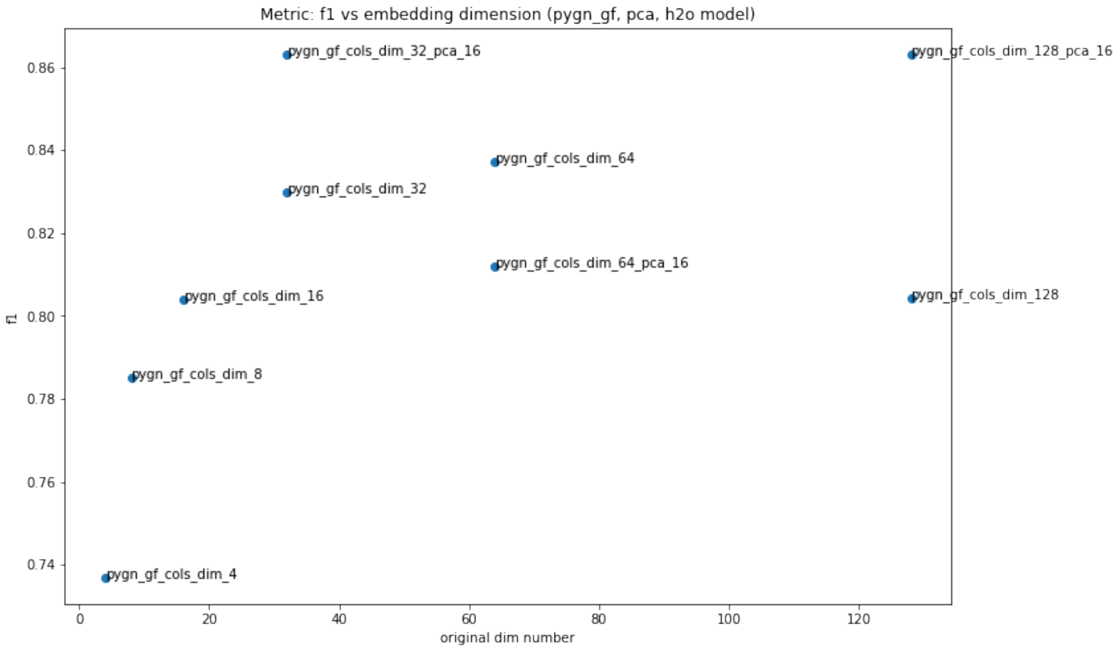

----

h2o, f1, pygn_gf, umap

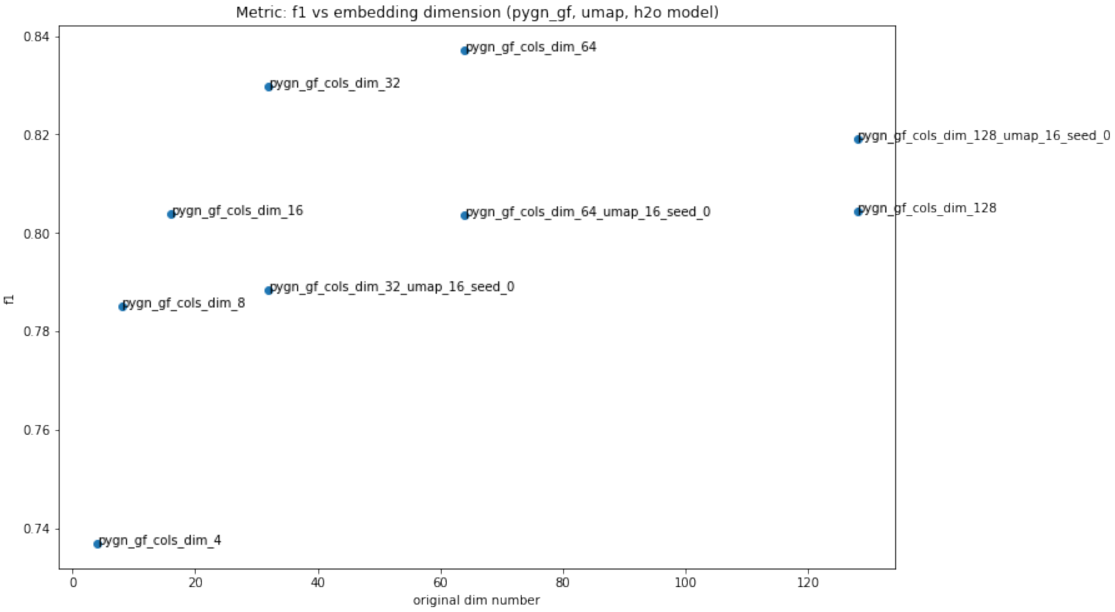

----

h2o, f1, pygn, pca

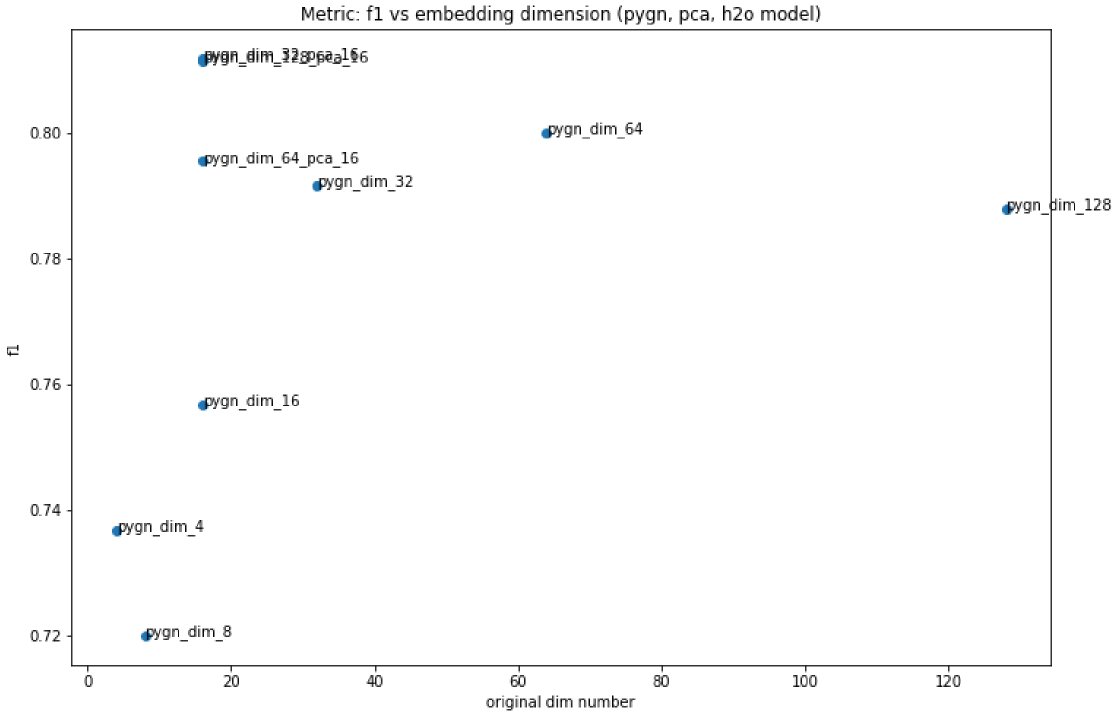

----

h2o, f1, pygn, umap

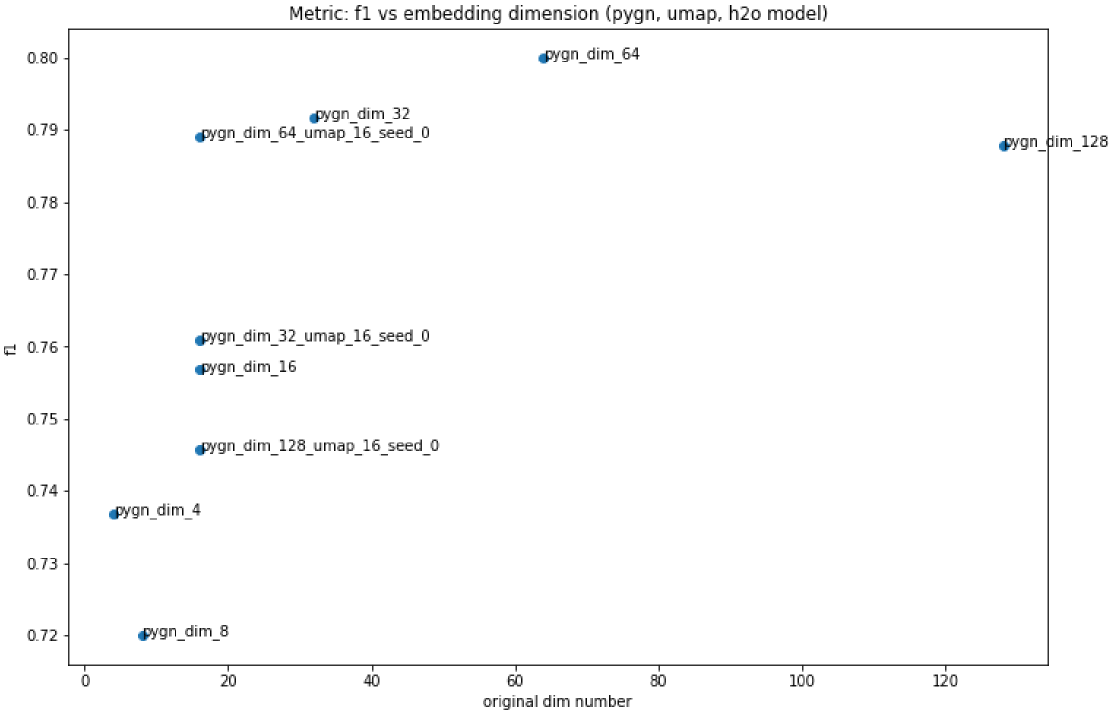

----

h2o, f1, node2vec, pca

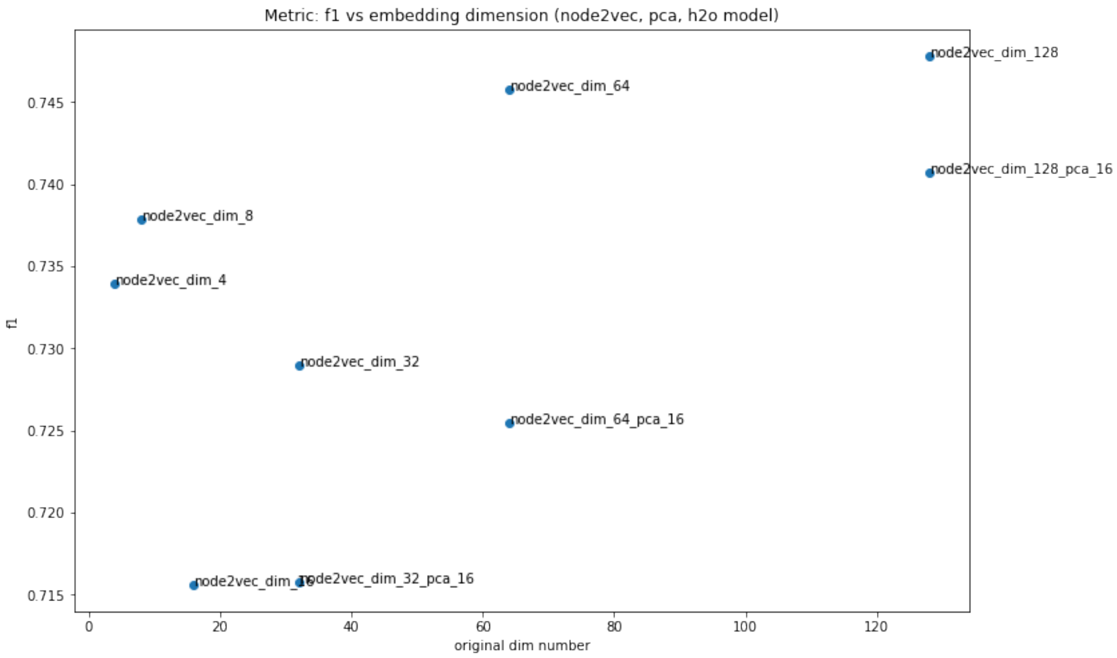

----

h2o, f1, node2vec, umap

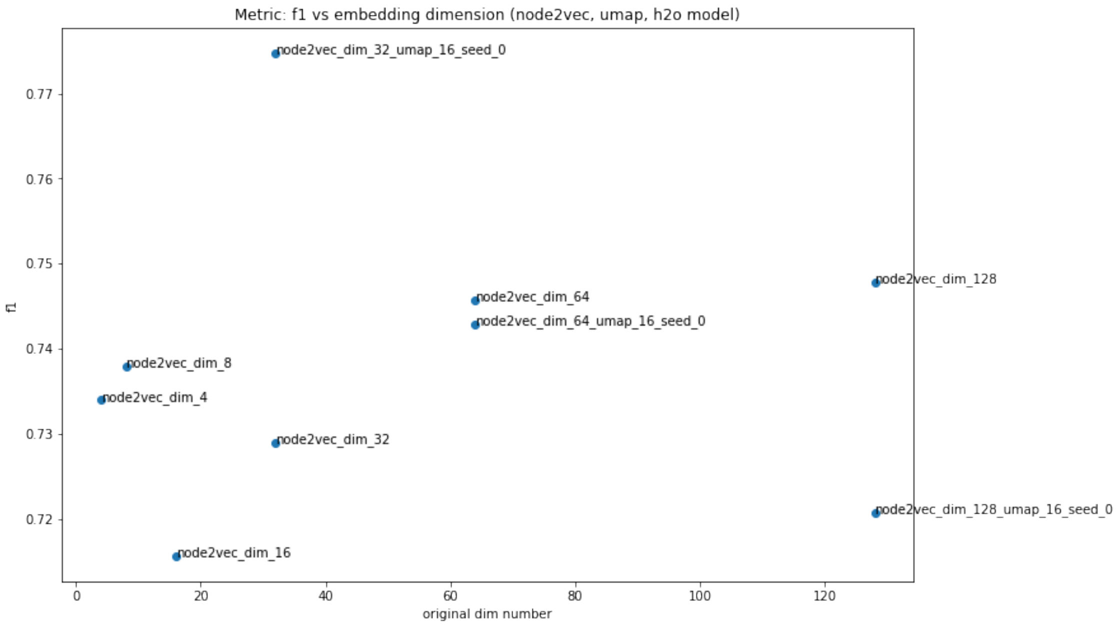

----

h2o, f1, deepwalk, pca

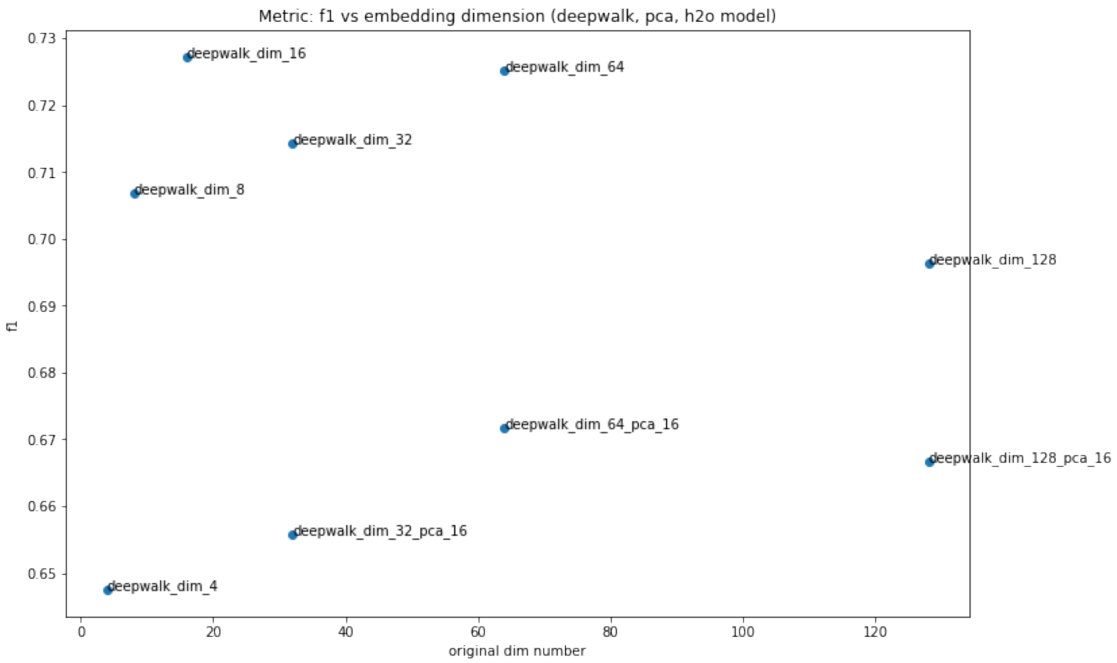

----

h2o, f1, deepwalk, umap

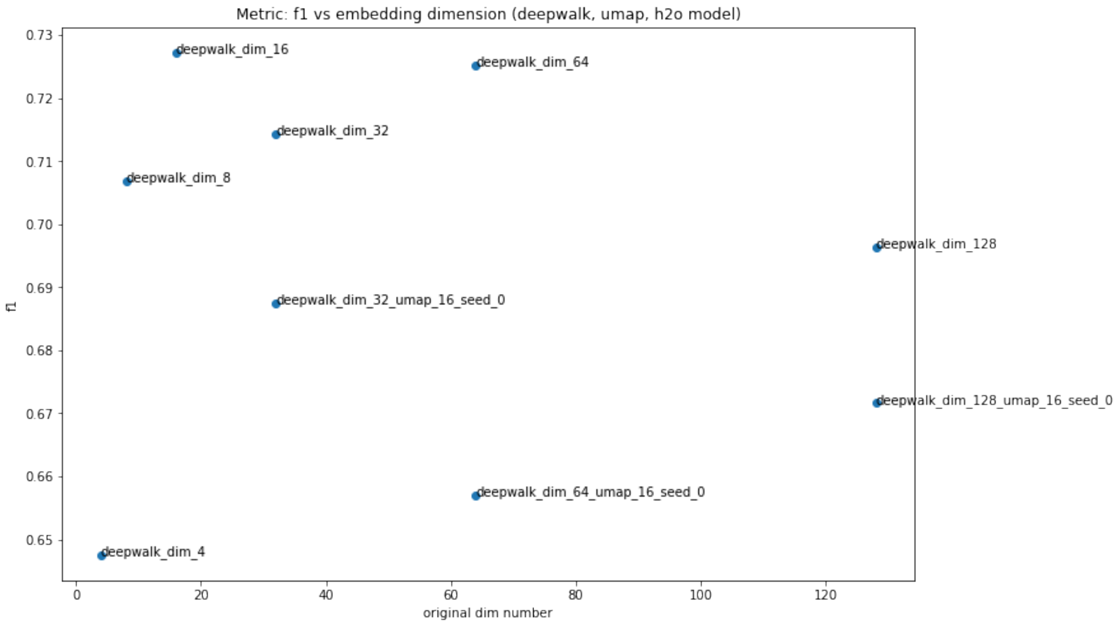

----

xgboost, f1, rolx, pca

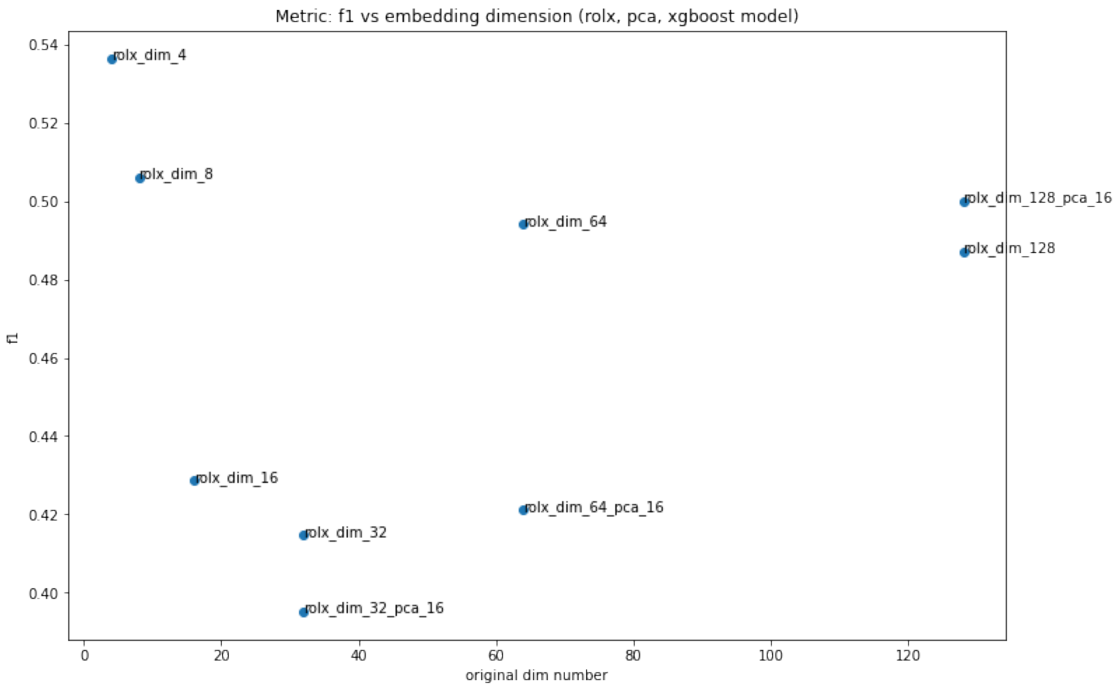

----

xgboost, f1, rolx, umap

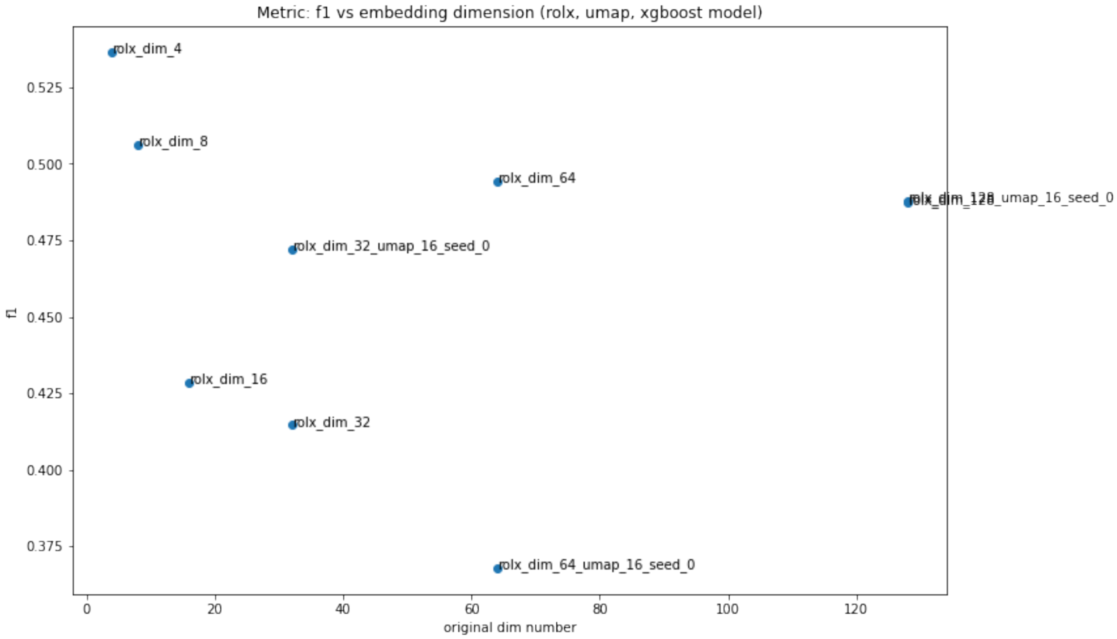

----

xgboost, f1, struc2vec, pca

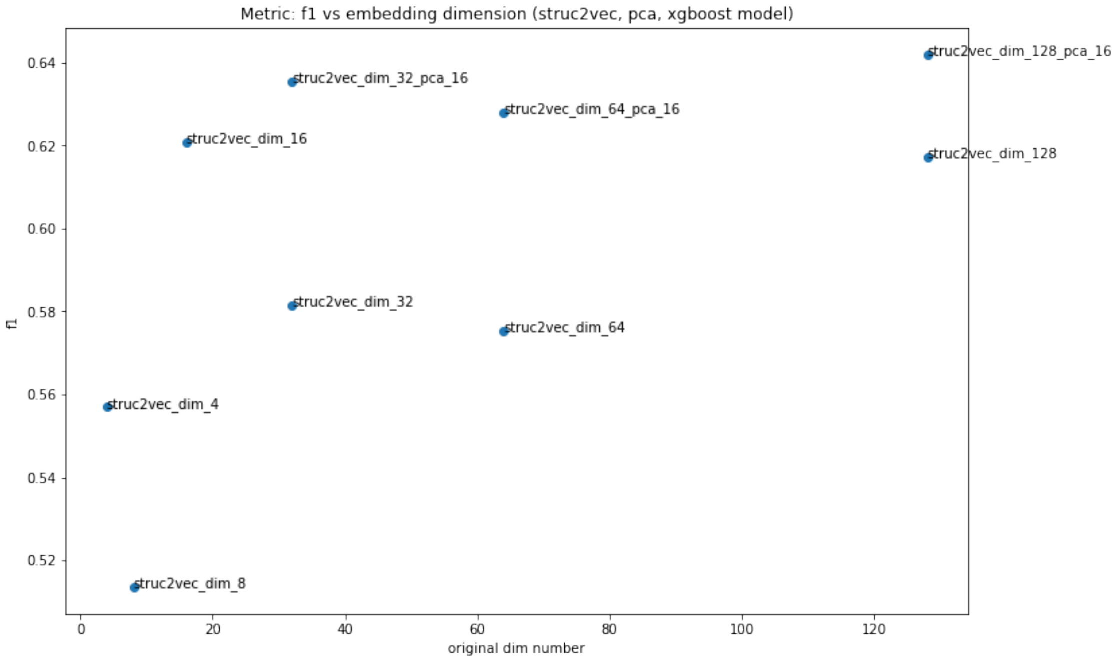

----

xgboost, f1, struc2vec, umap

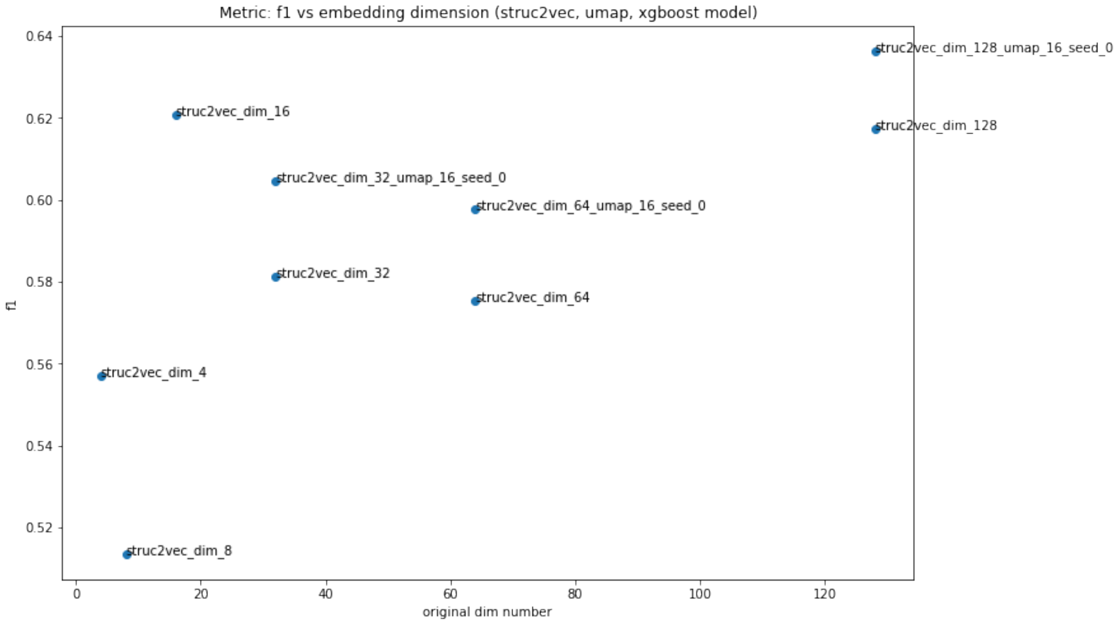

----

xgboost, f1, pygn_degree, pca

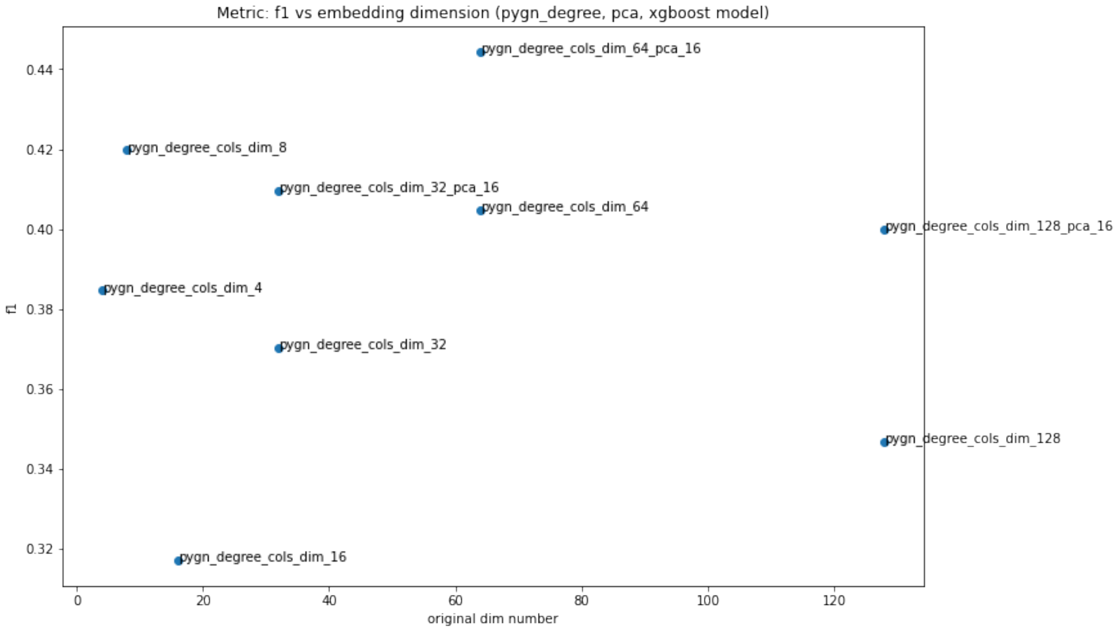

----

xgboost, f1, pygn_degree, umap

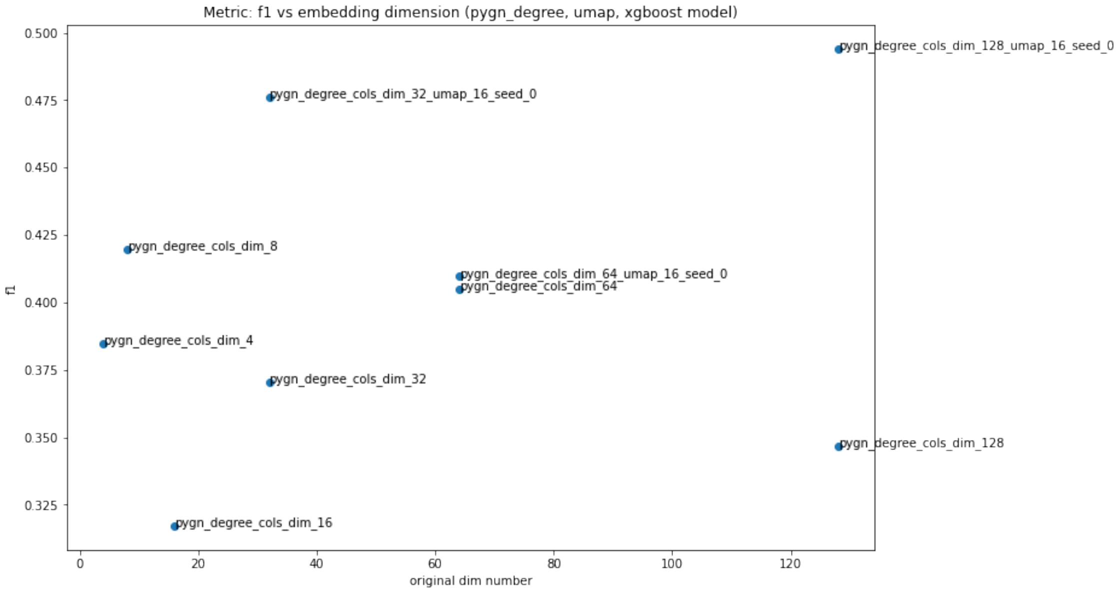

----

xgboost, f1, pygn_gf, pca

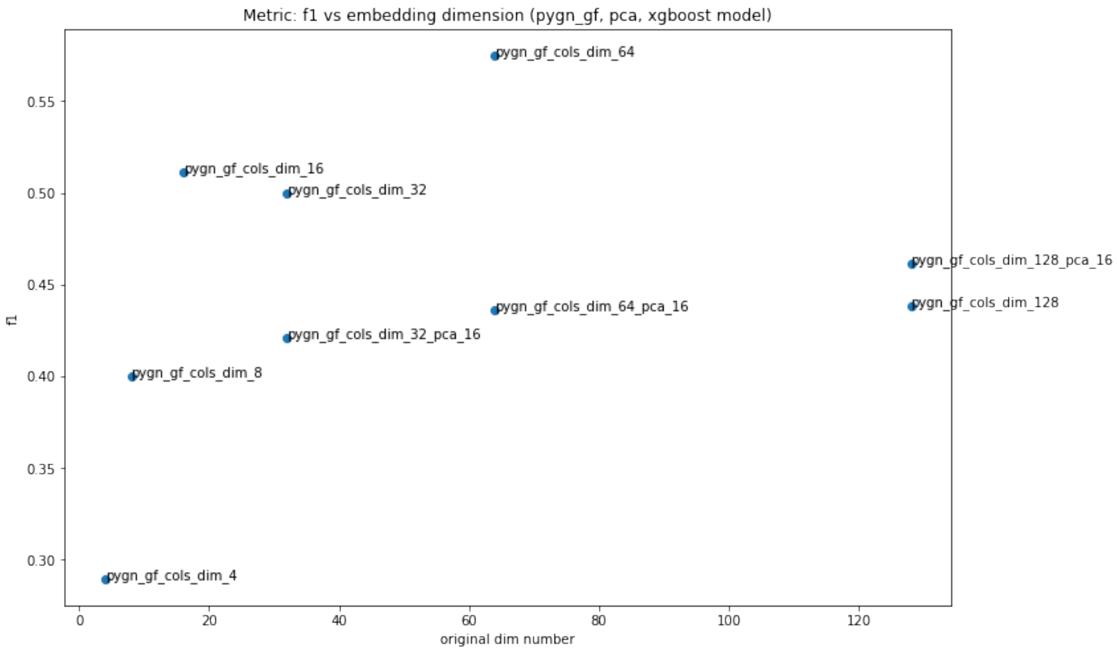

----

xgboost, f1, pygn_gf, umap

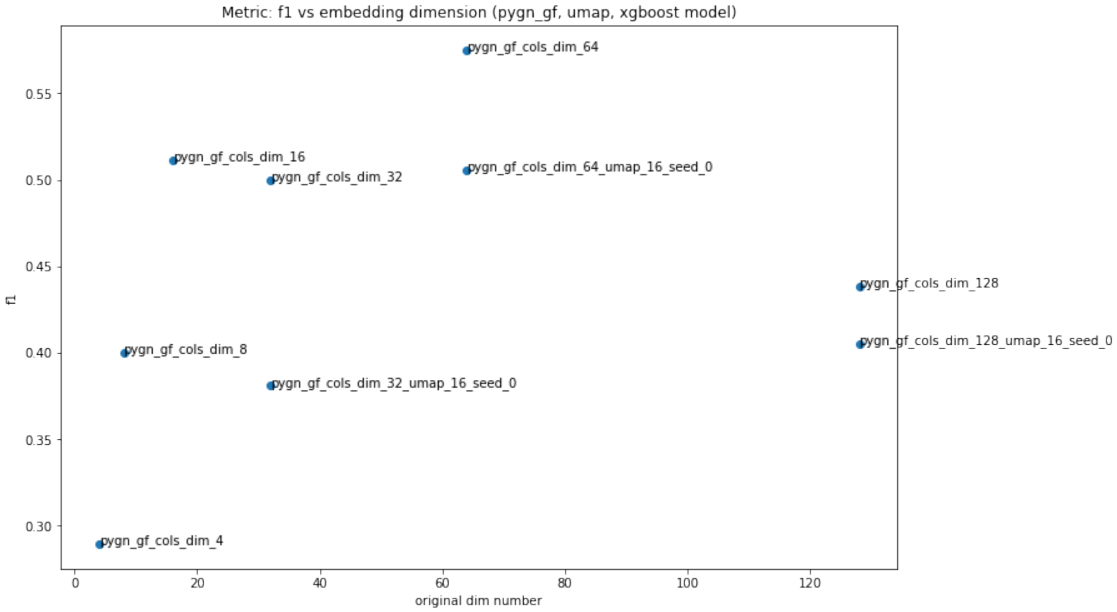

----

xgboost, f1, pygn, pca

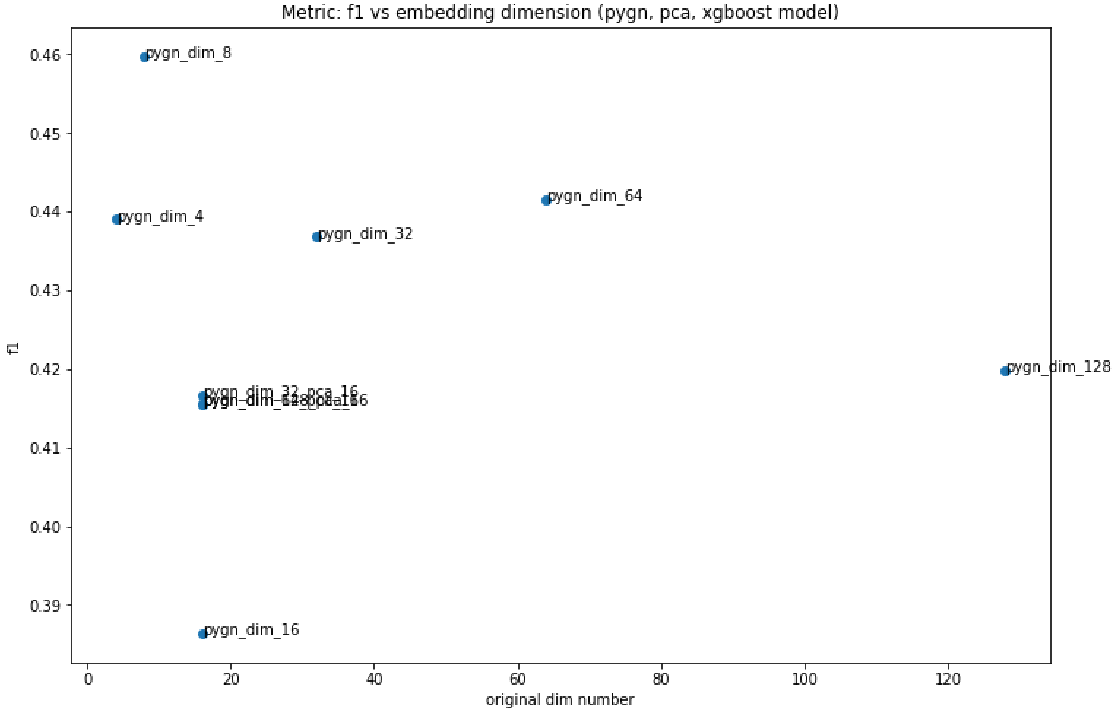

----

xgboost, f1, pygn, umap

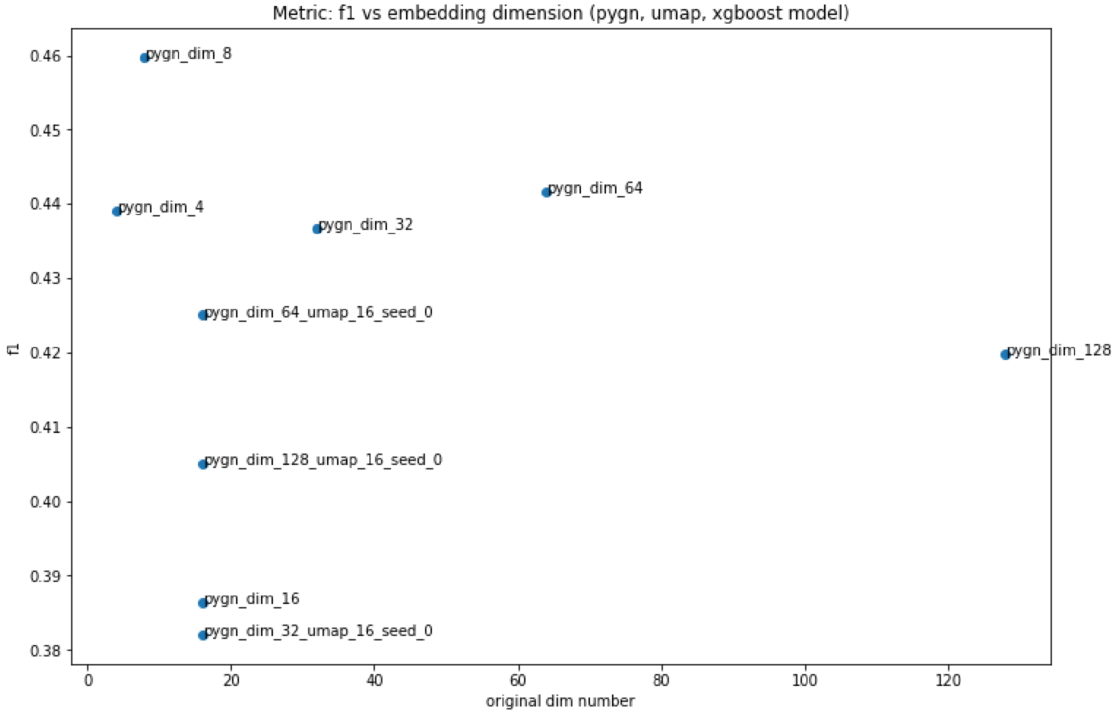

----

xgboost, f1, node2vec, pca

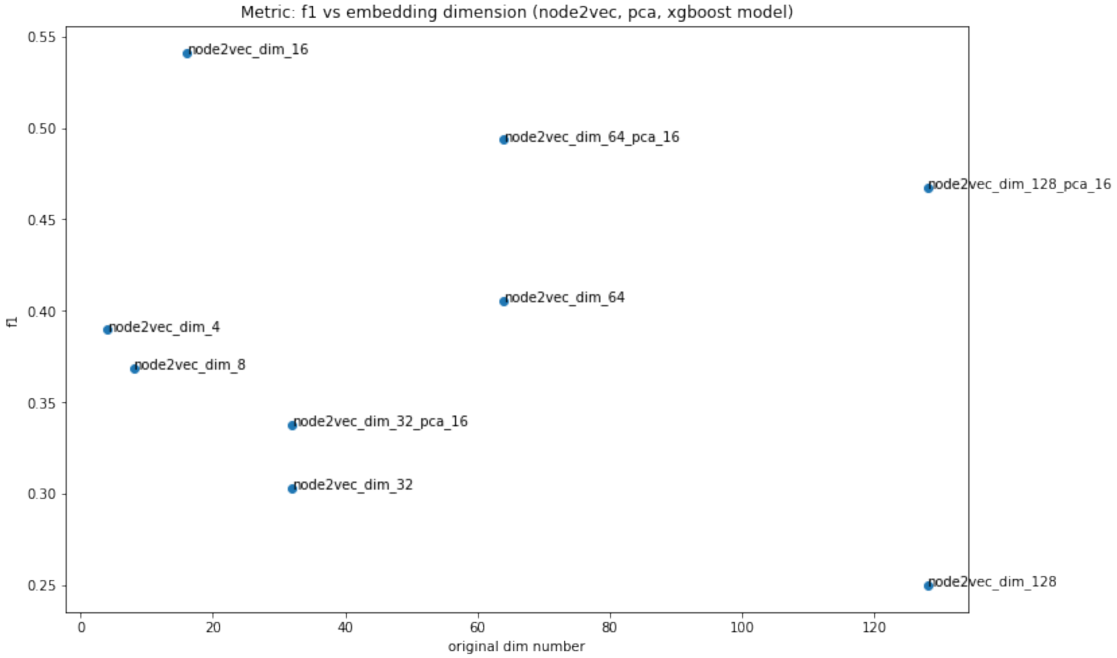

----

xgboost, f1, node2vec, umap

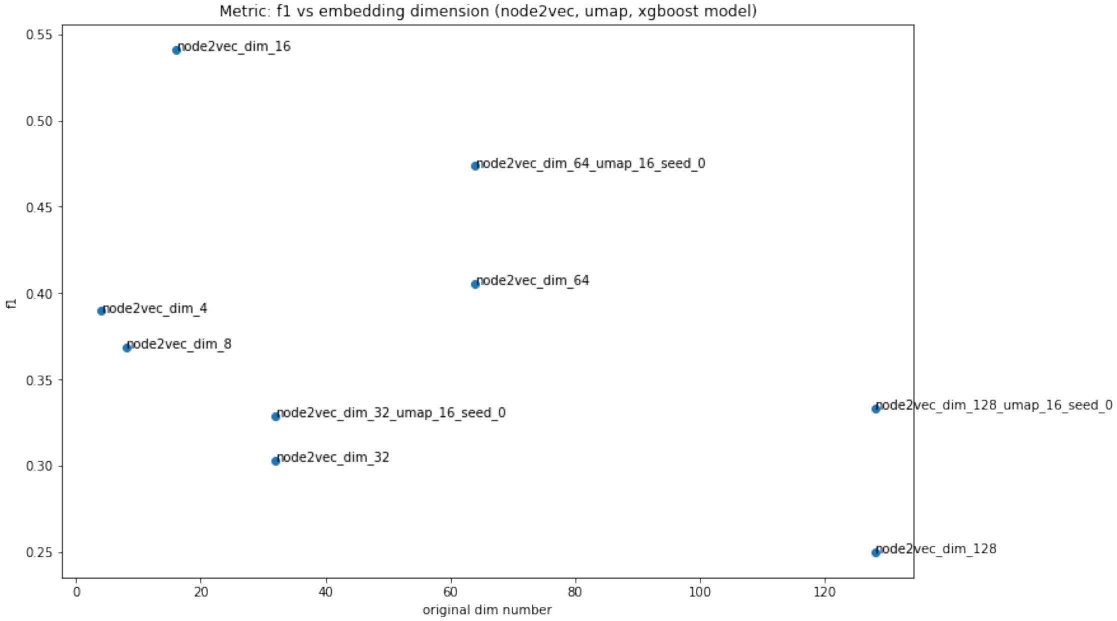

----

xgboost, f1, deepwalk, pca

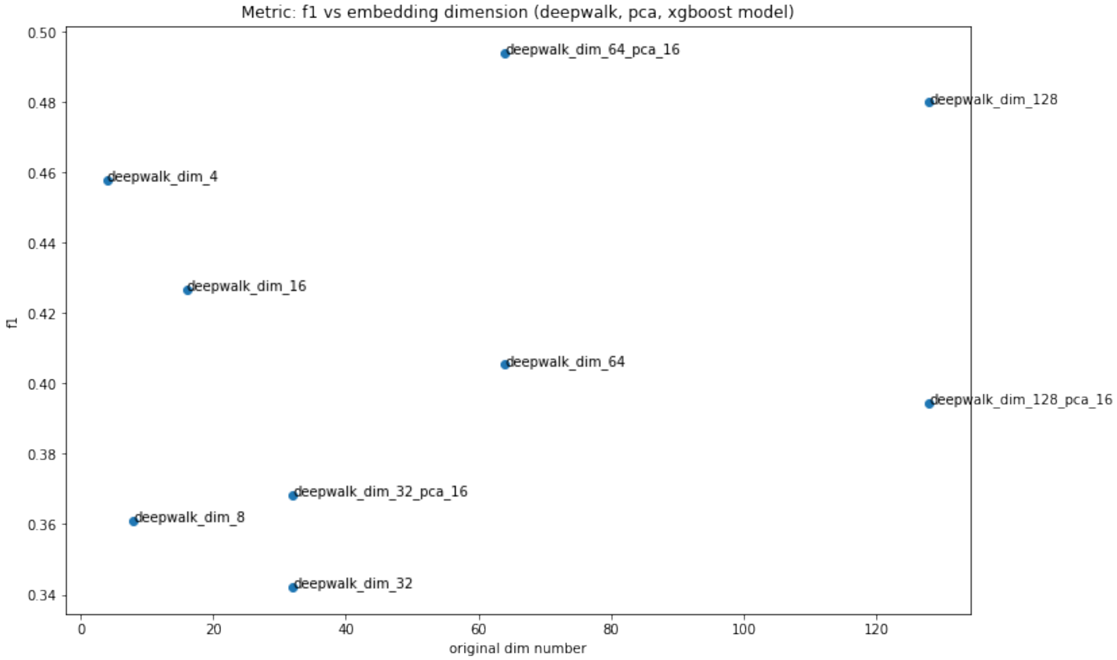

----

xgboost, f1, deepwalk, umap

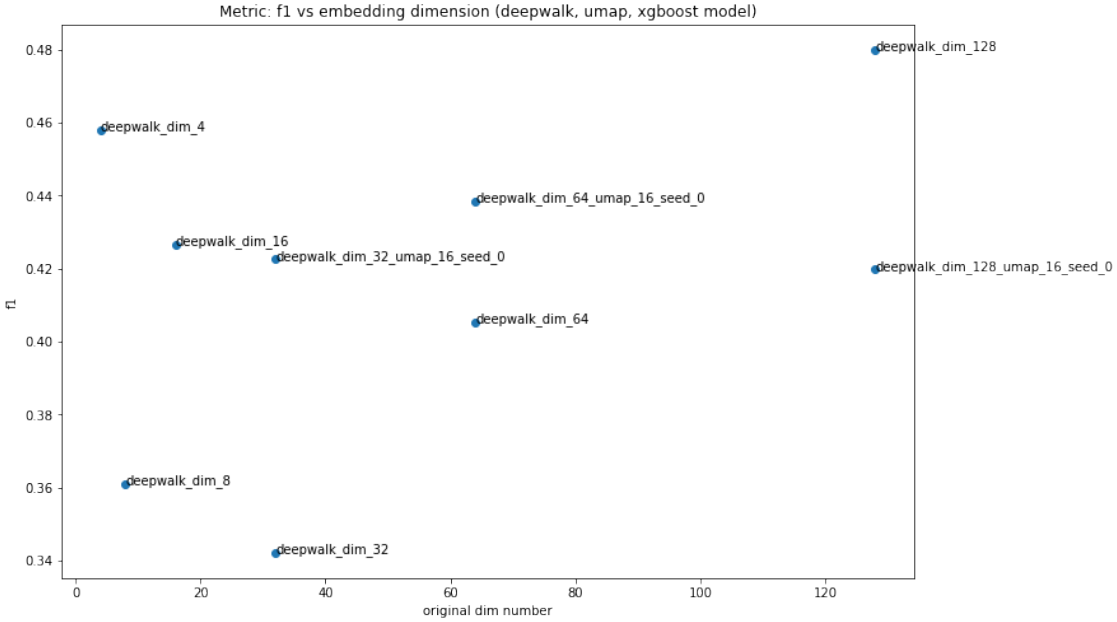

Supplement: S1 Appendix — The Technical appendix can be found under: https://www.kaggle.com/datasets/agatasko/tech-appendix. List of supplements: plots:a. 01_TwiBot_20_histograms.htmlb. 02_Bitcoin_OTC_histograms.htmlc. 03_Bitcoin_Alpha_histograms.htmld. 04_TwiBot_20_dimensionality.htmle. 05_Bitcoin_OTC_dimensionality.htmlf. 06_Bitcoin_Alpha_dimensionality.htmltables:a. 01_TwiBot_20_statistics.csvb. 02_Bitcoin_OTC_statistics.csvc. 03_Bitcoin_Alpha_statistics.csvd. 04_TwiBot_20_results.csve. 05_Bitcoin_OTC_results.csvf. 06_Bitcoin_Alpha_results.csvg. 07_TwiBot_20_compression_results.csvh. 08_Bitcoin_OTC_compression_results.csvi. 09_Bitcoin_Alpha_compression_results.csv (ZIP) [file pone.0315849.s001.zip › tech_appendix/plots/pdf/05_Bitcoin_OTC_dimensionality.pdf]
